# Functional Changes in Brain Activity Using Hypnosis: A Systematic Review

**DOI:** 10.3390/brainsci12010108

**Published:** 2022-01-13

**Authors:** Thomas Gerhard Wolf, Karin Anna Faerber, Christian Rummel, Ulrike Halsband, Guglielmo Campus

**Affiliations:** 1 Department of Restorative, Preventive and Pediatric Dentistry, School of Dental Medicine, University of Bern, CH-3010 Bern, Switzerland; karin.faerber@students.unibe.ch (K.A.F.); guglielmo.campus@zmk.unibe.ch (G.C.); 2 Department of Periodontology and Operative Dentistry, University Medical Center of the Johannes Gutenberg University Mainz, D-55131 Mainz, Germany; 3 Support Center for Advanced Neuroimaging (SCAN), University Institute of Diagnostic and Interventional Neuroradiology, Bern University Hospital (Inselspital), University of Bern, CH-3010 Bern, Switzerland; christian.rummel@insel.ch; 4 Department of Psychology, Neuropsychology, University of Freiburg, D-79085 Freiburg im Breisgau, Germany; ulrike.halsband@gmx.de; 5 Department of Surgery, Microsurgery and Medicine Sciences, School of Dentistry, University of Sassari, I-07100 Sassari, Italy; 6 Department of Pediatric, Preventive Dentistry and Orthodontics, School of Dentistry, Sechenov First Moscow State Medical University, 119991 Moscow, Russia

**Keywords:** brain activity, CT, EEG, functional changes, fMRI, imaging technique, hypnosis, PET, SPECT, systematic review

## Abstract

Hypnosis has proven a powerful method in indications such as pain control and anxiety reduction. As recently discussed, it has been yielding increased attention from medical/dental perspectives. This systematic review (PROSPERO-registration-ID-CRD42021259187) aimed to critically evaluate and discuss functional changes in brain activity using hypnosis by means of different imaging techniques. Randomized controlled trials, cohort, comparative, cross-sectional, evaluation and validation studies from three databases—Cochrane, Embase and Medline via PubMed from January 1979 to August 2021—were reviewed using an ad hoc prepared search string and following the Preferred Reporting Items for Systematic Review and Meta-Analyses (PRISMA) guidelines. A total of 10,404 articles were identified, 1194 duplicates were removed and 9190 papers were discarded after consulting article titles/abstracts. Ultimately, 20 papers were assessed for eligibility, and 20 papers were included after a hand search (*n*^total^ = 40). Despite a broad heterogenicity of included studies, evidence of functional changes in brain activity using hypnosis was identified. Electromyography (EMG) startle amplitudes result in greater activity in the frontal brain area; amplitudes using Somatosensory Event-Related Potentials (SERPs) showed similar results. Electroencephalography (EEG) oscillations of θ activity are positively associated with response to hypnosis. EEG results showed greater amplitudes for highly hypnotizable subjects over the left hemisphere. Less activity during hypnosis was observed in the insula and anterior cingulate cortex (ACC).

## 1. Introduction

Hypnosis is defined as “a state of consciousness involving focused attention and reduced peripheral awareness characterized by an enhanced capacity for response to suggestion,” according to the American Psychological Association (APA) Division 30 [1]. Hypnosis changes the state of consciousness of a person and allows unconscious experiences to become a modified way of looking at reality [2]. Judging by empirical evidence of its effectiveness in clinically diverse fields of application, hypnosis is furthermore described as an amount of biological, cognitive, and social perspectives [3]. In the state of hypnotic trance, control of the consciousness is in the background, modified by attention, concentration and letting go of thoughts while access to the unconsciousness is created [4,5]. Neutral hypnosis involves states of relaxation in which the subject responds only to important or particularly strong environmental stimuli and has reduced perception to peripheral stimuli [1,2]. Increasing activation in the visual center under hypnosis is related to a subjectively perceived degree of relaxation [2]. This means that the degree of subjectively perceived relaxation is usually greater in deep hypnosis than in a light hypnotic trance because the focus of attention on the inner experience is increased, allowing an increased capacity for responses to suggestion [1,2]. Hypnosis illustrates that the intervention modulates attentional control, which modifies emotions and the nervous system and interacts with past experiences in the subconscious. Suggestions during hypnosis can cause dynamic changes in brain activity [6]. Areas responsible for processing cognition and emotion show greater activity during hypnosis, as well as hypnosis-induced changes in functional connectivity between anterior cingulate cortex (ACC) and the large neural network [4,7]. Dynamic changes and connectivity in brain activity occurs not only during suggestion of analgesia but also naturally in the awake state [8].

With various imaging methods, such as EEG (electroencephalography), fMRI (functional magnetic resonance imaging), fNIRS (near-infrared spectroscopy), PET (positron emission tomography), SPECT (single-photon emission computed tomography) and CT (computer tomography), functional, metabolic and structural information about the brain can be obtained. Using hypnosis, brain activity can be demonstrated using these imaging methods.

The aim of the present study was to perform a systematic review of articles published in the last four decades, investigating the functional changes in brain activity using hypnosis by means of different imaging methods. A broad review of the use of different imaging methods demonstrating plastic brain-activity changes during hypnosis should be provided to gain information for future study designs and to review whether similarities or differences exist regarding medical applications in the literature. The hypothesis of this systematic review is that differences can be observed in functional changes in brain activity when comparing the normal/resting state and the hypnotic state.

## 2. Materials and Methods

### 2.1. Review Design

The review protocol was registered in the international prospective register of systematic reviews, PROSPERO, on 5 July 2021 with ID-CRD42021259187 (https://www.crd.york.ac.uk/prospero; last access: 12 January 2022). The Preferred Reporting Items for Systematic Reviews and Meta-Analyses (PRISMA) were adopted throughout the process of the present systematic review [9]. The inclusion and exclusion criteria and research questions were organized following PICOS guidelines [10]:

Population: Adults of all genders (>18 years)
Intervention: Medical subject headings (MeSH) terms and keywords related to the topic studied were applied. The Study Quality-assessment tool (https://www.nhlbi.nih.gov/health-topics/study-quality-assessment-tools; last access: 12 January 2022) of the National Heart, Lung and Blood Institute was used to rate the quality of the included papers.
Comparator: Different imaging techniques that show different functional changes in brain activity: computer tomography (CT), electroencephalogram (EEG), electromyogram (EMG), electrooculogram (EOG), functional magnetic resonance imaging (fMRI), functional near-infrared spectroscopy (fNIRS), magnetic resonance imaging (MRI), positron emission tomography (PET), regional cerebral blood flow (rCBF) and single-photon emission computer tomography (SPECT).
Outcome: The quality-control tool of the National Herat, Lung and Blood Institute included criteria about adequate randomization, participation rate, similarity of groups/population and adherence to the intervention protocols, as well as sources of bias (publication bias, eligible persons, exposure measures, blinding, validity, selection bias, information bias, etc.). For each part, yes/no/cannot determine was selected. In the end, each study/paper was scored as good if the study had the least risk for bias, fair if the study was predisposed to some bias and poor if it was possible that the study was biased.

### 2.2. Eligibility Criteria

In this systematic review, randomized controlled trials, cohort, comparative, cross-sectional, evaluation and validation studies reporting brain-activity changes using hypnosis by means of imaging methods such as CT, EEG, EMG, EOG, fMRI, fNIRS, MRI PET, rCBF or SPECT were reviewed. Only human studies published in English from 1 January 1979 to 31 August 2021 were collected and evaluated.

### 2.3. Information Sources

Electronic databases, such as Cochrane, Embase and MEDLINE via PubMed, were taken into consideration and screened for articles. Grey literature was retrieved via opengrey.eu (http://www.opengrey.eu; last access: 12 January 2022).

### 2.4. Information Sources and Search Strategy

Several search strategies were applied. The following search string with medical subject headings (MeSH) terms and keywords was used: (hypnosis OR hypnotizability OR hypnotic OR suggestibility OR suggestion OR hypnotic state OR consciousness OR susceptibility OR attention OR mental practice OR cognitive task OR resting state OR intention OR loss of control OR awareness of movements OR autogenetic training OR perception OR paralysis OR inhibition OR emotion OR behaviour OR behavior OR possession trance OR passivity OR regulation of consciousness OR attention) AND (dental phobia OR fear OR dental fear OR pain OR dental pain OR acute pain OR chronic pain OR pain threshold OR dental pain threshold OR perception threshold OR hypnotic focused analgesia) AND (EEG OR electroencephalography OR fMRI OR functional magnetic resonance imaging OR fNIRS OR near infrared spectroscopy OR functional near infrared spectroscopy OR PET OR positron emission tomography OR SPECT OR single photon emission computed tomography OR CT OR computer tomography OR regional cerebral blood flow OR neuroimaging OR structural and functional cerebral correlate OR functional connectivity OR local neuronal activity OR functional brain activity change OR brain activity OR cerebral somatic pain modulation OR brain imaging OR mental imagery OR resting-state functional connectivity OR cerebral hemodynamics OR affective neurofeedback OR feedback effect OR whole-connectivity profile OR voxel based morphometry). Using the bibliographies of full-text articles, cross-referencing was performed. Via opengrey.eu, grey literature was also retrieved.

### 2.5. Study Selection

After the comparison of the various string research studies with cross-referencing, all duplicates were excluded. Titles and abstracts of all references were read independently by two authors (K.A.F. and T.G.W.). The full texts of articles with titles and abstracts that appeared to fit the eligibility criteria were then assessed by the same authors. References for which the full texts fulfilled the eligibility criteria were included in this systematic review. Any disagreement in the selection process was resolved in a discussion between peers. In the case of continuous controversy, a third author (G.C.) was consulted.

### 2.6. Data Collection, Summary Measures and Synthesis of Results

For each included reference, data collection and synthesis were carried out by two authors (K.A.F. and T.G.W.). The excluded and included articles are summarized in tables (Supplementary Materials S1–S3). The following data were extracted and input in a table: last name of the first author, country where the study was conducted, age, sex, methods used for evaluation of brain activity under hypnosis with different imagine techniques, and results of the comparison. The data were summarized in different tables to make the synthesis easier.

### 2.7. Assessment of Bias across Studies

The quality and choice of the included papers were obtained by two authors (K.A.F. and G.C.) according to the respective customized quality-assessment tool generated by the National Heart, Lung, and Blood Institute for controlled intervention studies, systematic reviews and meta-analyses, observational cohort and cross-sectional studies, case-control studies, pre-post studies with no control group and case series studies, as well as the accompanying quality-assessment tool guidance for assessing the quality of controlled intervention studies (https://www.nhlbi.nih.gov/health-topics/study-quality-assessment-tools; last access: 12 January 2022). Quality control included criteria about adequate randomization, participation rate, similarity of groups/population, adherence to the intervention protocols and sources of bias (publication bias, eligible persons, exposure measures, blinding, validity, selection bias, information bias, etc.). For each part, yes/no/cannot determine was selected. Each study/paper was scored as good if the study had the least risk for bias, fair if the study was predisposed to some bias and poor if it was possible that the study was biased.

## 3. Results

**Figure 1:**
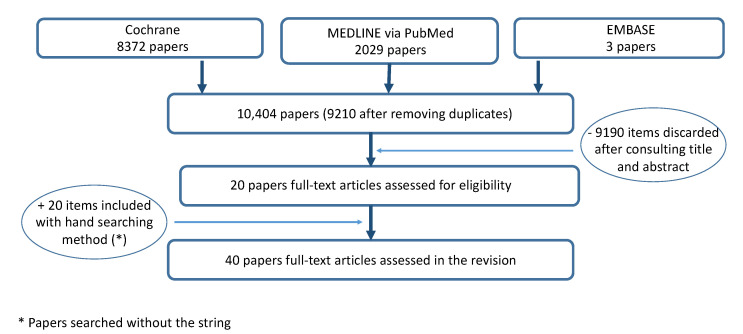
Flow chart of the search.

The search identified 10′404 papers; 9210 were selected after removing 1194 duplicates. A total of 9190 studies were discarded after consulting titles and abstracts. A total of 20 articles were assessed for eligibility, and after evaluating the full text, 20 articles were included. A total of 40 papers were included (Figure 1). Quality-assessment scores of the included papers are listed in Supplementary Materials (Table S2, Quality assessment of included papers). Of the 40 included papers, 7 papers were classified as poor, 15 as fair and 18 as good quality (Table 1). A description of data concerning neutral hypnosis and suggestions for analgesia is provided in Table 2. Table 3 illustrates a description of data obtained in hypnotized and non-hypnotized participants.

Three studies concerning suggestion for analgesia found similar results for highly hypnotizable subjects, all obtained by the EEG method [22,25,48]. An ERP (event-related potential) study showed that placebo analgesia released higher P200 waves in the frontal left hemisphere and during hypnosis, while placebo analgesia involved activity of the left hemisphere, including the occipital region [48]. Pain reduction is related to larger EMG startle amplitudes and N100 and P200 waves, as well as enhanced activity within frontal, parietal, anterior and posterior cingulate gyres [48]. Highly hypnotizable participants had a larger P200 wave than the low-hypnotizability group [48]. A SERP study found that N2 amplitudes in highly hypnotizable subjects were greater over frontal and temporal scalp sites and displayed a larger N2 peak over temporal sites during focused analgesia [22]. Furthermore, a study examining the relationship between pain perception and EEG responses found significant pain and distress reductions in highly hypnotizable subjects for focused analgesia during hypnosis, which indicates a reduction for focused analgesia during hypnosis and post-hypnosis conditions [25].

Two studies from De Pascalis et al. [16,17] concerning suggestion for analgesia reported EEG results for highly hypnotizable subjects. Both studies found greater amplitudes in highly hypnotizable subjects over the left hemisphere [16,17]. The amplitudes in the left hemisphere were greater than those in the right hemisphere (δ, θ_1_, θ_2_, α_1_, β_1_ and β_2_), and during hypnosis/analgesia, highly hypnotizable subjects displayed significant reductions in pain [16]. Highly hypnotizable subjects showed greater θ_1_ amplitude over the left frontal compared to the right hemisphere and in the posterior areas, as well as more θ_1_ activity in the left and right frontal areas and in the right posterior area compared to the low-hypnotizability group [17]. Even for α_1_, more activity was observed in the left hemisphere over the frontal region as well as for α_2_ (greater over the left-frontal compared to the right-frontal site of the scalp [17].

Four studies obtained similar EEG results, three of which collected data concerning neutral hypnosis [15,21,44]. EEG oscillations of θ activity are positively associated with response to hypnosis [15,21,44,46]. θ power is associated with greater response to hypnotic analgesia and individuals who score high on measures of hypnotizability, which means that hypnotic procedures increase θ power [46]. Even more specific EEG findings showed significantly greater high θ activity for the high-hypnotizability group as compared to the low-hypnotizability group at parietal and occipital sites during both hypnosis and waking relaxation conditions [21]. In relation to low-susceptibility participants, high-susceptibility participants had significantly greater θ power during the hypnotic condition, as well as greater α power [44]. Graffin et al. showed that during hypnosis, high-susceptibility subjects had a significant increase in θ power in the more posterior areas of the cortex, and α activity increased among all sites [15].

Another two studies also used the EEG method, and all their data concerned suggestion for analgesia [30,33]. Highly hypnotizable participants had a significant reduction in phase-ordered γ patterns for focused analgesia during hypnosis [25], and θ activity during hypnosis had a peak in the posterior section of the brain, along with lateral shifting [30]. Brain activity changed under hypnosis from the left to the right hemisphere and from the anterior to the posterior brain segment [30].

Two other findings obtained by EEG, with data concerning neutral hypnosis, found higher α duration/more α activity in highly hypnotizable subjects [11,13], which are in agreement with a study mentioned above that used the same method and collected data concerning neutral hypnosis [44].

In order to chart results that were not obtained with an EEG method but by imaging methods, two studies were considered—one with thulium-YAG event-related fMRI and one with fMRI— and during hypnosis, the same activation for the insula could be determined [35,47]. The thulium-YAG event-related fMRI study showed that different regions were less activated during hypnosis compared to normal wakefulness: brainstem, right primary somatosensory cortex and the left and right insula [35]. In the fMRI study, activity was found in the left amygdala and bilaterally in the anterior cingulate cortex (ACC), insula and hippocampus under the fear condition [47]. Under hypnosis, all these areas showed reduced activation [47].

Six other studies obtained using imaging methods, such as PET [18,19], fMRI [27,39,43] and MRI [42], concerned neutral hypnosis (except one) [42] and presented similar outcomes. In hypnotized participants, increased activity in the right ACC was noted in all studies, independent of the method [18,19,27,39,42,43].

Another study with fMRI data and concerning neutral hypnosis found reduced activity in the dorsal anterior cingulate cortex during hypnosis [49]. This study also found increased functional connectivity between the dorsolateral prefrontal cortex and the insula in the salience network during hypnosis, as well as reduced connectivity between the executive control network and the default mode network [49].

In a PET analysis, significant activation could be observed in right-sided extrastriate area and ACC during the hypnotic state [20], while contrary results were observed using event-related fMRI and EEG coherence measures [28]. High-susceptibility subjects showed increased conflict-related neural activity in the ACC under the hypnosis condition [28].

## 4. Discussion

The aim of this study was to ascertain data concerning functional and/or metabolic changes in brain activity using hypnosis by means of different imaging techniques. The most common variables applied in the evaluated papers were high- versus low hypnotizability subjects, as well as hypnosis versus control, and placebo or normal/awake condition.

Owing to the various imaging techniques applied, summarizing the results was demanding. Placebo treatment in waking highly hypnotizable subjects resulted larger P200 waves than in low-hypnotizability groups in EMG, which was associated with pain reduction [49]. Studies examining EEG oscillations of θ activity showed that greater response to hypnosis is associated with reduction in pain, and pain intensity ratings are significantly below pain threshold with exposure to hypnotic analgesia. EEG findings also showed significantly greater θ activity during hypnosis for the high-hypnotizability as compared to low-hypnotizability group at parietal and occipital sites [21]. A recent review not covered in this study also notes that hypnosis is associated more with θ oscillations, while hypnotic response has been shown to be associated with changes in patterns of γ oscillations [51].

Highly hypnotizable subjects displayed significantly lower total and β1 amplitudes in EEG measures, and amplitudes in the left hemisphere were found to be greater than those in the right hemisphere compared to the low-hypnotizabilty group [16]. Highly hypnotizable subjects also showed greater θ1 amplitudes over the left frontal region compared to the right hemisphere and in the posterior areas, as well as more α1 activity in the left hemisphere over the frontal region during waking-rest, hypnosis-rest1 and hypnosis-rest2. Across emotional conditions, highly hypnotizable subjects had a greater α2 amplitude than the low-hypnotizability group, as well as greater α2 activity over the left-frontal compared to the right-frontal site of the scalp [17]. θ power in patterns of EEG activity significantly increased for the baseline and hypnosis groups in the more posterior areas of the cortex, whereas α activity increased across all sites [15]. θ and β1 bands were located more posteriorly in the high- than low-hypnotizabilty group, and the source gravity of the θ frequency band was to the left of the centers of both β bands, and for the low-hypnotizability group, to the right [24]. During hypnosis, highly hypnotizable subjects had significantly greater activity α than the low-hypnotizability group, and their θ activity was greater during hypnosis than the pre-hypnosis condition [44].

In a recent study from Santacangelo et al. [52], it was explained how the molecular effect had an effect in hypnotized participants. Oxytocin can contribute to suggestion-induced analgesia in highly hypnotizable subjects through activation of the endogenous opioid system [52]. In highly susceptible subjects, the oxytocin receptor gene occurs more frequently, so during a hypnosis, the hypnotizability score is higher and oxytocin release will be lower [52].

Thulium-YAG event-related fMRI showed less activity in regions (brainstem, left and right insula, right primary somatosensory cortex) under hypnosis than normal wakefulness and an increased functional connectivity between S1 and distant insular and prefrontal cortices [35]. Highly susceptible subjects displayed higher ACC activation under hypnosis [28]. A functional magnetic resonance imaging (fMRI) study found that during hypnosis, there was reduced activity in the dorsal anterior cingulate cortex (dACC), increased functional connectivity between the dorsolateral prefrontal cortex (DLPFC; ECN (executive control network)) and the insula in the SN (salience network) and reduced connectivity between the ECN (DLPFC) and the DMN (default mode network and posterior cingulate cortex (PCC) [49]. The largest reduction in pain rating was found in highly hypnotizable subjects during focused analgesia using hypnosis. Somatosensory event-related potentials (SERPs) showed that the N2 amplitude in highly hypnotizable subjects compared to the low-hypnotizability group was greater over frontal and temporal scalp sites than parietal and central sites [22]. High hypnotizable subjects experienced greater pain relief than the low-hypnotizability group in response to hypnosis, and highly hypnotizable subjects also showed significant reductions in somatosensory event-related phase-ordered patterns for focused analgesia during hypnosis [25].

When comparing these results with a recent study from De Souza et al. [53], a justification can be provided as to why the ACC plays a role in hypnosis. GABA concentration in the ACC was positively associated with the hypnotic induction profile in that a higher GABA concentration is associated with greater hypnotizability in individuals [53]. With increasing hypnotizability, a higher GABA concentration could be observed, spanning the same dACC (dorsal anterior cingulate cortex) regions with decreased activity during hypnosis in highly hypnotizable subjects [53]. However, a recent narrative review clarifies that the role of the DLPFC appears to depend on hypnosis and on the type of suggestion given; this is the reason why both activated and reduced activity of the dACC has already been determined [51]. During hypnosis, connectivity between the DLPFC and dACC activation is increased [51].

Positron emission tomography (PET) analysis showed that a hypnotic state induced a significant activation of a right-sided extrastriate area and the anterior cingulate cortex. This activation is related to pain perception and unpleasantness during hypnotic states [20]. Significant increases in PET measures of rCBF (regional cerebral blood flow) during hypnosis were found (left-sided and involved extrastriate visual cortex, inferior parietal lobule, precentral and adjacent promotor cortex and the depth of ventrolateral prefrontal cortex, close to the insular cortex), as well as decreases in rCBF during hypnosis as compared to normal alertness in the left temporal cortex, right temporal cortex, medial prefrontal cortex, posterior cingulate and adjacent precuneus, right promotor cortex and right cerebellar hemisphere [18]. Hypnosis was accompanied by significant increases in rCBF and δ EEG activity. An increase in rCBF subtraction was found in the caudal part of the right anterior cingulate sulcus, the right anterior superior temporal gyrus and the left insula, as well as a decrease associated with hypnosis in the parietal cortex [19]. During hypnosis, dental-phobic subjects showed significantly reduced activation in the left amygdala and bilaterally in the anterior cingulate cortex (ACC), insula and hippocampus [47].

Our primary aim was to focus on functional brain changes during hypnosis in dental applications. However, to the best of our knowledge, there is a limited number of papers available concerning dental applications [6]. Using dental stimuli in a fMRI trial, main effects for anxiety states were found in the left amygdala and bilaterally in the anterior cingulate cortex (ACC), insula and hippocampus (R < L) [47]. Under the hypnosis condition, dental-phobic subjects showed significantly reduced activation in all of these areas [47].

It is difficult to generalize the results due to the various imaging techniques employed and the different application areas of hypnosis. Due to the heterogeneity of the studies, the numerous imaging methods employed and the lack of comparability, a more uniform description is recommended in the design of future hypnosis studies investigating brain activity by means of imaging methods. Hypnotizability does not play a role in patient-specific individual hypnosis treatment, but it is used as a criterion in many studies and examined with validated questionnaires. In the future investigation not only of highly hypnotizable but also medium- and low-hypnotizability subjects is recommended in order to provide a better evaluation of the effectiveness of hypnosis on the whole population.

## 5. Conclusions

- Despite a broad heterogenicity of included studies, evidence of functional changes in brain activity using hypnosis could be determined.
- EMG startle amplitudes indicate higher activity over the frontal brain area; amplitudes using SERP showed similar results.
- EEG oscillations of θ activity are positively associated with response to hypnosis; EEG results showed greater amplitudes for highly hypnotizable subjects over the left hemisphere.
- Less ACC and insula activity was observed during hypnosis.

## Figures and Tables

**Table 1:** Overview of included studies with information on type, imaging method and quality assessment.

No	Author	Type of Study	Method	Quality Assessment
1	London et al. [11]	OS	EEG	Fair
2	Hart [12]	CCS	EEG	Good
3	Morgan et al. [13]	OS	EEG	Poor
4	Tebecis et al. [14]	CCS	EEG	Fair
5	Graffin et al. [15]	CS	EEG	Good
6	De Pascalis et al. [16]	CSS	EEG	Fair
7	De Pascalis et al. [17]	COS	EEG	Fair
8	Maquet et al. [18]	CCS	PET	Fair
9	Rainville et al. [19]	OS	EEG, PET	Fair
10	Faymonville et al. [20]	CSS	PET	Fair
11	Freeman et al. [21]	CSS	EEG	Good
12	De Pascalis et al. [22]	CCS	rCBF + EEG	Good
13	Friedrich et al. [23]	CSS	Thulium YAG Laser + EEG	Fair
14	Isotani et al. [24]	CSS	EEG	Fair
15	De Pascalis et al. [25]	OS	EEG	Good
16	Harandi et al. [26]	RCT	RIA	Poor
17	Wager et al. [27]	CCS	fMRI	Poor
18	Egner et al. [28]	OS	EEG, fMRI	Fair
19	Batty et al. [29]	RCT	EEG	Fair
20	Eitner et al. [30]	CICS	EEG	Good
21	Saadat et al. [31]	RCT	STAI	Good
22	Milling et al. [32]	RCT	CURSS	Fair
23	De Pascalis et al. [33]	CCS	EEG	Fair
24	Marc et al. [34]	RCT	SP	Good
25	Vanhaudenhuyse et al. [35]	CSS	fMRI	Good
26	Krummenacher, [36]	CCS	rTMS	Poor
27	Miltner et al. [37]	CSS	EEG	Poor
28	Brockardt et al. [38]	RCS	rTMS	Good
29	Pyka et al. [39]	CT	fMRI	Poor
30	Trehune et al. [40]	CCS	EEG	Poor
31	Zeidan et al. [41]	RCS	MRI	Good
32	Stein et al. [42]	PCS	MRI	Good
33	Hilbert et al. [43]	CCS	fMRI	Good
34	Williams et al. [44]	OS	EEG	Good
35	Dufresne et al. [45]	RCT	OAH, SHSS:A	Fair
36	Jensen et al. [46]	RCT	EEG	Fair
37	Halsband et al. [47]	CCS	fMRI	Good
38	De Pascalis et al. [48]	CCS	EEG, EMG	Good
39	Jiang et al. [49]	OS	fMRI	Good
40	Williams et al. [50]	RCT	EEG	Good

CICS: comparative interdisciplinary clinical study; CCS: case-control study; CS: comparative study; CSS: cross-sectional Study; CT: clinical trial; CURSS: Carleton University Responsiveness to Suggestion Scale; EEG: electroencephalography; EMG: electromyography; fMRI: functional magnetic resonance imaging; MRI: magnetic resonance imaging; PET: positron-emission tomography; OAH: McConkey’s Opinions About Hypnosis scale; OS: observational study; rCBF: regional cerebral blood flow; RCS: retrospective cohort study; RCT: randomized clinical trial; RIA: rapid-induction analgesia, SHSS:A: Stanford Hypnotic Susceptibility Scales, Form A; SP: surgical pain; STAI: State-Trait Anxiety Inventory; rTMS: repetitive transcranial magnetic stimulation.

**Table 2:** Description of data concerning neutral hypnosis and suggestions for analgesia.

No	Author	Neutral Hypnosis	Suggestion for Analgesia
1	London et al. [11]	x	
2	Hart [12]	x	
3	Morgan et al. [13]	x	
4	Tebecis et al. [14]	x	
5	Graffin et al. [15]	x	
6	De Pascalis et al. [16]		x
7	De Pascalis et al. [17]		x
8	Maquet et al. [18]	x	
9	Rainville et al. [19]	x	x
10	Faymonville et al. [20]		x
11	Freeman et al. [21]		x
12	De Pascalis et al. [22]		x
13	Friedrich et al. [23]		x
14	Isotani et al. [24]	x	
15	De Pascalis et al. [25]		x
16	Harandi et al. [26]		x
17	Wager et al. [27]	x	
18	Egner et al. [28]	x	
19	Batty et al. [29]	x	
20	Eitner et al. [30]		x
21	Saadat et al. [31]	x	
22	Milling et al. [32]	x	
23	De Pascalis et al. [33]		x
24	Marc et al. [34]		x
25	Vanhaudenhuyse et al. [35]		x
26	Krummenacher, [36]		x
27	Miltner et al. [37]	x	
28	Brockardt et al. [38]		x
29	Pyka et al. [39]	x	
30	Terhune et al. [40]	x	
31	Zeidan et al. [41]	x	
32	Stein et al. [42]		x
33	Hilbert et al. [43]	x	
34	Williams et al. [44]	x	
35	Dufresne et al. [45]	x	
36	Jensen et al. [46]		x
37	Halsband & Wolf [47]	x	
38	De Pascalis et al. [48]		x
39	Jiang et al. [49]	x	
40	Williams et al. [50]	x	

**Table 3:** Description of data obtained in hypnotized and non-hypnotized participants.

No	Author	Description of Data Obtained in Hypnotized Participants	Description of Data Obtained in Non-Hypnotized Participants
1	London et al. [11]	High α duration	Lower α duration → problem in high susceptibility: they produce high α under waking condition; no change observed under hypnosis
2	Hart [12]	Rhythm and high susceptibility are positively related	
3	Morgan et al. [13]	More α activity in highly hypnotizable subjects	More α activity
4	Tebecis et al. [14]	- No difference in mean power of the whole EEG spectrum - Trend toward increased θ	No differences in mean power of the whole EEG spectrum
5	Graffin et al. [15]	- Initial baseline period: high susceptibibility, greater θ power in the more frontal areas of the cortex - Period preceding and following standardized hypnotic induction: low susceptibility increased θ activity; high susceptibility decreased - actual hypnotic induction: θ power increased for both in the more posterior areas of the cortex, and α activity increased across all sites	
6	De Pascalis et al. [16]	High susceptibility: - Significant reduction in pain and distress - EEG activity recorded from central and posterior sites showed total and δ EEG amplitude reductions, as well as θ1 reduction on the left side - Decrease in the level of sympathetic activity Low susceptibility: - EEG activity recorded from posterior displayed δ amplitude reduction - EEG activity recorded from frontal, central, posterior displayed reduction in θ1	High susceptibility: less reduction in pain and distress
7	De Pascalis et al. [17]	High susceptibility: - Higher level of visual imagery than low susceptibility - Higher level of emotionality than low susceptibility - Greater θ1 amplitude over left frontal compared to right hemisphere and posterior areas Low susceptibility: - Greater θ1 amplitude over left frontal sites compared to right	- Greater θ1 amplitude in the right hemisphere compared to the left in posterior recording sites - High susceptibility produced more θ2 - α1 activity high in waking rest and hypnotized for high susceptibility in the left hemisphere over frontal region
8	Maquet et al. [18]	Activation of widespread, mainly left-sided set of cortical areas involving occipital, parietal, precentral, premotor, ventrolateral and prefrontal cortices, as well as a few right-sided regions (occipital, anterior, cingulate cortices)	Activates the anterior part of both temporal lobes, basal forebrain structures and some left mesiotemporal areas (not hypnotized but listening to autobiographical material)
9	Rainville et al. [19]	- Significant increases in both occipital rCBF and δ EEG activity - Peak increases in rCBF were observed in the caudal part of the right anterior cingulate sulcus and bilaterally in the inferior frontal gyri - Hypnosis-related decreases in rCBF were found in the right inferior parietal lobule, the left precuneus and the posterior cingulate gyrus - Medial and lateral posterior parietal cortices showed suggestion-related increases overlapping partly with regions of hypnosis-related decrease	- Consistent ACC activation in response to experimental painful stimuli Pain-related effect was independent
10	Faymonville et al. [20]	- Decrease in both pain sensation and the unpleasantness of noxious stimuli - Noxious stimulation caused an increase in regional cerebral blood flow in the thalamic nuclei, as well as anterior cingulate and insular cortices - Significant activation of a right-sided extrastriate area and the anterior cingulate cortex	- Activity in the anterior (mid-)cingulate cortex was differently related to pain perception and unpleasantness in the hypnotic state compared to control situations
11	Freeman et al. [21]	High hypnotizability: - Significantly greater pain relief for hypnosis vs. distraction or waking relaxation conditions Significantly greater pain relief than low hypnotizability - Significantly greater high θ activity as compared to low hypnotizability at parietal and occipital sites	Significantly greater high θ activity for high hypnotizability as compared to low hypnotizability at parietal and occipital sites
12	De Pascalis et al. [22]	High susceptibility: - Focused analgesia induced the greatest reduction in pain rating - N2 amplitude was greater over frontal and temporal scalp sites than over parietal and central sites - Low susceptibility: - N2 was greater over temporal sites than over frontal, parietal and central sites - Larger N2 peak over temporal sites during focused analgesia	P3 peaks were smaller during focused analgesia, deep relaxation and dissociated imagery conditions compared to placebo
13	Friedrich et al. [23]	- Pain reports were significantly reduced - Amplitudes of the late laser-evoked brain potential (LEP) components N200 and P320 were significantly smaller for distraction of attention than the control condition	N200 and P320 were higher
14	Isotani et al. [24]	High susceptibility: - full-band global dimensional complexity was higher than in low susceptibility	Before hypnosis, high and low hypnotizability were in different brain electric states, with more posterior brain activity gravity centers (excitatory right, routine or relaxation left) and higher dimensional complexity (higher arousal) in high than the low-hypnotizability group
15	De Pascalis et al. [25]	- Ohase-ordered γ scores over frontal scalp site predicted pain ratings High hypnotizability: - Significant pain and distress reductions for focused analgesia during hypnosis and, to a greater extent, during post-hypnosis condition compared to low and medium hypnotizability - Significant reductions in phase-ordered γ patterns for focused analgesia during hypnosis and post-hypnosis conditions	Phase-ordered γ scores over central scalp site predicted subjects’ pain ratingsPhase-ordered γ scores over frontal scalp site predicted pain ratings for high, medium and low hypnotizability
16	Harandi et al. [26]	Degree of pain and anxiety caused by physiotherapy decreased significantly	
17	Wager et al. [27]		Placebo analgesia was related to decreased brain activity in pain-sensitive brain regions, including the thalamus, insula and anterior cingulate cortex Placebo analgesia was associated with increased activity during anticipation of pain in the prefrontal cortex
18	Egner et al. [28]	High susceptibility: - Participants displayed increased conflict-related neural activity compared to baseline - Decrease in functional connectivity (EEG γ band coherence) between frontal midline and left lateral scalp sites	Cognitive-control-related LFC activity did not differ
19	Batty et al. [29]	- Further evidence that operant control over the theta/alpha ratio is possible - Elevation of the theta/alpha ratio proved no more successful than the other interventions	
20	Eitner et al. [30]	- (α-) θ-activity during hypnosis with a peak in the posterior section of the brainalong with lateral shifting Brain activity changed under hypnosis from the left to the right hemisphere, and with further intensification of the trance state, it changed from the anterior to the posterior brain segments	β waves indicating an awakened state
21	Saadat et al. [31]	- Significantly less anxious post-intervention as compared with patients in the attention-control group and the control group - Significant decrease of 56% in anxiety level	Increase of 47% in anxiety
22	Milling et al. [32]	The extent of mediation increased as participants gained more experience with the interventions	
23	De Pascalis et al. [33]	High susceptibility: - Experienced significant pain and distress reductions during post-hypnotic analgesia as compared to hypnotic analgesia - Smaller number of target stimuli and displayed a significant amplitude reduction in the midline frontal and central N140 and P200 SERP components	Less pain and lower distress levels No significant SERP differences
24	Marc et al. [34]	- Mental imagery of a secure place was the strategy used by most women (71%) in the hypnosis group. A significant propotion of them used focal analgesia (39%)	
25	Vanhaudenhuyse et al. [35]	- Intensity-matched stimuli in both the non-painful and painful range failed to elicit any cerebral activation - Increases in functional connectivity between S1 and distant anterior insular and prefrontal cortices	Stimuli in the non-painful range activated brainstem, contralateral primary somatosensory (S1) and bilateral insular cortices Painful stimuli activated additional areas, encompassing thalamus, bilateral striatum, anterior cingulate (ACC), premotor and dorsolateral prefrontal cortices Contralateral thalamus, bilateral striatum and ACC activated more than in hypnosis
26	Krummenacher, [36]	Significant increase in pain threshold and tolerance	The sensation of pain was not affected
27	Miltner et al. [37]	- Significantly less painful sensations - Smaller magnitudes of more topographically focused brain oscillations within the γ band above the primary sensory representation areas of the stimulated hand/finger in response to the noxious stimuli - Slower oscillations were significantly reduced at more extended brain areas spanning the primary and secondary sensory and more frontal executive brain areas	Slow oscillations within focused and extended brain areas broke down completely during hypnotic oscillations, as compared to the distraction condition
28	Brockardt et al. [38]	- Left dorsolateral prefrontal TMS may produce analgesic effects by acting through a cortical perceived-control circuit regulating limbic and brainstem areas of the pain circuit - Perceived control on the emotional dimension of pain but not the sensory/discriminatory dimension	
29	Pyka et al. [39]	- Increased connectivity of the precuneus with the right dorsolateral prefrontal cortex, angular gyrus and a dorsal part of the precuneus - Functional connectivity of the medial frontal cortex and the primary motor cortex remained unchanged	Functional connectivity of the medial frontal cortex and the primary motor cortex remained unchanged compared to hypnotized participants
30	Terhune et al. [40]	High suggestibility: - Experienced greater state dissociation and exhibited lower frontal-parietal phase synchrony in the α2 frequency band than low suggestibility	
31	Zeidan et al. [41]	- Significantly reduced pain unpleasantness by 57% and pain intensity ratings by 40% - Reduced pain-related activation of the contralateral primary somatosensory cortex - Reductions in pain intensity ratings were associated with increased activity in the anterior cingulate cortex and anterior insula, areas involved in the cognitive regulation of nociceptive processing - Reductions in pain unpleasantness ratings were associated with orbitofrontal cortex activation, an area implicated in reframing the contextual evaluation of sensory events	
32	Stein et al. [42]	- Positively correlated with fractional anisotropy in the right dorsolateral prefrontal cortex, left rostral anterior cingulate cortex and the periaqueductal gray region - Stronger placebo analgesic responses = increased mean fractional anisotropy values within, white matter tracts connecting the periaqueductal gray with pain-control regions, such as the rostral anterior cingulate cortex and the dorsolateral prefrontal cortex	
33	Hilbert et al. [43]	Increased activation in the insula, anterior cingulate cortex, orbitofrontal cortex, and thalamus in dental-phobic subjects compared to healthy controls during auditory stimulation	Activation in orbitofrontal and prefrontal gyri in dental-phobic subjects related to processes of cognitive control
34	Williams et al. [44]	High susceptibility: - θ had greater activity post-hypnosis → θ is an index of relaxation that continues after hypnosis - α posterior power increased from the pre-hypnosis to hypnosis conditions and decreased post-hypnosis - Greater α power than in low susceptibility during both pre-hypnosis and hypnosis Low susceptibility: - α posterior power decreased from the pre-hypnosis to hypnosis conditions and increased post-hypnosis	High susceptibility: α posterior power decreased Greater α power than low susceptibility Low susceptibility: Higher α posterior power compared to hypnosis
35	Dufresne et al. [45]	No significant difference	
36	Jensen et al. [46]	- More presession θ power was associated with greater response to hypnotic analgesia - Less baseline α power predicted pain reduction with meditation	
37	Halsband & Wolf [47]	- Dental-phobic subjects, main effects of fear condition: Left amygdala and bilaterally in the anterior cingulate cortex (ACC), insula and hippocampus (R < L) → significant reduction in all areas during hypnosis - No amygdala activation	Reduced neural activity patterns No amygdala activation Less bilateral activation in the insula and ACC compared to dental-phobic subjects
38	De Pascalis et al. [48]	- Highly hypnotizable participant placebo treatment produced significant reductions in pain and distress perception - Placebo analgesia involved activity of the left hemisphere, including the occipital region - Pain reduction was associated with larger EMG startle amplitudes and N100 and P200 responses, as well as enhanced activity within the frontal, parietal, anterior and posterior cingulate gyres	Highly hypnotizable participants: placebo treatment produced significant reductions in pain and distress perception During placebo analgesia, P200 wave was larger in the frontal left hemisphere
39	Jiang et al. [49]	Reduced activity in the dACC, increased functional connectivity between the dorsolateral prefrontal cortex (DLPFC;ECN(executive control network)) and the insula in the SN (salience network), and reduced connectivity between the ECN (DLPFC) and the DMN (PCC(posterior cingulate cortex))	
40	Williams et al. [50]	Protocol only	Protocol only

## Data Availability

No new data were created or analyzed in this study. Data sharing is not applicable to this article.

## Supplementary Materials

The following are available online at https://www.mdpi.com/article/10.3390/brainsci12010108/s1, Table S1: List of excluded papers, Table S2: Quality assessment of included papers, Table S3: PRISMA checklist.
