# Supplementary material for: Functional Changes in Brain Activity Using Hypnosis: A Systematic Review
Source: Brain Sci. 2022 Jan 13;12(1):108. doi: 10.3390/brainsci12010108 (PMC8773773; doi:10.3390/brainsci12010108)
Supplement: Supplementary file 1 [file brainsci-12-00108-s001.zip › 3_Table S1.suppl.Material_Listofexcludedpapers.pdf]

List of excluded papers

ID	Author	Year	Sources	reason for exclusion
1	Zuo et al.,	2021	Medicine (Baltimore). 2021 Mar 5; 100(9):e24535	
2	Zhou et al.,	2021	Soc Cogn Affect Neurosci. 2021 May 21; 16(6):621-631	non pertinent
3	Zhengpeng et al.	2021	Chinese journal of tissue engineering research, 2021; 25(12):1826-1830	non pertinent
4	Zheng et al.,	2021	Pain Med. 2021 Mar 18; 22(3):606-615	non pertinent
5	Zhang et al.,	2021	Ann Palliat Med. 2021 May; 10(5):5908-5918	non pertinent
6	Zhang et al.,	2021	Reg Anesth Pain Med. 2021 Feb; 46(2):145-150	non pertinent
7	Zandonai et al.,	2021	J Sports Sci. 2021 Jul; 39(13):1452-1460	non pertinent
8	Zabik et al.,	2021	Biological psychiatry, 2021; 89(9):104-	non pertinent
9	Younis et al.,	2021	J Cereb Blood Flow Metab. 2021 Mar; 41(3):604-616	non pertinent
10	Yang et al.,	2021	Cells, 2021; 10(7)	non pertinent
11	Wu et al.,	2021	Pain management nursing, 2021; https://doi.org/10.1016/j.pmn.2021.01.004	non pertinent
12	Woodbury et al.,	2021	Pain Med. 2021 Mar 18; 22(3):715-726	non pertinent
13	Williamson et al.,	2021	Health technology assessment, 2021; 25(16):VIII-33	non pertinent
14	Wilby et al.,	2021	Health technology assessment (Winchester, England), 2021; 25(24):1-86	non pertinent
15	Wijenberg et al.,	2021	J Clin Exp Neuropsychol. 2021 Feb; 43(1):66-77	non pertinent
16	Westphal et al.,	2021	Translational psychiatry, 2021; 11(1):389	non pertinent
17	Werdyani et al.,	2021	Osteoarthritis and cartilage, 2021; 29:17	non pertinent

18	Wei et al.,	2021	BMJ Open. 2021 Aug 16; 11(8):e048093	non pertinent
19	Wang et al.,	2021	J Orthop Res. 2021 Jul; 39(7):1470-1478	non pertinent
20	Wang et al.,	2021	Frontiers in neuroscience, 2021; 15	non pertinent
21	Wagatsuma et al.,	2021	Pain management nursing, 2021	non pertinent
22	Wang et al.,	2021	Zhongahu yi xue za zhi, 2021; 101(21):1592-1597	non pertinent
23	Vogt et al.,	2021	Anesthesiology. 2021 Jul 1; 135(1):69-82	non pertinent
24	Venkatesh et al.,	2021	Am J Emerg Med. 2021 Jan; 39:102-108	non pertinent
25	Vencatachellum et al.,	2021	European journal of pain (united kingdom), 2021	non pertinent
26	Vaninetti et al.,	2021	Scientific reports, 2021; 11(1):9573-	non pertinent
27	Upadhyay et al.,	2021	Journal of perinatology, 2021	non pertinent
28	Toyooka et al.,	2021	BMC Musculoskelet Disord. 2021 Mar 18; 22(1):285	non pertinent
29	Tong et al.,	2021	Clin Respir J. 2021 Sep; 15(9):1012-1018	non pertinent
30	Thakkar et al.,	2021	Eur Arch Paediatr Dent. 2021 Jun; 22(3):459-467	non pertinent
31	Thakar et al.,	2021	Indian journal of medical research, supplement, 2021; 153(1-2):151-158	non pertinent
32	Tchaconas et al.,	2021	Pediatrics, 2021; 147(3):636-637	non pertinent
33	Tang et al.,	2021	PLoS One. 2021 Mar 26; 16(3):e0247678	non pertinent
34	Takami et al.,	2021	Spine J. 2021 Mar; 21(1):446-454	non pertinent
35	Takahashi et al.,	2021	BMC Musculoskelet Disord. 2021 Feb 11; 22(1):167	non pertinent
36	Taguchi et al.,	2021	Medicine (Baltimore). 2021 Feb 12; 100(6):e23859	non pertinent
37	Sweeney et al.,	2021	Pilot and feasibility studies, 2021; 7(1)	non pertinent

38	Susano et al.,	2021	Anesth Analg. 2021 Mar 1; 132(3):846-855	non pertinent
39	Sun et al.,	2021	Trials. 2021 Jun 14; 22(1):399	non pertinent
40	Stuepp et al.,	2021	J Oral Maxillofac Surg. 2021 May79(5):1025.e1-1025.e8	non pertinent
41	Strauss et al.,	2021	Journal of pain, 2021	non pertinent
42	Steuber	2021	J Psychiatr Res. 2021 Sep; 141:325-332	non pertinent
43	Srikesavan et al.,	2021	BMJ Open. 2021 Jun 11; 11(6):e040829	non pertinent
44	Solaymani-Dodaran et al.,	2021	International immunopharmacology, 2021; 95:107522	non pertinent
45	Siper et al.,	2021	Genes (Basel). 2021 Feb 27; 12(3):351	non pertinent
46	Simis et al.,	2021	Pain medicine (Malden, Mass.), 2021	non pertinent
47	Siddiqi et al.,	2021	Spine deformity, 2021; 9(1):13-40	non pertinent
48	Sheth et al.,	2021	Clin Rheumatol. 2021 Feb; 40(2):735-740	non pertinent
49	Seo et al.,	2021	Biol Psychiatry Cogn Neurosci Neuroimaging. 2021 Mar; 6(3):299-309	non pertinent
50	Serper et al.,	2021	Liver Int. 2021 Apr; 41(4):692-704	non pertinent
51	Schweda et al.,	2021	Osteoarthritis and cartilage, 2021; 29:384-385	non pertinent
52	Schuppan et al.,	2021	New England journal of medicine, 2021; 385(1):35-45	non pertinent
53	Schnider et al.,	2021	Clin Pharmacol Ther. 2021 Aug; 110(2):401-408	non pertinent
54	Schneider et al.,	2021	Journal of pain, 2021; 22(5):605-606	non pertinent
55	Schmidt et al.,	2021	BMJ Open. 2021 Jun 23; 11(6):e045969	non pertinent
56	Schensul et al.,	2021	BMC Oral Health. 2021 Jul 21; 21(1):362	non pertinent
57	Schega et al.,	2021	BMC Geriatr. 2021 Mar 2; 21(1):151	non pertinent

58	Sahgal et al.,	2021	Lancet Oncol. 2021 Jul; 22(7):1023-1033	non pertinent
59	Saeedi et al.,	2021	International journal of pharmaceutical research, 2021; 13(3):671-684	non pertinent
60	Saadi et al.	2021	PloS one, 2021; 16(3)	non pertinent
61	Russ et al.,	2021	J Back Musculoskelet Rehabil. 2021; 34(1):139-147	non pertinent
62	Rubino et al.,	2021	Obesity facts, 2021; 14(1):118-119	non pertinent
63	Rosenberg et al.,	2021	Spine (Phila Pa 1976). 2021 Mar 15; 46(6):347-355	non pertinent
64	Rocha et al.,	2021	Neuroscience letters, 2021; 742:135539	non pertinent
65	Riva et al.,	2021	Anaesthesia. 2021 Jan; 76(1):27-35	non pertinent
66	Riegner et al.,	2021	Journal of pain, 2021; 22(5):605-	non pertinent
67	Rengasamy et al.,	2021	Cognitive therapy and research, 2021; 45:795-804	non pertinent
68	Ren et al.,	2021	Journal of healthcare engineering, 2021; 2021	non pertinent
69	Rausch et al.,	2021	Military medical research	non pertinent
70	Rasoul et al.,	2021	Clinical medicine, journal of the royal college of physicians of london, 2021; 21(2):90-95	non pertinent
71	Rao et al.,	2021	Journal of clinical oncology, 2021; 39(6)	non pertinent
72	Rajasekaran et al.,	2021	Eur Spine J. 2021 Jul; 30(7):2069-2081	non pertinent
73	Raja et al.,	2021	Trials. 2021 Aug 28; 22(1):574	non pertinent
74	Pratscher et al.,	2021	Nutrients. 2021 May 5; 13(5):1556	non pertinent
75	Perkins et al.,	2021	Translational psychiatry, 2021; 11(1)	non pertinent
76	Pérez Duenas et al.,	2021	Arch Bronconeumol. 2021 Jan; 57 Suppl 1.:35-46	non pertinent
77	Peng et al.,	2021	Journal of shoulder and elbow surgery, 2021	non pertinent

78	Pei et al.,	2021	Medicine, 2021; 100(19):e25948	non pertinent
79	Paulus et al.,	2021	Neuropsychopharmacology. 2021 Apr; 46(5):1011-1019	non pertinent
80	Patel et al.,	2021	PLoS One. 2021 Feb 1; 16(2):e0245827	non pertinent
81	Patel et al.,	2021	JAMA network open, 2021	non pertinent
82	Pang et al.,	2021	World journal of clinical cases, 2021; 8(14):2942-2949	non pertinent
83	Pappolla et al.,	2021	Pain Physician. 2021 Mar; 24(2):175-184	non pertinent
84	Pacini et al.,	2021	Frontiers in pharmacology, 2020; 12	non pertinent
85	Öztürk et al.,	2021	J Clin Neurosci. 2021 Aug; 90:144-151	non pertinent
86	Oliver-Fornies et al.,	2021	Trials, 2021; 22(1)	non pertinent
87	Oikonomou et al.,	2021	European heart journal, 2021	non pertinent
88	Obermann et al.,	2021	The lancet. Neurology, 2021; 20(1):29-37	non pertinent
89	Nianjun et al.,	2021	Chinese journal of tissue engineering research, 2021; 25(6):866-872	non pertinent
90	Neogi et al.	2021	BJOG, 2021; 128(2):29-30	non pertinent
91	Naji et al.,	2021	Int J Oral Maxillofac Surg. 2021 May; 50(5):683-690	non pertinent
92	Muthulingam et al.,	2021	Neuromodulation, 2021	non pertinent
93	Mowafy et al.,	2021	J Anesth. 2021 Aug; 35(4):515-524	non pertinent
94	Mouraux et al.,	2021	Trials. 2021 Jun 17; 22(1):404	non pertinent
95	Moerkerke et al.,	2021	European neuropsychopharmacology, 2021; 44:51-52	non pertinent
96	Ming et al.,	2021	Medicine (Baltimore). 2021 Jan 8; 100(1):e23998	non pertinent
97	Mead et al.,	2021	British journal of haematology, 2021; 193(1):96-	non pertinent
98	McPhee et al.,	2021	The journal of pain, 2021	non pertinent
99	McLarty et al.,	2021	Vet Radiol Ultrasound. 2021 Jul; 62(4):498-506	non pertinent

100	McLean et al.,	2021	Otol Neurotol. 2021 Aug 1; 42(7):e849-e857	non pertinent
101	McHugo et al.,	2021	J Nerv Ment Dis. 2021 Feb 1; 209(2):92-99	non pertinent
102	Mawla et al.,	2021	Arthritis Rheumatol. 2021 Jul; 73(7):1318-1328	non pertinent
103	Maheu et al.,	2021	Osteoarthritis and cartilage, 2021; 29:420-421	non pertinent
104	Ma et al.,	2021	Brain Behav. 2021 Apr; 11(4):e02059	non pertinent
105	Lychagin et al.,	2021	Open access macedonian journal of medical sciences, 2021; 9(B):398-402	non pertinent
106	Luo et al.,	2021	Journal of orthopaedics and traumatology, 2021; 22(1)	non pertinent
107	Lunger et al.,	2021	BMJ open, 2021; 11(1)	non pertinent
108	Lopes et al.,	2021	European archives of oto-rhino-laryngology, 2021	non pertinent
109	Liu et al.,	2021	Indian journal of pharmaceutical sciences, 2021; 83:148-153	non pertinent
110	Listik et al.,	2021	Eur J Pain. 2021 Jul; 25(6):1355-1366	non pertinent
111	Lelic et al.,	2021	The journal of pain, 2021	not pertinent
112	Legakis et al.,	2021	Ann Palliat Med. 2021 Jun; 10(6):6336-6343	non pertinent
113	Lee et al.,	2021	Eur J Pediatr. 2021 May; 180(5):1413-1422	not pertinent
114	Lazaro-Navas et al.,	2021	Int J Environ Res Public Health. 2021 Jun 3; 18(11):6018	not pertinent
115	Lasocki et al.,	2021	BMJ open, 2021; 11(1)	not pertinent
116	Lam et al.,	2021	Medicine (Baltimore). 2021 Feb 26; 100(8):e23718	not pertinent
117	Laird et al.,	2021	Complement Ther Clin Pract. 2021 May; 43:101374	not pertinent
118	Kumar et al.,	2021	Neurol Sci. 2021 Jan; 42(1):131-139	not pertinent

119	Korgaonkar et al.,	2021	Translational psychiatry, 2021; 11(1)	not pertinent
120	Koo et al.,	2021	J Headache Pain. 2021 Apr 20; 22(1):28	not pertinent
121	Komulainen et al.,	2021	NeuroImage, 2021; 237	not pertinent
122	Kold et al.,	2021	Pain. 2021 Jun 1; 162(6):1659-1668	not pertinent
123	Kohnen et al.,	2021	J Adv Nurs. 2021 Jul;77(7):3058-3072	not pertinent
124	Klimas et al.,	2021	Contemporary clinical trials, 2021; 107	not pertinent
125	Kisby et al.,	2021	Alcoholism: clinical and experimental research, 2021; 45(1):103A-	not pertinent
126	Kim et al.,	2021	European urology focus, 2021	not pertinent
127	Kim et al.,	2021	Journal of clinical medicine, 2021; 10(15)	not pertinent
128	Kim et al.,	2021	Journal of personalized medicine, 2021; 11(5)	not pertinent
129	Khong et al.,	2021	Eur J Sport Sci. 2021 Feb; 21(2):224-230	not pertinent
130	Khera et al.,	2021	BMJ open, 2021; 11(3)	not pertinent
1301	Khanna et al.,	2021	J Assoc Physicians India. 2021 Apr; 69(4):11-12	not pertinent
132	Kerautret et al.,	2021	Brain science, 2021; 11(6)	not pertinent
133	Kang et al.,	2021	Spine journal, 2021	not pertinent
134	Kanen et al.,	2021	Biol Psychiatry Cogn Neurosci Neuroimaging. 2021 May; 6(5):590-598	not pertinent
135	Kadali et al.,	2021	International journal of infectious diseases, 2021; 106:376-381	not pertinent
136	Juraskova et al.,	2021	BMJ Open. 2021 May 28; 11(5):e043224	not pertinent
137	Jones et al.,	2021	J Med Internet Res. 2021 Apr 30; 23(4):e25872	not pertinent
138	Jones et al.,	2021	Eur J Pain. 2021 Jan; 25(1):149-159	not pertinent
139	Jin et al.,	2021	Orthopaedic surgery, 2021	not pertinent

140	Jianjian et al.,	2021	Medicine, 2021; 100(9):e24535-	not pertinent
141	Jeon et al.,	2021	Neuro-oncology, practice, 2021; 8(1):48-59	not pertinent
142	Jande Beur et al.,	2021	J Bone Miner Res. 2021 Apr; 36(4):627-635	not pertinent
143	Inose et al.,	2021	Qual Life Res. 2021 Jan; 30(1):129-135	not pertinent
144	Imseeh et al.,	2021	Int J Hyperthermia. 2021; 38(1):623-632	not pertinent
145	Imanishi et al.,	2021	J Bone Miner Res. 2021 Feb; 36(2):262-270	not pertinent
146	Hyun et al.,	2021	Journal of Korean Neurosurgical Society, 2021; 64(4):562-574	not pertinent
147	Hurlemann et al.,	2021	Biological psychiatry, 2021; 89(9):33-	not pertinent
148	Hofman et al.,	2021	Lancet, 2021; 397(10276):797-804	not pertinent
149	Hofman et al.,	2021	Social neuroscience, 2021; 16(4):362-374	not pertinent
150	Hernandez-Franco et al.,	2021	Toxicon, 2021; 190:33-	not pertinent
151	Hedberg et al.,	2021	BMC Oral Health. 2021 Jun 9; 21(1):297	not pertinent
152	Hebel et al.	2021	Brain stimulation, 2021; 14(2):335-343	not pertinent
153	Heapy et al.,	2021	J Behav Med. 2021 Apr; 44(2):260-269	not pertinent
154	Hasvik et al.,	2021	Clin Orthop Relat Res. 2021 Apr 1; 479(4):651-663	not pertinent
155	Harvey et al.,	2021	BMC Pediatr. 2021 Aug 28; 21(1):368	not pertinent
156	Harvie et al.,	2021	Animal cognition 2021; 24(2):239-250	not pertinent
157	Hamadani et al.,	2021	The lancet haematology, 2021; 8(6):e433-e445	not pertinent
158	Harba et al.,	2021	Dental and medical problems, 2021; 58(1):81-88	not pertinent
159	Griffith et al.,	2021	Emerg Radiol. 2021 Jun; 28(3):485-495	not pertinent

160	Gruber et al.,	2021	Exp Clin Psychopharmacol. 2021 Apr; 29(2):147-156	not pertinent
161	Gregoret et al.,	2021	Eur J Pain. 2021 Jul; 25(6):1241-1253	not pertinent
162	Grahl et al.,	2021	Journal of pain, 2021; 22(5):602-	not pertinent
163	Gikic et al.,	2021	J Oral Rehabil. 2021 Jul; 48(7):785-797	not pertinent
164	Gholami et al.,	2021	Neurophysiologie clinique, 2021; 51(4):319-328	not pertinent
165	Gatti et al.,	2021	Eur J Neurosci. 2021 Jan; 53(2):556-570	not pertinent
166	Garrison et al.,	2021	Cognitive, affective & behavioral neuroscience, 2021; CN-02242328	not pertinent
167	Garrett et al.,	2021	Bipolar disorders, 2021	not pertinent
168	Gardoki-Soutoet al.,	2021	Trials, 2021; 22(1)	not pertinent
169	Garcia et al.,	2021	J Med Internet Res. 2021 Feb 22; 23(2):e26292	not pertinent
170	Fortin et al.,	2021	BMC musculoskeletal disorders, 2021; 22(1)	not pertinent
171	Forogh et al.,	2021	Neurophysiology clinique, 2021	not pertinent
172	Fisher et al.,	2021	Nat Med. 2021 Jan; 27(1):165-173	not pertinent
173	Ferrero et al.,	2021	arthritis and rheumatism, 2021; 51(4):831-838	not pertinent
174	Fenton et al.,	2021	Trials. 2021 Feb 27; 22(1):167	not pertinent
175	Feger et al.,	2021	Eur Radiol. 2021 Mar; 31(3):1471-1481	not pertinent
176	Falahatgar et al.,	2021	Osteoarthritis and cartilage, 2021; 29:399-401	not pertinent
177	Esrick et al.,	2021	N Engl J Med. 2021 Jan 21; 384(3):205-2015	not pertinent
178	Ettridge et al.,	2021	Qual Life Res. 2021 Feb; 30(2):407-423	not pertinent
179	Elbogen et al.,	2021	Pain Med. 2021 Feb 23; 22(2):329-337	not pertinent
180	Düzkaya et al.,	2021	J Emerg Nurs. 2021 Jan; 47(1):76-87	not pertinent

181	Du et al.,	2021	Medicine, 2021; 100(7):e24786	not pertinent
182	Dou et al.,	2021	Psychopharmacology (Berl). 2021 Mar; 238(3):677-689	not pertinent
183	Direito et al.,	2021	Autism, 2021	not pertinent
184	De Souza et al.,	2021	Brain stimulation, 2021	not pertinent
185	Deng et al.,	2021	Clinical breast cancer, 2021	not pertinent
186	de Jong-Schmit et al.,	2021	International journal of geriatric psychiatry, 2021; 36(1):46-53	not pertinent
187	De Icco et al.,	2021	Clin Neurophysiol. 2021 Jan; 132(1):126-136	not pertinent
188	de Groot et al.,	2021	Psychol Sci. 2021 Apr; 32(4):558-572	not pertinent
189	de Gooijer et al.,	2021	Lancet Respir Med. 2021 Jun; 9(6):585-592	not pertinent
190	Das et al.,	2021	Journal of laryngology and otology, 2021; 135(1):39-44	not pertinent
191	Dai et al.,	2021	Arthroscopy - journal of arthroscopic and related surgery, 2021; 37(1):340-358	not pertinent
192	Dabelic et al.,	2021	Libri oncologici, 2021; 49(1):54-56	not pertinent
193	Cruz et al.,	2021	Clin Oral Implants Res. 2021 Jun; 32(6):711-720	not pertinent
194	Croosu et al.,	2021	Journal of neuroimaging, 2021; https://doi.org/10.1111/jon.12902	not pertinent
195	Cowfer et al.,	2021	Pediatric blood & cancer, 2021; 68(3):34-35	not pertinent
196	Conklin et al.,	2021	J Neurol Sci. 2021 Feb 15; 421:117308	not pertinent
197	Cobb et al.,	2021	J Anxiety Disord. 2021 Mar; 78:102344	not pertinent
198	Christensen et al.,	2021	Nordic journal of psychiatry, 2021; 75(1):1-8	not pertinent
199	Chrisman et al.,	2021	Frontiers in pediatrics, 2021; 9	not pertinent

200	Chen et al.,	2021	Experimental and therapeutic medicine, 2021; 22(2)	not pertinent
201	Chen et al.,	2021	Clin Interv Aging. 2021 May 17; 16:843-852	not pertinent
202	Chen et al.,	2021	Journal of clinical oncology, 2021; 39(6)	not pertinent
203	Chhabra et al.,	2021	J Ultrasound Med. 2021 Jun;40(6):1147-1153	not pertinent
204	Cerritelli et al.,	2021	Scientific reports, 2021; 11(1):4556-	not pertinent
205	Cederström et al.,	2021	J Sci Med Sport. 2021 Mar; 24(3):258-263	not pertinent
206	Carswell et al.,	2021	Trials. 2021 Aug 30; 22(1):580	not pertinent
207	Burns et al.,	2021	Pain, 2021	not pertinent
208	Bush et al.,	2021	Journal of pain, 2021; 22(5):603-	not pertinent
209	Bryant et al.,	2021	Translational psychiatry, 2021; 11(1)	not pertinent
210	Britting et al.,	2021	Trials. 2021 Aug 21; 22(1):552	not pertinent
211	Bourdon et al.,	2021	Hum Reprod. 2021 Jan 25; 36(2):349-357	not pertinent
212	Blank et al.,	2021	Diseases of the colon and rectum, 2021; 64(2):225-233	not pertinent
213	Berry-Kravis et al.	2021	Biological psychiatry, 2021; 89(9):226-227	not pertinent
214	Berezovskaya et al.,	2021	Osteoarthritis and cartilage, 2021; 29:272-273	not pertinent
215	Benoit et al.,	2021	Early human development, 2021; 154	not pertinent
216	Benito et al.,	2021	Evidence-based practice in child and adolescent mental health, 2021; 6(2):173-190	not pertinent
217	Bayram et al.,	2021	Am J Emerg Med. 2021 Sep; 47:125-130	not pertinent
218	Bandak et al.,	2021	Knee. 2021 Jan; 28:256-265	not pertinent
219	Balocco et al.,	2021	Reg Anesth Pain Med. 2021 Jan; 46(1):35-40	not pertinent
220	Balconi et al.,	2021	Pain Res Manag. 2021 Apr 3; 2021:5585060	not pertinent

221	Al-Karagholi et al.,	2021	J Cereb Blood Flow Metab. 2021 Jun; 41(6):1328-1337	not pertinent
222	Ahmadi et al.,	2021	Trials. 2021 Jan 25; 22(1):87	not pertinent
223	Addiego et al.,	2021	Life Sci. 2021 Oct 1; 282:119749	non pertinent
224	Ackermann et al.,	2021	J Hand Ther. 2021 Apr-Jun; 34(2):309-314	non pertinent
225	Abonour et al.,	2021	Br J Haematol. 2021 Apr; 193(1):93-100	non pertinent
226	AbdElkader et al.,	2021	Egyptian journal of neurology, psychiatry and neurosurgery, 2021; 57(1)	non pertinent
227	Ziegeler et al.,	2020	Neurology. 2020 Nov 17; 95(20):e2794-e2802	non pertinent
228	Zhu et al.,	2020	Trials. 2020 Sep 16; 21(1):795	non pertinent
229	Zhu et al.,	2020	BMJ open, 2020; 10(10)	non pertinent
230	Zhao et al.,	2020	Psychophysiology; 2020:e13717-	non pertinent
231	Zhang et al.,	2020	Chinese journal of tissue engineering research, 2020; 24(15):2405-2409	non pertinent
232	Zehry et al.,	2020	Neuroreport, 2020; 31(5):406-410	non pertinent
233	Zdziarski-Horodyski et al.,	2020	Clinical orthopaedics and related research, 2020; 478(4):792-804	non pertinent
234	Zale et al.,	2020	Experimental and clinical psychopharmacology, 2020	non pertinent
235	Zahirovic	2020	Libri oncologici, 2020; 48(1):58-59	non pertinent
236	Zahid et al.,	2020	JMIR mhealth and uhealth, 2020; 8(4):e17108-	non pertinent
237	Yung et al.,	2020	J Man Manip Ther. 2020 May; 28(2):82-93	non pertinent
238	Yu et al.,	2020	Journal of clinical medicine, 2020; 9(6):1-16	non pertinent
239	Younossi et al.	2020	Hepatology (Baltimore, Md.), 2020; 72(1):1019A-1020A	non pertinent

240	You et al.,	2020	International journal of clinical and experimental medicine, 2020;	non pertinent
241	Ying et al.,	2020	J Orthop Res. 2020 Feb; 38(2):269-276	non pertinent
242	Yearwood et al.,	2020	Neuromodulation. 2020 Jan; 23(1):56-63	non pertinent
243	Yanzhi et al.,	2020	Brain structure & function, 2020; 226:151-162	non pertinent
244	Yang et al.,	2020	PLoS One. 2020 Nov 5; 15(11):e0241828	non pertinent
245	Yan et al.,	2020	Neural Plast. 2020 Apr 25; 2020:5701042	non pertinent
246	Xu et al.,	2020	Medicine (Baltimore). 2020 Dec 18; 99(51):e23812	non pertinent
247	Xu et al.,	2020	International journal of clinical and experimental medicine, 2020; 13(5):3531-3539	non pertinent
248	Xiong et al.,	2020	Chin J Integr Med. 2020 Aug; 26(8):609-616	non pertinent
249	Xing et al.,	2020	BMC Anesthesiol. 2020 May 19; 20(1):117	non pertinent
250	Wyndow et al.,	2020	Osteoarthritis and cartilage, 2020; 28:397-398	non pertinent
251	Wu et al.,	2020	J Nurs Scholarsh. 2020 Sep; 52(5):467-475	non pertinent
252	Wilson et al.,	2020	JAMA, 2020; 342(22):2282-2291	non pertinent
253	Williams et al.,	2020	Circulation. 2020 May 5; 141(18):1452-1462	non pertinent
254	Williams et al.,	2020	Contemporary clinical trials, 2020; 90:105935	non pertinent
255	Whi et al.,	2020	Medicine (Baltimore). 2020 Jul 2; 99(27):e21047	non pertinent
256	West et al.,	2020	Radiotherapy and oncology, 2020; 152:658-	non pertinent
257	Wenting et al.,	2020	Neural plasticity, 2020	non pertinent
258	Wendling et al.,	2020	J Rheumatol. 2020 Mar; 47(3):349-353	non pertinent

259	Wehrmeyer et al.,	2020	Journal of the American Geriatrics Society, 2020; 68(1):154-	non pertinent
260	Wang et al.,	2020	Osteoarthritis and cartilage, 2020; 28:478-479	non pertinent
261	Wang et al.,	2020	International journal of clinical and experimental medicine, 2020; 13(3):1596-1603	non pertinent
262	Wang et al.,	2020	PloS one, 2020; 15(12)	non pertinent
263	Wang et al.,	2020	Brain Behav. 2020 Aug; 10(8):e01713	non pertinent
264	Wagner et al.,	2020	Neuroimage. 2020 Oct 1; 219:117024	non pertinent
265	Wagner et al.,	2020	Hemasphere, 2020; 4:460-461	non pertinent
266	Wadley et al.,	2020	PLoS One. 2020 Dec 3; 15(12):e0243366	non pertinent
267	Voza et al.,	2020	Journal of pain research, 2020; 13:491-502	non pertinent
268	Voruz et al.,	2020	Neuropsychologia. 2020 Jul; 144:107494	non pertinent
269	Visvanathan et al.,	2020	J Stroke Cerebrovasc Dis. 2020 Aug; 29(8):104993	non pertinent
270	Vicknasingam et al.,	2020	Yale J Biol Med. 2020 Jun 29; 93(2):229-238	non pertinent
271	Venturella et al.,	2020	Human reproduction. Conference: 36th annual meeting, ESHRE, 2020; 35(1):i11	ongoing – not published yet
272	Velez et al.,	2020	Annals of oncology, 2020; 31:224-225	non pertinent
273	Varrassi et al.,	2020	Pain practice, 2020; 20(1):40-	ongoing – not published yet
274	Vaninetti et al.,	2020	Brain stimulation, 2020; 13(6):1853-	non pertinent
275	van Tilburg et al.,	2020	Gastroenterology, 2020; 158(6):907	non pertinent
276	van Hoof et al.,	2020	Frontiers in surgery, 2020; 7:5	ongoing – not published yet
277	van Heusden et al.,	2020	Journal of clinical monitoring and computing, 2020	non pertinent
278	Van Der Stouwe et al.,	2020	Schizophrenia bulletin, 2020; 46:245-	non pertinent

279	Vallone et al.,	2020	Neurologist. 2020 Nov; 25(6):162-167	non pertinent
280	Vahldiek et al.,	2020	Rofo. 2020 Oct; 192(10):945-951	non pertinent
281	van der Heide et al.,	2020	J Parkinsons Dis. 2020; 10(4): 1355-1364	non pertinent
282	Uyugr-Kucukseymen et al.,	2020	Neurophysiol Clin. 2020 Sep; 50(4):279-288	non pertinent
283	Uysal et al.,	2020	J Thorac Cardiovasc Surg. 2020 Mar; 159(3):943-953	non pertinent
284	Ulmner V	2020	J Oral Rehabil. 2020 Oct; 47(10):1202-1211	non pertinent
285	Turgeon et al.,	2020	Canadian journal of cardiology, 2020; 36(10):22-	non pertinent
286	Treffel et al.,	2020	Int J Mol Sci. 2020 May 26; 21(11):3748	non pertinent
287	Tonin et al.,	2020	Cranio. 2020 Jan; 38(1):34-42	non pertinent
288	Tong et al.,	2020	International journal of clinical and experimental medicine, 2020; 13(4):2782-2789	non pertinent
289	Togioka et al.,	2020	Br J Anaesth. 2020 May; 124(5):553-561	non pertinent
290	Thomas et al.,	2020	Journal of Alzheimer's disease, 2020; 75(2):617-631	non pertinent
291	Themelis et al.,	2020	Psychosomatic medicine, 2020; 82(6):A162-	non pertinent
292	Thamkhantho et al.,	2020	Chotmaihet thangphaet (Journal of the Medical Association of Thailand), 2020; 103(5):442-448	non pertinent
293	Teixeira et al.,	2020	Scand J Pain. 2020 Oct 25; 20(4):651-661	non pertinent
294	Teferi et al.,	2020	Biological psychiatry, 2021; 89(9):289-	non pertinent
295	Tawfeek et al.,	2020	Central european journal of urology, 2020; 73(4):1-7	non pertinent
296	Tang et al.,	2020	BMJ open, 2020; 10(3):e034764	non pertinent
297	Tang et al.,	2020	Trials. 2020 May 24; 21(1):421	non pertinent

298	Tam et al.,	2020	Vasc Med. 2020 Jun; 25(3):223-225	non pertinent
299	Tadrous et al.,	2020	JAMA Netw Open. 2020 May 1; 3(5):e205724	non pertinent
300	Suso-Marti et al.,	2020	Physiol Behav. 2020 Mar 1; 215:112774	non pertinent
301	Sun et al.,	2020	Translational psychiatry, 2020; 10(1)	non pertinent
302	Suffoletto et al.,	2020	Pain Med. 2020 Nov 7; 21(7):1393-1399	non pertinent
303	Strand et al.,	2020	Arthritis Res Ther. 2020 Oct 15; 22(1):243	non pertinent
304	Stewart et al.,	2020	Obstetrics and gynecology, 2020; 135:27	non pertinent
305	Stewart et al.,	2020	Journal of minimally invasive gynecology, 2020; 27(7):8-	non pertinent
306	Stetzelberger et al.,	2020	Clinical orthopaedics and related research, 2020	non pertinent
307	Stenzl et al.,	2020	European urology open science, 2020; 19:e1701-	non pertinent
308	Stankovic et al.,	2020	Experimental brain research, 2020; 238(11):2641-2651	non pertinent
309	Soni et al.,	2020	Rheumatology (Oxford). 2020 Aug 1; 59(8):2074-2084	non pertinent
310	Somaiah et al.,	2020	Oncoimmunology. 2020 Nov 19; 9(1):1847846	non pertinent
311	Soh et al.,	2020	J Clin Monit Comput. 2020 Aug; 34(4):715-723	non pertinent
312	Smet et al.,	2020	Clinical neurophysiology, 2020; 131(4)	non pertinent
313	Skvortsova et al.,	2020	PLoS One. 2020 Mar 19; 15(3):e0229692	non pertinent
314	Singh et al.,	2020	JAMA cardiology, 2020	non pertinent
315	Simis et al.	2020	Clinical neurophysiology, 2020; 131(8):1806-1814	non pertinent

316	Siegel et al.,	2020	Lancet Psychiatry. 2020 Nov; 7(11):971-981	non pertinent
317	Sidhu et al.,	2020	Orphanet J Rare Dis. 2020 Sep 16; 15(1):251	non pertinent
318	Siebenga et al.,	2020	Clin Transl Sci. 2020 Mar; 13(2):318-324	non pertinent
319	Shrestha et al.	2020	Chest, 2020; 158(4):A710-	non pertinent
320	Shin et al.,	2020	BMC Anesthesiol. 2020 Feb 26; 20(1):49	non pertinent
321	Sharpe et al.,	2020	Global advances in health and medicine, 2020; 9:103-	non pertinent
322	Sharma et al.,	2020	Indian journal of ophthalmology, 2020; 68(4):577-582	ongoing – not published yet
323	Shahidi et al.,	2020	Spine (Phila Pa 1976). 2020 Feb 15; 45(4):E189-E195	ongoing – not published yet
324	Seward et al.,	2020	Journal of orthopaedic surgery and research, 2020; 15(1):531	not published yet
325	Sevinc et al.,	2020	Global advances in health and medicine, 2020; 9:25-	ongoing – not published yet
326	Sevinc et al.,	2020	Brain Behav. 2020 Sep; 10(9):e01766	initial ethics approval
327	Sevel et al.,	2020	Brain Imaging Behav. 2020 Jun; 14(3):881-896	non pertinent
328	Seward et al.,	2020	J Orthop Surg Res. 2020 Nov 13; 15(1):531	non pertinent
329	Serrano et al.,	2020	Journal of pain research, 2020; 13:2297-2311	non pertinent
330	Seminowicz et al.,	2020	Pain, 2020; 161(8):1837-1846	non pertinent
331	Seminowicz et al.,	2020	Pain. 2020 Aug; 161(8):1837-1846	non pertinent
332	Seemann et al.,	2020	Rofo. 2020 Apr; 192(4):335-342	non pertinent
333	Schwab et al.,	2020	Psychiatry Res Neuroimaging. 2020 Jun 30; 300:111066	non pertinent
334	Schwarzmeier et al.,	2020	Int J Methods Psychiatr Res. 2020 Jun; 29(2):e1812	non pertinent

335	Schneider et al.,	2020	European journal of pain (united kingdom), 2020; 24(5):945-955	non pertinent
336	Schmid et al.,	2020	Annals of oncology, 2020; 31:81-	non pertinent
337	Schindler et al.,	2020	Psychopharmacology (Berl). 2020 Oct; 237(10):3097-3107	non pertinent
338	Schimanski et al.,	2020	Oncoimmunology, 2020; 9(1)	non pertinent
339	Scheuzger et al.,	2020	Crit Care. 2020 Jan 17; 24(1):18	non pertinent
340	Schmid et al.,	2020	Br J Anaesth. 2020 Sep; 125(3):330-335	non pertinent
341	Schett et al.,	2020	The lancet rheumatology, 2020; 2(10):e623-e632	non pertinent
342	Schertz et al.,	2020	Clinical neurophysiology, 2020; 131(4):e16-e17	non pertinent
343	Scherrenberg et al.,	2020	Acta clinical Belgica, 2020; 75:35-36	non pertinent
344	Schenker et al.,	2020	Annals of the rheumatic diseases, 2020; 79(1):135-136	non pertinent
345	Santocchi et al.,	2020	Frontiers in psychiatry, 2020; 11	non pertinent
346	Sanghera et al.,	2020	Radiotherapy and oncology, 2020; 150:23-24	non pertinent
347	Sanghera et al.,	2020	International journal of radiation oncology biology physics, 2020; 108(3):e21-e22	non pertinent
348	Sander et al.,	2020	JAMA Psychiatry. 2020 Oct 1; 77(10):1001-1011	non pertinent
349	Sanabria-Mazo et al.,	2020	Journal of clinical medicine, 2020; 9(10):1-16	non pertinent
350	Saleh et al.,	2020	NMR Biomed. 2020 Apr; 33(4):e4227	non pertinent
351	Salas Noain et al.,	2020	American journal of gastroenterology, 2020; 115:1628-	non pertinent
352	Salamone et al.,	2020	BMJ open, 2020; 10(3)	non pertinent
353	Ruchman et al.,	2020	Journal of general internal medicine, 2020; 35(1):532-	non pertinent

354	Ruan et al.	2020	Osteoarthritis and cartilage, 2020; 28:473-474	non pertinent
355	Rovati et al.,	2020	Osteoarthritis and cartilage, 2020; 28(1):22-30	non pertinent
356	Rodrigues et al.,	2020	Lasers in medical science, 2020; 35(1):269-	non pertinent
357	ROCCS	2020	Lancet. 2020 Feb 8; 395(10222):417-426	non pertinent
358	Rivas et al.,	2020	J Therm Biol. 2020 Dec; 94:102741	non pertinent
359	Rios-Diaz et al.,	2020	Lancet (london, england), 2020; 395(10222):417-426	non pertinent
360	Riordan et al.,	2020	Implementation science, 2020; 15(1):34-	non pertinent
361	Ridderbusch et al.,	2020	Neuroimage: clinical, 2020; 27	non pertinent
362	Ribeiro et al.,	2020	Occup Environ Med. 2020 Jul; 77(7):462-469	non pertinent
363	Ree et al.,	2020	Eur J Pain. 2020 Jan; 24(1):110-121	non pertinent
364	Redmond et al.,	2020	Int J Radiat Oncol Biol Phys. 2020 Feb 1; 106(2):261-268	non pertinent
365	Rech et al.,	2020	Annals of the rheumatic diseases, 2020; 79(1):77-78	non pertinent
366	Rastogi et al.,	2020	Toxicol Appl Pharmacol. 2020 May 15; 395:114976	non pertinent
367	Ramesh et al.,	2020	Movement disorders, 2020; 35(1):474-	non pertinent
368	Ramaseshan et al.,	2020	Am J Obstet Gynecol. 2020 Aug; 223(2):262.e1-262.e8	non pertinent
369	Raque-Bogda et al.,	2020	J Psychosoc Oncol. 2020 Nov-Dec; 38(6):649-669	non pertinent
370	Rapcan et al.,	2020	Wien Klin Wochenschr. 2020 Sep; 132(17-18):526-534	non pertinent
371	Raju et al.,	2020	BMC Anesthesiol. 2020 Sep 3; 20(1):222	non pertinent
372	Rae Olmsted et al.,	2020	JAMA Psychiatry. 2020 Feb 1; 77(2):130-138	non pertinent

373	Rabinak et al.,	2020	Psychopharmacology (Berl). 2020 Jun; 237(6):1813-1826	non pertinent
374	Qiao et al.,	2020	Neuroscience letters, 2020; 738	non pertinent
375	Pruiksma et al.,	2020	J Clin Sleep Med. 2020 Jan 15; 16(1):29-40	non pertinent
376	Pollonini et al.,	2020	J Neuroimaging. 2020 Nov; 30(6):808-814	non pertinent
377	Pleus et al.,	2020	IBROS reports, 2020; 9:78-84	non pertinent
378	Platts-Mills et al.,	2020	Trials. 2020 Jul 6; 21(1):615	non pertinent
379	Planchuelo-Gomez et al.,	2020	J Headache Pain. 2020 Jan 2; 21(1):1	non pertinent
380	Pieracci et al.,	2020	J Trauma Acute Care Surg. 2020 Feb; 88(2):249-257	non pertinent
381	Pielkenrood et al.,	2020	Radiotherapy and oncology, 2020; 152:201-202	non pertinent
382	Petrone et al.,	2020	Spine journal, 2020; 20(9):200	non pertinent
383	Petersen et al.,	2020	Clin J Pain. 2020 Jan; 36(1):34-40	non pertinent
384	Pernicova et al.,	2020	The lancet. Diabetes & endocrinology, 2020; 8(4):278-291	non pertinent
385	Perez-Llanes et al.,	2020	Physiotherapy theory and practice, 2020; 1-10	non pertinent
386	Peng et al.,	2020	Neuroimage Clin. 2020; 28:102454	non pertinent
387	Peabody et al.,	2020	Diagnostics, 2020; 18(8)	non pertinent
388	Paulus et al.,	2020	Neuropsychopharmacology, 2020	non pertinent
389	Patterson et al.,	2020	American journal of emergency medicine, 2020	non pertinent
390	Patel et al.,	2020	Am J Respir Crit Care Med. 2020 Sep 1; 202(5):690-699	non pertinent
391	Patel et al.,	2020	Ann Surg. 2020 Aug; 272(2):284-287	non pertinent
392	Patcharatrakul et al.,	2020	Gastroenterology, 2020; 158(6):1148	non pertinent
393	Patcharatrakul et al.,	2020	Nutrients. 2020 Dec 4; 12(12):3740	non pertinent
394	Paskins et al.,	2020	Osteoarthritis and cartilage, 2020; 28:479-	non pertinent

395	Paskins et al.,	2020	Rheumatology (united kingdom), 2020; 59:ii2-	non pertinent
396	Park et al.,	2020	Female pelvic medicine & reconstructive surgery, 2020; 26(10):95-	non pertinent
397	Park et al.,	2020	Medicine (Baltimore). 2020 Sep 4; 99(36):e21823	non pertinent
398	Pardanani et al.,	2020	Blood, 2020; 136(1):10-12	non pertinent
399	Paravlic et al.,	2020	J Orthop Surg Res. 2020 Oct 2; 15(1):451	non pertinent
400	Pape et al.,	2020	Therapeutic advances in gastroenterology, 2020; 13	non pertinent
401	Pajediene et al.,	2020	Med Hypotheses. 2020 Mar; 136:109512	non pertinent
402	Owen et al.,	2020	Eur Spine J. 2020 Aug; 29(8):1887-1899	non pertinent
403	Oliva et al.,	2020	BMJ. 2020 Mar 4; 368:m283	non pertinent
404	Ogdie et al.,	2020	Annals of the rheumatic diseases, 2020; 79(1):1477-	non pertinent
405	O'Gara et al.,	2020	Anesth Analg. 2020 Mar; 130(3):586-595	non pertinent
406	Obert et al.,	2020	Scientific reports, 2020; 10(1):19444	non pertinent
407	Nurko et al.,	2020	Gastroenterology, 2020; 158(6):161-	non pertinent
408	Nouri-Vaskeh et al.,	2020	Complement Ther Med. 2020 Mar: 49:102351	non pertinent
409	Nord et al.,	2020	Current biology, 2020	non pertinent
410	Nielsen et al.,	2020	Acta Anaesthesiol Scand. 2020 Mar; 64(3):368-377	non pertinent
411	Nguy et al.,	2020	J Parkinsons Dis. 2020; 10(3):1161-1170	non pertinent
412	Nasb et al.,	2020	BMC Musculoskelet Disord. 2020 Jan 15; 21(1):33	non pertinent
413	Nanda et al.,	2020	Topics in antiviral medicine, 2020; 28(1):399-400	non pertinent
414	Naidoo et al.,	2020	Journal of acquired immune deficiency syndromes, 2020	non pertinent

415	Nah et al.,	2020	British journal of educational psychology, 2020	non pertinent
416	Naderi et al.,	2020	Iranian journal of psychiatry and behavioral sciences, 2020; 14(3)	non pertinent
417	Mutubuki et al.,	2020	Physiother Res Int. 2020 Jan; 25(1):e1796	non pertinent
418	Muriel-Sanchez et al.,	2020	Journal of clinical medicine, 2020; 9(3)	non pertinent
419	Muller et al.,	2020	Pract Radiat Oncol. 2020 Nov-Dec; 10(6):e444-e451	non pertinent
420	Mulder et al.,	2020	Antimicrobial resistance and infection control, 2020; 9(1):84	non pertinent
421	Morriss et al.,	2020	BMJ Open. 2020 Jul 7; 10(7):e038430	non pertinent
422	Morasco et al.,	2020	Contemp Clin Trials. 2020 Mar; 90:105957	non pertinent
423	Morales Tejera et al.,	2020	Somatosens Mot Res. 2020 Sep; 37(3):138-148	non pertinent
424	Moller et al.,	2020	Sci Rep. 2020 Jun 16; 10(1):9710	non pertinent
425	Miscusi et al.,	2020	Eur Spine J. 2020 Jun; 29(6):1410-1415	non pertinent
426	Mioduszewski et al.,	2020	Journal of cancer survivorship, 2020	non pertinent
427	Metcalfe et al.,	2020	BMJ open, 2020; 10(5)	non pertinent
428	Mehta et al.,	2020	Female pelvic medicine & reconstructive surgery, 2020; 26(10):159-160	non pertinent
429	Mehta et al.,	2020	JAMA Netw Open. 2020 Dec 1; 3(12):e2028328	non pertinent
430	McCrae et al.,	2020	BMJ Open. 2020 Sep 15; 10(9):e033760	non pertinent
431	McClung et al.,	2020	Journal of bone and mineral research, 2020; 35(1):245-246	non pertinent
432	Mavromatis et al.,	2020	Clinical neurophysiology, 2020; 131(4):e136-	non pertinent
433	Maurer et al.,	2020	Int J Environ Res Public Health. 2020 Sep 18; 17(18):6801	non pertinent

434	Mattia et al.,	2020	BMC Neurol. 2020 Jun 27; 20(1):254	non pertinent
435	Martins et al.,	2020	Int J Environ Res Public Health. 2020 Feb 15; 17(4):1258	non pertinent
436	Mathew et al.,	2020	Neuroregulation, 2020; 7(1):30-44	non pertinent
437	Mathersul et al.,	2020	Global advances in health and medicine, 2020; 9	non pertinent
438	Marra et al.,	2020	J Urol. 2020 Dec; 204(6):1209-1215	non pertinent
439	Marker et al.,	2020	Diabetes Technol Ther. 2020 Jan; 22(1):25-33	non pertinent
440	Maranesi et al.,	2020	BMJ open, 2020; 10(6)	non pertinent
441	Manchikanti et al.,	2020	Pain physician, 2020; 23(3):1-127	non pertinent
442	Malm et al.,	2020	BMC Neurol. 2020 Aug 20; 20(1):311	non pertinent
443	Maier et al.,	2020	Frontiers in psychiatry, 2020; 11	non pertinent
444	Mahanna-Gabrielli et al.,	2020	Anesth Analg. 2020 Jun; 130(6):1516-1523	non pertinent
445	Madsen et al.,	2020	Trials. 2020 Sep 22; 21(1):805	non pertinent
446	Machingaidze et al.,	2020	PLoS One. 2020 Sep 28; 15(9):0239731	non pertinent
447	Mace et al.,	2020	Gerontologist, 2020	non pertinent
448	Ma et al.,	2020	Circulation, 2020; 141(1)	non pertinent
449	Lyu et al.,	2020	Orthop Surg. 2020 Oct; 12(5): 1430-1438	non pertinent
450	Lübke et al.,	2020	Arch Sex Behav. 2020 Feb; 49(2):635-644	non pertinent
451	Luo et al.,	2020	Neural plasticity, 2020; 2020:13	non pertinent
452	Lukes et al.,	2020	Obstetrics and gynecology, 2020; 135:7-	non pertinent
453	Lui et al.,	2020	Journal of general internal medicine, 2020; 35(1):574-	non pertinent
454	Lübke et al.,	2020	Archives of sexual behavior, 2020; 49(2):635-644	non pertinent
455	Lu et al.,	2020	Intern Med. 2020 Oct 15; 59(20):2471-2480	non pertinent
456	Lowenstern et al.,	2020	JAMA Cardiol. 2020 Feb 1; 5(2):193-201	non pertinent

457	Lorenzin et al.,	2020	Front Immunol. 2020 May 15; 11:936	non pertinent
458	Lopez-de-Uralde-Villanueva et al.,	2020	Musculoskelet Sci Pract. 2020 Jun; 47:102138	non pertinent
459	Loh et al.,	2020	World journal of surgery, 2020; 44(8):2656-2666	non pertinent
460	Llewellyn et al.,	2020	Neuroregulation, 2020; 7(4):165-166	non pertinent
461	Liu et al.	2020	Chinese journal of tissue engineering research, 2020; 24(35):5688-5694	non pertinent
462	Linnemayr et al.	2020	AIDS and behavior, 2020	non pertinent
463	Lindinger et al.,	2020	Alcoholism, clinical and experimental research, 2020	non pertinent
464	Limakatso et al.,	2020	Physiotherapy. 2020 Dec; 109:65-74	non pertinent
465	Lim et al.,	2020	J Int Med Res. 2020 Apr; 48(4):300060519893165	non pertinent
466	Lier et al.,	2020	Sci Rep. 2020 Jun 3; 10(1):9067	non pertinent
467	Lieberz et al.,	2020	Neuropsychopharmacology. 2020 Jun; 45(7):1134-1140	non pertinent
468	Liao et al.,	2020	Ann Thorac Surg. 2020 Mar; 109(3):887-893	non pertinent
469	Li et al.,	2020	Arthritis Care Res (Hoboken). 2020 Dec; 72(12):1755-1765	non pertinent
470	Li et al.,	2020	J Tradit Chin Med. 2020 Dec; 40(6):1041-1051	non pertinent
471	Li et al.,	2020	Trials. 2020 Nov 30; 21(1):989	non pertinent
472	Li et al.,	2020	Medical Science monitor, 2020; 26:e926833	non pertinent
473	Li et al.,	2020	Neuromodulation, 2020; 23(6):838-846	non pertinent
474	Li et al.,	2020	Zhonggou zhen jiu (Chinese acupuncture & moxibustion), 2020; 40(12):1281-1285	non pertinent
475	Letafatkar et al.,	2020	Scand J Med Sci Sports. 2020 Mar; 30(3):429-441	non pertinent

476	Leiter et al.,	2020	Orthopaedic journal of sports medicine, 2020; 8(9)	non pertinent
477	Lee-Archer et al.,	2020	Anaesthesia. 2020 Nov; 75(11):1461-1468	non pertinent
478	Lee et al.,	2020	Clin Implant Dent Relat Res. 2020 Oct;22(5):622-630	non pertinent
479	Lee et al.,	2020	Spine journal, 2020; 20(7):1114-1124	non pertinent
480	Lee et al.,	2020	Clin Oral Implants Res. 2020 Jan; 31(1):1-9	non pertinent
481	La Touche et al.,	2020	Pain Med. 2020 Oct 1; 21(10):2186-2199	non pertinent
482	La Touche et al.,	2020	J Mot Behav. 2020; 52(4):444-455	non pertinent
483	Laslett et al.,	2020	Trials. 2020 Jan 14; 21(1):79	non pertinent
484	Larsen et al.,	2020	BMC Cancer. 2020 Mar 3; 20(1):169	non pertinent
485	Larsen et al.,	2020	Osteoarthritis and cartilage, 2020; 28:173-	non pertinent
486	Lannin et al.,	2020	Stroke. 2020 Feb 51(2):556-562	non pertinent
487	Lange et al.,	2020	Neuropsychopharmacology. 2020 Feb; 45(3):534-541	non pertinent
488	Lam et al.,	2020	Echocardiography. 2020 Aug; 37(8):1199-1204	non pertinent
489	Lakshmanan et al.,	2020	Journal of cardiovascular computed tomography, 2020; 14(3):47-	non pertinent
490	Labau et al.,	2020	Brain. 2020 Mar 1; 143(3):771-782	non pertinent
491	Kwiatek et al.,	2020	Internal medicine journal, 2020; 50(2):48-49	non pertinent
492	Kurosawa et al.,	2020	Pain Med. 2020 Oct 1; 21(10):2105-2110	non pertinent
493	Kundranda et al.,	2020	Annals of oncology: offical journal of the european society for medical oncology, 2020; 31(1):79-87	non pertinent
494	Krishnamoorthy et al.,	2020	J Neurosurg Anesthesiol. 2020 Apr; 32(2):156-161	non pertinent

495	Kreuder et al.,	2020	Proc Natl Acad Sci U S A. 2020 May 26; 117(21):11781-11787	non pertinent
496	Kozinc et al.,	2020	Appl Ergon. 2020 Jul; 86:103117	non pertinent
497	Kou et al.,	2020	Psychological medicine, 2020; 1-10	non pertinent
498	Kothari et al.,	2020	Brain Stimul. 2020 May-Jun; 13(3):554-561	non pertinent
499	Korpershoek et al.,	2020	Trials, 2020; 21(1)	non pertinent
500	Koppenaal et al.,	2020	BMC Musculoskelet Disord. 2020 Apr 22; 21(1):265	non pertinent
501	Kopacz et al.,	2020	Pain Res Manag. 2020 Oct 10; 2020:1036306	non pertinent
502	Kondo et al.,	2020	Journal of knee surgery, 2020; https://doi.org/10.1055/s-0040-1721035	non pertinent
503	Kohns et al.,	2020	Clin J Pain. 2020 Sep; 36(9):683-692	non pertinent
504	Kohnen et al.,	2020	J Am Med Dir Assoc. 2020 Nov; 21(11):1643-1650	non pertinent
505	Kobbernagel et al.,	2020	The lancet. Respiratory medicine, 2020; 8(5):493-505	non pertinent
506	Kitagawa et al.,	2020	Journal of nutritional science and vitaminology, 2020; 66(5):417-426	non pertinent
507	Kim et al.,	2020	J Neurointerv Surg. 2020 Nov; 12(11):1137-1141	non pertinent
508	Kim et al.,	2020	Neuroimage. 2020 Aug 15; 217:116899	non pertinent
509	Kim et al.,	2020	Neurosurgery. 2020 Jun 1; 86(6):825-834	non pertinent
510	Kim et al.,	2020	Pain Med. 2020 Apr 1; 21(4):677-685	non pertinent
511	Kim et al.,	2020	Abdom Radiol (NY). 2020 Mar; 45(3):623-631	non pertinent
512	Kim et al.,	2020	Psychological medicine, 2020; 1-10	non pertinent
513	Kim et al.,	2020	Integrative medicine research, 2020; 9	non pertinent

514	Killgore et al.,	2020	Biological psychiatry, 2020; 87(9):417-	non pertinent
515	Khalsa et al.,	2020	Psychosomatic medicine, 2020; 82(6):A163-A164	non pertinent
516	Khalifeh et al.,	2020	J Matern Fetal Neonatal Med. 2020 Feb; 33(4):542-546	non pertinent
517	Khalfa et al.,	2020	Am J Clin Nutr. 2020 Jan 1; 111(1):131-140	non pertinent
518	Kempton et al.,	2020	J Am Acad Orthop Surg. 2020 Sep 15; 28(18):772-779	non pertinent
519	Keene et al.,	2020	Physiotherapy. 2020 Jun; 107:252-266	non pertinent
520	Karpouzas et al.,	2020	Arthritis Rheumatol. 2020 Sep; 72(9):1467-1475	non pertinent
521	Karady et al.,	2020	JAMA Netw Open. 2020 Dec 1; 3(12):e2028312	non pertinent
522	Kalbe et al.,	2020	Parkinson's disease, 2020; 2020	non pertinent
523	Kaiser et al.,	2020	Br J Anaesth. 2020 Sep; 125(3):291-297	non pertinent
524	Ji-Wen et al.,	2020	Shanghai kou qiang yi xue [Shanghai journal of stomatology], 2020; 29(6):623-627	non pertinent
525	Jin et al.,	2020	Osteoporos Int. 2020 Sep; 31(9):1721-1732	non pertinent
526	Ji et al.,	2020	International journal of clinical and experimental medicine, 2020; 13(2):925-932	non pertinent
527	Jarvik et al.,	2020	JAMA Netw Open. 2020 Sep 1; 3(9):e2015713	non pertinent
528	Jaikaran et al.,	2020	BMJ case reports, 2020; 13(12)	non pertinent
529	Izquierdo-Alventosa et al.,	2020	Int J Environ Res Public Health. 2020 May 21; 17(10):3634	non pertinent
530	Ispirgil et al.,	2020	Cranio. 2020 Mar; 38(2):99-108	non pertinent
531	Ismail et al.,	2020	Chest, 2020; 158(4):A2075-A2076	non pertinent
532	Ishii et al.,	2020	Acta Med Okayama. 2020 Feb; 74(1):17-26	non pertinent

533	Iljazi et al.,	2020	Cephalalgia. 2020 Jul; 40(8):818-827	non pertinent
534	Imam et al.,	2020	Journal of shoulder and elbow surgery, 2020; 29(10):2015-2026	non pertinent
535	Hwang et al.,	2020	Knee Surg Sports Traumatol Arthrosc. 2020 Jul; 28(7):2343-2353	non pertinent
536	Hurd et al.,	2020	J Orthop Surg Res. 2020 Mar 30; 15(1):122	non pertinent
537	Huh et al.,	2020	Medicine (Baltimore). 2020 Jan; 99(5):e18441	ongoing
538	Houde et al.,	2020	Neurobiol Learn Mem. 2020 Mar; 169:107174	non pertinent
539	Houbiers et al.,	2020	World journal of urology, 2020	non pertinent
540	Hosseinzadeh et al.,	2020	Clinical orthopaedics and related research, 2020	non pertinent
541	Hosomi et al.,	2020	Pain. 2020 Feb; 161(2):351-360	non pertinent
542	Hood et al.,	2020	Blood, 2020; 136(1):57-58	non pertinent
543	Hong et al.,	2020	Dermatol Surg. 2020 Feb; 46(2):213-219	non pertinent
544	Holmberg et al.,	2020	Spine (Phila Pa 1976). 2020 Dec 15; 45(24):1751-1757	non pertinent
545	Holden et al.,	2020	Rheumatology (united kingdom), 2020; 59:ii65-ii66	non pertinent
546	Holden et al.,	2020	Osteoarthritis and cartilage, 2020; 28:499-	non pertinent
547	Hill et al.,	2020	Diseases of the colon and rectum, 2020; 63(6):e325-e326	non pertinent
548	Herzig et al.,	2020	Scandinavian journal of pain, 2020	non pertinent
549	Henry et al.,	2020	Journal of general internal medicine, 2020; 35(1):104-	non pertinent
550	Henrich et al.,	2020	J Consult Clin Psychol. 2020 Apr; 88(4):295-310	non pertinent
551	Hemani et al.,	2020	Neuromodulation, 2020; 23(3):e320-	non pertinent
552	Herzog et al.,	2020	Danish medical journal, 2020; 67(1)	non pertinent

553	Hedstrom et al.,	2020	Pancreatology, 2020; 20:86-87	non pertinent
554	Heany et al.,	2020	Experimental brain research, 2020; 238(3):751-759	non pertinent
555	He et al.,	2020	Osteoarthritis and cartilage, 2020; 28:57-	non pertinent
556	Hauser-Ulrich et al.,	2020	JMIR Mhealth Uhealth. 2020 Apr 3; 8(4):e15806	non pertinent
557	Hasegawa et al.,	2020	Spine (Phila Pa 1976). 2020 Aug 1; 45(15):E892-E902	non pertinent
558	Hagiwata et al.,	2020	Brain Stimul. 2020 Jan-Feb; 13(1):60-68	non pertinent
559	Hack et al.,	2020	Breast. 2020 Apr; 50:11-18	non pertinent
560	Guiote Moreno et al.,	2020	European journal of nuclear medicine and molecular imaging, 2020; 47(1):533-	non pertinent
561	Guensch et al.,	2020	J Am Heart Assoc. 2020 Mar 3; 9(5):e014739	non pertinent
562	Guangming	2020	Journal of digestive diseases, 2020; 21(1):30-	non pertinent
563	Gu et al.,	2020	Medical science monitor, 2020; 26	non pertinent
564	Green et al.,	2020	Postgraduate medicine, 2020; 132(1):19-21	non pertinent
565	Gratzke et al.,	2020	Journal for immunotherapy of cancer, 2020; 8(3):A211-	non pertinent
566	Graff et al.,	2020	Journal for immunotherapy of cancer, 2020; 8(3):A210-	non pertinent
567	Graff et al.,	2020	Journal of clinical oncology, 2020; 38(6)	non pertinent
568	Goudman et al.,	2020	Neuromodulation. 2020 Jan; 23(1):74-81	non pertinent
569	Gossrau et al.,	2020	Pain Med. 2020 Oct 1; 21(10):2546-2551	non pertinent
570	Gorka et al.,	2020	Psychopharmacology (Berl). 2020 Jun; 237(6):1873-1883	non pertinent
571	Goldstein et al.,	2020	Neurotherapeutics. 2020 Jul; 17(3):774-783	non pertinent

572	Gkolias et al.,	2020	Eur J Pain. 2020 Oct; 24(9):1716-1729	non pertinent
573	Gitlin et al.,	2020	Anesthesiology. 2020 Nov 1; 133(5):1021-1028	non pertinent
574	Gilmore et al.,	2020	Neuromodulation, 2020; 23(3):e48-e49	non pertinent
575	Gilbert et al.,	2020	Biol Psychiatry Cogn Neurosci Neuroimaging. 2020 Mar; 5(3):354-363	non pertinent
576	Gjefsen et al.,	2020	BMC musculoskeletal disorders, 2020; 21(1)	non pertinent
577	Gillespie et al.,	2020	Pilot and feasibility studies, 2020; 6(1)	non pertinent
578	Ghelman et al.,	2020	Brain Behav. 2020 Apr; 10(4):e01590	non pertinent
579	Ghanizada et al.,	2020	J Headache Pain. 2020 Feb 24; 21(1):19	non pertinent
580	Gezginaslan et al.,	2020	Archives of rheumatology, 2020; 35(1):78-89	non pertinent
581	Gelauff et al.,	2020	Parkinsonism Relat Disord. 2020 Jan; 70:1-6	non pertinent
582	Garen et al.,	2020	Annals of the rheumatic diseases, 2020; 79(1):1278-	non pertinent
583	Garcia-Azorin et al.,	2020	Medicine (Baltimore). 2020 Jul 17; 99(29):e20900	non pertinent
584	Ganesh et al.,	2020	BMJ Open. 2020 Oct 14; 10(10):e040466	non pertinent
585	Ganer Herman et al.,	2020	Obstet Gynecol. 2020 Jun; 135(6):1345-1352	non pertinent
586	Gamba et al.,	2020	Foot Ankle Int. 2020 Mar; 41(3):267-274	non pertinent
587	Frey et al.,	2020	Neuropsychopharmacology, 2020; 45(9):1431-1437	non pertinent
588	Franettovich et al.,	2020	J Foot Ankle Res. 2020 May 8; 13(1):20	non pertinent
589	Foster et al.,	2020	Health Technol Assess. 2020 Oct; 24(49):1-130	non pertinent
590	Fitzpatrick et al.,	2020	Drug design, development and therapy, 2020; 14:2707-2713	non pertinent

591	Fitzke et al.,	2020	United european gastroenterology journal, 2020; 8(8):548-	non pertinent
592	Fillingim et al.,	2020	Contemporary clinical trials, 2020; 98	not published yet
593	Ferdinandov et al.,	2020	Revmatologiia (bulgaria), 2020; 28(3):39-53	not published yet
594	Farrokhi et al.,	2020	Pain medicine (Malden, Mass.), 2020; 21(2):45-52	non pertinent
595	Faridi et al.,	2020	Circ Cardiovasc Qual Outcomes. 2020 Mar; 13(3):e006275	non pertinent
596	Fan et al.,	2020	J Thorac Cardiovasc Surg. 2020 Aug; 160(2):532-539.e2	non pertinent
597	Elmallawany et al.,	2020	Open access macedonian journal of medical science, 2020; 8:807-814	trial ongoing
598	Eckerdal et al.,	2020	Anesth Analg. 2020 Mar; 130(3):615-624	trial ongoing
599	Ebrahimi et al.,	2020	Neuropsychopharmacology. 2020 Feb; 45(3):499-506	no results posted
600	Durst et al.,	2020	Osteoarthritis and cartilage, 2020; 28:463-	study ongoing
601	Duff et al.,	2020	Lancet Digit Health. 2020 Aug 24; 2(9):e458-e467	trial ongoing
602	Drukker et al.,	2020	Journal of psychosomatic research, 2020; 139	trial ongoing
603	Drechsler et al.,	2020	Nephrology dialysis transplantation, 2020; 35(3):iii351-	non pertinent
604	Douglas et al.,	2020	Chest. 2020 Oct; 158(4): 1431-1445	trial ongoing
605	Dory et al.,	2020	Academic medicine, 2020	trial ongoing
606	Donthula et al.,	2020	Clin Orthop Relat Res. 2020 Feb; 478(2):381-388	trial ongoing
607	Dindo et al.,	2020	Contemp Clin Trials. 2020 Mar; 90:105954	non pertinent
608	Dieras et al.,	2020	The lancet. Oncology, 2020; 21(10):1269-1282	non pertinent

609	D'Haens et al.,	2020	United european gastroenterology journal, 2020; 8(8):409-410	trial ongoing
610	D'Haens et al.,	2020	Gastroenterology, 2020; 158(6):437	not published
611	de Zoete et al.,	2020	Phys Ther. 2020 Feb 7; 100(2):268-282	not published
612	Dezfuli et al.,	2020	Pakistan journal of medical and health sciences, 2020; 14(2):701-704	non pertinent
613	DeSouza et al.,	2020	Brain Struct Funct. 2020 Jan; 225(1):161-172	trial ongoing
614	Derksen et al.,	2020	Journal of cachexia, sarcopenia and muscle, 2020	non pertinent
615	Depaulis et al.,	2020	Trials, 2020; 10(7)	non pertinent
616	de Melo et al.,	2020	Sci Rep. 2020 Nov 3; 10(1):18955	trial ongoing
617	De Groote et al.,	2020	Neuromodulation. 2020 Jan; 23(1):46-55	trial ongoing
618	Deftereos et al.,	2020	Hellenic J Cardiol. 2020 Jan-Feb; 61(1):42-45	trial ongoing
619	Day et al.,	2020	Contemp Clin Trials. 2020 Jun; 93:106000	trial ongoing
620	Day et al.,	2020	J Pain. 2020 Jan-Feb; 21(1-2):161-169	non pertinent
621	Davies et al.,	2020	Transl Psychiatry. 2020 Sep 13; 10(1):311	trial ongoing
622	Darracq	2020	Am J Emerg Med. 2020 Dec; 38(12):2759.e1-2759.e4	non pertinent
623	Darnall et al.,	2020	Pain Med. 2020 Aug 1; 21(8):1523-1531	non pertinent
624	Daniel et al.,	2020	Sci Rep. 2020 Dec 14; 10(1):21840	trial ongoing
625	Damschroder et al.,	2020	J Med Internet Res. 2020 Aug 4; 22(8):e19216	non pertinent
626	Dalla Volta et al.	2020	Frontiers in neurology, 2020, 11	trial ongoing
627	Dafsari et al.,	2020	Brain Stimul. 2020 Nov-Dec; 13(6):1697-1705	non pertinent
628	Cybinski et al.,	2020	Pharmacopsychiatry, 2020; 53(2):92-	trial ongoing

629	Courties et al.,	2020	Arthritis and rheumatology, 2020; 72(10):2205-2207	trial ongoing
630	Cormack et al.,	2020	Br J Clin Pharmacol. 2020 Jul; 86(7):1387-1397	non pertinent
631	Corbelli et al.,	2020	J Headache Pain. 2020 Jan 16; 21(1):6	non pertinent
632	Clinton et al.,	2020	Chest, 2020; 158(4):A1861-	trial ongoing
633	Cisler et al.,	2020	Translational psychiatry, 2020; 10(1)	trial ongoing
634	Cillessen et al.,	2020	J Med Internet Res. 2020 Oct 2; 22(10):e17526	non pertinent
635	Cho et al.,	2020	Gastrointestinal endoscopy, 2020; 91(6):AB187-AB188	non pertinent
636	Chihai et al.	2020	Archives of the balkan medical union, 2020; 55(4):634-641	non pertinent
637	Cheung et al.,	2020	Complement Ther Clin Pract. 2020 Nov; 41:101254	non pertinent
638	Chen et al.,	2020	BMC Public Health. 2020 Sep 1; 20(1):1332	non pertinent
639	Chen et al.,	2020	European journal of pharmaceutical sciences, 2020; 151:105384	non pertinent
640	Chen et al.,	2020	Neuropsychopharmacology, 2020; 45(12):2070-2078	non pertinent
641	Chatterjee et al.,	2020	International journal of spine surgery, 2020; 14(6):924-935	trial ongoing
642	Chai et al.,	2020	J Med Internet Res. 2020 May 20; 22(5):e18537	trial ongoing
643	Cerritelli et al.,	2020	Sci Rep. 2020 Feb 21; 10(1):3214	trial ongoing
644	Centeno et al.,	2020	Stem cells international, 2020; 2020	non pertinent
645	Cederqvist et al.,	2020	Annals of the rheumatic diseases, 2020	non pertinent
646	Cedeno et al.,	2020	Pain practice, 2020; 20(1):67-	non pertinent
647	Castiglione et al.,	2020	Abdom Radiol (NY). 2020 Apr; 45(4):1162-1173	trial ongoing
648	Cassano	2020	Biological psychiatry, 2020; 87(9):128-	non pertinent

649	Carroll et al.,	2020	J Head Trauma Rehabil. 2020 Nov/Dec; 35(6):E513-E523	non pertinent
650	Cappellini et al.,	2020	New England journal of medicine, 2020; 382(13):1219-1231	trial ongoing
651	Calixtre et al.,	2020	Musculoskelet Sci Pract. 2020 Apr; 46:102108	non pertinent
652	Cai et al.,	2020	JAMA. 2020 Apr 21; 323(15):1456-1466	non pertinent
653	Cai et al.,	2020	Osteoarthritis and cartilage, 2020; 28:265-	non pertinent
654	Bushey et al.,	2020	J Telemed Telecare. 2020 Dec; 26(10):619-626	non pertinent
655	Burley et al.,	2020	Horm Behav. 2020 Sep; 125:104837	non pertinent
656	Burkhouse et al.,	2020	Neuropsychopharmacology. 2020 Feb; 45(3):561-569	non pertinent
657	Bureau et al.,	2020	Eur Radiol. 2020 Nov; 30(11):5933-5941	non pertinent
658	Brownstein et al.,	2020	Journal of clinical medicine, 2020; 9(11):1-14	non pertinent
659	Bräscher et al.,	2020	Psychosomatic medicine, 2020; 82(7):708-714	non pertinent
660	Brandt et al.,	2020	BMJ open, 2020; 10(9):e040985-	non pertinent
661	Bozzini et al.,	2020	BMC urology, 2020; 20(1):67	non pertinent
662	Boroda et al.,	2020	Brain stimulation, 2020; 13(4):1059-1068	non pertinent
663	Borg et al.,	2020	Annals of oncology, 2020; 31:1152	non pertinent
664	Boon et al.,	2020	J Cyst Fibros. 2020 Jul; 19(4):562-568	non pertinent
665	Boll et al.,	2020	Neuropharmacology. 2020 Jul; 171:108105	non pertinent
666	Bossioneault et al.,	2020	Spine J. 2020 Feb; 20(2):292-299	non pertinent
667	Boissoneault et al.,	2020	Drug Alcohol Depend. 2020 Feb 1; 207:107811	non pertinent
668	Boertien et al.,	2020	BMJ open, 2020; 10(8)	non pertinent

669	Bodily et al.,	2020	Journal of pain management, 2020; 13(2):149-166	non pertinent
670	Bismuth et al.,	2020	Neuropsychologie clinique, 2020; 50(1):5-20	non pertinent
671	Bezalel et al.,	2020	Diabetes technology & therapeutics, 2020; 22:A148-A149	non pertinent
672	Berry-Kravis et al.	2020	Journal of the American Academy of Child and Adolescent Psychiatry, 2020; 59(10):265-	non pertinent
673	Bernardi et al.,	2020	Ann Surg. 2020 Mar; 271(3):434-439	non pertinent
674	Bernard et al.,	2020	Annals of the rheumatic diseases, 2020; 79(1):794-795	non pertinent
675	Benson et al.,	2020	Brain Behav Immun. 2020 Aug; 88:294-301	non pertinent
676	Benavides et al.,	2020	Pain. 2020 Mar; 161(3):619-629	non pertinent
677	Becker et al.,	2020	Drug Alcohol Depend. 2020 Jul 1; 212:108062	non pertinent
678	Beall et al.,	2020	International journal of spine surgery, 2020; 14(2):239-253	non pertinent
679	Baraliakos et al.,	2020	Annals of the rheumatic diseases, 2020; 79(1):67-68	non pertinent
680	Balocco et al.,	2020	Regional anesthesia and pain medicine, 2020	non pertinent
681	Balderston et al.,	2020	Translational psychiatry, 2020; 10(1)	non pertinent
682	Balderston et al.,	2020	Neuropsychopharmacology. 2020 Mar; 45(4):694-702	non pertinent
683	Baettig et al.,	2020	Brain imaging and behavior, 2020; 14(6):2073-2083	non pertinent
684	Bae et al.	2020	Toxins, 2020; 12(9)	non pertinent
685	Badran et al.,	2020	Brain Stimul. 2020 Nov-Dec; 13(6):1805-1812	non pertinent

686	Bach et al.,	2020	European archives of psychiatry and clinical neuroscience, 2020	non pertinent
687	Azabou et al.,	2020	Frontiers in medicine, 2020; 7	non pertinent
688	Awada et al.,	2020	Journal for immunotherapy of cancer, 2020; 8(2)	non pertinent
689	Awad et al.,	2020	Clin Neurophysiol. 2020 May; 131(5):1059-1067	non pertinent
690	As-Sanie et al.,	2020	Fertility and sterility, 2020; 114(3):e77-	non pertinent
691	Araneda et al.,	2020	BMC neurology, 2020; 20(1):133	non pertinent
692	Andreasen et al.,	2020	Scand J Rheumatol. 2020 Jul; 49(4):312-322	non pertinent
693	Anderson et al.,	2020	Anesth Analg. 2020 Apr; 130(4):1045-1053	non pertinent
694	Ali et al.,	2020	BJS Open, 2020	non pertinent
695	Al-Hendy et al.,	2020	Fertility and sterility, 2020; 114(3); https://doi.org/10.1016/j.fertnstert.2020.08.259	non pertinent
696	Alakhdar Mohmara et al.,	2020	Sci Rep. 2020 Apr 16; 10(1):6503	non pertinent
697	Ailioaie et al.,	2020	Annals of the rheumatic diseases, 2020; 79(1):924-	non pertinent
698	Antal et al.,	2020	Brain sciences, 2020; 10(11):1-10	non pertinent
699	Andreasen et al.,	2020	Scandinavian journal of rheumatology, 2020; 49(4):312-322	non pertinent
700	Ahn et al.,	2020	J Clin Neurosci. 2020 Jul; 77:128-133	non pertinent
701	Aguilera-Lizarraga et al.,	2020	Neurogastroenterology and motility, 2020; 32	non pertinent
702	Nct – Yeh	2018	National library of Medicine. 2018	non pertinent
703	Nct – Paravlic et al.,	2018	PLoS One. 2019 Aug; 14(8):e0221089	non pertinent
704	Nct – Danneels	2018	National library of Medicine. 2018	non pertinent
705	Nct – Tahan	2018	National library of Medicine. 2018	non pertinent
706	Nct – Osmanlliu	2018	National library of Medicine. 2018	non pertinent

707	Nct – Yu	2018	National library of Medicine. 2018	non pertinent
708	Nct – Campbell-Yeo	2018	National library of Medicine. 2018	non pertinent
709	Nct – Duymaz	2018	National library of Medicine. 2018	non pertinent
710	Nct – Gaudreault	2018	National library of Medicine. 2018	non pertinent
711	Nct – Kong	2018	National library of Medicine. 2018	non pertinent
712	Nct – Lekander	2018	National library of Medicine. 2018	non pertinent
713	Nct – Shalvata Mental Health Center	2018	National library of Medicine. 2018	non pertinent
714	Nct – Berna	2018	National library of Medicine. 2018	non pertinent
715	Nct – Ismail	2018	National library of Medicine. 2018	non pertinent
716	Nct – Lekander	2018	National library of Medicine. 2018	non pertinent
717	Nct – Tang	2018	National library of Medicine. 2018	non pertinent
718	Nct – Hanlon	2018	National library of Medicine. 2018	non pertinent
719	Nct – Greenwald	2018	National library of Medicine. 2018	non pertinent
720	Nct – Schnitzer	2018	National library of Medicine. 2018	non pertinent
721	Nct – Lavigne	2018	National library of Medicine. 2018	non pertinent
722	Nct – Karris	2018	National library of Medicine. 2018	non pertinent
723	Nct – Wong	2018	National library of Medicine. 2018	non pertinent
724	Nct – Karlsten	2018	National library of Medicine. 2018	non pertinent
725	Nct – The Leeds Teaching Hospitals NHS Trust	2018	National library of Medicine. 2018	non pertinent
726	Nct – Straudi	2018	National library of Medicine. 2018	non pertinent
727	Nct – Silva	2018	National library of Medicine. 2018	non pertinent
728	Nct – Sahlem	2018	National library of Medicine. 2018	non pertinent
729	Nct – Kong	2018	National library of Medicine. 2018	non pertinent
730	Nct – Ch'ang	2018	National library of Medicine. 2018	non pertinent
731	Nct – France et al.,	2018	Contemp Clin Trials. 2018 Jun; 69:83-91	non pertinent
732	Nct – Harmer	2018	National library of Medicine. 2018	non pertinent
733	Nct – McKee et al.,	2018	Urology. 2019 Nov; 133:72-77	non pertinent
734	Nct – Chan et al.,	2018	PLoS One. 2018 Jul; 13(7):e0200987	non pertinent

735	Nasir et al.,	2018	J of the european academy of dermatology and venereology: JEADV. 2018; 32(1):145-151	non pertinent
736	Nam et al.,	2018	Drug design, development an therapy. 2018; 12:1413-1420	non pertinent
737	Naganuma et al.,	2018	Gastroenterology. 2018; 154(4):935-947	non pertinent
738	Mutti-Packer et al.,	2018	BMC Public Health. 2018; 18(1):1361	non pertinent
739	Murina et al.,	2018	European j of obstetric, gynecology and reproductive biology. 2018; 228:148-153	non pertinent
740	Morgan et al.,	2018	European j of applied physiology. 2018; 118(3):595-605	non pertinent
741	Mombelli et al.,	2018	Vaccine. 2018; 36(41):6163-6169	non pertinent
742	Moghimi et al.,	2018	J of basic and clinical physiology and pharmacology. 2018; 29(4):321-327	non pertinent
743	Mitchell et al.,	2018	JAMA internal medicine. 2018; 178(5):681-690	non pertinent
744	Miskowiak et al.,	2018	Acta psychiatrica Scandinavica. 2018; 138(4):336-347	non pertinent
745	Miki et al.,	2018	J of orthopaedic science. 2018; 23(3):483-487	non pertinent
746	Messing et al.,	2018	JAMA – j of the american medical association. 2018; 319(18):1880-1888	non pertinent
747	Merz et al.,	2018	Neuropsychopharmacology. 2018; 43(2):384-392	non pertinent
748	Merrill et al.,	2018	Arthritis & rheumatology (hoboken N.J.). 2018; 70(2):266-276	non pertinent
749	Merlin et al.,	2018	AIDS Behav. 2018; 22(8):2733-2742	non pertinent
750	Meitzer et al.,	2018	JAMA internal medicine. 2018; 178(8):1051-1057	non pertinent

751	Meijer et al.,	2018	JAMA – j of the american medical association. 2018; 320(19):2010-2019	non pertinent
752	Meier et al.,	2018	Phytomedicine. 2018; 39:85-92	non pertinent
753	Mehta et al.,	2018	Ophthalmology retinal. 2018; 2(4):268-275	non pertinent
754	Mehta et al.,	2018	J of clinical and experimental hepatology. 2018; 8(3):224-234	non pertinent
755	McGuire et al.,	2018	American j of psychiatry. 2018; 175(3):225-231	non pertinent
756	McCrae et al.,	2018	J of clinical sleep medicine. 2018; 14(9):1595-1603	non pertinent
757	Mayer-Davis et al.,	2018	The lancet. Child & adolescent health. 2018; 2(9):635-646	non pertinent
758	Mascarenhas et al.,	2018	JAMA oncology. 2018; 4(5):652-659	non pertinent
759	Maruani et al.,	2018	Trials. 2018; 19(1)	non pertinent
760	Martinsen et al.,	2018	Clin Physiol Funct Imaging. 2018; 38(3):508-516	non pertinent
761	Martinez et al.,	2018	Blood. 2018; 132	non pertinent
762	Mariano et al.,	2018	Biological psychiatry. 2018; 83(9):200-201	non pertinent
763	Mao et al.,	2018	Medical science monitor. 2018; 24:2928-2936	non pertinent
764	Malfliet et al.,	2018	Pain physician. 2018; 21(1):E13-e24	non pertinent
765	Luo et al.,	2018	Neuroimage. 2018; 171:311-322	non pertinent
766	Ludvik et al.,	2018	The lancet. Diabetes & endocrinology. 2018; 6(5):370-381	non pertinent
767	Luc-Harkey et al.,	2018	BMC musculoskeletal disorders. 2018; 19(1):258	non pertinent
768	Luc-Harkey et al.,	2018	Osteoarthritis and cartilage. 2018; 26:406-407	non pertinent
769	Lubega et al.,	2018	Scandinavian j of pain. 2018; 18(1):19-27	non pertinent
770	Lou et al.,	2018	Hong kong j of paediatrics. 2018; 23(1):116-	non pertinent

771	Lopresti et al.,	2018	J of affective disorders. 2018; 232:349-357	non pertinent
772	Lönndahl et al.,	2018	Acta dermato-venereologica. 2018; 98(3):324-328	non pertinent
773	Liou et al.,	2018	Gastroenterology. 2018; 155(4):1109-1119	non pertinent
774	Lin et al.,	2018	Diseases of the colon and rectum. 2018; 61(5):e267-e268	non pertinent
775	Lim et al.,	2018	Orthop Res. 2018; 36(3):913-920	non pertinent
776	Lichtman et al.,	2018	Circulation. 2018; 137(8):781-790	non pertinent
777	Libzon et al.,	2018	J of child neurology. 2018; 33(9):565-571	non pertinent
778	Liang et al.,	2018	Neuroinformatics. 2018; 16(3-4):403-410	non pertinent
779	Li et al.,	2018	Future oncology (london, england). 2018; 14(20):2031-2044	non pertinent
780	Li et al.,	2018	Peerj. 2018(6)	non pertinent
781	Lendaro et al.,	2018	BMJ Open. 2018; 8(7)	non pertinent
782	Leleu et al.,	2018	American j of hematology. 2018; 93(8):985-993	non pertinent
783	Lejeune et al.,	2018	Neurorehabilitation and neural repair. 2018; 32(4-5):331	non pertinent
784	Lee et al.,	2018	J of laproendoscopic & advanced surgical techniques. Part a. 2018; 28(9):1061-1067	non pertinent
785	Lee et al.,	2018	Sci Rep. 2018; 8(1):12971	non pertinent
786	Lee et al.,	2018	World neurosurgery. 2018; 109:e194-e202	non pertinent
787	Lee et al.,	2018	J of clinical oncology. 2018; 36(18):1853-1860	non pertinent
788	Lee	2018	J of thoracic oncology. 2018; 13(10):194-195	non pertinent
789	Laue et al.,	2018	Benefical microbes. 2018; 9(1):35-50	non pertinent
790	Lattermann et al.,	2018	Biomed research international. 2018	non pertinent

791	Larino-Noia et al.,	2018	Pancreatology. 2018; 18(3):280-285	non pertinent
792	Lannin et al.,	2018	Int J Stroke. 2018; 13(6):648-653	non pertinent
793	Lagueux et al.,	2018	Clin J Pain. 2018; 34(2):145-154	non pertinent
794	Ladouceur et al.,	2018	Neuroscience. 2018; 387:201-213	non pertinent
795	Ladenstein et al.,	2018	Lancet oncology. 2018; 19(12):1617-1629	non pertinent
796	Lacy et al.,	2018	American j of gastroenterology. 2018; 113(2):216-224	non pertinent
797	Labuschagne et al.,	2018	Prog Neuropsychophar macol Biol Psychiatry. 2018; 81:11-16	non pertinent
798	Laaksonen et al.,	2018	Br J Anaesth. 2018; 121(1):281-290	non pertinent
799	Kumru et al.,	2018	Brain research bulletin. 2018; 140:205-211	non pertinent
800	Kumar et al.,	2018	Osteoarthritis and cartilage. 2018; 26:18-19	non pertinent
801	Krylova et al.,	2018	Clinical EEG and neuroscience. 2018; 49(6):NP6-	non pertinent
802	Kreuder et al.,	2018	Human brain mapping. 2018	non pertinent
803	Krebs et al.,	2018	Jama. 2018; 319(9):872-882	non pertinent
804	Kothari et al.,	2018	J Prosthodont Res. 2018; 62(2):268-272	non pertinent
805	KoOdziej et al.,	2018	BMJ Open. 2018; 8(5)	non pertinent
806	Kong et al.,	2018	Neuroimage Clin. 2018; 18:325-334	non pertinent
807	Kon et al.,	2018	American j of sports medicine. 2018; 46(1):171-180	non pertinent
808	Kloek et al.,	2018	Phys Ther. 2018; 98(7):560-570	non pertinent
809	Kivitz et al.,	2018	J of rheumatology, 45(4):456-464	non pertinent
810	Kissinger et al.,	2018	The lancet. Infectious diseases. 2018; 18(11):1251-1259	non pertinent
811	Kingsbury et al.,	2018	Arthritis and rheumatology. 2018; 70:454-455	non pertinent
812	Kim et al.,	2018	Clinical orthopaedics and related research. 2018; 476(1):6-15	non pertinent

813	Kim et al.,	2018	Anesthesia and Analgesia. 2018; 127(3):635-641	non pertinent
814	Kerckhove et al.,	2018	European j of pain (London, England). 2018; 22(7):1321-1330	non pertinent
815	Kct - Cho	2018	CRIS – clinical research information service. 2018	non pertinent
816	Kaufmann et al.,	2018	Lancet (london, england). 2018; 391(10123):860-869	non pertinent
817	Kassim et al.,	2018	Saudi j of anaesthesia. 2018; 12(1):95-102	non pertinent
818	Karlapudi et al.,	2018	J of medicinal food. 2018; 21(5):511-520	non pertinent
819	Karkon Varnosfaderani et al.,	2018	J of integrative medicine. 2018; 16(2):126-131	non pertinent
820	Kang et al.,	2018	Evidence-based complementary and alternative medicine. 2018	non pertinent
821	Kandavel et al.,	2018	Human reproduction. Conference: 34th annual meeting of the european society of human reproduction and embryology. ESHRE. Barcelona, spain. 2018; 33 Suppl 1	non pertinent
822	Kallionpaa et al.,	2018	Br J Anaesth. 2018; 121(1):270-280	non pertinent
823	Kalimuddin et al.,	2018	Trials. 2018; 19(1)	non pertinent
824	Kalafatakis et al.,	2018	Proceedings of the National Academy of Sciences of the United States of America. 2018; 115(17):E4091-E4100	non pertinent
825	Kahramanoglu Akosy et al.,	2018	Medical principles and practice. 2018; 26(6):523-529	non pertinent
826	Kaczocha et al.,	2018	BMC anesthesiology. 2018; 18(1)	non pertinent
827	Jprn – Sato	2018	UMIN CTR – clinical trial. 2018	unpublished
828	Jprn – Mizobuchi	2018	UMIN CTR – clinical trial. 2018	unpublished
829	Jprn – mizobuchi	2018	UMIN CTR – clinical trial. 2018	unpublished

830	Joseph et al.,	2018	International j of therapeutic massage & bodywork. 2018; 11(4):16-22	non pertinent
831	Jones et al.,	2018	Osteoporosis international. 2018; 29(1):282-	non pertinent
832	Jin et al.,	2018	Osteoarthritis and cartilage. 2018; 26(12):1609-1618	non pertinent
833	Jin et al.,	2018	Clinical and translational science. 2018; 11(1):46-53	non pertinent
834	Jiang et al.,	2018	Restor Neurol Neurosci. 2018; 36(5): 605-620	non pertinent
835	Jia et al.,	2018	Pain Physician. 2018; 21(5): 469-481	non pertinent
836	Jensen et al.,	2018	Int J Clin Exp Hypn. 2018; 66(3):231-264	non pertinent
837	Jprn – Janssen Pharmaceutical K.K., Japan	2018	JAPIC Clinical trials information. 2018	non pertinent
838	Jablonski et al.,	2018	Clinical respiratory j. 2018; 12(1):183-192	non pertinent
839	Isrctn – McLeod	2018	ISRCTN Registry. 2018	non pertinent
840	Isrctn – Webley	2018	ISRCTN Registry. 2018	non pertinent
841	Isrctn – Thompson	2018	ISRCTN Registry. 2018	non pertinent
842	Isrctn – Le Conte	2018	ISRCTN Registry. 2018	ongoing
843	Isrctn – Berglind	2018	ISRCTN Registry. 2018	non pertinent
844	Isrctn – Ironside	2018	ISRCTN Registry. 2018	non pertinent
845	Ishaque et al.,	2018	BMC gastroenterology. 2018; 18(1)	non pertinent
846	Irct20180619040156N – Fallah	2018	IRCT Iran Registry of Clinical Trials. 2018	non pertinent
847	Irct20180427039436N – Faharani	2018	IRCT Iran Registry of Clinical Trials. 2018	non pertinent
848	Irct20171223038021N – Tajrishi	2018	IRCT Iran Registry of Clinical Trials. 2018	non pertinent
849	Irct20171219037966N – Mircheraghi	2018	IRCT Iran Registry of Clinical Trials. 2018	non pertinent
850	Irct20171129037676N – Salehinejad	2018	IRCT Iran Registry of Clinical Trials. 2018	non pertinent

851	Isrct20150721023 277N – Taghizade	2018	IRCT Iran Registry of Clinical Trials. 2018	non pertinent
852	Isrct20141218020 364N – Ayaz	2018	IRCT Iran Registry of Clinical Trials. 2018	non pertinent
853	Insogna et al.,	2018	J of bone and mineral research. 2018; 33(8):1383-1393	non pertinent
854	Ikeda et al.,	2018	Cancer science. 2018; 109(1):215-224	non pertinent
855	Ikeda et al.,	2018	Eur J Pain. 2018; 22(6):1170-1179	non pertinent
856	Ibanez et al.,	2018	European j of pediatrics. 2018; 177(12):1851-1858	non pertinent
857	Hywzn – Ribeiro	2018	Registro brasileiro de ensaios clinicos. 2018	non pertinent
858	Hyun et al.,	2018	Medicine (Baltimore). 2018; 97(39):e12595	non pertinent
859	Hunter et al.,	2018	Osteoarthritis and cartilage. 2018; 26:270-271	non pertinent
860	Hung et al.,	2018	PLoS One. 2018; 13(2):e0192043	non pertinent
861	Huhn et al.,	2018	Frontiers in Psychiatry. 2018; 9(FEB)	non pertinent
862	Huang et al.,	2018	Trials. 2018; 19(1)	non pertinent
863	Huang et al.,	2018	Clinical infectious diseases. 2018; 66(8):1222-1229	non pertinent
864	Hu et al.,	2018	BMC Med Imaging. 2018; 18(1):26	non pertinent
865	Hsu et al.,	2018	American j of hematology. 2018; 93(12):1493-1500	non pertinent
866	Howard et al.,	2018	Trials. 2018; 19(1):55	non pertinent
867	Hong et al.,	2018	International orthopaedics. 2018	non pertinent
868	Hoffman et al.,	2018	Steroids. 2018; 134:43-52	non pertinent
869	Hinsberg et al.,	2018	Coronary artery disease. 2018; 29(3):230-236	non pertinent
870	Hetland et al.,	2018	Critical care nurse. 2018; 38(1):17-23	non pertinent
871	Heo et al.,	2018	BMJ Open. 2018; 8(5)	non pertinent
872	Henssen et al.,	2018	PLoS One. 2018; 13(1):e0191774	non pertinent
873	Haugstad et al.,	2018	Scandinavian j of pain. 2018; 2(3):124-129	non pertinent

874	Hartley et al.,	2018	Lancet. 2018; 392(10164):2595-2605	non pertinent
875	Harrison et al.,	2018	Lancet. 2018; 391(10126):1173-1185	non pertinent
876	Harper et al.,	2018	BMJ Open. 2018; 8(10):e023769	non pertinent
877	Harding et al.,	2018	Clin Oncol (R Coll Radiol). 2018; 30(4):233-242	non pertinent
878	Harada-Shiba et al.,	2018	J of artherosclerosis and thrombosis. 2018; 25(5):422-429	non pertinent
879	Hansen et al.,	2018	CNS Neurosci Ther. 2018; 24(9):820-827	non pertinent
880	Hansen et al.,	2018	PLoS One. 2018; 13(8):e0201642	non pertinent
881	Hansen et al.,	2018	Regional anesthesia and pain medicine. 2018; 43(7):e54-	non pertinent
882	Hammoud et al.,	2018	Biological psychiatry. 2018; 83(9):348-	non pertinent
883	Haller et al.,	2018	Trials. 2018; 19(1)	non pertinent
884	Hafner et al.,	2018	J of neuromuscular diseases. 2018; 5:116-	non pertinent
885	Haddad et al.,	2018	PLoS One. 2018; 13(4):e0195287	non pertinent
886	Gupta et al.,	2018	Lung India. 2018; 35(1):9-15	non pertinent
887	Guilkey et al.,	2018	J of telemedicine and telecare. 2018; 24(1):44-50	non pertinent
888	Gudin et al.,	2018	Postgrad Med. 2018; 130(1):42-51	non pertinent
889	Gude et al.,	2018	Implementation science. 2018; 13(1):33	non pertinent
890	Gu et al.,	2018	Expert review of vaccines. 2018; 17(3):257-262	non pertinent
891	Grent-´t-Jong et al.,	2018	Brain. 2018; 141(8):2511-2526	non pertinent
892	Grahl et al.,	2018	Elife. 2018; 7	non pertinent
893	Goulet et al.,	2018	Contemporary clinical trials. 2018; 65:1-7	non pertinent
894	Gordon et al.,	2018	JAMA Psychiatry. 2018; 75(2):149-157	non pertinent
895	Gollub et al.,	2018	J of Pain. 2018; 19(5):515-527	non pertinent
896	Goldway et al.,	2018	Biological psychiatry. 2018; 83(9):447-	non pertinent

897	Gold et al.,	2018	J of drugs in dermatology. 2018; 17(1):66-73	non pertinent
898	Goebel et al.,	2018	Scandinavian j of pain. 2018; 18(1):29-37	non pertinent
899	Glatt et al.,	2018	Annals of the rheumatic diseases. 2018; 77(4):523-532	non pertinent
900	Gianluca et al.,	2018	Research in psychotherapy: psychopathology, process and outcome. 2018; 21:43-44	non pertinent
901	Ghasemi et al.,	2018	Anti-inflammatory & anti-allergy agents in medicinal chemistry. 2018; 17(1):57-67	non pertinent
902	Gartner et al.,	2018	Multiple sclerosis j – experimental, translational and clinical. 2018; 4(2)	non pertinent
903	Garcia-Villa et al.,	2018	Thrombosis research. 2018; 164:202-203	non pertinent
904	Gao et al.,	2018	Trials. 2018; 19(1)	non pertinent
905	Ganda et al.,	2018	Diabetes, obesity & metabolism. 2018; 20(10):2389-2398	non pertinent
906	Fox et al.,	2018	N Engl J Med. 2018; 379(9):846-855	non pertinent
907	Fox et al.,	2018	Neurology. 2018; 90(15)	non pertinent
908	Fox et al.,	2018	Gut. 2018; 67:A208-	non pertinent
909	Fortin et al.,	2018	Musculoskelet Sci Pract. 2018; 38:1-7	non pertinent
910	Fitzgibbon et al.,	2018	European j of pain (London, England). 2018; 22(7):1255-1267	non pertinent
911	Fiori et al.,	2018	Clin Exp Rheumatol 36 Suppl. 2018; 113(4):135-141	non pertinent
912	Finn et al.,	2018	Alcohol and alcoholism. 2018; 53(4):376-385	non pertinent
913	Fh5r - Freitas	2018	Registro brasileiro de ensaios clinicos. 2018	non pertinent
914	Fenster et al.,	2018	Pediatric emergency care. 2018; 34(9):607-612	non pertinent

915	Fc62c – Oliveira-Souza	2018	Registro brasileiro de ensaios clinicos. 2018	non pertinent
916	Fattahi et al.,	2018	International immunopharmacology. 2018; 54:112-117	non pertinent
917	Farrokhi et al.,	2018	Pain Physician. 2018; 21(4):383-406	non pertinent
918	Euctr - eudract_number:2018-001060-35	2018	EU clinical trial register. 2018	ongoing
919	Euctr - eudract_number:2017-002708-28	2018	EU clinical trial register. 2018	ongoing
920	Euctr - eudract_number:2017-003414-10	2018	EU clinical trial register. 2018	ongoing
921	Euctr - eudract_number:2017-003497-14	2018	EU clinical trial register. 2018	ongoing
922	Euctr - eudract_number:2017-003827-31	2018	EU clinical trial register. 2018	ongoing
923	Euctr - eudract_number:2017-003497-14	2018	EU clinical trial register. 2018	ongoing
924	Euctr - eudract_number:2017-003414-10	2018	EU clinical trial register. 2018	ongoing
925	Euctr - eudract_number:2017-004226-15	2018	EU clinical trial register. 2018	ongoing
926	Esfandiarpour et al.,	2018	Clinical j of sport medicine. 2018; 28(3):e76-e77	non pertinent
927	Erdal et al.,	2018	Drugs & aging. 2018; 1-14	non pertinent
928	Eineluoto et al.,	2018	Eur Urol Oncol. 2018; 1(3):202-207	non pertinent
929	Egerton et al.,	2018	Osteoarthritis and cartilage. 2018; 26:269-	non pertinent
930	Edelbol-Carlman et al.,	2018	Scand J Pain. 2018; 18(1):81-91	non pertinent
931	Dziewas et al.,	2018	Lancet neurology. 2018; 17(10):849-859	non pertinent
932	Dueñas-Garcia et al.,	2018	Female pelvic medicine & reconstructive surgery. 2018; 24(2):95-99	non pertinent
933	Drks – Kyung-Eun	2018	DRKS – Deutsches Register Klinischer Studien. 2018	non pertinent
934	Drks – Weber	2018	DRKS – Deutsches Register Klinischer Studien. 2018	non pertinent

935	Drks – Baumeister	2018	DRKS – Deutsches Register Klinischer Studien. 2018	non pertinent
936	Drks – Buchwitz	2018	DRKS – Deutsches Register Klinischer Studien. 2018	non pertinent
937	Dreno et al.,	2018	Lancet oncology. 2018; 19(7):916-929	non pertinent
938	Dréno et al.,	2018	British j of cancer. 2018; 118(6):777-784	non pertinent
939	Drake et al.,	2018	BMC neurology. 2018; 18(1)	non pertinent
940	Donskov et al.,	2018	Acta oncologica. 2018; 57(5):589-594	non pertinent
941	Dong et al.,	2018	Trials. 2018; 19(1):607	non pertinent
942	Domènech et al.,	2018	J of Crohn's & colitis. 2018; 12(6):687-694	non pertinent
943	Doherty-Restrepo et al.,	2018	J of allied health. 2018; 47(2):107-112	non pertinent
944	Ditre et al.,	2018	J of abnormal psychology. 2018 May	non pertinent
945	Ditre et al.,	2018	Behaviour research and therapy. 2018	non pertinent
946	Digenio et al.,	2018	Diabetes care. 2018; 41(4):807-814	non pertinent
947	Derakhshani et al.,	2018	J of sport rehabilitation. 2018; 1-30	non pertinent
948	Derakshan et al.,	2018	Anesthesiology and pain medicine. 2018; 8(5)	non pertinent
494	Dennison et al.,	2018	Clinical pharmacology in drug development. 2018; 7(8):860-870	non pertinent
950	Deng et al.,	2018	Trials. 2018; 19(1):701	non pertinent
951	DeLozier et al.,	2018	Gabi j. 2018; 7(2)	non pertinent
952	Delanian et al.,	2018	Radiotherapy and oncology. 2018; 127	non pertinent
953	Deitos et al.,	2018	Frontiers in human neuroscience. 2018; 12	non pertinent
954	Dehoratius et al.,	2018	Patient. 2018; 11(3):361-369	non pertinent

955	Degala et al.,	2018	J of oral and maxillofacial surgery. 2018; 76(9):1847-1858	non pertinent
956	DeRuysscher et al.,	2018	J of clinical oncology. 2018; 36(23):2366-2377	non pertinent
957	De Pokomandy et al.,	2018	HIV Med. 2018; 19(2):81-89	non pertinent
958	De Icco et al.,	2018	J of headache and pain. 2018; 19	non pertinent
959	De Icco et al.,	2018	Cephalalgia. 2018; 38(10):1658-1664	non pertinent
960	De Hoon et al.,	2018	Clinical pharmacology and therapeutics. 2018; 103(5):815-825	non pertinent
961	De Almeida Basano et al.,	2018	American j of tropical medicine and hygiene. 2018; 98(3):786-790	non pertinent
962	De Almeida et al.,	2018	Neuroregulation. 2018; 5(4):150-151	non pertinent
963	Davis et al.,	2018	JAMA pediatrics. 2018; 172(8):741-748	non pertinent
964	Das Nair et al.,	2018	Clinical rehabilitation. 2018; 32(6):777-789	non pertinent
965	Dai et al.,	2018	J Headache Pain. 2018; 19(1):24	non pertinent
966	Da Silva et al.,	2018	Physiology & behavior. 2018; 194:77-82	non pertinent
967	D'Angelo et al.,	2018	Investigational new drugs. 2018; 36(1):103-113	non pertinent
968	Ctri – Ramchandani	2018	Clinical trial registry India. 2018	non pertinent
969	Ctri – Tripathy	2018	Clinical trial registry India. 2018	non pertinent
970	Ctri – Bedekar	2018	Clinical trial registry India. 2018	non pertinent
971	Cruz-Correa et al.,	2018	Gastroenterology. 2018; 155(3):668-673	non pertinent
972	Cree et al.,	2018	Therapeutic advances in neurological disorders. 2018; 11	non pertinent
973	Cornelissen et al.,	2018	Eur J Anaesthesiol. 2018; 35(1):49-59	non pertinent
974	Coric et al.,	2018	J of neurosurgery. Spine. 2018; 28(3):252-261	non pertinent

975	Cooper et al.,	2018	Neuropsychopharmacology. 2018; 43(10):2046-2055	non pertinent
976	Conaghan et al.,	2018	Osteoarthritis and cartilage. 2018; 26:25-26	non pertinent
977	Cometti et al.,	2018	Peerj. 2018; (11)	non pertinent
978	Coll et al.,	2018	Cytotherapy. 2018; 20(5):33-34	non pertinent
979	Colizzi et al.,	2018	Exp Clin Psychopharmacol. 2018; 26(6):582-598	non pertinent
980	Cohen et al.,	2018	American j of clinical oncology. 2018; 41(3):230-235	non pertinent
981	Clayton et al.,	2018	J of sexual medicine. 2018; 15(1):43-51	non pertinent
982	Claessens et al.,	2018	Breast cancer research and treatment. 2018; 172(2):413-423	non pertinent
983	Chung et al.,	2018	Comput Biol Med. 2018; 101:146-152	non pertinent
984	Christopher et al.,	2018	American j on addictions. 2018; 27(4):290-291	non pertinent
985	Christiansen et al.,	2018	Physical therapy. 2018; 98(7):578-584	non pertinent
986	Chow et al.,	2018	J of clinical oncology. 2018; 36(19):1913-1921	non pertinent
987	Choi et al.,	2018	Neurosurgery. 2018; 82(3):289-297	non pertinent
988	Choi et al.,	2018	Coronary artery disease. 2018; 29(2):104-113	non pertinent
989	Choi et al.,	2018	Lasers in surgery and medicine. 2018	non pertinent
990	Choi et al.,	2018	Paediatric anaesthesia. 2018; 28(8):739-744	non pertinent
991	Cho et al.,	2018	Evidence-based complementary and alternative medicine. 2018	non pertinent
992	ChiCtr – Yuan	2018	Chinese clinical trial registry. 2018	non pertinent
993	ChiCtr – Tun	2018	Chinese clinical trial registry. 2018	non pertinent
994	ChiCtr – Zongmei	2018	Chinese clinical trial registry. 2018	non pertinent
995	ChiCtr – Chen	2018	Chinese clinical trial registry. 2018	non pertinent
996	ChiCtr - Li	2018	Chinese clinical trial registry. 2018	non pertinent
997	ChiCtr – Shuo	2018	Chinese clinical trial registry. 2018	non pertinent

998	ChiCtr – Yunliang	2018	Chinese clinical trial registry. 2018	non pertinent
999	ChiCtr – Kexin	2018	Chinese clinical trial registry. 2018	non pertinent
1000	ChiCtr – Jiancai	2018	Chinese clinical trial registry. 2018	non pertinent
1001	ChiCtr – Dan	2018	Chinese clinical trial registry. 2018	non pertinent
1002	ChiCtr - Xiang	2018	Chinese clinical trial registry. 2018	non pertinent
1003	Chi et al.,	2018	Lancet Oncol. 2018; 19(2):194-206	non pertinent
1004	Chen et al.,	2018	Clinical pharmacology in drug development. 2018; 7(8):880-888	non pertinent
1005	Chary-Valckenaere et al.,	2018	Int J Biometerol. 2018; 62(6):1003-1014	non pertinent
1006	Chang et al.,	2018	Medicine (Baltimore). 2018; 97(25):e11193	non pertinent
1007	Champion et al.,	2018	Annals of cardiac anaesthesia. 2018; 21(1):8-14	non pertinent
1008	Cats et al.,	2018	The lancet. Oncology. 2018; 19(5):616-628	non pertinent
1009	Carrizosa-Moog et al.,	2018	Canadian j of neurological sciences. 2018; 45(6):624-632	non pertinent
1010	Carmichael et al.,	2018	J of nutrition, health & aging. 2018; 22(7):837-846	non pertinent
1011	Carek et al.,	2018	Clinical j of sport medicine. Conference: 27th annual meeting of american medical society for sports medicine. AMSSM 2018. United states. 2018; 28(2):e19	non pertinent
1012	Cai et al.,	2018	Annals of the rheumatic diseases. 2018; 77:788-	non pertinent
1013	C4jxp – Engel	2018	Registro brasileiro de ensaios clinicos. 2018	non pertinent
1014	Bumbea et al.,	2018	Pain practice. Conference: 9th World Congress of the World Institute of Pain, WIP 2018 Ireland; 18:98	non pertinent

1015	Bruno et al.,	2018	Arguivos de neuro-psiguiatria. 2018; 76(4):213-218	non pertinent
1016	Brochado et al.,	2018	Braz Oral Res. 2018; 32:e50	non pertinent
1017	Brewer et al.,	2018	Fatigue: biomedicine, health and behavior. 2018; 6(3):132-140	non pertinent
1018	Brannon et al.,	2018	Psychol Health. 2018; 33(6):701-712	non pertinent
1019	Borthwick et al.,	2018	PLoS One. 2018; 13(5)	non pertinent
1020	Boonhong et al.,	2018	J of back and musculoskeletal rehabilitation. 2018; 31(3):507-513	non pertinent
1021	Boomershine et al.,	2018	Rheumatology and therapy. 2018; 5(1):271-281	non pertinent
1022	Boocock et al.,	2018	Occupational and environmental medicine. 2018; 75:A268-	non pertinent
1023	Boire et al.,	2018	J of rheumatology. 2018; 45(7):1027-1028	non pertinent
1024	Blumberger et al.,	2018	Lancet. 2018; 391(10131):1683-1692	non pertinent
1025	Blom et al.,	2018	J of clinical lipidology. 2018; 12(5):1234-1243.e1235	non pertinent
1026	Bjelovic et al.,	2018	J Gastrointest Surg. 2018; 22(11):1939-1949	non pertinent
1027	Biswas et al.,	2018	Regional anesthesia and pain medicine. 2018; 43(2):154-160	non pertinent
1028	Bischoff-Ferrari et al.,	2018	RMD open. 2018; 4(2)	non pertinent
1029	Birbara et al.,	2018	J of pain research. 2018; 11:151-164	non pertinent
1030	Bergmann et al.,	2018	Epilepsy Behav. 2018; 84:173-178	non pertinent
1031	Benitez-Camps et al.,	2018	J of hypertension. 2018; 36(8):1656-1662	non pertinent
1032	Benatar et al.,	2018	Neurology. 2018; 90(7):e565-e574	non pertinent
1033	Bembich et al.,	2018	Pediatrics. 2018; 142(3)	non pertinent
1034	Bekele et al.,	2018	J of internal medicine. 2018; 284(3):292-306	non pertinent

1035	Beissner et al.,	2018	Biol Psychiatry. 2018; 84(10):734-742	non pertinent
1036	Bayart et al.,	2018	Pediatric dermatology. 2018; 35(5):719-	non pertinent
1037	Barut et al.,	2018	Turkish j of medical sciences. 2018; 48(4):716-723	non pertinent
1038	Banerjee et al.,	2018	Annals of oncology. 2018; 29(4):917-923	non pertinent
1039	Balanyuk et al.,	2018	Acta Biomed. 2018; 89(4-s):55-63	non pertinent
1040	Back et al.,	2018	Mol Psychiatry. 2018; 23(7):1584-1589	non pertinent
1041	Auvichayapat et al.,	2018	J of pain research. 2018; 11:2301-2309	non pertinent
1042	Atthakomol et al.,	2018	BMC musculoskeletal disorders. 2018; 19(1)	non pertinent
1043	Atallah et al.,	2018	J of endourology/Endourological Society. 2018; 32:A241-	non pertinent
1044	Ascierto et al.,	2018	Lancet oncology. 2018; 19(4):510-520	non pertinent
1045	Asari et al.,	2018	Psychopharmacology (Berl). 2018; 235(9):2631-2642	non pertinent
1046	Artusi et al.,	2018	Movement disorders. 2018; 33:95-	non pertinent
1047	Armstrong-Hough et al.,	2018	J Acquir Immune Defic Syndr. 2018; 77(4):400-404	non pertinent
1048	Armio et al.,	2018	Schizophrenia bulletin. 2018; 44:391-	non pertinent
1049	Arenberger et al.,	2018	Current therapeutic research – clinical and experimental. 2018; 88:1-7	non pertinent
1050	Arbillaga-Etxarri et al.,	2018	The european respiratory j. 2018; 52(4)	non pertinent
1051	Arabi et al.,	2018	Trials. 2018; 19(1)	non pertinent
1052	Anstensrud et al.,	2018	European j of heart failure. 2018; 20:125-	non pertinent
1053	Ansermino et al.,	2018	Anesthesia and Analgesia. 2018; 127(1):23-24	non pertinent

1054	Angius et al.,	2018	Brain Stimuli. 2018; 11(1):108-117	non pertinent
1055	Andruchow et al.,	2018	Canadian j of emergency medicine. 2018; 20:32-33	non pertinent
1056	Andersson et al.,	2018	Scandinavian j of rheumatology. 2018; 47:28-	non pertinent
1057	Amraoui et al.,	2018	JAMA network open. 2018; 1(4):e181164	no oral conditions
1058	Altiparmak et al.,	2018	Saudi medical j. 2018; 39(6):579-585	non pertinent
1059	Alphs et al.,	2018	Early intervention in psychiatry. 2018; 12(1):55-65	non pertinent
1060	Allen et al.,	2018	Annals of thoracic surgery. 2018; 105(5):1344-1350	non pertinent
1061	Akeju et al.,	2018	Clin Neurophysiol. 2018; 129(1):69-78	non pertinent
1062	Aitken et al.,	2018	Osteoarthritis Cartilage. 2018; 26(7):880-887	non pertinent
1063	Aitken et al.,	2018	BMC Musculoskelet Disord. 2018; 19(1):217	non pertinent
1064	Aidelbaum et al.,	2018	J of psychopharmacology (Oxford, England). 2018; 32(5):541-551	non pertinent
1065	Ahrari et al.,	2018	Biological psychiatry. 2018; 83(9):199-200	non pertinent
1066	Ahn et al.,	2018	J of Pain. 2018	non pertinent
1067	Ahmed et al.,	2018	BMC Neurol. 2018; 18(1):191	non pertinent
1068	Ahlander et al.,	2018	J Clin Nurs. 2018; 27(5-6):1250-1261	non pertinent
1069	Agrawal et al.,	2018	Indian heart j. Conference: 70th annual conference of cardiological society of india. India. 2018; 70(Supplement 2):41-42	non pertinent
1070	Adler et al.,	2018	J of integrative medicine. 2018; 16(3):178-184	non pertinent
1071	Adams et al.,	2018	Int J Nurs Stud. 2018; 77:106-114	non pertinent

1072	Actrn – Manning	2018	Australian New Zealand Clinical Trials Registry. 2018	non pertinent
1073	Actrn – Meyrick	2018	Australian New Zealand Clinical Trials Registry. 2018	non pertinent
1074	Actrn – Scott	2018	Australian New Zealand Clinical Trials Registry. 2018	non pertinent
1075	Actrn – Sun	2018	Australian New Zealand Clinical Trials Registry. 2018	non pertinent
1076	Actrn – Stanton	2018	Australian New Zealand Clinical Trials Registry. 2018	non pertinent
1077	Actrn – Sherrington	2018	Australian New Zealand Clinical Trials Registry. 2018	non pertinent
1078	Actrn – Jones	2018	Australian New Zealand Clinical Trials Registry. 2018	non pertinent
1079	Actrn – Macdessi	2018	Australian New Zealand Clinical Trials Registry. 2018	non pertinent
1080	Actrn – Cao	2018	Australian New Zealand Clinical Trials Registry. 2018	non pertinent
1081	Actrn – Goodchild	2018	Australian New Zealand Clinical Trials Registry. 2018	non pertinent
1082	Actrn – Hides	2018	Australian New Zealand Clinical Trials Registry. 2018	non pertinent
1083	Actrn – Short	2018	Australian New Zealand Clinical Trials Registry. 2018	non pertinent
1084	Actrn – Harris	2018	Australian New Zealand Clinical Trials Registry. 2018	non pertinent
1085	Actrn – De Andrade	2018	Australian New Zealand Clinical Trials Registry. 2018	non pertinent
1086	Actrn – Kirby	2018	Australian New Zealand Clinical Trials Registry. 2018	non pertinent
1087	Nakamura et al.,	2018	Clinical orthopaedics and related research. 2018; 476(11):2169-2173	non pertinent

1088	Geusens et al.,	2018	Osteologie. 2018; 27(1):A18-	non pertinent
1089	Dittert et al.,	2018	Frontiers in behavioral neuroscience. 2018; 12	non pertinent
1090	Peng et al.,	2018	Frontiers in human neuroscience. 2018; 12	non pertinent
1091	Barron et al.,	2018	Biological psychiatry. Conference: 73rd Annual Scientific Convention and Meeting of the Society of Biological Psychiatry, SOBP 2018. United States. 2018; 83(9 Supplement 1):403	non pertinent
1092	Grimm et al.,	2018	European neuropsychopharmacology	non pertinent
1093	Cuatrecasas et al.,	2018	Endocrine connections. 2018; 7(10):1031-1039	non pertinent
1094	Ahmed et al.,	2018	BMC neurology. 2018; 18(1)	non pertinent
1095	Van Selm et al.,	2018	European j of pain (united kingdom). 2018; 22(8):1448-1456	non pertinent
1096	Naegel et al.,	2018	Neurology Conference: 70th Annual Meeting of the American Academy of Neurology, AAN 2018. United States; 90(15 Supplement 1)	non pertinent
1097	Fairchild	2018	J of the American Academy of Child and Adolescent Psychiatry. 2018; 57(12):911-913	non pertinent
1098	Kong et al.,	2018	Neuroimage: clinical. 2018; 18(325-334)	non pertinent
1099	Liao et al.,	2018	Neuroscience letters. 2018; 674(54-59)	non pertinent
1100	?	2018	Brain stimulation. 2018; 11(6):A1-A12	non pertinent
1101	Zwaans et al.,	2017	Trials. 2017; 18(1)	non pertinent
1102	Zinkus et al.,	2017	Medical science monitor. 2017; 23:3019-3025	non pertinent

1103	Zinke et al.,	2017	PLoS One. 2017; 12(10)	non pertinent
1104	Zhu et al.,	2017	Trials. 2017; 18(1)	non pertinent
1105	Zhu et al.,	2017	J of digestive diseases. 2017; 18(8):453-460	non pertinent
1106	Zhu et al.,	2017	Clinical pharmacology in drug development. 2017; 6(6):604-613	non pertinent
1107	Zhou et al.,	2017	J of endourology / Endourological Society. 2017; 31(3):278-282	non pertinent
1108	Zhou et al.,	2017	International j of surgery (London, England). 2017; 37 Suppl 1:539-545	non pertinent
1109	Zheng et al.,	2017	Zhongguo zhen jiu (Chinese acupuncture & moxibustionj). 2017; 37(8):869-873	non pertinent
1110	Zheng et al.,	2017	Osteoarthritis and cartilage. Conference: 2017 osteoarthritis research society international. OARIS world congress. United states. 2017; 25(Supplement 1):180-181	non pertinent
1111	Zheng et al.,	2017	American j of medicine. 2017; 130(10):1211-1218	non pertinent
1112	Zhao et al.,	2017	J of neurosurgical anaesthesiology. 2017; 29(2):132-139	non pertinent
1113	Zhang et al.,	2017	The lancet. Oncology. 2017; 18(3):371-383	non pertinent
1114	Zhang et al.,	2017	Clinical j of pain. 2017; 33(5):435-442	non pertinent
1115	Zhang et al.,	2017	J of neurology. 2017; 264(4):654-663	non pertinent
1116	Zhang et al.,	2017	Medicine (united states). 2017; 96(32)	non pertinent
1117	Zesiewicz et al.,	2017	Neurodegenerative disease management. 2017; 7(1):49-59	non pertinent

1118	Zerbini et al.,	2017	Brazilian j of infectious diseases. 2017; 21(1):63-70	non pertinent
1119	Zeller et al.,	2017	Circulation cardiovascular interventions. 2017; 10(9)	non pertinent
1120	Zelenetz et al.,	2017	The lancet. Oncology. 2017; 18(3):297-311	non pertinent
1121	Zarski et al.,	2017	J of sexual medicine. 2017; 14(2):238-254	non pertinent
1122	Zarei et al.,	2017	Pain medicine (united states). 2017; 18(1)	non pertinent
1123	Zakrzewska et al.,	2017	Lancet neurol. 2017; 16(4):291-300	non pertinent
1124	Yue et al.,	2017	Open access j of clinical trials. 2017; 9:41-57	non pertinent
1125	Young et al.,	2017	BMJ Open. 2017; 7(7):e017190	non pertinent
1126	Young et al.,	2017	Biol Res Nurs. 2017; 19(3):318-328	non pertinent
1127	Yoshino et al.,	2017	Cancer chemotherapy and pharmacology. 2017; 79(1):107-116	non pertinent
1128	Yin et al.,	2017	BMJ Open. 2017; 7(9):e014881	non pertinent
1129	Yilmazer et al.,	2017	Ear, nose & throat j. 2017; 96(7):250-256	non pertinent
1130	Yearwood et al.,	2017	Neuromodulation. 2017; 20(2):e61-	non pertinent
1131	Yazbeck-Karam et al.,	2017	Anesthesia and Analgesia. 2017; 124(2):438-444	non pertinent
1132	Yang et al.,	2017	Neuroimage. 2017	non pertinent
1133	Yang et al.,	2017	J of orthopaedic translation. 2017	non pertinent
1134	Yadav et al.,	2017	Saudi j of anaesthesia. 2017; 11(2):158-162	non pertinent
1135	Xue et al.,	2017	Health and quality of life outcomes. 2017; 15(1)	non pertinent
1136	Xu et al.,	2017	Archives of gynecology and obstetrics. 2017; 295(5):1167-1174	non pertinent
1137	Xu et al.,	2017	Medicine. 2017; 96(27):e7477	non pertinent
1138	Xie et al.,	2017	Medical science monitor. 2017; 23:704-711	non pertinent

1139	Wu et al.,	2017	Diabetes research and clinical practice. 2017; 127:35-43	non pertinent
1140	Wolf et al.,	2017	Gastroenterology. 2017; 153(2):410-419.e417	non pertinent
1141	Wirth et al.,	2017	Osteoarthritis Cartilage. 2017; 25(8):1313-1323	non pertinent
1142	Williams et al.,	2017	Heart (British Cardiac Society). 2017; 103:A6-A7	non pertinent
1143	Wilcox et al.,	2017	New England j of medicine. 2017; 376(4):305-317	non pertinent
1144	Wigal et al.,	2017	J of child and adolescent psychopharmacology. 2017; 27(8):690-699	non pertinent
1145	Widge et al.,	2017	Experimental neurology Part 4. 2017; 287:461-472	non pertinent
1146	Wibawa et al.,	2017	European heart j. 2017; 19:E45-	non pertinent
1147	Werumeus Buning et al.,	2017	PLoS One. 2017; 12(7)	non pertinent
1148	Weiter et al.,	2017	The lancet. Neurology. 2017; 16(8):610-619	non pertinent
1149	Weisstanner et al.,	2017	Swiss Med Wkly. 2017; 147:w14454	non pertinent
1150	Weiner et al.,	2017	Applied ergonomics. 2017; 60:22-29	non pertinent
1151	Weil et al.,	2017	Health and quality of life outcomes. 2017; 15(1)	non pertinent
1152	Weber et al.,	2017	New England j of medicine. 2017; 377(19):1824-1835	non pertinent
1153	Wassif et al.,	2017	Genetics in medicine. 2017; 19(3):297-305	non pertinent
1154	Wang et al.,	2017	Arthritis research & therapy. 2017; 19(1)	non pertinent
1155	Wang et al.,	2017	Biomed research international. 2017; 2017:6875968	non pertinent
1156	Wang et al.,	2017	Oncotarget. 2017; 8(34):57505-57515	non pertinent
1157	Wang et al.,	2017	International j of surgery (London, England); 41:44-49	non pertinent
1158	Wang et al.,	2017	Neurological research. 2017; 39(1):36-44	non pertinent

1159	Wang et al.,	2017	J of international medical research. 2017; 45(2):533-539	non pertinent
1160	Wang et al.,	2017	BMC urology. 2017; 17(1):2	non pertinent
1161	Wang et al.,	2017	Clinical j of Pain. 2017; 33(2):99-108	non pertinent
1162	Wang et al.,	2017	Medicine. 2017; 96(7):e6119	non pertinent
1163	Wan Zaidi et al.,	2017	J of the neurological sciences. 2017; 381:1119-	non pertinent
1164	Wan et al.,	2017	Supportive care in cancer. 2017; 25(2):234-	non pertinent
1165	Walter et al.,	2017	J of neural transmission. 2017; 124(10):1277-1278	non pertinent
1166	Walter et al.,	2017	European j of clinical pharmacology. 2017; 73(12):1579-1587	non pertinent
1167	Wallace et al.,	2017	Scandinavian j of pain. 2017; 17:243-251	non pertinent
1168	Vodicar et al.,	2017	Clin Spine Surg. 2017; 30(6):E707-e712	non pertinent
1169	Vo et al.,	2017	Regional anesthesia and pain medicine. 2017; 42(6)	non pertinent
1170	Viksveen et al.,	2017	Trials. 2017; 18(1)	non pertinent
1171	Verstovsek et al.,	2017	J of hematology & oncology. 2017; 10(1):1-14	non pertinent
1172	Verhees et al.,	2017	Trials. 2017; 18(1):326	non pertinent
1173	Vecchio et al.,	2017	J of headache and pain. 2017; 18(1)	non pertinent
1174	Varela-Fascinetto et al.,	2017	Pediatric transplantation. 2017; 21(1)	non pertinent
1175	Van Triest et al.,	2017	J of clinical oncology. Conference: 2017 annual meeting of the american society of clinical oncology, ASCO. United states. 2017; 35(Supplement 1)	non pertinent
1176	Van Laethem et al.,	2017	Targeted oncology. 2017; 12(1):97-109	non pertinent
1177	Van Ginckel et al.,	2017	Phys Ther. 2017; 97(5):537-549	non pertinent

1178	Van der Naalt et al.,	2017	Lancet Neurol. 2017; 16(7):532-540	non pertinent
1179	Van Den Driest et al.,	2017	BMJ Open. 2017; 7(9)	non pertinent
1180	Valentin et al.,	2017	Physiological reports. 2017; 5(5)	non pertinent
1181	Usichenko et al.,	2017	Brain stimulation. 2017; 10(2):370-371	non pertinent
1182	Umbricht et al.,	2017	Neuropsychopharmocology. 2017; 42(9):1914-1923	non pertinent
1183	Uldrick et al.,	2017	Oncologist. 2017; 22(5):505-e549	non pertinent
1184	Tutticci et al.,	2017	J of gastroenterology and hepatology (australia). 2017; 32:30-	non pertinent
1185	Turner et al.,	2017	Cancer research. Conference: 39th annual CTRC-AACR san antonio breast cancer symposium. United states. 2017; 77(4 Supplement 1)	non pertinent
1186	Tupper-Carey et al.,	2017	Singapore medical j. 2017; 58(8):481-487	non pertinent
1187	Tumuluru et al.,	2017	J of child and adolescent psychopharmacology. 2017; 28(8):708-714	non pertinent
1188	Tuinmann et al.,	2017	Psycho-oncology. 2017; 26(3):377-384	non pertinent
1189	Tschugg et al.,	2017	Eur Spine J. 2017; 26(3):857-864	non pertinent
1190	Tsay et al.,	2017	Antimicrobial agents and chemotherapy. 2017; 61(11)	non pertinent
1191	Trew et al.,	2017	Archieves of gynecology and obstetrics. 2017; 295(2):383-395	non pertinent
1192	Treanor et al.,	2017	Vaccine. 2017; 35(6):923-928	non pertinent
1193	Treadwell et al.,	2017	J of inborn errors of metabolism and screening. 2017; 5	non pertinent
1194	Tracy et al.,	2017	Psychoneuroendocrinology. 2017; 83:101-110	non pertinent
1195	Toure et al.,	2017	Clinical infectious diseases. 2017; 65(10):1711-1720	non pertinent

1196	Toprak Celenay et al.,	2017	Rheumatology international. 2017; 37(11):1799-1806	non pertinent
1197	Tombal et al.,	2017	Annals of oncology. 2017; 28:v291-v292	non pertinent
1198	Tolcher et al.,	2017	Cancer chemotherapy and pharmacology. 2017; 79(4):673-680	non pertinent
1199	Thompson et al.,	2017	Antiviral therapy. 2017; 22(3):215-223	non pertinent
1200	Thibaut et al.,	2017	Neurosci Lett. 2017; 637:207-214	non pertinent
1201	Thibaut et al.,	2017	Frontiers in neurology. 2017; 8(NOV)	non pertinent
1202	Thibaut et al.,	2017	Brain stimulation. 2017; 10(4):e40-	non pertinent
1203	Theodoraki et al.,	2017	Regiona anesthesia and pain medicine. 2017; 42(5):e10-e11	non pertinent
1204	Tesar et al.,	2017	J of the american society of nephrology: JASN. 2017; 28(11):3404-3413	non pertinent
1205	Terzi et al.,	2017	Spine (Phila Pa 1976). 2017; 42(15):1130-1138	non pertinent
1206	Teply et al.,	2017	J of clinical oncology. 2017; 35(15)	non pertinent
1207	Ten Heggeler et al.,	2017	Best practice and research: clinical gastroenterology. 2017	non pertinent
1208	Taylor et al.,	2017	Academic emergency medicine. Conference: 2017 annual meeting of the society for academic emergency medicine. SAEM 2017. United states. 2017; 24:62	non pertinent
1209	Tap et al.,	2017	The lancet. Oncology. 2017; 18(8):1089-1103	non pertinent
1210	Tang et al.,	2017	Sleep. 2017; 40:A137-	non pertinent
1211	Tan et al.,	2017	J of clinical endocrinology and metabolism. 2017; 102(7):2364-2372	non pertinent

1212	Tan et al.,	2017	PLoS One. 2017; 12(4):e0175625	non pertinent
1213	Tamm et al.,	2017	Scientific reports. 2017; 7(1):12236-	non pertinent
1214	Tamarelle et al.,	2017	Trials. 2017; 18(1)	non pertinent
1215	Tally et al.,	2017	J of orthopaedic research. 2017; 35	non pertinent
1216	Talan et al.,	2017	Annals of emergency medicine. 2017; 70(1):1-11.e19	non pertinent
1217	Taddio et al.,	2017	J of Pain. 2017; 18(9):1060-1066	non pertinent
1218	Taddio et al.,	2017	CMAJ: Canadian Medical Association j. 2017; 189(6):E227-E234	non pertinent
1219	Szucs et al.,	2017	PLoS One. 2017; 12(2)	non pertinent
1220	Swisher et al.,	2017	Lancet oncology. 2017; 18(1):75-87	non pertinent
1221	Swider et al.,	2017	PLoS One. 2017; 12(9):e0182616	non pertinent
1222	Supuran et al.,	2017	Expert opinion on drug discovery. 2017; 12(1):61-88	non pertinent
1223	Supan et al.,	2017	American j of topical medicine and hygiene. 2017; 97(2):514-525	non pertinent
1224	Sun et al.,	2017	PLoS One. 2017; 12(5)	non pertinent
1225	Sun et al.,	2017	Cancer chemotherapy and pharmacology. 2017; 79(6):1099-1107	non pertinent
1226	Sullivan et al.,	2017	J of Pain. 2017; 18(3):308-318	non pertinent
1227	Sullivan et al.,	2017	American j of psychiatry. 2017; 174(5):459-467	non pertinent
1228	Su et al.,	2017	Medicine. 2017; 96(7):e5859	non pertinent
1229	Strand et al.,	2017	RMD open. 2017; 3(1)	non pertinent
1230	Strand et al.,	2017	Arthritis care & research. 2017; 69(4):592-598	non pertinent
1231	Storheim et al.,	2017	Trials. 2017; 18(1):596	non pertinent
1232	Storheim et al.,	2017	Musculoskelet Disord. 2017; 18(1):145	non pertinent
1233	Stoltz et al.,	2017	J Pediatr Nurs. 2017; 37:91-96	non pertinent

1234	Steens et al.,	2017	Antimicrobial agents and chemotherapy. 2017; 61(7)	non pertinent
1235	Steele et al.,	2017	Sleep. 2017; 40:A97-	non pertinent
1236	Stamatiou et al.,	2017	J of surgical research. 2017; 213:147-157	non pertinent
1237	Spironelli et al.,	2017	Frontiers in human neuroscience. 2017; 11	non pertinent
1238	Spigel et al.,	2017	Lung cancer (Amsterdam, Netherlands). 2017; 113:79-84	non pertinent
1239	Spengler et al.,	2017	Biol Psychiatry. 2017; 82(12):885-894	non pertinent
1240	Sparks et al.,	2017	Manipulative Physiol Ther. 2017; 40(9):625-634	non pertinent
1241	Spahn et al.,	2017	Expert opinion on investigational drugs. 2017; 26(2):155-160	non pertinent
1242	Soulieres et al.,	2017	Lancet oncology. 2017; 18(3):323-335	non pertinent
1243	Sort et al.,	2017	BMJ Open. 2017; 7(5):e016001	non pertinent
1244	Song et al.,	2017	Vaccine. 2017; 35(2):313-320	non pertinent
1245	Song et al.,	2017	J Neurosci. 2017; 37(29):6938-6945	non pertinent
1246	Snidvongs et al.,	2017	Health technology assessment (Winchester, England). 2017; 21(74):1-130	non pertinent
1247	Sneller et al.,	2017	Science translational medicine. 2017; 9(419)	non pertinent
1248	Smith et al.,	2017	J of clinical oncology. 2017; 35(15)	non pertinent
1249	Smith et al.,	2017	Academic emergency medicine. 2017; 24(2):161-167	non pertinent
1250	Small et al.,	2017	British j of pain. 2017; 11(2):68-	non pertinent
1251	Slee et al.,	2017	Blood. 2017; 130	non pertinent
1252	Sistla et al.,	2017	J of surgical research. 2017; 212:94-100	non pertinent
1253	Singla et al.,	2017	J of pain research. 2017; 10:2413-2424	non pertinent
1254	Singh et al.,	2017	Contraception. 2017; 96(2):118-123	non pertinent

1255	Singh et al.,	2017	Clinical cancer research. 2017;	non pertinent
1256	Singh et al.,	2017	International j of chronic obstructive pulmonary disease. 2017; 12:2001-2014	non pertinent
1257	Singer et al.,	2017	Stroke: a j of cerebral circulation. 2017; 48	non pertinent
1258	Simson et al.,	2017	Trials. 2017; 18(1):184	non pertinent
1259	Simpson	2017	Dermatology and therapy. 2017; 7(2):243-248	non pertinent
1260	Simpson et al.,	2017	J of Pain. 2017; 18(1):42-53	non pertinent
1261	Short et al.,	2017	Sleep. 2017; 40:A434-A435	non pertinent
1262	Short et al.,	2017	Multiple sclerosis j. 2017; 23(3):729-730	non pertinent
1263	Shirley et al.,	2017	Drugs. 2017; 77(1):107-112	non pertinent
1264	Shin et al.,	2017	Clinical therapeutics. 2017; 39(2):268-278.e262	non pertinent
1265	Shehata et al.,	2017	Helicobacter. 2017; 22(5)	non pertinent
1266	Shaw et al.,	2017	Brain stimulation. 2017; 10(4):e60-e61	non pertinent
1267	Sharma et al.,	2017	J of the american heart association. 2017; 6(11)	non pertinent
1268	Shanahan et al.,	2017	Nicotine & tobacco research. 2017; 19(8):944-951	non pertinent
1269	Sevel et al.,	2017	J of pain. 2017; 18(4):56-	non pertinent
1270	Seo et al.,	2017	Clinical and experimental vaccine research. 2017; 6(1):38-44	non pertinent
1271	Seo et al.,	2017	Anesthesia and analgesia. 2017; 124(5):115-116	non pertinent
1272	Seo et al.,	2017	Toxins. 2017; 9(11)	non pertinent
1273	Seng et al.,	2017	Headache. 2017; 57(4):593-604	non pertinent
1274	Senan	2017	J of thoracic oncology. 2017; 12(1):42-43	non pertinent
1275	Seeger et al.,	2017	J of neural transmission. 2017; 124(10):1321-	non pertinent
1276	Seddon et al.,	2017	Lancet oncology. 2017	non pertinent

1277	Seckl et al.,	2017	J of clinical oncology. 2017; 35(12):1506-1514	non pertinent
1278	Scoville et al.,	2017	Arthritis & rheumatology. 2017; 69	non pertinent
1279	Scott et al.,	2017	Anaesthesia. 2017; 72(4):479-487	non pertinent
1280	Schmalzle et al.,	2017	Proc Natl Acad Sci U S A. 2017; 114(20):5153-5158	non pertinent
1281	Schlumberger et al.,	2017	Annals of oncology : official j of the european society for medical oncology. 2017; 28(11):2813-2819	non pertinent
1282	Schliessbach et al.,	2017	Scnadinavian j of pain. 2017; 17:107-115	non pertinent
1283	Schicho et al.,	2017	Medical science monitor. 2017; 23:796-800	non pertinent
1284	Scheffer et al.,	2017	Radiology. 2017; 282(2):585-597	non pertinent
1285	Saviola et al.,	2017	Clinical rheumatology. 2017; 1-8	non pertinent
1286	Sasai et al.,	2017	Diabetes, metabolic syndrom and obesity: targets and therapy. 2017; 10:297-309	non pertinent
1287	Sankhala et al.,	2017	J of clinical oncology. Conference: 2017 annual meeting of the american society of clincial oncology, ASCO. United states. 2017; 35(15 Supplement 1)	non pertinent
1288	Sankarasubramani an et al.,	2017	Brain connect. 2017; 7(3):182-196	non pertinent
1289	Sanfilippo et al.,	2017	Drugs in r and d. 2017; 17(1):167-175	non pertinent
1290	Sands et al.,	2017	Gastroenterology. 2017; 153(1):77-86.e76	non pertinent
1291	Sander et al.,	2017	BMC Psychiatry. 2017; 17(1):36	non pertinent
1292	Sandahl et al.,	2017	Trails. 2017; 18(1):520	non pertinent
1293	Sánchez et al., Blas et al.,	2017	International j of neuroscience. 2017; 127(5):404-411	non pertinent

1294	Salemis et al.,	2017	Drug discoveries & therapeutics. 2017; 11(1):47-50	non pertinent
1295	Salamati et al.,	2017	Iranial j of pharmaceutical research. 2017; 16(1):404-409	non pertinent
1296	Saisho et al.,	2017	Antiviral therapy. 2017; 22(4):313-323	non pertinent
1297	Ryu et al.,	2017	International j of medical sciences. 2017; 14(10):994-1001	non pertinent
1298	Ryan et al.,	2017	Neuropsychopharmacology. 2017; 42(5):1012-1023	non pertinent
1299	Russell et al.,	2017	Arteriosclerosis, thrombosis and vascular biology. 2017; 37	non pertinent
1300	Rugo et al.,	2017	Jama. 2017; 317(1):37-47	non pertinent
1301	Rovati et al.,	2017	Arthrithis & rheumatology. 2017; 69	non pertinent
1302	Rouphael et al.,	2017	Lancet (london, england). 2017; 390(10095):649-658	non pertinent
1303	Rossi et al.,	2017	Biol Psychol. 2017; 122:21-32	non pertinent
1304	Rosenberg et al.,	2017	FASEB j. 2017; 31(1)	non pertinent
1305	Rose et al.,	2017	Multiple sclerosis and related disorders. 2017; 17:32-40	non pertinent
1306	Ronzio et al.,	2017	European j of physiotherapy. 2017; 19:62-63	non pertinent
1307	Roman-Blas et al.,	2017	Arthritis & rheumatology (hoboken, N.J.). 2017; 69(1):77-85	non pertinent
1308	Rodriguez et al.,	2017	American j of emergency medicine. 2017; 35(1):164-170	non pertinent
1309	Rockey et al.,	2017	J of investigative medicine. 2017; 65(4):759-764	non pertinent
1310	Rittmeyer et al.,	2017	Lancet (london, england). 2017; 389(10066):255-265	non pertinent
1311	Ringel-Kulka et al.,	2017	American j of gastroenterology. 2017; 112(1):145-151	non pertinent

1312	Riks et al.,	2017	Cephalalgia. Conference: 18th congress of the international headache society. IHC 2017. Canada. 2017; 37(Supplement 1):54	non pertinent
1313	Riberio et al.,	2017	PLoS One. 2017; 12(11):e0187013	non pertinent
1314	Riberio et al.,	2017	BMJ Open. 2017; 7(1):e015568	non pertinent
1315	Reinhardt et al.,	2017	Circulation. 2017; 136	non pertinent
1316	Regules et al.,	2017	New England j of medicine. 2017; 376(4):330-341	non pertinent
1317	Reck et al.,	2017	Lung cancer. (Amsterdam, Netherlands). 2017; 112:181-187	non pertinent
1318	Reavis et al.,	2017	Neuropsychophar macoloy. 2017; 43:586-587	non pertinent
1319	Razani et al.,	2017	Phytotherapy research: PTR. 2017; 31(11):1731-1738	non pertinent
1320	Rauck et al.,	2017	Current medical research and opinion. 2017; 33(11):1921-1933	non pertinent
1321	Rathod et al.,	2017	International j of radiation oncology biology physics. 2017; 99(2):E226-	non pertinent
1322	Rash et al.,	2017	BMJ Open. 2017; 7(4):e014909	non pertinent
1323	Ranganathan et al.,	2017	Peritoneal dyalisis international. 2017; 37(4):420-428	non pertinent
1324	Ramo et al.,	2017	BMJ Open. 2017; 7(7)	non pertinent
1325	Ramanan et al.,	2017	New England j of medicine. 2017; 376(17):1637-1646	non pertinent
1326	Rafil et al.,	2017	J of Alzheimer's disease. 2017; 58(2):401-411	non pertinent
1327	Raeissadat et al.,	2017	Clinical rheumatology. 2017; 36(4):933-940	non pertinent
1328	Radwan et al.,	2017	BMC Cancer. 2017; 17(1):453	non pertinent
1329	Radicioni et al.,	2017	Pediatric neurology. 2017; 66:63-68	non pertinent

1330	Rabinak et al.,	2017	Biological psychiatry. 2017; 81(10):327-	non pertinent
1331	Rabinak et al.,	2017	Neuropsychopharmacology. 2017; 43:339-	non pertinent
1332	Purcell et al.,	2017	International j of lower extremity wounds. 2017; 16(3):163-172	non pertinent
1333	Puentedura et al.,	2017	J of manipulative and physiological therapeutics. 2017; 40(8):615-623	non pertinent
1334	Pudi et al.,	2017	Respiratory research. 2017; 18(1):182	non pertinent
1335	Prosenz et al.,	2017	J of clinical neuroscience. 2017; 40:195-197	non pertinent
1336	Prosenz et al.,	2017	Pain. 2017; 158(7):1264-1271	non pertinent
1337	Prologo et al.,	2017	J of vascular and interventional radiology. 2017; 28(1):24-34.e24	non pertinent
1338	Pridgen et al.,	2017	J of pain research. 2017; 10:451-460	non pertinent
1339	Price et al.,	2017	Clinical j of pain. 2017; 33(4):310-318	non pertinent
1340	Pour et al.,	2017	J of acupuncture and medical studies. 2017; 10(3):187-192	non pertinent
1341	Poply	2017	British j of pain. 2017; 11(2):10-	non pertinent
1342	Polli et al.,	2017	Eur J Phys Rehabil Med. 2017; 53(1):14-23	non pertinent
1343	Pogacnik et al.,	2017	Cells Tissues Organes. 2017; 203(4):258-266	non pertinent
1344	Pocwierz-Marciniak et al.,	2017	Health psychology report. 2017; 5(2):173-185	non pertinent
1345	Pliszka et al.,	2017	J of child and adolescent psychopharmacology. 2017; 27(6):474-482	non pertinent
1346	Pivot et al.,	2017	European j of cancer (Oxford, England: 1990). 2017; 86:82-90	non pertinent
1347	Piotrowska et al.,	2017	The lancet diabetes and endocrinology. 2017; 5(3):214-223	non pertinent

1348	Pinto-Sanchez et al.,	2017	Gastroenterology. 2017; 153(2):448-459.e448	non pertinent
1349	Pinkerton et al.,	2017	Maturitas. 2017; 100:57-63	non pertinent
1350	Piedimonte et al.,	2017	Eur J Pain. 2017; 21(5):874-885	non pertinent
1351	Phillips et al.,	2017	J of clinical oncology. 2017; 35(15)	non pertinent
1352	Petrofsky et al.,	2017	Clinical j of sport medicine. 2017; 27(4):329-337	non pertinent
1353	Petrella et al.,	2017	European j of cancer (Oxford, England : 1990). 2017; 86:115-124	non pertinent
1354	Petersen et al.,	2017	J of Alzheimer's disease. 2017; 55(2):569-574	non pertinent
1355	Peter et al.,	2017	J of ethnopharmacology. 2017; 209:288-293	non pertinent
1356	Peerdeman et al.,	2017	Eur J Pain. 2017; 21(8):1366-1377	non pertinent
1357	Pedersen et al.,	2017	European j of anaesthesiology. 2017; 34(9):602-608	non pertinent
1358	Passamonti et al.,	2017	The lancet. Oncology. 2017; 18(1):88-99	non pertinent
1359	Parra et al.,	2017	Regional anesthesia and pain medicine. 2017; 42(1):17-24	non pertinent
1360	Park et al.,	2017	European j of cancer (Oxford, England : 1990); 83:32-42	non pertinent
1361	Park et al.,	2017	J of gastroenterology and hepatology. 2017; 32(3):589-594	non pertinent
1362	Park et al.,	2017	Physiother Theroy Pract. 2017; 33(3):218-226	non pertinent
1363	Pariser et al.,	2017	J of drugs in dermatology. 2017; 16(2):127-132	non pertinent
1364	Panackal	2017	J of urology. 2017; 197(4):e1217-	non pertinent

1365	Palomba et al.,	2017	Hematological oncology. Conference: 14th international conference on malignant lymphoma palazzo die congressi. Switzerland. 2017; 35:137-138	non pertinent
1366	Pactr – Gabriel	2017	Pan African Clinical Trials Registry. 2017	ongoing
1367	Pactr – Ragab	2017	Pan African Clinical Trials Registry. 2017	non pertinent
1368	Ouellette et al.,	2017	BMJ Open. 2017; 7(8):e013080	non pertinent
1369	Ory et al.,	2017	J of neuropsychology. 2017; 11(3):378-395	non pertinent
1370	Opoka et al.,	2017	Blood. 2017; 130(24):2585-2593	non pertinent
1371	Oosterhuis et al.,	2017	J Physiother. 2017; 63(3):144-153	non pertinent
1372	Olivier et al.,	2017	American j of respiratory and critical care medicine. 2017; 195(6):814-823	non pertinent
1373	Oliveira et al.,	2017	Neuroimmudomodulation. 2017; 24(6):293-299	non pertinent
1374	Oliva et al.,	2017	The lancet. Haematology. 2017; 4(3):e127-e136	non pertinent
1375	Olff et al.,	2017	European neuropsychopharmacology. 2017; 27:527-	non pertinent
1376	Okuno	2017	Cardiovascular and interventional radiology. 2017; 40(2):89-	non pertinent
1377	Okkerse et al.,	2017	British j of clinical pharmacology. 2017; 83(4):751-763	non pertinent
1378	Ofluoglu et al.,	2017	Medicina oral, patologia oral y cirugia bucal. 2017; 22(2):e159-e166	non pertinent
1379	Nunes et al.,	2017	British j of cancer. 2017; 116(5):584-591	non pertinent
1380	Nunes et al.,	2017	Global spine j. 2017; 7(2):105-	non pertinent
1381	Ntr – van Marle	2017	Netherlands Trial Register. 2017	non pertinent

1382	Ntr – Hermans	2017	Netherlands Trial Register. 2017	non pertinent
1383	Ntr - Evers	2017	Netherlands Trial Register. 2017	non pertinent
1384	Ntr - Evers	2017	Netherlands Trial Register. 2017	non pertinent
1385	Norrby et al.,	2017	Vaccine. 2017; 35(12):1652-1661	non pertinent
1386	Noronha Oliveira et al.,	2017	Implant dentistry. 2017; 26(6):832-840	non pertinent
1387	Noorwali et al.,	2017	Proceedings of the nutrition society. 2017; 76(OCE1):E6-	non pertinent
1388	Nizamuddin et al.,	2017	Medical forum mounthly. 2017; 28(7):40-43	non pertinent
1389	Niu et al.,	2017	Mol pain. 2017; 13:1744806916683684	non pertinent
1390	Nishizuka et al.,	2017	J of orthopaedics science. 2017; 22(2):289-294	non pertinent
1391	Nishikawa et al.,	2017	International j of cancer. 2017; 140(1):188-196	non pertinent
1392	Ning et al.,	2017	J of General Internal Medicine. 2017; 32(2):495-496	non pertinent
1393	Nielsen et al.,	2017	Pain. 2017; 158(3):463-470	non pertinent
1394	Nielsen et al.,	2017	J Physiol. 2017; 595(14):4857-4873	non pertinent
1395	Nicolini et al.,	2017	Blood. 2017; 130	non pertinent
1396	Nguyen et al.,	2017	Stem cells translational medicine. 2017; 6(1):187-195	non pertinent
1397	Negrin et al.,	2017	Ann Thorac Surg. 2017; 103(5):1587-1593	non pertinent
1398	Nedeljkovix et al.,	2017	J of Pain. 2017; 18(4):70-71	non pertinent
1399	Nedeljkovic et al.,	2017	BMJ Open. 2017; 7(2):e011035	non pertinent
1400	Nct – von Horn et al.,	2017	Hum Reprod. 2017 Apr.; 32(4):832-835	non pertinent
1401	Nct – Navalesi	2017	National library of Medicine. 2017	non pertinent
1402	Nct – Hughes	2017	National library of Medicine. 2017	non pertinent
1403	Nct – Wang	2017	National library of Medicine. 2017	non pertinent
1404	Nct – Bardsley	2017	National library of Medicine. 2017	non pertinent
1405	Nct – Chan	2017	National library of Medicine. 2017	non pertinent
1406	Nct – Roy	2017	National library of Medicine. 2017	non pertinent
1407	Nct – Streckmann	2017	National library of Medicine. 2017	non pertinent

1408	Nct – Winson	2017	National library of Medicine. 2017	non pertinent
1409	Nct – Vissers	2017	National library of Medicine. 2017	non pertinent
1410	Nct – Chong	2017	National library of Medicine. 2017	non pertinent
1411	Nct – Peolsson et al.,	2017	BMJ Open. 2019 Feb., 9(2):e027387	non pertinent
1412	Nct – Dong et al.,	2017	Trials. 2018 Nov.; 19(1):607	non pertinent
1413	Nct – Senthna	2017	National library of Medicine. 2017	non pertinent
1414	Nct – Shawki	2017	National library of Medicine. 2017	non pertinent
1415	Nct – Gohary	2017	National library of Medicine. 2017	non pertinent
1416	Nct – Hurtado	2017	National library of Medicine. 2017	non pertinent
1417	Nct – Iancu	2017	National library of Medicine. 2017	non pertinent
1418	Nct – Kulu et al.,	2017	Trials. 2018 Jul; 19(1):377	non pertinent
1419	Nct – Cha	2017	National library of Medicine. 2017	non pertinent
1420	Nct – Guillot	2017	National library of Medicine. 2017	non pertinent
1421	Nct – Yesavage	2017	National library of Medicine. 2017	non pertinent
1422	Nct – Ganapathy	2017	National library of Medicine. 2017	non pertinent
1423	Nct – Somri	2017	National library of Medicine. 2017	non pertinent
1424	Nct – Moberg et al.,	2017	J Med Internet Res. 2019 Jun; 21(6):e12556	non pertinent
1425	Nct - Benoit	2017	National library of Medicine. 2017	non pertinent
1426	Nct – Gretebeck	2017	National library of Medicine. 2017	non pertinent
1427	Nct – Wagner	2017	National library of Medicine. 2017	non pertinent
1428	Nct – Rimmer et al.,	2017	Contemp Clin Trials. 2018 Aug; 71:186-193	non pertinent
1429	Nct – Morris	2017	National library of Medicine. 2017	non pertinent
1430	Nct – Kim et al.,	2017	Trials. 2019 Jan; 20(1):56	non pertinent
1431	Nct – Prüss-Volz	2017	National library of Medicine. 2017	non pertinent
1432	Nct – Peolsson et al.,	2017	BMC Musculoskelet Disord. 2017 Dez; 18(1):524	non pertinent
1433	Nct – Deau	2017	National library of Medicine. 2017	non pertinent
1434	Nct – Mescouto	2017	National library of Medicine. 2017	non pertinent
1435	Nct – Kouvelas	2017	National library of Medicine. 2017	non pertinent
1436	Nct – Gómez-Pérez et al.,	2017	Trials. 2018 Nov; 19(1):660	non pertinent
1437	Nct – Ashar	2017	National library of Medicine. 2017	non pertinent

1438	Nct – Wang	2017	National library of Medicine. 2017	non pertinent
1439	Nct – Fitzgerald	2017	National library of Medicine. 2017	non pertinent
1440	Nct – Schwartz	2017	National library of Medicine. 2017	non pertinent
1441	Nct – Buhrman	2017	National library of Medicine. 2017	non pertinent
1442	Nct – Taipei Veterans General Hospital, Taiwan	2017	National library of Medicine. 2017	non pertinent
1443	Nct – Pacella	2017	National library of Medicine. 2017	non pertinent
1444	Nayak et al.,	2017	Antimicrobial agents and chemotherapy. 2017; 61(8)	non pertinent
1445	Nava et al.,	2017	Pancreatology. 2017; 17(4):34-	non pertinent
1446	Musters et al.,	2017	Annals of surgery. 2017; 265(6):1074-1081	non pertinent
1447	Murata-Ooiwa et al.,	2017	J of arthroplasty. 2017; 32(10):3024-3028	non pertinent
1448	Munjal et al.,	2017	J of headache and pain. 2017; 18(1)	non pertinent
1449	Mukaihara et al.,	2017	J of anesthesia. 2017; 31(6):829-836	non pertinent
1450	Mueller et al.,	2017	Transl Psychiatry. 2017; 7(4):e1084	non pertinent
1451	Motzer et al.,	2017	J of clinical oncology. 2017; 35(35):3916-3923	non pertinent
1452	Moskal et al.,	2017	Alcoholism: clinical and experimental research. 2017; 41:225A-	non pertinent
1453	Morton et al.,	2017	Surgery for obesity and related diseases. 2017; 13(10):63-	non pertinent
1454	Morte et al.,	2017	American j of reproductive immunology. 2017; 78(3)	non pertinent
1455	Morris et al.,	2017	PLoS One. 2017; 12(2)	non pertinent
1456	Morin et al.,	2017	American j of obstetrics and gynecology. 2017	non pertinent
1457	Moreno et al.,	2017	Arthritis & rheumatology. 2017; 69	non pertinent
1458	Morawiec et al.,	2017	BMJ Open. 2017; 7(7)	non pertinent
1459	Moraska et al.,	2017	Am J Phys Med Rehabil. 2017; 96(9):639-645	non pertinent
1460	Morabito et al.,	2017	Lung cancer (Amsterdam, Netherlands). 2017; 108:15-21	non pertinent

1461	Moosmayer et al.,	2017	BMC musculoskeletal disorders. 2017; 18(1):138	non pertinent
1462	Moosa et al.,	2017	National j of physiology, pharmacy and pharmacology. 2017; 7(8):839-843	non pertinent
1463	Moon et al.,	2017	Pain Physician. 2017; 20(2):27-35	non pertinent
1464	Monteith et al.,	2017	Frontiers in pharmacology. 2017; 8(OCT)	non pertinent
1465	Monreal-Carrillo et al.,	2017	Support Care Cancer. 2017; 25(10):3143-3149	non pertinent
1466	Monfort et al.,	2017	Med Clin (Barc). 2017; 148(12):539-547	non pertinent
1467	Moeller et al.,	2017	Cephalalgia. Conference: 18th congress of the international headache society. IHC 2017. Canada; 37(1 Supplement):70-71	non pertinent
1468	Modak et al.,	2017	Annals of cardiac anaesthesia. 2017; 20(2):163-168	non pertinent
1469	Mizrachi et al.,	2017	Human reproduction (Oxford, England). 2017; 32(6):1202-1207	non pertinent
1470	Miyamoto et al.,	2017	BMC Cancer. 2017; 17(1)	non pertinent
1471	Miskowiak et al.,	2017	J Psychopharmacol. 2017; 31(9):1215-1224	non pertinent
1472	Minamide et al.,	2017	J Orthop Sci. 2017; 22(3):377-383	non pertinent
1473	Miller	2017	Neurodegenerative disease management. 2017; 7(1):9-29	non pertinent
1474	Miklowitz et al.,	2017	Bipolar disorders. 2017; 19:46-47	non pertinent
1475	Meyer et al.,	2017	The lancet. Gastroenterology & hepatology. 2017; 2(8):565-575	non pertinent
1476	Meuret et al.,	2017	Depression and anxiety. 2017; 34(12):1096-1105	non pertinent
1477	Metz et al.,	2017	Neuropsychopharmacology. 2017; 42(9):1825-1832	non pertinent

1478	Menne et al.,	2017	Nephrology, dialysis, transplantation. 2017; 32(2):307-315	non pertinent
1479	Melnick et al.,	2017	J of general internal medicine. Conference: 40th annual meeting of the society of general internal medicine, SGIM 2017. United states; 32(2 Supplement 1):504	non pertinent
1480	Mell et al.,	2017	International j of radiation oncology biology physics. 2017; 97(3):536-545	non pertinent
1481	Mekel-Bobrov et al.,	2017	Neuromodulation. 2017; 20(2):e54-	non pertinent
1482	Mehan et al.,	2017	Clinical rhinology. 2017; 10(3):113-119	non pertinent
1483	Meesters et al.,	2017	Archives of disease in childhood: fetal and neonatal edition. 2017; 102(2):F167-F169	non pertinent
1484	McLaughlin et al.,	2017	Brain stimulation. 2017; 10(4):e37-	non pertinent
1485	McKay et al.,	2017	Clinical cancer research. 2017; 23(4):935-945	non pertinent
1486	McEvoy et al.,	2017	Contemp Clin Trials. 2017; 60:34-41	non pertinent
1487	McDonald et al.,	2017	JAMA oncology. 2017; 3(7):953-959	non pertinent
1488	McDermott et al.,	2017	Cancer research. Confernece: 39th annual CTRC-AACR san antonio breast cancer symposium. United states. 2017; 77(4 Supplement 1)	non pertinent
1489	Mazzocca et al.,	2017	Arthroscopy. 2017; 33(6):1138-1148	non pertinent
1490	Mayorga et al.,	2017	Scandinavian j of pain. 2017; 17:134-143	non pertinent
1491	Mayer et al.,	2017	PLoS medicine. 2017; 14(11):e1002435	non pertinent
1492	May et al.,	2017	Sci Rep. 2017; 7:44083	non pertinent

1493	Matsuoka et al.,	2017	BMC Cancer. 2017; 17(1):674	non pertinent
1494	Mathew et al.,	2017	Neuropsychopharmacology. 2017; 42(13):2567-2574	non pertinent
1495	Matangila et al.,	2017	International j of antimicrobial agents. 2017; 49(3):339-347	non pertinent
1496	Masuda et al.,	2017	Japanese j of clinical oncology. 2017; 47(5):385-392	non pertinent
1497	Mason et al.,	2017	BMJ Open. 2017; 7(1):e014324	non pertinent
1498	Mascarenhas et al.,	2017	Leukemia research. 2017; 53:13-19	non pertinent
1499	Maruyama et al.,	2017	Canadian j of gastroenterology and hepatology. 2017	non pertinent
1500	Martin et al.,	2017	The lancet. Oncology. 2017; 18(12):1688-1700	non pertinent
1501	Marth et al.,	2017	European j of cancer. 2017; 70:111-121	non pertinent
1502	Mariano et al.,	2017	Biological psychiatry. 2017; 81(10):263-	non pertinent
1503	Marbury et al.,	2017	Drug design, development and therapy. 2017; 11:373-382	non pertinent
1504	Maranon et al.,	2017	Surgical endoscopy and other interventional techniques. 2017; 31(2):98-	non pertinent
1505	Maniquis-Smigel et al.,	2017	Anesthesiology and pain medicine. 2017; 7(1)	non pertinent
1506	Manco-Johnson et al.,	2017	J Thromb Haemost. 2017; 15(11):2115-2124	non pertinent
1507	Mak et al.,	2017	BMJ Open. 2017; 7(7):e015191	non pertinent
1508	Maitre et al.,	2017	Arch Dis Child Fetal Neonatal Ed. 2017; 102(5):F428-f433	non pertinent
1509	Maher et al.,	2017	J of maternal-fetal & neonatal medicine. 2017; 30(18):2179-2184	non pertinent
1510	Maeda et al.,	2017	Brain. 2017; 140(4):914-927	non pertinent
1511	Madhi et al.,	2017	Expert review of vaccines. 2017; 16(6):641-656	non pertinent
1512	Madan et al.,	2017	Vaccine. 2017; 35(10):1431-1439	non pertinent

1513	Macrinici et al.,	2017	Regional anesthesia and pain medicine. 2017; 42(1):10-16	non pertinent
1514	Macintyre et al.,	2017	BMC medicine. 2017; 15(1)	non pertinent
1515	Machado et al.,	2017	J of neurosurgery. Conference: 85th american association of neurological surgeons annual scientific meeting, AANS 2017. United states; 126(4):A1430-A1431	non pertinent
1516	Ma et al.,	2017	Ear, nose & throat j. 2017; 96(4-5):E21	non pertinent
1517	Luz-Santos et al.,	2017	Trials. 2017; 18(1):609	non pertinent
1518	Luo et al.,	2017	Paediatric drugs. 2017; 19(5):479-486	non pertinent
1519	Luo et al.,	2017	Neuroimage. 2017; 162:127-137	non pertinent
1520	Lundkvist et al.,	2017	Diabetes, obesity & metabolism. 2017; 19(1):49-60	non pertinent
1521	Lu et al.,	2017	Arthroscopy – j of arthroscopic and related surgery. 2017; 33(10):e65-	non pertinent
1522	Lota et al.,	2017	European j of heart failure. Conference: heart failure 2017 and the 4th world congress on acute heart failure. France; 19:258	non pertinent
1523	Lorenz et al.,	2017	Trails. 2017; 18(1)	non pertinent
1524	Lopes de Jesus et al.,	2017	PLoS One. 2017; 12(7):e0179185	non pertinent
1525	Löffler et al.,	2017	European j of pain (London, England). 2017; 21(10):1623-1631	non pertinent
1526	Liu et al.,	2017	Hum Brain Mapp. 2017; 38(9):4386-4397	non pertinent
1527	Liu et al.,	2017	Human brain mapping. 2017; 38(10):5250-5259	non pertinent
1528	Lipsman et al.,	2017	Lancet Psychiatry. 2017; 4(4):285-294	non pertinent
1529	Link et al.,	2017	Lancet infecious diseases. 2017; 17(8):854-866	non pertinent
1530	Ling et al.,	2017	Nutrients. 2017; 9(10)	non pertinent

1531	Lima et al.,	2017	European j of pain (united kingdom). 2017; 21(1):159-165	non pertinent
1532	Liljencrantz et al.,	2017	European j of pain (united kingdom). 2017	non pertinent
1533	Ligsay et al.,	2017	J of neurodevelopmental disorders. 2017; 9(1)	non pertinent
1534	Liebschutz et al.,	2017	JAMA internal medicine. 2017; 177(9):1265-1272	non pertinent
1535	Liang et al.,	2017	Oncotarget. 2017; 8(31):51869-51877	non pertinent
1536	Li-Kim-Moy et al.,	2017	J of paediatrics and child health. 2017; 53(1):47-54	non pertinent
1537	Li et al.,	2017	Cephalalgia. 2017; 37(2):161-176	non pertinent
1538	Li et al.,	2017	Neurologist. 2017; 22(5):157-165	non pertinent
1539	Li et al.,	2017	BMC rheumatology. 2017; 1(1)	non pertinent
1540	Li	2017	Pharmacology & therapeutics. 2017	non pertinent
1541	Levinger et al.,	2017	Physical therapy in sport. 2017; 27:29-37	non pertinent
1542	Leung et al.,	2017	Osteoarthritis and cartilage. 2017; 25:172-173	non pertinent
1543	Letafatkar et al.,	2017	J of back and musculoskeletal rehabilitation. 2017; 30(4):767-778	non pertinent
1544	Lelic et al.,	2017	British j of clinical pharmacology. 2017; 83(4):764-776	non pertinent
1545	Lelic et al.,	2017	Neuropharmacology. 2017; 123:455-464	non pertinent
1546	Lefevre et al.,	2017	Regulatory toxicology and pharmacology : RTP. 2017; 83:54-65	non pertinent
1547	Lee et al.,	2017	Clin Cancer Res. 2017; 23(16):4556-4568	non pertinent
1548	Lee et al.,	2017	BMC Anesthesiol. 2017; 17(1):136	non pertinent
1549	Lee et al.,	2017	World neurosurgery. 2017	non pertinent

1550	Lee et al.,	2017	Proceedings of the National Academy of Sciences of the United States of America. 2017; 114(45):E9445-E9454	non pertinent
1551	Lazaridou et al.,	2017	Clin J Pain. 2017; 33(3):215-221	non pertinent
1552	Layton et al.,	2017	Cardiovascular and interventional radiology. 2017; 40(2):95-97	non pertinent
1553	Larsen et al.,	2017	Wound repair and regeneration. 2017; 25(6):984-993	non pertinent
1554	Langhans et al.,	2017	Ann Surg. 2017; 266(1):29-35	non pertinent
1555	Laneri et al.,	2017	Hum Brain Mapp. 2017; 38(8):4034-4046	non pertinent
1556	Lane et al.,	2017	Acta dermato-venereologica. 2017; 97(7):882-	non pertinent
1557	Lamusuo et al.,	2017	European j of pain (united kingdom). 2017; 21(9):1505-1515	non pertinent
1558	Lagerkranser et al.,	2017	Scandinavian j of pain. 2017	non pertinent
1559	Labat et al.,	2017	BJOG. 2017; 124(2):251-260	non pertinent
1560	Kurowski et al.,	2017	J of head trauma rehabilitation. 2017; 32(2):79-89	non pertinent
1561	Kurganova et al.,	2017	Zhurnal nevrologii i psihiatrii imeni s. 2017; 117(10):49-54	non pertinent
1562	Kundu et al.,	2017	Neuropsychopharmacology. 2017; 43:420-421	non pertinent
1563	Kunas et al.,	2017	European neuropsychopharmacology. 2017; 27:1005-1006	non pertinent
1564	Kumar et al.,	2017	Clnical therapeutics. 2017; 39(1):150-158	non pertinent
1565	Kristensen et al.,	2017	Nutrition & metabolism. 2017; 14(1):1-11	non pertinent
1566	Krebs et al.,	2017	Contemporary clinical trials. 2017; 62:130-139	non pertinent
1567	Krafft et al.,	2017	Eur J Pain. 2017; 21(9):1538-1549	non pertinent

1568	Kovacic et al.	2017	The lancet. Gastroenterology & hepatology. 2017; 2(10):727-737	non pertinent
1569	Kotrashetti et al.,	2017	International j of oral and maxillofacial surgery. 2017; 46(1):54-58	non pertinent
1570	Kong et al.,	2017	Oncotarget. 2017; 8(4):7003-7013	non pertinent
1571	Komulainen et al.,	2017	Psychiatry Res Neuroimaging; 263:61-69	non pertinent
1572	Kolk et al.,	2017	J Shoulder Elbow Surg. 2017; 26(8):1407-1415	non pertinent
1573	Kohútová et al.,	2017	Physiological research. 2017; 66(6):1041-1047	non pertinent
1574	Köhler-Forsberg et al.,	2017	Depression and anxiety. 2017; 34(3):281-290	non pertinent
1575	Kohl et al.,	2017	BMC dermatology. 2017; 17(1):12	non pertinent
1576	Koga et al.,	2017	Cancer science. 2017; 108(12):2326-2332	non pertinent
1577	Koch et al.,	2017	J Psychiatry Neurosci. 2017; 42(5):331-342	non pertinent
1578	Koban et al.,	2017	J Neurosci. 2017; 37(13):3621-3631	non pertinent
1579	Kline et al.,	2017	Emergency medicine j. 2017; 34(2):82-88	non pertinent
1580	Kives et al.,	2017	J of obstetrics and gynaecology canada. 2017; 39(2):82-90	non pertinent
1581	Kim et al.,	2017	Neuroreport. 2017; 28(14):896-902	non pertinent
1582	Kim et al.,	2017	PLoS One. 2017; 12(3):e0173026	non pertinent
1583	Kim et al.,	2017	Medicine. 2017; 96(7):e6107	non pertinent
1584	Kim et al.,	2017	Physical therapy in sport. 2017; 24:67-73	non pertinent
1585	Kim et al.,	2017	PLoS One. 2017; 12(1)	non pertinent
1586	Kim et al.,	2017	Diabetes, obesity & metabolism. 2017; 19(12):1762-1772	non pertinent
1587	Kiessling et al.,	2017	Science translational medicine. 2017; 9(414)	non pertinent

1588	Khalili et al.,	2017	J of isfahan medical school. 2017; 34(410):1481-1486	non pertinent
1589	Keyan et al.,	2017	Psychoneuroendocrinology. 2017; 77:150-157	non pertinent
1590	Kerherve et al.,	2017	Frontiers in physiology. 2017; 8(APR)	non pertinent
1591	Kerckhove et al.,	2017	BMJ Open. 2017; 7(7):e015380	non pertinent
1592	Kells et al.,	2017	Veterinary anaesthesia and analgesia. 2017; 44(5):1156-1165	non pertinent
1593	Kelleners-Smeets et al.,	2017	J of investigative dermatology. 2017; 137(3):539-540	non pertinent
1594	Keles et al.,	2017	J of back and musculoskeletal rehabilitation. 2017; 30(3):543-550	non pertinent
1595	Kay et al.,	2017	Human psychopharmacology. 32(4)	non pertinent
1596	Kawalder et al.,	2017	British j of haematology. Conference: 57th annual scientific meetin of the british society for haematology. United kingdom. 2017; 176:18	non pertinent
1597	Kaufman	2017	Expert opinion on pharmacotherapy. 2017; 18(4):433-444	non pertinent
1598	Katz et al.,	2017	Acad Emerg Med. 2017; 24(8):968-982	non pertinent
1599	Kato et al.,	2017	Pancreas. 2017; 46(7):880-886	non pertinent
1600	Karaduman et al.,	2017	Pain research & management. 2017:2454267	non pertinent
1601	Karabayirli et al.,	2017	European archives of oto-laryngology. 2017; 274(1):239-245	non pertinent
1602	Kapoor et al.,	2017	J of anaesthesiology, clinical pharmacology. 2017; 33(2):164-171	non pertinent
1603	Kamenov et al.,	2017	Maturitas. 2017; 99:20-26	non pertinent

1604	Kalita et al.,	2017	Neurol India. 2017; 65(3):506-511	non pertinent
1605	Kalfon et al.,	2017	Intesive Care Med. 2017; 43(12):1829-1840	non pertinent
1606	Jung et al.,	2017	American j of orthodontics and dentofacial orthopedics. 2017; 152(5):582-591	non pertinent
1607	Juliato et al.,	2017	Climacteric. 2017; 20(1):62-66	non pertinent
1608	Juel et al.,	2017	Pancreas. 2017; 46(2):170-176	non pertinent
1609	Jprn, Umin - Sunagawa	2017	UMIN Clinical Trials Registry. 2017	non pertinent
1610	Jprn, Umin - Uehara	2017	UMIN Clinical Trials Registry. 2017	non pertinent
1611	Jprn, Umin - Mizuno	2017	UMIN Clinical Trials Registry. 2017	non pertinent
1612	Johansson et al.,	2017	British j of clinical pharmacology. 2017; 83(9):2008-2014	non pertinent
1613	Johannsen et al.,	2017	Psycho-oncology. 2017; 26(12):2208-2214	non pertinent
1614	Jinno et al.,	2017	Nutrients. 2017; 9(3)	non pertinent
1615	Jeon et al.,	2017	The lancet gastroenterology and hepatology. 2017; 2(11):793-804	non pertinent
1616	Jensen et al.,	2017	BMC complementary and alternative medicine. 2017; 17	non pertinent
1617	Jeffcoate et al.,	2017	Health technology assessment. 2017; 21(34)	non pertinent
1618	Jayne et al.,	2017	J of the american science of nephrology : JASN. 2017; 28(9):2756-2767	non pertinent
1619	Jawad et al.,	2017	BMC oral health. 2017; 17(1):53	non pertinent
1620	Janvier et al.,	2017	J of nuclear cardiology. 2017; 24(1):34-40	non pertinent
1621	Jang et al.,	2017	BMC Anesthesiol. 2017; 17(1):73	non pertinent
1622	Jamshidi et al.,	2017	Arthritis research & therapy. 2017; 19(1)	non pertinent
1623	Jahangard et al.,	2017	Psychiatry research. 2017; 251:182-191	non pertinent

1624	Jacob et al.,	2017	American j of gastroenterology. 2017; 112: 663-664	non pertinent
1625	Isrctn – Small	2017	ISRCTN Registry. 2017	non pertinent
1626	Isrctn – Bibby	2017	ISRCTN Registry. 2017	non pertinent
1627	Isrctn – de Bono	2017	ISRCTN Registry. 2017	non pertinent
1628	Isrctn – Fennell	2017	ISRCTN Registry. 2017	non pertinent
1629	Isrctn – Gusi	2017	ISRCTN Registry. 2017	non pertinent
1630	Isrctn – Little	2017	ISRCTN Registry. 2017	non pertinent
1631	Isrctn – Thew	2017	ISRCTN Registry. 2017	non pertinent
1632	Isrctn – Søgaard	2017	ISRCTN Registry. 2017	non pertinent
1633	Isrctn – Davies	2017	ISRCTN Registry. 2017	non pertinent
1634	Isrctn – Ritter	2017	ISRCTN Registry. 2017	non pertinent
1635	Irct20091215002861N - Masoudi	2017	IRCT – Iranian Registry of Clinical Trials. 2017	non pertinent
1636	Irct2017022327728N – Mahmoodi	2017	IRCT – Iranian Registry of Clinical Trials. 2017	non pertinent
1637	Irct2015121425523N - Dadashi	2017	IRCT – Iranian Registry of Clinical Trials. 2017	non pertinent
1638	Irct201606091959N - Malakouti	2017	IRCT – Iranian Registry of Clinical Trials. 2017	non pertinent
1639	Inusa et al.,	2017	Blood. 2017; 130	non pertinent
1640	Imam	2017	J of nephrology. 2017; 30(6):803-809	non pertinent
1641	Ide et al.,	2017	Orthopaedic j of sports medicine. 2017; 5(9)	non pertinent
1642	Ide et al.,	2017	Brain stimulation. 2017; 10(2):522-523	non pertinent
1643	Ibrahimi et al.,	2017	Cephalalgia. 2017; 37(1):94-98	non pertinent
1644	Hwang et al.,	2017	World J Gastroenterol. 2017; 23(25):4615-4623	non pertinent
1645	Husain et al.,	2017	J of psychopharmacology (Oxford, England). 2017; 31(9):1166-1175	non pertinent

1646	Humaidan et al.,	2017	Human reproduction (Oxford, England). 2017; 32(3):544-555	non pertinent
1647	Hull et al.,	2017	Current alzheimer research. 2017; 14(7):696-708	non pertinent
1648	Huffman et al.,	2017	Clin J Pain. 2017; 33(7):569-578	non pertinent
1649	Huddart et al.,	2017	International j of radiation oncology biology physics. 2017; 99(2):118-119	non pertinent
1650	Hu et al.,	2017	Saudi j of gastroenterology. 2017; 23(5):275-280	non pertinent
1651	Hsu et al.,	2017	PLoS One. 2017; 12(9)	non pertinent
1652	Hovingh et al.,	2017	J of clinical lipidology. 2017; 11(6):1448-1457	non pertinent
1653	Hovdenak Jakobsen et al.,	2017	Acta Oncol. 2017; 56(2):307-313	non pertinent
1654	Hoppe et al.,	2017	British j of haematology. 2017; 177(4):620-629	non pertinent
1655	Hopewell et al.,	2017	BMJ Open. 2017; 7(7):e018004	non pertinent
1656	Hollinger et al.,	2017	BMJ Open. 2017; 7(7):e015783	non pertinent
1657	Hofmann et al.,	2017	Transfusion. 2017; 57:99A-	non pertinent
1658	Hochhaus et al.,	2017	New England j of medicine. 2017; 376(10):917-927	non pertinent
1659	Ho et al.,	2017	Skeletal radiology. Conference: 40th annual scientific meeting of the society of skeletal radiology. United states. Conference start: 20170319. Conference end: 20170322. 2017; 45(3):431	non pertinent
1660	Hirsch et al.,	2017	Clinical lung cancer. 2017; 18(1):43-49	non pertinent
1661	Hidalgo-Tenorio et al.,	2017	AIDS research and therapy. 2017; 14(1)	non pertinent
1662	Herrmann et al.,	2017	Brain stimuli. 2017; 10(2):291-297	non pertinent
1663	Hellerstein et al.,	2017	J of affective disorders. 2017; 210:258-264	non pertinent

1664	Heller et al.,	2017	Health technology assessement. 2017; 21(20):1-277	non pertinent
1665	Heimburger et al.,	2017	European j of nuclear medicine and molecular imaging: 1-9	non pertinent
1666	Heidenreich et al.,	2017	European urology. 2017; 71(4):534-542	non pertinent
1667	Heeren et al.,	2017	Soc Cogn Affect neurosci. 2017; 12(2):251-260	non pertinent
1668	Hebestreit et al.,	2017	J of headache and pain. 2017; 18(1):116	non pertinent
1669	Hebestreit et al.,	2017	PLoS One. 2017; 12(10):e0184406	non pertinent
1670	Hebestreit et al.,	2017	Cephalalgia. 2017; 37(1):21-	non pertinent
1671	Hebestreit et al.,	2017	J Headache Pain. 2017; 18(1):116	non pertinent
1672	Heapy et al.,	2017	JAMA internal medicine. 2017; 177(6):765-773	non pertinent
1673	He et al.,	2017	Biomedical research (india). 2017; 28(4):1890-1893	non pertinent
1674	Harrington et al.,	2017	Lancet oncology. 2017; 18(8):1104-1115	non pertinent
1675	Hardy-Fairbanks et al.,	2017	J of obstetrics and gynaecology. 2017; 37(8):1009-1014	non pertinent
1676	Handen et al.,	2017	J of the American Academy of Child and Adolescent Psychiatry. 2017; 849-856.e846	non pertinent
1677	Han et al.,	2017	BMC Cancer. 2017; 17(1)	non pertinent
1678	Han et al.,	2017	J Clin Anesth. 2017; 37:86-91	non pertinent
1679	Han et al.,	2017	Cancer research and treatment. 2017; 49(1):10-19	non pertinent
1680	Hamiditabar et al.,	2017	Clinical nuclear medicine. 2017	non pertinent
1681	Halawi et al.,	2017	The lancet gastroenterology and hepatology. 2017; 2(12):842-843	non pertinent
1682	Hageman et al.,	2017	Archives of trauma research. 2017; 6(1)	non pertinent
1683	Hachach-Haram et al.,	2017	International wound j. 2017; 14(1):241-249	non pertinent
1684	Habig et al.,	2017	BMC Neurol. 2017; 17(1):184	non pertinent
1685	Haaker et al.,	2017	Nat Commun. 2017; 8:15495	non pertinent

1686	Guo et al.,	2017	Archives of physical medicine and rehabilitation. 2017; 98(1):36-42	non pertinent
1687	Guo et al.,	2017	Medicine (united states). 2017; 96(51)	non pertinent
1688	Guillemont et al.,	2017	Health education research. 2017; 32(4):332-342	non pertinent
1689	Guerard et al.,	2017	Orphanet j of rare diseases. 2017; 12(1)	non pertinent
1690	Gucalp et al.,	2017	Cancer research. Conference: 39th annual CTRC-AACR san antonio breast cancer symposium. United states. 2017; 77(4 Supplement 1)	non pertinent
1691	Gu et al.,	2017	Molecular pain. 2017; 13	non pertinent
1692	Grothe et al.,	2017	Osteoarthritis and cartilage. 2017; 25(1):46-52	non pertinent
1693	Groenvold et al.,	2017	Palliative medicine. 2017; 31(9):814-824	non pertinent
1694	Gozzi et al.,	2017	Neuropsychophar macology. 2017; 42(7):1409-1419	non pertinent
1695	Govindasamy et al.,	2017	J of clinical and diagnostic research. 2017; 11(2):RC13-RC16	non pertinent
1696	Gorodetzky et al.,	2017	Drug and alcohol dependence. 2017; 176:79-88	non pertinent
1697	Gordon et al.,	2017	J of sports medicine and physical fitness. 2017; 57(12):1669-1675	non pertinent
1698	Goodman et al.,	2017	J of clinical psychiatry. 2017; 78(1):105-114	non pertinent
1699	Gonzalez-Osuna et al.,	2017	Contemporary clinical trials. 2017; 61:16-22	non pertinent
1700	Gongora et al.,	2017	J of the American College of Cardiology. 2017; 69(11):1428-	non pertinent
1701	Gomez-Rios et al.,	2017	J Matern Fetal Neonatal Med. 2017; 30(4):437-441	non pertinent
1702	Goldthorpe et al.,	2017	J Oral Facial Pain Headache. 2017; 31(1):61-71	non pertinent

1703	Gojo et al.,	2017	Clinical cancer research. 2017; 23(3):697-706	non pertinent
1704	Glincher et al.,	2017	Pediatric blood and cancer. Conference: 30th annual meeting of the american society of pediatric hematology/oncology. ASPHO 2017. Canada; 64:89	non pertinent
1705	Glasser et al.,	2017	J oft he peripheral nervous system. 2017; 22(3):291-292	non pertinent
1706	Gibson	2017	J of gastroenterology and hepatology (australia). 2017; 32:32-35	non pertinent
1707	Ghazikhanian et al.,	2017	Bone Marrow Transplant. 2017; 52(2):279-284	non pertinent
1708	George et al.,	2017	Contemporary clinical trials. 2017; 56-9-17	non pertinent
1709	George et al.,	2017	European j of cancer. 2017; 76:52-59	non pertinent
1710	Gao et al.,	2017	Artificals cells, nanomedicine and biotechnology. 2017; 45(5):1045-1050	non pertinent
1711	Gane et al.,	2017	ACS infectious diseases. 2017; 3(2):144-151	non pertinent
1712	Gagnadoux et al.,	2017	American j of respiratory and critical care medicine. 2017; 195(9):1244-1252	non pertinent
1713	Fukushima et al.,	2017	Supportive care in cancer. 2017; 1-8	non pertinent
1714	Fujioka et al.,	2017	JPEN. J of parenetal and enteral nutrition. 2017; 41(8):1419-1422	non pertinent
1715	Fujino et al.,	2017	EBioMedicine. 2017; 17:199-205	non pertinent
1716	Fries et al.,	2017	Immunity and ageing. 2017; 14(1)	non pertinent
1717	Fried et al.,	2017	J Am Geriatr Soc. 2017; 65(10):2265-2271	non pertinent
1718	Frias et al.,	2017	Diabetes, obesity & metabolism. 2017; 19(1):40-48	non pertinent

1719	Frey et al.,	2017	Pain Physician. 2017; 20(7):E1063-e1072	non pertinent
1720	Freund et al.,	2017	Annals of emergency medicine. 2017; 70(4):30-	non pertinent
1721	Frenck et al.,	2017	Vaccine. 2017; 35(2):375-384	non pertinent
1722	Franko et al.,	2017	J of hand surgery. 2017; 42(3):175-181.e171	non pertinent
1723	Fordyce et al.,	2017	JAMA Cardiol. 2017; 2(4):400-408	non pertinent
1724	Fleischmann et al.,	2017	Arthritis & rheumatology. 2017; 69	non pertinent
1725	Fitzgerald et al.,	2017	New England j of medicine. 2017; 376(1):41-51	non pertinent
1726	Fernandez et al.,	2017	Cerebellum. 2017; 16(1):168-177	non pertinent
1727	Ferencik et al.,	2017	European heart j. 2017; 38:652-653	non pertinent
1728	Fensky et al.,	2017	J of neural transmission. 2017; 124(10):1313-	non pertinent
1729	Feng et al.,	2017	BMJ Open. 2017; 7(10):e017227	non pertinent
1730	Faria et al.,	2017	EBioMedicine. 2017; 24:179-188	non pertinent
1731	Fann et al.,	2017	J of the head trauma rehabilitation. 2017; 32(5):332-342	non pertinent
1732	Fan et al.,	2017	Medicine. 2017; 96(7):e6106	non pertinent
1733	Falloon et al.,	2017	Clinical and vaccine immunology. 2017; 24(9)	non pertinent
1734	Fallon et al.,	2017	British j of pain. 2017; 11(3):119-133	non pertinent
1735	Falla et al.,	2017	Physiotherapy (united kingdom). 2017; 103:e84-	non pertinent
1736	Ezhov et al.,	2017	Atherosclerosis. Supplements 30:187-192	non pertinent
1737	Exler et al.,	2017	Med Decis Making. 2017; 37(6):725-729	non pertinent
1738	Everaerd et al.,	2017	Biological psychiatry. Cognitive neuroscience and neuroimaging. 2017; 2(7):591-598	non pertinent

1739	Euctr – Netherlands Cancer Institute	2017	EU Clinical Trial Register. 2017	non pertinent
1740	Euctr - Roussy	2017	EU Clinical Trial Register. 2017	non pertinent
1741	Euctr – MRI Research Unit. Radiology Department, Hospital del Mar	2017	EU Clinical Trial Register. 2017	non pertinent
1742	Euctr - Alfasigma	2017	EU Clinical Trial Register. 2017	non pertinent
1743	Euctr – RWTH Aachen University/CTC-A	2017	EU Clinical Trial Register. 2017	non pertinent
1744	Emoto et al.,	2017	Endocrine j. 2017; 64(2):191-206	non pertinent
1745	Elkins et al.,	2017	The lancet neurology. 2017; 16(3):217-226	non pertinent
1746	Eljezi et al.,	2017	European j of anesthesiology. 2017; 34(2):56-65	non pertinent
1747	Eljezi et al.,	2017	Pain Physician. 2017; 20(6):	non pertinent
1748	Eker et al.,	2017	J of anesthesia. 2017; 31(2):206-211	non pertinent
1749	Eisner et al.,	2017	Stereotactic and functional neurosurgery. 2017; 95:367-	non pertinent
1750	Ebrahimi et al.,	2017	Neurobiology of learning and memory. 2017; 142(Pt B):209-217	non pertinent
1751	Ebert et al.,	2017	Am J Sports Med. 2017; 45(13):2965-2974	non pertinent
1752	Ebata et al.,	2017	J Bone Joint Surg Am. 2017; 99(5):365-372	non pertinent
1753	Durie et al.,	2017	Lancet (london, england). 2017; 389(10068):519-527	non pertinent
1754	Dunbar et al.,	2017	Clinical and translational science. 2017; 10(6):455-469	non pertinent
1755	Dufour et al.,	2017	International j of cardiology. 2017; 228:754-760	non pertinent
1756	Duffield et al.,	2017	Anesthesia and Analgesia. 2017; 124(3):857-862	non pertinent
1757	Drottning et al.,	2017	J of headache and pain. 2017; 18(1)	non pertinent
1758	Drks - Lis	2017	Deutsches Register klinischer Studien. 2017	non pertinent
1759	Drks - Haukkala	2017	Deutsches Register klinischer Studien. 2017	non pertinent

1760	Drks - Boecker	2017	Deutsches Register klinischer Studien. 2017	non pertinent
1761	Drks - Lotze	2017	Deutsches Register klinischer Studien. 2017	non pertinent
1762	Drks – Meyer-Friessem	2017	Deutsches Register klinischer Studien. 2017	non pertinent
1763	Drks - Flor	2017	Deutsches Register klinischer Studien. 2017	non pertinent
1764	Dougados et al.,	2017	Annals of the rheumatic diseases. 2017; 76(1):88-95	non pertinent
1765	Dorff et al.,	2017	Cancer. 2017; 123(23):4566-4573	non pertinent
1766	Doran et al.,	2017	J of nuclear cardiology. 2017; 24(3):1062-1070	non pertinent
1767	Donaldson et al.,	2017	Brain stimulation. Conference: 2nd international brain stimulation conference. Spain. 2017; 10(2):378	non pertinent
1768	Dolzhikova et al.,	2017	Human vaccines and immunotherapeutics. 2017; 13(3):613-620	non pertinent
1769	Dessery et al.,	2017	Prosthetics and orthotics international. 2017; 41(4):356-363	non pertinent
1770	Derksen et al.,	2017	J of cachexia, sarcopenia and muscle. 2017; 8(6):1013-	non pertinent
1771	Dengler et al.,	2017	Pain Physician. 2017; 20(6):537-550	non pertinent
1772	Deng et al.,	2017	PLoS One. 2017; 12(12)	non pertinent
1773	DelBello et al.,	2017	J of the American Academy of Child and Adolescent Psychiatry. 2017; 56(12):1015-1025	non pertinent
1774	Delage et al.,	2017	Trials. 18(1):517	non pertinent
1775	Del Blanco et al.,	2017	Nurse education today. 2017; 55:45-53	non pertinent
1776	Del Blanco et al.,	2017	Nurse Educ Today. 2017; 55:45-53	non pertinent

1777	Dehghani et al.,	2017	American heart j. 2017; 192-105-112	non pertinent
1778	Dedic et al.,	2017	European heart j. 2017; 38:90-	non pertinent
1779	De Prates et al.,	2017	Clinical infectious diseases. 2017; 64(1):67-71	non pertinent
1780	De Niet et al.,	2017	The lancet. Gastroenterology & hepatology. 2017; 2(8):576-584	non pertinent
1781	De la Gala et al.,	2017	British j of anaesthesia. 2017; 119(4):655-663	non pertinent
1782	Davis et al.,	2017	Br J Neurosurg. 2017; 31(2):205-208	non pertinent
1783	Daud et al.,	2017	J of hematology & oncology. 2017; 10(1):1-9	non pertinent
1784	Das et al.,	2017	PLoS One. 2017; 12(3)	non pertinent
1785	Danne et al.,	2017	J of pediatrics. 2017; 181:146-153	non pertinent
1786	Dagan et al.,	2017	Experimental brain research. 2017; 235(8):2463-2472	non pertinent
1787	Dadey	2017	American j of therapeutics. 2017; 24(4):e373-e380	non pertinent
1788	Curtin et al.,	2017	J of hand surgery. 2017; 42(3):166-174	non pertinent
1789	Cunningham et al.,	2017	The lancet. Oncology. 2017; 18(3):357-370	non pertinent
1790	Culver et al.,	2017	Investigative opthalmology & visual science. 2017; 58(6):BIO52-BIO60	non pertinent
1791	Ctrl - Kumar	2017	Clinical Trials Registry India. 2017	non pertinent
1792	Ctrl - Mallikarjunaiah	2017	Clinical Trials Registry India. 2017	non pertinent
1793	Cruz-Diaz et al.,	2017	Complementary therapies in medicine. 2017; 33:72-77	non pertinent
1794	Cremon et al.,	2017	Alimentary pharmacology & therapeutics. 2017; 45(7):909-922	non pertinent
1795	Cranston et al.,	2017	Trials. 2017; 18(1):111	non pertinent

1796	Cranen et al.,	2017	J of medical internet research. 2017; 19(1):e26	non pertinent
1797	Coyne et al.,	2017	Clinical therapeutics. 2017; 39(1):75-88	non pertinent
1798	Coyle et al.,	2017	Alimentary pharmacology & therapeutics. 2017; 45(12):1524-1533	non pertinent
1799	Cortes et al.,	2017	Brain stimulation. 2017; 10(1):e13-	non pertinent
1800	Cortes et al.,	2017	Haematologica. 2017; 102(3):519-528	non pertinent
1801	Cooper et al.,	2017	Physiotherapy (united kingdom). 2017; 103:e117-e118	non pertinent
1802	Collaku et al.,	2017	J of pain research. 2017; 10:669-678	non pertinent
1803	Colic et al.,	2017	European neuropsychopharmacology. 2017; 27:704-705	non pertinent
1804	Coffeng et al.,	2017	European j of neurology. Conference: 3rd congress of the european academy of neurology. Netherlands. 2017; 24:220	non pertinent
1805	Clave et al.,	2017	Therapeutic advances in gastroenterology. 2017; 10(3):311-322	non pertinent
1806	Clark	2017	Cardiovascular and interventional radiology. 2017; 40(2):94-	non pertinent
1807	Chupp et al.,	2017	Lancet Respir Med. 2017; 5(5):390-400	non pertinent
1808	Chung et al.,	2017	Arthrithis care & research. 2017; 69(7):973-981	non pertinent
1809	Chu et al.,	2017	J of addiction medicine. 2017; 11(5):342-349	non pertinent
1810	Chomiak et al.,	2017	Medicine (Baltimore). 2017; 96(5):e5934	non pertinent
1811	Choi et al.,	2017	Medicine (Baltimore). 2017; 96(16):e6670	non pertinent
1812	Choe et al.,	2017	Clin Exp Pharmacol Physiol. 2017; 44(1):30-40	non pertinent

1813	Cho et al.,	2017	BMC complementary and alternative medicine. 2017; 17(1)	non pertinent
1814	Cho et al.,	2017	International j of clinical practice. 2017; 71(5)	non pertinent
1815	Chi – Dapeng	2017	Chinese Clinical Trial Registry. 2017	non pertinent
1816	Chi – Aihong	2017	Chinese Clinical Trial Registry. 2017	non pertinent
1817	Chi –Shi	2017	Chinese Clinical Trial Registry. 2017	non pertinent
1818	Chi – Zeng	2017	Chinese Clinical Trial Registry. 2017	non pertinent
1819	Cheng et al.,	2017	Sci Rep. 2017; 7(1):13054	non pertinent
1820	Chen et al.,	2017	PLoS One. 2017; 12(5)	non pertinent
1821	Chen et al.,	2017	Molecular genetics and metabolism. 2017; 120(1-2):35-	non pertinent
1822	Chatelain et al.,	2017	J of clinical endocrinology and metabolism. 2017; 102(5): 1673-1682	non pertinent
1823	Chapman et al.,	2017	Annals of oncology. 2017; 28(10):2581-2587	non pertinent
1824	Chang et al.,	2017	PLoS One. 2017; 12(6):e0180328	non pertinent
1825	Chai et al.,	2017	IEEE Trans Neural Syst Rehabil Eng. 2017; 25(5):469-480	non pertinent
1826	Cavalli et al.,	2017	Soc Cogn Affect Neurosci. 2017; 12(6):976-983	non pertinent
1827	Cattaneo et al.,	2017	J of parkinson's disease. 2017; 7(1):95-101	non pertinent
1828	Castro-Sanchez et al.,	2017	Pain Physician. 2017; 20(2):37-52	non pertinent
1829	Castro et al.,	2017	J Clin Monit Comput. 2017; 31(4):851-860	non pertinent
1830	Cash et al.,	2017	Therapeutic advances in gastroenterology. 2017; 10(9):689-699	non pertinent

1831	Cascavilla et al.,	2017	Haematologica. Conference: 46th congress of the italian society of hematology. Italy. 2017; 102(Supplement 3):112-113	non pertinent
1832	Carello et al.,	2017	Italian j of pediatrics. 2017; 43(1)	non pertinent
1833	Cao et al.,	2017	Pain Physician. 2017; 20(5):E687-e699	non pertinent
1834	Candalh-Touta et al.,	2017	International j of computer assisted radiology and surgery. 2017; 12(1):132-133	non pertinent
1835	Campbell et al.,	2017	Pain Physician. 2017; 20(1):E183-E193	non pertinent
1836	Campbell et al.,	2017	World j of gastroenterology. 2017; 23(3):560-562	non pertinent
1837	Cameron et al.,	2017	The lancet. Oncology. 2017; 18(7):929-945	non pertinent
1838	Calvino	2017	Douleurs. 2017; 18(1):3-8	non pertinent
1839	Caicedo et al.,	2017	International j of surgery case reports. 2017; 33:135-138	non pertinent
1840	Cai et al.,	2017	Diabetic medicine. 2017; 34(6):813-820	non pertinent
1841	Caeyenberghs et al.,	2017	American j of neuroradiology. 2017; 38(1):183-191	non pertinent
1842	Cadoni et al.,	2017	Gastrointestinal endoscopy. 2017; 85(1):210-218.e211	non pertinent
1843	R.B.R. c73cm - Ribeiro	2017	Registro Brasileiro de Ensaios Clínicos. 2017	non pertinent
1844	Brolese et al.,	2017	Surgical endoscopy and other interventional techniques. 2017; 31(2):503-	non pertinent
1845	Brock et al.,	2017	Trials. 2017; 18(1)	non pertinent
1846	Briolo et al.,	2017	Expert review of hematology. 2017; 10(3):193-205	non pertinent
1847	Braun et al.,	2017	Annals of the rheumatic diseases. 2017; 76(6):1070-1077	non pertinent

1848	Bradley et al.,	2017	Pediatrics. 2017; 139(3)	non pertinent
1849	Bouffard et al.,	2017	J Neuroophthalmol. 2017; 37(2):122-125	non pertinent
1850	Bottelier et al.,	2017	Psychiatry research. Neuroimaging. 2017; 269:36-42	non pertinent
1851	Bossi et al.,	2017	Annals of oncology: official j of the european society for medical oncology. 2017; 28(10):2547-2551	non pertinent
1852	Bosanquet et al.,	2017	Trials. 2017; 18(1)	non pertinent
1853	Borckardt et al.,	2017	Brain stimulation. 2017	non pertinent
1854	Bond et al.,	2017	BMC pregnancy and childbirth. 2017; 17(1)	non pertinent
1855	Bolm et al.,	2017	J of surgical research. 2017; 212:246-252	non pertinent
1856	Boland et al.,	2017	Human psychopharmacology. 2017; 32(1)	non pertinent
1857	Bogliacino et al.,	2017	Proceedings of the National Academy of Sciences of the United States of America. 2017; 114(32):8505-8510	non pertinent
1858	Boettcher et al.,	2017	Human reproduction. 2017; 32.i257-i258	non pertinent
1859	Boateng et al.,	2017	Brain stimulation. 2017; 10(4):e82-e83	non pertinent
1860	Blum et al.,	2017	Leukemia. 2017; 31(1):34-39	non pertinent
1861	Bloos et al.,	2017	Intensive Care Med. 2017; 43(11):1602-1612	non pertinent
1862	Blom et al.,	2017	J of clinical lipidology. Conference: 2017 annual scientific sessions of the national lipid association. United states. 2017; 11(3):816	non pertinent
1863	Bloembergen et al.,	2017	Osteoporosis international. 2017; 28:635-636	non pertinent

1864	Bissonnette et al.,	2017	J of the American Academy of Dermatology. 2017; 76(1):33-39	non pertinent
1865	Birring et al.,	2017	The lancet. Respiratory medicine. 2017; 5(10):806-815	non pertinent
1866	Birring et al.,	2017	BMJ Open. 2017; 7(1):e014112	non pertinent
1867	Bilimoria et al.,	2017	Jt Comm J Qual Patient Saf. 2017; 43(5):241-250	non pertinent
1868	Bierma-Zeinstra et al.,	2017	Osteoarthritis and cartilage. 2017; 25:439-440	non pertinent
1869	Beyer-Westendorf et al.,	2017	The lancet. Haematology. 2017; 4(3):e105-e113	non pertinent
1870	Bethlehem et al.,	2017	Transl Psychiatry. 2017; 7(4):e1099	non pertinent
1871	Bertsch et al.,	2017	Psychoneuroendocrinology. 2017; 83:49-	non pertinent
1872	Bergmann et al.,	2017	Spine. 2017	non pertinent
1873	Berginström et al.,	2017	J of head trauma rehabilitation. 2017; 32(2):E46-E54	non pertinent
1874	Berger et al.,	2017	Frontiers in immunology. 2017; 8(NOV)	non pertinent
1875	Benson et al.,	2017	Oncologist. 2017; 22(3):241-242 and e249-e215	non pertinent
1876	Benedetti et al.,	2017	Bone marrow transplantation. Conference: 43rd annual meeting of the european society for blood and marrow transplantation. France. 2017; 52(Supplement 1):393	non pertinent
1877	Bendell et al.,	2017	British j of cancer. 2017; 116(5):575-583	non pertinent
1878	Bendell et al.,	2017	Oncologist. 2017; 22(3):264-271	non pertinent
1879	Bello et al.,	2017	Arthritis & rheumatology (hoboken, N.J.). 2017; 69(8):1661-1669	non pertinent
1880	Beissner et al.,	2017	Home healthcare now. 2017; 35(2):105-112	non pertinent
1881	Beissner et al.,	2017	Biological psychiatry. 2017 Aug.	non pertinent

1882	Beinholzl et al.,	2017	European psychopharmacology. 2017; 27:718-719	non pertinent
1883	Beer et al.,	2017	The lancet. Oncology. 2017; 18(11):1532-1542	non pertinent
1884	Beaver et al.,	2017	Clinical cancer research. 2017; 23(14):3479-3483	non pertinent
1885	Beall et al.,	2017	Pain Physician. 2017; 20(6):521-528	non pertinent
1886	Bauer et al.,	2017	J Nucl Med. 2017; 58(4):678-681	non pertinent
1887	Barnebey et al.,	2017	American j of ophthalmology. 2017; 176:61-69	non pertinent
1888	Barker-Davies et al.,	2017	BMC musculoskeletal disorders. 2017; 18(1):204	non pertinent
1889	Barban et al.,	2017	Brain sciences. 2017; 7(2)	non pertinent
1890	Baraniuk et al.,	2017	Sci Rep. 2017; 7(1):15338	non pertinent
1891	Banerji et al.,	2017	New England j of medicine. 2017; 376(8):717-728	non pertinent
1892	Bally et al.,	2017	BMJ Open. 2017; 7(7)	non pertinent
1893	Bally et al.,	2017	Peerj. 2017; 2017(5)	non pertinent
1894	Badran et al.,	2017	Brain stimulation. Conference: 2nd international brain stimulation conference. Spain. Conference start: 20170305. Conference end: 20170308. 2017; 10(2):378	non pertinent
1895	Badday et al.,	2017	American j of respiratory and critical care medicine. 2017; 195	non pertinent
1896	Babakhani et al.,	2017	J Neurosurg Anesthesiol. 2017; 29(3):291-297	non pertinent
1897	Azria et al.,	2017	Annals of oncology. 2017; 28(10):2436-2442	non pertinent
1898	Avrillon et al.,	2017	Future oncology. (london, england). 2017; 13(4):321-335	non pertinent
1899	Avidan et al.,	2017	Lancet. 2017; 390(10091):267-275	non pertinent
1900	Auyong et al.,	2017	Anesthesia and Analgesia. 2017; 124(3):959-965	non pertinent

1901	Asgari et al.,	2017	J of clinical anesthesia. 2017; 38:13-17	non pertinent
1902	Asgari et al.,	2017	Pain research & management. 2017:1721460	non pertinent
1903	Asgari et al.,	2017	Pain research & management. 2017	non pertinent
1904	Arnold et al.,	2017	Neurology. 2017; 89(7):710-713	non pertinent
1905	Armbruster et al.,	2017	Horm Behav. 2017; 94:97-105	non pertinent
1906	Arima et al.,	2017	Anticancer research. 2017; 37(2):909-914	non pertinent
1907	Arabmotlagh et al.,	2017	J of orthopaedic research. 2017; 35(1):154-159	non pertinent
1908	Antonini et al.,	2017	European neuropsychophar macology. 2017; 27:1105-1106	non pertinent
1909	Anton et al.,	2017	Alcoholism, clinical and experimental research. 2017; 41(7):1370-1380	non pertinent
1910	Andrews et al.,	2017	Crit Care Med. 2017; 45(5):883-890	non pertinent
1911	Amiri farahani et al.,	2017	Archives of gynecology and obstetrics. 2017; 296(2):277-283	non pertinent
1912	Alohali et al.,	2017	Brain stimulation. 2017; 10(4):e46-e47	non pertinent
1913	Almadori et al.,	2017	Arthrithis & rheumatology. 2017; 69	non pertinent
1914	Aliberti et al.,	2017	Pulmonary pharmacology & therapeutics. 2017; 45:191-201	non pertinent
1915	Alhussien et al.,	2017	Brain stimulation. 2017; 10(4):e47-e48	non pertinent
1916	Alders et al.,	2017	Menopause (New York, N.Y.). 2017; 24(12):1453-	non pertinent
1917	Alamdarsaravi et al.,	2017	Psychiatry research. 2017; 255-59-65	non pertinent
1918	Aitken et al.,	2017	Osteoporosis international. 2017; 28(1):59-	non pertinent
1919	Ahn et al.,	2017	J of pain. 2017; 18(4):87-88	non pertinent
1920	Aggarwal et al.,	2017	International J of Oral and Maxillofacial Surgery. 2017; 46:223-	non pertinent

1921	Adelowo et al.,	2017	Female pelvic medicine & reconstructive surgery. 2017; 23(1):1-7	non pertinent
1922	Adams et al.,	2017	Neuropsychopharmacology. 2017; 43:295-	non pertinent
1923	Actrn – Hazratwala	2017	Australian New Zealand Clinical Trials Registry. 2017	non pertinent
1924	Actrn - Ho	2017	Australian New Zealand Clinical Trials Registry. 2017	non pertinent
1925	Actrn - Fitzgibbon	2017	Australian New Zealand Clinical Trials Registry. 2017	non pertinent
1926	Actrn – Innes-Wong	2017	Australian New Zealand Clinical Trials Registry. 2017	non pertinent
1927	Actrn – Yates	2017	Australian New Zealand Clinical Trials Registry. 2017	non pertinent
1928	Actrn – Haines	2017	Australian New Zealand Clinical Trials Registry. 2017	non pertinent
1929	Actrn – Armstrong	2017	Australian New Zealand Clinical Trials Registry. 2017	non pertinent
1930	Actrn – Lowe	2017	Australian New Zealand Clinical Trials Registry. 2017	non pertinent
1931	Actrn –Rickard	2017	Australian New Zealand Clinical Trials Registry. 2017	non pertinent
1932	Actrn – Singh	2017	Australian New Zealand Clinical Trials Registry. 2017	non pertinent
1933	Actrn – Wahid	2017	Australian New Zealand Clinical Trials Registry. 2017	non pertinent
1934	Actrn – Ewais	2017	Australian New Zealand Clinical Trials Registry. 2017	non pertinent
1935	Actrn – Khow	2017	Australian New Zealand Clinical Trials Registry. 2017	non pertinent
1936	Abdel-Kader et al.,	2017	Int Urol Nephrol. 2017; 49(1):27-30	non pertinent
1937	Abbas et al.,	2017	Contraception. 2017; 95(3):251-256	non pertinent

1938	Aaron et al.,	2017	Clinical j of sport medicine. 2017; 27(3):271-277	non pertinent
1939	Ezhov et al.,	2017	Atherosclerosis supplements. 2017; 30(pp187-192)	non pertinent
1940	Feng et al.,	2017	Chinese j of tissue engineering research. 2017; 21(35):5630-5635	non pertinent
1941		2017	Supportive care in cancer. Conference: 2017 international MASCC/ISOO Symposium: Supportive Care in Cancer. United States. 2017; 25(2 Supplement 1):236	non pertinent
1942	Bhattacharyya et al.,	2017	Scientific reports. 2017; 7(1):15025-	non pertinent
1943	Geusens et al.,	2017	Osteoporosis international. Conference: World Congress on Osteoporosis, Osteoarthritis and Musculoskeletal Diseases, WCO-IOF-ESCEO 2017. Italy; 28(1 Supplement 1):51-52	non pertinent
1944	Zinkovich et al.,	2017	Annals of oncology. Conference: 42nd ESMO Congress, ESMO 2017. Spain; 28(Supplement 5):v121	non pertinent
1945		2017	Neuropsychopharmacology. Conference: 56th Annual Meeting of the American College of Neuropsychopharmacology, ACNP 2017. United States; 43(Supplement 1):198	non pertinent

1946		2017	Neuropsychopharmacology. Conference: 56th Annual Meeting of the American College of Neuropsychopharmacology, ACNP 2017. United States; 43(Supplement 1):347	non pertinent
1947	Soravia et al.,	2017	Psychoneuroendocrinology. Conference: 47th Annual Meeting of the International Society of Psychoneuroendocrinology Genes and Hormones: Key Factors of Wellbeing Throughout the Life Span. Switzerland. 2017; 83(Supplement 1):41	non pertinent
1948	Gaebel et al.,	2017	European archieves of psychiatry and clinical neuroscience. Conference: 6th European Conference on Schizophrenia Research: Advancing Research – Promoting Recovery. Germany. 2017; 267(1 Supplement 1):93-94	non pertinent
1949	Klass et al.,	2017	Neurobiology of learning and memory. 2017; 144(pp235-247)	non pertinent
1950	Jeong et al.,	2017	J of orthopaedic surgery (hong kong). 2017; 25(2):2309499017718908)	non pertinent

1951	Patrick et al.,	2017	Biological psychiatry. Conference: 72nd Annual Scientific Convention and Meeting of the Society of Biological Psychiatry, SOBP 2017. United States; 81(10 Supplement 1):70	non pertinent
1952	Karsan et al.,	2017	Cephalalgia. Conference: 18th Congress of the International Headache Society, ICH 2017. Canada; 37(1 Supplement 1):28	non pertinent
1953		2017	Neuropsychopharmacology. Conference: 56th Annual Meeting of the American College of Neuropsychopharmacology, ACNP 2017. United States. 43(Supplement 1):569	non pertinent
1954	Gude et al.,	2017	Implementation science. 2017; 12(1):68	non pertinent
1955	Bandak et al.,	2017	Osteoarthritis and cartilage. Conference: 2017 Osteoarthritis Research Society International, OARSI World Congress. United States; 25(Supplement 1):15	non pertinent
1956	Zunhammer et al.,	2016	Sci Rep. 2016; 6:31606	non pertinent
1957	Zielinski et al.,	2016	The j of trauma and acute care surgery. 2016; 80(2):237-242	non pertinent
1958	Ziauddeen et al.,	2016	Neuropsychopharmacology. 2016; 41(11):2647-2657	non pertinent
1959	Zhou et al.,	2016	Orthopaedic surgery. 2016; 8(1):44-50	non pertinent
1960	Zheng et al.,	2016	Medicine (united states). 2016; 95(24)	non pertinent
1961	Zhao et al.,	2016	Anti-cancer drugs. 2016; 27(7):689-694	non pertinent

1962	Zhang et al.,	2016	Biomedical research (india). 2016; 27(2):401-405	non pertinent
1963	Zhai et al.,	2016	BMC aneshesiology. 2016; 16(1):80	non pertinent
1964	Zhai et al.,	2016	National medical j of china. 2016; 96(28):2229-2233	non pertinent
1965	Zeidan et al.,	2016	J of neuroscience. 2016; 36(11):3391-3397	non pertinent
1966	Zecca et al.,	2016	J of pain and symptom management. 2016; 52(6):783-794.e786	non pertinent
1967	Zdenkowski et al.,	2016	Annals of oncology: official j of the european society for medical oncology. 2016; 27(5):806-812	non pertinent
1968	Yuksel et al.,	2016	Annals of the rheumatic diseases. 2016; 75:1307-	non pertinent
1969	Yuan et al.,	2016	Cancer prevention research (philadelphia, pa.). 2016; 9(5):396-405	non pertinent
1970	Yu et al.,	2016	Medical science monitor. 2016; 22:1687-1693	non pertinent
1971	Yovell et al.,	2016	American j of psychiatry. 2016; 173(5):491-498	non pertinent
1972	Youssef et al.,	2016	Obesity surgery. 2016; 26(3):494-504	non pertinent
1973	Younes et al.,	2016	Lancet oncology. 2016; 17(9):1283-1294	non pertinent
1974	Yoshino et al.,	2016	Cancer science. 2016; 107(5):659-665	non pertinent
1975	Yoon et al.,	2016	Gut and liver. 2016; 10(4):520-525	non pertinent
1976	Yokomizo et al.,	2016	J of urology. 2016; 195(1):41-46	non pertinent
1977	Yin et al.,	2016	Medicine (united states). 2016; 95(12)	non pertinent
1978	Yeka et al.,	2016	J of infectious diseases. 2016; 213(7):1134-1142	non pertinent
1979	Ydemann et al.,	2016	Danish medical j. 2016; 63(6):1-5	non pertinent

1980	Yayehd et al.,	2016	Arch Cardiovasc Dis. 2016; 109(1):4-12	non pertinent
1981	Yasuda et al.,	2016	J of diabetes investigation. 2016; 7(1):100-108	non pertinent
1982	Yao et al.,	2016	J of clinical anesthesia. 2016; 31:13-18	non pertinent
1983	Yao et al.,	2016	Neuroimage. 2016; 130:230-240	non pertinent
1984	Yao et al.,	2016	Medicine (united states). 2016; 95(26)	non pertinent
1985	Yan et al.,	2016	PLoS One. 2016; 11(12)	non pertinent
1986	Yan et al.,	2016	BMC musculoskeletal disorders. 2016; 17(1)	non pertinent
1987	Yamaguchi et al.,	2016	Medical mycology j. 2016; 57(4):E93-E110	non pertinent
1988	Xu et al.,	2016	Nutrition j. 2016; 15:9	non pertinent
1989	Xiao et al.,	2016	Chin J Integr Med. 2016; 22(11):846-854	non pertinent
1990	Wudneh et al.,	2016	Therapeutics and clinical risk management. 2016; 12:1293-1300	non pertinent
1991	Wu et al.,	2016	International j of clinical pharmacology and therapeutics. 2016; 54(12):935-949	non pertinent
1992	Wu et al.,	2016	Prostaglandins & other lipid mediators. 2016; 126:24-28	non pertinent
1993	Wood et al.,	2016	Spine j. conference: 31st Annual Meeting of the North American Spine Society. United States. 2016; 16(10 Supplement 1):142	non pertinent
1994	Winther et al.,	2016	PLoS One. 2016; 11(6)	non pertinent
1995	Wilson et al.,	2016	Trials. 2016; 17(1)	non pertinent
1996	Willick et al.,	2016	J of nuclear medicine. 2016; 57	non pertinent
1997	Williams	2016	Neuropsychopharmacology. 2016; 41:75	non pertinent

1998	Wijayasinghe et al.,	2016	British j of anaesthesia. 2016; 116(6):829-837	non pertinent
1999	Wermke et al.,	2016	British j of haematology. 2016; 175(5):917-924	non pertinent
2000	Webster et al.,	2016	Pain medicine (Malden, Mass.). 2016; 17(6):1112-1130	non pertinent
2001	Webb et al.,	2016	European j of clinical pharmacology. 2016; 72(4):447-457	non pertinent
2002	Waterschoot et al.,	2016	European urology, supplements. Conference: 8th meeting of the EAU section of genito-urinary reconstructive surgeons. ESGURS 2016. Spain; 15(8):377	non pertinent
2003	Ware et al.,	2016	Lancet. 2016; 387(10019):661-670	non pertinent
2004	Wanigasekera et al.,	2016	Anesthesiology. 2016; 124(1):159-168	non pertinent
2005	Wang et al.,	2016	Magn Reson Imaging. 2016; 34(5):609-616	non pertinent
2006	Wang et al.,	2016	Osteoarthritis and cartilage. 2016; 24:327-	non pertinent
2007	Wang et al.,	2016	J of the pediatric infectious diseases society. 2016; 5(2):170-179	non pertinent
2008	Wang et al.,	2016	Oncotarget. 2016; 7(14):18705-18712	non pertinent
2009	Walter et al.,	2016	Neuropsychopharmacology. 2016; 41(6):1659-1669	non pertinent
2010	Walsh et al.,	2016	BMJ Open. 2016; 6(3):e010148	non pertinent
2011	Walsh et al.,	2016	The lancet. Respiratory medicine. 2016; 4(10):807-817	non pertinent
2012	Wald et al.,	2016	Jama. 2016; 316(23):2495-2503	non pertinent
2013	Waelinitz et al.,	2016	J of obstetrics and gynaecology. 2016; 36(1):106-113	non pertinent
2014	Wade et al.,	2016	BMJ Open. 2016; 6(1):e008166	non pertinent

2015	Volz et al.,	2016	Pain. 2016; 157(2):429-437	non pertinent
2016	Vinik et al.,	2016	BMC neurology. 2016; 16(1)	non pertinent
2017	Vinik et al.,	2016	Target oncology. 2016; 11(6):815-824	non pertinent
2018	Vidal et al.,	2016	J of pain and symptom management. 2016; 52(4):570-574.e579	non pertinent
2019	Vey et al.,	2016	Oncotarget. 2016; 7(22):32532-32542	non pertinent
2020	Veselis et al.,	2016	Sleep Med. 2016; 27-28:115-120	non pertinent
2021	Verstovsek et al.,	2016	J of hematology & oncology. 2016; 9(1)	non pertinent
2022	Vermersch et al.,	2016	European neurology. 2016; 76(5-6):216-226	non pertinent
2023	Vercruyssen et al.,	2016	Clin Oral Implants Res. 2016; 27(4):427-432	non pertinent
2024	Velipasaoglu et al.,	2016	J of maternal-fetal & neonatal medicine. 2016; 29(23):3838-3842	non pertinent
2025	Vecchio et al.,	2016	Neuroscience letters. 2016; 626:149-157	non pertinent
2026	Vaseghi et al.,	2016	European j of neuroscience. 2016; 43(9):1161-1172	non pertinent
2027	Van Rooij et al.,	2016	Neuropsychopharmacology. 2016; 41:295-	non pertinent
2028	Van Lunzen et al.,	2016	AIDS (london, england). 2016; 30(2):251-259	non pertinent
2029	Van Hoorn et al.,	2016	European j of obstetrics, gynecology and reproductive biology. 2016; 197:168-173	non pertinent
2030	Van Cutsem et al.,	2016	Targeted oncology. 2016; 11(3):383-400	non pertinent
2031	Van Broekhoven et al.,	2016	BMC Cancer. 2016; 16:686	non pertinent
2032	Vacas et al.,	2016	Anesthesia and Analgesia. 2016; 123(1):206-212	non pertinent
2033	Ursing et al.,	2016	PLoS One. 2016; 11(9)	non pertinent
2034	Urquhart et al.,	2016	Trials. 2016; 17(1):514	non pertinent
2035	Urbanska et al.,	2016	Pediatric infectious disease j. 2016; 35(2):142-145	non pertinent

2036	Ugwu et al.,	2016	International j of gynaecology and obstetrics. 2016; 133(2):173-177	non pertinent
2037	Ueno et al.,	2016	International j of molecular sciences. 2016; 17(6)	non pertinent
2038	Udristoiu et al.,	2016	J of affective disorders. 2016; 199:6-12	non pertinent
2039	Uchida et al.,	2016	Blood. 2016; 127(13):1633-1641	non pertinent
2040	Tsukada et al.,	2016	The bone & joint j. 2016; 98-B(2):194-200	non pertinent
2041	Tsuchiya et al.,	2016	International j of clinical oncology. 2016; 21(6):1085-1090	non pertinent
2042	Tsianakas et al.,	2016	Experimental dermatology. 2016; 25(6):428-433	non pertinent
2043	Tryfonidis et al.,	2016	European j of cancer (Oxford, England : 1990). 2016; 53:144-154	non pertinent
2044	Truong et al.,	2016	J of the american heart association. 2016; 5(3):e003137	non pertinent
2045	Trneny et al.,	2016	The lancet. Oncology. 2016; 17(3):319-331	non pertinent
2046	Torres et al.,	2016	Clinical j of the American Society of Nephrology. 2016; 11(5):803-811	non pertinent
2047	Tongpeth et al.,	2016	Circulation. 2016; 134	non pertinent
2048	Thybo et al.,	2016	BMC anesthesiology. 2016; 16:21	non pertinent
2049	Thomas et al.,	2016	Oral radiology. 2016; 32(3):160-166	non pertinent
2050	Thirumalai et al.,	2016	J of clinical endocrinology and metabolism. 2016; 101(7):2937-2944	non pertinent
2051	Thera et al.,	2016	Malaria j. 2016; 15(1)	non pertinent
2052	Tetreault et al.,	2016	PLoS Biol. 2016; 14(10):e1002570	non pertinent
2053	Tepes et al.,	2016	European j of gastroenterology & hepatology. 2016; 28(6):676-683	non pertinent

2054	Ten Hove et al.,	2016	J of neuro-ophthalmology. 2016; 36(1):13-19	non pertinent
2055	Telles et al.,	2016	Med Sci Monit. 2016; 22:3228-3247	non pertinent
2056	Telen et al.,	2016	Vox sanguinis. Conference: 34th international congress of the international society of blood transfusion. United arab emirates. Conference start:20160903. Conference end:20160908. 2016; 111:64	non pertinent
2057	Tarrago Mda et al.,	2016	Medicine (Baltimore). 2016; 95(17):e3353	non pertinent
2058	Tang et al.,	2016	J of orthopaedic surgery (hong kong). 2016; 24(2):183-187	non pertinent
2059	Tang et al.,	2016	Lancet oncology. 2016; 17(12):1720-1731	non pertinent
2060	Tandon et al.,	2016	J of psychopharmacology (Oxford, England). 2016; 30(1):69-77	non pertinent
2061	Tanaka et al.,	2016	Molecular medicine (Cambridge, Mass.). 2016; 22:338-348	non pertinent
2062	Tan et al.,	2016	Clinical and experimental pharmacology & physiology. 2016; 43(3):304-311	non pertinent
2063	Tan et al.,	2016	Mediators of inflammation. 2016; 2016:4173962	non pertinent
2064	Talan et al.,	2016	Annals of emergeny medicine. 2016 Jul	non pertinent
2065	Takemaru et al.,	2016	J of plastic, reconstructive & aesthetic surgery. 2016; 69(4):493-496	non pertinent

2066	Takahashi et al.,	2016	FASEB j. conference: experimental biology 2016, EB San diego, CA united states. Conference start:20160402. Conference end:20160406. Conference publication: (var.pagings); 30	non pertinent
2067	Tack et al.,	2016	Neurogastroenterology and motility. 2016; 28(4):487-497	non pertinent
2068	Svaton et al.,	2016	European surgery – acta chirurgica austrica. Conference: 29th european federation congress of the international college of surgeons, ICS 2015. Czech republic; 48(3 Supplement 1):194-196	non pertinent
2069	Suttiruksa et al.,	2016	Asian biomedicine. 2016; 10(5):435-445	non pertinent
2070	Sun et al.,	2016	Menopause (New York N.Y.). 2016; 23(7):740-748	non pertinent
2071	Sun et al.,	2016	Clinical therapeutics. 2016; 38(1):31-38	non pertinent
2072	Sulzberger et al.,	2016	J of the european academy of dermatology and verenology: JEADV. 2016; 30 Suppl 1:9-17	non pertinent
2073	Sultana et al.,	2016	American j of gastroenterology. 2016; 111:906-907	non pertinent
2074	Su et al.,	2016	J of child and adolescent psychopharmacology. 2016; 26(4):362-371	non pertinent
2075	Stundner et al.,	2016	Br J Anaesth. 2016; 116(3):405-412	non pertinent
2076	Strowitzki et al.,	2016	Reproductive biology and endocrinology. 2016; 14(1)	non pertinent

2077	Stoustrup et al.,	2016	J of rheumatology. Conference: 71st annual meeting of the canadian rheumatology association. CRA 2016, Canada. Conference start:20160217. Conference end:20160220; 43(6):1209-1210	non pertinent
2078	Storme et al.,	2016	Gynecological surgery. 2016; 13(3):139-146	non pertinent
2079	Stinson, Conelly et al.,	2016	Pediatric rheumatology. 2016; 14	non pertinent
2080	Stinson, Gatsonis et al.,	2016	Pediatric rheumatology. 2016; 14	non pertinent
2081	Stillman et al.,	2016	Am Heart J. 2016; 179:19-28	non pertinent
2082	Steiner et al.,	2016	BMC complementary and alternative medicine. 2016; 16(1)	non pertinent
2083	Satrkstein et al.,	2016	J of stroke and cerebrovascular diseases. 2016; 25(5):1119-1127	non pertinent
2084	Srivastava et al.,	2016	J of clinical and diagnostic research. 2016; 10(9):UC01-UC04	non pertinent
2085	Spiridigliozzi et al.,	2016	American j of medical genetics. Part a. 2016; 170(6):1545-1555	non pertinent
2086	Soriano-Maldonado et al.,	2016	PLoS One. 2016; 11(2)	non pertinent
2087	Sonne et al.,	2016	BMC Psychiatry. 2016; 16(1)	non pertinent
2088	Song et al.,	2016	J of digestive diseases. 2016; 17(4):260-267	non pertinent
2089	Song et al.,	2016	Helicobacter. 2016; 21(5):382-388	non pertinent
2090	Song et al.,	2016	International orthopaedics. 2016; 40(2):295-299	non pertinent
2091	Song et al.,	2016	J of acquired immune deficiency syndromes. 2016; 72(4):400-407	non pertinent
2092	Sohoni et al.,	2016	American j of emergency and medicine. 2016; 34(4):730-734	non pertinent

2093	Sobell et al.,	2016	Acta dermato-venerologica. 2016; 96(4):514-520	non pertinent
2094	Smallwood et al.,	2016	Psychiatry Res Neuroimaging. 2016; 250:12-14	non pertinent
2095	R.B.R. sjz -	2016		non pertinent
2096	Sivalingam et al.,	2016	J of urology. 2016; 195(2):385-390	non pertinent
2097	Sinke et al.,	2016	Cortex: a j devoted to the study of the nervous system and behavior. 2016; 78:1-14	non pertinent
2098	Simpson et al.,	2016	Diabetes care. 2016; 39(9):1493-1500	non pertinent
2099	Simonelli et al.,	2016	Annals of oncology. 2016; 27(9):1782-1787	non pertinent
2100	Sidhu et al.,	2016	Liver international. 2016; 36(3):378-385	non pertinent
2101	Shukla et al.,	2016	International j of research in ayuverda and pharmacy. 2016; 7(2):8-11	non pertinent
2102	Shoushtarian et al.,	2016	J Clin Monit Comput. 2016; 30(6):833-844	non pertinent
2103	Shinozaki et al.,	2016	J Dent Res. 2016; 95(10):1138-1146	non pertinent
2104	Shimonovich et al.,	2016	BMC emergency medicine. 2016; 16(1):43	non pertinent
2105	Sheldon et al.,	2016	Vaccine. 2016; 34(18):2082-2091	non pertinent
2106	Shayan et al.,	2016	J of clinical and diagnostic research. 2016; 10(12):QC13-QC16	non pertinent
2107	Shavandi et al.,	2016	J of sports medicine and physical fitness. 2016; 56(9):990-996	non pertinent
2108	Sharon et al.,	2016	Am J Med. 2016; 129(7):755-758	non pertinent
2109	Sharma et al.,	2016	BMC musculoskeletal disorders. 2016; 17(1)	non pertinent

2110	Shalabi et al.,	2016	Blood. Conference: 58th annual meeting of the american society of hematology, ASH 2016. United states. Conference start:20161203. Conference end:20161206; 128(22)	non pertinent
2111	Shah et al.,	2016	J of rheumatology. 2016; 43(9):1665-1671	non pertinent
2112	Shah et al.,	2016	Alcoholism. Clinical and experimental research. Conference: 39th annual scientific meeting of the research society on alcoholism. New orleans, LA united states. Conference start: 20160625. Conference end:20160629. Conference publication: (var.pagings). 40:217A	non pertinent
2113	Serrano et al.,	2016	European j of anaesthesiology. 2016; 33(6):436-443	non pertinent
2114	Sermeus et al.,	2016	J canadien d'anesthesie (Canadian j of anaesthesia). 2016; 63(1):46-55	non pertinent
2115	Serinken et al.,	2016	Foot & ankle international / American Orthopaedic Foot and Ankle Society (and) Swiss Foot and Ankle Society. 2016; 37(9):989-993	non pertinent
2116	Segelov et al.,	2016	J of clinical oncology. 2016; 34(19):2258-2264	non pertinent
2117	Schwenk et al.,	2016	J of rehabilitation research and development. 2016; 53(6):945-958	non pertinent
2118	Schwenk et al.,	2016	Gerontology. 2016; 62(5):553-563	non pertinent

2119	Schweden et al.,	2016	J Anxiety Disord. 2016; 43:99-105	non pertinent
2120	Schwandt et al.,	2016	Alcoholism: clinical and experimental research. Conference: 39th annual scientific meeting of the research society on alcoholism. New orleans. LA united states. Conference start: 20160625. Conference end: 20160629. Conference publication: (var.pagings). 2016; 40:165A	non pertinent
2121	Schürmann et al.,	2016	AIDS (london, england). 2016; 30(1):57-63	non pertinent
2122	Schrubbe et al.,	2016	BMC musculoskeletal disorders. 2016; 17(1):359	non pertinent
2123	Schröder et al.,	2016	Neurourology and urodynamics. 2016; 35(5):582-588	non pertinent
2124	Schrag et al.,	2016	Obstetrics and gynecology. 2016; 127(2):199-201	non pertinent
2125	Schöffski et al.,	2016	Lancet (london, england). 2016; 387(10028):1629-1637	non pertinent
2126	Schmidt et al.,	2016	Alimentary pharmacology & therapeutics. 2016; 44(3):259-270	non pertinent
2127	Schloesser et al.,	2016	Neuropsychopharmacology. 2016; 41:555-556	non pertinent
2128	Schliessbach et al.,	2016	J of Pain. Conference: 35th Annual Scientific Meeting of the American Pain Society. Autin, TX United States. Conference Publication:(var.pagings). 2016 Apr; 17:(4 SUPPL. 1):78	non pertinent
2129	Schiopu et al.,	2016	Arthritis research & therapy. 2016; 18(1):131	non pertinent
2130	Schinhan et al.,	2016	Clin J Sport Med. 2016; 26(3):199-205	non pertinent

2131	Schaible et al.,	2016	Movement disorders. 2016; 31:410-	non pertinent
2132	Schadendorf et al.,	2016	Melanoma research Conference: 16th World Congress on Cancers oft he Skin 2016. Austria. Conference start: 20160831. Conference end: 20160903; 26:e43-e44	non pertinent
2133	Sbidian et al.,	2016	J of investigative dermatology. 2016; 136(6):1124-1129	non pertinent
2134	Saw et al.,	2016	BMC Musculoskelet Disord. 2016; 17:236	non pertinent
2135	Sarig Bahat et al.,	2016	Manual therapy. 2016; 25:e35-	non pertinent
2136	Santos et al.,	2016	J of acquired immune deficiency syndromes (1999). 2016; 72(1):21-30	non pertinent
2137	Samadani	2016	J of neurotrauma. 2016; 33:A4-	non pertinent
2138	Salem et al.,	2016	Gastroenterology. 2016; 151(6):1155-1563.e1152	non pertinent
2139	Saad et al.,	2016	Lancet oncology. 2016; 17(9):1306-1316	non pertinent
2140	Ryu et al.,	2016	Eur Spine J. 2016; 25(12):4067-4079	non pertinent
2141	Ruwe et al.,	2016	J of clinical phsychopharmacology. 2016; 36(5):457-464	non pertinent
2142	Ruperto et al.,	2016	Lancet (london, england). 2016; 387(10019):671-678	non pertinent
2143	Runge et al.,	2016	Regional anesthesia and pain medicine. 2016; 41(4):445-451	non pertinent
2144	Rummel et al.,	2016	The lancet. Oncology. 2016; 17(1):57-66	non pertinent
2145	Roy et al.,	2016	British j of radiology. 2016; 89(1062):20150966	non pertinent

2146	Rotter et al.,	2016	European spine j. conference: 11th german spine conference – annual meeting of the german spine society. Germany. Conference start: 20161201. Confernece end: 20161203. 2016; 25(11):3815	non pertinent
2147	Rossano et al.,	2016	Trials. 2016; 17(1):523	non pertinent
2148	Ross et al.,	2016	J of orthopaedic research. 2016; 34	non pertinent
2149	Rosenfeld et al.,	2016	Anaesthesia. 2016; 71(4):380-388	non pertinent
2150	Rogol et al.,	2016	Andrology. 2016; 4(1):46-54	non pertinent
2151	Robleda et al.,	2016	Intensive care medicine. 2016; 42(2):183-191	non pertinent
2152	Robertson et al.,	2016	Lancet (london, england). 2016; 388(10063):2997-3005	non pertinent
2153	Robbins et al.,	2016	Neurology. 2016; 87(24):2522-2526	non pertinent
2154	Rini et al.,	2016	Lancet oncology. 2016; 17(11):1599-1611	non pertinent
2155	Riel et al.,	2016	BMC musculoskeletal disorders. 2016; 17(1)	non pertinent
2156	Ricotti et al.,	2016	PLoS One. 2016; 11(4)	non pertinent
2157	Richeldi et al.,	2016	Respiratory medicine. 2016; 113:74-79	non pertinent
2158	Richards et al.,	2016	Osteoarthritis and cartilage. 2016; 24:470-	non pertinent
2159	Rheims et al.,	2016	Trials. 2016; 17(1):529	non pertinent
2160	Reisman et al.,	2016	Chest. Conference: CHEST 2016. United states. Conference start: 20161022. Conference end: 20161026. 2016; 150(4 Supplement 1):928A	non pertinent
2161	Reilingh et al.,	2016	American j of sports medicine. 2016; 44(5):1292-1300	non pertinent
2162	Rebbeck et al.,	2016	J of physiotherapy. 2016; 62(2):111	non pertinent

2163	Rauck et al.,	2016	Pain practice. 2016; 16:18	non pertinent
2164	Ratziu et al.,	2016	Gastroenterology. 2016; 150(5):1147-1159e1145	non pertinent
2165	Rashaan et al.,	2016	Trials. 2016; 17(1)	non pertinent
2166	Rao et al.,	2016	Headache. 2016; 56(2):331-340	non pertinent
2167	Rampurwala et al.,	2016	Investigational new drugs. 2016; 1-8	non pertinent
2168	Ramalho et al.,	2016	Lasers in medical science. 2016; 31(9):1899-1905	non pertinent
2169	Raje et al.,	2016	Clinical cancer research. 2016; 22(23):5688-5695	non pertinent
2170	Rajan et al.,	2016	J Neurosurg Anesthesiol. 2016; 28(4):282-290	non pertinent
2171	Raghavan et al.,	2016	BJOG. 2016; 123(1):120-127	non pertinent
2172	Raak et al.,	2016	Homeopathy. 2016; 105(1):37-38	non pertinent
2173	Quoix et al.,	2016	The lancet. Oncology. 2016; 17(2):212-223	non pertinent
2174	Quenardelle et al.,	2016	Cerebrovasc Dis. 2016; 42(3-4):205-212	non pertinent
2175	Qi et al.,	2016	Biological & pharmaceutical bulletin. 2016; 39(9):1455-1460	non pertinent
2176	Putzier et al.,	2016	Spine j. 2016; 16(7):811-819	non pertinent
2177	Pursnani et al.,	2016	American j of cardiology. 2016; 117(3):319-324	non pertinent
2178	Puiu et al.,	2016	Arthritis & rheumatology (hoboken, N.J.). 2016; 68(6):1511-1521	non pertinent
2179	Pricop et al.,	2016	International urology and nephrology. 2016; 48(2):183-189	non pertinent
2180	Preller et al.,	2016	Proc Natl Acad Sci U S A. 2016; 113(18):5119-5124	non pertinent
2181	Preece et al.,	2016	BMC Musculoskeletal Disord. 2016; 17(1):372	non pertinent
2182	Prawira et al.,	2016	Investigational new drugs. 2016; 34(5):575-583	non pertinent
2183	Prajna et al.,	2016	JAMA ophthalmology. 2016; 134(12):1365-1372	non pertinent

2184	Powers et al.,	2016	Contemporary clinical trials. 2016; 50:265-272	non pertinent
2185	Portincasa et al.,	2016	J of gastrointestinal and liver diseases. 2016; 25(2):151-157	non pertinent
2186	Popat et al.,	2016	The lancet haematology. 2016; 3(12):e572-e580	non pertinent
2187	Pons et al.,	2016	Annals of physical and rehabilitation medicine. 2016; 59:e162-	non pertinent
2188	Pinsornsak et al.,	2016	BMC musculoskeletal disorders. 2016; 17:313	non pertinent
2189	Pimenta et al.,	2016	Phytotherapy research:PTR. 2016; 30(4):613-617	non pertinent
2190	Piette et al.,	2016	JMIR research protocols. 5(2):e353.2016 apr	non pertinent
2191	Pieper et al.,	2016	J of the American Geriatrics Society. 2016; 64(2):261-269	non pertinent
2192	Pickut et al.,	2016	Movement disorders. 2016; 31:419-	non pertinent
2193	Pickering et al.,	2016	Drug design, development and therapy. 2016; 10:3969-3976	non pertinent
2194	Pichon et al.,	2016	International j of radiation oncology biology physics. 2016; 96(4):840-847	non pertinent
2195	Phan et al.,	2016	J of gastroenterology and hepatology (australia). 2016; 31:59-	non pertinent
2196	Phan et al.,	2016	Vaccine. 2016; 34(45):5449-5456	non pertinent
2197	Petschow et al.,	2016	PLoS One. 2016; 11(10)	non pertinent
2198	Pers et al.,	2016	Stem cells translational medicine. 2016; 5(7):847-856	non pertinent
2199	Perry et al.,	2016	Manual therapy. 2016; 25:e88-	non pertinent
2200	Pérol et al.,	2016	Lung cancer (Amsterdam, Netherlands). 2016; 93:95-103	non pertinent

2201	Perez et al.,	2016	Breast cancer research. 2016; 18(1)	non pertinent
2202	Per - Mateo	2016	Clinical trials peruvian registry – CTPR. 2016	non pertinent
2203	Pepin et al.,	2016	Human vaccines & immunotherapeutics. 2016; 12(12):3072-3078	non pertinent
2204	Peng et al.,	2016	Clinical therapeutics. 2016; 38(1//8147835 (NSFC) * National Natural Science Foundation of China*):192e192-201e192	non pertinent
2205	Pelletier et al.,	2016	Annals of the rheumatic diseases. 2016; 75:836-	non pertinent
2206	Pekyl et al.,	2016	New England j of medicine. 2016; 374(10):913-927	non pertinent
2207	Peker Tekdal et al.,	2016	Clinical oral implants research. 2016; 27(5):535-544	non pertinent
2208	Pedraza et al.,	2016	Int Orthop. 2016; 40(12):2519-2526	non pertinent
2209	Pazzaglia et al.,	2016	Neuroscience. 2016; 333:244-251	non pertinent
2210	Pawsey et al.,	2016	Drugs in R&D. 2016; 16(2):181-191	non pertinent
2211	Pathan et al.,	2016	Lancet (london, england). 2016; 387(10032):1999-2007	non pertinent
2212	Parratte et al.,	2016	Clinical orthopaedics and related research. 2016; 474(10):2085-2093	non pertinent
2213	Park et al.,	2016	The lancet. Oncology. 2016; 17(5):577-589	non pertinent
2214	Park et al.,	2016	Oncotarget. 2016; 7(46):75482-75491	non pertinent
2215	Park et al.,	2016	Uorl J. 2016; 13(4):2759-2764	non pertinent
2216	Park et al.,	2016	Pain practice. 2016; 16(7):814-819	non pertinent
2217	Paramarta et al.,	2016	J of translational medicine. 2016; 14(1):308	non pertinent

2218	Papa et al.,	2016	J of translational medicine. 2016; 14(1)	non pertinent
2219	Panza et al.,	2016	Med Sci Sports Exerc. 2016; 48(1):1-6	non pertinent
2220	Pandharipande et al.,	2016	Radiology. 2016; 281(3):835-846	non pertinent
2221	Paloyelis et al.,	2016	J Neuroendocrinol. 2016; 28(4)	non pertinent
2222	Palm et al.,	2016	Restor Neurol Neurosci. 2016; 34(2):189-199	non pertinent
2223	Pagidipati et al.,	2016	J of the American College of Cardiology. 2016; 67(22):2607-2616	non pertinent
2224	Ostlund-lagerstrom et al.,	2016	Nutr J. 2016; 15(1):80	non pertinent
2225	Ost	2016	Radiotherapy and oncology. 2016; 119:292-293	non pertinent
2226	Ormarsson et al.,	2016	Acta paediatrica. 2016; 105(6):689-694	non pertinent
2227	Orfila et al.,	2016	Stroke. Conference: american heart association/american stroke association 2016 international stroke conference and state-of-the-science stroke nursing symposium los angeles, CA united states. Conference start: 20160216. Conference end:20160219. Conference publication: (var.pagings). 47	non pertinent
2228	Orefice et al.,	2016	Neurotherapeutics. 2016; 13(2):428-438	non pertinent
2229	Olsen et al.,	2016	J of critical care. 2016; 36:207-211	non pertinent
2230	Ollivier et al.,	2016	Clinical orthopaedics and related research. 2016; 474(1):60-68	non pertinent

2231	Olesen et al.,	2016	Pancreatology. Conference: 48th annual meeting of the european pancreatic club, EPC 2016. Liverpool united kingdom. Conference start: 20160706. Conference end: 20160709. Conference publication: (var.pagings). 16(3 SUPPL 1):43-44	non pertinent
2232	Okusaka et al.,	2016	Cancer science. 2016; 107(12):1791-1799	non pertinent
2233	Okebe et al.,	2016	EBioMedicine. 2016; 13:348-355	non pertinent
2234	O'Shaughnessy et al.,	2016	Annals of oncology: official j of the european society for medical oncology. 2016; 27(1):106-113	non pertinent
2235	Ntr – Holewijn	2016	Netherlands Trial Registry. 2016	non pertinent
2236	Nowicka et al.,	2016	Biomed research international. 2016	non pertinent
2237	Notte et al.,	2016	Nursing. 2016; 46(2):17-23	non pertinent
2238	North et al.,	2016	Pain practice. 2016; 16(6):720-729	non pertinent
2239	Noguchi et al.,	2016	Clinical cancer research. 2016; 22(1):54-60	non pertinent
2240	Niv et al.,	2016	Nutrition & metabolism. 2016; 13(1)	non pertinent
2241	Nilsson et al.,	2016	Eur J Gastroenterol Hepatol. 2016; 28(5):514-524	non pertinent
2242	Nickel et al.,	2016	Pain practice. 2016; 16(7):820-830	non pertinent
2243	Nella et al.,	2016	J of clinical endocrinology and metabolism. 2016; 101(12):4690-4698	non pertinent
2244	Neijenhuis et al.,	2016	United european gastroenterology j. 2016; 4(5):A162-	non pertinent
2245	Nct – Scarlata	2016	National Library of Medicine. 2016	non pertinent

2246	Nct – Chi-Wen	2016	National Library of Medicine. 2016	non pertinent
2247	Nct – Han	2016	National Library of Medicine. 2016	non pertinent
2248	Nct – Grassi	2016	National Library of Medicine. 2016	non pertinent
2249	Nct – Hoirisch-Clapauch	2016	National Library of Medicine. 2016	non pertinent
2250	Nct – Geoffrion	2016	National Library of Medicine. 2016	non pertinent
2251	Nct – Bohm-Starke	2016	National Library of Medicine. 2016	non pertinent
2252	Nct – Miranda	2016	National Library of Medicine. 2016	non pertinent
2253	Nct – Zahran	2016	National Library of Medicine. 2016	non pertinent
2254	Nct – Oei	2016	National Library of Medicine. 2016	non pertinent
2255	Nct – Pelletier	2016	National Library of Medicine. 2016	non pertinent
2256	Nct – Arnold	2016	National Library of Medicine. 2016	non pertinent
2257	Nct – Rabinak	2016	National Library of Medicine. 2016	non pertinent
2258	Nct – Moro	2016	National Library of Medicine. 2016	non pertinent
2259	Nct – Michel-Cherqui	2016	National Library of Medicine. 2016	non pertinent
2260	Nct – Suhar	2016	National Library of Medicine. 2016	non pertinent
2261	Nct - Bailey	2016	National Library of Medicine. 2016	non pertinent
2262	Nct – Facina	2016	National Library of Medicine. 2016	non pertinent
2263	Nct – Bhardwaj	2016	National Library of Medicine. 2016	non pertinent
2264	Nct - Nazarini	2016	National Library of Medicine. 2016	non pertinent
2265	Nct - Waubant	2016	National Library of Medicine. 2016	non pertinent
2266	Nct - Miller	2016	National Library of Medicine. 2016	non pertinent
2267	Nct – Hall	2016	National Library of Medicine. 2016	non pertinent
2268	Nct – Mahajan	2016	National Library of Medicine. 2016	non pertinent
2269	Nct - Jia	2016	National Library of Medicine. 2016	non pertinent
2270	Nct – Zhang	2016	National Library of Medicine. 2016	non pertinent
2271	Nct – Chrisman	2016	National Library of Medicine. 2016	non pertinent
2272	Nct – Sussman	2016	National Library of Medicine. 2016	non pertinent
2273	Nct – Liechti	2016	National Library of Medicine. 2016	non pertinent
2274	Nct – Karatosun	2016	National Library of Medicine. 2016	non pertinent
2275	Nct – Nieves	2016	National Library of Medicine. 2016	non pertinent
2276	Nct – Namrawy	2016	National Library of Medicine. 2016	non pertinent
2277	Nct - Stavropoulou	2016	National Library of Medicine. 2016	non pertinent
2278	Nct – Tasian	2016	National Library of Medicine. 2016	non pertinent

2279	Nct – Beidel	2016	National Library of Medicine. 2016	non pertinent
2280	Nct – Paul et al.,	2016	BMC Musculoskelet Disord. 2016 Aug; 17(1):360	non pertinent
2281	Nct – Hughes	2016	National Library of Medicine. 2016	non pertinent
2282	Nct – Bond	2016	National Library of Medicine. 2016	non pertinent
2283	Nct – Oh et al.,	2016	Am J Sports Med. 2018 Mar; 46(3):679-686	non pertinent
2284	Nct – Sheffield Hallam University	2016	National Library of Medicine. 2016	non pertinent
2285	Nct – Cheung	2016	National Library of Medicine. 2016	non pertinent
2286	Nct – Cristiana Care Health Services	2016	National Library of Medicine. 2016	non pertinent
2287	Nct – Hillis-Trupe	2016	National Library of Medicine. 2016	non pertinent
2288	Nct – Germanotta et al.,	2016	J Neuroeng Rehabil. 2018 May; 15(1):39	non pertinent
2289	Nct - Kvarstein	2016	National Library of Medicine. 2016	non pertinent
2290	Nct - Spies	2016	National Library of Medicine. 2016	non pertinent
2291	Nct – Apkarian	2016	National Library of Medicine. 2016	non pertinent
2292	Nct – University Hospital, Montpellier	2016	National Library of Medicine. 2016	non pertinent
2293	Nct – Roitman	2016	National Library of Medicine. 2016	non pertinent
2294	Nct - Heapy	2016	National Library of Medicine. 2016	non pertinent
2295	Nct – Barrera	2016	National Library of Medicine. 2016	non pertinent
2296	Nct – Crenshaw	2016	National Library of Medicine. 2016	non pertinent
2297	Nct – Jensen	2016	National Library of Medicine. 2016	non pertinent
2298	Nct – Madan	2016	National Library of Medicine. 2016	non pertinent
2299	Nct – Sanzo	2016	National Library of Medicine. 2016	non pertinent
2300	Nct – Liu-Ambrose	2016	National Library of Medicine. 2016	non pertinent
2301	Nct – Schuman-Olivier	2016	National Library of Medicine. 2016	non pertinent
2302	Nct - Putman	2016	National Library of Medicine. 2016	non pertinent
2303	Nct - Kvarstein	2016	National Library of Medicine. 2016	non pertinent
2304	Nct - Williams	2016	National Library of Medicine. 2016	non pertinent
2305	Nan et al.,	2016	J of neurogastroenterology and motility. 2016; 22(1):118-128	non pertinent

2306	Nalamachu et al.,	2016	Current medical research and opinion. 2016; 32(7):1311-1317	non pertinent
2307	Naik et al.,	2016	Anesthesia and Analgesia. 2016; 122(5):1646-1653	non pertinent
2308	Myhre et al.,	2016	Anesthesiology. 2016; 124(1):141-149	non pertinent
2309	Murugaiyan et al.,	2016	Annals of indian academy of neurology. 2016; 19(6):109-	non pertinent
2310	Muresanu et al.,	2016	Stroke: a j of cerebral circulation. 2016; 47(1):151-159	non pertinent
2311	Mulvenna et al.,	2016	Lancet. 2016; 388(10055):2004-2014	non pertinent
2312	Mueller et al.,	2016	Experimental dermatology. Conference: 2nd inflammatory skin disease summit. United states. 2016; 25:27	non pertinent
2313	Mu et al.,	2016	Clinical rheumatology. 2016; 35(1):165-173	non pertinent
2314	Mourad et al.,	2016	European j of cardiovascular nursing. 2016; 15:44-	non pertinent
2315	Moulton et al.,	2016	Radiation oncology (london, england). 2016; 11(1)	non pertinent
2316	Mosquera et al.,	2016	Cancer genetics. 2016; 209(12):567-581	non pertinent
2317	Mosaffa-Jahromi et al.,	2016	J of ethnopharmacology. 2016; 194:937-946	non pertinent
2318	Moryl et al.,	2016	J of opioid management. 2016; 12(1):47-55	non pertinent
2319	Morrow et al.,	2016	Molecular therapy – oncolytics. 2016; 3	non pertinent
2320	Morrissey et al.,	2016	Manual therapy. 2016; 25:e107-e108	non pertinent
2321	Morin et al.,	2016	J of sexual medicine. 2016; 13(6):243-	non pertinent
2322	Morin et al.,	2016	Trials. 2016; 17(1)	non pertinent
2323	Morel et al.,	2016	PLoS One. 2016; 11(4)	non pertinent

2324	Moore et al.,	2016	BMC anesthesiology. 2016; 16(1)	non pertinent
2325	Mooney et al.,	2016	Clinical pharmacology and therapeutics. 2016; 100(4):344-352	non pertinent
2326	Moeen	2016	Paediatric anaesthesia. 2016; 26(8):807-814	non pertinent
2327	Miyazaki et al.,	2016	Methods in pharmacology and toxicology. 2016; 37-52	non pertinent
2328	Miyazaki et al.,	2016	Cardiovascular and interventional radiology. 2016; 39(10):1464-1470	non pertinent
2329	Miskowiak et al.,	2016	Neuropsychopharmacology. 2016; 41:361-	non pertinent
2330	Mischkowski et al.,	2016	Soc Cogn Affect Neurosci. 2016; 11(9):1345-1353	non pertinent
2331	Mir et al.,	2016	The lancet. Oncology. 2016; 17(5):632-641	non pertinent
2332	Mir et al.,	2016	Lancet oncology. 2016; 17(12):1732-1742	non pertinent
2333	Miller et al.,	2016	J of clinical endocrinology and metabolism. 2016; 101(8):3163-3170	non pertinent
2334	Meyers et al.,	2016	Clinical pharmacology in drug development. 2016; 5(6):450-459	non pertinent
2335	Metyas et al.,	2016	Arthrithis and rheumatology. Conference: american college of rheumatology/association of rheumatology health professionals annual scientific meeting, ACR/ARHP 2016. United states. Conference start:20161111. Conference end: 20161116; 68:64-65	non pertinent

2336	Menzaghi et al.,	2016	Postgraduate medicine. Conference: 2016 pain week conference. United states. Conference start: 20160906. Conference end: 20160910; 128:86-87	non pertinent
2337	Mendis et al.,	2016	J Sci Med Sport. 2016; 19(11):866-871	non pertinent
2338	Mendell et al.,	2016	Annals of neurology. 2016; 79(2):257-271	non pertinent
2339	Meissner et al.,	2016	Obstet Gynecol. 2016; 128(5):1134-1142	non pertinent
2340	Meisel et al.,	2016	European j of cancer (Oxford, Enland : 1990). 2016; 56:93-100	non pertinent
2341	McMurdo et al.,	2016	Arthritis care & research. 2016; 68(5):716-721	non pertinent
2342	McLeod et al.,	2016	Brain injury. 2016; 30(5-6):635-	non pertinent
2343	McGowan et al.,	2016	PLoS One. 2016; 11(6)	non pertinent
2344	McEneny et al.,	2016	Atherosclerosis. 2016; 244:172-178	non pertinent
2345	McCormick et al.,	2016	PM and r. 2016; 8(11):1039-1045	non pertinent
2346	McClelland et al.,	2016	PLoS One. 2016; 11(3):e0148606	non pertinent
2347	McBain et al.,	2016	Annals of the rheumatic diseases. 2016; 75(7):1343-1349	non pertinent
2348	Mazzini et al.,	2016	Amyotropic lateral sclerosis and frontotemporal degeneration. Conference: 27th international symposium an ALS/MND. Ireland. Conference start: 20161207. Conference end: 20161209; 17:243	non pertinent
2349	Mayr et al.,	2016	British j of anaesthesia. 2016; 116(1):90-99	non pertinent
2350	Mauermann et al.,	2016	Anesthesiology. 2016; 124(2):453-463	non pertinent
2351	Mathur et al.,	2016	Indian j of dental research. 2016; 27(6):623-629	non pertinent

2352	Mathieson et al.,	2016	Trials. 2016; 17(1)	non pertinent
2353	Mathialagan et al.,	2016	Oral oncology. 2016; 61:142-145	non pertinent
2354	Massé-Alarie et al.,	2016	Scandinavian j of pain. 2016; 12:74-83	non pertinent
2355	Masman et al.,	2016	J Pain Symptom Manage. 2016; 52(2):212-220.e213	non pertinent
2356	Martin et al.,	2016	Drugs in r and d. 2016; 16(1):81-92	non pertinent
2357	Mariano et al.,	2016	Pain Med. 2016; 17(4):737-745	non pertinent
2358	Manvar et al.,	2016	Regional anesthesia and pain medicine. 2016; 41(5)	non pertinent
2359	Manning et al.,	2016	Academic emergency medicine. 2016; 23(7):766-771	non pertinent
2360	Mangas-Sanjuan et al.,	2016	British j of clincial pharmacology. 2016; 82(1):92-107	non pertinent
2361	Malavera et al.,	2016	J Pain. 2016; 17(8):911-918	non pertinent
2362	Malagaria et al.,	2016	Cardiovascular and interventional radiology. 2016; 39(10):1379-1391	non pertinent
2363	Maksymowych et al.,	2016	J of rheumatology. 2016; 43(6):1232-1233	non pertinent
2364	Majumder et al.,	2016	European j of cancer (Oxford, England : 1990). 2016; 65:43-51	non pertinent
2365	Mai et al.,	2016	Guangming j of chinese medicine (guang ming zhong yi). 2016; 31(15):2226-2229	non pertinent
2366	Mahshidfar et al.	2016	Indian j of critical care medicine. 2016; 20(8):465-468	non pertinent

2367	Mahler et al.,	2016	Allergy: european j of allergy and clinical immunology. Conference: 35th Annual Congress of the European Academy of Allergy and Clinical Immunology, EAACI 2016. Austria. Conference start: 20160611. Conference end: 20160615; 71:298	non pertinent
2368	Mahipal et al.,	2016	Cancer chemotherapy and pharmacology. 2016; 78(1):157-165	non pertinent
2369	Maheu et al.,	2016	Rheumatologia. 2016; 30(2):19-32	non pertinent
2370	Mahabir et al.,	2016	J of affective disorders. 2016; 192:98-103	non pertinent
2371	Madro et al.,	2016	Anaesthesiol Intensive Ther. 2016; 48(4):234-238	non pertinent
2372	Madan et al.,	2016	BJU international. 2016; 118(4):590-597	non pertinent
2373	Machado et al.,	2016	Toxins (Basel). 2016; 8(12)	non pertinent
2374	Ma et al.,	2016	Neuroimage. 2016; 135:186-196	non pertinent
2375	Ma et al.,	2016	Zhonguo xiu fu chong jian wai ke za zhi (Chinese j of reparative and reconstructive surgery). 2016; 30(3):328-335	non pertinent
2376	Lyu et al.,	2016	Frontiers in behavioral neuroscience. 2016; 10(OCT)	non pertinent
2377	Lyu et al.,	2016	Brain impairment. 2016; 17(3):286-287	non pertinent
2378	Luongo et al.,	2016	Biomed research international. 2016	non pertinent
2379	Luo et al.,	2016	Research and reports in urology. 2016; 8:217-224	non pertinent
2380	Lulla et al.,	2016	Neuro-oncology. 2016; 18(9):1319-1325	non pertinent
2381	Lugo et al.,	2016	Nutrition j. 2016; 15:14	non pertinent

2382	Luder et al.,	2016	Annals of the rheumatic diseases. 2016; 75:1308-	non pertinent
2383	Ludbrook et al.,	2016	British j of dermatology. 2016; 174(5):985-995	non pertinent
2384	Lu et al.,	2016	PLoS One. 2016; 11(6)	non pertinent
2385	Louca Jounger et al.,	2016	PLoS One. 2016; 11(12):e0168703	non pertinent
2386	Lou et al.,	2016	Clinical otolaryngology. 2016; 41(6):744-749	non pertinent
2387	LoRusso et al.,	2016	Clinical cancer research. 2016; 22(13):3227-3237	non pertinent
2388	Loret et al.,	2016	Retrovirology. 2016; 13(1)	non pertinent
2389	Lohle et al.,	2016	Cardiovascular and interventional radiology. 2016; 39(3):123-124	non pertinent
2390	Liu et al.,	2016	Basic & clinical pharmacology & toxicology. 2016; 119(1):93-100	non pertinent
2391	Lipton et al.,	2016	The lancet. Oncology. 2016; 17(5):612-621	non pertinent
2392	Liou et al.,	2016	Gut. 2016; 65(11):1784-1792	non pertinent
2393	Liou et al.,	2016	American j of gastroenterology. 2016; 111(3):381-387	non pertinent
2394	Lin et al.,	2016	Medicine (united states). 2016; 95(34)	non pertinent
2395	Lin et al.,	2016	Canadian j of emergency medicine. 2016; 18(4):315-318	non pertinent
2396	Lin et al.,	2016	Sci Rep. 2016; 6:37816	non pertinent
2397	Lin et al.,	2016	BMC musculoskeletal disorders. 2016; 17(1):374	non pertinent
2398	Limsrivilai et al.,	2016	American j of gastroenterology. 2016; 111(2):217-224	non pertinent
2399	Lima et al.,	2016	Arthritis care & research. 2016; 68(1):91-98	non pertinent
2400	Liang et al.,	2016	Psychiatry research. 2016; 240:80-87	non pertinent

2401	Li et al.,	2016	Stroke. Conference: american heart association/american stroke association 2016 international stroke conference and state-of-the-angeles. CA united states. Conference start: 20160216. Conference end: 20160219. Conference publication: (var.pagings); 47	non pertinent
2402	Lin et al.,	2016	BMC anesthesiology. 2016; 16:24	non pertinent
2403	Lin et al.,	2016	Medicine (united states). 2016; 95(15)	non pertinent
2404	LeWitt et al.,	2016	Movement disorders. 2016; 31(9):1356-1365	non pertinent
2405	Lewis et al.,	2016	PLoS One. 2016; 11(4)	non pertinent
2406	Lewis et al.,	2016	Alcohol Clin Exp Res. 2016; 40(12):2519-2527	non pertinent
2407	Levy-Cooperman et al.,	2016	Epilepsy & behavior. 2016; 61:63-71	non pertinent
2408	Leung et al.,	2016	Trials. 2016; 17(1):421	non pertinent
2409	Lelic et al.,	2016	Eur J Neurosci. 2016; 44(11):2966-2974	non pertinent
2410	Lekander et al.,	2016	Brain Behav Immun. 2016; 56:34-41	non pertinent
2411	Lei et al.,	2016	Beneficial microbes. 2016; 7(5):631-637	non pertinent
2412	Lee et al.,	2016	Z Orthop Unfall. 2016; 154(6):571-577	non pertinent
2413	Lee et al.,	2016	J of rheumatology. 2016; 43(1):38-45	non pertinent
2414	Lee et al.,	2016	Clinical therapeutics. 2016; 38(3):557-573	non pertinent
2415	Lee et al.,	2016	Dermatologic therapy. 2016; 29(3):184-190	non pertinent

2416	Ledson et al.,	2016	Thorax. Conference: british thoracic society winter meeting 2016. United kingdom; 71:A138-A139	non pertinent
2417	Leblanc et al.,	2016	Anaesth Crit Care Pain Med. 2016; 35(2):109-114	non pertinent
2418	Leasure et al.,	2016	Ochsner j. 2016; 16(1):e5-e6	non pertinent
2419	Lazarus et al.,	2016	PLoS One. 2016; 11(11)	non pertinent
2420	Lauche et al.,	2016	Complementary therapies in medicine. 2016; 26:171-177	non pertinent
2421	Larsson et al.,	2016	Radiation protection dosimetry. 2016; 169(1-4):123-129	non pertinent
2422	Laque et al.,	2016	Clinical Neurophysiology. 2016; 127(3):e9-	non pertinent
2423	Lambertini et al.,	2016	Supportive care in cancer. 2016; 24(3):1285-1294	non pertinent
2424	Lam et al.,	2016	Gut. 2016; 65(1):91-99	non pertinent
2425	Labuschagne et al.,	2016	J of neurology, neurosurgery and psychiatry. 2016; 87:A103-	non pertinent
2426	Labrenz et al.,	2016	Neuroimage. 2016; 130:104-114	non pertinent
2427	Kunz et al.,	2016	J of the european academy of dermatology and venereology : JEADV. 2016; 30(2):293-298	non pertinent
2428	Kunoe et al.,	2016	BMC pharmacology and toxicology. 2016; 17(1)	non pertinent
2429	Kumar et al.,	2016	Blood. Confernece: 58th annual meeting of the american society of hematology. ASH 2016. United states. Conference start: 20161203. Conference end: 20161206; 128(22)	non pertinent
2430	Kudiganti et al.,	2016	Lipids in health and disease. 2016; 15(1)	non pertinent
2431	Kucyi et al.,	2016	Pain. 2016; 157(9):1895-1904	non pertinent

2432	Krumpl et al.,	2016	European j of pharmaceutical sciences. 2016; 92:64-73	non pertinent
2433	Kroes et al.,	2016	Neuropsychopharmacology. 2016; 41(6):1569-1578	non pertinent
2434	Kovanen et al.,	2016	European addiction research. 2016; 22(2):70-79	non pertinent
2435	Kosek et al.,	2016	Brain Behav Immun. 2016; 58:218-227	non pertinent
2436	Koh et al.,	2016	Anaesthesia. 2016; 71(6):692-699	non pertinent
2437	Koch et al.,	2016	Neuropsychopharmacology. 2016; 41(6):1495-1504	non pertinent
2438	Koch et al.,	2016	Neuropsychopharmacology. 2016; 41(8):2041-2051	non pertinent
2439	Knoop	2016	Psycho-oncology. 2016; 25:119-121	non pertinent
2440	Kilic et al.,	2016	Saudi medical j. 2016; 37(11):1191-1195	non pertinent
2441	Kittipeerachon et al.,	2016	Schizophrenia research. 2016; 176(2-3):231-238	non pertinent
2442	Kise et al.,	2016	Bmj. 2016; 354:i3740	non pertinent
2443	King et al.,	2016	Frontiers in Psychiatry. 2016; 7(SEP)	non pertinent
2444	Kimura et al.,	2016	International j of urology. 2016; 23(5):395-403	non pertinent
2445	Kimura et al.,	2016	BMJ Open. 2016; 6(9):e010999	non pertinent
2446	Kim et al.,	2016	Yonsei medical j. 2016; 57(2):490-495	non pertinent
2447	Kim et al.,	2016	Blood. Conference: 58th annual meeting of the american society of hematology. ASH 2016. United states. Conference start: 20161203. Conference end: 20161206; 128(22)	non pertinent
2448	Kim et al.,	2016	European spine j. 2016; 25(5):1614-1619	non pertinent
2449	Kim et al.,	2016	Clinical orthopaedics and related research. 2016; 474(3):776-783	non pertinent

2450	Kim et al.,	2016	Medicine. 2016; 95(18):e3593	non pertinent
2451	Kim et al.,	2016	Yonsei Med J. 2016; 57(4):980-986	non pertinent
2452	Kim et al.,	2016	CNS spectrums. 2016; 21(6):466-477	non pertinent
2453	Khurana et al.,	2016	J of cancer research and therapeutics. 2016; 12(6):69-	non pertinent
2454	Khasnavis et al.,	2016	Molecular genetics and metabolism. 2016; 118(3):160-166	non pertinent
2455	Khalil et al.,	2016	J of clinical anesthesia. 2016; 32:242-247	non pertinent
2456	Khalil et al.,	2016	Clinical cancer research. 2016; 22(21):5204-5210	non pertinent
2457	Khachatryan et al.,	2016	New armenian medical j. 2016; 10(3):11-16	non pertinent
2458	Kerbrat et al.,	2016	N Engl J Med. 2016; 375(18):1717-1725	non pertinent
2459	Keipert	2016	Advances in experimental medicine and biology. 2016; 923:23-29	non pertinent
2460	Keefe et al.,	2016	Annals of oncology. 2016; 27(8):1579-1585	non pertinent
2461	Kearns et al.,	2016	Anaesthesia. 2016; 71(12):1431-1440	non pertinent
2462	Kct - Yoonhee	2016	Clinical Research Information Service – CRIS. 2016	non pertinent
2463	Kazandijan et al.,	2016	Oncologist. 2016; 21(5):634-642	non pertinent
2464	Kawai et al.,	2016	Japanese j of clinical oncology. 2016; 46(3):248-253	non pertinent
2465	Kasang et al.,	2016	PLoS One. 2016; 11(1)	non pertinent
2466	Karamanolis et al.,	2016	Arthritis research & therapy. 2016; 18(1)	non pertinent
2467	Kaput et al.,	2016	J of General Internal Medicine. 2016; 31(2):661-	non pertinent
2468	Kangovi et al.,	2016	J of General Internal Medicine. 2016; 31(2):173-174	non pertinent

2469	Kang et al.,	2016	Cancer research and treatment. 2016; 48(2):458-464	non pertinent
2470	Jung et al.,	2016	BMJ Open. 2016; 6(11)	non pertinent
2471	Juel et al.,	2016	Pain Pract. 2016; 16(6):669-679	non pertinent
2472	Jprn, Umin – Tetsuaki	2016	UMIN-CTR Clinical Trial. 2016	non pertinent
2473	Jprn, Umin - Nakamura	2016	UMIN-CTR Clinical Trial. 2016	non pertinent
2474	Jprn, Umin – Shimizu	2016	UMIN-CTR Clinical Trial. 2016	non pertinent
2475	Jprn, Umin – Shin-ichi konno	2016	UMIN-CTR Clinical Trial. 2016	non pertinent
2476	Jounger et al.,	2016	PLoS One. 2016; 11(12)	non pertinent
2477	Jones et al.,	2016	Pain and therapy. 2016; 5(1):107-113	non pertinent
2478	Jones et al.,	2016	Dermatologic surgery. 2016; 42(1):38-49	non pertinent
2479	Johansson	2016	Acta orthopaedica. 2016; 87(1):79-82	non pertinent
2480	Johannesson et al.,	2016	J of lower genital tract disease. 2016; 20(4):6-8	non pertinent
2481	Jia et al.,	2016	Zhongguo xiu fu chong jian wai ke za zhi (Chinese j of reparative and reconstructive surgery). 2016; 30(3):328-335	non pertinent
2482	Jensen et al.,	2016	Int J Clin Exp Hypn. 2016; 64(1):1-23	non pertinent
2483	Janum et al.,	2016	Innate immunity. 2016; 22(7):546-556	non pertinent
2484	Janssens et al.,	2016	Clinical biomechanics (Bristol, Avon). 2016; 32:40-48	non pertinent
2485	Janssens et al.,	2016	Radiotherapy and oncology. 2016; 119(2):213-220	non pertinent

2486	Janin	2016	Foot and ankle surgery. Conference: foot international 2016 congress. Berlin germany. Conference start: 20160623. Conference end: 20160625. Conference publication: (var.pagings); 22(2 SUPPL 1):18	non pertinent
2487	Jamshed et al.,	2016	Vaccine. 2016; 34(5):630-635	non pertinent
2488	James et al.,	2016	Health technology assessment. 2016; 20(53):1-127	non pertinent
2489	Jafarpour et al.,	2016	J of ethnopharmacology. 2016; 179:170-176	non pertinent
2490	Jafarinia et al.,	2016	J of affective disorders. 2016; 204:1-8	non pertinent
2491	Jacobs et al.,	2016	Breast cancer research and treatment. 2016; 155(1):77-84	non pertinent
2492	Jackson et al.,	2016	Heart (British Cardiac Society). 2016; 102:A95-	non pertinent
2493	Izurieta et al.,	2016	Pediatric infectious disease j. 2016; 35(2):e35-e47	non pertinent
2494	Isrctn – Linni	2016	ISRCTN registry. 2016	non pertinent
2495	Isrctn – Tschugg	2016	ISRCTN registry. 2016	non pertinent
2496	Isrctn – Gordon	2016	ISRCTN registry. 2016	non pertinent
2497	Isrctn – Foster	2016	ISRCTN registry. 2016	non pertinent
2498	Isrctn – Gliner	2016	ISRCTN registry. 2016	non pertinent
2499	Isrctn – Bhattacharyya	2016	ISRCTN registry. 2016	non pertinent
2500	Isrctn - Stathi	2016	ISRCTN registry. 2016	non pertinent
2501	Isabelle et al.,	2016	J of clinical oncology. 2016; 34(36):4345-4353	non pertinent
2502	Ironside et al.,	2016	Biol Psychiatry. 2016; 79(10):823-830	non pertinent
2503	Irct2016092125067N – Hosieni	2016	Iranian Registry of Clinical Trials – IRCT. 2016	non pertinent
2504	Irct2016022926846N - Ghanbarinia	2016	Iranian Registry of Clinical Trials – IRCT. 2016	non pertinent
2505	Irct2015111625067N – Hosieni	2016	Iranian Registry of Clinical Trials – IRCT. 2016	non pertinent

2506	Irct201512194443 N – Zakerimoghadam	2016	Iranian Registry of Clinical Trials – IRCT. 2016	non pertinent
2507	Isrct20150110106 1N	2016	Iranian Registry of Clinical Trials – IRCT. 2016	non pertinent
2508	Iosifidis et al.,	2016	Surgical and radiological anatomy. 2016; 38(1):33	non pertinent
2509	Ickmans et al.,	2016	J Physiother. 2016; 62(3):165	non pertinent
2510	Ichesco et al.,	2016	Arthritis and rheumatology. Conference: american college of rheumatology/association of rheumatology health professionals annual scientific meeting. ACR/ARHP 2016. United states. Conference start: 20161111. Conference end:20161116; 68:54-55	non pertinent
2511	Hussain et al.,	2016	Clinical cancer research. 2016; 22(13):3192-3200	non pertinent
2512	Hurlemann et al.,	2016	Neuropsychopharmacology. 2016; 41:333-	non pertinent
2513	Hung et al.,	2016	The lancet. Infectious diseases. 2016; 16(2):209-218	non pertinent
2514	Hughes-Austin et al.,	2016	Arthritis and rheumatology. Conference: american college of rheumatology/association of rheumatology health professionals annual scientific meeting. ACR/ARHP 2016. United states. Conference start: 20161111. Conference end: 20161116. 68:10-11	non pertinent
2515	Huang et al.,	2016	Oral Dis. 2016; 22(8):766-774	non pertinent

2516	Hsieh et al.,	2016	PM & r : the j of injury, function, and rehabilitation. 2016; 8(12):1127-1135	non pertinent
2517	Hopulele-Petri et al.,	2016	European neuropsychopharmacology. 2016; 26:416-	non pertinent
2518	Holmes et al.,	2016	J of pediatrics. 2016; 169:128-134e121	non pertinent
2519	Holm et al.,	2016	Eur J Pain. 2016; 20(4):626-638	non pertinent
2520	Hofmann et al.,	2016	Palliative medicine. 2016; 30(6):NP79-NP80	non pertinent
2521	Hofmann et al.,	2016	J of opioid management. 2016; 12(5):323-331	non pertinent
2522	Hmamouchi et al.,	2016	Clinical and experimental rheumatology. 2016; 34(3):396-403	non pertinent
2523	Hillila et al.,	2016	Scandinavian j of gastroenterology. 2016; 51(1):16-21	non pertinent
2524	Hill et al.,	2016	Annals of the rheumatic diseases. 2016; 75(1):23-29	non pertinent
2525	Higurashi et al.,	2016	The lancet. Oncology. 2016; 17(4):475-483	non pertinent
2526	Higashikawa et al.,	2016	European j of clinical nutrition. 2016; 70(5):582-587	non pertinent
2527	Hickey et al.,	2016	Breast cancer research and treatment. 2016; 158(1):79-90	non pertinent
2528	Hickey et al.,	2016	Malaria j. 2016; 15(1)	non pertinent
2529	Hermanowicz et al.,	2016	CNS Drugs. 2016; 30(5):443-454	non pertinent
2530	Heo et al.,	2016	Korean J Intern Med. 2016; 31(2):260-266	non pertinent
2531	Henriquez et al.,	2016	Manual therapy. Conference: IFOMPT 2016 conference. United kingdom. Conference start: 20160704. Conference end: 20160708; 25:e103-e104	non pertinent
2532	Henriksen et al.,	2016	Osteoarthritis and cartilage. 2016; 24:437-	non pertinent

2533	Hendren et al.,	2016	J of child and adolescent psychopharmacology. 2016; 26(9):774-783	non pertinent
2534	Henckens et al.,	2016	Social cognitive and effective neuroscience. 2016; 11(4):663-673	non pertinent
2535	Heapy et al.,	2016	BMC musculoskeletal disorders. 2016 Feb; 17:85	non pertinent
2536	Healey et al.,	2016	BMJ Open. 2016; 6(9)	non pertinent
2537	Hayamizu et al.,	2016	Neuroimage. 2016; 130:175-183	non pertinent
2538	Hassan et al.,	2016	European spine j. 2016; 25(4):1056-1063	non pertinent
2539	Hasanpour Dehkordi	2016	J Sports Med Phys Fitness. 2016; 56(11):1417-1422	non pertinent
2540	Hasanain et al.,	2016	Brazilian j of infectious diseases. 2016; 20(3):250-254	non pertinent
2541	Hartling et al.,	2016	Clin Psychol Psychother. 2016; 23(6):509-522	non pertinent
2542	Harte et al.,	2016	Pain. 2016; 157(9):1933-1945	non pertinent
2543	Harrison et al.,	2016	Alimentary pharmacology & therapeutics. 2016; 44(11-12):1183-1198	non pertinent
2544	Harris et al.,	2016	JAMS j of acupuncture and meridian studies. 2016; 9(4):346-	non pertinent
2545	Harrington et al.,	2016	Oncotargets and therapy. 2016; 9:7081-7093	non pertinent
2546	Harbeck et al.,	2016	Annals of oncology : official j of the european society for medical oncology. 2016; 27(6):1047-1054	non pertinent
2547	Hanling et al.,	2016	Regional anesthesia and pain medicine. 2016; 41(4):494-500	non pertinent
2548	Haney et al.,	2016	Neuropsychopharmacology. 2016; 41(8):1974-1982	non pertinent
2549	Halvorsen et al.,	2016	Manual therapy. 2016; 25:e72-	non pertinent

2550	Hakim et al.,	2016	J Extra Corpor Technol. 2016; 48(4):173-178	non pertinent
2551	Hachulla et al.,	2016	Annals of the rheumatic diseases. 2016; 75(6):1009-1015	non pertinent
2552	Habiger et al.,	2016	Behavioural neurology. 2016:7036415	non pertinent
2553	Gurler et al.,	2016	Minerva stomatologica. 2016; 65(4):207-212	non pertinent
2554	Gupta et al.,	2016	Pain Physician. 2016; 19(6):349-356	non pertinent
2555	Gu et al.,	2016	Drug design, development and therapy. 2016; 10:1525-1531	non pertinent
2556	Grimm et al.,	2016	European neuropsychopharmacology. 2016; 26:218-	non pertinent
2557	Griggs et al.,	2016	Neurology. 2016; 87(20):2123-2131	non pertinent
2558	Greenway et al.,	2016	Clinical obesity. 2016; 6(5):285-295	non pertinent
2559	Greenberg et al.,	2016	PLoS One. 2016; 11(6)	non pertinent
2560	Gray et al.,	2016	Trials. 2016; 17(1):579	non pertinent
2561	Gras	2016	Drugs of today (barcelona, spain : 1998). 2016; 52(10):543-550	non pertinent
2562	Grant et al.,	2016	Pediatr Crit Care Med. 2016; 17(12):1131-1141	non pertinent
2563	Gozzi et al.,	2016	Neuropsychopharmacology. 2016	non pertinent
2564	Gowin et al.,	2016	Alcohol Clin Exp Res. 2016; 40(5):979-987	non pertinent
2565	Gowin et al.,	2016	Alcoholism: clinical and experimental research. Conference: 39th annual scientific meeting of the research society on alcoholism. New orleans. LA united states. Conference start: 20160625. Conference end: 20160629. Conference publication (var.pagings); 40:263A	non pertinent

2566	Gougeon et al.,	2016	Clin J Pain. 2016; 32(3):238-245	non pertinent
2567	Gorka et al.,	2016	Neuropsychophar macology. 2016; 41(7):1888-1896	non pertinent
2568	Goren et al.,	2016	Dermatologic therapy. 2016; 29(3):160-163	non pertinent
2569	Gordon et al.,	2016	Annals of emergency medicine. Conference: american college of emergency physicians. ACEP 2016research forum. United states; 68(4 Supplement 1):47-	non pertinent
2570	Goossens et al.,	2016	European j of cancer. 2016; 69:9-18	non pertinent
2571	Goody et al.,	2016	International j of radiation oncology biology physics. 2016; 96(5):994-1002	non pertinent
2572	Goncalves et al.,	2016	J of neurology, neurosurgery and psychiatry. 2016; 87(10):1127-1132	non pertinent
2573	Gomez et al.,	2016	Lancet oncology. 2016; 17(12):1672-1682	non pertinent
2574	Goldwater et al.,	2016	Clinical pharmacology: advances and applications. 2016; 8:203-212	non pertinent
2575	Goldstein et al.,	2016	Neuropsychophar macology. Confernece: 55th annual meeting of the american college of neuropsychophar macology. ACNP 2016. United states. Conference start: 20161204. Conference end: 20161208; 41:373	non pertinent
2576	Goksen et al.,	2016	Eur J Phys Rehabil Med. 2016; 52(4):431-439	non pertinent
2577	Goh et al.,	2016	PLoS medicine. 2016; 13(12)	non pertinent
2578	Goettel et al.,	2016	BMC Anesthesiol. 2016; 16(1):118	non pertinent
2579	Goettel et al.,	2016	Br J Anaesth. 2016; 116(6):811-821	non pertinent

2580	Glover et al.,	2016	The lancet. Oncology. 2016; 17(2):224-233	non pertinent
2581	Glassman et al.,	2016	Behav Modif. 2016; 40(5):748-776	non pertinent
2582	Glasser et al.,	2016	J of the peripheral nervous system. 2016; 21(3):260-261	non pertinent
2583	Giusti	2016	Annals of the rheumatic diseases. 2016; 75:45-	non pertinent
2584	Girish et al.,	2016	Biomedical j. 2016; 39(1):72-80	non pertinent
2585	Gingnell et al.,	2016	Br J Psychiatry. 2016; 209(3):229-235	non pertinent
2586	Gimbel et al.,	2016	Pain. 2016; 157(11):2517-2526	non pertinent
2587	Germain et al.,	2016	Sleep. Conference: 30th annual meeting of the associated professional sleep societies. LLC, SLEEP 2016. Denver, CO united states. Conference start: 20160611. Conference end: 20160615. Conference publication: (var.pagings); 39:A96-A97	non pertinent
2588	Gerber et al.,	2016	Clinical lung cancer. 2016; 17(3):169-176	non pertinent
2589	Georgopoulos et al.,	2016	European j of internal medicine. 2016; 32:84-90	non pertinent
2590	Gentile et al.,	2016	European j of vascular and endovascular surgery. 2016; 52(5):643-649	non pertinent
2591	Geiger et al.,	2016	Head & neck. 2016; 38(12):1759-1764	non pertinent
2592	Geha et al.,	2016	JAMA neurology. 2016; 73(6):659-667	non pertinent
2593	Gbadeyan et al.,	2016	Brain stimulation. 2016; 9(4):545-552	non pertinent
2594	Garg et al.,	2016	Clinical infectious diseases. 2016; 62(3):383-391	non pertinent
2595	Garcia-Manero et al.,	2016	The lancet. Oncology. 2016; 17(4):496-508	non pertinent

2596	Gao et al.,	2016	Brain research. 2016; 1632:73-81	non pertinent
2597	Ganz et al.,	2016	Lancet (london, england). 2016; 387(10021):857-865	non pertinent
2598	Ganderton et al.,	2016	BMC women's health. 2016; 16:32	non pertinent
2599	Gaber et al.,	2016	J of obstetrics and gynaecology research. 2016; 42(12):1773-1781	non pertinent
2600	Furie et al.,	2016	Lupus science and medicine. 2016; 3(1)	non pertinent
2601	Ftouni et al.,	2016	Scottish medical j. 2016; 61(1):NP37-NP38	non pertinent
2602	Frijling et al.,	2016	Social cognitive and affective neuroscience. 2016; 11(2):327-336	non pertinent
2603	Friedland et al.,	2016	J of acquired immune deficiency syndromes. 2016; 73(5):489-496	non pertinent
2604	Frick et al.,	2016	Eur neuropsychopharmacol. 2016; 26(11):1775-1783	non pertinent
2605	Freeman et al.,	2016	Trials. 2016; 17(1):134	non pertinent
2606	Francis et al.,	2016	Health technology assessment. 2016; 20(19)	non pertinent
2607	Fonzo et al.,	2016	Neuropsychopharmacology. 2016; 41:291-292	non pertinent
2608	Folprecht et al.,	2016	Annals of oncology : official j of the european society for medical oncology. 2016; 27(7):1273-1279	non pertinent
2609	Flury et al.,	2016	Am J Sports Med. 2016; 44(8):2136-2146	non pertinent
2610	Flood et al.,	2016	J of Pain. 2016; 17(5):600-605	non pertinent
2611	Flohr et al.,	2016	J of vascular surgery. 2016; 63(2):399-406	non pertinent
2612	Fiteni et al.,	2016	The european respiratory j. 2016; 48(3):861-872	non pertinent
2613	Firouzian et al.,	2016	American j of emergency medicine. 2016; 34(3):443-448	non pertinent

2614	Findling et al.,	2016	Pediatric drugs. 2016; 18(5):367-378	non pertinent
2615	Fernández et al.,	2016	Clinical infectious diseases. 2016; 62(5):568-573	non pertinent
2616	Fenton et al.,	2016	JAMA Intern Med. 2016; 176(2):191-197	non pertinent
2617	Feliu-Soler et al.,	2016	BMC Complement Altern Med. 2016;	non pertinent
2618	Fehrenbacher et al.,	2016	Lancet (london, england). 2016; 387(10030):1837-1846	non pertinent
2619	Falsini et al.,	2016	J of translational medicine. 2016; 14(1)	non pertinent
2620	Fallon et al.,	2016	J of clinical oncology. 2016; 34(6):550-556	non pertinent
2621	Falcone et al.,	2016	Brain stimulation. 2016; 9(2):191-196	non pertinent
2622	Fagotti et al.,	2016	European j of cancer (Oxford, England : 1990). 2016; 59:22-3	non pertinent
2623	Fabrizi et al.,	2016	Sci Rep. 2016; 6:28642	non pertinent
2624	Fabbri et al.,	2016	Respiratory research. 2016; 17(1)	non pertinent
2625	Euctr – N.L.	2016	EU Clinical Trial Register. 2016	non pertinent
2626	Euctr – E.S.	2016	EU Clinical Trial Register. 2016	non pertinent
2627	Euctr – E.S.	2016	EU Clinical Trial Register. 2016	non pertinent
2628	Euctr – D.E.	2016	EU Clinical Trial Register. 2016	non pertinent
2629	Euctr – D.E.	2016	EU Clinical Trial Register. 2016	non pertinent
2630	Estape et al.,	2016	Psycho-oncology. 2016; 25:130-131	non pertinent
2631	English et al.,	2016	Archives of physical medicine and rehabilitation. 2016; 97(2):273-280	non pertinent
2632	Engel et al.,	2016	United european gastroenterology j. 2016; 4(5):A618-	non pertinent
2633	Ellis et al.,	2016	Annals of allergy, asthma and immunology. 2016; 117(3):310-317	non pertinent
2634	Ellingsrud et al.,	2016	International j of cardiology. 2016; 202:870-873	non pertinent
2635	Ellegaard et al.,	2016	Arthritis research & therapy. 2016; 18(1)	non pertinent

2636	El-Karaksy et al.,	2016	J of interferon & cytokine research. 2016; 36(1):1-8	non pertinent
2637	Eisenried et al.,	2016	Anesthesia and analgesia. Conference: 2016 annual meeting of the international anesthesia research society. IARS 2016. United states. Conference start: 20160321. Conference end: 20160324; 122(5 Supplement 3):347	non pertinent
2638	Eigentler et al.,	2016	Annals of oncology. 2016; 27(8):1625-1632	non pertinent
2639	Edwards et al.,	2016	Pain medicine (Malden, Mass). 2016; 17(10):1882-1891	non pertinent
2640	Eckstein et al.,	2016	Neuropsychopharmacology. 2016; 41(4):932-939	non pertinent
2641	Eaton et al.,	2016	Annals of surgical oncology. 2016; 23(7):2137-2145	non pertinent
2642	Dymond et al.,	2016	Clinical therapeutics. 2016; 38(11):2447-2458	non pertinent
2643	Duval et al.,	2016	Sci Rep. 2016; 6:22542	non pertinent
2644	Durgam et al.,	2016	International clincial psychopharmacology. 2016; 31(2):61-68	non pertinent
2645	Ducreux et al.,	2016	Annals of oncology. Conference: 41st european society for medical oncology congress, ESMO 2016. Denmark. Conference start: 20161007. Conference end: 20161011; 27	non pertinent

2646	Drummond et al.,	2016	Blood. Conference: 58th annual meeting of the american society of hematology. ASH 2016. United states. Conference start: 20161203. Conference end: 20161206; 128(22)	non pertinent
2647	Drks - Bethge	2016	Deutsches Register Klinischer Studien – DRKS. 2016	non pertinent
2648	Drks – Cabanel	2016	Deutsches Register Klinischer Studien – DRKS. 2016	non pertinent
2649	Drks – Feirreira de Sá	2016	Deutsches Register Klinischer Studien – DRKS. 2016	non pertinent
2650	Drks – Magerl	2016	Deutsches Register Klinischer Studien – DRKS. 2016	non pertinent
2651	Drks – Kinfe	2016	Deutsches Register Klinischer Studien – DRKS. 2016	non pertinent
2652	Drks – Meissner	2016	Deutsches Register Klinischer Studien – DRKS. 2016	non pertinent
2653	Driessen et al.,	2016	European j of cancer (Oxford, England : 1990). 2016; 52:77-84	non pertinent
2654	Draper-Rodi et al.,	2016	Manual therapy. 2016; 25:e167-e168	non pertinent
2655	Downs-Canner et al.,	2016	Molecular therapy. 2016; 24(8):1492-1501	non pertinent
2656	Dou et al.,	2016	Medicine (Baltimore). 2016; 95(42):e5193	non pertinent
2657	Dolder et al.,	2016	Neuropsychopharmacology. 2016; 41(11):2638-2646	non pertinent
2658	Ding et al.,	2016	Osteoarthritis and cartilage. 2016; 24:49-	non pertinent
2659	Dimopoulos et al.,	2016	Lancet oncology. 2016; 17(1):27-38	non pertinent
2660	Dicko et al.,	2016	The lancet. Infectious diseases. 2016; 16(6):674-684	non pertinent
2661	Diaz-Piedra et al.,	2016	Brain Imaging Behav. 2016; 10(4):1184-1197	non pertinent

2662	Di Stefano et al.,	2016	Digestive and liver disease. 2016; 48:e92	non pertinent
2663	Di Pierro et al.,	2016	Acta bio-medica. 2016; 87(3):259-265	non pertinent
2664	Deterding et al.,	2016	Lancet infectious diseases. 2016	non pertinent
2665	Desai et al.,	2016	Zeitschrift für Kinderchirurgie (Surgery in infancy and childhood). 2016; 26(2):160-163	non pertinent
2666	Deppermann et al.,	2016	Behav Brain Res. 2016; 307:208-217	non pertinent
2667	Deplanque et al.,	2016	J of the American Academy of Dermatology. 2016; 74(6):1077-1085	non pertinent
2668	Deogaonkar et al.,	2016	Neuromodulation. 2016; 19(2):142-153	non pertinent
2669	Denis et al.,	2016	Eur J Pain. 2016; 20(5):800-810	non pertinent
2670	Dempster et al.,	2016	J of bone and mineral research. 2016; 31(7):1429-1439	non pertinent
2671	Delgado et al.,	2016	Pediatrics. 2016; 137(2)	non pertinent
2672	Dedic et al.,	2016	J of the American College of Cardiology. 2016; 67(1):16-26	non pertinent
2673	De Wit et al.,	2016	J of internal medicine. 2016; 279(3):283-292	non pertinent
2674	De Souza et al.,	2016	Menopause (New York, N.Y.). 2016; 23(11):1252-1256	non pertinent
2675	De Santis et al.,	2016	The lancet. Infectious diseases. 2016; 16(3):311-320	non pertinent
2676	De Ridder et al.,	2016	Neuromodulation. 2016; 19(1):47-59	non pertinent
2677	De Neve et al.,	2016	Radiotherapy and oncology. 2016; 119:246-247	non pertinent
2678	De Mey et al.,	2016	Drug research. 2016; 66(7):384-392	non pertinent
2679	De Looze et al.,	2016	European j of general practice. 2016; 22(2):111-118	non pertinent
2680	De la Torre et al.,	2016	The lancet. Neurology. 2016; 15(8):801-810	non pertinent
2681	De lure et al.,	2016	Injury. 2016; 47:44-48	non pertinent

2682	De Greef, B.T.A., Merkies et al.,	2016	Trials. 2016; 17(1)	non pertinent
2683	De Greef, B.T.A., Geerts et al.,	2016	Trials. 2016; 17(1)	non pertinent
2684	De Francesco et al.,	2016	J of gastrointestinal and liver diseases. 2016; 25(2):147-150	non pertinent
2685	Dawson et al.,	2016	Stroke: a j of cerebral circulation. 2016; 47(1):143-150	non pertinent
2686	Davis et al.,	2016	J of pediatrics. 2016; 172:136-141e131	non pertinent
2687	Das et al.,	2016	Saudi j of gastroenterology. 2016; 22(4):309-315	non pertinent
2688	Das Nair et al.,	2016	Trials. 2016; 17:54	non pertinent
2689	Das et al.,	2016	J of opioid management. 2016; 12(6):421-430	non pertinent
2690	Darnall et al.,	2016	Pain Med. 2016; 17(2):250-263	non pertinent
2691	Dancey et al.,	2016	Pain. 2016; 157(8):1682-1695	non pertinent
2692	Curran et al.,	2016	Trials. 2016; 17(1)	non pertinent
2693	Curigliano et al.,	2016	Breast (edinburgh, scotland). 2016; 28:191-198	non pertinent
2694	Ctrl – Wakankar	2016	Clinical Trials Registry – India. 2016	non pertinent
2695	Ctrl - Deolekar	2016	Clinical Trials Registry – India. 2016	non pertinent
2696	Cristofanilli et al.,	2016	The lancet. Oncology. 2016; 17(4):425-439	non pertinent
2697	Couturier et al.,	2016	Revue de medecine interne. 2016; 37(12):834-839	non pertinent
2698	Couture et al.,	2016	AANA j. 2016; 84(3):159-165	non pertinent
2699	Cote et al.,	2016	Annals of otology, rhinology and laryngology. 2016; 125(4):317-324	non pertinent
2700	Cosme et al.,	2016	Helicobacter. 2016; 21(1):29-34	non pertinent
2701	Correll et al.,	2016	J of clincial psychiatry. 2016; 77:3-24	non pertinent

2702	Cornpropst et al.,	2016	Allergy:european j of allergy and clinical immunology. 2016; 71(12):1676-1683	non pertinent
2703	Coriat et al.,	2016	International j of nanomedicine. 2016; 11:6207-6216	non pertinent
2704	Conaghan et al.,	2016	Annals of the rheumatic diseases. 2016; 75(6):1024-1033	non pertinent
2705	Comninos et al.,	2016	Endocrine reviews. 2016; 37(2)	non pertinent
2706	Colonnello et al.,	2016	Psychoneuroendo crinology. 2016; 68:69-73	non pertinent
2707	Colloca et al.,	2016	Biological psychiatry. 2016; 79(10):794-802	non pertinent
2708	Colletti et al.,	2016	Therapeutics and clinical risk management. 2016; 12:1365-1376	non pertinent
2709	Collett et al.,	2016	American j of cardiovascular drugs. 2016; 16(1):43-53	non pertinent
2710	Clive et al.,	2016	The lancet oncology. 2016; 17(8):1094-1104	non pertinent
2711	Clark et al.,	2016	Cardiovascular and interventional radiology. 2016; 39(3):124-125	non pertinent
2712	Chuah et al.,	2016	Medicine (united states). 2016; 95(19)	non pertinent
2713	Choi et al.,	2016	American j of preventive medicine. 2016; 51(5 Supplement 3):743-751	non pertinent
2714	Cho et al.,	2016	Joint, bone, spine. 2016; 83(3):324-329	non pertinent
2715	Chi, Ctr Ior - Zhifu	2016	Chinese Clinical Trial Registy – ChCTR. 2016	non pertinent
2716	Chi, Ctr Inr – Sun	2016	Chinese Clinical Trial Registy – ChCTR. 2016	non pertinent
2717	Chi, Ctr Ior - Huang	2016	Chinese Clinical Trial Registy – ChCTR. 2016	non pertinent
2718	Chi, Ctr Inr - Chen	2016	Chinese Clinical Trial Registy – ChCTR. 2016	non pertinent

2719	Chhabra et al.,	2016	International j of oral and maxillofacial surgery. 2016; 45(12):1644-1651	non pertinent
2720	Cheng et al.,	2016	Hepatology. 2016; (baltimore, md). 2016; 64(3):774-784	non pertinent
2721	Chen et al.,	2016	Acta pharmacologica sinica. 2016; 37(11):1509-1515	non pertinent
2722	Chen et al.,	2016	J Orthop Surg Res. 2016; 11(1):47	non pertinent
2723	Chen et al.,	2016	Medicine (Baltimore). 2016; 95(2):e2292	non pertinent
2724	Chan et al.,	2016	Sleep. Conference: 30th annual meeting of the associated professional sleep societies. LLC, SLEEP 2016. Denver, CO united states. Conference start: 20160611. Conference end: 20160615. Conference publication: (var.pagings); 39:A205	non pertinent
2725	Chan et al.,	2016	The lancet. Oncology. 2016; 17(3):367-377	non pertinent
2726	Chalkiadis et al.,	2016	Developmental medicine and child neurology. 2016; 58(4):402-408	non pertinent
2727	Cerezo-Téliez et al.,	2016	Pain. 2016; 157(9):1905-1917	non pertinent
2728	Cavelti-Weder et al.,	2016	Molecular therapy. 2016; 24(5):1003-1012	non pertinent
2729	Castro-Marrero et al.,	2016	Clinical nutrition (edinburgh, scotland). 2016; 35(4):826-834	non pertinent
2730	Caserini et al.,	2016	International j of clinical pharmacology and therapeutics. 2016; 54(1):19-27	non pertinent
2731	Case et al.,	2016	Eneuro. 2016; 3(1):1312-1327	non pertinent
2732	Cardenas et al.,	2016	Revista colombiana de psiquiatria. 2016	non pertinent

2733	Cao et al.,	2016	Clinical pharmacology in drug development. 2016; 5(6):435-449	non pertinent
2734	Campbell et al.,	2016	Pain. 2016; 157(6):1297-1304	non pertinent
2735	Caltagirone et al.,	2016	Transl Stroke Res. 2016; 7(1):54-69	non pertinent
2736	Callegari et al.,	2016	Cardiology in the young. Conference: 50th annual meeting of the association for european paediatric and congenital cardiology, AEPC. Italy. Conference start: 20160601. Conference end: 20160604; 26:42-43	non pertinent
2737	Buvanendran et al.,	2016	Regional anesthesia and pain medicine. 2016; 41(5)	non pertinent
2738	Butler et al.,	2016	Endocrine reviews. 2016; 37(2)	non pertinent
2739	Burke et al.,	2016	Clinical lymphoma, myeloma & leukemia. 2016; 16(4):182-190.e181	non pertinent
2740	Burhan et al.,	2016	Behavioural neurology. 2016:1637392	non pertinent
2741	Bunge et al.,	2016	J of acquired immune deficiency syndromes (1999). 2016; 71(5)	non pertinent
2742	Buerkle et al.,	2016	Haematologica. 2016; 101:61-	non pertinent
2743	Buerkle et al.,	2016	Haematologica. 2016; 101:146-147	non pertinent
2744	Buckley	2016	Irish j of medical science. 2016; 185(3):561-564	non pertinent
2745	Bschleipfer et al.,	2016	European surgery – acta chirurgica austriaca. 2016; 48(3):211-213	non pertinent
2746	Bruce et al.,	2016	Neurology. 2016; 87(18):1871-1877	non pertinent
2747	Brown et al.,	2016	PLoS One. 2016; 11(5)	non pertinent

2748	Brouwers et al.,	2016	Oncotarget. 2016; 7(21):29977-29988	non pertinent
2749	Brose et al.,	2016	Lancet oncology. 2016; 17(9):1272-1282	non pertinent
2750	Brinkhuizen et al.,	2016	J of the American Academy of Dermatology. 2016; 75(1):126-134	non pertinent
2751	Brietzke et al.,	2016	Frontiers in neuroscience. 2016; 9(JAN)	non pertinent
2752	Bridgewater et al.,	2016	BMC Cancer. 2016; 16(1)	non pertinent
2753	Bray et al.,	2016	J of traumatic stress. 2016; 29(4):340-348	non pertinent
2754	Braden et al.,	2016	Brain and behavior. 2016; 6(3):1-13	non pertinent
2755	Broye et al.,	2016	Clinical lung cancer. 2016; 17(2):150-160	non pertinent
2756	Boyce et al.,	2016	British j of pharmacology. 2016; 173(10):1580-1588	non pertinent
2757	Bourcier et al.,	2016	Annals of intensive care. 2016; 6(1)	non pertinent
2758	Borlak et al.,	2016	Oncotarget. 2016; 7(19):28059-28074	non pertinent
2759	Bonani et al.,	2016	American j of transplantation. 2016; 16(6):1882-1891	non pertinent
2760	Boku et al.,	2016	Revista brasileira de anestesiologia. 2016; 66(1):37-43	non pertinent
2761	Bois et al.,	2016	Lancet oncology. 2016; 17(1):78-89	non pertinent
2762	Blom et al.,	2016	Clinical cardiology. Conference: 2016 congress on atherosclerotic cardiovascular disease prevention. United states. 2016; 39:5-6	non pertinent
2763	Bloch et al.,	2016	J of child and adolescent psychopharmacology. 2016; 26(4):327-334	non pertinent
2764	Blikman et al.,	2016	BMJ Open. 2016; 6(3)	non pertinent
2765	Blackhall et al.,	2016	Annals of oncology. 2016; 27:iii35-iii41	non pertinent

2766	Binder et al.,	2016	Pain management. 2016; 6(3):229-	non pertinent
2767	Bilhim	2016	Cardiovascular and interventional radiology. 2016; 39(3):64-65	non pertinent
2768	Bhatnager	2016	Supportive care in cancer. 2016; 24(1):29-30	non pertinent
2769	Bhandari et al.,	2016	Clinical orthopaedics and related research. 2016; 474(5):1234-1244	non pertinent
2770	Betsy et al.,	2016	Photodiagnosis and photodynamic therapy. 2016; 14:84-90	non pertinent
2771	Bernabe-Garcia et al.,	2016	Annals of nutrition & metabolism. 2016; 69(2):150-160	non pertinent
2772	Bermudez-Millan et al.,	2016	J Nutr. 2016; 146(10):2051-2057	non pertinent
2773	Bergfeld et al.,	2016	JAMA Psychiatry. 2016; 73(5):456-464	non pertinent
2774	Bergeron et al.,	2016	J of consulting and clinical psychology. 2016; 84(3):259-268	non pertinent
2775	Benes et al.,	2016	Gastroenterologie a hepatologie. 2016; 70(6):491-499	non pertinent
2776	Benedetti et al.,	2016	J of physiology. 2016; 594(19):5647-5660	non pertinent
2777	Ben-Ami et al.,	2016	Annals of oncology. 2016; 27(9):1794-1799	non pertinent
2778	Bell et al.,	2016	Current medical research and opinion. 2016; 32(5):959-965	non pertinent
2779	Behre et al.,	2016	J of clinical endocrinology and metabolism. 2016; 101(12):4779-4788	non pertinent

2780	Batticciotto et al.,	2016	Arthritis and rheumatology. Conference: american college of rheumatology/association of rheumatology health professionals annual scientific meeting. ACR/ARHP 2016. United states. Conference start: 20161111. Conference end: 20161116; 68:957-958	non pertinent
2781	Bart et al.,	2016	Medicine (united states). 2016; 95(3)	non pertinent
2782	Barrett et al.,	2016	Pain practice. 2016; 16(7):856-868	non pertinent
2783	Barrera et al.,	2016	The lancet. Infectious diseases. 2016; 16(4):421-430	non pertinent
2784	Barrachina et al.,	2016	Anesthesia and Analgesia. 2016; 122(4):986-995	non pertinent
2785	Barbara et al.,	2016	Gut. 2016; 65(1):82-90	non pertinent
2786	Banks et al.,	2016	Arch Phys Med Rehabil. 2016; 97(8):1254-1261	non pertinent
2787	Balius et al.,	2016	Current therapeutic research clinical and experimental. 2016; 78:1-7	non pertinent
2788	Bairey et al.,	2016	Eur Heart J. 2016; 37(19):1504-1513	non pertinent
2789	Bai et al.,	2016	Clinical therapeutics. 2016; 38(2):358-369	non pertinent
2790	Badran et al.,	2016	Neuropsychopharmacology. Conference: 55th annual meeting of the american college of neuropsychopharmacology, ACNP 2016. United states. Conference start: 20161204. Conference end: 20161208; 41:464	non pertinent
2791	Babalonis et al.,	2016	Addiction biology. 2016; 21(1):146-158	non pertinent

2792	Aydinok et al.,	2016	Blood. Conference: 58th annual meeting of the american society of hematology, ASH 2016. United states. Conference start: 20161203. Conference end: 20161206; 128(22)	non pertinent
2793	Ayache et al.,	2016	Frontiers in neuroscience. 2016; 10(APR)	non pertinent
2794	Ather et al.,	2016	F1000research. 2016; 5	non pertinent
2795	Assouline et al.,	2016	Blood. 2016; 128(2):185-194	non pertinent
2796	Assem et al.,	2016	Hepatology international. 2016; 10(2):377-385	non pertinent
2797	Arzola-Paniagua et al.,	2016	Obesity (silver spring, md.). 2016; 24(7):1454-1463	non pertinent
2798	Armstrong et al.,	2016	Lancet (london, england). 2016; 387(10019):679-690	non pertinent
2799	Armstrong et al.,	2016	The lancet. Oncology. 2016; 17(3):378-388	non pertinent
2800	Armand et al.,	2016	British j of haematology. 2016; 173(1):96-104	non pertinent
2801	Arjadi et al.,	2016	Trials. 2016; 17(1)	non pertinent
2802	Argoff et al.,	2016	Clin J Pain. 2016; 32(7):555-561	non pertinent
2803	Arendt-Nielsen et al.,	2016	Pain. 2016; 157(9):2057-2067	non pertinent
2804	Arden et al.,	2016	Osteoarthritis and cartilage. 2016; 24(11):1858-1866	non pertinent
2805	Aragones et al.,	2016	BMC Psychiatry. 2016; 16(1)	non pertinent
2806	Apostolopoulos et al.,	2016	Scandinavian j of gastroenterology. 2016; 51(2):145-151	non pertinent
2807	Anonymous	2016	Lancet. 2016; 388(10043):464	non pertinent
2808	Anonymous	2016	Frontiers in computational neuroscience. 2016; 19(DEC)	non pertinent
2809	Angiolillo et al.,	2016	J of the American College of Cardiology. 2016; 67(6):603-613	non pertinent
2810	Andresen et al.,	2016	Pain. 157(9):2097-2103	non pertinent

2811	Andersson et al.,	2016	Human reproduction (Oxford, England). 2016; 31(1):67-74	non pertinent
2812	An et al.,	2016	Endocrine j. 2016; 63(10):885-895	non pertinent
2813	Almallah et al.,	2016	J of clinical and diagnostic research. 2016; 10(11):ZC23-ZC28	non pertinent
2814	Allison et al.,	2016	J of neuroinflammation. 2016; 13(1)	non pertinent
2815	Alexander et al.,	2016	J of clinical oncology. 2016; 34(34):4094-4101	non pertinent
2816	Albu et al.,	2016	Int J Psychophysiol. 2016; 109:147-152	non pertinent
2817	Al-Motowa et al.,	2016	Middle east african j of ophthalmology. 2016; 23(4):307-310	non pertinent
2818	Al-Batran et al.,	2016	Lancet oncology. 2016; 17(12):1697-1708	non pertinent
2819	Akural et al.,	2016	Scandinavian j of pain. 2016; 11:59-64	non pertinent
2820	Akeju et al.,	2016	PLoS One. 2016; 11(10):e0163431	non pertinent
2821	Ahn et al.,	2016	J of the American College of Cardiology. 2016; 67(12):1412-1422	non pertinent
2822	Ahn et al.,	2016	Cios clinics in orthopedic surgery. 2016; 8(1):78-83	non pertinent
2823	Ahmad et al.,	2016	J of nuclear cardiology. 2016; 23(2):287-297	non pertinent
2824	Addy et al.,	2016	Clinical pharmacology in drug development. 2016; 5(5):374-382	non pertinent
2825	Actrn – Hart	2016	ANZCTR – Australian New Zealand Clinical Trials Registry. 2016	non pertinent
2826	Actrn – Fitzgibbon	2016	ANZCTR – Australian New Zealand Clinical Trials Registry. 2016	non pertinent

2827	Actrn – Nordin	2016	ANZCTR – Australian New Zealand Clinical Trials Registry. 2016	non pertinent
2828	Actrn – Kim	2016	ANZCTR – Australian New Zealand Clinical Trials Registry. 2016	non pertinent
2829	Actrn – Knaevelsrud	2016	ANZCTR – Australian New Zealand Clinical Trials Registry. 2016	non pertinent
2830	Actrn – Halaki	2016	ANZCTR – Australian New Zealand Clinical Trials Registry. 2016	non pertinent
2831	Acosta et al.,	2016	J of neurogastroenterology and motility. 2016; 22(1):69-77	non pertinent
2832	Abonour et al.,	2016	Blood. Conference: 58th annual meeting of the american society of hematology. ASH 2016. United states. Conference start: 20161203. Conference end: 20161206; 128(22)	non pertinent
2833	Abend et al.,	2016	Transl Psychiatry. 2016; 6(10):e913	non pertinent
2834	Abdulatif et al.,	2016	Anaesthesia. 2016; 71(10):1177-1185	non pertinent
2835	AbdElmagied et al.,	2016	Am J Obstet Gynecol. 2016; 215(3):388.e318	non pertinent
2836	Abdel-Kadar et al.,	2016	Obesity surgery. 2016; 26(12):2899-2905	non pertinent
2837	Abdallah et al.,	2016	Anesthesiology. 2016; 124(5):1053-1064	non pertinent
2838	Sugiyama et al.,	2016	J of clinical oncology. 2016 Aug; 34(24):2881-2887	non pertinent
2839	Poiley et al.,	2016	Arthritis and rheumatology. 2016 Aug; 68(8):2027-2034	non pertinent
2840	Hodge et al.,	2016	PLoS One. 2016 Apr; 11(4):e0153151	non pertinent
2841	Reck et al.,	2016	J of thoracic oncology. 2016; 11(6):808-818	non pertinent

2842	Kiru et al.,	2016	Health technology assessment. 2016 Aug; 20(59):1-180	non pertinent
2843	Allas et al.,	2016	Diabetes, obesity and metabolism. 2016 Sep; 18(9):868-874	non pertinent
2844	Schaller et al.,	2016	J of the european academy of dermatology and venerology. 2016; 30(6):966-973	non pertinent
2845	Park et al.,	2016	Clinical therapeutics. 2016 Aug; 38(8):1858-1868.e2	non pertinent
2846	Yanaranop et al.,	2016	Asia-pacific j of clinical oncology. 2016 Sep; 12(3):289-299	non pertinent
2847	Gerrits et al.,	2016	Fertility and sterility. 2016 Apr; 105(4):1056-1062	non pertinent
2848	Imamura et al.,	2016	Annals of surgical oncology. 2016 Sep; 23(9):2928-2935	non pertinent
2849	Wang et al.,	2016	Medicine (united states). 2016 Jul; 95(28):e4135	non pertinent
2850	Meador et al.,	2016	Epilepsy and behavior. 2016 Sep; 62:267-275	non pertinent
2851	Xu et al.,	2016	Integrative cancer therapies. 2016 Sep; 15(3):349-357	non pertinent
2852	Shabsigh et al.,	2016	Sexual medicine. 2016 Sep; 4(3):135-144	non pertinent
2853	Park et al.,	2016	Otolaryngology – head and neck surgery (united states). 2016 Aug; 155(2):324-331	non pertinent
2854	Régis et al.,	2016	J of neurosurgery. 2016 Apr; 124(4):1079-1087	non pertinent
2855	Yao et al.,	2016	Medicine (united states). 2016 Jul; 95(29):e4274	non pertinent
2856	Zhao et al.,	2016	Thyroid. 2016 Aug; 26(8):1019-1029	non pertinent
2857	Chao et al.,	2016	Elife. 2016 Aug; e15691	non pertinent
2858	Kimura et al.,	2016	International j of urology. 2016 May; 23(5):395-403	non pertinent
2859	Fisher et al.,	2016	Clinical ophthalmology. 2016 Jul; 10:1297-1303	non pertinent

2860	Sheehan et al.,	2016	New england j of medicine. 2016 Aug; 375(7):619-630	non pertinent
2861	Eberl et al.,	2016	European j of anaesthesiology. 2016 Sep; 33(9):631-637	non pertinent
2862	Knudson et al.,	2016	Diseases of the colon and rectum. 2016 Sep; 59(9):862-869	non pertinent
2863	Kumar	2016	World j of gastroenterology. 2016 Aug; 22(31):7069-7079	non pertinent
2864	Thompson et al.,	2016	Clinical infectious diseases. 2016 Aug; 63(3):356-362	non pertinent
2865	Morris et al.,	2016	Clinical cancer research. 2016 Aug; 22(15):3774-3781	non pertinent
2866	Jakacki et al.,	2016	J of neuro-oncology. 2016 Aug; 129(1):131-138	non pertinent
2867	Dunn et al.,	2016	British j of haematology. 2016 Apr; 173(2):253-259	non pertinent
2868	Leung et al.,	2016	Trials. 2016 Aug; 17(1):421	non pertinent
2869	Simons et al.,	2016	Anesthesiology. 2016 Sep; 125(3):525-534	non pertinent
2870	Dowlati et al.,	2016	J of pharmacology and experimental therapeutics. 2016 May; 357(2)	non pertinent
2871	Solomon et al.,	2016	J of clinical oncology. 2016 Aug; 34(24):2858-2865	non pertinent
2872	Crews et al.,	2016	Diabetes care. 2016 Aug; 39(8):1371-1377	non pertinent
2873	Rhee et al.,	2016	Drug design, development and therapy. 2016 Aug; 10:2525-2534	non pertinent
2874	Prostran et al.,	2016	Frontiers in aging neuroscience. 2016 Jun; 8:144	non pertinent
2875	Punga et al.,	2016	Dermatologic surgery. 2016; 42(8):967-976	non pertinent
2876	Da Graca-Tarragó et al.,	2016	Pain medicine (united states). 2016 May; 17(5):877-891	non pertinent

2877	No authors listed	2016	Prescrire international. 2016 Mar; 25(169):61-64	non pertinent
2878	Younes et al.,	2016	The lancet oncology. 2016 May; 17(5):622-631	non pertinent
2879	Graff et al.,	2016	Cancer. 2016 Aug; 122(16):2524-2533	non pertinent
2880	Harrison et al.,	2016	Leukemia. 2016 Aug, 30(8):1701-1707	non pertinent
2881	Van Broekhoven et al.,	2016	BMC cancer. 2016 Aug; 16(1):686	non pertinent
2882	Cooper et al.,	2016	Clinical cancer research. 2016 Aug; 22(16):4014-4022	non pertinent
2883	Brodosi et al.,	2016	Annals of hepatology. 2016 Sep-Oct; 15(5):673-681	non pertinent
2884	Schweizer et al.,	2016	Prostate. 2016 Sep; 76(13):1218-1226	non pertinent
2885	Asghar et al.,	2016	PLoS ONE. 2016 Mar; 11(3):e0150334	non pertinent
2886	Krishna et al.,	2016	Clinical science. 2016; 130(3):127-150	non pertinent
2887	Gan et al.,	2016	J of clinical anesthesia. 2016 Jun; 31:249-258	non pertinent
2888	Kimball et al.,	2016	New england j of medicine. 2016 Aug; 375(5):422-434	non pertinent
2889	Kopp et al.,	2016	BMJ open. 2016 Jul; 6(7):e0106651	non pertinent
2890	Schrubbe et al.,	2016	BMC musculoskeletal disorders. 2016 Aug; 17(1):359	non pertinent
2891	Giusti et al.,	2016	COPD: j of chronic obstructive pulmonary disease. 2016 Sep; 13(5):555-560	non pertinent
2892	Medeiros et al.,	2016	Pain medicine. (united states). 2016 Jan; 17(1):122-135	non pertinent
2893	Norton et al.,	2016	J of anxiety disorders. 2016 May; 40:18-28	non pertinent
2894	Nikulina et al.,	2016	Pain. 2016 Jun; 157(8):1791-1798	non pertinent

2895	Shi et al.,	2016	Complementary therapies in medicine. 2016 Apr; 25:71-77	non pertinent
2896	Arnold et al.,	2016	Pediatric rheumatology. 2016 Jul; 14(1):46	non pertinent
2897	Kirichenko et al.,	2016	Phytomedicine. 2016 Oct; 23(11):1198-1210	non pertinent
2898	Li et al.,	2016	Scientific reports. 2016; 6:20298	non pertinent
2899	Zunhammer et al.,	2015	Psychosom Med. 2015; 77(2):156-166	non pertinent
2900	Zongo et al.,	2015	Antimicrobial agents and chemotherapy. 2015; 59(8):4387-4396	non pertinent
2901	Zimran et al.,	2015	Blood cells, molecules & diseases. 2015; 54(1):9-16	non pertinent
2902	Zhu et al.,	2015	British j of cancer. 2015; 112(3):429-437	non pertinent
2903	Zhu et al.,	2015	The lancet. Oncology. 2015; 16(7):859-870	non pertinent
2904	Zhou et al.,	2015	Hematology (amsterdam, netherlands). 2015; 20(3):129-136	non pertinent
2905	Zheng et al.,	2015	Clinical gastroenterology and hepatology. 2015; 13(7//30772878): 1285-1292	non pertinent
2906	Zheng et al.,	2015	Current medical research and opinion. 2015; 31(6):1191-1200	non pertinent
2907	Zhao et al.,	2015	Chin J Integr Med. 2015; 21(11):855-865	non pertinent
2908	Zhang et al.,	2015	Medical oncology (Northwood. London, England). 2015; 32(5):147	non pertinent
2909	Zhang et al.,	2015	Gut. 2015; 64(11):1715-1720	non pertinent
2910	Zhang et al.,	2015	Medicine (united states). 2015; 94(4)	non pertinent
2911	Zeuzem et al.,	2015	Alimentary pharmacology & therapeutics. 2015; 42(7):829-844	non pertinent

2912	Zeidan et al.,	2015	J Neurosci. 2015; 35(46):15307-15325	non pertinent
2913	Zapater et al.,	2015	Basic & clinical pharmacology & toxicology. 2015; 116(3):257-263	non pertinent
2914	Zaman et al.,	2015	European j of cancer (Oxford, England : 1990). 2016; 51(10):1212-1220	non pertinent
2915	Zaghloul et al.,	2015	Pediatric blood and cancer. Conference: 2015 american society of pediatric hematology/oncology. ASPHO 2015. United states. Conference start: 20150506. Conference end: 20150509; 62:80	non pertinent
2916	Zaghloul et al.,	2015	Pediatric blood and cancer. Conference: 2015 american society of pediatric hematology/oncology. ASPHO 2015. United states. Conference start: 20150506. Conference end: 20150509; 62:53	non pertinent
2917	Yun et al.,	2015	Movement disorders. 2015; 30(2):206-213	non pertinent
2918	Yuen et al.,	2015	J of hepatology. 2015; 62(3):526-532	non pertinent
2919	Yu et al.,	2015	Lung cancer: targets and therapy. 2015; 6:1-11	non pertinent
2920	Young et al.,	2015	Neurosurgery. 2015; 77(6):908-915	non pertinent
2921	You et al.,	2015	Medicine. 2016; 94(45):e1968	non pertinent
2922	Yokoe et al.,	2015	J Oral Maxillofac Surg. 2015; 73(3):402-409	non pertinent
2923	Yin et al.,	2015	Pharmacology. 2015; 96(3-4):155-162	non pertinent
2924	Yeo et al.,	2015	Pain physician. 2015; 18(2):173-178	non pertinent
2925	Ye et al.,	2015	Oncotarget. 2015; 7(6):6639-6648	non pertinent

2926	Yazer et al.,	2015	Regional anesthesia and pain medicine. 2015; 40(1):11-15	non pertinent
2927	Yang et al.,	2015	BMC anesthesiology. 2015; 15:161	non pertinent
2928	Yang et al.,	2015	J of oral and maxillofacial surgery. 2015; 73(6):1065-1072	non pertinent
2929	Yang et al.,	2015	Addict Biol. 2015; 20(3):523-533	non pertinent
2930	Wright et al.,	2015	Otalaryngology – head and neck surgery (united states). 2015; 153(1 SUPPL. 1):132-134	non pertinent
2931	Worden et al.,	2015	Endocrine-related cancer. 2015; 22(6):877-887	non pertinent
2932	Wolinsky et al.,	2015	Multiple sclerosis and related disorders. 2015; 4(4):370-376	non pertinent
2933	Wolf et al.,	2015	Radiotherapy and oncology. 2015; 115:27	non pertinent
2934	Wojtalik et al.,	2015	Schizophrenia bulletin. 2015; 41:243	non pertinent
2935	Wilens et al.,	2015	J of the American Academy of Child and Adolescent Psychiatry. 2015; 54(11):916-925.e912	non pertinent
2936	Wierzba et al.,	2015	PLoS One. 2015; 10(7):e0132305	non pertinent
2937	Whitehorn et al.,	2015	Clinical infectious diseases. 2015; 62(4):468-476	non pertinent
2938	Westman et al.,	2015	Alcoholism: clinical and experimental research. 2015; 39:149A	non pertinent
2939	Wenzler et al.,	2015	Antimicrobial agents and chemotherapy. 2015; 59(12):7232-7239	non pertinent
2940	Weiss et al.,	2015	Pediatric rheumatology. 2015; 13(1)	non pertinent
2941	Weisel et al.,	2015	Clinical lymphoma, myeloma & leukemia. 2015; 15(9// (GSK) *Novartis* // *Novartis*):519-530	non pertinent

2942	Weinstock et al.,	2015	Diabetes, obesity & metabolism. 2015; 17(9):849-858	non pertinent
2943	Weigel et al.,	2015	Pain Physician. 2015; 18(2):185-194	non pertinent
2944	Wehry et al.,	2015	Current psychiatry reports. 2015; 17(7// 1R01MH086517 (NIH) *National Institutes of Health* // (NIMH) *National Institutes of Health*)	non pertinent
2945	Warren Olanow et al.,	2015	Annals of neurology. 2015; 78(2):248-257	non pertinent
2946	Wang et al.,	2015	Trials. 2015; 16:584	non pertinent
2947	Wang et al.,	2015	J of alternative and complementary medicine (New York. N.Y.); 21(11):667-672	non pertinent
2948	Wang et al.,	2015	Chinese medical j. 2015; 128(1):46-50	non pertinent
2949	Wang et al.,	2015	Oral Dis. 2015; 21(5):572-582	non pertinent
2950	Wang et al.,	2015	International j of clinical and experimental medicine. 2015; 8(3):3579-3584	non pertinent
2951	Walti et al.,	2015	Musculoskelet Disord. 2015; 16:83	non pertinent
2952	Wall et al.,	2015	BMJ Open. 2015; 5(12)	non pertinent
2953	Walker et al.,	2015	PLoS One. 2015; 10(2)	non pertinent
2954	Wagenlehner et al.,	2015	Lancet (london, england). 2015; 385(9981):1949-1956	non pertinent
2955	Volz et al.,	2015	BMC Neurosci. 2015; 16:15	non pertinent
2956	Volticovschi-Iosob et al.,	2015	Cephalalgia. 2015; 35(6 SUPPL. 1):63	non pertinent
2957	Vogel et al.,	2015	Biol Psychiatry. 2015; 78(12):830-839	non pertinent
2958	Vodicar et al.,	2015	European spine j. (var.pagings). 2015; 24(6 SUPPL. 1):775-776	non pertinent

2959	Visser et al.,	2015	Psychoneuroendocrinology. 2015; 55_8-20	non pertinent
2960	Viscusi et al.,	2015	Pain. 2015; 157(1):264-272	non pertinent
2961	Vincent et al.,	2015	Reproductive sciences. 2015; 22:111A	non pertinent
2962	Vijan et al.,	2015	JAMA – j of the american medical association. 2015; 314(17):1861-1862	non pertinent
2963	Vierimaa et al.,	2015	Diseases of the colon and rectum. 2015; 58(10):943-949	non pertinent
2964	Vesikari et al.,	2015	Pediatric infectious disease j. 2015; 34(12):e298-e307	non pertinent
2965	Verstovsek et al.,	2015	Haematologica. 2015; 100(4):479-488	non pertinent
2966	Verkleij et al.,	2015	British j of general practice. 2015; 65(637):e530-e537	non pertinent
2967	Verduri et al.,	2015	PLoS One. 2015; 10(3):e0118241	non pertinent
2968	Venkitaraman et al.,	2015	European urology. 2015; 67(4):673-679	non pertinent
2969	Vega et al.,	2015	Transplantation. 2015; 99(8):1681-1690	non pertinent
2970	Vaseghi et al.,	2015	Frontiers in human neuroscience. 2015; 9(SEPTEMBER)	non pertinent
2971	Vaseghi et al.,	2015	European j of neuroscience. 2015; 42(7):2426-2437	non pertinent
2972	Vardi et al.,	2015	Aliment Pharmacol Ther. 2015; 42(3):365-374	non pertinent
2973	Vanelderen et al.,	2015	Anesthesiology. 2015;122(2):399-406	non pertinent
2974	Van Zundert	2015	Regional anesthesia and pain medicine. 2015; 40(5):e32-e34	non pertinent
2975	Van Rooij et al.,	2015	Neuropsychopharmacology. 2015; 40(3):667-675	non pertinent
2976	Van Dulmen et al.,	2015	Physiotherapy (united kingdom). 2015; 101:e1573-1574	non pertinent

2977	Van der Heijde et al.,	2015	Rheumatology (oxford, england). 2015; 54(7):1210-1219	non pertinent
2978	Van den Elsen et al.,	2015	Neurology. 2015; 84(23):2338-2346	non pertinent
2979	Van den Bosch et al.,	2015	Neonatology. 2015; 108(1):8-15	non pertinent
2980	Van Cutsem et al.,	2015	Annals of oncology: official j of the european society for medical oncology. 2015; 26(1):149-156	non pertinent
2981	Valle et al.,	2015	The lancet. Oncology. 2015; 16(8):967-978	non pertinent
2982	Unlu et al.,	2015	Pain research & manamegemt. 2015; 20(2):107-111	non pertinent
2983	Unger et al.,	2015	BMC medicine. 2015; 13(1)	non pertinent
2984	Ulgey et al.,	2015	Turkish j of medical sciences. 2015; 45(6):1292-1299	non pertinent
2985	Ubel et al.,	2015	Biological psychology. 2015; 108:78-84	non pertinent
2986	Tuttle et al.,	2015	Pain. 2015; 156(12):2616-2626	non pertinent
2987	Tutag Lehr et al.,	2015	Clinical J of Pain. 2015; 31(5//*Gerber Foundation*):451-458	non pertinent
2988	Tucci et al.,	2015	Therapeutic advances in endocrinology and metabolism. 2015; 6(4):155-162	non pertinent
2989	Tsukada et al.,	2015	J of bone and joint surgery. American volume. 2015; 97(5):367-373	non pertinent
2990	Troyer et al.,	2015	Academic emergency medicine. 2015; 22(5):525-535	non pertinent
2991	Trotti et al.,	2015	Annals of neurology. 2015; 78(3//UL1TR000454(NCATS) *National Center for Advancing Translational Sciences*):454-465	non pertinent

2992	Trimble et al.,	2015	Lancet (london, england). 2015; 386(10008):2078-2088	non pertinent
2993	Trenkwalder et al.,	2015	The lancet. Neurology. 2015; 14(12):1161-1170	non pertinent
2994	Trédan et al.,	2015	Clinical breast cancer. 2015; 15(1):8-15	non pertinent
2995	Trapp et al.,	2015	Back Musculoskelet Rehabil. 2015; 28(4):651-660	non pertinent
2996	Trachtman et al.,	2015	BMC nephrology. 2015; 16(1)	non pertinent
2997	Toosizadeh et al.,	2015	Gerontology. 2015; 61(1):3-14	non pertinent
2998	Tomel et al.,	2015	Psycho-oncology. 2015; 24:158-159	non pertinent
2999	Toledo et al.,	2015	BMC gastroenterology. 2015; 15(1)	non pertinent
3000	Togashi et al.,	2015	Neurosurgery. 2015;76(2):125-134	non pertinent
3001	Tilbrook et al.,	2015	Trails. 2015: 16(1)	non pertinent
3002	Tiemann et al.,	2015	Pain. 2015; 156(2):289-296	non pertinent
3003	Tiberg et al.,	2015	British j of clinical pharmacology. 2015; 80(3):460-472	non pertinent
3004	Thomas	2015	Physiotherapy (united kingdom). 2015; 101:e1512	non pertinent
3005	Tetreault et al.,	2015	Osteoarthritis and cartilage. 2015; 23_A354-	non pertinent
3006	Terkawi et al.,	2015	Pain Physician. 2015; 18(2):E139-E146	non pertinent
3007	Telnes et al.,	2015	Acta anaesthesiologica Scandinavica. 2015; 59(4):496-504	non pertinent
3008	Tedesco Triccas et al.,	2015	Physiotherapy (united kingdom). 2015; 101:e1496-1497	non pertinent
3009	Tatsugami et al.,	2015	International j of cardiovascular imaging. 2015; 31(5):1045-1052	non pertinent
3010	Tasdelen et al.,	2015	J of child and adolescent psychopharmacology. 2015; 25(6):494-500	non pertinent
3011	Tas et al.,	2015	Medical science monitor. 2015; 21:2382-2386	non pertinent

3012	Tarantino et al.,	2015	Annals of surgery. 2015; 262(6):934-940	non pertinent
3013	Tang et al.,	2015	Mediators of inflammation. 2015; 2015:939431	non pertinent
3014	Tan et al.,	2015	European j of pain (united kingdom). 2015; 19(2):271-280	hypnosis for lower back pain
3015	Talpur et al.,	2015	Iranian red crescent medical j. 2015; 17(2)	non pertinent
3016	Tajadini et al.,	2015	Complementary therapies in medicine. 2015; 23(6):767-772	non pertinent
3017	Tai et al.,	2015	Biomed research international. 2015	non pertinent
3018	Taguchi et al.,	2015	J of pain research. 2015; 8:487-497	non pertinent
3019	Tack et al.,	2015	Neurogastroenterology and motility. 2015; 27(2):258-268	non pertinent
3020	Syal et al.,	2015	Psychoneuroendocrinology. 2015; 53:10-15	non pertinent
3021	Swenson et al.,	2015	J Clin Anesth. 2015; 27(8):652-657	non pertinent
3022	Svenningsson et al.,	2015	Brain. 2015; 138(Pt 4):963-973	non pertinent
3023	Suter et al.,	2015	BMC Med Imaging. 2015; 15:11	non pertinent
3024	Sur et al.,	2015	European urology. 2015; 67(5):959-964	non pertinent
3025	Sullivan-Bolyai et al.,	2015	Diabetes educator. 2015; 41(5):537-549	non pertinent
3026	Stundner et al.,	2015	Regional anesthesia and pain medicine. 2015; 40(5)	non pertinent
3027	Stuart et al.,	2015	British j of haematology. 2015; 168(6):796-805	non pertinent
3028	Strauss et al.,	2015	Clinical neurophysiology. 2015; 126(8):e83	non pertinent
3029	Stoddard et al.,	2015	Neuropsychopharmacology. 2015; 40:290-291	non pertinent
3030	Stinson et al.,	2015	Arthritis and rheumatology. 2015; 67	non pertinent

3031	Steinman et al.,	2015	J of behavoir therapy and experimental psychiatry. 2015; 49(Pt A):53-60	non pertinent
3032	Steffens et al.,	2015	Physiotherapy (united kingdom). 2015; 101:e520-	non pertinent
3033	Steenen et al.,	2015	Trails. 2015; 16:536	non pertinent
3034	Stahel et al.,	2015	The lancet. Oncology. 2015; 16(16):1651-1658	non pertinent
3035	Song et al.,	2015	Human reproduction (Oxford, England). 2015; 31(2):332-338	non pertinent
3036	Song et al.,	2015	Haematologica. 2015; 100(2):e63-67	non pertinent
3037	Song et al.,	2015	Vaccine. 2015; 33(41):5437-5444	non pertinent
3038	Soeter et al.,	2015	Biological psychiatry. 2015; 78(12):880-886	non pertinent
3039	Soen et al.,	2015	Current medical research and opinion. 2015; 31(9):1771-1779	non pertinent
3040	Snodgrass et al.,	2015	Physiotherapy (united kingdom). 2015; 101:e1414-	non pertinent
3041	Smolen et al.,	2015	Annals of the rheumatic diseases. 2015; 74(5):843-850	non pertinent
3042	Smith et al.,	2015	JAMA neurology. 2015; 72(1):88-95	non pertinent
3043	Small et al.,	2015	Trials. 2015; 16:329	non pertinent
3044	Slctr – Hapangama et al.,	2015	Sri Lanka Clinical Trials Registry. 2015	non pertinent
3045	Singla et al.,	2015	Physician and sportsmedicine. 2015; 43(2):126-137	non pertinent
3046	Singh et al.,	2015	Indian heart j. 2015; 67:27-28	non pertinent
3047	Simmons et al.,	2015	Clinical ophthalmology (auckland, N.Z.). 2015; 9:657-664	non pertinent
3048	Sieper et al.,	2015	Annals of the rheumatic diseases. 2015; 74:271-272	non pertinent
3049	Siepe et al.,	2015	Eur Spine J. 2015; 24(4):838-851	non pertinent
3050	Sienkiewicz et al.,	2015	European j of pediatrics. 2015; 174(11):1475-1480	non pertinent

3051	Shukuya et al.,	2015	The lancet. Oncology. 2015; 16(16):1630-1638	non pertinent
3052	Shin et al.,	2015	PLoS One. 2015; 10(9):e0138422	non pertinent
3053	Shin et al.,	2015	Neuropsychopharmacology. 2015; 40(8):1919-1927	non pertinent
3054	Shin et al.,	2015	Neural Plast. 2015; 2015:630932	non pertinent
3055	Shin et al.,	2015	clinical gastroenterology and hepatology. 2015; 13(4):701-708	non pertinent
3056	Shang et al.,	2015	J of child and adolescent psychopharmacology. 2015; 25(7):566-573	non pertinent
3057	Shaaban et al.,	2015	Contraception. 2015; 92(4):301-307	non pertinent
3058	Setnik et al.,	2015	J of clinical pharmacology. 2015; 55(12):1351-1361	non pertinent
3059	Seo et al.,	2015	Clinical and vaccine immunology. 2015; 22(11):1160-1165	non pertinent
3060	Sener et al.,	2015	Turkish j of medical sciences. 2015; 45(3):694-699	non pertinent
3061	Senda et al.,	2015	European j of nuclear medicine and molecular imaging. 2015; 42(1):89-96	non pertinent
3062	Sen et al.,	2015	Urology. 2015; 85(6):1247-1251	non pertinent
3063	Sekiguchi et al.,	2015	J of orthopaedic science. 2015; 20(4):617-623	non pertinent
3064	Scott et al.,	2015	Pain research and management. 2015; 20(3):e67	non pertinent
3065	Sclafani et al.,	2015	International j of radiation oncology, biology, physics. 2015; 93(2):303-312	non pertinent
3066	Schuind et al.,	2015	J of infectious diseases. 2015; 212(4):531-541	non pertinent
3067	Schroer et al.,	2015	J of arthroplasty. 2015; 30(9 Supplement):64-67	non pertinent
3068	Schreiner et al.,	2015	Schizophrenia research. 2015; 169(1-3):393-399	non pertinent

3069	Scholl et al.,	2015	Regional anesthesia and pain medicine. Conference: 40th annual regional anesthesia and acute pain medicine meeting of the american society of regional anesthesia and pain medicine. ASRA 2015 las vegas. NV united states. Conference start: 20150514. Conference end: 20150516. Conference publication: (var.pagings); 40(5)	non pertinent
3070	Schneider et al.,	2015	Physiotherapy (united kingdom). 2015; 101:e1349	non pertinent
3071	Schmitt et al.,	2015	J of cardiovascular pharmacology. 2015; 65(6//*Roche*):611-619	non pertinent
3072	Schmidt et al.,	2015	American j of respiratory and critical care medicine. 2015; 191	non pertinent
3073	Schmidt et al.,	2015	Brain. 2015; 138(Pt 1):217-228	non pertinent
3074	Schlaeger et al.,	2015	J of sexual medicine. 2015; 12(4):1019-1027	non pertinent
3075	Schietroma et al.,	2015	J of the american college of surgeons. 2015; 220(5):921-933	non pertinent
3076	Scherz et al.,	2015	J of clinical pharmacology. 2015; 55(6):688-697	non pertinent
3077	Schaub et al.,	2015	BMC Psychiatry. 2015; 15(1)	non pertinent
3078	Schadendorf et al.,	2015	European j of cancer (Oxford, England : 1990). 2015; 51(7):833-840	non pertinent
3079	Scahill et al.,	2015	American j of psychiatry. 2015; 172(12):1197-1206	non pertinent

3080	Scagliotti et al.,	2015	Annals of oncology: official j of the european society for medical oncology. 2015; 26(3):497-504	non pertinent
3081	Sato et al.,	2015	Sci Rep. 2015; 5:12432	non pertinent
3082	Sargin et al.,	2015	Paediatric anaesthesia. 2015; 25(9):950-955	non pertinent
3083	Sardar et al.,	2015	Spine j. 2015; 15(10 SUPPL. 1):89	non pertinent
3084	Sanford et al.,	2015	British j of haematology. 2015; 171(4):471-477	non pertinent
3085	Sandor et al.,	2015	International j of cardiology. 2015; 179:470-475	non pertinent
3086	Sandhu et al.,	2015	Trials. 2015; 16(1)	non pertinent
3087	Sanders et al.,	2015	Arthritis Rheumatol. 2015; 67(3):741-751	non pertinent
3088	Sandborn et al.,	2015	Alimentary pharmacology & therapeutics. 2015; 41(5):409-418	non pertinent
3089	Sanci et al.,	2015	PLoS One. 2015; 10(9):566849	non pertinent
3090	Salehi et al.,	2015	Annals of cardiac anaesthesia. 2015; 18(4):486-490	non pertinent
3091	Sahraian et al.,	2015	Trials. 2015; 16:94	non pertinent
3092	Saez-Llorens et al.,	2015	Human vaccines & immunotherapeutics. 2015; 11(6):1507-1517	non pertinent
3093	Saeed et al.,	2015	International j of surgery (London, England). 2015; 13:76-79	non pertinent
3094	Sacheck et al.,	2015	Clinical trials (london, england). 2015; 12(1):45-53	non pertinent
3095	Sabatine et al.,	2015	New England j of medicine. 2015; 372(16):1500-1509	non pertinent
3096	Saanijoki et al.,	2015	Med Sci Sports Exerc. 2015; 47(12):2604-2611	non pertinent
3097	Saad et al.,	2015	European urology. 2015; 68(4):570-577	non pertinent

3098	Rynn et al.,	2015	J of the American Academy of Child and Adolescent Psychiatry. 2015; 54(3):180-190	non pertinent
3099	Rütgen et al.,	2015	Proceedings of the National Academy of Sciences of the United States of America. 2015; 112(41):E5638-5646	non pertinent
3100	Rutgen et al.,	2015	J Neurosci. 2015; 35(23):8938-3947	non pertinent
3101	Rosenfield et al.,	2015	N Engl J Med. 2015; 373(2):145-153	non pertinent
3102	Röllig et al.,	2015	The lancet. Oncology. 2015; 16(16):1691-1699	non pertinent
3103	Rogers et al.,	2015	J of thoracic and cardiovascular surgery. 2015; 150(6):1610-1619.e1613	non pertinent
3104	Roerink et al.,	2015	Trials. 2015; 16:439	non pertinent
3105	Rivolta et al.,	2015	Schizophr Bull. 2015; 41(5):1105-1114	non pertinent
3106	Riley et al.,	2015	Man Ther. 2015; 20(3):469-474	non pertinent
3107	Riegersperger et al.,	2015	Trials. 2015; 16:182	non pertinent
3108	Rief et al.,	2015	PLoS One. 2015; 10(9):e0136737	non pertinent
3109	Riederer	2015	J fur neurologie, neurochirurgie und psychiatrie. 2015; 16(2):82-83	non pertinent
3110	Riederer	2015	J fur neurologie, neurochirurgie und psychiatrie. 2015; 16(3):119-120	non pertinent
3111	Richardson et al.,	2015	NeuroRehabilitation. 2015; 36(1):115-126	non pertinent
3112	Remenova et al.,	2015	Orphanet j of rare diseases. 2015; 10:81	non pertinent
3113	Reinisch et al.,	2015	Gut. 2015; 64(6):894-900	non pertinent
3114	Ranawaka et al.,	2015	International j of dermatology. 2015; 54(5):555-563	non pertinent
3115	Ramsauer et al.,	2015	The lancet. Infectious diseases. 2015; 15(5):519-527	non pertinent
3116	Rambaldi et al.,	2015	The lancet. Oncology. 2015; 16(15):1525-1536	non pertinent

3117	Ramalingam et al.,	2015	Annals of oncology: official j of the european society for medical oncology. 2015; 26(8):1741-1748	non pertinent
3118	Rajan et al.,	2015	Urologic oncology: seminars and originals investigations. 2015; 33(8):337.e331-337.e336	non pertinent
3119	Raghu et al.,	2015	European respiratory j. 2015; 46(6):1740-1750	non pertinent
3120	Pursnani et al.,	2015	Circulation. Cardiovascular imaging. 2015; 8(3)	non pertinent
3121	Puiu et al.,	2015	Neurology. 2015; 84	non pertinent
3122	Pryor et al.,	2015	Br J Anaesth. 2015; 115 Suppl. 1:i104-i113	non pertinent
3123	Provenza et al.,	2015	Clinical rheumatology. 2015; 34(8):1455-1462	non pertinent
3124	Prossin et al.,	2015	Neuropsychopharmacology. 2015; 40(3):554-565	non pertinent
3125	Pross et al.,	2015	British j of clinical pharmacology. 2015; 80(3):480-492	non pertinent
3126	Prestgaard et al.,	2015	Pain. 2015; 156(9):1683-1691	non pertinent
3127	Preller et al.,	2015	European psychiatry. 2015; 30:22	non pertinent
3128	Preller et al.,	2015	Biological psychiatry. 77(9 SUPPL 1):134S-135S	non pertinent
3129	Preis et al.,	2015	PLoS One. 2015; 10(8):e0137056	non pertinent
3130	Postow et al.,	2015	New England j of medicine. 2015; 372(21):2006-2017	non pertinent
3131	Pollo-Flores et al.,	2015	Digestive and liver disease. 2015; 47(11):957-963	non pertinent
3132	Pogacnik Murillo et al.,	2015	Osteoarthritis and cartilage. 2015; 23:A389-A390	non pertinent

3133	Pleguezuelos et al.,	2015	Clinical and vaccine immunology. 2015; 22(7):828-835	non pertinent
3134	Place et al.,	2015	The lancet. Oncology. 2015; 16(16):1677-1690	non pertinent
3135	Pirio Richardson et al.,	2015	PLoS One. 2015; 10(4):e0124937	non pertinent
3136	Pilge et al.,	2015	Eur J Anaesthesiol. 2015; 32(5):354-365	non pertinent
3137	Pijnenburg et al.,	2015	Brain connectivitiy. 2015; 5(5):303-311	non pertinent
3138	Pignata et al.,	2015	The lancet. Oncology. 2015; 16(5):561-568	non pertinent
3139	Piessevaux et al.,	2015	Neurogatroenterology and motility. 2015; 27(6):805-815	non pertinent
3140	Pierce et al.,	2015	Drug design, development and therapy. 2015; 9:1257-1268	non pertinent
3141	Pickering et al.,	2015	Pain Physician. 2015; 18(3):249-257	non pertinent
3142	Pickering et al.,	2015	Drug Des Devel Ther. 2015; 9:3853-3862	non pertinent
3143	Pickard et al.,	2015	Lancet. 2015; 386(9991):341-349	non pertinent
3144	Phillips	2015	Osteoarthritis and cartilage. 2015; 23:A357	non pertinent
3145	Pezzuolo et al.,	2015	Annals of oncology. Conference: 17th national congress of medical oncology rome italy. Conference start: 20151023. Conference end: 20151025. Conference publication: (var.pagings); 26	non pertinent
3146	Petre et al.,	2015	Hum Brain Mapp. 2015; 36(2):683-694	non pertinent
3147	Perrone et al.,	2015	Annals of oncology: official j of the european society for medical oncology. 2015; 26(4):675-682	non pertinent

3148	Perotti et al.,	2015	Anesthesia and Analgesia. 2015; 121(2):348-356	non pertinent
3149	Perez et al.,	2015	The lancet. Oncology. 2015; 16(15):1556-1568	non pertinent
3150	Pereira et al.,	2015	Trials. 2015; 16(1)	non pertinent
3151	Pelletier et al.,	2015	Arthritis and rheumatology. 2015; 67	non pertinent
3152	Peerdeman et al.,	2015	PLoS One. 2015; 10(10):e0139563	non pertinent
3153	Pauls et al.,	2015	American j of obstetrics and gynecology. 2015; 213(5):718e711-e718e717	non pertinent
3154	Pascher et al.,	2015	American j of transplantation. 2015; 15(5):1283-1292	non pertinent
3155	Pasalar et al.,	2015	Explore: the j of science and healing. 2015; 11(3):199-207	non pertinent
3156	Park et al.,	2015	Surgical endoscopy. 2015; 29(2):453-457	non pertinent
3157	Park et al.,	2015	Pain Physician. 2015; 18(1):93-99	non pertinent
3158	Papaoiu et al.,	2015	J of investigative dermatology. 2015; 135(2):560-568	non pertinent
3159	Papi et al.,	2015	The lancet respiratory medicine. 2015; 3(2):109-119	non pertinent
3160	Papakostas et al.,	2015	American j of psychiatry. 2015; 172(12):1251-1258	non pertinent
3161	Paintsil et al.,	2015	AIDS Care. 2015; 27 Suppl 1:18-27	non pertinent
3162	Packman et al.,	2015	J of pharmaceutical health care and sciences. 2015; 1(1)	non pertinent
3163	Ozturk et al.,	2015	BMC anesthesiology. 2015; 15:104	non pertinent
3164	Ozdemir et al.,	2015	J Back Musculoskelet Rehabil. 2015; 28(4):749-753	non pertinent
3165	Oza et al.,	2015	The lancet. Oncology. 2015; 16(1):87-97	non pertinent
3166	Overton et al.,	2015	Anals of internal medicine. 2015; 162(12):815-824	non pertinent
3167	Otsuka et al.,	2015	J of nippon medical school. 2015; 82(1):21-26	non pertinent

3168	Otruba	2015	Bolest. 2015; 18(1):29-35	non pertinent
3169	Ostenfeld et al.,	2015	Clinical J of Pain. 2015; 31(4//(GSK)*GlaxoSmithKline*):283-293	non pertinent
3170	Oslin et al.,	2015	JAMA Psychiatry. 2015; 72(5):430-437	non pertinent
3171	Ormerod et al.,	2015	JAMA dermatology. 2015; 151(8):854-861	non pertinent
3172	Orliaguet et al.,	2015	Anesthesiology. 2015; 122(4):759-767	non pertinent
3173	Omosa-Manyonyi	2015	PLoS One. 2015; 10(5//(USAID)*United States Agency for International Development*)	non pertinent
3174	Oliveira et al.,	2015	Cephalalgia. 2015; 35(6):43-	non pertinent
3175	Olesen et al.,	2015	Anesthesia and Analgesia. 2015; 121(5):1165-1175	non pertinent
3176	Olesen et al.,	2015	Scandinavian j of gastroenterology. 2015; 50(2):138-144	non pertinent
3177	Ohnmeiss et al.,	2015	Spine j. 2015; 15(10):195S-196S	non pertinent
3178	Ogata et al.,	2015	PLoS One. 2015; 10(3)	non pertinent
3179	O'Neill et al.,	2015	Trials. 2015; 16:186	non pertinent
3180	O'Malley et al.,	2015	Physiotherapy (united kingdom). 2015; 101:eS1140-eS1141	non pertinent
3181	Nygaard et al.,	2015	Alzheimer's research & therapy. 2015; 7(1)	non pertinent
3182	Ntr – Evers	2015	Netherlands Trial Register. 2015	non pertinent
3183	Noguchi et al.,	2015	Cancer immunology, immunotherapy. 2015; 64(4):493-505	non pertinent
3184	Noble et al.,	2015	Health technology assessment. 2015; 19(83):1-93	non pertinent
3185	Nisand et al.,	2015	Medecine nucleaire. 2015; 39(6):502-513	non pertinent
3186	Nielsen et al.,	2015	Basic & clinical pharmacology & toxicology. 2015; 116(2):140-145	non pertinent

3187	Nguyen-Lu et al.,	2015	Canadian j of anesthesia. 2015; 62(8):866-874	non pertinent
3188	Nguyen et al.,	2015	Arthritis research & therapy. 2015; 17(1)	non pertinent
3189	Newby	2015	Lancet. 2015; 385(9985):2383-2391	non pertinent
3190	Nenke et al.,	2015	Psychoneuroendocrinology. 2015; 56:157-167	non pertinent
3191	Neilson et al.,	2015	BMJ Open. 2015; 5(4):e006874	non pertinent
3192	Ndounga et al.,	2015	Malaria j. 2015; 14(1//(EDCTP):* World Health Organization*//(WHO)*World Health Organization)*)	non pertinent
3193	Nct - Walsh	2015	National Library of Medicine. 2015	non pertinent
3194	Nct – Tuuli	2015	National Library of Medicine. 2015	non pertinent
3195	Nct – Isaac	2015	National Library of Medicine. 2015	non pertinent
3196	Nct – Lindberg-Larsen et al.,	2015	Acta Anaesthesiol Scand. 2018 Aug; 62(7):882-892	non pertinent
3197	Nct – Zhang	2015	National Library of Medicine. 2015	non pertinent
3198	Nct – Li	2015	National Library of Medicine. 2015	non pertinent
3199	Nct – Pattison	2015	National Library of Medicine. 2015	non pertinent
3200	Nct – van Poppelen et al.,	2015	BMC Neurol. 2020 Jan; 20(1):40	non pertinent
3201	Nct – Maruani et al.,	2015	Trials. 2018 Jun; 19(1):340	non pertinent
3202	Nct – Cataldo	2015	National Library of Medicine. 2015	non pertinent
3203	Nct – Woolley	2015	National Library of Medicine. 2015	non pertinent
3204	Nct – Chan	2015	National Library of Medicine. 2015	non pertinent
3205	Nct – Hanling	2015	National Library of Medicine. 2015	non pertinent
3206	Nct – Morse	2015	National Library of Medicine. 2015	non pertinent
3207	Nct – Neuts et al.,	2015	Reg Anesth Pain Med. 2018 Oct; 43(7):738-744	non pertinent
3208	Nct – Vogt	2015	National Library of Medicine. 2015	non pertinent
3209	Nct – Coleman	2015	National Library of Medicine. 2015	non pertinent
3210	Nct – Kyriacou	2015	National Library of Medicine. 2015	non pertinent
3211	Nct – Kim	2015	National Library of Medicine. 2015	non pertinent
3212	Nct – McDonald	2015	National Library of Medicine. 2015	non pertinent
3213	Nct – Menderes	2015	National Library of Medicine. 2015	non pertinent

3214	Nct – Schuster	2015	National Library of Medicine. 2015	non pertinent
3215	Nct – Visser	2015	National Library of Medicine. 2015	non pertinent
3216	Nct – Butler	2015	National Library of Medicine. 2015	non pertinent
3217	Nct – Universitair Ziekenhuis Brussel	2015	National Library of Medicine. 2015	non pertinent
3218	Nct – Liaw	2015	National Library of Medicine. 2015	non pertinent
3219	Nct – Barnes	2015	National Library of Medicine. 2015	non pertinent
3220	Nct – Hendler	2015	National Library of Medicine. 2015	non pertinent
3221	Nct - Lefebvre	2015	National Library of Medicine. 2015	non pertinent
3222	Nct – Sprecher	2015	National Library of Medicine. 2015	non pertinent
3223	Nct – Gouin	2015	National Library of Medicine. 2015	non pertinent
3224	Nct – Caumo	2015	National Library of Medicine. 2015	non pertinent
3225	Nct – Le et al.,	2015	Biol Psychiatry Cogn Neurosci Neuroimaging. 2018 Oct; 3(10):836-843	non pertinent
3226	Nct – Thastum et al.,	2015	EClinicalMedicine . 2019 Dec; 17:100214	non pertinent
3227	Nct – Ritvo et al.,	2015	JMIR Res Protoc. 2017 Aug; 6(8):e165	non pertinent
3228	Nct – Buxbaum	2015	National Library of Medicine. 2015	non pertinent
3229	Nct – Malluche	2015	National Library of Medicine. 2015	non pertinent
3230	Nct – Goddard	2015	National Library of Medicine. 2015	non pertinent
3231	Nct – Prüss-Volz	2015	National Library of Medicine. 2015	non pertinent
3232	Nct – Bruun Wyller	2015	National Library of Medicine. 2015	non pertinent
3233	Nct – Teachman	2015	National Library of Medicine. 2015	non pertinent
3234	Nct – Deitos et al.,	2015	Front Hum Neurosci. 2018 Nov; 12:406	non pertinent
3235	Nct – Dornbierer et al.,	2015	Int J Neuropsychopharmacol. 2019 Oct; 22(10):631-639	non pertinent
3236	Nct – Gordon	2015	National Library of Medicine. 2015	non pertinent
3237	Nct – Oken	2015	National Library of Medicine. 2015	non pertinent
3238	Nct – Cerritelli	2015	National Library of Medicine. 2015	non pertinent
3239	Nct – Kara	2015	National Library of Medicine. 2015	non pertinent
3240	Nct – American University of Beirut Medical Center	2015	National Library of Medicine. 2015	non pertinent

3241	Nct – Ostacoli	2015	National Library of Medicine. 2015	non pertinent
3242	Nct – Kong et al.,	2015	Trials. 2018 Dec; 19(1):685	non pertinent
3243	Nct – Day et al.,	2015	Rehabil Psychol. 2019 Aug; 64(3):279-287	non pertinent
3244	Nct – Rakel	2015	National Library of Medicine. 2015	non pertinent
3245	Nct – Lin	2015	National Library of Medicine. 2015	non pertinent
3246	Nct – Anderson	2015	National Library of Medicine. 2015	non pertinent
3247	Nct – Calmo	2015	National Library of Medicine. 2015	non pertinent
3248	Nct – Rawdon et al.,	2015	Psychiatry Res. 2018 May; 263:257-267	non pertinent
3249	Nct – Verma	2015	National Library of Medicine. 2015	non pertinent
3250	Nct – Brill	2015	National Library of Medicine. 2015	non pertinent
3251	Nct – Fashokun	2015	National Library of Medicine. 2015	non pertinent
3252	Nct – Borckardt	2015	National Library of Medicine. 2015	non pertinent
3253	Nct – Caumo	2015	National Library of Medicine. 2015	non pertinent
3254	Nayak et al.,	2015	J of neuro-oncology. 2015; 123(1):129-134	non pertinent
3255	Nawijn et al.,	2015	European neuropsychopharmacology. 2015; 25:323-324	non pertinent
3256	Natelson et al.,	2015	J Pain. 2015; 16(11):1211-1219	non pertinent
3257	Najafi et al.,	2015	Progress in orthodontics. 2015; 16(1)	non pertinent
3258	Murphy et al.,	2015	J of child and adolescent psychopharmacology. 2015; 25(1):57-64	non pertinent
3259	Murakami et al.,	2015	Arthritis research & therapy. 2015; 17:224	non pertinent
3260	Mullane et al.,	2015	Antimicrobial agents and chemotherapy. 2015; 59(3):1435-1440	non pertinent
3261	Morin et al.,	2015	International urogynecology j and pelvic floor dysfunction. 2015; 26(1):47-49	non pertinent
3262	Moreau et al.,	2015	Blood. 2015; 126(23):395	non pertinent
3263	Mooney et al.,	2015	Drug and alcohol dependence. 2015; 153:94-103	non pertinent
3264	Montandon et al.,	2015	Sleep. 2015; 38:A143-A144	non pertinent

3265	Monsereenusorn et al.,	2015	Chotmaihet thangphaet (J of the Medical Association of Thailand). 2015; 98(4):358-364	non pertinent
3266	Monnig et al.,	2015	Alcoholism: clinical and experimental research. 2015; 39:149A	non pertinent
3267	Monagle et al.,	2015	Anesthesia and Analgesia. 2015; 121(4):914-924	non pertinent
3268	Molina et al.,	2015	New England j of medicine. 2015; 373(23//*Bill and Melinda Gates Foundation*):2237-2246	non pertinent
3269	Moliadze et al.,	2015	Brain research bulletin. 2015; 119(Pt A):25-33	non pertinent
3270	Moisset et al.,	2015	Brain Stimul. 2015; 8(1):135-141	non pertinent
3271	Mohammadinejad et al.,	2015	Annals of pharmacotherapy. 2015; 49(9):953-961	non pertinent
3272	Mitchell et al.,	2015	J of pain and symptom management. 2015; 50(3):289-296	non pertinent
3273	Mistraletti et al.,	2015	Minerva Anestesiologica. 2015; 81(12):1298-1310	non pertinent
3274	Min et al.,	2015	Chinese j of tissue engineering research. 2015; 19(3):335-339	non pertinent
3275	Mily et al.,	2015	PLoS One. 2015; 10(9):2013-0366*Hjart-Lungfonden	non pertinent
3276	Mills et al.,	2015	Patient. 2015; 8(4):359-371	non pertinent
3277	Miller et al.,	2015	Orthop Nurs. 2015; 34(4):227-234	non pertinent
3278	Miller et al.,	2015	BMC pharmacology and toxicology. 2015; 16(1)	non pertinent
3279	Milani et al.,	2015	J of arthroplasty. 2015; 30(11):2038-2042	non pertinent
3280	Miclescu et al.,	2015	Scandinavian j of pain. 2015; 8:37-44	non pertinent

3281	Mestre et al.,	2015	Parkinsonism & related disorders. 2015; 21(11):1349-1354	non pertinent
3282	Mesa et al.,	2015	Haematologica. 2015; 100:336-337	non pertinent
3283	Meng et al.,	2015	Chin Med J (Engl). 2015; 128(10):1321-1325	non pertinent
3284	Mello et al.,	2015	Neural plasticity. 2015	non pertinent
3285	Meeus et al.,	2015	Pain practice. 2015; 15(2):98-106	non pertinent
3286	McRae et al.,	2015	J of emergency medicine. 2015; 48(5):581-589	non pertinent
3287	McQuaid et al.,	2015	CMAJ: Canadian Medical Association j. 2015; 187(7//(NIHR)*National Institute for Health Research*):E215-E225	non pertinent
3288	McNamee et al.,	2015	Sleep. 2015; 38:A116	non pertinent
3289	McKavanagh et al.,	2015	European heart j cardiovascular imaging. 2015; 16(4):441-448	non pertinent
3290	McCaskey et al.,	2015	Trials. 2015; 16:571	non pertinent
3291	Mayer et al.,	2015	New England j of medicine. 2015; 372(20):1909-1919	non pertinent
3292	May et al.,	2015	J of neuroscience. 2015; 35(3):1199-1210	non pertinent
3293	Maurice-Szamburski et al.,	2015	JAMA – j of the american medical association. 2015; 313(9):916-925	non pertinent
3294	Matre et al.,	2015	Sleep. 38(10):1607-1617	non pertinent
3295	Martini et al.,	2015	PLoS One. 2015; 10(6)	non pertinent
3296	Martinez et al.,	2015	Lancet (london, england). 2015; 385(9971):857-866	non pertinent
3297	Martin et al.,	2015	J of clinical oncology. 2015; 33(9):1045-1052	non pertinent
3298	Martel-Pelletier et al.,	2015	Osteoporosis international. 2015; 26(1 SUPPL 1):144-145	non pertinent

3299	Marseglia et al.,	2015	J of biological regulators and homeostatic agents. 2015; 29(2):509-514	non pertinent
3300	Marrs et al.,	2015	British j of dermatology. 2015; 173(5):1125-1129	non pertinent
3301	Marottoli et al.,	2015	J of the american geriatrics society. 2015; 63:184	non pertinent
3302	Mardjuadi et al.,	2015	Targeted oncology. 2015; 10(3):375-383	non pertinent
3303	Marder et al.,	2015	Biological psychiatry. 2015; 77(9 Suppl 1):300	non pertinent
3304	Marashi et al.,	2015	Acta medica iranica. 2015; 53(12):733-737	non pertinent
3305	Mao et al.,	2015	Appl Ergon. 2015; 51:80-84	non pertinent
3306	Manwani et al.,	2015	American j of hematology. 2015; 90(5):381-385	non pertinent
3307	Manimmanakorn et al.,	2015	Int J Sports Physiol Perform. 2015; 10(3):388-395	non pertinent
3308	Major et al.,	2015	Gut. 2015; 64:A33	non pertinent
3309	Mahner et al.,	2015	European j of cancer (Oxford, England : 1990). 2015; 51(3):352-358	non pertinent
3310	Mahler et al.,	2015	Respiratory medicine. 2015; 109(8):1031-1039	non pertinent
3311	Maggiore et al.,	2015	Therapeutics and clinical risk management. 2015; 11:557-563	non pertinent
3312	Macian et al.,	2015	Trials. 2015; 16:134	non pertinent
3313	Maas et al.,	2015	BMC medical education. 2015; 15:203	non pertinent
3314	Maas et al.,	2015	Physiotherapy (united kingdom). 2015; 101:e921-e922	non pertinent
3315	Lv et al.,	2015	International j of clinical and experimental medicine. 2015; 8(5):7359-7366	non pertinent
3316	Luo et al.,	2015	Chinese traditional and herbal drugs. 2015; 46(6):878-880	non pertinent
3317	Lunn et al.,	2015	Anesthesiology. 2015; 122(4):884-894	non pertinent

3318	Lund et al.,	2015	Radiation oncology (london, england). 2015; 10(1)	non pertinent
3319	Lueken et al.,	2015	J of affective disorders. 2015; 184:182-192	non pertinent
3320	Lueken et al.,	2015	J Neural Transm (Vienna). 2015; 122(1):135-144	non pertinent
3321	Luckenbill et al.,	2015	Archives of orthopaedic and trauma surgery. 2015; 135(4):455-464	non pertinent
3322	Loomba et al.,	2015	Regional anesthesia and pain medicine. Conference: 40th annual regional anesthesia and acute pain medicine meeting of the american society of regional anesthesia and pain medicine, ASRA 2015 las vegas, NV united states. Conference start: 20150514. Conference end: 20150516. Conference publication (var.pagings); 40(5)	non pertinent
3323	Lonial et al.,	2015	New England j of medicine. 2015; 373(7):621-631	non pertinent
3324	Lomonte et al.,	2015	J of rheumatology. 2015; 42(9):1677-1684	non pertinent
3325	Liu et al.,	2015	J of neurosurgery. Spine. 2015; 23(1):94-98	non pertinent
3326	Lin et al.,	2015	Internet interventions. 2015; 2(1):7-16	non pertinent
3327	Lin et al.,	2015	Respiratory medicine. 2015; 109(1):44-53	non pertinent
3328	Lim et al.,	2015	Cinical drug investigation. 2015; 35(12):833-842	non pertinent
3329	Lim	2015	Evidence-based medicine. 2015; 20(3):105	non pertinent
3330	Lier et al.,	2015	Pain practice. 2015; 15(5):487-495	non pertinent

3331	Liebowitz et al.,	2015	The lancet. Infectious diseases. 2015; 15(9):1041-1048	non pertinent
3332	Li et al.,	2015	Frontiers in pharmacology. 2015; 6(NOV)	non pertinent
3333	Levy et al.,	2015	Human vaccines & immunotherapeutics. 2015; 11(3):620-631	non pertinent
3334	Levkovitz et al.,	2015	World psychiatry. 2015; 14(1):64-73	non pertinent
3335	Leung et al.,	2015	Trials. 2015; 16:200	non pertinent
3336	Letko et al.,	2015	Ophthalmology. 2015; 122(5):939-948	non pertinent
3337	Leroux-Roels et al.,	2015	Vaccine. 2015; 33(4):577-584	non pertinent
3338	Leombruni et al.,	2015	Clinical and experimental rheumatology. 2015; 33(1 Suppl 88):82-85	non pertinent
3339	Leng et al.,	2015	Clinical J of Pain. 2015; 31(7):612-620	non pertinent
3340	Leitgeb et al.,	2015	Haematologica. 2015; 100:468	non pertinent
3341	Leffler et al.,	2015	Gastroenterology. 2015; 148(7):1311-1319.e1316	non pertinent
3342	Lee et al.,	2015	Br J Anaesth. 2015; 114(4):663-668	non pertinent
3343	Lee et al.,	2015	International j of hyperthermia. 2015; 31(8):869-874	non pertinent
3344	Lee et al.,	2015	Cancer. 2015; 121(3//*National Cancer Institute*):432-440	non pertinent
3345	Lee et al.,	2015	International j of clinical and experimental medicine. 2015; 8(8):12135-12148	non pertinent
3346	Lee et al.,	2015	Arch Gerontol Geriatr. 2015; 61(2):154-160	non pertinent
3347	Lee et al.,	2015	Physiol Behav. 2015; 140:148-155	non pertinent
3348	Lee et al.,	2015	World j of gastroenterology. 2015; 21(1):351-359	non pertinent
3349	Lee et al.,	2015	Spine j. 2015; 15(7):1519-1526	non pertinent

3350	Lee et al.,	2015	Clinics and research in hepatology and gastroenterology. 2015; 39(2):237-24	non pertinent
3351	Ledgerwood et al.,	2015	Contemporary clinical trials. 2015; 44:112-118	non pertinent
3352	Ledgerwood et al.,	2015	PLoS One. 10(5):e0125914	non pertinent
3353	Lechner et al.,	2015	Evidence-based complementary and alternative medicine. 2015	non pertinent
3354	Le et al.,	2015	J of clinical oncology. 2015; 33(12):1325-1333	non pertinent
3355	Lazaridou et al.,	2015	J of pain. 2015; 16(4 SUPPL 1):105	non pertinent
3356	Lauruschkus et al.,	2015	Physiotherapy (united kingdom). 2015; 101:e838-	non pertinent
3357	Lankhorst et al.,	2015	Osteoarthritis and cartilage. 2015; 23:A375-	non pertinent
3358	Langley et al.,	2015	J of the pediatric infectious diseases society. 2015; 4(3):242-251	non pertinent
3359	Langenfeld et al.,	2015	Trials. 16:233	non pertinent
3360	Lange et al.,	2015	J of General Internal Medicine. 2015; 30:177-	non pertinent
3361	Lal et al.,	2015	New England j of medicine. 2015; 372(22):2087-2096	non pertinent
3362	Lak et al.,	2015	Trauma monthly. 2015; 20(2):12-16	non pertinent
3363	Lacy et al.,	2015	PLoS One. 2015; 10(7)	non pertinent
3364	Lacourt et al.,	2015	Brain, behavior, and immunity. 2015; 44:32-36	non pertinent
3365	Kwako et al.,	2015	Psychopharmacology (Berl). 2015; 232(1):295-304	non pertinent
3366	Kurmann et al.,	2015	157(1):144-154	non pertinent
3367	Kurganova et al.,	2015	Zhurnal nevrologii i psikhiatrii imeni S.S. Korsakova. 2015; 115(4):30-35	non pertinent
3368	Kuoppamäki et al.,	2015	J of neural transmission (vienna, austria : 1990). 122(12):1709-1714	non pertinent

3369	Kummar et al.,	2015	Clinical cancer research. 2015; 21(7):1574-1582	non pertinent
3370	Kumar et al.,	2015	Exp Brain Res. 2015; 233(6):1745-1759	non pertinent
3371	Kukkonen et al.,	2015	J Bone Joint Surg Am. 2015; 97(21):1729-1737	non pertinent
3372	Kucukoglu et al.,	2015	Italian j of pediatrics. 2015; 41:61	non pertinent
3373	Krug et al.,	2015	New England j of medicine. 2015; 372(21):1987-1995	non pertinent
3374	Kroger et al.,	2015	Neurology. 2015; 84(21):2124-2131	non pertinent
3375	Kowatch et al.,	2015	J of child and adolescent psychopharmacology. 2015; 25(4):306-313	non pertinent
3376	Kovacheva et al.,	2015	Anesthesiology. 2015; 123(1):92-100	non pertinent
3377	Kotela et al.,	2015	Biomed Res Int. 2015; 2015:165908	non pertinent
3378	Koike et al.,	2015	J of neurology. 2015; 262(3):752-759	non pertinent
3379	Kogure et al.,	2015	J of nippon medical school = nippon ika daigaku zasshi. 2015; 82(3):124-129	non pertinent
3380	Koenig et al.,	2015	BMC Psychiatry. 2015; 15:150	non pertinent
3381	Koch et al.,	2015	European neuropsychopharmacology. 2015; 25:266-267	non pertinent
3382	Klein et al.,	2015	British j of dermatology. 2015; 173(1):192-200	non pertinent
3383	Klafke et al.,	2015	Trials. 2015; 16(1)	non pertinent
3384	Kingsbury et al.,	2015	Trials. 2015; 16:77	non pertinent
3385	Kimby et al.,	2015	Leukemia & lymphoma. 2015; 56(9):2598-2607	non pertinent
3386	Kim et al.,	2015	Anticancer research. 2015; 35(6):3531-3536	non pertinent
3387	Kim et al.,	2015	Pharmacology. 2015; 96(1-2):68-75	non pertinent
3388	Kim et al.,	2015	Surgical endoscopy. 2015; 29(12):3795-3802	non pertinent
3389	Kim et al.,	2015	PLoS One. 2015; 10(4)	non pertinent

3390	Kim et al.,	2015	Digestive diseases and sciences. 2015; 60(6):1724-1732	non pertinent
3391	Kim et al.,	2015	Current medical research and opinion. 2015; 31(2):379-389	non pertinent
3392	Kim et al.,	2015	BMC anesthesiology. 2015; 15:139	non pertinent
3393	Khodayari-Rostamabad et al.,	2015	Anesthesiology. 2015; 122(1):140-149	non pertinent
3394	Khayat et al.,	2015	Complementary therapies in medicine. 2015; 23(3):318-324	non pertinent
3395	Khalili-Mahani et al.,	2015	Neuroimage. 2015; 108:396-409	non pertinent
3396	Kereiakes et al.,	2015	American heart j. 2015; 169(6):906-915	non pertinent
3397	Kelly et al.,	2015	Diabetes, obesity & metabolism. 2015; 17(4):414-422	non pertinent
3398	Kehoe et al.,	2015	Lancet (london, england). 2015; 386(9990):249-257	non pertinent
3399	Kazandijan et al.,	2015	J of clinical oncology. 2015; 33(15 SUPPL 1)	non pertinent
3400	Kaya et al.,	2015	Nephrology dialysis transplantation. 2015; 30:iii549	non pertinent
3401	Kataria et al.,	2015	Regional anesthesia and pain medicine. Conference: 40th annual regional anesthesia and acute pain medicine meeting of the american society of regional anesthesia and pain medicine. ASRA 2015 las vegas, NV united states. Conferece start: 20150514. Conference end: 20150516. Conference publication: (var.pagings):40(5)	non pertinent
3402	Kass-Iliyya et al.,	2015	Parkinsonism Relat Disord. 2015; 21(12):1454-1460	non pertinent

3403	Kashefi et al.,	2015	Physiotherapy research: PTR. 2015; 29(1):114-119	non pertinent
3404	Karthikeyan et al.,	2015	Circulation. 2015; 132	non pertinent
3405	Karsdal et al.,	2015	Osteoarthritis and cartilage. 2015; 23:A394-A395	non pertinent
3406	Karsdal et al.,	2015	Osteoarthritis and cartilage. 2015; 23(4):550-558	non pertinent
3407	Kapil et al.,	2015	Clinical therapeutics. 2015; 37(10):2286-2296	non pertinent
3408	Kang et al.,	2015	Annals of oncology : official j of the european society for medical oncology. 2015; 26(12):2457-2463	non pertinent
3409	Kanat et al.,	2015	Neuropsychopharmacology. 2015; 40(11):2632-2638	non pertinent
3410	Kanamoto et al.,	2015	J of ocular pharmacology and therapeutics. 2015; 31(3):156-164	non pertinent
3411	Jund et al.,	2015	Acta oto-laryngologica. 2015; 135(1):42-50	non pertinent
3412	Jprn - Sakuhara	2015	UMIN.-CTR Clinical Trial. 2015	non pertinent
3413	Joura et al.,	2015	New England j of medicine. 2015; 372(8):711-723	non pertinent
3414	Joe et al.,	2015	Cancer prevention research (philadelphia, pa). 2015; 8(12):1131-1137	non pertinent
3415	Jicha et al.,	2015	Drug and alcohol dependence. 2015; 157:179-183	non pertinent
3416	Ji et al.,	2015	J of clinical oncology. 2015; 33(34):4093-4098	non pertinent
3417	Jeyamohan et al.,	2015	Neurosurg Spine. 2015; 23(2):137-143	non pertinent
3418	Jensterie et al.,	2015	Hormones. 2015; 14(1):81-90	non pertinent
3419	Jenkins et al.,	2015	Alimentary pharmacology & therapeutics. 2015; 41(7):636-648	non pertinent

3420	Jarvik et al.,	2015	Contemporary clinical trials. 2015; 45(Pt B):157-163	non pertinent
3421	Jaramillo et al.,	2015	Brain Imaging Behav. 2015; 9(3):445-455	non pertinent
3422	Jaracz et al.,	2015	General hospital psychiatry. 2015; 37(1):36-39	non pertinent
3423	Jackson et al.,	2015	Jama. 2015; 314(3):237-246	non pertinent
3424	Jaberzadeh et al.,	2015	Brain stimulation. 2015; 8(2):337-338	non pertinent
3425	Izikson et al.,	2015	Vaccine. 2015; 33(48):6622-6628	non pertinent
3426	Iwamura et al.,	2015	BMC urology. 2015; 15:120	non pertinent
3427	Isrctn – Critchley	2015	ISRCTN registry. 2015	non pertinent
3428	Isrctn – Hunter	2015	ISRCTN registry. 2015	non pertinent
3429	Isrctn – Kirkham	2015	ISRCTN registry. 2015	non pertinent
3430	Isrctn - Bruin	2015	ISRCTN registry. 2015	non pertinent
3431	Ishida et al.,	2015	Antimicrobial agents and chemotherapy. 2015; 59(8):4919-4929	non pertinent
3432	Irct2015050922165N - Nejatbakhsh	2015	Iranian Registry of Clinical Trials. 2015	non pertinent
3433	Irct2015041421773N - Mozafari	2015	Iranian Registry of Clinical Trials. 2015	non pertinent
3434	Irct2014121020272N – Dahmardeh	2015	Iranian Registry of Clinical Trials. 2015	non pertinent
3435	Irct2014120215620N - Alireza	2015	Iranian Registry of Clinical Trials. 2015	non pertinent
3436	Irct201411288729N – Javadi	2015	Iranian Registry of Clinical Trials. 2015	non pertinent
3437	Irct201406154443N – Moghadam	2015	Iranian Registry of Clinical Trials. 2015	non pertinent
3438	Inan et al.,	2015	Acta neurologica scandinavica. 2015; 132(4):270-277	non pertinent
3439	Ichesco et al.,	2015	Arthritis and rheumatology. 2015; 67	non pertinent
3440	Hwang et al.,	2015	World neurosurgery. 2015; 83(1):108-113	non pertinent
3441	Hwang et al.,	2015	World j of gastroenterology. 2015; 21(35):10234-10241	non pertinent

3442	Hurwitz et al.,	2015	J of clinical oncology. 2015; 33(34):4039-4047	non pertinent
3443	Hurvitz et al.,	2015	The lancet. Oncology. 2015; 16(7):816-829	non pertinent
3444	Hurt et al.,	2015	International j of radiation oncology, biology, physics. 2015; 93(4):810-818	non pertinent
3445	Hunter et al.,	2015	Gastroenterology. 2015; 148(2):324-333	non pertinent
3446	Hunter et al.,	2015	Osteoarthritis Cartilage. 2015; 23(7):1090-1098	non pertinent
3447	Hung et al.,	2015	Nephrology, dialysis, transplantation. 2015; 30(2):266-274	non pertinent
3448	Hubacher et al.,	2015	Contraception. 2015; 91(3):185-192	non pertinent
3449	Huang et al.,	2015	Gastroenterology. 2015; 148(4 SUPPL 1):678-679	non pertinent
3450	Hu et al.,	2015	Plos neglected tropical diseases. 2015; 9(3)	non pertinent
3451	Hu et al.,	2015	PLoS One. 2015; 10(3):e0121264	non pertinent
3452	Hu et al.,	2015	Chinese j of tissue engineering research. 2015; 19(13):1995-1999	non pertinent
3453	Hsu et al.,	2015	PLoS One. 2015; 10(2)	non pertinent
3454	Hsin-Ti et al.,	2015	Current pharmaceutical biotechnology. 2015; 16(12):1063-1069	non pertinent
3455	Hofmann et al.,	2015	Contemporary clinical trials. 2015; 43:223-230	non pertinent
3456	Hoffmann et al.,	2015	Circulation. 2015; 132	non pertinent
3457	Hodkinson et al.,	2015	Pain. 2015; 156(7):1301-1310	non pertinent
3458	Hisey et al.,	2015	J Spinal Disord Tech. 2015; 28(4):E237-243	non pertinent
3459	Hill et al.,	2015	J of clinical rheumatology. 2015; 21(3):120-125	non pertinent
3460	Hiemstra et al.,	2015	European child & adolescent psychiatry. 2015; 24(1 SUPPL 1):162-163	non pertinent

3461	Herren	2015	BMC proceedings. 2015; 9(3)	non pertinent
3462	Herigstad et al.,	2015	Chest. 2015; 148(4):953-961	non pertinent
3463	Heo et al.,	2015	J of gastroenterology and hepatology (australia). 2015; 30(9):1361-1366	non pertinent
3464	Henningsson et al.,	2015	Transl Psychiatry. 2015; 5:e688	non pertinent
3465	Helistrom et al.,	2015	Urology. 2015; 86(2):291-298	non pertinent
3466	Heidrich et al.,	2015	PLoS One. 2015; 10(6)	non pertinent
3467	He et al.,	2015	Clin Neurol Neurosurg. 2015; 134:130-135	non pertinent
3468	He et al.,	2015	Sleep. 2015; 38(3):423-431	non pertinent
3469	He et al.,	2015	J of spinal disorders & techniques. 2015; 28(7):E427-432	non pertinent
3470	Hatemi et al.,	2015	New England j of medicine. 2015; 372(16):1510-1518	non pertinent
3471	Hartvickson et al.,	2015	International j of infectious diseases. 2015; 41:65-72	non pertinent
3472	Harris et al.,	2015	PLoS Med. 2015; 12(2):e1001783	non pertinent
3473	Harrington et al.,	2015	Expert review of anticancer therapy. 2015; 15(12):1389-1403	non pertinent
3474	Hara et al.,	2015	Physiotherapy (united kingdom). 2015; 101:e523-524	non pertinent
3475	Hansen et al.,	2015	Basic Clin Pharmacol Toxicol. 2015; 117(5):316-322	non pertinent
3476	Hansen et al.,	2015	Br J Clin Pharmacol. 2015; 79(6):926-936	non pertinent
3477	Hansen	2015	BMC Complement Alern Med. 2015;	non pertinent
3478	Hansen et al.,	2015	Trials. 2015; 16:166	non pertinent
3479	Handprasertpong et al.,	2015	Chotmaihet thangphaet (J of the Medical Association of Thailand). 2015; 98(8):734-738	non pertinent

3480	Handen et al.,	2015	J of the American Academy of Child and Adolescent Psychiatry. 2015; 54(11):905-915	non pertinent
3481	Han et al.,	2015	Human vaccines and immunotherapeutics. 2015; 11(5):1094-1101	non pertinent
3482	Halota et al.,	2015	J of viral hepatitis. 2015; 22(8):651-657	non pertinent
3483	Halligan et al.,	2015	Health technology assessment. 2015; 19(54)	non pertinent
3484	Hale et al.,	2015	J of pain research. 2015; 8:623-636	non pertinent
3485	Halasa et al.,	2015	J of the pediatric infectious diseases society. 2015; 4(3):214-224	non pertinent
3486	Hainsworth et al.,	2015	Cancer. 2015; 121(10):1654-1661	non pertinent
3487	Hahn et al.,	2015	JAMA Psychiatry. 2015; 72(1):68-74	non pertinent
3488	Hagenaars et al.,	2015	Frontiers in Psychiatry. 2015; 6(JUN)	non pertinent
3489	Hadi et al.,	2015	International j of pediatric otorhinolaryngology. 2015; 79(5):671-676	non pertinent
3490	Guo et al.,	2015	Trials. 2015; 16:124	non pertinent
3491	Guevara-gutierrez et al.,	2015	J of dermatological treatment. 2015; 26(6):548-550	non pertinent
3492	Gudavalli et al.,	2015	Trials. 2015; 16:259	non pertinent
3493	Guazzi et al.,	2015	European j of oral implantology. 2015; 8(3):283-290	non pertinent
3494	Guan et al.,	2015	PLoS One. 2015; 10(4):e0123675	non pertinent
3495	Gross et al.,	2015	The lancet HIV. 2015; 2(1):e12-e19	non pertinent
3496	Grob et al.,	2015	The lancet. Oncology. 2015; 16(13):1389-1398	non pertinent
3497	Grillon et al.,	2015	Neuropsychopharmacology. 2015; 40(5):1064-1071	non pertinent

3498	Grigg-Damberger et al.,	2015	Child and adolescent psychiatric clinics of north america. 2015; 24(1):145-176	non pertinent
3499	Green et al.,	2015	Oncologist. 2015; 20(5):516-522	non pertinent
3500	Green et al.,	2015	J of clinical psychiatry. 2015; 76(10):1359-1365	non pertinent
3501	Graversen et al.,	2015	Neuropsychobiolo gy. 2015; 71(4):241-252	non pertinent
3502	Graversen et al.,	2015	Basic Clin Pharmacol Toxicol. 2015; 116(5):414-422	non pertinent
3503	Grant et al.,	2015	J of clinical apheresis. 2015; 30(2):126-127	non pertinent
3504	Grainger et al.,	2015	Lancet (london, england). 2015; 386(10004):1649-1658	non pertinent
3505	Gracies et al.,	2015	Lancet Neurol. 2015; 14(10):992-1001	non pertinent
3506	Gough et al.,	2015	JAMA surgery. 2015; 150(9):835-840	non pertinent
3507	Gorse et al.,	2015	Vaccine. 2015; 33(9):1151-1159	non pertinent
3508	Gorka et al.,	2015	Neuropsychophar macology. 2015; 40(2):278-286	non pertinent
3509	Gorka et al.,	2015	International j of neuropsychophar macology. 2015; 18(3)	non pertinent
3510	Gorka et al.,	2015	Neuropsychophar macology. 2015; 40:447	non pertinent
3511	Gong et al.,	2015	J of arthroplasty. 2015; 30(11):1897-1901	non pertinent
3512	Gollwitzer et al.,	2015	J Bone Joint Surg Am. 2015; 97(9):701-708	non pertinent
3513	Goldstein-Piekarski et al.,	2015	Biological psychiatry. 2015; 77(9 SUPPL 1):243	non pertinent
3514	Godeaux et al.,	2015	Vaccine. 2015; 33(18):2189-2195	non pertinent
3515	Gnant et al.,	2015	Lancet. 2015; 386(9992):433-443	non pertinent
3516	Gladkov et al.,	2015	Oncologist. 2015; 21(1):7-15	non pertinent
3517	Gisbert et al.,	2015	Alimentary pharmacology & therapeutics. 2015; 41(8):768-775	non pertinent

3518	Gilron et al.,	2015	Pain. 2015; 156(8):1440-1448	non pertinent
3519	Ghanima et al.,	2015	Lancet (london, england). 2015; 385(9978):1653-1661	non pertinent
3520	Ghaleiha et al.,	2015	Psychiatry research. 2015; 229(1-2):181-187	non pertinent
3521	Ghaeminia et al.,	2015	J of cranio-maxillo-facial surgery. 2015; 43(10):2158-2167	non pertinent
3522	Genovese et al.,	2015	J of clinical rheumatology. 2015; 21(5):231-238	non pertinent
3523	Geng et al.,	2015	Conf Proc IEEE Eng Med Biol Soc. 2015:2071-2074	non pertinent
3524	Gaztanaga et al.,	2015	Atherosclerosis. 2015; 240(1):53-60	non pertinent
3525	Gauvreau et al.,	2015	American j of respiratory and critical care medicine. 2015; 191(2):161-167	non pertinent
3526	Gatlin et al.,	2015	Foot Ankle Int. 2015; 36(3):288-292	non pertinent
3527	Garland et al.,	2015	J of behavioral medicine. 2015; 38(2):327-336	non pertinent
3528	Garcia-Aguilar et al.,	2015	Lancet oncology. 2015; 16(15):1537-1546	non pertinent
3529	Garcia et al.,	2015	Lancet oncology. 2015; 16(1):108-116	non pertinent
3530	Fukushima et al.,	2015	Supportive care in cancer. 2015; 23(1 SUPPL 1):100	non pertinent
3531	Fruhauf et al.,	2015	J of the european academy of dermatology and venerology : JEADV. 2015; 29(5):919-924	non pertinent
3532	Frey et al.,	2015	Vaccine. 2015; 33(1):163-173	non pertinent
3533	Freeman et al.,	2015	BMJ (online). 2015; 350	non pertinent
3534	Fredrickson et al.,	2015	Anaesthesia and intensive care. 2015; 43(4):449-453	non pertinent
3535	Frass et al.,	2015	Complementary therapies in medicine. 2015; 23(3):309-317	non pertinent
3536	Fox et al.,	2015	BMJ Open. 2015; 5(1)	non pertinent

3537	Forward et al.,	2015	Perm J. 2015; 19(4):18-28	non pertinent
3538	Forsth et al.,	2015	European spine j. (var.pagings). 2015; 24(6 SUPPL 1):683	non pertinent
3539	Forst et al.,	2015	J of rehabilitation research and development. 2015; 52(4):397-406	non pertinent
3540	Fonzo et al.,	2015	Neuropsychopharmacology. 2015; 40:278-279	non pertinent
3541	Foerster et al.,	2015	Arthritis and rheumatology. 2015; 67(2):576-581	non pertinent
3542	Florkiewicz et al.,	2015	Acta anaesthesiologica Scandinavica. 2015; 59(8):999-1008	non pertinent
3543	Fleming et al.,	2015	Neurorehabilitation and neural repair. 2015; 29(2):143-152	non pertinent
3544	Fleet et al.,	2015	BJOG. 2015; 122(7):983-992	non pertinent
3545	Flaherty et al.,	2015	J of clinical oncology. 2015; 33(21):2384-2391	non pertinent
3546	Fisher et al.,	2015	Neuropsychopharmacology. 2015; 40(6):1510-1518	non pertinent
3547	Finn et al.,	2015	The lancet. Oncology. 2015; 16(1):25-35	non pertinent
3548	Feuerbach et al.,	2015	British j of pharmacology. 2015; 172(5):1292-1304	non pertinent
3549	Feng et al.,	2015	Dermatologic surgery. 2015; 41:56-63	non pertinent
3550	Fei et al.,	2015	European j of gynaecological oncology. 2015; 36(4):442-446	non pertinent
3551	Fehse et al.,	2015	Pain medicine (united states). 2015; 16(10):1967-1974	non pertinent
3552	Feger et al.,	2015	Radiol. 2015; 25(7):2115-2124	non pertinent
3553	Farell et al.,	2015	Obesity surgery. 2015; 25(11):1996-1997	non pertinent
3554	Fang et al.,	2015	Pain Pract. 2015; 15(7):595-603	non pertinent
3555	Fald Elmula et al.,	2015	J of hypertension. 2015; 33(12):2534-2545	non pertinent
3556	Everaerd et al.,	2015	Neuroimage. 2015; 112:218-224	non pertinent

3557	Euctr N.L.	2015	EU Clinical Trials Register. 2015	non pertinent
3558	Euctr G. B.	2015	EU Clinical Trials Register. 2015	non pertinent
3559	Euctr G. B.	2015	EU Clinical Trials Register. 2015	non pertinent
3560	Euctr F. R.	2015	EU Clinical Trials Register. 2015	non pertinent
3561	Euctr F. R.	2015	EU Clinical Trials Register. 2015	non pertinent
3562	Euctr F. R.	2015	EU Clinical Trials Register. 2015	non pertinent
3563	Euctr F. R.	2015	EU Clinical Trials Register. 2015	non pertinent
3564	Euctr E. S.	2015	EU Clinical Trials Register. 2015	non pertinent
3565	Esposito et al.,	2015	Eur J Oral Implantol. 2015; 8(3):257-268	non pertinent
3566	Emrich et al.,	2015	Mmw-fortschritte der medizin. 2015; 157:9-16	non pertinent
3567	Embree et al.,	2015	Clinical and vaccine immunology. 2015; 22(3):282-290	non pertinent
3568	Elsenbruch et al.,	2015	Neurogastroenterology and motility. 2015;	non pertinent
3569	Egorova et al.,	2015	Pain. 2015; 156(7):1342-1347	non pertinent
3570	Egbers et al.,	2015	Diagn Intery Radiol. 2015; 21(3):215-221	non pertinent
3571	Eckstein et al.,	2015	Biological psychiatry. 2015; 77(9):47-	non pertinent
3572	Eckstein et al.,	2015	Biol Psychiatry. 2015; 78(3):194-202	non pertinent
3573	Eckstein et al.,	2015	Arthritis Rheumatol. 2015; 67(12):3184-3189	non pertinent
3574	Eckhoff et al.,	2015	European j of cancer (Oxford, England : 1990). 2015; 51(3):292-300	non pertinent
3575	Eberl et al.,	2015	BMC complementary and alternative medicine. 2015; 15:406	non pertinent
3576	Ebbert et al.,	2015	JAMA – j of the american medical association. 2015; 313(7):687-694	non pertinent
3578	R. B. R. dxp3k – de Freitas	2015	Registro Brasileiro de Ensaios Clinicos. 2015	non pertinent

3579	Dwivedi et al.,	2015	International j of research in ayurverda and pharmacy. 2015; 6(2):157-160	non pertinent
3580	Duffy et al.,	2015	Nutrition (Burbank, Los Angeles County, Calif.). 2015; 31(10):1247-1254	non pertinent
3581	Drumond Marra et al.,	2015	Behavioural neurology. 2015	non pertinent
3582	Drks - Donath	2015	Deutsches Register klinischer Studien. 2015	non pertinent
3583	Drks – Heissel	2015	Deutsches Register klinischer Studien. 2015	non pertinent
3584	Drks – Straube	2015	Deutsches Register klinischer Studien. 2015	non pertinent
3585	Drks – Szabo	2015	Deutsches Register klinischer Studien. 2015	non pertinent
3586	Driban et al.,	2015	Arthritis and rheumatology. 2015; 67	non pertinent
3587	Douglas et al.,	2015	New England j of medicine. 2015; 372(14):1291-1300	non pertinent
3588	Doring et al.,	2015	European j of pain (united kingdom). 2015; 19(7):966-972	non pertinent
3589	Donnell et al.,	2015	Brain stimulation. 2015; 8(6):1085-1092	non pertinent
3590	Dongre et al.,	2015	Biomed research international. 2015:284154	non pertinent
3591	Dongol et al.,	2015	J of oral and maxillofacial surgery. 2015; 73(6):1124-1132	non pertinent
3592	Doebele et al.,	2015	Cancer. 2015; 121(6):883-892	non pertinent
3593	Do et al.,	2015	J of breast cancer. 2015; 18(1):87-96	non pertinent
3594	Dixon-Gordon et al.,	2015	Compr Psychiatry. 2015;	non pertinent
3595	Dixon et al.,	2015	Respiratory research. 2015; 16(1)	non pertinent
3596	Dingemans et al.,	2015	Annals of oncology. 2015; 26(11):2286-2293	non pertinent
3597	Dinat et al.,	2015	PLoS One. 2015; 10(5)	non pertinent
3598	Diers et al.,	2015	Brain Res. 2015; 1594:173-182	non pertinent

3599	Diaz-Piedra et al.,	2015	Sleep. 2015; 38:A310	non pertinent
3600	Di Vasta et al.,	2015	Obstetrics and gynecology. 2015; 126(3):617-627	non pertinent
3601	Deplanque et al.,	2015	Annals of oncology : official j of the european society for medical oncology. 2015; 26(6):1194-1200	non pertinent
3602	Denduluri et al.,	2015	Regional anesthesia and pain medicine. Conference: 40th annual regional anesthesia and acute pain medicine meeting of the american society of regional anesthesia and pain medicine, ASRA 2015 las vegas, NV united states. Conference start: 20150514. Conference end: 20150516. Conference publication: (var.pagings); 40(5)	non pertinent
3603	Della Negra et al.,	2015	Pediatric infectious disease j. 2015; 34(4):398-405	non pertinent
3604	Del Valle Rubido et al.,	2015	European neuropsychopharmacology. 2015; 25:646-647	non pertinent
3605	Deichmann et al.,	2015	Vaccine. 2015; 33(20):2379-2386	non pertinent
3606	Deftereos et al.,	2015	Circulation. 2015; 132(15):1395-1403	non pertinent
3607	De Wolf et al.,	2015	Radiotherapy and oncology. 2015; 115:27	non pertinent
3608	De Silva et al.,	2015	British j of cancer. 2015; 113(9):1305-1312	non pertinent
3609	De Lange-Brokaar et al.,	2015	Arthritis Rheumatol. 2015; 67(3):733-740	non pertinent
3610	De La Garza et al.,	2015	Progress in neuro-psychopharmacology & biological psychiatry. 2015; 59:40-48	non pertinent

3611	De Kleine et al.,	2015	J of anxiety disorders. 2015; 34:63-67	non pertinent
3612	De Dreu et al.,	2015	Soc Cogn Affect Neurosci. 2015; 10(5):721-728	non pertinent
3613	De Bejczy et al.,	2015	Alcoholism, clinical and experimental research. 2015; 39(11):2189-2199	non pertinent
3614	Davis et al.,	2015	European j of cancer (Oxford, England : 1990). 2015; 51(13):1794-1802	non pertinent
3615	Davies et al.,	2015	Jama. 2015; 314(7):687-699	non pertinent
3616	Das et al.,	2015	European j of anaesthesiology. 2015; 32(4):278-280	non pertinent
3617	Danese et al.,	2015	Gut. 2015; 64(2):243-249	non pertinent
3618	Dahlke et al.,	2015	J of chlinical pharmacology. 2015; 55(4):415-422	non pertinent
3619	Da Silva Ribeiro et al.,	2015	Topics in stroke rehabilitation. 2015; 22(4):299-305	non pertinent
3620	D'Haens et al.,	2015	Gut. 2015; 64(8):1227-1235	non pertinent
3621	Currow et al.,	2015	J of pain and symptom management. 2015; 49(5):814-821	non pertinent
3622	Crunelle et al.,	2015	Human brain mapping. 2015; 36(10):4222-4230	non pertinent
3623	Crockett et al.,	2015	Curr Biol. 2015; 25(14):1852-1859	non pertinent
3624	Crevat et al.,	2015	Pediatric infectious disease j. 2015; 34(8):884-892	non pertinent
3625	Cozzi et al.,	2015	Joint, bone, spine. 2015; 82(2):104-108	non pertinent
3626	Coughtrey et al.,	2015	Behav Cogn Psychother. 2015; 43(3):257-269	non pertinent
3627	Cotreau et al.,	2015	Clinical pharmacology in drug development. 2015; 4(2):137-142	non pertinent
3628	Cortes et al.,	2015	Breast cancer research and treatment. 2015; 154(3):509-520	non pertinent

3629	Corte et al.,	2015	Respiratory research. 2015; 16(1)	non pertinent
3630	Corli et al.,	2015	J Rheumatol. 2015; 42(12):2376-2382	non pertinent
3631	Cömert Kilic et al.,	2015	J of oral and maxillofacial surgery. 2015; 73(8):1473-1483	non pertinent
3632	Combs et al.,	2015	American j of obstetrics and gynecology. 2015; 213(3):364e361-364e312	non pertinent
3633	Collins et al.,	2015	British j of haematology. 2015; 170(6):886-890	non pertinent
3634	Clavijo et al.,	2015	Cardiovascular revascularization medicine. 2015; 16(8):450-454	non pertinent
3635	Clark-Elford et al.,	2015	International j of neuropsychophar macology. 2015; 18(2):1-7	non pertinent
3636	Chu et al.,	2015	Drug Alcohol Depend. 2015; 153:314-322	non pertinent
3637	Chowdhury et al.,	2015	Annals of indian academy of neurology. 2015; 18(6 SUPPL 1):79	non pertinent
3638	Chow et al.,	2015	The lancet. Oncology. 2015; 16(15):1463-1472	non pertinent
3639	Childress et al.,	2015	J of child and adolescent psychopharmacol ogy. 2015; 25(5):402-414	non pertinent
3640	Chen et al.,	2015	Mol Pain. 2015; 11:67	non pertinent
3641	Chen et al.,	2015	Zhongguo zhen jiu (Chinese acupuncture & moxibustion). 2015; 35(10):1005-1009	non pertinent
3642	Chen et al.,	2015	J of cataract and refractive surgery. 2015; 41(2):415-421	non pertinent
3643	Chapman et al.,	2015	Lancet. 2015; 386(9991):360-368	non pertinent
3644	Chang et al.,	2015	Turkish j of gastroenterology. 2015; 26(5):417-422	non pertinent
3645	Chang et al.,	2015	Pain medicine (Malden, Mass.). 2015; 16(12):2397-2404	non pertinent

3646	Chandra et al.,	2015	Malaria j. 2015; 14:108	non pertinent
3647	Chandar et al.,	2015	J of pediatric gastroenterology and nutrition. 2015; 60(6):762-768	non pertinent
3648	Cesne et al.,	2015	Lancet oncology. 2015; 16(3):312-319	non pertinent
3649	Cerit et al.,	2015	European neuropsychopharmacology. 2015; 25(12):2289-2299	non pertinent
3650	Celik et al.,	2015	Biomed research international. 2015; 349806	non pertinent
3651	Ceko et al.,	2015	J Neurosci. 2015; 35(33):11595-11605	non pertinent
3652	Catus et al.,	2015	The lancet neurology. 2015; 14(10):992-1001	non pertinent
3653	Castelo-Branco et al.,	2015	Maturitas. 2015; 81(1):76-82	non pertinent
3654	Case et al.,	2015	Neuromuscular disorders. 2015; 25(4):321-332	non pertinent
3655	Cao et al.,	2015	Scandinavian j of gastroenterology. 2015; 50(10):1185-1190	non pertinent
3656	Calvaruso et al.,	2015	American j of hematology. 2015; 90(7):634-638	non pertinent
3657	Calcaterra et al.,	2015	PLoS One. 2015; 10(6):e0125813	non pertinent
3658	Cakir-Özkan et al.,	2015	J of craniofacial surgery. 2015; 26(7):e595-599	non pertinent
3659	Cai et al.,	2015	International j of clinical pharmacology and therapeutics. 2015; 53(9):765-771	non pertinent
3660	Cady et al.,	2015	Headache. 2015; 55(4):529-542	non pertinent
3661	Byrne et al.,	2015	Depression and anxiety. 2015; 32(6):408-414	non pertinent
3662	Butchart et al.,	2015	Neurology. 2015; 84(21):2161-2168	non pertinent
3663	Burton et al.,	2015	American j of medical genetics. Part a. 2015; 167A(10):2272-2281	non pertinent
3664	Burmester et al.,	2015	Annals of the rheumatic diseases. 2015; 74(6):1037-1044	non pertinent

3665	Burfein et al.,	2015	J of sport rehabilitation. 2015; 24(4):405-412	non pertinent
3666	Budde et al.,	2015	Clinical nephrology. 2015; 83(1):11-21	non pertinent
3667	Brunner et al.,	2015	Arthritis & rheumatology (hoboken, N.J.). 2015; 67(5):1377-1385	non pertinent
3668	Bruix et al.,	2015	The lancet. Oncology. 2015; 16(13):1344-1354	non pertinent
3669	Bruehl et al.,	2015	J of Pain. 2015; 16(7):666-675	non pertinent
3670	Broom et al.,	2015	Clinical genitourinary cancer. 2015; 13(1):50-58	non pertinent
3671	Brodaty	2015	International psychogeriatrics. 2015; 27:22-23	non pertinent
3672	Brenke et al.,	2015	European spine j. 2015; 24(12):2832-2840	non pertinent
3673	Brayer et al.,	2015	American j of hematology. 2015; 90(7):602-607	non pertinent
3674	Brandao Filho et al.,	2015	Trials. 2015; 16:415	non pertinent
3675	Bradley et al.,	2015	The lancet. Oncology. 2015; 16(2):187-199	non pertinent
3676	Bradfield et al.,	2015	Pediatric blood & cancer. 2015; 62(6):1004-1010	non pertinent
3677	Boyle et al.,	2015	European j of cancer (Oxford, England : 1990). 2015; 51(6):685-696	non pertinent
3678	Bottelier et al.,	2015	European neuropsychopharmacology. 2015; 25:461-462	non pertinent
3679	Bochicchio et al.,	2015	J of the american college of surgeons. 2015; 220(1):70-81	non pertinent
3680	Blay et al.,	2015	The lancet. Oncology. 2015; 16(5):531-540	non pertinent
3681	Bissonnette et al.,	2015	British j of dermatology. 2015; 172(5):1395-1406	non pertinent
3682	Bilen et al.,	2015	Cancer. 2015; 121(1):69-76	non pertinent
3683	Bhosale et al.,	2015	J Basic Clin Physiol Pharmacol. 2015; 26(3):217-222	non pertinent

3684	Beydon et al.,	2015	Anaesthesia, critical care & pain medicine. 2015; 34(3):165-171	non pertinent
3685	Berwouts et al.,	2015	Radiotherapy and oncology. 2015; 115(2):272-278	non pertinent
3686	Berthelot et al.,	2015	Joint, bone, spine. 2015; 82(6):397-401	non pertinent
3687	Bernhard et al.,	2015	The lancet. Oncology. 2015; 16(7):848-858	non pertinent
3688	Bernasconi et al.,	2015	European neuropsychophar macology. 2015; 25(4):474-482	non pertinent
3689	Berlit et al.,	2015	Archives of gynecology and obstetrics. 2015; 291(3):585-590	non pertinent
3690	Bergman et al.,	2015	BMC Public Health. 2015; 15:632	non pertinent
3691	Bergeron et al.,	2015	American j of respiratory and critical care medicine. 2015; 191(11):1242-1249	non pertinent
3692	Bezon et al.,	2015	Regional anesthesia and pain medicine. Conference: 40th annual regional anesthesia and acute pain medicine meeting of the american society of regional anesthesia and pain medicine. ASRA 2015 las vegas, NV united states. Conference start: 20150514. Conference end: 20150516. Conference publication: (var.pagings); 40(5)	non pertinent
3693	Benson et al.,	2015	Brain Behav Immun. 2015; 48:222-231	non pertinent
3694	Benitez-Camps et al.,	2015	BMC cardiovascular disorders. 2015; 15(1)	non pertinent

3695	Bender Ignacio et al.,	2015	J of infectious diseases. 2015; 212(12):1949-1956	non pertinent
3696	Belkouch et al.,	2015	Pan african medical j. 2015; 20	non pertinent
3697	Beinert et al.,	2015	Eur J Phys Rehabil Med. 2015; 51(6):825-832	non pertinent
3698	Becker et al.,	2015	The lancet. Diabetes & endocrinology. 2015; 3(12):948-957	non pertinent
3699	Bear et al.,	2015	The lancet. Oncology. 2015; 16(9):1037-1048	non pertinent
3700	Bayer et al.,	2015	Obstetrics and gynecology. 2015; 126(1):37-46	non pertinent
3701	Bauer et al.,	2015	American j of tropical medicine and hygiene. 2015; 93(3):441-453	non pertinent
3702	Basgol et al.,	2015	International urogynecology j and pelvic floor dysfunction. 2015; 26(1 SUPPL 1):113	non pertinent
3703	Baron et al.,	2015	Pain practice. 2015; 15(5):455-470	non pertinent
3704	Bannwarth et al.,	2015	Rheumatology (united kingdom). 2015; 54(11):2116-2117	non pertinent
3705	Bamford et al.,	2015	J of psychopharmacology (Oxford, England). 2015; 29(5):634-641	non pertinent
3706	Balta et al.,	2015	American surgeon. 2015; 81(2):182-186	non pertinent
3707	Ball et al.,	2015	Health technology assessment. 2015; 19(12):1-187	non pertinent
3708	Baker et al.,	2015	J Oral Rehabil. 2015; 42(10):742-750	non pertinent
3709	Baker et al.,	2015	European j of integrative medicine. 2015; 7(2):136-142	non pertinent
3710	Bai et al.,	2015	Asian j of andrology. 2015; 17(1):61-67	non pertinent
3711	Bagger-Sjoback et al.,	2015	PLoS One. 2015; 10(3)	non pertinent

3712	Bae et al.,	2015	Spine (Phila Pa 1976). 2015; 40(11):759-766	non pertinent
3713	Avallone et al.,	2015	Oncotarget. 2015; 6(30):30394-30407	non pertinent
3714	Ataei et al.,	2015	Hematological oncology. 2015; 33(2):67-74	non pertinent
3715	Ashford et al.,	2015	Physiotherapy. 2015; 101(1):88-94	non pertinent
3716	Ash et al.,	2015	Kidney international. 2015; 88(2):404-411	non pertinent
3717	Arnold et al.,	2015	Pediatric rheumatology. 2015; 13(1)	non pertinent
3718	Arakane et al.,	2015	Vaccine. 2015; 33(46):6340-6350	non pertinent
3719	Antoun et al.,	2015	European j of cancer (Oxford, England : 1990). 2015; 51(17):2570-2577	non pertinent
3720	Anota et al.,	2015	PLoS One. 2015; 10(5)	non pertinent
3721	Andrews et al.,	2015	N Engl J Med. 2015; 373(25):2403-2412	non pertinent
3722	Andersson et al.,	2015	JAMA Psychiatry. 2015; 72(7):659-667	non pertinent
3723	Anderson et al.,	2015	Drug and alcohol dependence. 2015; 150:170-174	non pertinent
3724	Anagnostou et al.,	2015	Lancet. 2015; 383(9925):1297-1304	non pertinent
3725	Altafini et al.,	2015	Clinical ophthalmology (auckland, N.Z.). 2015; 9:2263-2270	non pertinent
3726	Alsohaibani et al.,	2015	Saudi j of gastroenterology. 2015; 21(4):220-225	non pertinent
3727	Allen et al.,	2015	Physiotherapy (united kingdom). 2015; 101:e55-e56	non pertinent
3728	Allen et al.,	2015	Can J Anaesth. 2015; 62(11):1169-1178	non pertinent
3729	Ali et al.,	2015	BMJ Open. 2015; 5(7):e008254	non pertinent
3730	Alam et al.,	2015	JAMA dermatology. 2015; 151(11):1194-1199	non pertinent

3731	Aktas et al.,	2015	European j of gastroenterology & hepatology. 2015; 27(7):769-775	non pertinent
3732	Akcam et al.,	2015	Saudi medical j. 2015; 36(3):286-290	non pertinent
3733	Aiello et al.,	2015	JAMA ophthalmology. 2015; 133(8):888-896	non pertinent
3734	Aichinger et al.,	2015	Clinical and vaccine immunology. 2015; 22(1):46-55	non pertinent
3735	Actrn – Howe	2015	Australian New Zealand Clinical Trials Registry. 2015	non pertinent
3736	Actrn – Hochsprung	2015	Australian New Zealand Clinical Trials Registry. 2015	non pertinent
3737	Acheson et al.,	2015	Depression and anxiety. 2015; 32(6):400-407	non pertinent
3738	Aboltins et al.,	2015	J of antimicrobial chemotherapy. 2015; 70(2):581-586	non pertinent
3739	Abdulqawi et al.,	2015	Lancet (london, england). 2015; 385(9974):1198-1205	non pertinent
3740	Abdel-Ghaffar et al.,	2015	Clinical otolaryngology. 2015; 40(3):219-226	non pertinent
3741	Abane et al.,	2015	The bone & joint j. 2015; 97-B(1):56-63	non pertinent
3742	Westberg et al.,	2015	Clinical infectious diseases. 2015 Jun; 60(12):1752-1759	non pertinent
3743	Adamson et al.,	2015	Lancet (london, england). 2015; 385(9985):2383-2391	non pertinent
3744	Zhong et al.,	2015	Tohoku j of experimental medicine. 2015 Oct; 237(3):183-191	non pertinent
3745	Wirth et al.,	2015	European urology. 2015 Mar; 67(3):482-491	non pertinent
3746	Budoff et al.,	2015	Coronary artery disease. 2015 Dec; 26(4):301-307	non pertinent

3747	Carbajal et al.,	2015	The lancet respiratory medicine. 2015 Oct; 3(10):796-812	non pertinent
3748	Zeiders et al.,	2015	International j of pediatric otorhinolaryngology. 2015 Dec; 79(12):2416-2423	non pertinent
3749	Bennett et al.,	2015	Drug design, development and therapy. 2015; 9:4639-4647	non pertinent
3750	Attipoe et al.,	2015	Annals of the rheumatic diseases. 2015; 74(SUPPL 2):987	non pertinent
3751	Farnia et al.,	2015	Pharmacopsychiatry. 2015; 48:156-163	non pertinent
3752	Saad et al.,	2015	Lancet oncology. 2015; 16(3):338-348	non pertinent
3753	Panagopoulos et al.,	2015	European j of pain (united kingdom). 2015; 19(7):899-907	non pertinent
3754	Norton et al.,	2015	Spine. 2015; 40(10):726-733	non pertinent
3755	?	2015	Current psychiatry. 2015; 14(3):41-42	non pertinent
3756	SCOT-HEART investogators	2015	Lancet. 2015; 385(9985):2383-2391	non pertinent
3757	Zorzi et al.,	2014	Osteoarthritis and cartilage. 2014; 22:194	non pertinent
3758	Zoric et al.,	2014	British j of anaesthesia. 2014; 112(4):722-728	non pertinent
3759	Zivadinov et al.,	2014	PLoS One. 2014; 9(3)	non pertinent
3760	Zhu et al.,	2014	BMC Complement Alern Med. 2014;	non pertinent
3761	Zhu et al.,	2014	BMC complementary and alternative medicine. 2014; 14	non pertinent
3762	Zhu et al.,	2014	The lancet. Oncology. 2014; 15(6):612-619	non pertinent
3763	Zhu et al.,	2014	New England j of medicine. 2014; 370(9):818-828	non pertinent
3764	Zhao et al.,	2014	PLoS One. 2014; 9(6):e99538	non pertinent
3765	Zhang et al.,	2014	Magn Reson Imaging. 2014; 32(4):359-365	non pertinent

3766	Zhang et al.,	2014	Zhongguo gu shang (China j of orthopaedics and traumatology). 2014; 27(1):38-40	non pertinent
3767	Zhang et al.,	2014	J of clinical pharmacology. 2014; 54(4):415-421	non pertinent
3768	Zhang et al.,	2014	Minimally invasive therapy & allied technologies: MITAT. 2014; 23(3):173-178	non pertinent
3769	Zhang et al.,	2014	J Popul Ther Clin Pharmacol. 2014; 21(3):e346-356	non pertinent
3770	Zhang et al.,	2014	Neurological sciences. 2014; 35(8):1209-1214	non pertinent
3771	Zeng et al.,	2014	Zhongguo xiu fu chong jian wai ke za zhi (Chinese j of reparative and reconstructive surgery). 2014; 28(1):74-78	non pertinent
3772	Zeinoddini et al.,	2014	J of psychiatric research. 2014; 59:125-131	non pertinent
3773	Yuvaraj et al.,	2014	Behavioral and brain functions. 2014; 10(1)	non pertinent
3774	Yu et al.,	2014	J of gastroenterology and hepatology. 2014; 29(6):1200-1206	non pertinent
3775	Yoon et al.,	2014	Neurorehabil Neural Repair. 2014; 28(3):250-259	non pertinent
3776	Yee et al.,	2014	British j of haematology. 2014; 166(3):401-409	non pertinent
3777	Yang et al.,	2014	Statistics in medicine. 2014; 33(20):3547-3555	non pertinent
3778	Yang et al.,	2014	J of international medicine research. 2014; 42(2):307-319	non pertinent
3779	Wynn et al.,	2014	Neuropsychopharmacology. 2014; 39:502	non pertinent
3780	Wylde et al.,	2014	Osteoarthritis and cartilage. 2014; 22:433-	non pertinent
3781	Wu et al.,	2014	Anesthesia and Analgesia. 2014; 119(3):686-692	non pertinent

3782	Wu et al.,	2014	The lancet. Oncology. 2014; 15(2):213-222	non pertinent
3783	Wu et al.,	2014	Brain stimulation. 2014; 7(2):212-218	non pertinent
3784	Wu et al.,	2014	Pediatrics international. 2014; 56(3):315-322	non pertinent
3785	Wu et al.,	2014	Helicobacter. 2014; 19(3):207-213	non pertinent
3786	Wu et al.,	2014	Complementary therapies in medicine. 2014; 22(3):456-462	non pertinent
3787	Wu et al.,	2014	Molecular and clinical oncology. 2014; 2(5):695-700	non pertinent
3788	Wood et al.,	2014	Emotion. 2014; 14(4):693-700	non pertinent
3789	Wojtysiak et al.,	2014	Spine. 39(21):1792-1800	non pertinent
3790	Winter et al.,	2014	J of pediatric gastroenterology and nutrition. 2014; 59(6):767-772	non pertinent
3791	Winhusen et al.,	2014	J of clinical psychiatry. 2014; 75(7):757-764	non pertinent
3792	Winhusen et al.,	2014	J of clinical psychiatry. 2014; 75(4):336-343	non pertinent
3793	Winchester et al.,	2014	J of pharmacology and experimental therapeutics. 2014; 351(2):259-269	non pertinent
3794	Williams et al.,	2014	Lancet (london, england). 2014; 384(9954):1586-1596	non pertinent
3795	Wilke et al.,	2014	The lancet. Oncology. 2014; 15(11):1224-1235	non pertinent
3796	Wilder-Smith et al.,	2014	Neurogastroenterol Motil. 2014; 26(4):489-498	non pertinent
3797	Wigal et al.,	2014	J of child and adolescent psychopharmacology. 2014; 24(10):562-569	non pertinent
3798	Wierckx et al.,	2014	J of sexual medicine. 2014; 11(8):1999-2011	non pertinent
3799	Widemann et al.,	2014	Neuro-oncology. 2014; 16(5):707-718	non pertinent

3800	Wick et al.,	2014	Clinical cancer research. 2014; 20(24):6304-6313	non pertinent
3801	Wibbenmeyer et al.,	2014	J of burn care & research. 2014; 35(2):136-142	non pertinent
3802	Westritschnig et al.,	2014	Human vaccines and immunotherapeutics. 2014; 10(1):2846-2859	non pertinent
3803	Westhoff et al.,	2014	BMC Cancer. 2014; 14(1)	non pertinent
3804	Wepner et al.,	2014	Pain. 2014; 155(2):261-268	non pertinent
3805	Weisstanner et al.,	2014	Neuroradiology. 2014; 56:231-232	non pertinent
3806	Weiss et al.,	2014	Drug Alcohol Depend. 2014; 140:118-122	non pertinent
3807	Weiss et al.,	2014	Pediatr Blood Cancer. 2014; 61(6):982-986	non pertinent
3808	Weisler et al.,	2014	Neuropsychiatric disease and treatment. 2014; 10:1-12	non pertinent
3809	Weiser et al.,	2014	Bipolar disorders. 2014; 16(4):441-447	non pertinent
3810	Weiland et al.,	2014	Acta Radiol. 2014; 55(6):715-724	non pertinent
3811	Webster et al.,	2014	J of drugs in dermatology. 2014; 13(6):665-670	non pertinent
3812	Watz et al.,	2014	The lancet. Respiratory medicine. 2014; 2(1):63-72	non pertinent
3813	Watson et al.,	2014	Molecular therapy. 2014; 22:292	non pertinent
3814	Wang et al.,	2014	American j of cardiovascular drug. 2014; 14(2):147-154	non pertinent
3815	Wang et al.,	2014	J of anesthesia. 2014; 28(5):790-793	non pertinent
3816	Wall et al.,	2014	JAMA neurology. 2014; 71(6):693-701	non pertinent
3817	Vollmer et al.,	2014	J of neurology. 2014; 261(4):773-783	non pertinent
3818	Vollmer et al.,	2014	Pain practice. 2014; 14(8):732-744	non pertinent
3819	Volkmann et al.,	2014	The lancet. Neurology. 2014; 13(9):875-884	non pertinent
3820	Voit et al.,	2014	The lancet. Neurology. 2014; 13(10):987-996	non pertinent

3821	Vlaev et al.,	2014	Health Psychol. 2014; 33(1):66-76	non pertinent
3822	Viscusi et al.,	2014	Clinical J of Pain. 2014; 30(2):102-110	non pertinent
3823	Vinik et al.,	2014	Diabetes care. 2014; 37(8):2302-2309	non pertinent
3824	Vestergaard et al.,	2014	Annals of thoracic surgery. 2014; 97(1):153-160	non pertinent
3825	Verdijk et al.,	2014	Vaccine. 2014; 32(39):4938-4944	non pertinent
3826	Van Tilburg et al.,	2014	Complementary therapies in medicine. 2014; 22(1):17-20	non pertinent
3827	Van Rhee et al.,	2014	The lancet. Oncology. 2014; 15(9):966-974	non pertinent
3828	Van Geest et al.,	2014	BMC musculoskeletal disorders. 2014; 15:129	non pertinent
3829	Van der Velden et al.,	2014	J of infectious diseases. 2014; 209(1):12-23	non pertinent
3830	Van Der Straten et al.,	2014	PLoS One. 2014; 9(2)	non pertinent
3831	Van den Heuvel et al.,	2014	Radiotherapy and oncology. 2014; 110(1):126-131	non pertinent
3832	Van Den Berg et al.,	2014	PLoS One. 2014; 9(10)	non pertinent
3833	Van de Glind et al.,	2014	Trials. 2014; 15(1)	non pertinent
3834	Van Cutsem et al.,	2014	Clinical cancer research. 2014; 20(16):4240-4250	non pertinent
3835	Valentino et al.,	2014	Haemophilia. 2014; 20(3):398-406	non pertinent
3836	Valayannopoulos et al.,	2014	J of hepatology. 2014; 61(5):1135-1142	non pertinent
3837	Umbricht et al.,	2014	Neuropsychopharmacology. 2014; 39(7):1568-1577	non pertinent
3838	Ucmak et al.,	2014	Cutaneous and ocular toxicology. 2014; 33(1):54-59	non pertinent
3839	Turner et al.,	2014	Headache. 2014; 54(2):269-277	non pertinent
3840	Turner et al.,	2014	American j of obstetrics and gynecology. 2014; 211(5):479.e471-479.e413	non pertinent
3841	Turncliff et al.,	2014	Schizophrenia research. 2014; 159(2-3):404-410	non pertinent

3842	Tsukada et al.,	2014	J of bone and joint surgery. American volume. 2014; 96(17):1433-1438	non pertinent
3843	Tse et al.,	2014	European neuropsychopharmacology. 2014; 24(10):1641-1649	non pertinent
3844	Tristram et al.,	2014	The lancet. Oncology. 2014; 15(12):1361-1368	non pertinent
3845	Toth et al.,	2014	Clin J Pain. 2014; 30(2):111-118	non pertinent
3846	Topazio et al.,	2014	BMC urology. 2014; 14(1)	non pertinent
3847	Toosizadeh et al.,	2014	Gerontology. 2014; 61(1):3-14	non pertinent
3848	Tombal et al.,	2014	Lancet oncology. 2014; 15(6):592-600	non pertinent
3849	Toledo Junior et al.,	2014	Revista da sociedade brasileira de medicina tropical. 2014; 47(6):756-762	non pertinent
3850	Tinoco et al.,	2014	Vaccine. 2014; 32(13):1480-1487	non pertinent
3851	Thollot et al.,	2014	Pediatric infectious disease j. 2014; 33(12):1246-1254	non pertinent
3852	Thienthong et al.,	2014	Anaesthesia, pain and intensive care. 2014; 18(1):38-42	non pertinent
3853	Theysohn et al.,	2014	Neurogastroenterol Motil. 2014; 26(12):1743-1753	non pertinent
3854	Theysohn et al.,	2014	J Altern Complement Med. 2014; 20(12):893-900	non pertinent
3855	Thankamony et al.,	2014	J of clinical endocrinology and metabolism. 2015; 99(2):639-647	non pertinent
3856	Tevaarwerk et al.,	2014	J of clinical oncology. 2014; 32(35):3948-3958	non pertinent
3857	Terras et al.,	2014	JAMA dermatology. 2014; 150(6):621-627	non pertinent
3858	Terkelsen et al.,	2014	Anesthesiology. 2014; 120(5):1225-1236	non pertinent
3859	Telch et al.,	2014	Am J Psychiatry. 2014; 171(10):1091-1098	non pertinent

3860	Tedesco Triccas et al.,	2014	Cerebrovascular diseases (basel, switzerland). 2014; 37:59-	non pertinent
3861	Tassorelli et al.,	2014	Parkinsonism & related disorders. 2014; 20(11):1140-1144	non pertinent
3862	Tanaka et al.,	2014	J Clin Monit Comput. 2014; 28(6):561-566	non pertinent
3863	Tan et al.,	2014	American j of ophthalmology. 2014; 157(1):237-247.e231	non pertinent
3864	Tamburella et al.,	2014	Frontiers in human neuroscience. 2014; 8(MAY)	non pertinent
3865	Taieb et al.,	2014	The lancet. Oncology. 2014;15(8):862-873	non pertinent
3866	Syngle et al.,	2014	Neurological sciences. 2014; 35(7):1067-1073	non pertinent
3867	Swenson et al.,	2014	European j of radiology. 2014; 83(4):733-738	non pertinent
3868	Swart et al.,	2014	BMC Musculoskelet Disord. 2014; 15:63	non pertinent
3869	Suzuki et al.,	2014	Neurogastroenterology and motility. 2014; 26(7):950-961	non pertinent
3870	Sulkowski et al.,	2014	New England j of medicine. 2014; 370(3):211-221	non pertinent
3871	Suffoletto et al.,	2014	Annals of emergency medicine. 2014; 64(6):664-672	non pertinent
3872	Strigo et al.,	2014	Translational psychiatry. 2014; 4	non pertinent
3873	Streicher et al.,	2014	J Bodyw Mov Ther. 2014; 18(3):489-496	non pertinent
3874	Strebkova et al.,	2014	Annals of the rheumatic diseases. 2014; 73	non pertinent
3875	Straube et al.,	2014	Transl Psychiatry. 2014; 4:e490	non pertinent
3876	Straube et al.,	2014	Psychother Psychosom. 2014; 83(4):222-233	non pertinent
3877	Stovner et al.,	2014	Cephalalgia. 2014; 34(7):523-532	non pertinent
3878	Stevenson et al.,	2014	Nutrition (Burbank, Los Angeles County, Calif.). 2014; 30(10):1151-1157	non pertinent

3879	Sternlicht et al.,	2014	Local and regional anesthesia. 2014; 7:69-74	non pertinent
3880	Staud et al.,	2014	European j of pain (London, England). 2014; 18(6):803-812	non pertinent
3881	Stasi et al.,	2014	Lung cancer: targets and therapy. 2014; 5:43-50	non pertinent
3882	Srinivasan et al.,	2014	American j of geriatric psychiatry. 2014; 22(3):135-136	non pertinent
3883	Spreen et al.,	2014	J of acquired immune deficiency syndrome (1999). 2014; 67(5):481-486	non pertinent
3884	Spierings et al.,	2014	Pain medicine (united states). 2014; 15(2):264-271	non pertinent
3885	Spick et al.,	2014	Eur J Radiol. 2014; 83(6):980-983	non pertinent
3886	Speierer et al.,	2014	Swiss medical weekly. 2014; 144:13-	non pertinent
3887	Soravia et al.,	2014	Depression and anxiety. 2014; 31(5):429-435	non pertinent
3888	Sood et al.,	2014	European urology, supplements. 2014; 13(1):e1020-e1020b	non pertinent
3889	Song et al.,	2014	Human vaccines & immunotherapeutics. 2014; 10(10):2958-2964	non pertinent
3890	Son et al.,	2014	Stereotact Funct Neurosurg. 2014; 92(4):218-226	non pertinent
3891	Smith et al.,	2014	Obesity (silver spring, md.). 2014; 22(10):2137-2146	non pertinent
3892	Smith et al.,	2014	J of clinical oncology. 2014; 21(30):3391-3399	non pertinent
3893	Slatkovska et al.,	2014	Calcified tissue international. 2014; 95(6):547-556	non pertinent
3894	Sisson et al.,	2014	Alimentary pharmacology & therapeutics. 2014; 40(1):51-62	non pertinent

3895	Singla et al.,	2014	Current medical research and opinion. 2014; 30(3):349-359	non pertinent
3896	Simpson et al.,	2014	Pain. 2014; 155(10):1943-1954	non pertinent
3897	Simon et al.,	2014	Menopause. 2014; 21(6):633-640	non pertinent
3898	Siddiqui et al.,	2014	Pain practice. 2014; 14(2):132-139	non pertinent
3899	Shpaner et al.,	2014	Neuroimage Clin. 2014; 5:365-376	non pertinent
3900	Short et al.,	2014	Anesth Analg. 2014; 118(5):981-986	non pertinent
3901	Shin et al.,	2014	Pediatric critical care medicine. 2014; 15(5):451-455	non pertinent
3902	Sheta et al.,	2014	Paediatric anaesthesia. 2014; 24(2):181-189	non pertinent
3903	Shervin et al.,	2014	American j of emergency medicine. 2014; 32(9):1011-1015	non pertinent
3904	Sheppard et al.,	2014	Investigative ophthalmology & visual science. 2014; 55(5):2993-3002	non pertinent
3905	Shenhav et al.,	2014	Emotion (Washington, D.C.). 2014; 14(2):301-309	non pertinent
3906	Shen et al.,	2014	J of clinical neuroscience. 2014; 21(9):1612-1616	non pertinent
3907	Shen et al.,	2014	Neurorehabilitation and neural repair. 2014; 28(6):524-535	non pertinent
3908	Shen et al.,	2014	Clinical respiratory j. 2014; 8(1):100-107	non pertinent
3909	Shavakhi et al.,	2014	Endoscopy. 2014; 46(8):633-639	non pertinent
3910	Sharma et al.,	2014	J of oral and maxillofacial surgery. 2014; 14(3):611-615	non pertinent
3911	Sharma et al.,	2014	Cardiovascular and interventional radiology. 2014; 37(2):142-143	non pertinent
3912	Sharara et al.,	2014	United european gastroenterology j. 2014; 2(3):179-188	non pertinent
3913	Shapiro	2014	World neurosurg. 2014; 81(5-6):830-835	non pertinent

3914	Shamji et al.,	2014	J of spinal disorders & techniques. 2014; 27(7):370-375	non pertinent
3915	Shakoor et al.,	2014	BMJ Open. 2014; 4(12):e005360	non pertinent
3916	Shah et al.,	2014	Blood. 2014; 123(15):2317-2324	non pertinent
3917	Serour et al.,	2014	Reproductive biology and endocrinology. 2014; 12(1)	non pertinent
3918	Seo et al.,	2014	Clinical and vaccine immunology. 2014; 21(7):989-996	non pertinent
3919	Selvaraj et al.,	2014	Neuropsychopharmacology. 2014; 39:223-224	non pertinent
3920	Seino et al.,	2014	Diabetes, obesity & metabolism. 2014; 16(8):739-747	non pertinent
3921	Scott et al.,	2014	Health technology assessment. 2014; 18(66):i-xxiv+1-164	non pertinent
3922	Schwartz et al.,	2014	Clinical drug investigation. 2014; 35(2):95-108	non pertinent
3923	Schuh-Hofer et al.,	2014	Somnologie. 2014; 18(1 SUPPL 1):74-75	non pertinent
3924	Schmidt-Wilcke et al.,	2014	Neuroimage Clin. 2014; 6:252-261	non pertinent
3925	Schmidt-Erfurth et al.,	2014	Ophthalmology. 2014; 121(1):193-201	non pertinent
3926	Schmidt et al.,	2014	Biol Psychiatry. 2014; 76(4):289-296	non pertinent
3927	Schistad et al.,	2014	Clinical j of pain. 2014; 30(10):869-874	non pertinent
3928	Schadendorf et al.,	2014	Annals of oncology: official j of the european society for medical oncology. 2014; 25(3):700-706	non pertinent
3930	Satoh et al.,	2014	J of clinical oncology. 2014; 32(19):2039-2049	non pertinent
3931	Satoh et al.,	2014	Oncologist. 2014; 19(7):712-719	non pertinent
3932	Sarchielli et al.,	2014	European neuropsychopharmacology. 2014; 24(8):1289-1297	non pertinent

3933	Sandvik et al.,	2014	European j of pain (united kingdom). 2014; 18(10):1490-1500	non pertinent
3934	San-Miguel et al.,	2014	The lancet. Oncology. 2014; 15(11):1195-1206	non pertinent
3935	Samarbakhsh et al.,	2014	Global spine j. 2014; 4	non pertinent
3936	Sallinen et al.,	2014	BMC surgery. 2014; 14:77	non pertinent
3937	Sakrajai et al.,	2014	Clinical J of Pain. 2014; 30(12):1076-1083	non pertinent
3938	Sahinovic et al.,	2014	Anesth Analg. 2014; 119(2):288-301	non pertinent
3939	Saccomanno et al.,	2014	Arthroscopy – j of arthroscopic and related surgery. 2014; 30(11):1491-1498	non pertinent
3940	Saari et al.,	2014	Hip international. 2014; 24(1):49-55	non pertinent
3941	Ryan et al.,	2014	BMC Musculoskelet Disord. 2014; 15:59	non pertinent
3942	Ruz et al.,	2014	Investigative ophthalmology and visual science. Conference: 2014 annual meeting of the association for research in vision and ophthalmology, AROV 2014. United states. 2014; 55(13):3673	non pertinent
3943	Ruetzler et al.,	2014	J of anesthesia. 2014; 28(4):580-586	non pertinent
3944	Rubel et al.,	2014	British j of dermatology. 2014; 171(5):1164-1171	non pertinent
3945	Rowley et al.,	2014	Blood. 2014; 123(20):3101-3104	non pertinent
3946	Roue et al.,	2014	Achrives of disease in childhood. 2014; 99:A163	non pertinent
3947	Rothgange et al.,	2014	Pain practice. 2014; 14:99-	non pertinent
3948	Roth et al.,	2014	International j of cardiology. 2014; 176(1):55-61	non pertinent
3949	Roth et al.,	2014	American j of clinical nutrition. 2014; 100(6):1587-1595	non pertinent

3950	Rodriguez-Torres et al.,	2014	Annals of hepatology. 2014; 13(4):364-375	non pertinent
3951	Roddy et al.,	2014	BMC musculoskeletal disorders. 2014; 15(1)	non pertinent
3952	Robinson et al.,	2014	Jama. 2014; 311(18):1870-1882	non pertinent
3953	Robbie et al.,	2014	Infectious diseases and therapy. 2014; 3(2):203-214	non pertinent
3954	Riquelme et al.,	2014	BMC Neuroscience. 2014; 15:10	non pertinent
3955	Rice et al.,	2014	Lancet (london, england). 2014; 383(9929):1637-1647	non pertinent
3956	Ribeiro et al.,	2014	J Orthop Sports Phys Ther. 2014; 44(9):702-711	non pertinent
3957	Ribas et al.,	2014	J of clinical pharmacology. 2014; 54(4):368-374	non pertinent
3958	Resik et al.,	2014	Vaccine. 2014; 32(42):5399-5404	non pertinent
3959	Rempe et al.,	2014	PLoS One. 2014; 9(11):e112325	non pertinent
3960	Reisinger et al.,	2014	Human vaccines and immunotherapeutics. 2014; 10(8):2395-2407	non pertinent
3961	Reif et al.,	2014	Mol Psychiatry. 2014; 19(1):122-128	non pertinent
3962	Reed et al.,	2014	Obstetrics and gynecology. 2014; 124(2 Pt 1):233-241	non pertinent
3963	Reddy et al.,	2014	Oral oncology. 2014; 50(5):498-505	non pertinent
3964	Reck et al.,	2014	The lancet. Oncology. 2014; 15(2):143-155	non pertinent
3965	Read et al.,	2014	Lancet (london, england). 2014; 384(9960):2123-2131	non pertinent
3966	Ravn et al.,	2014	Basic & clinical pharmacology & toxicology. 2014; 115(3):257-267	non pertinent
3967	Raskin et al.,	2014	Clinical J of Pain. 2014; 30(5):379-390	non pertinent
3968	Raskin et al.,	2014	Gastroenterology. 2014; 147(4):793-802	non pertinent

3969	Ramtvedt et al.,	2014	J of child and adolescent psychopharmacology. 2014; 24(3):130-139	non pertinent
3970	Ramo-Tello et al.,	2014	Multiple sclerosis (houndmills, basingstoke, england). 2014; 20(6):717-725	non pertinent
3971	Raksakietisak et al.,	2014	Chotmaihet thangphaet (J of the Medical Association of Thailand). 2014; 97(5):530-535	non pertinent
3972	Rajan et al.,	2014	Lancet oncology. 2014; 15(2):191-200	non pertinent
3973	Rahe-Meyer et al.,	2014	Anesthesiology. 2014; 121(5):969-977	non pertinent
3974	Raftopoulos et al.,	2014	Supportive care in cancer. 2014; 23(3):723-732	non pertinent
3975	Radnovich et al.,	2014	J of pain research. 2014; 7:727-735	non pertinent
3976	Raby et al.,	2014	The american j on addictions. 2014; 23(1):68-75	non pertinent
3977	Rabinak et al.,	2014	Neurobiol Learn Mem. 2014; 113:125-134	non pertinent
3978	Querellou et al.,	2014	Clin Nucl Med. 2014; 39(1):8-13	non pertinent
3979	Quaedflieg et al.,	2014	J Psychopharmacol. 2014; 28(1):39-48	non pertinent
3980	Qi et al.,	2014	Regional anesthesia and pain medicine. 2014; 39(3):208-213	non pertinent
3981	Pozzi et al.,	2014	Eur J Oral Implantol. 2014; 7(3):229-242	non pertinent
3982	Powell et al.,	2014	J Magn Reson Imaging. 2014; 39(2):419-426	non pertinent
3983	Pourmoghaddas et al.,	2014	Biomed research international. 2014:191026	non pertinent
3984	Post et al.,	2014	Neuropsychopharmacology. 2014; 39:346-647	non pertinent
3985	Porsteinsson et al.,	2014	JAMA-journal of the american medical association. 2014; 311(7):682-691	non pertinent
3986	Popova et al.,	2014	Neuroimage Clin. 2014; 6:156-165	non pertinent

3987	Poonai et al.,	2014	CMAJ: Canadian Medical Association j. 2014; 186(18):1358-1363	non pertinent
3988	Poder et al.,	2014	Vaccine. 2014; 32(9):1121-1129	non pertinent
3989	Pistilli et al.,	2014	Eur J Oral Implantol. 2014; 7(2):153-171	non pertinent
3990	Pinkerton et al.,	2014	J of clinical endocrinology and metabolism. 2014; 99(2):E189-E198	non pertinent
3991	Pinkerton et al.,	2014	J of women's health (2002). 2014; 23(1):18-28	non pertinent
3992	Pimentel et al.,	2014	Digestive diseases and sciences. 2014; 59(6):1278-1285	non pertinent
3993	Pignata et al.,	2014	The lancet. Oncology. 2014; 15(4):396-405	non pertinent
3994	Phan	2014	Biological psychiatry. 2014; 75(9 SUPPL 1):27	non pertinent
3995	Pfleiderer et al.,	2014	J of neural transmission. 2014; 121(11):1417-1428	non pertinent
3996	Petersen et al.,	2014	Pain. 2014; 155(5):929-936	non pertinent
3997	Peng et al.,	2014	PLoS One. 2014; 9(3):e91052	non pertinent
3998	Pellegrino et al.,	2014	J of spinal disorders & techniques. 2014; 27(8):409-414	non pertinent
3999	Pekyavas et al.,	2014	Fizyoterapi rehabilitasyon. 2014; 25(1 SUPPL 1):31	non pertinent
4000	Pedersen et al.,	2014	Pain. 2014; 155(5):881-888	non pertinent
4001	Pecina et al.,	2014	J of neuroscience. 2014; 34(17):5874-5881	non pertinent
4002	Pecina et al.,	2014	Mol Psychiatry. 2014; 19(3):385-391	non pertinent
4003	Paulsen et al.,	2014	J of clinical oncology. 2014; 32(29):3221-3228	non pertinent
4004	Pattee et al.,	2014	Current medical research and opinion. 2014; 30(11):2255-2265	non pertinent
4005	Patel et al.,	2014	Diabetes technology & therapeutics. 2014; 16(1):15-19	non pertinent

4006	Papaconstantinou et al.,	2014	Cutaneous and ocular toxicology. 2014; 33(3):173-180	non pertinent
4007	Paech et al.,	2014	Anaesthesia and intensive care. 2014; 42(1):15-22	non pertinent
4008	Padhi et al.,	2014	J of clinical pharmacology. 2014; 54(2):168-178	non pertinent
4009	Oxlund et al.,	2014	J of the american society of hypertension : JASH. 2014; 8(12):872-881	non pertinent
4010	Owonikoko et al.,	2014	Cancer chemotherapy and pharmacology. 2014; 73(1):171-180	non pertinent
4011	Ory-Magne et al.,	2014	Neurology. 2014; 82(4):300-307	non pertinent
4012	Olsen et al.,	2014	Osteoporos Int. 2014; 25(8):2017-2025	non pertinent
4013	Olivares et al.,	2014	British j of nutrition. 2014; 112(1):30-40	non pertinent
4014	Olischar et al.,	2014	Paediatr Anaesth. 2014; 24(11):1149-1157	non pertinent
4015	Olanow et al.,	2014	The lancet. Neurology. 2014; 13(2):141-149	non pertinent
4016	Okihara et al.,	2014	International j of urology. 2014; 21(3):271-276	non pertinent
4017	Oh et al.,	2014	J of gastroenterology and hepatology (australia). 2014; 29(6):1171-1176	non pertinent
4018	Ogutu et al.,	2014	Malaria j. 2014; 13(1)	non pertinent
4019	Ogino et al.,	2014	Anesth Analg. 2014; 118(6):1317-1325	non pertinent
4020	Oertel et al.,	2014	Clin Pharmacol Ther. 2014; 96(6):684-693	non pertinent
4021	O'Riordan et al.,	2014	J of aerosol medicine and pulmonary drug delivery. 2014; 27(3):200-208	non pertinent
4022	Novotna et al.,	2014	Clinical therapeutics. 2014; 36(3):357-367	non pertinent
4023	Noveck et al.,	2014	J of the american heart association. 2014; 3(4)	non pertinent

4024	Norman et al.,	2014	Vaccine. 2014; 32(16):1856-1862	non pertinent
4025	Nolte et al.,	2014	Annals of allergy, asthma and immunology. 2014; 113(1):93-100	non pertinent
4026	Nistal-Nuno et al.,	2014	F1000research. 2014; 3	non pertinent
4027	Niesters et al.,	2014	Brain Connect. 2014; 4(6):404-416	non pertinent
4028	Nielsen et al.,	2014	Physical therapy. 2014; 94(2):197-209	non pertinent
4029	Newby et al.,	2014	Lancet (london, england). 2014; 384(9949):1187-1195	non pertinent
4030	Nelson et al.,	2014	J Vasc Surg. 2014; 59(5):1181-1193	non pertinent
4031	Nct – Vendittoli	2014	National Library of Medicine. 2014	non pertinent
4032	Nct – Moss et al.,	2014	JACC Cardiovasc Imaging. 2019 Aug; pii:1936-878X(19)30557-1	non pertinent
4033	Nct – Lindberg-Larsen	2014	National Library of Medicine. 2014	non pertinent
4034	Nct – Andersen	2014	National Library of Medicine. 2014	non pertinent
4035	Nct – Imhof et al.,	2014	Int Sch Res Notices. 2015 Feb; 2015:785497	non pertinent
4036	Nct – Breebaart	2014	National Library of Medicine. 2014	non pertinent
4037	Nct – Rodríguez	2014	National Library of Medicine. 2014	non pertinent
4038	Nct – Black	2014	National Library of Medicine. 2014	non pertinent
4039	Nct – University of Alberta	2014	National Library of Medicine. 2014	non pertinent
4040	Nct – Irwin	2014	National Library of Medicine. 2014	non pertinent
4041	Nct – Drew	2014	National Library of Medicine. 2014	non pertinent
4042	Nct – Nair	2014	National Library of Medicine. 2014	non pertinent
4043	Nct – Mendonca	2014	National Library of Medicine. 2014	non pertinent
4044	Nct – Rabinak	2014	National Library of Medicine. 2014	non pertinent
4045	Nct – Ramasubbu	2014	National Library of Medicine. 2014	non pertinent
4046	Nct – Fisher et al.	2014	Diabetes Care. 2018 Sep; 41(9):1862-1869	non pertinent
4047	Nct – Turhanoglu	2014	National Library of Medicine. 2014	non pertinent
4048	Nct – Huang	2014	National Library of Medicine. 2014	non pertinent
4049	Nct – Waxman	2014	National Library of Medicine. 2014	non pertinent
4050	Nct – Choi	2014	National Library of Medicine. 2014	non pertinent

4051	Nct – Meldrum	2014	National Library of Medicine. 2014	non pertinent
4052	Nct – Del Coso	2014	National Library of Medicine. 2014	non pertinent
4053	Nct – Feitosa et al.,	2014	Rev Assoc Med Bras. 2019 Mar; 65(3):316-318	non pertinent
4054	Nct – Sly	2014	National Library of Medicine. 2014	non pertinent
4055	Nct – Sung	2014	National Library of Medicine. 2014	non pertinent
4056	Nct – Stevens-Lapsley	2014	National Library of Medicine. 2014	non pertinent
4057	Nct – Buccino	2014	National Library of Medicine. 2014	non pertinent
4058	Nct – Braten et al.,	2014	BMJ. 2019 Oct; 367:I5654	non pertinent
4059	Nct – Mendonca et al.,	2014	Eur J Anaesthesiol. 2020 Mar; 37(3):224-234	non pertinent
4060	Nct – Khemiri et al.,	2014	Alcohol Clin Exp Res. 2019 Jan; 43(1):135-146	non pertinent
4061	Nct – Elbogen	2014	National Library of Medicine. 2014	non pertinent
4062	Nct – Aframian	2014	National Library of Medicine. 2014	non pertinent
4063	Nct – Asarnow	2014	National Library of Medicine. 2014	non pertinent
4064	Nct – Pigeon	2014	National Library of Medicine. 2014	non pertinent
4065	Nct – Apkarian	2014	National Library of Medicine. 2014	non pertinent
4066	Nct – Poulin	2014	National Library of Medicine. 2014	non pertinent
4067	Nct – Reckziegel et al.,	2014	BMJ Open. 2017 Jun; 7(6):e014013	non pertinent
4068	Nct – Pfizer	2014	National Library of Medicine. 2014	non pertinent
4069	Nct – Allen	2014	National Library of Medicine. 2014	non pertinent
4070	Nct – Weiner	2014	National Library of Medicine. 2014	non pertinent
4071	Nct – Keith	2014	National Library of Medicine. 2014	non pertinent
4072	Nct – Chang	2014	National Library of Medicine. 2014	non pertinent
4073	Nct – Graco et al.,	2014	Thorax. 2018 Sep; 73(9):864-871	non pertinent
4074	Nct – Lepage	2014	National Library of Medicine. 2014	non pertinent
4075	Nct – Mahajan	2014	National Library of Medicine. 2014	non pertinent
4076	Nct – Adamson	2014	National Library of Medicine. 2014	non pertinent
4077	Nct – Vereecke	2014	National Library of Medicine. 2014	non pertinent
4078	Nct – Sharon	2014	National Library of Medicine. 2014	non pertinent
4079	Navarro Coy et al.,	2014	BMC musculoskeletal disorders. 2014; 15:452	non pertinent

4080	Nasser et al.,	2014	Arthritis care & research. 2014; 66(2):293-300	non pertinent
4081	Narouze et al.,	2014	Regional anesthesia and pain medicine. 2014; 39(5 SUPPL 1):e112-e116	non pertinent
4082	Naoumova et al.,	2014	Swedish dental j. Supplement. 2014; 234:7-118	non pertinent
4083	Nankabirwa et al.,	2014	Clinical infectious diseases. 2014; 58(10):1404-1412	non pertinent
4084	Nam et al.,	2014	Clinical rheumatology. 2014; 33(12):1807-1814	non pertinent
4085	Nam et al.,	2014	Annals oft he rheumatic diseases. 2014; 73(1):75-85	non pertinent
4086	Nakae et al.,	2014	European j of anaesthesiology. 2014; 31:122	non pertinent
4087	Nagata et al.,	2014	Eur J Radiol. 2014; 83(12):2172-2176	non pertinent
4088	Nagahara et al.,	2014	Scandinavian j of gastroenterology. 2014; 49(4):409-417	non pertinent
4089	Naderer et al.,	2014	Antimicrobial agents and chemotherapy. 2014; 58(1):419-423	non pertinent
4090	Murdoch et al.,	2014	International j of clinical pharmacology and therapeutics. 2014; 52(4):267-276	non pertinent
4091	Mungee et al.,	2014	Neuroreport. 2014; 25(7):480-484	non pertinent
4092	Mundt et al.,	2014	Sleep. 2014; 37:A266	non pertinent
4093	Mugie et al.,	2014	Gastroenterology. 2014; 147(6):1285-1295.e1281	non pertinent
4094	Mudaliar et al.,	2014	Diabetes technology & therapeutics. 2014; 16(3):137-144	non pertinent
4095	Motzer et al.,	2014	The lancet. Oncology. 2014; 15(3):286-296	non pertinent
4096	Motomura et al.,	2014	BMC neuroscience. 2014; 15(1)	non pertinent
4097	Moss et al.,	2014	Paediatric anaesthesia. 2014; 24(5):483-489	non pertinent

4098	Moser et al.,	2014	AJNR. American j of neuroradiology. 2014; 35(8):1467-1474	non pertinent
4099	Moosmayer et al.,	2014	J Bone Joint Surg Am. 2014; 96(18):1504-1514	non pertinent
4100	Montaser-Kouhsari et al.,	2014	J of dermatological treatment. 2014; 25(4):337-341	non pertinent
4101	Monk et al.,	2014	The lancet. Oncology. 2014; 15(8):799-808	non pertinent
4102	Monga et al.,	2014	International Journal of Clinical and Experimental Hypnosis. 2014; 62(2): 164-178	non pertinent
4103	Moen et al.,	2014	British j of sports medicine. 2014; 48(18):1358-1363	non pertinent
4104	Mischoulon et al.,	2014	J of clinical psychiatry. 2014; 75(4):370-376	non pertinent
4105	Miokovic et al.,	2014	Med Sci Sports Exerc. 2014; 46(8):1506-1516	non pertinent
4106	Miller et al.,	2014	Clinical j of Pain. 2014; 30(12):1015-1022	non pertinent
4107	Miller et al.,	2014	Physiol Behav. 2014; 124:8-14	non pertinent
4108	Miki et al.,	2014	Psycho-oncology. 2014; 23(8):906-913	non pertinent
4109	Miehlke et al.,	2014	Gastroenterology. 2014; 146(5):1222-1230.e1222	non pertinent
4110	Middleton et al.,	2014	The lancet. Oncology. 2014; 15(8):829-840	non pertinent
4111	Mickelson et al.,	2014	J of hand surgery. 2014; 39(10):1933-1941	non pertinent
4112	Michalak et al.,	2014	Current medical research and opinion. 2014; 30(4):711-718	non pertinent
4113	Mesa et al.,	2014	Haematologica. 2014; 99(2):292-298	non pertinent
4114	Merz et al.,	2014	Soc Cogn Affect Neurosci. 2014; 9(9):1426-1434	non pertinent
4115	Menzies et al.,	2014	J of behavioral medicine. 2014; 37(1):70-80	non pertinent
4116	Menz et al.,	2014	BMC musculoskeletal disorders. 2014; 15:86	non pertinent
4117	Meissner et al.,	2014	Anesthesiology. 2014; 121(6):1281-1291	non pertinent

4118	Meier et al.,	2014	Osteoporosis international. 2014; 25(1):359-366	non pertinent
4119	McQuaid et al.,	2014	J of pain. 2014; 15(4 SUPPL 1):108	non pertinent
4120	McNicholl et al.,	2014	Gut. 2014; 63(2):244-249	non pertinent
4121	McManus et al.,	2014	Pediatric blood & cancer. 2014; 61(5):815-820	non pertinent
4122	McLeod et al.,	2014	British j of anaesthesia. 2014; 113(5):875-883	non pertinent
4123	McKeever et al.,	2014	Paediatr Anaesth. 2014; 24(7):766-773	non pertinent
4124	McKavanagh et al.,	2014	Irish j of medical science. 2014; 183(8):420-421	non pertinent
4125	McIntyre et al.,	2014	Arthritis and rheumatology. 2014; 66(2):451-460	non pertinent
4126	McGrouther et al.,	2014	Current medical research and opinion. 2014; 30(4):733-739	non pertinent
4127	McGlinchey et al.,	2014	Brain injury. 2014; 28(5-6):624-625	non pertinent
4128	McDonald et al.,	2014	BMC Cancer. 2014; 14:264	non pertinent
4129	McCrae et al.,	2014	Sleep. 2014; 37:A176-A177	non pertinent
4130	McClinton et al.,	2014	Trials. 2014; 15:238	non pertinent
4131	Mayer et al.,	2014	Gut. 2014; 63(3):442-450	non pertinent
4132	Mauger et al.,	2014	Experimental physiology. 2014; 99(1):164-171	non pertinent
4133	Mattia et al.,	2014	Neuromodulation. 2014; 17(3):e29-e30	non pertinent
4134	Matro et al.,	2014	Alimentary pharmacology & therapeutics. 2014; 40(6):610-619	non pertinent
4135	Mataix-Cols et al.,	2014	British j of psychiatry. 2014; 204(1):77-78	non pertinent
4136	Masala et al.,	2014	Cardiovasc Intervent Radiol. 2014; 37(2):476-481	non pertinent
4137	Martinsen et al.,	2014	PLoS One. 2014; 9(9):e108637	non pertinent
4138	Martin et al.,	2014	J of women's health. 2014; 23(5):389-396	non pertinent

4139	Martin et al.,	2014	International j of clinical pharmacology and therapeutics. 2014; 53(4):301-316	non pertinent
4140	Martin et al.,	2014	Anesthesiology. 2014; 121(4):740-752	non pertinent
4141	Marques et al.,	2014	Frontiers in human neuroscience. 2014; 8(AUG)	non pertinent
4142	Marks et al.,	2014	Trials. 2014; 15:503	non pertinent
4143	Margaret et al.,	2014	BMJ Open. 2014; 4(10)	non pertinent
4144	Marek et al.,	2014	Alzheimer's & dementia. 2014; 10(5 Suppl):364-373	non pertinent
4145	Marciniak et al.,	2014	World j of gastroenterology. 2014; 20(43):16323-16333	non pertinent
4146	Mannick et al.,	2014	Science translational medicine. 2014; 6(268):268ra179	non pertinent
4147	Manitpisitkul et al.,	2014	Epilepsy research. 2014; 108(9):1519-1532	non pertinent
4148	Manchikanti et al.,	2014	Pain Physician. 2014; 17(1):E61-74	non pertinent
4149	Mamolo et al.,	2014	J Eur Acad Dermatol Venereol. 2014; 28(2):192-203	non pertinent
4150	Mamell et al.,	2014	Rheumatology international. 2014; 34(8):1047-1052	non pertinent
4151	Maksimow et al.,	2014	PLoS One. 2014; 9(11):e113616	non pertinent
4152	Maintz et al.,	2014	J of tropical medicine. 2014	non pertinent
4153	Mainardi et al.,	2014	J of Pain. 2014; 15(4):388-394	non pertinent
4154	Maheu et al.,	2014	Annals of the rheumatic diseases. 2014; 73(2):376-384	non pertinent
4155	Macdonald et al.,	2014	Medicine and science in sports and exercise. 2014; 46(1):131-142	non pertinent
4156	Lumba-Brown et al.,	2014	Pediatric emergency care. 2014; 30(3):139-145	non pertinent

4157	Lueken et al.,	2014	Psychol Med. 2014; 44(2):381-394	non pertinent
4158	Lueken et al.,	2014	European neuropsychopharmacology. 2014; 24:142-	non pertinent
4159	Ludidi et al.,	2014	Neurogastroenterology and motility. 2014; 26(5):705-714	non pertinent
4160	Luciano et al.,	2014	J of the experimental analysis of behavior. 2014; 101(1):94-111	non pertinent
4161	Lotvall et al.,	2014	J of negative results in biomedicine. 2014; 13(1)	non pertinent
4162	Lonsdorf et al.,	2014	Soc Cogn Affect Neurosci. 2014; 9(12):1973-1983	non pertinent
4163	Lonsdorf et al.,	2014	Psychopharmacology (Berl). 2014; 231(9):1949-1962	non pertinent
4164	Lohmander et al.,	2014	Arthritis & rheumatology (hoboken, N.J.). 2014; 66(7):1820-1831	non pertinent
4165	Lockwood et al.,	2014	Fertility and sterility. 2014; 101(1):112-119.e113	non pertinent
4166	Lluch et al.,	2014	J Manipulative Physiol Ther. 2014; 37(9):660-666	non pertinent
4167	Llanos-Cuentas et al.,	2014	Lancet (london, england). 2014; 383(9922):1049-1058	non pertinent
4168	Liu et al.,	2014	Menopause (New York, N.Y.). 2014; 21(6):653-660	non pertinent
4169	Liu et al.,	2014	Gut. 2014; 63(9):1410-1415	non pertinent
4170	Liu et al.,	2014	Clinical interventions in aging. 2014; 9:341-350	non pertinent
4171	Liu et al.,	2014	J of palliative medicine. 2014; 17(7):837-840	non pertinent
4172	Liu et al.,	2014	Chinese medical j. 2014; 127(5):825-829	non pertinent
4173	Ling et al.,	2014	Chinese medical j. 2014; 127(5):905-910	non pertinent
4174	Lindsay et al.,	2014	Trials. 2014; 15:12	non pertinent

4175	Lin et al.,	2014	International J of Psychophysiology. 2014; 94(3):292-297	non pertinent
4176	Lin et al.,	2014	J of child and adolsescent psychopharmacology. 2014; 24(4):190-200	non pertinent
4177	Lim et al.,	2014	J of bone and joint surgery. American volume. 2014; 96(7):597-602	non pertinent
4178	Lim et al.,	2014	BMC infectious diseases. 2014; 14:530	non pertinent
4179	Liebowitz et al.,	2014	American j of psychiatry. 2014; 171(6):675-682	non pertinent
4180	Libri et al.,	2014	British j of anaesthesia. 2014; 112(2):337-347	non pertinent
4181	Liao et al.,	2014	Chinese j of contemporary pediatrics. 2014; 16(5):508-512	non pertinent
4182	Liang et al.,	2014	Human vaccines & immunotherapeutics. 2014; 10(5):1382-1390	non pertinent
4183	Li et al.,	2014	Human vaccines & immunotherapeutics. 2014; 10(3):557-565	non pertinent
4184	Li et al.,	2014	J of endourology/Endourology Society. 2014; 28(7):881-886	non pertinent
4185	Li et al.,	2014	New England j of medicine. 2014; 370(9):829-837	non pertinent
4186	Li et al.,	2014	Chinese j of tissue engineering research. 2014; 18(34):5561-5565	non pertinent
4187	Lewis et al.,	2014	J of bone and mineral research. 2014; 29(11):2489-2497	non pertinent
4188	Levy et al.,	2014	J of clinical oncology. 2014; 32(17):1797-1803	non pertinent
4189	Levy et al.,	2014	American j of transplantation. 2014; 14(3):635-646	non pertinent
4190	Levy et al.,	2014	Annals of oncology. 2014; 25(12):2351-2356	non pertinent

4191	Levy et al.,	2014	J of thoracic oncology. 2014; 9(7):1031-1035	non pertinent
4192	Letko et al.,	2014	Female pelvic medicine & reconstructive medicine. 2014; 20:291-292	non pertinent
4193	Leownorasate et al.,	2014	Chotmaihet thangphaet (J of the Medical Assosciation of Thailand). 2014; 97(12):1332-1337	non pertinent
4194	Lelic et al.,	2014	Neuropharmacology. 2014; 77:422-427	non pertinent
4195	Lei et al.,	2014	Hua zhong ke ji da xue xue bao. Yi xue Ying De wen ban (J of Huazhong University of Science and Technology. Medical sciences). 2014; 34(4):616-620	non pertinent
4196	Lee et al.,	2014	J of clinical endocrinology and metabolism. 2014; 99(9):3153-3159	non pertinent
4197	Lee et al.,	2014	Eur Neuropsychopharmacology. 2014; 24(9):1483-1491	non pertinent
4198	Lee et al.,	2014	Respiratory medicine. 2014; 109(1):54-62	non pertinent
4199	Lee et al.,	2014	Scandinavian j of gastroenterology. 2014; 49(8):917-924	non pertinent
4200	Lee et al.,	2014	Investigational new drugs. 2014; 32(3):561-568	non pertinent
4201	Lee et al.,	2014	Yonsei medical j. 2014; 55(4):928-936	non pertinent
4202	Lee et al.,	2014	J Shoulder Elbow Surg. 2014; 23(3):e53-60	non pertinent
4203	Lee et al.,	2014	New England j of medicine. 2014; 370(25):2377-2386	non pertinent
4204	Lee et al.,	2014	Endoscopy. 2014; 46(4):291-298	non pertinent
4205	Lee et al.,	2014	J of the National Cancer Institute. 2014; 106(12)	non pertinent

4206	Lean et al.,	2014	International j of obesity (2005). 2014; 38(5):689-697	non pertinent
4207	Le Lamer et al.,	2014	J of translational medicine. 2014; 12(1)	non pertinent
4208	Le Guen et al.,	2014	Anesthesiology. 2014; 120(2):355-364	non pertinent
4209	Lawitz et al.,	2014	Lancet (london, england). 2014; 383(9916):515-523	non pertinent
4210	Laursen et al.,	2014	The lancet. Respiratory medicine. 2014; 2(8):638-646	non pertinent
4211	Lauretti et al.,	2014	The korean j of pain. 2014; 27(4):345-352	non pertinent
4212	Laurà et al.,	2014	Muscle & nerve. 2014; 50(3):366-371	non pertinent
4213	Latner et al.,	2014	Appetite. 2014; 77:77-82	non pertinent
4214	Larsen et al.,	2014	Breast cancer: targets and therapy. 2014; 6:179-189	non pertinent
4215	Langer et al.,	2014	J of clinical oncology. 2014; 32(19):2059-2066	non pertinent
4216	Landis et al.,	2014	BMC Urol. 2014; 14:58	non pertinent
4217	Lamothe et al.,	2014	PLoS One. 2014; 9(6)	non pertinent
4218	Lalla et al.,	2014	Oral Oncol. 2014; 50(11):1098-1103	non pertinent
4219	Lai et al.,	2014	Gut. 2014; 63(6):996-1004	non pertinent
4220	Labrie et al.,	2014	J of sexual medicine. 2014; 11(7):1766-1785	non pertinent
4221	La Cesa et al.,	2014	Magn Reson Imaging. 2014; 32(3):236-240	non pertinent
4222	Kwon et al.,	2014	The lancet. Oncology. 2014; 15(7):700-712	non pertinent
4223	Kutluk et al.,	2014	European j of gastroenterology & hepatology. 2014; 26(11):1202-1208	non pertinent
4224	Kurganova et al.,	2014	Zhurnal nevrologii i psikhiatrii imeni S.S. Korsakova. 2014; 114(6):31-37	non pertinent

4225	Kumar et al.,	2014	JAMA dermatology. 2014; 150(10):1072-1078	non pertinent
4226	Krysiak et al.,	2014	Cardiovascular therapeutics. 2014; 32(2):40-46	non pertinent
4227	Kruis et al.,	2014	Gastroenterology. 2014; 146(5 SUPPL 1):-187	non pertinent
4228	Kroes et al.,	2014	Neuroimage. 2014; 84:825-832	non pertinent
4229	Krenk et al.,	2014	J of clinical sleep medicine. 2014; 10(3):321-326	non pertinent
4230	Kowdley et al.,	2014	New England j of medicine. 2014; 370(3):222-232	non pertinent
4231	Kowdley et al.,	2014	New England j of medicine. 2014; 370(20):1879-1888	non pertinent
4232	Kosugi et al.,	2014	J of pain and symptom management. 2014; 47(6):990-1000	non pertinent
4233	Korkmaz et al.,	2014	Paediatrics and international child health. 2014; 34(3):198-202	non pertinent
4234	Koceja et al.,	2014	J of rehabilitation research and development. 2014; 51(8):1331-1338	non pertinent
4235	Knoop et al.,	2014	Arthritis care & research. 2014; 66(1):63-68	non pertinent
4236	Kline et al.,	2014	Circulation. Cardiovascular imaging. 2014; 7(1):66-73	non pertinent
4237	Klein et al.,	2014	J of affective disorders. 2014; 166:93-97	non pertinent
4238	Kita et al.,	2014	Clinical therapeutics. 2014; 36(12):1958-1971	non pertinent
4239	Kim et al.,	2014	J of oral and maxillofacial surgery. 2014; 72(7):1281-1290	non pertinent
4240	Kim et al.,	2014	Spine j. 2014; 14(8):1559-1566	non pertinent
4241	Kim et al.,	2014	J Clin Monit Comput. 2014; 28(4):371-376	non pertinent
4242	Kim et al.,	2014	Gut and liver. 2014; 8(4):371-379	non pertinent

4243	Kim et al.,	2014	Human vaccines & immunotherapeutics. 2014; 10(9):2656-2663	non pertinent
4244	Kibuuka et al.,	2014	Lancet. 2014; 385(9977):1545-1554	non pertinent
4245	Khodayari-Rostamabad et al.,	2014	Conf Proc IEEE Eng Med Biol Soc; 2014:4290-4293	non pertinent
4246	Khodaie-Ardakani et al.,	2014	Psychiatry research. 2014; 215(3):540-546	non pertinent
4247	Khan et al.,	2014	J canadien d'anesthesie (Canadian j of anaesthesia). 2014; 61(3):242-248	non pertinent
4248	Kessner et al.,	2014	PLoS One. 2014; 9(9):e109014	non pertinent
4249	Kct – Ki-Ok	2014	CRIS – Clinical Research Information Service. 2014	non pertinent
4250	Kaufman et al.,	2014	Human gene therapy. 2014; 25(5):452-460	non pertinent
4251	Kassubek et al.,	2014	BMC neurology. 2014; 14:42	non pertinent
4252	Karlson et al.,	2014	Cardiology. 2014; 127(2):73-82	non pertinent
4253	Kapusuz et al.,	2014	Biomed research international. 2014	non pertinent
4254	Kanungo et al.,	2014	PLoS One. 2014; 9(7):e99381	non pertinent
4255	Kane et al.,	2014	J of clinical psychiatry. 2014; 75(11):1254-1260	non pertinent
4256	Kamboj et al.,	2014	European j of pain (London, England). 2014; 18(10):1376-1384	non pertinent
4257	Kalita et al.,	2014	J of the neurological sciences. 2014; 342(1-2):127-132	non pertinent
4258	Kahn et al.,	2014	Lancet. 2014; 383(9920):880-888	non pertinent
4259	Jung et al.,	2014	Arthroscopy – j of arthroscopic and related surgery. 2014; 30(10):1261-1268	non pertinent
4260	Jprn, Umin – Tatsuhiro	2014	UMIN-CTR Clinical Trial. 2014	non pertinent
4261	Jprn, Umin – Meigen	2014	UMIN-CTR Clinical Trial. 2014	non pertinent

4262	Jprn, Umin – Shimada	2014	UMIN-CTR Clinical Trial. 2014	non pertinent
4263	Joo et al.,	2014	J Clin Anesth. 2014; 26(6):475-479	non pertinent
4264	Joly et al.,	2014	BJU international. 2014; 115(1):65-73	non pertinent
4265	Johansson et al.,	2014	Brain injury. 2014; 28(3):304-310	non pertinent
4266	Ji et al.,	2014	Clinical therapeutics. 2014; 36(1):84-100.e109	non pertinent
4267	Jensen et al.,	2014	J Pain. 2014; 15(12):1328-1337	non pertinent
4268	Jenkins et al.,	2014	J of bone and joint surgery. American volume. 2014; 96(10):851-858	non pertinent
4269	Jahangard et al.,	2014	European neuropsychopharmacology. 2014; 24(8):1210-1221	non pertinent
4270	Jagla et al.,	2014	Pain. 2014; 155(10):2056-2062	non pertinent
4271	Jafari et al.,	2014	Archives of iranian medicine. 2014; 17(7):466-470	non pertinent
4272	Iveson et al.,	2014	The lancet. Oncology. 2014; 15(9):1007-1018	non pertinent
4273	Ittichaikulthol et al.,	2014	Chotmaihet thangphaet (j of the Medical Association of Thailand). 2014; 97(7):705-709	non pertinent
4274	Isrctn – Schaefert	2014	ISRCTN Registry. 2014	non pertinent
4275	Isrctn – Alberts	2014	ISRCTN Registry. 2014	non pertinent
4276	Ishoy et al.,	2014	BMJ Open. 2014; 4(1)	non pertinent
4277	Irct2014031016919N - Tavakkolizade	2014	IRCT – Iranian Registry of Clinical Trials. 2014	non pertinent
4278	Irct201407228717N - Asgari	2014	IRCT – Iranian Registry of Clinical Trials. 2014	non pertinent
4279	Irct201407048872N - Saeedi	2014	IRCT – Iranian Registry of Clinical Trials. 2014	non pertinent
4280	Inagaki et al.,	2014	Psychosomatic medicine. 2014; 76(3):A-108	non pertinent

4281	Ilfeld et al.,	2014	Regional anesthesia and pain medicine. 2014; 39(2):89-96	non pertinent
4282	Ihle et al.,	2014	Pain. 2014; 155(10):2080-2087	non pertinent
4283	Hysek et al.,	2014	The international j of neuropsychopharmacology. 2014; 17(3):371-381	non pertinent
4284	Hye et al.,	2014	J of vascular surgery. 2014; 60(2):454-461.e451	non pertinent
4285	Hwang et al.,	2014	Int J Rehabil Res. 2014; 37(2):167-172	non pertinent
4286	Hussein et al.,	2014	Eur Spine J. 2014; 23(9):1992-1999	non pertinent
4287	Husebo et al.,	2014	European j of pain (London, England). 2014; 18(10):1419-1430	non pertinent
4288	Hughbanks-Wheaton et al.,	2014	Investigative ophthalmology & visual science. 2014; 55(8):4958-4966	non pertinent
4289	Huei Hern	2014	Irish j of medical science. 2014; 183(4 SUPPL 1):176-178	non pertinent
4290	Huang et al.,	2014	J of arthroplasty. 2014; 29(12):2342-2346	non pertinent
4291	Huang et al.,	2014	Human vaccines and immunotherapeutics. 2014; 10(1):2790-2795	non pertinent
4292	Hu et al.,	2014	Chinese medical j. 2014; 127(15):2721-2726	non pertinent
4293	Howard et al.,	2014	Cardiovascular diabetology. 2014; 13(1)	non pertinent
4294	Hoskin et al.,	2014	The lancet. Oncology. 2014; 15(12):1397-1406	non pertinent
4295	Hosbond et al.,	2014	Atherosclerosis. 2014; 236(2):230-236	non pertinent
4296	Horta de Marcedo et al.,	2014	Psychiatry research. 2014; 220(1-2):125-128	non pertinent
4297	Hopkins et al.,	2014	American j of respiratory and cricital care medicine. 2014; 189	non pertinent

4298	Homan et al.,	2014	Int J Neuropsychopharmacol. 2014; 17(9):1419-1428	non pertinent
4299	Hollander	2014	Neuropsychopharmacology. 2014; 39:63	non pertinent
4300	Hollanda et al.,	2014	Neurology international. 2014; 6(4):70-73	non pertinent
4301	Hoeger Bement et al.,	2014	J Clin Neurophysiol. 2014; 31(1):94-98	non pertinent
4302	Hobl et al.,	2014	J of the American College of Cardiology. 2014; 63(7):630-635	non pertinent
4303	Hirsh et al.,	2014	J of thoracic oncology. 2014; 9(1):83-90	non pertinent
4304	Hirsch et al.,	2014	Clinical J of Pain. 2014; 30(12):1023-1032	non pertinent
4305	Heyse et al.,	2014	Clinical orthopaedics and related research. 2014; 472(1):272-276	non pertinent
4306	Herbinger et al.,	2014	Human vaccines and immunotherapeutics. 2014; 10(1):2768-2775	non pertinent
4307	Heo et al.,	2014	Digestive and liver disease. 2014; 46(11):980-984	non pertinent
4308	Henry et al.,	2014	J of diabetes and its complications. 2014; 28(3):393-398	non pertinent
4309	Henley et al.,	2014	Current medical research and opinion. 2014; 30(10):2021-2032	non pertinent
4310	Heller et al.,	2014	J of ocular pharmacology and therapeutics. 2014; 30(10):815-822	non pertinent
4311	Heinemann et al.,	2014	The lancet. Oncology. 2014; 15(10):1065-1075	non pertinent
4312	Hedengran et al.,	2014	Lipids. 2014; 50(2):165-175	non pertinent
4313	Hayton et al.,	2014	PLoS One. 2014; 9(7)	non pertinent
4314	Hawkes et al.,	2014	American j of tropical medicine and hygiene. 2014; 91(5):452-	non pertinent
4315	Hawkes et al.,	2014	Contemporary clinical trials. 2014; 39(1):14-21	non pertinent

4316	Hatherill et al.,	2014	Vaccine. 2014; 32(31):3982-3988	non pertinent
4317	Haruna et al.,	2014	J of interferon & cytokine research. 2014; 34(2):111-116	non pertinent
4318	Harfterkamp et al.,	2014	J of child and adolescent psychopharmacology. 2014; 24(9):481-485	non pertinent
4319	Hansen	2014	Danish medical j. 2014; 61(2):B4801	non pertinent
4320	Han et al.,	2014	J korean geriatr soc. 2014; 18(3):153-161	non pertinent
4321	Hammitt et al.,	2014	PLoS One. 2014; 9(1)	non pertinent
4322	Hamada	2014	J of oral and maxillofacial surgery. 2014; 72(9):e91-e92	non pertinent
4323	Halliday et al.,	2014	PLoS medicine. 2014; 11(1)	non pertinent
4324	Hall et al.,	2014	European j of cancer (Oxford, England : 1990). 2014; 50(14):2375-2389	non pertinent
4325	Haffizulla et al.,	2014	The lancet. Infectious diseases. 2014; 14(7):609-618	non pertinent
4326	Hadji et al.,	2014	Breast cancer research and treatment. 2014; 144(2):343-351	non pertinent
4327	Hackett et al.,	2014	International j of clinical practice. 2014; 68(2):203-215	non pertinent
4328	Gupta et al.,	2014	Int J Pediatr Otorhinolaryngol. 2014; 78(10):1686-1691	non pertinent
4329	Guo et al.,	2014	Supportive care in cancer. 2014; 22(5):1223-1231	non pertinent
4330	Guo et al.,	2014	Chinese j of experimental traditional medical formulae (zhong guo shi yan fang ji xue za zhi). 2014; 20(15):195-199	non pertinent
4331	Guhn et al.,	2014	Frontiers in behavioral neuroscience. 2014; 8(FEB)	non pertinent
4332	Guerrini et al.,	2014	Epilepsia. 2014; 55(4):568-578	non pertinent

4333	Gubelin Harcha et al.,	2014	J of the American Academy of Dermatology. 2014; 70(3):489-498-498	non pertinent
4334	Guan et al.,	2014	Zhongguo gu shang (China j of orthopaedics and traumatology). 2014; 27(1):13-16	non pertinent
4335	Grillon et al.,	2014	Neuropsychopharmacology. 2014; 40(5):1064-1071	non pertinent
4336	Gracies et al.,	2014	Archives of physical medicine and rehabilitation. 2014; 95(7):1303-1311	non pertinent
4337	Gowin et al.,	2014	Neuropsychopharmacology. 2014; 39:274	non pertinent
4338	Gorka et al.,	2014	Int J Neuropsychopharmacology. 2014; 18(3)	non pertinent
4339	Gorka et al.,	2014	J Psychopharmacol. 2014; 28(12):1115-1124	non pertinent
4340	Gordon et al.,	2014	British j of dermatology. 2014; 170(3):705-715	non pertinent
4341	Gonzales et al.,	2014	Clinical pharmacology and therapeutics. 2014; 96(3):390-396	non pertinent
4342	Goldstein-Piekarski et al.,	2014	Neuropsychopharmacology. 2014; 39:249	non pertinent
4343	Goldberg et al.,	2014	British j of ophthalmology. 2014; 98(7):926-931	non pertinent
4344	Gold et al.,	2014	J of drugs in dermatology. 2014; 13(11):1380-1386	non pertinent
4345	Gokhale et al.,	2014	Experimental hematology and oncology. 2014; 3(1)	non pertinent
4346	Gohil et al.,	2014	Knee Surg Sports Traumatol Arthrosc. 2014; 22(5):1090-1096	non pertinent
4347	Goforth et al.,	2014	Sleep. 2014; 37(6):1053-1060	non pertinent
4348	Goebel et al.,	2014	Trials. 2014; 15:404	non pertinent
4349	Gligorov et al.,	2014	The lancet. Oncology. 2014; 15(12):1351-1360	non pertinent

4350	Giovannoni et al.,	2014	The lancet. Neurology. 2014; 13(5):472-481	non pertinent
4351	Gimenez et al.,	2014	J Rheumatol. 2014; 41(11):2240-2248	non pertinent
4352	Gimenez et al.,	2014	Eur Neuropsychophar macol. 2014; 24(1):105-116	non pertinent
4353	Gibson et al.,	2014	Schizophrenia research. 2014; 156(2-3):261-265	non pertinent
4354	Giannantoni et al.,	2014	Urology. 2014; 83(2):400-405	non pertinent
4355	Gewandter et al.,	2014	Supportive care in cancer. 2014; 22(7):1807-1814	non pertinent
4356	Germain et al.,	2014	Sleep. 2014; 37:A276-	non pertinent
4357	Geraghty et al.,	2014	BMJ Open. 2014; 4(7):e005871	non pertinent
4358	George et al.,	2014	Brain stimulation. 2014; 7(3):421-431	non pertinent
4359	Genovese et al.,	2014	J of rheumatology. 2014; 41(4):629-639	non pertinent
4360	Geisthoff et al.,	2014	Thrombosis research. 2014; 134(3):565-571	non pertinent
4361	Gay et al.,	2014	J Manipulative Physiol Ther. 2014; 37(9):614-627	non pertinent
4362	Gasser et al.,	2014	J of nervous and mental disease. 2014; 202(7):513-520	non pertinent
4363	Garvey et al.,	2014	Diabetes care. 2014; 37(12):3309-3316	non pertinent
4364	Garcia-Manero et al.,	2014	American j of hematology. 2014; 89(9):E156-E162	non pertinent
4365	Garbelotti et al.,	2014	Gait Posture. 2014; 40(1):150-153	non pertinent
4366	Gao et al.,	2014	J of cranio-maxillo-facial surgery. 2014; 42(8):1577-1582	non pertinent
4367	Gadelha et al.,	2014	The lancet. Diabetes & endocrinology. 2014; 2(11):875-884	non pertinent
4368	Fu et al.,	2014	British j of anaesthesia. 2014; 112(3):506-513	non pertinent
4369	Fu et al.,	2014	Clinical schizophrenia & related psychoses. 2014; 8(2):101-109A	non pertinent

4370	Frijling et al.,	2014	European neuropsychophar macology. 2014; 24:602	non pertinent
4371	French et al.,	2014	Acta neurologica scandinavica. 2014; 129(3):143-153	non pertinent
4372	Frederiksen et al.,	2014	European neuropsychophar macology. 2014; 24(12):1873-1884	non pertinent
4373	Franconi et al.,	2014	Neurological sciences. 2014; 35(SUPPL 1):99-105	non pertinent
4374	Francavilla et al.,	2014	J of clinical gastroenterology. 2014; 48(5):407-413	non pertinent
4375	Fracalanza et al.,	2014	J Anxiety Disord. 2014; 28(6):559-569	non pertinent
4376	Ford et al.,	2014	British j of clinical pharmacology. 2014; 78(3):498-508	non pertinent
4377	Ford et al.,	2014	The lancet. Oncology. 2014; 15(1):78-86	non pertinent
4378	Forcelini et al.,	2014	Neurogastroenter ol Motil. 2014; 26(2):187-195	non pertinent
4379	Fooladi et al.,	2014	J of sexual medicine. 2014; 11(3):831-839	non pertinent
4380	Flodin et al.,	2014	Brain connectivity. 2014; 4(8):587-594	non pertinent
4381	Flaim et al.,	2014	J of the american heart association. 2014; 3(2)	non pertinent
4382	Fizazi et al.,	2014	The lancet. Oncology. 2014; 15(10):1147-1156	non pertinent
4383	Fizazi et al.,	2014	The lancet. Oncology. 2014; 15(9):975-985	non pertinent
4384	Finckh et al.,	2014	Arthritis research & therapy. 2014; 16(5):458	non pertinent
4385	Figus et al.,	2014	Eye (london, england). 2014; 28(4):422-429	non pertinent
4386	Ferrucci et al.,	2014	Melanoma research. 2014; 25(3):239-245	non pertinent
4387	Fernandes et al.,	2014	J of sexual medicine. 2014; 11(5):1262-1270	non pertinent
4388	Fayed et al.,	2014	CNS neuroscience & therapeutics. 2014; 20(11):999-1007	non pertinent

4389	Favrat et al.,	2014	PLoS One. 2014; 9(4):e94217	non pertinent
4390	Fanelli et al.,	2014	Minerva Anesthesiologica. 2014; 80(1):66-75	non pertinent
4391	Fan et al.,	2014	Annals of surgical oncology. 2014; 21(12):3876-3881	non pertinent
4392	Fallah et al.,	2014	Arch Iran Med. 2014; 17(11):741-745	non pertinent
4393	Eudract_number - ?	2014	?	non pertinent
4394	Euctr N.L.	2014	EU Clinical Trial Register. 2014	non pertinent
4395	Euctr H.R.	2014	EU Clinical Trial Register. 2014	non pertinent
4396	Euctr E.S.	2014	EU Clinical Trial Register. 2014	non pertinent
4397	Euctr D.K.	2014	EU Clinical Trial Register. 2014	non pertinent
4398	Euctr D.E.	2014	EU Clinical Trial Register. 2014	non pertinent
4399	Euctr C.Z.	2014	EU Clinical Trial Register. 2014	non pertinent
4400	Esposito et al.,	2014	Eur J Oral Implantol. 2014; 7(2):129-137	non pertinent
4401	Espey et al.,	2014	American j of obstetrics and gynecology. 2014; 210(3):208.e201-205	non pertinent
4402	Eslamparast et al.,	2014	American j of clinical nutrition. 2014; 99(3):535-542	non pertinent
4403	Eskin et al.,	2014	J of emergency medicine. 2014; 47(1):65-70	non pertinent
4404	Escalante et al.,	2014	Cancer j (sudbury, mass.). 2014; 20(1):8-14	non pertinent
4405	Emslie et al.,	2014	J of child and adolescent psychopharmacology. 2014; 24(4):170-179	non pertinent
4406	Emmert et al.,	2014	Frontiers in behavioral neuroscience. 2014; 8(OCT)	non pertinent
4407	Ellis et al.,	2014	The lancet. Oncology. 2014; 15(12):1379-1388	non pertinent
4408	El-Kheir et al.,	2014	Cell transplantation. 2014; 23(6):729-745	non pertinent
4409	Eckstein et al.,	2014	Neuropsychopharmacology. 2014; 39:192-193	non pertinent
4410	Eberl et al.,	2014	Techniques in coloproctology. 2014; 18(8):745-752	non pertinent

4411	Eathakkattu et al.,	2014	Internal medicine j. 2014; 44:5-	non pertinent
4412	Dutton et al.,	2014	Lancet Oncol. 2014; 15(8):894-904	non pertinent
4413	Dunlop et al.,	2014	Trials. 2014; 15:240	non pertinent
4414	Ducreux et al.,	2014	European j of cancer. 2014; 50(18):3098-3106	non pertinent
4415	Dubeau et al.,	2014	J of urology. 2014; 191(2):395-404	non pertinent
4416	Du et al.,	2014	J of clinical gastroenterology. 2014; 48(4):328-335	non pertinent
4417	Du Bois et al.,	2014	J of clinical oncology. 2014; 32(30):3374-3381	non pertinent
4418	Droitcourt et al.,	2014	Chest. 2014; 146(4):1046-1054	non pertinent
4419	Dreicer et al.,	2014	Clinical cancer research. 2014; 20(5):1335-1344	non pertinent
4420	Douillard et al.,	2014	Clinical colorectal cancer. 2014; 13(1):14-26.e11	non pertinent
4421	Doslikova et al.,	2014	Osteoarthritis and cartilage. 2014; 22:89	non pertinent
4422	Dong et al.,	2014	J of neurosurgery: spine. 2014; 20(1):53-59	non pertinent
4423	Doedee et al.,	2014	PLoS One. 2014; 9(6)	non pertinent
4424	Dodick et al.,	2014	The lancet. Neurology. 2014; 13(9):885-892	non pertinent
4425	Dodick et al.,	2014	The lancet neurology. 2014; 13(11):1100-1107	non pertinent
4426	Dilger et al.,	2014	J of Crohn's & colitis. 2014; 8(9):970-980	non pertinent
4427	Difede et al.,	2014	Neuropsychophar macology. 2014; 39(5):1052-1058	non pertinent
4428	Diamond et al.,	2014	Reproductive sciences (thousands oaks, calif.). 2014; 21(3):363-371	non pertinent
4429	Diamant et al.,	2014	The lancet. Diabetes & endocrinology. 2014; 2(6):464-473	non pertinent
4430	Di Simplicio et al.,	2014	Psychol Med. 2014; 44(2):241-252	non pertinent
4431	Di Cicco et al.,	2014	J of cystic fibrosis. 2014; 13(4):455-460	non pertinent

4432	Dewhirst et al.,	2014	International j of pediatric otorhinolaryngology. 2014; 78(7):1090-1094	non pertinent
4433	Devlin et al.,	2014	Chest. 2014; 145(6):1204-1212	non pertinent
4434	Devane et al.,	2014	Current medical research and opinion. 2014; 30(6):1179-1187	non pertinent
4435	Detke et al.,	2014	J of clinical psychopharmacology. 2014; 34(4):426-434	non pertinent
4436	Detert et al.,	2014	Trials. 2014; 15(1)	non pertinent
4437	Denison et al.,	2014	Diabetes, obesity & metabolism. 2014; 16(4):334-343	non pertinent
4438	Demant et al.,	2014	Pain. 2014; 155(11):2263-2273	non pertinent
4439	Dehghani et al.,	2014	J of isfahan medical school. 2014; 31(267):2155-2161	non pertinent
4440	Deharo et al.,	2014	Annales de cardiologie ET d'angeiologie. 2014; 63(4):222-227	non pertinent
4441	Deeks	2014	Biodrugs. 2014; 28(4):403-410	non pertinent
4442	De Wit et al.,	2014	Diabetologia. 2014; 57(9):1812-1819	non pertinent
4443	De Waele et al.,	2014	Surgical infections. 2014; 15(5):567-575	non pertinent
4444	De Kam et al.,	2014	Platelets. 2014; 25(7):480-487	non pertinent
4445	De Francesco et al.,	2014	J of medical microbiology. 2014; 63(Pt 5):748-752	non pertinent
4446	Davis et al.,	2014	Cancer j (united states). 2014; 20(5):330-344	non pertinent
4447	Dang et al.,	2014	Drug design, development and therapy. 2014; 8:1441-1449	non pertinent
4448	Dancey et al.,	2014	Experimental brain research. 2014; 232(9):2879-2889	non pertinent
4449	Dall'Agnol et al.,	2014	J of Pain. 2014; 15(8):845-855	non pertinent
4450	Daggett et al.,	2014	Brain injury. 2014; 28(5-6):554-555	non pertinent

4451	Cuzick et al.,	2014	Lancet (london, england). 2014; 383(9922):1041-1048	non pertinent
4452	Cuttler et al.,	2014	J Subst Abuse Treat. 2014; 47(4):299-305	non pertinent
4453	Cutler et al.,	2014	J of the American Academy of Child and Adolescent Psychiatry. 2014; 53(10):1092-1101	non pertinent
4454	Cummings et al.,	2014	Vaccine. 2014; 32(19):2251-2259	non pertinent
4455	Crockett et al.,	2014	Proc Natl Acad Sci U S A. 2014; 111(48):17320-17325	non pertinent
4456	Cravioto et al.,	2014	Contraception. 2014; 90(2):147-153	non pertinent
4457	Cranston et al.,	2014	AIDS research and human retroviruses. 2014; 30(2):184-189	non pertinent
4458	Craggs et al.,	2014	J Pain. 2014; 15(4):435-446	non pertinent
4459	Courcoulas et al.,	2014	JAMA surgery. 2014; 149(7):707-715	non pertinent
4460	Costantini et al.,	2014	International urogynecology j. 2014; 25(9):1173-1178	non pertinent
4461	Corsini-Munt et al.,	2014	Trials. 2014; 15(1)	non pertinent
4462	Corrie et al.,	2014	The lancet. Oncology. 2014; 15(6):620-630	non pertinent
4463	Cornwell et al.,	2014	Behav Brain Res. 2014; 261:258-264	non pertinent
4464	Corey et al.,	2014	Antimicrobial agents and chemotherapy. 2014; 58(11):6518-6527	non pertinent
4465	Cooper et al.,	2014	Health technology assessment (Winchester, England). 2014; 18(54):1-128	non pertinent
4466	Cook et al.,	2014	The lancet. Oncology. 2014; 15(8):874-885	non pertinent
4467	Constantine et al.,	2014	Pediatric emergency care. 2014; 30(7):474-478	non pertinent
4468	Conroy et al.,	2014	The lancet. Oncology. 2014; 15(3):305-314	non pertinent
4469	Coll et al.,	2014	Cytotherapy. 2014; 16:97-98	non pertinent

4470	Cohen et al.,	2014	Anesthesiology. 2014; 121(5):1045-1055	non pertinent
4471	Cohen et al.,	2014	Pain medicine (Malden, Mass.). 2014; 15(4):588-602	non pertinent
4472	Coffey et al.,	2014	Emergency medicine j. 2014; 31(8):613-618	non pertinent
4473	Clausen et al.,	2014	Trials. 2014; 15(1)	non pertinent
4474	Choy et al.,	2014	BMC Cancer. 2014; 14(1)	non pertinent
4475	Chopra et al.,	2014	Arthritis & rheumatology (hoboken, N.J.). 2014; 66(2):319-326	non pertinent
4476	Chong et al.,	2014	Phytotherapy research: PTR. 2014; 28(10):1520-1526	non pertinent
4477	Choi et al.,	2014	International j of clinical pharmacology and therapeutics. 2014; 52(1):55-63	non pertinent
4478	Cho et al.,	2014	Europace. 2014; 16(7):1000-1006	non pertinent
4479	Chi – Peking University School & Hospital of Stomatology	2014	Chinese Clinical Trial Registry. 2014	non pertinent
4480	Chi – Guangdong 999 Brain Hospital	2014	Chinese Clinical Trial Registry. 2014	non pertinent
4481	Chey et al.,	2014	New England j of medicine. 2014; 370(25):2387-2396	non pertinent
4482	Chen et al.,	2014	Sci Rep. 2014; 4:6482	non pertinent
4483	Chen et al.,	2014	Clinical pharmacokinetics. 2014; 53(11):1033-1044	non pertinent
4484	Charles et al.,	2014	Parkinsonism & related disorders. 2014; 20(7):731-737	non pertinent
4485	Chapman et al.,	2014	J Psychopharmacol. 2014; 28(12):1135-1142	non pertinent
4486	Chang et al.,	2014	Academic emergency medicine. 2014; 21(3):228-235	non pertinent
4487	Chandon et al.,	2014	PLoS One. 2014; 9(8)	non pertinent

4488	Chan et al.,	2014	J of the american medical directors association. 2014; 15(8):607.e605-607.e612	non pertinent
4489	Chan et al.,	2014	International j of radiation oncology, biology, physics. 2014; 90(4):756-764	non pertinent
4490	Cecchetti et al.,	2014	Trials. 2014; 15(1)	non pertinent
4491	Catley et al.,	2014	Manual therapy. 2014; 19(5):461-466	non pertinent
4492	Carrieri et al.,	2014	PLoS One. 2014; 9(11)	non pertinent
4493	Cannata et al.,	2014	Minerva Anestesiologica. 2014; 80(1):58-65	non pertinent
4494	Camuri et al.,	2014	European neuropsychopharmacology. 2014; 24:61-62	non pertinent
4495	Campisi et al.,	2014	Osteoarthritis and cartilage. 2014; 22:475-476	non pertinent
4496	Campbell et al.,	2014	Neurobiology of aging. 2014; 35(10):2246-2248	non pertinent
4497	Cady et al.,	2014	Headache. 2014; 54(1):80-93	non pertinent
4498	Cabido et al.,	2014	J Strength Cond Res. 2014; 28(4):1050-1057	non pertinent
4499	Butts et al.,	2014	The lancet. Oncology. 2014; 15(1):59-68	non pertinent
4500	Burke et al.,	2014	American j of hematology. 2014; 89(9):889-895	non pertinent
4501	Bureau et al.,	2014	AJNR Am J Neuroradiol. 2014; 35(8):1467-1474	non pertinent
4502	Buijze et al.,	2014	J Hand Surg Am. 2014; 39(4):621-627	non pertinent
4503	Bugarski-Kirola et al.,	2014	European neuropsychopharmacology. 2014; 24(7):1024-1036	non pertinent
4504	Bucci et al.,	2014	Techniques in coloproctology. 2014; 18(4):345-353	non pertinent
4505	Bruinsma et al.,	2014	BMC musculoskeletal disorders. 2014; 15(1)	non pertinent
4506	Breitschaft et al.,	2014	Diabetes research and clinical practice. 2014; 103(3):458-465	non pertinent

4507	Boyle et al.,	2014	The lancet. Respiratory medicine. 2014; 2(7):527-538	non pertinent
4508	Boyer et al.,	2014	Neurology. 2014; 82(14):1231-1238	non pertinent
4509	Bowen et al.,	2014	Lancet (london, england). 2014; 384(9960):2132-2140	non pertinent
4510	Bot et al.,	2014	Clinical orthopaedics and related research. 2014; 472(8):2542-2549	non pertinent
4511	Bosma et al.,	2014	Magn Reson Imaging. 2014; 32(5):473-481	non pertinent
4512	Bortone et al.,	2014	Paediatric anaesthesia. 2014; 24(6):614-619	non pertinent
4513	Böning et al.,	2014	Hormone research in paediatrics. 2014; 82:395-	non pertinent
4514	Bonham	2014	Obstetrical & gynecological survey. 2014; 70(4):274-283	non pertinent
4515	Bolinder et al.,	2014	Diabetes, obesity & metabolism. 2014; 16(2):159-169	non pertinent
4516	Boccard-Binet et al.,	2014	Stereotactic and functional neurosurgery. 2014; 92:38	non pertinent
4517	Blom et al.,	2014	New England j of medicine. 2014; 370(19):1809-1819	non pertinent
4518	Blackhall et al.,	2014	J of thoracic oncology. 2014; 9(11):1625-1633	non pertinent
4519	Biswal et al.,	2014	International j of clinical pharmacology and therapeutics. 2014; 52(11):996-1004	non pertinent
4520	Billy et al.,	2014	Pm r. 2014; 6(9):790-795	non pertinent
4521	Bieber et al.,	2014	J of drugs in dermatology. 2014; 13(7):791-798	non pertinent
4522	Bible et al.,	2014	J of clinical endocrinology and metabolism. 2014; 99(5):1687-1693	non pertinent
4523	Bharti et al.,	2014	Paediatric anaesthesia. 2014; 24(11):1158-1163	non pertinent

4524	Berry et al.,	2014	Global advances in health and medicine. 2014; 3(2):28-33	non pertinent
4525	Bernasconi et al.,	2014	Cerebral cortex (new york, N.Y. : 1991). 2014; 24(12):3221-3231	non pertinent
4526	Berlin et al.,	2014	BMJ (online). 2014; 348	non pertinent
4527	Berende et al.,	2014	BMC Infect Dis. 2014; 14:543	non pertinent
4528	Benson et al.,	2014	Brain, behavior and immunity. 2014; 40:e49	non pertinent
4529	Belfer et al.,	2014	Pain Med. 2014; 15(9):1590-1602	non pertinent
4530	Beken et al.,	2014	J Child Neurol. 2014; 29(5):617-622	non pertinent
4531	Beck et al.,	2014	International j of colorectal disease. 2014; 29(12):1527-1534	non pertinent
4532	Baumunk et al.,	2014	Urologia internationalis. 2014; 93(2):193-201	non pertinent
4533	Batki et al.,	2014	Alcoholism, clinical and experimental research. 2014; 38(8):2169-2177	non pertinent
4534	Bath-Hextall et al.,	2014	The lancet. Oncology. 2014; 15(1):96-105	non pertinent
4535	Basiri-Moghadam et al.,	2014	JPMA. The j of the pakistan medical association. 2014; 64(8):874-878	non pertinent
4536	Bartolo et al.,	2014	J of the neurological sciences. 2014; 349(1-2):117-123	non pertinent
4537	Bart et al.,	2014	Science translational medicine. 2014; 6(234):234ra255	non pertinent
4538	Barrett-Lee et al.,	2014	The lancet. Oncology. 2014; 15(1):114-122	non pertinent
4539	Barkan et al.,	2014	Emergency medicine j. 2014; 31(8):649-653	non pertinent
4540	Barchuk et al.,	2014	Pulmonary pharmacology & therapeutics. 2014; 29(1):15-23	non pertinent
4541	Bang et al.,	2014	Die pharmazie. 2014; 70(4):269-273	non pertinent
4542	Ballinger et al.,	2014	J Autism Dev Disord. 2014; 44(10):2536-2546	non pertinent

4543	Ball et al.,	2014	J of affective disorders. 2014; 167:215-223	non pertinent
4544	Balanescu et al.,	2014	Annals of the rheumatic diseases. 2014; 73(9):1665-1672	non pertinent
4545	Bakish et al.,	2014	J of psychiatry & neuroscience. 2014; 39(1):40-49	non pertinent
4546	Baas et al.,	2014	Frontiers in behavioral neuroscience. 2014; 8(SEP)	non pertinent
4547	Aysenne et al.,	2014	Neurocritical care. 2014; 21(1 SUPPL 1):61	non pertinent
4548	Aurora et al.,	2014	Acta neurologica scandinavica. 2014; 129(1):61-70	non pertinent
4549	Auliac et al.,	2014	Lung cancer (Amsterdam, Netherlands). 2014; 85(3):415-419	non pertinent
4550	Auffret et al.,	2014	American j of emergency medicine. 2014; 32(6):655-659	non pertinent
4551	Atukunda et al.,	2014	PLoS medicine. 2014; 11(11):e1001752	non pertinent
4552	Attie et al.,	2014	American j of hematology. 2014; 89(7):766-770	non pertinent
4553	Attarian et al.,	2014	Orphanet j of rare diseases. 2014; 9(1)	non pertinent
4554	Attari et al.,	2014	J of research in medical sciences. 2014; 19(7):592-598	non pertinent
4555	Atsmon et al.,	2014	Vaccine. 2014; 32(44):5816-5823	non pertinent
4556	Asrani et al.,	2014	American j of transplantation. 2014; 14(2):356-366	non pertinent
4557	Aslaksen et al.,	2014	Exp Brain Res. 2014; 232(6):1865-1873	non pertinent
4558	Ashrafi et al.,	2014	Pediatric neurology. 2014; 51(4):503-508	non pertinent
4559	Asadollahi et al.,	2014	Headache. 2014; 54(1):94-108	non pertinent
4560	Aronson et al.,	2014	Diabetes care. 2014; 37(10):2746-2754	non pertinent
4561	Arnold et al.,	2014	Current medical research and opinion. 2014; 30(10):2069-2083	non pertinent

4562	Areces et al.,	2014	Amino acids. 2014; 46(5):1169-1176	non pertinent
4563	Arden et al.,	2014	Current medical research and opinion. 2014; 30(2):279-286	non pertinent
4564	Araujo et al.,	2014	Regional anesthesia and pain medicine. 2014; 39(5 SUPPL 1):e321	non pertinent
4565	Anseline et al.,	2014	Photodiagnosis and photodynamic therapy. 2014; 11(4):467-471	non pertinent
4566	André et al.,	2014	The lancet. Oncology. 2014; 15(6):580-591	non pertinent
4567	Andersson et al.,	2014	American j of cardiovascular drugs. 2014; 14(3):217-227	non pertinent
4568	Amr et al.,	2014	J of pain and symptom management. 2014; 48(5):944-956.e942	non pertinent
4569	Amorim et al.,	2014	Acta anaesthesiologica Scnadinavica. 2014; 58(9):1101-1110	non pertinent
4570	Amin et al.,	2014	Lancet. 2014; 383(9927):1474-1482	non pertinent
4571	Amin et al.,	2014	Brain. 2014; 137(Pt 3):779-794	non pertinent
4572	Amemiya et al.,	2014	J of atherosclerosis and thrombosis. 2014; 21(12):1298-1307	non pertinent
4573	Alsallom et al.,	2014	Neurosciences (Riyadh). 2014; 19(3):218-223	non pertinent
4574	Alberer et al.,	2014	Travel medicine and infectious disease. 2014; 12(5):485-493	non pertinent
4575	Alavi et al.,	2014	Asian cardiovascular & thoracic annals. 2014; 22(6):694-699	non pertinent
4576	Ahrén et al.,	2014	Diabetologia. 2014; 57(7):1304-1307	non pertinent
4577	Ahmed et al.,	2014	European neuropsychopharmacology. 2014; 24(9):1475-1482	non pertinent

4578	Aggrawal et al.,	2014	J of ayuverda and integrative medicine. 2014; 5(2):117-124	non pertinent
4579	Adelman et al.,	2014	J of General Internal Medicine. 2014; 29:371-	non pertinent
4580	Actrn – Rodriguez-Blanco	2014	Australian New Zealand Clinical Trials Registry. 2014	non pertinent
4581	Actrn – Brosseau	2014	Australian New Zealand Clinical Trials Registry. 2014	non pertinent
4582	Actrn - Alvarez	2014	Australian New Zealand Clinical Trials Registry. 2014	non pertinent
4583	Abuelkheir et al.,	2014	J of international medical research. 2014; 42(2):329-336	non pertinent
4584	Abdulkadyrov et al.,	2014	British j of haematology. 2014; 165(6):814-823	non pertinent
4585	Abdallah et al.,	2014	Anesthesiology. 2014; 121(6):1302-1310	non pertinent
4586	Heinemann et al.,	2014	Lancet Oncology. 2014; 15(10):1065-1075	non pertinent
4587	Moon et al.,	2014	J of neurosurgery. 2014; 121(5):1085-1092	non pertinent
4588	Aronne et al.,	2014	Postgraduate medicine. 2014; 126(6):7-18	non pertinent
4589	Tung et al.,	2014	Annals of clinical and translational neurology. 2014; 1(9):633-638	non pertinent
4590	EACTA Abstracts	2014	Applied cardiopulmonary pathophysiology. 2014; 17(2):233-234	non pertinent
4591	Zhang et al.,	2013	J of alternative and complementary medicine. 2013; 19(7):A31	non pertinent
4592	Zhang et al.,	2013	Prog Neuropsychopharmacol Biol Psychiatry. 2013; 40:364-373	non pertinent
4593	Zhang et al.,	2013	Cardiology in the young. 2013; 23(1):54-60	non pertinent

4594	Yuan et al.,	2013	Experimental brain research. 2013; 226(1):15-24	non pertinent
4595	Yim et al.,	2013	Am J Sports Med. 2013; 41(7):1565-1570	non pertinent
4596	Yang et al.,	2013	Vaccine. 2013; 31(40):4389-4397	non pertinent
4597	Yang et al.,	2013	Turkish j of gastroenterology. 2013; 24(3):197-203	non pertinent
4598	Yan et al.,	2013	Asian pacific j of tropical medicine. 2013; 6(11):912-915	non pertinent
4599	Wright et al.,	2013	British j of clinical pharmacology. 2013; 76(6):917-931	non pertinent
4600	Wollmer et al.,	2013	Toxicon. 2013; 68:72	non pertinent
4601	Williams et al.,	2013	Contraception. 2013; 88(3):401-407	non pertinent
4602	Williams et al.,	2013	Neuropsychopharmacology. 2013; 38:217-218	non pertinent
4603	William Hanke et al.,	2013	Dermatologic surgery. 2013; 39(6):891-899	non pertinent
4604	Wijburg et al.,	2013	Molecular genetics and metabolism. 2013; 108(2):99	non pertinent
4605	Wietecha et al.,	2013	J of child and adolescent psychopharmacology. 2013; 23(9):605-613	non pertinent
4606	Wickström et al.,	2013	Acta obstetrica ET gynecologica scandinavica. 2013; 92(12):1375-1382	non pertinent
4607	Whiteley	2013	J of science and medicine in sport. 2013; 16:e54-	non pertinent
4608	Werner et al.,	2013	J of bone and joint surgery. American volume. 2013; 95(7):577-584	non pertinent
4609	Weng et al.,	2013	Psychol Sci. 2013; 24(7):1171-1180	non pertinent
4610	Weiland et al.,	2013	Molecular therapy. 2013; 21(9):1796-1805	non pertinent
4611	Weigand et al.,	2013	Brain Stimul. 2013; 6(6):905-912	non pertinent
4612	Wegner et al.,	2013	Brain, behavior, and immunity. 2013; 29:19	non pertinent

4613	Wang-Gillam et al.,	2013	Investigational new drugs. 2013; 31(3):707-713	non pertinent
4614	Waller et al.,	2013	BMC Musculoskelet Disord. 2013; 14:82	non pertinent
4615	Waibel et al.,	2013	J Drugs Dermatol. 2013; 12(11):1237-1242	non pertinent
4616	Wadden et al.,	2013	International j of obesity (2015). 2013; 37(11):1443-1451	non pertinent
4617	Vourvahis et al.,	2013	Antiviral therapy. 2013; 18(6):745-754	non pertinent
4618	Vorsanger et al.,	2013	J of opioid management. 2013; 9(4):281-290	non pertinent
4619	Volz et al.,	2013	PLoS One. 2013; 8(1):e52968	non pertinent
4620	Villa et al.,	2013	Leukemia & lymphoma. 2013; 54(7):1387-1395	non pertinent
4621	Vik-Mo et al.,	2013	Cancer immunology, immunotherapy. 2013; 62(9):1499-1509	non pertinent
4622	Verstovsek et al.,	2013	Haematologica. 2013; 98(12):1865-1871	non pertinent
4623	Vera-Llonch et al.,	2013	Alimentary pharmacology & therapeutics. 2013; 38(2):124-133	non pertinent
4624	Vassal et al.,	2013	Clin Neurophysiol. 2013; 124(9):1861-1867	non pertinent
4625	Vanuytsel et al.,	2013	Neurogastroenterology and motility. 2013; 25(5):e353-361	non pertinent
4626	Vanuytsel et al.,	2013	Clinical gastroenterology and hepatology. 2013; 11(9):1115-1121	non pertinent
4627	Van Zundert	2013	Regional anesthesia and pain medicine. 2013; 38(5):E5-E8	non pertinent
4628	Van Tiel et al.,	2013	PLoS One. 2013; 8(11)	non pertinent
4629	Van Ast et al.,	2013	Biological psychiatry. 2013; 74(11):809-816	non pertinent
4630	Tzabazis et al.,	2013	Mol Pain. 2013; 9:33	non pertinent

4631	Turkoz et al.,	2013	Paediatr Anaesth. 2013; 23(11):1078-1083	non pertinent
4632	Tsujino et al.,	2013	J of gastroenterology. 2013; 48(8):982-988	non pertinent
4633	Tse et al.,	2013	J of clinical nursing. 2013; 22(13-14):1843-1856	non pertinent
4634	Tsai et al.,	2013	Childs Nerv Syst. 2013; 29(7):1123-1129	non pertinent
4635	Truong et al.,	2013	Circulation. 2013; 127(25):2494-2502	non pertinent
4636	Treister et al.,	2013	Neuroscience letters. 2013; 548:115-119	non pertinent
4637	Töx et al.,	2013	Endoscopy. 2013; 45(6):439-444	non pertinent
4638	Toro et al.,	2013	Supportive care in cancer. 2013; 21:139	non pertinent
4639	Thomaes et al.,	2013	Soc Cogn Affect Neurosci. 2013; 8(2):190-200	non pertinent
4640	Terry et al.,	2013	Pain. 2013; 154(12):2759-2768	non pertinent
4641	Terburg et al.,	2013	Neuropsychophar macology. 2013; 38(13):2708-2716	non pertinent
4642	Taylor et al.,	2013	Archives of physical medicine and rehabilitation. 2013; 94(10):e29-	non pertinent
4643	Taylor et al.,	2013	Neuropsychophar macology. 2013; 38:149-150	non pertinent
4644	Tanaka et al.,	2013	Explore (new york, N.Y.). 2013; 9(2):82-86	non pertinent
4645	Tanaka et al.,	2013	PLoS One. 2013; 8(11):e80731	non pertinent
4646	Tabor et al.,	2013	Pain. 2013; 154(10):1961-1965	non pertinent
4647	Suzuki et al.,	2013	Neuropsychiatric disease and treatment. 2013; 9:1281-1287	non pertinent
4648	Suris et al.,	2013	Annals of clinical psychiatry. 2013; 25(1):33-40	non pertinent
4649	Sulkowski et al.,	2013	Annals of internal medicine. 2013; 159(2):86-96	non pertinent
4650	Sugimoto et al.,	2013	World j of surgery. 2013; 37(10):2454-2457	non pertinent
4651	Stumpf et al.,	2013	PLoS One. 2013; 8(11):e79123	non pertinent
4652	Stefani et al.,	2013	PLoS One. 2013; 8(10):e74107	non pertinent

4653	Spaeth et al.,	2013	Arthritis research & therapy. 2013; 15(6)	non pertinent
4654	Soliman et al.,	2013	Experimental and clinical transplantation. 2013; 11(6):494-498	non pertinent
4655	Smits et al.,	2013	J of psychiatric research. 2013; 47(10):1455-1461	non pertinent
4656	Smith et al.,	2013	Breast cancer research and treatment. 2013; 142(2):381-388	non pertinent
4657	Smaill et al.,	2013	Science translational medicine. 2013; 5(205)	non pertinent
4658	Sloan-Lancaster et al.,	2013	Diabetes care. 2013; 36(8):2239-2246	non pertinent
4659	Slingerland et al.,	2013	Clinical therapeutics. 2013; 35(12):1946-1954	non pertinent
4660	Sieper et al.,	2013	Annals of the rheumatic disease. 2013; 71	non pertinent
4661	Sidharta et al.,	2013	J of clinical pharmacology. 2013; 53(11):1131-1138	non pertinent
4662	Shepherd	2013	Phlebolymphology (pp. 1-65). 2013; 20(1):27-29	non pertinent
4663	Sheng et al.,	2013	Biol Psychol. 2013; 92(2):380-386	non pertinent
4664	Shamay-Tsoory et al.,	2013	Psychoneuroendocrinology. 2013; 38(12):3139-3144	non pertinent
4665	Sewell	2013	Biological psychiatry. 2013; 73(9 SUPPL 1):46	non pertinent
4666	Sethi et al.,	2013	Paediatric anaesthesia. 2013; 23(12):1131-1137	non pertinent
4667	Seminowicz et al.,	2013	J of Pain. 2013; 14(12 SUPPL):1573-1584	non pertinent
4668	Seidman et al.,	2013	Clinical breast cancer. 2013; 13(4):239-246.e231	non pertinent
4669	Seddik et al.,	2013	European j of clinical pharmacology. 2013; 69(9):1709-1715	non pertinent
4670	Schneider et al.,	2013	Emotion (Washington, D.C.). 2013; 13(5):852-866	non pertinent

4671	Schmid et al.,	2013	Cardiovasc Ther. 2013; 31(3):e1-6	non pertinent
4672	Schmid et al.,	2013	Pain. 2013; 154(11):2372-2380	non pertinent
4673	Schmid et al.,	2013	Gastroenterology. 2013; 144(5 SUPPL 1):555	non pertinent
4674	Scheifele et al.,	2013	Human vaccines & immunotherapeutics. 2013; 9(11):2460-2473	non pertinent
4675	Scavuzzi et al.,	2013	Human reproduction (Oxford, England). 2013; 28(8):2118-2125	non pertinent
4676	Saxena et al.,	2013	PLoS One. 2013; 8(3):e57685	non pertinent
4677	Savaiano et al.,	2013	Nutrition j. 2013; 12:160	non pertinent
4678	Sassi et al.,	2013	Neuromodulation. 2013; 16(5):401-406	non pertinent
4679	Sarinopoulos et al.,	2013	Patient Educ Couns. 2013; 90(2):220-225	non pertinent
4680	Sansone et al.,	2013	J of orthopaedics and traumatology. 2013; 14(1):110-	non pertinent
4681	Sanga et al.,	2013	Pain. 2013; 154(10):1910-1919	non pertinent
4682	Sampath et al.,	2013	Leukemia research. 2013; 37(11):1461-1467	non pertinent
4683	Salminen et al.,	2013	Acta oncologica. 2013; 52(1):30-37	non pertinent
4684	Sahlström et al.,	2013	International J of Oral and Maxillofacial Surgery. 2013; 42(3):356-363	non pertinent
4685	Rowland et al.,	2013	Human vaccines and immunotherapeutics. 2013; 9(1):50-62	non pertinent
4686	Rosenstock et al.,	2013	Diabetes care. 2013; 36(3):498-504	non pertinent
4687	Rosati et al.,	2013	Cancer chemotherapy and pharmacology. 2013; 71(1):257-264	non pertinent
4688	Rodrigues et al.,	2013	Arg Bras Cardiol. 2013; 101(2):149-153	non pertinent
4689	Roddy et al.,	2013	Br J Gen Pract. 2013; 63(607):e141-148	non pertinent

4690	Rochas et al.,	2013	Cerebral cortex (new york, N.Y. : 1991). 2014; 23(7):1517-1525	non pertinent
4691	Robinson et al.,	2013	Neuroimage. 2013; 78:217-223	non pertinent
4692	Robertson et al.,	2013	Breast cancer research. 2013; 15(2)	non pertinent
4693	Ring et al.,	2013	Biol Psychol. 2013; 93(3):373-376	non pertinent
4694	Rhee et al.,	2013	Endocrine j. 2013; 60(2):167-172	non pertinent
4695	Reichmann	2013	J of neurology. 2013; 260:6-7	non pertinent
4696	Reginster	2013	Osteoporosis international. 2013; 24(1 SUPPL 1):75-76	non pertinent
4697	Ravn et al.,	2013	J of pain research. 2013; 6:23-38	non pertinent
4698	Rauck et al.,	2013	Pain practice. 2013; 13(6):485-496	non pertinent
4699	Rajpal et al.,	2013	Advances in therapy. 2013; 30(10):907-923	non pertinent
4700	Radulescu et al.,	2013	PLoS One. 2013; 8(7):e68485	non pertinent
4701	Rabinak et al.,	2013	Biological psychiatry. 2013; 73(9 SUPPL 1):34	non pertinent
4702	Rabago et al.,	2013	American j of physical medicine & rehabilitation. 2013; 92(7):587-596	non pertinent
4703	Rabago et al.,	2013	Archives of physical medicine and rehabilitation. 2013; 94(11):2075-2082	non pertinent
4704	Rabago et al.,	2013	PM and r. 2013; 5(9):225-	non pertinent
4705	Quigley et al.,	2013	Vox sanguinis. 2013; 105:250-251	non pertinent
4706	Purdon et al.,	2013	Proc Natl Acad Sci U S A. 2013; 110(12):E1142-1151	non pertinent
4707	Pujade-Lauraine et al.,	2013	J of clinical oncology. 2013; 31(15 SUPPL 1)	non pertinent
4708	Predel et al.,	2013	BMC musculoskeletal disorders. 2013; 14	non pertinent
4709	Prasad et al.,	2013	Schizophrenia bulletin. 2013; 39(4):857-866	non pertinent

4710	Polacco et al.,	2013	European j of inflammation. 2013; 11(3):847-853	non pertinent
4711	Plow et al.,	2013	Trials. 2013; 14:241	non pertinent
4712	Pietsch et al.,	2013	Knee Surg Sports Traumatol Arthrosc. 2013; 21(10):2234-2240	non pertinent
4713	Pienta et al.,	2013	Investigational new drugs. 2013; 31(3):760-768	non pertinent
4714	Perry et al.,	2013	JAMA – j of the american medical association. 2013; 310(12):1248-1255	non pertinent
4715	Perrino et al.,	2013	Clinical drug investigation. 2013; 33(6):441-449	non pertinent
4716	Pereyra et al.,	2013	World j of gastroenterology. 2013; 19(31):5103-5110	non pertinent
4717	Pereira et al.,	2013	Experimental neurology. 2013; 239:248-255	non pertinent
4718	Perdikakis et al.,	2013	Skeletal Radiol. 2013; 42(1):99-105	non pertinent
4719	Pépin et al.,	2013	Vaccine. 2013; 31(47):5572-5578	non pertinent
4720	Peolsson et al.,	2013	Spine (Phila Pa 1976). 2013; 38(4):300-307	non pertinent
4721	Pelletier et al.,	2013	Basic and clinical pharmacology and toxicology. 2013; 113:17	non pertinent
4722	Peciña et al.,	2013	Neuropsychopharmacology. 2013; 38(4):639-646	non pertinent
4723	Pavia-Ruz et al.,	2013	Human vaccines and immunotherapeutics. 2013; 9(9):1978-1988	non pertinent
4724	Paulis et al.,	2013	Inflammation and allergy – drug targets. 2013; 12(1):61-67	non pertinent
4725	Patua et al.,	2013	Archives of gynecology and obstetrics. 2013; 288(6):1243-1248	non pertinent
4726	Park et al.,	2013	J of nutritional biochemistry. 2013; 24(7):1367-1372	non pertinent
4727	Parente et al.,	2013	International j of colorectal disease. 2013; 28(10):1423-1431	non pertinent

4728	Papanicolaou et al.,	2013	J of nutrition, health & aging. 2013; 17(6):533-543	non pertinent
4729	Pan et al.,	2013	Wei mian yu gan ran za zhi (J of microbiology, immunology, and infection). 2013; 46(6):448-455	non pertinent
4730	Packer et al.,	2013	JACC. Heart failure. 2013; 1(4):308-314	non pertinent
4731	Ouedraogo et al.,	2013	PLoS One. 2013; 8(11)	non pertinent
4732	Otsyula et al.,	2013	Malaria j. 2013; 12(1)	non pertinent
4733	Ostenson et al.,	2013	Diabetes, metabolic syndrome and obesity: targets and therapy. 2013; 6:171-185	non pertinent
4734	Orellana Silva et al.,	2013	Pain medicine (united states). 2013; 14(3):422-429	non pertinent
4735	Onesti et al.,	2013	European j of pain (London, England). 2013; 17(9):1347-1356	non pertinent
4736	Omair et al.,	2013	BMC musculoskeletal disorders. 2013; 14:105	non pertinent
4737	Oka et al.,	2013	Psychotherapy and psychosomatics. 2013; 82:78-79	non pertinent
4738	Ojurongbe et al.,	2013	J of infection in developing countries. 2013; 7(12):975-982	non pertinent
4739	Ohtori et al.,	2013	Spine. 2013; 38(8):E487-E492	non pertinent
4740	Ohtori et al.,	2013	Yonsei medical j. 2013; 54(5):1253-1258	non pertinent
4741	Odekerken et al.,	2013	The lancet. Neurology. 2013; 12(1):37-44	non pertinent
4742	Nwankwo et al.,	2013	Investigational new drugs. 2013; 31(3):653-660	non pertinent
4743	Ntr - Helmondt	2013	Netherlands Trial Register. 2013	non pertinent
4744	Ntr – Schotanus	2013	Netherlands Trial Register. 2013	non pertinent
4745	Ntr - Lith	2013	Netherlands Trial Register. 2013	non pertinent
4746	Novack et al.,	2013	J of ocular pharmacology and therapeutiics. 2013; 29(7):674-680	non pertinent

4747	Nolte et al.,	2013	Ann Hematol. 2013; 92(2):191-198	non pertinent
4748	Niesvizky et al.,	2013	Clinical cancer research. 2013; 19(8):2248-2256	non pertinent
4749	Nicholas et al.,	2013	Neurology. 2013; 80(1 MeetingAbstracts)	non pertinent
4750	Nguyen et al.,	2013	Gastrointestinal endoscopy. 2013; 78(6):892-901	non pertinent
4751	Newhouse et al.,	2013	Neuropsychopharmacology. 2013; 38(13):2632-2643	non pertinent
4752	Newcorn et al.,	2013	J of the American Academy of Child and Adolescent Psychiatry. 2013; 52(9):921-930	non pertinent
4753	Nesioonpour et al.,	2013	Hernia. 2013; 17(4):465-470	non pertinent
4754	Nes et al.,	2013	Patient education and counseling. 2013; 93(3):451-458	non pertinent
4755	Nelson et al.,	2013	Trials. 2013; 14:325	non pertinent
4756	Nct - Rustaden	2013	National Library of Medicine. 2013	non pertinent
4757	Nct – Ishibe et al.,	2013	Surg Endosc. 2017 Oct; 31(10):3890-3897	non pertinent
4758	Nct – Toritani et al.,	2013	Int J Colorectal Dis. 2019 Jul; 34(7):1211-1220	non pertinent
4759	Nct – Chiu	2013	National Library of Medicine. 2013	non pertinent
4760	Nct – Duarte	2013	National Library of Medicine. 2013	non pertinent
4761	Nct – Randelli et al.,	2013	J Arthroplasty. 2019 Jul; 34(7):1374-1381	non pertinent
4762	Nct – Longmuir	2013	National Library of Medicine. 2013	non pertinent
4763	Nct – Clinical Sciences & Operations Sanofi	2013	National Library of Medicine. 2013	non pertinent
4764	Nct – Hendler	2013	National Library of Medicine. 2013	non pertinent
4765	Nct – Camilleri	2013	National Library of Medicine. 2013	non pertinent
4766	Nct – Boyles	2013	National Library of Medicine. 2013	non pertinent
4767	Nct – Naranjo-Rodriguez	2013	National Library of Medicine. 2013	non pertinent
4768	Nct – Johnson	2013	National Library of Medicine. 2013	non pertinent
4769	Nct – Datta	2013	National Library of Medicine. 2013	non pertinent
4770	Nct – Paulus	2013	National Library of Medicine. 2013	non pertinent
4771	Nct – Curatolo	2013	National Library of Medicine. 2013	non pertinent

4772	Nct – Finnes et al.,	2013	J Occup Health Psychol. 2019 Feb.; 24(1):198-212	non pertinent
4773	Nct – AgoneX Biopharmaceuticals	2013	National Library of Medicine. 2013	non pertinent
4774	Nct – Somers et al.,	2013	JMIR Mhealth Uhealth. 2018 Mar; 6(3):e66	non pertinent
4775	Nct – Silva	2013	National Library of Medicine. 2013	non pertinent
4776	Nct – Lau	2013	National Library of Medicine. 2013	non pertinent
4777	Nct – Agarwal	2013	National Library of Medicine. 2013	non pertinent
4778	Nct – Haythornthwaite	2013	National Library of Medicine. 2013	non pertinent
4779	Nct – Sørensen	2013	National Library of Medicine. 2013	non pertinent
4780	Nct – Yarnitsky	2013	National Library of Medicine. 2013	non pertinent
4781	Nct – OSF Heathcare System	2013	National Library of Medicine. 2013	non pertinent
4782	Nct – DiProspero	2013	National Library of Medicine. 2013	non pertinent
4783	Nct – Tarragó	2013	National Library of Medicine. 2013	non pertinent
4784	Nct – Ehde	2013	National Library of Medicine. 2013	non pertinent
4785	Nct – Caumo	2013	National Library of Medicine. 2013	non pertinent
4786	Nct – Pickrell	2013	National Library of Medicine. 2013	non pertinent
4787	Nct – Straudi	2013	National Library of Medicine. 2013	non pertinent
4788	Nct – Thorn	2013	National Library of Medicine. 2013	non pertinent
4789	Nct – Thomas	2013	National Library of Medicine. 2013	non pertinent
4790	Nct – Simons	2013	National Library of Medicine. 2013	non pertinent
4791	Nct – Schlegel	2013	National Library of Medicine. 2013	non pertinent
4792	Naumann et al.,	2013	Strahlenther Onkol. 2013; 189(9):745-752	non pertinent
4793	Nasa et al.,	2013	Indian j of gastroenterology. 2013; 32(6):392-396	non pertinent
4794	Motomura et al.,	2013	PLoS One. 2013; 8(2):e56578	non pertinent
4795	Moreno-Duarte et al.,	2013	Topics in spinal cord injury rehabilitation. 2013; 19(1):9-10	non pertinent
4796	Moore et al.,	2013	Pain medicine (Malden, Mass.). 2013; 14(11):1730-1740	non pertinent
4797	Moodie et al.,	2013	Pain medicine (Malden, Mass.). 2013; 14(6):916-924	non pertinent

4798	Montag et al.,	2013	Eur Arch Psychiatry Clin Neurosci. 2013; 263 Suppl 2: 169-175	non pertinent
4799	Monfort et al.,	2013	Arthritis and rheumatism. 2013; 65:913-914	non pertinent
4800	Mondelli et al.,	2013	Neurophysiologie clinique. 2013; 43(4):205-215	non pertinent
4801	Mohebati et al.,	2013	Cancer prevention research (philadelphia, pa.). 2013; 6(7):646-655	non pertinent
4802	Mohammadi et al.,	2013	Clinical neuropharmacology. 2013; 36(6):179-184	non pertinent
4803	Moein et al.,	2013	Brain injury. 2013; 27(12):1454-1460	non pertinent
4804	Modabber et al.,	2013	Trials. 2013; 14:238	non pertinent
4805	Mochizuki et al.,	2013	Pain. 2013; 154(10):1989-1998	non pertinent
4806	Miner et al.,	2013	Acad Emerg Med. 2013; 20(4):330-337	non pertinent
4807	Miguel et al.,	2013	The lancet. Oncology. 2013; 14(11):1055-1066	non pertinent
4808	Meulenbeld et al.,	2013	Cancer chemotherapy and pharmacology. 2013; 72(4):909-916	non pertinent
4809	Messier et al.,	2013	BMC muskuloskeletal disorders. 2013; 14:208	non pertinent
4810	Meissner et al.,	2013	Anesthesiology. 2013; 119(4):941-953	non pertinent
4811	Meek et al.,	2013	Eur J Clin Pharmacol. 2013; 69(3):365-371	non pertinent
4812	Mease et al.,	2013	Therapeutic advances in musculoskeletal disease. 2013; 5(3):113-126	non pertinent
4813	McNaughton et al.,	2013	J Affect Disord. 2013; 148(1):104-111	non pertinent
4814	McKavanagh et al.,	2013	International j of cardiovascular imaging. 2013; 29(7):1547-1555	non pertinent
4815	McKavanagh et al.,	2013	European heart j. 2013; 34:320-321	non pertinent

4816	McAlindon et al.,	2013	Jama. 2013; 309(2):155-162	non pertinent
4817	Matthey et al.,	2013	Pain Physician. 2013; 16(5):E553-562	non pertinent
4818	Martinez et al.,	2013	Pain. 2013; 154(8):1197-1203	non pertinent
4819	Martin et al.,	2013	Arthritis research & therapy. 2013; 15(5)	non pertinent
4820	Marsh et al.,	2013	J Child Psychol Psychiatry. 2013; 54(8):900-910	non pertinent
4821	Markenson et al.,	2013	J of clinical rheumatology. 2013; 19(6):317-323	non pertinent
4822	Marhofer et al.,	2013	Br J Anaesth. 2013; 110(3):438-442	non pertinent
4823	Mansson et al.,	2013	Psychiatry research – neuroimaging. 2013; 214(3):229-237	non pertinent
4824	Mansson et al.,	2013	Biological psychiatry. 2013; 73(9 SUPPL 1):72	non pertinent
4825	Mansson et al.,	2013	Psychiatry Res. 2013; 214(3):229-237	non pertinent
4826	Manor et al.,	2013	European psychiatry. 2013; 28(6):386-391	non pertinent
4827	Malinka et al.,	2013	Clinical transplantation. 2013; 27(5):E538-545	non pertinent
4828	Ly et al.,	2013	Pain. 2013; 154(10):2072-2077	non pertinent
4829	Luo et al.,	2013	Human vaccines & immunotherapeutics. 2013; 9(8):1725-1734	non pertinent
4830	Lueken et al.,	2013	American j of psychiatry. 2013; 170(11):1345-1355	non pertinent
4831	Lougee et al.,	2013	J Bodyw Mov Ther. 2013; 17(1):59-68	non pertinent
4832	Lotsch et al.,	2013	Naunyn-schmiedeberg's archives of pharmacology. 2013; 386:48	non pertinent
4833	Lopez-Castellanos et al.,	2013	Movement disorders. 2013; 28:35-36	non pertinent
4834	Loebermann et al.,	2013	PLoS One. 2013; 8(8)	non pertinent

4835	Liu et al.,	2013	Clinical orthopaedics and related research. 2013; 471(2):562-568	non pertinent
4836	Liu et al.,	2013	CNS Drugs. 2013; 27(12):1093-1102	non pertinent
4837	Liu et al.,	2013	Obesity (silver spring, md.). 2013; 21(10):1991-1996	non pertinent
4838	Lind et al.,	2013	Am J Sports Med. 2013; 41(12):2753-2758	non pertinent
4839	Lind et al.,	2013	Arthroscopy – j of arthroscopic and related surgery. 2013; 29(10 SUPPL 1):e148	non pertinent
4840	Liaw et al.,	2013	International j of nursing studies. 2013; 50(7):883-894	non pertinent
4841	Li et al.,	2013	Chinese j of cancer research. 2013; 25(6):749-755	non pertinent
4842	Li et al.,	2013	J of the european academy of dermatology and venereology : JEADV. 2013; 28(12):1821-1826	non pertinent
4843	Lev et al.,	2013	Headache. 2013; 53(7):1054-1070	non pertinent
4844	Leknes et al.,	2013	Pain. 2013; 154(3):402-410	non pertinent
4845	Lee et al.,	2013	Pain medicine (Malden, Mass.). 2013; 14(1):5-13	non pertinent
4846	Lee et al.,	2013	Pain. 2013; 154(1):124-134	non pertinent
4847	Lee et al.,	2013	Korean j of anesthesiology. 2013; 64(2):138-142	non pertinent
4848	Lee et al.,	2013	J of gastroenterology and hepatology (australia). 2013; 28(11):1699-1704	non pertinent
4849	Leblicq et al.,	2013	Pediatr Blood Cancer. 2013; 60(5): 741-747	non pertinent
4850	Le Graverand et al.,	2013	Annals of the rheumatic diseases. 2013; 72(2):187-195	non pertinent
4851	Lathrop et al.,	2013	Contraception. 2013; 88(6):725-729	non pertinent

4852	Langhorst et al.,	2013	Alimentary pharmacology & therapeutics. 2013; 38(5):490-500	non pertinent
4853	Lang et al.,	2013	Clinical genitourinary cancer. 2013; 11(4):407-415	non pertinent
4854	Lalezari et al.,	2013	Antiviral therapy. 2013; 18(6):755-764	non pertinent
4855	Labus et al.,	2013	Gastroenterology. 2013; 145(6):1253-1261.e1253	non pertinent
4856	Laaksonen et al.,	2013	Alcohol and alcoholism (oxford, oxfordshire). 2013; 48(6):687-693	non pertinent
4857	Kullgren et al.,	2013	J of General Internal Medicine. 2013; 28:5-	non pertinent
4858	Kulkantrakorn et al.,	2013	Pain practice. 2013; 13(6):497-503	non pertinent
4859	Kuivalainen et al.,	2013	European j of pain (London, England). 2013; 17(9):1357-1364	non pertinent
4860	Kristjansdottir et al.,	2013	J Med Internet Res. 2013; 15(1):e5	non pertinent
4861	Krischak et al.,	2013	J of shoulder and elbow surgery. 2013; 22(9):1173-1179	non pertinent
4862	Krein et al.,	2013	J of medical internet research. 2013; 15(8):e181	non pertinent
4863	Kostandy et al.,	2013	Neonatal Netw. 2013; 32(4):274-280	non pertinent
4864	Kortekaas et al.,	2013	PLoS One. 2013; 8(4):e61926	non pertinent
4865	Kohn et al.,	2013	Am J Sports Med. 2013; 41(9):2128-2135	non pertinent
4866	Knolle et al.,	2013	Pain. 2013; 154(12):2729-2736	non pertinent
4867	Knezveic et al.,	2013	Regional anesthesia and pain medicine. 2013; 38(1)	non pertinent
4868	Klumpp et al.,	2013	Prog Neuropsychopharmacol Biol Psychiatry. 2013; 45:83-91	non pertinent
4869	Kloppenburg	2013	Annals of the rheumatic diseases. 2013; 72	non pertinent

4870	Klapwijk et al.,	2013	Horm Behav. 2013; 64(2):314-322	non pertinent
4871	Kircher et al.,	2013	Biol Psychiatry. 2013; 73(1):93-101	non pertinent
4872	King et al.,	2013	Ergonomics. 2013; 56(1):59-68	non pertinent
4873	Kim et al.,	2013	International j of medical sciences. 2013; 10(10):1327-1332	non pertinent
4874	Kim et al.,	2013	Clinical therapeutics. 2013; 35(7):934-940	non pertinent
4875	Kim et al.,	2013	Minerva Anestesiologica. 2013; 79(11):1248-1258	non pertinent
4876	Kim et al.,	2013	Clinical therapeutics. 2013; 35(9):1350-1360.e1351	non pertinent
4877	Kim et al.,	2013	Clinical therapeutics. 2013; 35(11):1734-1743	non pertinent
4878	Kim et al.,	2013	Primary care companion to the j of clinical psychiatry. 2013; 15(6)	non pertinent
4879	Kim et al.,	2013	J of alternative and complementary medicine. 2013; 19(7):A8	non pertinent
4880	Kim et al.,	2013	J Clin Anesth. 2013; 25(3):173-176	non pertinent
4881	Kilinc et al.,	2013	Annals of the rheumatic diseases. 2013; 72	non pertinent
4882	Kieninger et al.,	2013	BMC infectious diseases. 2013; 13(1)	non pertinent
4883	Kerlan et al.,	2013	Annales d'endocrinologie. 2013; 74(5-6):487-490	non pertinent
4884	Kelly et al.,	2013	J Am Coll Surg. 2013; 217(6):1080-1089	non pertinent
4885	Kaur et al.,	2013	J Behav Ther Exp Psychiatry. 2013; 44(4):469-476	non pertinent
4886	Karolczak et al.,	2013	Pharmacological research. 2013; 74:7-22	non pertinent
4887	Karadas et al.,	2013	European neurology. 2013; 70(1-2):95-101	non pertinent

4888	Kaliomäki et al.,	2013	European j of pain (London, England). 2013; 17(10):1465-1471	non pertinent
4889	Jungquist et al.,	2013	Sleep. 2013; 36:A266-267	non pertinent
4890	Jprn – Saori Nishio	2013	UMIN-CTR Clinical Trial. 2013	non pertinent
4891	Jprn – Chen-Ta Li	2013	UMIN-CTR Clinical Trial. 2013	non pertinent
4892	Jo et al.,	2013	American j of sports medicine. 2013; 41(10):2240-2248	non pertinent
4893	Jinzaki et al.,	2013	International j of cardiovascular imaging. 2013; 29(1 SUPPL):7-20	non pertinent
4894	Ji et al.,	2013	PLoS One. 2013; 8(2)	non pertinent
4895	Jeyamohan et al.,	2013	Clinical neurosurgery. 2013; 60:172-173	non pertinent
4896	Jetté et al.,	2013	Neurorehabilitation and neural repair. 2013; 27(7):636-643	non pertinent
4897	Jensen et al.,	2013	European j of contraception & reproductive health care. 2013; 18(4):274-283	non pertinent
4898	Jelsness-Jørgensen et al.,	2013	Scandinavian j of gastroenterology. 2013; 48(7):890-895	non pertinent
4899	Jayasudha	2013	Nurs J India. 2013; 104(1):10-13	non pertinent
4900	Jarcho et al.,	2013	Pain. 2013; 154(7):987-996	non pertinent
4901	Jacquet et al.,	2013	JIAS – J of the international aids society. 2013	non pertinent
4902	Janssen et al.,	2013	Vaccine. 2013; 31(46):5306-5313	non pertinent
4903	Jakobsen et al.,	2013	J of thoracic oncology. 2013; 8:390-391	non pertinent
4904	Jacek et al.,	2013	J of neuroimmunology. 2013; 255(1-2):85-91	non pertinent
4905	Isserles et al.,	2013	Brain Stimuli. 2013; 6(3):377-383	non pertinent
4906	Isrctn – Ross	2013	ISRCTN registry. 2013	non pertinent
4907	Isrctn – Fallon	2013	ISRCTN registry. 2013	non pertinent
4908	Isrctn – Aeberli	2013	ISRCTN registry. 2013	non pertinent
4909	Isrctn – Ryan	2013	ISRCTN registry. 2013	non pertinent

4910	Isrctn – de Bruin	2013	ISRCTN registry. 2013	non pertinent
4911	Ishii et al.,	2013	Brain Res. 2013; 1529-105-112	non pertinent
4912	Irct2013032212848N - Tahririan	2013	Iranian Registry of Clinical Trials. 2013	non pertinent
4913	Iorno et al.,	2013	Current medical research and opinion. 2013; 29(12):1685-1690	non pertinent
4914	Incayawar	2013	J of pain. 2013; 14(4 SUPPL 1):103	non pertinent
4915	Imbimbo et al.,	2013	Alzheimer disease and associated disorders. 2013; 27(3):278-286	non pertinent
4916	Hutson et al.,	2013	Lancet oncology. 2013; 14(13):1287-1294	non pertinent
4917	Hussey et al.,	2013	BMC pharmacology and toxicology. 2013; 14	non pertinent
4018	Huneke et al.,	2013	PLoS One. 2013; 8(10):e78278	non pertinent
4919	Hughes et al.,	2013	Obesity (silver spring, md.). 2013; 21(9):1782-1788	non pertinent
4920	Hughes et al.,	2013	Molecular genetics and metabolism. 2013; 109(3):269-275	non pertinent
4921	Huber et al.,	2013	Pain. 2013; 154(9):1509-1518	non pertinent
4922	Hougaard et al.,	2013	Scandinavian j of pain. 2013; 4(1):48-52	non pertinent
4923	Hostetler et al.,	2013	J Pharmacol Exp Ther. 2013; 347(2):478-486	non pertinent
4924	Hornboll et al.,	2013	J Psychopharmacol. 2013; 27(10):903-914	non pertinent
4925	Hooker et al.,	2013	Psychiatry Res. 2013; 213(2):99-107	non pertinent
4926	Honoris et al.,	2013	J of cardiovascular computed tomography. 2013; 7:89-90	non pertinent
4927	Hong et al.,	2013	Clinical cancer research. 2013; 19(17):4824-4831	non pertinent
4928	Hollander et al.,	2013	Diabetes care. 2013; 36(12):4022-4029	non pertinent
4929	Hoexter et al.,	2013	European neuropsychopharmacology. 2013; 23(7):569-580	non pertinent

4930	Hobl et al.,	2013	Circulation. 2013; 128(22 SUPPL 1)	non pertinent
4931	Hillmen et al.,	2013	British j of haematology. 2013; 162(1):62-73	non pertinent
4932	Henry et al.,	2013	Psychiatry research. 2013; 213(3):217-224	non pertinent
4933	Henningsson et al.,	2013	European neuropsychopharmacology. 2013; 23:214-215	non pertinent
4934	Hencel et al.,	2013	Neuro Endocrinol Lett. 2013; 34(3):217-221	non pertinent
4935	Hemstreet et al.,	2013	J of immunotherapy. 2013; 36(1):57-65	non pertinent
4936	Hellio le Graverand et al.,	2013	Ann Rheum Dis. 2013; 72(2):187-195	non pertinent
4937	Hellio le Graverand et al.,	2013	Semin Arthritis Rheum. 2013; 43(1):1-8	non pertinent
4938	He et al.,	2013	Eur Spine J. 2013; 22(10):2256-2263	non pertinent
4939	Hassan et al.,	2013	Medical forum monthly. 2013; 24(4):66-69	non pertinent
4940	Hart et al.,	2013	Arch Orthop Trauma Surg. 2013; 133(9):1295-1301	non pertinent
4941	Harrison et al.,	2013	British j of haematology. 2013; 162(2):229-239	non pertinent
4942	Harris et al.,	2013	Anesthesiology. 2013; 119(6):1453-1464	non pertinent
4943	Hao et al.,	2013	J of thoracic oncology. 2013; 8:48-49	non pertinent
4944	Han et al.,	2013	European j of endocrinology. 2013; 168(6):887-893	non pertinent
4945	Han et al.,	2013	Human vaccines and immunotherapeutics. 2013; 9(9):1971-1977	non pertinent
4946	Haefeli et al.,	2013	J Clin Neurophysiol. 2013; 30(3):291-298	non pertinent
4947	Haddad et al.,	2013	Contraception. 2013; 88(6):737-743	non pertinent
4948	Haaker et al.,	2013	Proc Natl Acad Sci U S A. 2013; 110(26):E2428-2436	non pertinent

4949	Gupta et al.,	2013	J of neurosurgical anesthesiology. 2013; 25(3):271-278	non pertinent
4950	Guereca et al.,	2013	J of pain. 2013; 14(4 SUPPL 1):47	non pertinent
4951	Guedj et al.,	2013	European j of nuclear medicine and molecular imaging. 2013; 40:217	non pertinent
4952	Guarany et al.,	2013	PLoS One. 2013; 8(2)	non pertinent
4953	Greif et al.,	2013	Heart (British Cardiac Society). 2013; 99(14):1004-1011	non pertinent
4954	Graudins et al.,	2013	Trials. 2013; 14(1)	non pertinent
4955	Grahn et al.,	2013	J of neurology. 2013; 260(7):1813-1821	non pertinent
4956	Grady et al.,	2013	Eur Neuropsychopharmacol. 2013; 23(5):368-378	non pertinent
4957	Gould et al.,	2013	Regulatory toxicology and pharmacology : RTP. 2013; 67(1):89-97	non pertinent
4958	Gorka et al.,	2013	Psychopharmacology. 2013; 229(1):141-154	non pertinent
4959	Gontero et al.,	2013	Urologic oncology: seminars and original investigations. 2013; 31(5):671-675	non pertinent
4960	Gong et al.,	2013	J of arthroplasty. 2013; 28(8):1301-1305	non pertinent
4961	Gobert et al.,	2013	Regional anesthesia and pain medicine. 2013; 38(5 SUPPL 1):E122	non pertinent
4962	Gitelman et al.,	2013	The lancet. Diabetes & endocrinology. 2013; 1(4):306-316	non pertinent
4963	Giorgi et al.,	2013	Clinical therapeutics. 2013; 35(9):1321-1336	non pertinent
4964	Gilron et al.,	2013	Clin J Pain. 2013; 29(2):124-131	non pertinent
4965	Gigante et al.,	2013	Helicobacter. 2013; 18:134	non pertinent
4966	Ghanizadeh et al.,	2013	Depression and anxiety. 2013; 30(11):1084-1088	non pertinent

4967	Geuter et al.,	2013	Neuroimage. 2013; 67:227-236	non pertinent
4968	Genovese et al.,	2013	J of endocrinological investigation. 2013; 36(8):606-616	non pertinent
4969	Geiger et al.,	2013	J of therapeutic ultrasound. Conference: 2nd european symposium on focused ultrasound therapy. Italy. Conference start: 20131010. Conference end: 20131011; 2	non pertinent
4970	Gammaitoni et al.,	2013	J of pain research. 2013; 6:565-570	non pertinent
4971	Galea et al.,	2013	J of neuroscience. 2013; 33(9):3981-3988	non pertinent
4972	Furl et al.,	2013	J Neurosci. 2013; 33(44):17435-17443	non pertinent
4973	French et al.,	2013	PLoS One. 2013; 8(6):e65471	non pertinent
4974	Freman et al.,	2013	Spine. 2013; 38(23):1986-1994	non pertinent
4975	Fountain et al.,	2013	Epilepsia. 2013; 54(1):58-65	non pertinent
4976	Fortin et al.,	2013	Phys Ther. 2013; 93(11):1540-1550	non pertinent
4977	Forkmann et al.,	2013	J Neurosci. 2013; 33(6):2571-2581	non pertinent
4978	Fonseca et al.,	2013	Case reports in oncology. 2013; 6(2):441-449	non pertinent
4979	Fisher et al.,	2013	European neuropsychopharmacology. 2013; 23:272-273	non pertinent
4980	Fisher et al.,	2013	Biological psychiatry. 2013; 73(9 SUPPL 1):104	non pertinent
4981	Fischer et al.,	2013	Pain medicine (Malden, Mass.). 2013; 14(9):1388-1399	non pertinent
4982	Finocchietti et al.,	2013	Med Biol Eng Comput. 2013; 51(1-2):113-122	non pertinent
4983	Findling et al.,	2013	J of child and adolescent psychopharmacology. 2013; 23(7):468-480	non pertinent

4984	Findling et al.,	2013	J of child and adolescent psychopharmacology. 2013; 23(1):11-21	non pertinent
4985	Findling et al.,	2013	J of child and adolescent psychopharmacology. 2013; 23(8):545-557	non pertinent
4986	Felson et al.,	2013	Arthritis and rheumatism. 2013; 65:717	non pertinent
4987	Felmingham et al.,	2013	Biol Psychiatry. 2013; 73(11):1059-1063	non pertinent
4988	Fardo et al.,	2013	PLoS One. 2013; 8(11):e81964	non pertinent
4989	Fallah et al.,	2013	Indian Pediatr. 2013; 50(2):233-235	non pertinent
4990	Euctr, N.L.	2013	EU Clinical Trials Register. 2013	non pertinent
4991	Euctr, G.B.	2013	EU Clinical Trials Register. 2013	non pertinent
4992	Euctr, E.S.	2013	EU Clinical Trials Register. 2013	non pertinent
4993	Euctr, E.S.	2013	EU Clinical Trials Register. 2013	non pertinent
4994	Euctr, D.K.	2013	EU Clinical Trials Register. 2013	non pertinent
4995	Euctr, D.E.	2013	EU Clinical Trials Register. 2013	non pertinent
4996	Euctr, D.E.	2013	EU Clinical Trials Register. 2013	non pertinent
4997	Ellingsen et al.,	2013	Proc Natl Acad Sci U S A. 2013; 110(44):17993-17998	non pertinent
4998	Elimian et al.,	2013	Prenatal diagnosis. 2013; 33(12):1158-1161	non pertinent
4999	Eberl et al.,	2013	BMC gastroenterology. 2013; 13:176	non pertinent
5000	Dy et al.,	2013	Investigational new drugs. 2013; 31(4):891-899	non pertinent
5001	Dubois et al.,	2013	Clinical J of Pain. 2013; 29(8):696-701	non pertinent
5002	Dryer et al.,	2013	Spine j. 2013; 13(9):30-	non pertinent
5003	Dreyer et al.,	2013	J of clinical investigation. 2013; 123(11):4654-4666	non pertinent
5004	Doukky et al.,	2013	International j of cardiovascular imaging. 2013; 29(5):1029-1037	non pertinent

5005	Dmitrovic et al.,	2013	Human reproduction (Oxford, England). 2013; 28(11):2958-2965	non pertinent
5006	Dittmann et al.,	2013	CNS Drugs. 2013; 27(12):1081-1092	non pertinent
5007	Dinning et al.,	2013	Gastroenterology. 2013; 144(5):61-	non pertinent
5008	Diler et al.,	2013	Psychiatry Res. 2013; 214(3):277-284	non pertinent
5009	Diers et al.,	2013	Schmerz (berlin, germany). 2013; 27(2):205-211	non pertinent
5010	Diemer et al.,	2013	European neuropsychopharmacology. 2013; 23(11):1551-1560	non pertinent
5011	Di Minno et al.,	2013	Experimental and clinical endocrinology & diabetes. 2013; 121(2):91-93	non pertinent
5012	Dhillon	2013	Biodrugs. 2013; 27(4):375-392	non pertinent
5013	Desai et al.,	2013	Thrombosis research. 2013; 132(3):341-345	non pertinent
5014	Derakhshanfar et al.,	2013	Acta medica Croatica. 2013; 67(5):401-405	non pertinent
5015	De Souza et al.,	2013	Medical science monitor. 2013; 19:716-722	non pertinent
5016	De Ridder et al.,	2013	World Neurosurg. 2013; 80(5):642-649.e641	non pertinent
5017	De Rezende et al.,	2013	J of psychopharmacology (Oxford, England). 2013; 27(12):1124-1133	non pertinent
5018	Davies et al.,	2013	Diabetes care. 2013; 36(5):1368-1376	non pertinent
5019	Dangers et al.,	2013	European respiratory j. 2013; 42	non pertinent
5020	Dahan et al.,	2013	Molecular medicine (Cambridge, Mass.). 2013; 19:334-345	non pertinent
5021	Cunningham et al.,	2013	J of cell communication and signaling. 2013; 7(1):76-	non pertinent
5022	Cui et al.,	2013	Molecular therapy. 2013; 21(7):1456-1463	non pertinent

5023	Creech et al.,	2013	Human vaccines & imunotherapeutics. 2013; 9(12):2548-2557	non pertinent
5024	Cote et al.,	2013	Sleep. 2013; 36:A97	non pertinent
5025	Corsetti et al.,	2013	Neurogastroenterology and motility. 2013; 25(4):e256-262	non pertinent
5026	Corr et al.,	2013	Psychopharmacology. 2013; 225(2):373-379	non pertinent
5027	Cognetti et al.,	2013	Cancer research. 2013; 73(24 SUPPL 1)	non pertinent
5028	Coghill et al.,	2013	European neuropsychopharmacology. 2013; 23(10):1208-1218	non pertinent
5029	Clauw et al.,	2013	Arthritis research & therapy. 2013; 15(4):R88	non pertinent
5030	Chiauzzi et al.,	2013	J of pain. 2013; 14(4 SUPPL 1):94	non pertinent
5031	Chi et al.,	2013	Chinese medical j. 2013; 126(9):1642-1646	non pertinent
5032	Chi - Huang	2013	Chinese Clinical Trial Registry. 2013	non pertinent
5033	Cheng et al.,	2013	Vaccine. 2013; 31(20):2471-2476	non pertinent
5034	Chaumeron et al.,	2013	Clinical orthopaedics and related research. 2013; 471(7):2284-2295	non pertinent
5035	Chase et al.,	2013	Spinal Cord. 2013; 51(11):847-851	non pertinent
5036	Chadha et al.,	2013	JAMA otolaryngology – head & neck surgery. 2013; 139(12):1301-1305	non pertinent
5037	Catanzaro et al.,	2013	Acta bio-medica. 2013; 84(1):30-37	non pertinent
5038	Casanueva et al.,	2013	Rheumatology international. 2013; 33(10):2665-2670	non pertinent
5039	Carre et al.,	2013	Soc Cogn Affect Neurosci. 2013; 8(2):224-229	non pertinent
5040	Carlorazo Rustia et al.,	2013	J of gastroenterology and hepatology. 2013; 28:211-	non pertinent
5041	Cariou et al.,	2013	Diabetes care. 2013; 36(10):2923-2930	non pertinent

5042	Candido et al.,	2013	Pain Physician. 2013; 16(5):497-511	non pertinent
5043	Camprubi-Robles et al.,	2013	J of neuroscience. 2013; 33(6):2582-2592	non pertinent
5044	Caliskan et al.,	2013	BMC anesthesiology. 2013; 13	non pertinent
5045	Cahn et al.,	2013	BMC pharmacology and toxicology. 2013; 14	non pertinent
5046	Busch et al.,	2013	Brain Stimul. 2013; 6(2):202-209	non pertinent
5047	Bundred et al.,	2013	Investigational new drugs. 2013; 31(4):949-958	non pertinent
5048	Bryan et al.,	2013	Int J Behav Nutr Phys Act. 2013; 10:139	non pertinent
5049	Bruhn et al.,	2013	BMJ Open. 2013; 3(4)	non pertinent
5050	Brown et al.,	2013	Clinical J of Pain. 2013; 29(3):233-244	non pertinent
5051	Briasoulis et al.,	2013	BMC Cancer. 2013; 13:263	non pertinent
5052	Boyce et al.,	2013	British j of clinical pharmacology. 2013; 76(5):689-698	non pertinent
5053	Boxer et al.,	2013	JACC. Heart failure. 2013; 1(1):84-90	non pertinent
5054	Bossong et al.,	2013	Eur Neuropsychopharmacol. 2013; 23(12):1687-1697	non pertinent
5055	Bos et al.,	2013	Psychoneuroendocrinology. 2013; 38(6):808-817	non pertinent
5056	Borja-Tabora et al.,	2013	BMC infectious diseases. 2013; 13(1)	non pertinent
5057	Borgwardt et al.,	2013	J of inherited metabolic disease. 2013; 36(6):1015-1024	non pertinent
5058	Borggraefe et al.,	2013	European j of paediatric neurology: EJPN	non pertinent
5059	Borckardt et al.,	2013	Clinical J of Pain. 2013; 29(11):925-928	non pertinent
5060	Boostani et al.,	2013	Iranian j of basic medical sciences. 2013; 16(3):213-216	non pertinent
5061	Bone et al.,	2013	J of clinical endocrinology and metabolism. 2013; 98(12):4691-4701	non pertinent

5062	Boll et al.,	2013	J of clinical oncology. 2013; 31(12):1522-1529	non pertinent
5063	Boiko et al.,	2013	Cornea. 2013; 32(10):1349-1353	non pertinent
5064	Block et al.,	2013	Nephron. Clinical practice. 2013; 123(1-2):93-101	non pertinent
5065	Blickman et al.,	2013	Pediatric radiology. 2013; 43:573-574	non pertinent
5066	Blasiak et al.,	2013	JAMA dermatology. 2013; 149(12):1392-1398	non pertinent
5067	Blacker et al.,	2013	Stroke research and treatment. 2013	non pertinent
5068	Biondi et al.,	2013	Pain Physician. 2013; 16(3):E237-246	non pertinent
5069	Bianchi et al.,	2013	The lancet. Respiratory medicine. 2013;1(5):377-385	non pertinent
5070	Bhatt et al.,	2013	Stroke. 2013; 44(12):e227	non pertinent
5071	Bethell et al.,	2013	PLoS One. 2013; 8(3)	non pertinent
5072	Bérczi	2013	Cardiovascular and interventional radiology. 2013; 36:182-183	non pertinent
5073	Benson et al.,	2013	Brain, behavior, and immunity. 2013; 32:e17	non pertinent
5074	Bembich et al.,	2013	Neonatology. 2013; 104(4):255-259	non pertinent
5075	Belczak et al.,	2013	Phlebology / venous forum of the royal society of medicine. 2013; 29(7):454-460	non pertinent
5076	Becerra et al.,	2013	J of pharmacology and experimental therapeutics. 2013; 345(1):41-51	non pertinent
5077	Bays et al.,	2013	American j of cardiology. 2013; 112(12):1885-1895	non pertinent
5078	Baudic et al.,	2013	J Psychiatr Res. 2013; 47(1):72-77	non pertinent
5079	Bateman et al.,	2013	J of pain research. 2013; 6:311-318	non pertinent
5080	Bareille et al.,	2013	Current drug therapy. 2013; 8(2):69-75	non pertinent

5081	Aziznejad et al.,	2013	J of babol university of medical sciences. 2013; 15(3):16-23	non pertinent
5082	Ascher et al.,	2013	J of the european academy of dermatology and venereology : JEADV. 2013; 28(12):1707-1715	non pertinent
5083	Arts et al.,	2013	BMC musculoskeletal disorders. 2013; 14:244	non pertinent
5084	Armstrong et al.,	2013	Clinical cancer research. 2013; 19(24):6891-6901	non pertinent
5085	Ansaldi et al.,	2013	Human vaccines & immunotherapeutics. 2013; 9(3):591-598	non pertinent
5086	Ang et al.,	2013	Clinical J of Pain. 2013; 29(9):747-754	non pertinent
5087	Anandakrishnan et al.,	2013	J canadien d'anesthesie (Canadian j of anaesthesia.). 2013; 60(11):1054-1060	non pertinent
5088	Amin et al.,	2013	Egyptian j of chest diseases and tuberculosis. 2013; 62(3):447-451	non pertinent
5089	Amadori et al.,	2013	The lancet. Oncology. 2013; 14(7):663-670	non pertinent
5090	Allen et al.,	2013	Health technology assessment. 2013; 17(57):1-139	non pertinent
5091	Allais et al.,	2013	Neurological sciences. 2013; 34(SUPPL 1):83-86	non pertinent
5092	Alaie et al.,	2013	Biological psychiatry. 2013; 73(9 SUPPL 1): 79	non pertinent
5093	Al-Aqil et al.,	2013	Poultry science. 2013; 92(1):33-40	non pertinent
5094	Ahmed Ali et al.,	2013	BMC gastroenterology. 2013; 13:49	non pertinent
5095	Afilalo et al.,	2013	Pain Physician. 2013; 16(1):27-40	non pertinent
5096	Adler et al.,	2013	PLoS One. 2013; 8(9)	non pertinent
5096	Actrn - Yang	2013	Australian New Zealand Clinical Trials Registry. 2013	non pertinent
5098	Ab Rashid et al.,	2013	Urology. 2013; 82(3):194-	non pertinent

5099	American College of Rheumatology	2013	Arthritis and rheumatism. 2013; 65(SUPPL 10):376-377	non pertinent
5100	Westin et al.,	2013	International j of biological markers. 2013; 28(3):233-241	non pertinent
5101	Karsdal et al.,	2013	Annals of the rheumatic diseases. 2013; 72(SUPPL 3)	non pertinent
5102	?	2013	Annals of the academy of medicine singapore. 2013; 42:1-	non pertinent
5103	Zhang et al.,	2012	Evidence-based complementary and alternative medicine. 2012	non pertinent
5104	Zhang et al.,	2012	J Nucl Med. 2012; 53(10):1573-1578	non pertinent
5105	Zhang et al.,	2012	Dissertation for doctoral degree of southern medical university (nanfang yike daxue boshi xuewei lunwen). 2012; i-vi	non pertinent
5106	Zhang et al.,	2012	Research in developmental disabilities. 2012; 33(4):1136-1146	non pertinent
5107	Zhang et al.,	2012	Acta oto-laryngologica. 2012; 132(6):583-589	non pertinent
5108	Zarraga et al.,	2012	Circulation: arrhythmia and electrophysiology. 2012; 5(4):739-747	non pertinent
5109	Yoshimura et al.,	2012	Experimental and therapeutic medicine. 2012; 3(3):457-462	non pertinent
5110	Yoon et al.,	2012	J of diabetes investigation. 2012; 3(3):309-317	non pertinent
5111	Yoon et al.,	2012	J Neurosurg Anesthesiology. 2012; 24(2):146-151	non pertinent
5112	Yang et al.,	2012	J of xi'an jiaotong university (medical sciences). 2012; 33(4):494-497	non pertinent
5113	Yang et al.,	2012	BMC Complement Altern Med. 2012;	non pertinent

5114	Williams et al.,	2012	Neuropsychopharmacology. 2012; 38:301-302	non pertinent
5115	Williams et al.,	2012	J of clinical psychiatry. 2012; 73(5):654-660	non pertinent
5116	Wieser et al.,	2012	Biol Psychol. 2012; 90(3):242-248	non pertinent
5117	Wibral et al.,	2012	PLoS One. 2012; 7(10)	non pertinent
5118	West et al.,	2012	J of oral and maxillofacial surgery. 2012; 70(9 SUPPL 2):e109-e110	non pertinent
5119	Weininger et al.,	2012	European j of radiology. 2012; 81(12):3703-3710	non pertinent
5120	Volz et al.,	2012	PLoS One. 2012; 7(3):e34273	non pertinent
5121	Volkmann et al.,	2012	The lancet. Neurology. 2012; 11(12):1029-1038	non pertinent
5122	Vissers	2012	Regional anesthesia and pain medicine. 2012; 37(5):E70-E72	non pertinent
5123	Vesikari et al.,	2012	Human vaccines and immunotherapeutics. 2012; 8(9):1283-1292	non pertinent
5124	Vesikari et al.,	2012	Human vaccines and immunotherapeutics. 2012; 8(12):1882-1891	non pertinent
5125	Versmessen et al.,	2012	BMC Cancer. 2012; 12:495	non pertinent
5126	Verbeek et al.,	2012	BMC Musculoskeletal Disord. 2012; 13:16	non pertinent
5127	Vazquez-Roque et al.,	2012	Gastroenterology. 2012; 142(5 SUPPL 1):132-133	non pertinent
5128	Vasilakis et al.,	2012	Orthopedics. 2012; 35(12):e1684-1691	non pertinent
5129	Vanelderen et al.,	2012	Regional anesthesia and pain medicine. 2012; 37(5):E98-E101	non pertinent
5130	Van Nieuwenhoven et al.,	2012	Eur J Gastroenterol Hepatol. 2012; 24(11):1259-1265	non pertinent
5131	Upadhyay et al.,	2012	Neuroimage. 2012; 59(4):3762-3773	non pertinent

5132	Ueberall et al.,	2012	Pain practice. 2012; 12:80	non pertinent
5133	Ueberall et al.,	2012	Current medical research and opinion. 2012; 28(10):1617-1634	non pertinent
5134	Tucker et al.,	2012	Gut. 2012; 61:A316	non pertinent
5135	Tousignant et al.,	2012	Disabil Rehabil. 2012; 34(3):196-201	non pertinent
5136	Torkildsen et al.,	2012	Archives of neurology. 2012; 69(8):1044-1051	non pertinent
5137	Tobinick et al.,	2012	CNS Drugs. 2012; 26(12):1051-1070	non pertinent
5138	Tillisch et al.,	2012	Aliment Pharmacol Ther. 2012; 35(3):360-367	non pertinent
5139	Thanh et al.,	2012	Malaria j. 2012; 11	non pertinent
5140	Taylor et al.,	2012	Pain. 2012; 153(6):1219-1225	non pertinent
5141	Tassorelli et al.,	2012	Neurorehabilitation and neural repair. 2012; 26(6):669	non pertinent
5142	Tao et al.,	2012	American j of psychiatry. 2012; 169(4):381-388	non pertinent
5143	Tang et al.,	2012	Clinical rheumatology. 2012; 31(7):1079-1086	non pertinent
5144	Tahtinen et al.,	2012	Pediatric infectious disease j. 2012; 31(12):1227-1232	non pertinent
5145	Suzuki et al.,	2012	Therapeutic advances in psychopharmacology. 2012; 2(6):227-234	non pertinent
5146	Strosberg et al.,	2012	Annals of oncology. 2012; 23(9):2335-2341	non pertinent
5147	Strawn et al.,	2012	J of the American Academy of Child and Adolescent Psychiatry. 2012; 51(6):642-651	non pertinent
5148	Stoppe et al.,	2012	Br J Anaesth. 2012; 108(1):80-88	non pertinent
5149	Stacchiotti et al.,	2012	J Clin Oncol. 2012; 30(9):914-920	non pertinent
5150	Spitz et al.,	2012	Prostate cancer and prostatic disease. 2012; 15(1):93-99	non pertinent
5151	Soravia et al.,	2012	Clinical EEG and neuroscience. 2012; 43(1):80	non pertinent

5152	Smith et al.,	2012	Soc Cogn Affect Neurosci. 2012; 7(7):831-840	non pertinent
5153	Skogseid et al.,	2012	Eur J Neurol. 2012; 19(4):610-615	non pertinent
5154	Sinha et al.,	2012	Anesth Analg. 2012; 115(1):202-206	non pertinent
5155	Siepe et al.,	2012	Eur Spine J. 2012; 21(11):2287-2299	non pertinent
5156	Sibunruang et al.,	2012	Clinical microbiology and infection. 2012; 18:238-239	non pertinent
5157	Shin et al.,	2012	Arthroscopy. 2012; 28(5):628-635	non pertinent
5158	Shigihara et al.,	2012	Brain Res. 2012; 1457:44-50	non pertinent
5159	Sheaves et al.,	2012	J of orthopaedic and sports physical therapy. 2012; 42(2):114-124	non pertinent
5160	Sharpe et al.,	2012	Psychother Psychosom. 2012; 81(3):145-152	non pertinent
5161	Shamim et al.,	2012	J of the pakistan medical association. 2012; 62(8):561-565	non pertinent
5162	Seymour et al.,	2012	J of neuroscience. 2012; 32(17):5833-5842	non pertinent
5163	Seo et al.,	2012	PLoS One. 2012; 7(6):e37808	non pertinent
5164	Selvan et al.,	2012	The scientific world j. 2012	non pertinent
5165	Selker et al.,	2012	American heart j. 2012; 163(3):315-322	non pertinent
5166	Schuurman et al.,	2012	Stereotactic and functional neurosurgery. 2012; 90:47-	non pertinent
5167	Schutz et al.,	2012	BMC Med. 2012; 10:78	non pertinent
5168	Schmitz et al.,	2012	Frontiers in Psychiatry. 2012; 3(AUG)	non pertinent
5169	Schabrun et al.,	2012	PLoS One. 2012; 7(12):e51298	non pertinent
5170	Sardar et al.,	2012	Spine j. 2012; 12(9 SUPPL 1):152	non pertinent
5171	Sala-Blanch et al.,	2012	Anesthesia and Analgesia. 2012; 114(5):1121-1127	non pertinent
5172	Rowbotham et al.,	2012	Pain. 2012; 153(4):862-868	non pertinent
5173	Rousseau et al.,	2012	Annals of oncology. 2012; 23(2):450-457	non pertinent

5174	Rousseau et al.,	2012	Paediatr Anaesth. 2012; 22(8):799-804	non pertinent
5175	Roomer et al.,	2012	Antiviral therapy. 2012; 17(3):509-517	non pertinent
5176	Roderick et al.,	2012	Rheumatology (united kingdom). 2012; 51:viii11-	non pertinent
5177	River et al.,	2012	Clin J Pain. 2012; 28(9):790-796	non pertinent
5178	Ridker et al.,	2012	Circulation. 2012; 126(23):2739-2748	non pertinent
5179	Ren et al.,	2012	Chin Med J (Engl). 2012; 125(9):1627-1632	non pertinent
5180	Reidler et al.,	2012	J of Pain. 2012; 13(5):450-458	non pertinent
5181	Reardon et al.,	2012	Cancer chemotherapy and pharmacology. 2012; 69(4):849-860	non pertinent
5182	Railhac et al.,	2012	Clinical rheumatology. 2012; 31(9):1347-1357	non pertinent
5183	Rabinak et al.,	2012	Biological psychiatry. 2012; 71(8 SUPPL 1):177	non pertinent
5184	Rabinak et al.,	2012	Neuropsychopharmacology. 2012; 38:287	non pertinent
5185	Quintard et al.,	2012	Annals of intensive care. 2012; 2(1)	non pertinent
5186	Qin et al.,	2012	J Neurosci. 2012; 32(12):4032-4041	non pertinent
5187	Poole et al.,	2012	Arthritis and rheumatism. 2012; 64:1111-	non pertinent
5188	Plumhans et al.,	2012	J Med Imaging Radiat Oncol. 2012; 56(5):532-537	non pertinent
5189	Pishvaian et al.,	2012	Cancer chemotherapy and pharmacology. 2012; 70(6):843-853	non pertinent
5190	Pfenningsdorf et al.,	2012	Clinical ophthalmology (auckland, N.Z.). 2012; 6(1):739-746	non pertinent
5191	Paul et al.,	2012	Global spine j. 2012; 2	non pertinent
5192	Patel et al.,	2012	Knee Surg Sports Traumatol Arthrosc. 2012; 20(6):1152-1158	non pertinent

5193	Patel et al.,	2012	Thorax. 2012; 67(5):418-425	non pertinent
5194	Pasha et al.,	2012	J of babol university of medical sciences. 2012; 14(1):71-76	non pertinent
5195	Park et al.,	2012	Alimentary pharmacology & therapeutics. 2012; 35(1):56-65	non pertinent
5196	Panahi et al.,	2012	Thescientificworld j. 2012:285274	non pertinent
5197	Paakkonen et al.,	2012	Pediatric infectious disease j. 2012; 31(5):436-438	non pertinent
5198	Ostergaard et al.,	2012	Vaccine. 2012; 30(4):774-783	non pertinent
5199	Osteras et al.,	2012	J Bodyw Mov Ther. 2012; 16(4):456-463	non pertinent
5200	Olsson et al.,	2012	Scandinavian j of pain. 2012; 3(3):183-	non pertinent
5201	Oliver et al.,	2012	Clinical and translational allergy. 2012; 2(1):11	non pertinent
5202	Olischar et al.,	2012	Neonatology. 2012; 101(4):293-300	non pertinent
5203	Ohtori et al.,	2012	Spine (Phila Pa 1976). 2012; 37(6):439-444	non pertinent
5204	Odekerken et al.,	2012	Movement disorders. 2012; 27:165	non pertinent
5205	Nystrom et al.,	2012	J of clinical endocrinology and metabolism. 2012; 97(9):3185-3195	non pertinent
5206	Nunley et al.,	2012	Spine (Phila Pa 1976). 2012; 37(6):445-451	non pertinent
5207	Ntr – Fernández	2012	Netherlands Trial Register. 2012	non pertinent
5208	Ntr - Nieuwhof	2012	Netherlands Trial Register. 2012	non pertinent
5209	Ntr - Dahan	2012	Netherlands Trial Register. 2012	non pertinent
5210	Nojima et al.,	2012	J of neuroscience. 2012; 32(4):1293-1300	non pertinent
5211	Nir et al.,	2012	Clin Neurophysiol. 2012; 123(3):605-612	non pertinent
5212	Niesters et al.,	2012	Anesthesiology. 2012; 117(4):868-877	non pertinent
5213	Nct - Buxbaum	2012	National Library of Medicine. 2012	non pertinent
5214	Nct – Ismail	2012	National Library of Medicine. 2012	non pertinent

5215	Nct – Richer	2012	National Library of Medicine. 2012	non pertinent
5216	Nct – Taylor	2012	National Library of Medicine. 2012	non pertinent
5217	Nct – van der Does	2012	National Library of Medicine. 2012	non pertinent
5218	Nct – Carletto et al.,	2012	Front Psychol. 2016 Apr; 7:526	non pertinent
5219	Nct – Soravia et al.,	2012	Depress Anxiety. 2018 Oct; 35(10):925-934	non pertinent
5220	Nct – Gouin	2012	National Library of Medicine. 2012	non pertinent
5221	Nct – Chastan	2012	National Library of Medicine. 2012	non pertinent
5222	Nct – Hunter	2012	National Library of Medicine. 2012	non pertinent
5223	Nct – Modi	2012	National Library of Medicine. 2012	non pertinent
5224	Nct – Rini	2012	National Library of Medicine. 2012	non pertinent
5225	Nct – Shani	2012	National Library of Medicine. 2012	non pertinent
5226	Nct – Wegener	2012	National Library of Medicine. 2012	non pertinent
5227	Nct – Airaksinen	2012	National Library of Medicine. 2012	non pertinent
5228	Nct – McCusker et al.,	2012	Can J Psychiatry. 2017 Jan; 62(1):67-72	non pertinent
5229	Nct – Hendler	2012	National Library of Medicine. 2012	non pertinent
5230	Nct – Borckardt	2012	National Library of Medicine. 2012	non pertinent
5231	Nayak et al.,	2012	African j of urology. 2012; 18(3):135-137	non pertinent
5232	Nave et al.,	2012	J of clinical psychiatry. 2012; 73(9):1179-1186	non pertinent
5233	Nathan et al.,	2012	J of clinical pharmacology. 2012; 52(10):1456-1467	non pertinent
5234	Mylona et al.,	2012	Cardiovascular and interventional radiology. 2012; 35(4):875-882	non pertinent
5235	Munoz Garcia et al.,	2012	Int J Oral Maxillofac Implants. 2012; 27(2):375-382	non pertinent
5236	Multanen et al.,	2012	J of bone and mineral research. 2012; 27	non pertinent
5237	Mukesh et al.,	2012	European j of surgical oncology. 2012; 38(10):918-924	non pertinent
5238	Moore et al.,	2012	American j of the medical sciences. 2012; 344(2):90-95	non pertinent
5239	Moont et al.,	2012	Brain Res. 2012; 1435:105-117	non pertinent

5240	Mondillon et al.,	2012	Neuropsychologia. 2012; 50(12):2869-2879	non pertinent
5241	Molica	2012	International j of hematologic oncology. 2012; 1(1):47-55	non pertinent
5242	Miguel et al.,	2012	Neuropsychopharmacology. 2012; 38:273-	non pertinent
5243	Mesa et al.,	2012	Value in health. 2012; 15(4):A227	non pertinent
5244	Meeusen et al.,	2012	Annals of neurology. 2012; 71(3):417-426	non pertinent
5245	McDonald et al.,	2012	Pain Med. 2012; 13(11):1397-1406	non pertinent
5246	McCabe	2012	International j of neuropsychopharmacology. 2012; 15:44	non pertinent
5247	Mauclere-Denost et al.,	2012	European j of endocrinology. 2012; 166(2):261-268	non pertinent
5248	Martini et al.,	2012	J of pain research. 2012; 5:51-59	non pertinent
5249	Mansour et al.,	2012	Osteoarthritis and cartilage. 2012; 20:47	non pertinent
5250	Manconi et al.,	2012	Annals of neurology. 2012; 71(6):834-844	non pertinent
5251	Malpeso et al.,	2012	Expert review of cardiovascular therapy. 2012; 10(2):155-157	non pertinent
5252	Maeoka et al.,	2012	Neurosci Lett. 2012; 512(1):12-16	non pertinent
5253	Madeddu et al.,	2012	Clinical nutrition (edinburgh, scotland). 2012; 31(2):176-182	non pertinent
5254	MacCoon et al.,	2012	Behav Res Ther. 2012; 50(1):3-12	non pertinent
5255	Lu et al.,	2012	Vaccine. 2012; 30(24):3526-3533	non pertinent
5256	Lopez et al.,	2012	Trials. 2012; 13	non pertinent
5257	Lonsdale et al.,	2012	BMC musculoskeletal disorders. 2012; 13:104	non pertinent
5258	Liu et al.,	2012	Chinese j of cardiology. 2012; 40(10):840-843	non pertinent
5259	Lischke et al.,	2012	Psychoneuroendocrinology. 2012; 37(9):1431-1438	non pertinent
5260	Linstedt et al.,	2012	Acta Anaesthesiol Scand. 2012; 56(6):762-769	non pertinent

5261	Lim et al.,	2012	Clinical endocrinology. 2012; 77(2):215-233	non pertinent
5262	Liekweg et al.,	2012	Supportive care in cancer. 2012; 20(11):2669-2677	non pertinent
5263	Li et al.,	2012	World chinese j of digestology. 2012; 20(24):2276-2280	non pertinent
5264	Lelic et al.,	2012	Eur J Pain. 2012; 16(6):827-837	non pertinent
5265	Lee et al.,	2012	Pain. 2012; 153(6):1301-1310	non pertinent
5266	Lee et al.,	2012	Complement Ther Med. 2012; 20(6):409-416	non pertinent
5267	Lee et al.,	2012	Arthroscopy. 2012; 28(1):34-42	non pertinent
5268	Lebon et al.,	2012	Appl Psychophysiol Biofeedback. 2012; 37(1):45-51	non pertinent
5269	Latif	2012	Malaysian orthopaedic j. 2012; 6:43-	non pertinent
5270	Labuschagne et al.,	2012	International j of neuropsychopharmacology. 2012; 15(7):883-896	non pertinent
5271	Kruger et al.,	2012	European psychiatry. 2012; 27	non pertinent
5272	Kraus et al.,	2012	Osteoarthritis and cartilage. 2012; 20(4):271-278	non pertinent
5273	Krahn et al.,	2012	Circulation: arrhytmia and electrophysiology. 2012; 5(5):933-940	non pertinent
5274	Kotsis et al.,	2012	Neurogastroenterology and motility. 2012; 24(10):935-e462	non pertinent
5275	Korovessis et al.,	2012	European j of orthopaedic surgery & traumatology: orthopedie traumatologie. 2012; 22(8):639-645	non pertinent
5276	Kocyigit et al.,	2012	Clin J Pain. 2012; 28(7):581-588	non pertinent
5277	Knorr et al.,	2012	Psychiatry Res. 2012; 200(2-3):354-360	non pertinent
5278	Kinsman et al.,	2012	Australian j of rural health. 2012; 20(2):59-66	non pertinent
5279	Kim et al.,	2012	American j of sports medicine. 2012; 40(4):815-821	non pertinent

5280	Kessous et al.,	2012	Archives of gynecology and obstetrics. 2012; 286(1):75-79	non pertinent
5281	Kemp et al.,	2012	Pain Physician. 2012; 15(5):E693-699	non pertinent
5282	Kawasaki et al.,	2012	J of cognitive neuroscience. 2012; 24(6):1358-1370	non pertinent
5283	Katz et al.,	2012	European neuropsychopharmacology. 2012; 22:220-221	non pertinent
5284	Karadas et al.,	2012	Balkan medical j. 2012; 29(2):184-187	non pertinent
5285	Kapural	2012	Regional anesthesia and pain medicine. 2012; 37(5 SUPPL 1):E56-60	non pertinent
5286	Kader et al.,	2012	Ortopedia, traumatologia, rehabilitiacja. 2012; 14(3):251-259	non pertinent
5287	Jones et al.,	2012	Arthritis and rheumatism. 2012; 64:418-	non pertinent
5288	Jeong et al.,	2012	Alimentary pharmacology & therapeutics. 2012; 35(9):1088-1096	non pertinent
5289	Jeong et al.,	2012	Anesthesiology. 2012; 116(5):1047-1056	non pertinent
5290	Jensen et al.,	2012	American j of clinical nutrition. 2012; 96(1):5-13	non pertinent
5291	Jensen et al.,	2012	Pain. 2012; 153(7):1495-1503	non pertinent
5292	Jakobsen et al.,	2012	J of cardiothoracic and vascular anesthesia. 2012; 26(6):1039-1047	non pertinent
5293	Isrctn - Torkington	2012	ISRCTN Registry. 2012	non pertinent
5294	Isrctn - Clark	2012	ISRCTN Registry. 2012	non pertinent
5295	Inagaki et al.,	2012	Neuroimage. 2012 , 59(4):3222-3226	non pertinent
5296	Iikuni et al.,	2012	J of bone and mineral metabolism. 2012; 30(6):674-682	non pertinent
5297	Huling et al.,	2012	J Oral Pathol Med. 2012; 41(2):149-152	non pertinent

5298	Huang et al.,	2012	Gastroenterology. 2012; 142(5 SUPPL 1):450	non pertinent
5299	Hu et al.,	2012	National medical j of china. 2012; 92(10):679-684	non pertinent
5300	Horneff et al.,	2012	Arthritis Res Ther. 2012; 14(5):R230	non pertinent
5301	Hoffmann et al.,	2012	New England j of medicine. 2012; 367(4):299-308	non pertinent
5302	Hindi Attar et al.,	2012	PLoS One. 2012; 7(8):e42397	non pertinent
5303	Henke et al.,	2012	J of clinical oncology. 2012; 30(15 SUPPL 1)	non pertinent
5304	Henke et al.,	2012	Onkologie. 2012; 35:139-	non pertinent
5305	Hendel et al.,	2012	Clinical cardiology. 2012; 35(6):354-358	non pertinent
5306	Heiney et al.,	2012	Cancer. 2012; 118(15):3822-3832	non pertinent
5307	Hawkey et al.,	2012	Alimentary pharmacology & therapeutics. 2012; 35(7):819-827	non pertinent
5308	Hatz et al.,	2012	Vaccine. 2012; 30(23):3470-3477	non pertinent
5309	Hashmi et al.,	2012	Molecular pain. 2012; 8:29	non pertinent
5310	Hamza et al.,	2012	Pain medicine (united states). 2012; 13(10):1304-1313	non pertinent
5311	Hampstead et al.,	2012	Neuropsychology. 2012; 26(3):385-399	non pertinent
5312	Gulec	2012	J of the european academy of dermatology and venereology: JEADV. 2012; 26(3):314-318	non pertinent
5313	Guilfoyle et al.,	2012	Spine J. 2012; 12(8):646-651	non pertinent
5314	Goff	2012	Neuropsychopharmacology. 2012; 38:21-22	non pertinent
5315	Godlewska et al.,	2012	Psychol Med. 2012; 42(12):2609-2617	non pertinent
5316	Gisbert et al.,	2012	Alimentary pharmacology & therapeutics. 2012; 35(8):941-947	non pertinent
5317	Gilman et al.,	2012	Neuropsychopharmacology. 2012; 37(2):467-477	non pertinent

5318	Giles et al.,	2012	J of clinical hypertension. 2012; 14(5):299-306	non pertinent
5319	George et al.,	2012	Alcoholism: clinical and experimental research. 2012; 36:269A	non pertinent
5320	Geliebter et al.,	2012	Appetite. 2012; 59(3):956-959	non pertinent
5321	Gedde-Dahl et al.,	2012	Complement Ther Clin Pract. 2012; 18(1):60-65	non pertinent
5322	Gamble et al.,	2012	Pediatr Emerg Care. 2012; 28(10):1003-1008	non pertinent
5323	Gabbay et al.,	2012	Pediatrics. 2012; 129(5):e1439-e1500	non pertinent
5324	Fukase et al.,	2012	Vaccine. 2012; 30(33):5030-5037	non pertinent
5325	Fujimoto et al.,	2012	Circ J. 2012; 76(10):2419-2425	non pertinent
5326	French et al.,	2012	Epilepsy currents. 2012; 12(1 SUPPL 1)	non pertinent
5327	Fonteyne et al.,	2012	Int J Radiat Oncol Biol Phys. 2012; 83(1):46-52	non pertinent
5328	Finan et al.,	2012	Clin J Pain. 2012; 28(8):687-692	non pertinent
5329	Feng et al.,	2012	J of clinical neuroscience. 2012; 19(8):1130-1133	non pertinent
5330	Fasmer et al.,	2012	J Acupunct Meridian Stud. 2012; 5(1):15-20	non pertinent
5331	Fairhurst et al.,	2012	PLoS One. 2012; 7(10):e48711	non pertinent
5332	Eudract number - Dörfler	2012	EU Clinical Trial Register. 2012	non pertinent
5333	Euctr, I.T.	2012	EU Clinical Trials Register. 2012	non pertinent
5334	Euctr, E.S.	2012	EU Clinical Trials Register. 2012	non pertinent
5335	Euctr, E.S.	2012	EU Clinical Trials Register. 2012	non pertinent
5336	Euctr, D.E.	2012	EU Clinical Trials Register. 2012	non pertinent
5337	Euctr, C.Z.	2012	EU Clinical Trials Register. 2012	non pertinent
5338	England et al.,	2012	Stroke: a j of cerebral circulation. 2012; 43(2):405-411	non pertinent
5339	Elwir et al.,	2012	Gastrointestinal endoscopy. 2012; 75(4):AB308-	non pertinent
5340	Ellingson et al.,	2012	J Pain. 2012; 13(2):195-206	non pertinent
5341	Edginton et al.,	2012	Clinical pharmacology and therapeutics. 2012; 91:45-46	non pertinent

5342	Ebert et al.,	2012	Am J Sports Med. 2012; 40(7):1527-1537	non pertinent
5343	Duval et al.,	2012	BMC infectious diseases. 2012; 12	non pertinent
5344	Durmus et al.,	2012	J Back Musculoskeletal Rehabil. 2012; 25(4):275-284	non pertinent
5345	Dubsky et al.,	2012	J of clinical oncology. 2012; 30(7):722-728	non pertinent
5346	Drks - Martin	2012	Deutsches Register Klinischer Studien. 2012	non pertinent
5347	Drescher et al.,	2012	Headache. 2012; 52(4):573-581	non pertinent
5348	Drake et al.,	2012	PLoS One. 2012; 7(4)	non pertinent
5349	Downar et al.,	2012	Biological psychiatry. 2012; 71(8):28-	non pertinent
5350	Diers et al.,	2012	Exp Brain Res. 2012; 218(4):619-628	non pertinent
5351	Demyttenaere et al.,	2012	Current medical research and opinion. 2012; 28(1):41-48	non pertinent
5352	Demir et al.,	2012	Rev Bras Anestesiol. 2012; 62(4):511-519	non pertinent
5353	Dellapina et al.,	2012	Pain. 2012; 153(11):2267-2273	non pertinent
5354	Della Cioppa et al.,	2012	Human vaccines and immunotherapeutics. 2012; 8(2):216-227	non pertinent
5355	Del-Ben et al.,	2012	Psychopharmacol. 2012; 26(4):443-451	non pertinent
5356	Deakin et al.,	2012	Neuropsychopharmacology. 2012; 38:209-210	non pertinent
5357	Deakin et al.,	2012	International j of neuropsychopharmacology. 2012; 15:144	non pertinent
5358	De Wijkerslooth et al.,	2012	Gut. 2012; 61(11):1552-1559	non pertinent
5359	Davis et al.,	2012	Age Ageing. 2012; 41(4):495-501	non pertinent
5360	Dasilva et al.,	2012	Headache. 2012; 52(8):1283-1295	non pertinent
5361	Damiano et al.,	2012	Human vaccines and immunotherapeutics. 2012; 8(10):1425-1430	non pertinent

5362	Daly et al.,	2012	Archives of general psychiatry. 2012; 69(10):1003-1013	non pertinent
5363	Cortes et al.,	2012	New England j of medicine. 2012; 367(22):2075-2088	non pertinent
5364	Collins et al.,	2012	Am J Obstet Gynecol. 2012; 206(5):438.e431-436	non pertinent
5365	Cluze et al.,	2012	Annals of oncology. 2012; 23(4):882-890	non pertinent
5366	Citerio et al.,	2012	Eur J Anaesthesiol. 2012; 29(8):371-379	non pertinent
5367	Chung et al.,	2012	J of gastroenterology and hepatology. 2012; 27(11):1675-1680	non pertinent
5368	Chua et al.,	2012	Brain injury. 2012; 26(4-5):492-493	non pertinent
5369	Chu et al.,	2012	J of neurogastroenterology and motility. 2012; 18(3):305//316	non pertinent
5370	Christanell et al.,	2012	Sports medicine, arthroscopy, rehabilitation, therapy and technology. 2012; 4(1)	non pertinent
5371	Cho et al.,	2012	Cancer. 2012; 118(24):6055-6062	non pertinent
5372	Cheng et al.,	2012	J of cognitive neuroscience. 2012; 24(6):1411-1419	non pertinent
5373	Chen et al.,	2012	J Ocul Pharmacol Ther. 2012; 28(4):414-419	non pertinent
5374	Chan et al.,	2012	PLoS One. 2012; 7(6):e40215	non pertinent
5375	Chambers et al.,	2012	Clinical cancer research. 2012; 18(9):2668-2678	non pertinent
5376	Cardoso et al.,	2012	Int J Radiat Oncol Biol Phys. 2012; 82(3):1285-1291	non pertinent
5377	Cao et al.,	2012	Trials. 2012; 13:131	non pertinent
5378	Camilleri et al.,	2012	American j of physiology. Gastrointestinal and liver physiology. 2012; 303(1):G120-128	non pertinent

5379	Calvanese et al.,	2012	Open colorectal cancer j. 2012; 5(1):9-14	non pertinent
5380	Buttagat et al.,	2012	Complement Ther Med. 2012; 20(4):167-174	non pertinent
5381	Busch et al.,	2012	Pain Med. 2012; 13(2):215-228	non pertinent
5382	Buijze et al.,	2012	J of hand surgery. 2012; 37(8):24-	non pertinent
5383	Brovold et al.,	2012	Arch Gerontol Geriatr. 2012; 55(2):453-459	non pertinent
5384	Brakemeier et al.,	2012	Nephrology dialysis transplantation. 2012; 27(1):423-428	non pertinent
5385	Bossong et al.,	2012	J of cognitive neuroscience. 2012; 24(3):588-599	non pertinent
5386	Borglum et al.,	2012	Reg Anesth Pain Med. 2012; 37(3):294-301	non pertinent
5387	Blow et al.,	2012	Alcoholism: clinical and experimental research. 2012; 36:24A-	non pertinent
5388	Beusterien et al.,	2012	Health and quality of life outcomes. 2012; 10	non pertinent
5389	Benson et al.,	2012	Pain. 2012; 153(4):794-799	non pertinent
5390	Behmanesth et al.,	2012	Koomesh. 2012; 14(3):309-315	non pertinent
5391	Barti et al.,	2012	Arch Orthop Trauma Surg. 2012; 132(1):41-50	non pertinent
5392	Barraclough	2012	J of thoracic oncology. 2012; 7(9 SUPPL 3):159	non pertinent
5393	Azedevo Silva et al.,	2012	Revista brasileira de anestesiologia. 2012; 62(1):3-9	non pertinent
5394	Aversa et al.,	2012	J of maternal-fetal and neonatal medicine. 2012; 25:134	non pertinent
5395	Aupperle et al.,	2012	Frontiers in human neuroscience. 2012; 6(MAR)	non pertinent
5396	Arndt et al.,	2012	Orthopaedics and traumatology: surgery and research. 2012; 98(SUPPL 6):131-138	non pertinent
5397	Armstrong et al.,	2012	J Nutr. 2012; 142(2):382-388	non pertinent

5398	Armitage et al.,	2012	Anxiety Stress Coping. 2012; 25(2):155-165	non pertinent
5399	Aluwini et al.,	2012	Radiotherapy and oncology. 2012; 103:84-85	non pertinent
5400	Alegre et al.,	2012	Pain. 2012; 153(7):1382-1389	non pertinent
5401	Al-Shoha et al.,	2012	Spine. 2012; 37(25):E1567-E1571	non pertinent
5402	Akbar et al.,	2012	BMC complementary and alternative medicine. 2012; 12	non pertinent
5403	Alkawa et al.,	2012	J of rheumatology. 2012; 39(1):167-173	non pertinent
5404	Ahn et al.,	2012	Hepatology international. 2012; 6(4):696-706	non pertinent
5405	Agren et al.,	2012	Science. 2012; 337(6101):1550-1552	non pertinent
5406	Actrn – Lehman	2012	Australian New Zealand Clinical Trials Registry. 2012	non pertinent
5407	Acheson et al.,	2012	Human psychopharmacology. 2012; 27(4):419-427	non pertinent
5408	Ziefle et al.,	2011	J of immunotherapy. 2011; 34(4):403-408	non pertinent
5409	Zhou et al.,	2011	Fertil Steril. 2011; 95(3):900-905	non pertinent
5410	Zhao et al.,	2011	Drug and alcohol dependence. 2011; 118(2-3):224-229	non pertinent
5411	Zhao et al.,	2011	Zhongguo wie zhong bing ji jiu yi xue (Chinese critical care medicine). 2011; 23(4):220-223	non pertinent
5412	Zeng et al.,	2011	Chinese j of oncology. 2011; 33(7):517-519	non pertinent
5413	Zanette et al.,	2011	SAAD Dig. 2011; 27:8-15	non pertinent
5414	Zaffagnini et al.,	2011	Am J Sports Med. 2011; 39(5):977-985	non pertinent
5415	Yoo et al.,	2011	European child & adolescent psychiatry. 2011; 20(3):127-135	non pertinent

5416	Yasuda et al.,	2011	J of diabetes investigation. 2011; 2(2):132-139	non pertinent
5417	Yang	2011	Drugs. 2011; 71(15):2067-2077	non pertinent
5418	Wyndaele et al.,	2011	BJU international. 2011; 107(4):603-611	non pertinent
5419	Wolfensberger et al.,	2011	Nucl Med Commun. 2011; 32(10):896-902	non pertinent
5420	Willy et al.,	2011	J of orthopaedic and sports physical therapy. 2011; 41(9):625-632	non pertinent
5421	Wigal et al.,	2011	J of child and adolescent psychopharmacology. 2011; 21(2):121-131	non pertinent
5422	Wehrfritz et al.,	2011	European j of pain (London, England). 2011; 15(9):907-912	non pertinent
5423	Wang et al.,	2011	PLoS One. 2011; 6(8):e23536	non pertinent
5424	Wang et al.,	2011	Lancet. 2011; 377(9778):1663-1672	non pertinent
5425	Wang et al.,	2011	Chinese medical j. 2011; 124(24):4144-4148	non pertinent
5426	Walter et al.,	2011	Naunyn-Schmiedeberg's archieves of pharmacology. 2011; 383:75	non pertinent
5427	Wallentine et al.,	2011	Anesth Analg. 2011; 113(4):738-741	non pertinent
5428	Wallach et al.,	2011	Ireael j of psychiatry and related sciences. 2011; 48(2):91-97	non pertinent
5429	Von Wagner et al.,	2011	Eur Radiol. 2011; 21(10):2046-2055	non pertinent
5430	Vokurka et al.,	2011	Medical science monitor. 2011; 17(10):CR572-CR576	non pertinent
5431	Verstovsek et al.,	2011	Blood. 2011; 118(21)	non pertinent
5432	Verstovsek et al.,	2011	Blood. 2011; 118(21)	non pertinent
5433	Veldhuyzen et al.,	2011	Alimentary pharmacology & therapeutics. 2011; 34(7):714-723	non pertinent

5434	Van Noord et al.,	2011	Pulmonary pharmacology & therapeutics. 2011; 24(6):666-672	non pertinent
5435	Van den Broeke et al.,	2011	Mol Pain. 2011; 7:28	non pertinent
5436	Van den Berg et al.,	2011	Psycho-oncology. 2011; 20:67-68	non pertinent
5437	Van Damme et al.,	2011	Vaccine. 2011; 29(35):5932-5939	non pertinent
5438	Tulloch et al.,	2011	Phyiotherapy (united kingdom). 2011; 97:e1261-e1262	non pertinent
5439	Torres et al.,	2011	American j of kidney diseases. 2011; 57(5):692-699	non pertinent
5440	Tompkins et al.,	2011	Knee Surg Sports Traumatol Arthrosc. 2011; 19(5):787-791	non pertinent
5441	Tettamanti et al.,	2011	Physiotherapy (united kingdom). 2011; 97(var.pagings):e1224-e1225	non pertinent
5442	Tadini et al.,	2011	J ect. 2011; 27(2):134-140	non pertinent
5443	Surguladze et al.,	2011	J Psychopharmacol. 2011; 25(6):722-733	non pertinent
5444	Strasser et al.,	2011	Clin Rheumatol. 2011; 30(5):623-632	non pertinent
5445	Stancak et al.,	2011	PLoS One. 2011; 6(5):e19744	non pertinent
5446	Staahl et al.,	2011	Basic ClinPharmacol Toxicol. 2011; 109(5):321-327	non pertinent
5447	Sripada et al.,	2011	Neuroimage. 2011; 55(1):371-380	non pertinent
5448	Sprenger et al.,	2011	Pain. 2011; 152(2):428-439	non pertinent
5449	Spironelli et al.,	2011	PLoS One. 2011; 6(9):e24932	non pertinent
5450	Soliman et al.,	2011	Middle East J Anaesthesiol. 2011; 21(3):325-334	non pertinent
5451	Smith et al.,	2011	Neurogastroenterol Motil. 2011; 23(7):648-e260	non pertinent
5452	Skriapas et al.,	2011	Urology. 2011; 77(4):905-908	non pertinent
5453	Simonsson et al.,	2011	Blood. 2011; 118(12):3228-3235	non pertinent
5454	Sieper et al.,	2011	Arthritis and rheumatism. 2011; 63(10	non pertinent

5455	Shukla et al.,	2011	Mol Pain. 2011; 7:45	non pertinent
5456	Short et al.,	2011	Pain. 2011; 152(11):2477-2484	non pertinent
5457	Shiratori et al.,	2011	Neurogastroenterol Motil. 2011; 23(4):323-329, e155-326	non pertinent
5458	Shahab et al.,	2011	Health psychol. 2011; 30(1):49-57	non pertinent
5459	Sedegah et al.,	2011	PLoS One. 2011; 6(10)	non pertinent
5460	Scott	2011	CNS Drugs. 2011; 25(12):1073-1085	non pertinent
5461	Schuster et al.,	2011	Physiotherapy (united kingdom). 2011; 97:e1116-	non pertinent
5462	Schunck et al.,	2011	J Psychopharmacol. 2011; 25(1):52-59	non pertinent
5463	Schoffski et al.,	2011	Lancet oncology. 2011; 12(11):1045-1052	non pertinent
5464	Schlimp et al.,	2011	Schmerz (berlin, germany). 2011; 25(3):290-295	non pertinent
5465	Satoh et al.,	2011	J of diabetes investigation. 2011; 2(6):457-463	non pertinent
5466	Ruutu et al.,	2011	Haematologica. 2011; 96(9):1344-1350	non pertinent
5467	Rutter et al.,	2011	Antiviral therapy. 2011; 16(8):1327-1333	non pertinent
5468	Rudwaleit et al.,	2011	J of rheumatology. 2011; 38(1):79-86	non pertinent
5469	Rubinstein et al.,	2011	CMAJ: Canadian Medical Association j. 2011; 183(13):E1033-E1037	non pertinent
5470	Rothrock et al.,	2011	Current medical research and opinion. 2011; 27(11):2185-2191	non pertinent
5471	Roth et al.,	2011	Postgraduate medicine. 2011; 123(6):180-188	non pertinent
5472	Roth et al.,	2011	International j of andrology. 2011; 34(6 PART 1):541-547	non pertinent
5473	Rota et al.,	2011	J of headache and pain. 2011; 12(3):339-345	non pertinent
5474	Rose et al.,	2011	BMC complementary and alternative medicine. 2011; 11:45	non pertinent

5475	Romics et al.,	2011	Urologia internationalis. 2011; 86(1):102-109	non pertinent
5476	Rogers et al.,	2011	American j of cardiology. 2011; 107(5):643-650	non pertinent
5477	Roessler et al.,	2011	European j of pain supplements. 2011; 5(1):156	non pertinent
5478	Richebe et al.,	2011	J Cardiothorac Vasc Anesth. 2011; 25(6):917-925	non pertinent
5479	Rezvani et al.,	2011	Haematologica. 2011; 96(3):432-440	non pertinent
5480	Renkawitz et al.,	2011	BMC musculoskeletal disorders. 2011; 12:192	non pertinent
5481	Reinke et al.,	2011	Transplantation proceedings. 2011; 43(5):1641-1646	non pertinent
5482	Razak et al.,	2011	British j of cancer. 2011; 104(5):756-762	non pertinent
5483	Randelli et al.,	2011	J of shoulder and elbow surgery. 2011; 20(4):518-528	non pertinent
5484	Raj et al.,	2011	Indian j of physiology and pharmacology. 2011; 55(5 SUPPL 1:63-64	non pertinent
5485	Pinera et al.,	2011	Eur Spine J. 2011; 20 Suppl. 3: 408-414	non pertinent
5486	Phatak et al.,	2011	J Pediatr Gastroenterol Nutr. 2011; 53(1):71-74	non pertinent
5487	Perez-Monteverde et al.,	2011	International j of clinical practice. 2011; 65(9):930-938	non pertinent
5488	Pelkonen et al.,	2011	Lancet infectious diseases. 2011; 11(8):613-621	non pertinent
5489	Pavelka et al.,	2011	Osteoarthritis and cartilage. 2011; 19(11):1294-1300	non pertinent
5490	Park et al.,	2011	Acta anaesthesiologica Scandinavica. 2011; 55(1):87-91	non pertinent
5491	Park et al.,	2011	Cancer chemotherapy and pharmacology. 2011; 68(5):1263-1271	non pertinent

5492	Parhizgar et al.,	2011	European j of medical research. 2011; 16:59	non pertinent
5493	Pareyson et al.,	2011	The lancet. Neurology. 2011; 10(4):320-328	non pertinent
5494	Pareyson et al.,	2011	J of the peripheral nervous system. 2011; 16:104-105	non pertinent
5495	Pareyson et al.,	2011	Lancet neurology. 2011; 10(4):320-328	non pertinent
5496	Pakzad et al.,	2011	Physiotherapy (united kingdom). 2011; 97(var.pagings):e956	non pertinent
5497	Padula et al.,	2011	Alcohol and alcoholism. 2011; 46(5):547-552	non pertinent
5498	Otten et al.,	2011	J of rheumatology. 2011; 38(10):2258-2263	non pertinent
5499	Osteras et al.,	2011	Physiotherapy (united kingdom). 2011; 97:e946-e947	non pertinent
5500	Orvieto et al.,	2011	BJU international. 2011; 108(7):1185-1189	non pertinent
5501	Olesen et al.,	2011	Aliment Pharmacol Ther. 2011; 34(8):878-887	non pertinent
5502	Oh et al.,	2011	Urology. 2011; 78(3):626-630	non pertinent
5503	Oh et al.,	2011	Clinics in orthopedic surgery. 2011; 3(1):55-61	non pertinent
5504	Ogura et al.,	2011	Alternative therapies in health and medicine. 2011; 17(6):12-17	non pertinent
5505	O'Connell et al.,	2011	Physiotherapy (united kingdom). 2011; 97(var.pagings):e913-e914	non pertinent
5506	Nunes et al.,	2011	Revista brasileira de anestesiologia. 2011; 61(3):304-310	non pertinent
5507	Ntr – Wijnhoven	2011	Netherlands Trial Register. 2011	non pertinent
5508	Ntr - Boonen	2011	Netherlands Trial Register. 2011	non pertinent
5509	Novotna et al.,	2011	European j of neurology. 2011; 18(9):1122-1131	non pertinent

5510	Norholt et al.,	2011	International j of clinical pharmacology and therapeutics. 2011; 49(12):722-729	non pertinent
5511	Nicholson et al.,	2011	Health technology assessment. 2011; 14(55):193-334	non pertinent
5512	Newberg et al.,	2011	Clin Ther. 2011; 33(12):1894-1903	non pertinent
5513	Neves et al.,	2011	Anais brasileiros de dermatologia. 2011; 86(6):1092-1101	non pertinent
5514	Nct – Heiney	2011	National Library of Medicine. 2011	non pertinent
5515	Nct – De Neve	2011	National Library of Medicine. 2011	non pertinent
5516	Nct – Kingwell	2011	National Library of Medicine. 2011	non pertinent
5517	Nct – Yerramasu et al.,	2011	Eur Heart J Cardiovasc Imaging. 2014 Aug; 15(8):886-92	non pertinent
5518	Nct – Rosenfeld	2011	National Library of Medicine. 2011	non pertinent
5519	Nct – Pickering	2011	National Library of Medicine. 2011	non pertinent
5520	Nct – Wai	2011	National Library of Medicine. 2011	non pertinent
5521	Nct – Pitman	2011	National Library of Medicine. 2011	non pertinent
5522	Nct – Essving	2011	National Library of Medicine. 2011	non pertinent
5523	Nct – Hölzel et al.,	2011	Front Behav Neurosci. 2016 Jun; 10:124	non pertinent
5524	Nct – Gouin	2011	National Library of Medicine. 2011	non pertinent
5525	Nct – Cermak	2011	National Library of Medicine. 2011	non pertinent
5526	Nct – Choy	2011	National Library of Medicine. 2011	non pertinent
5527	Nct – Slonim	2011	National Library of Medicine. 2011	non pertinent
5528	Nct – Lee et al.,	2011	Arthritis Rheumatol. 2018 Aug; 70(8):1308-1318	non pertinent
5529	Nct – King	2011	National Library of Medicine. 2011	non pertinent
5530	Nct – Yarnitsky	2011	National Library of Medicine. 2011	non pertinent
5531	Nct – d_yarnitsky	2011	National Library of Medicine. 2011	non pertinent
5532	Nct – Söderlund	2011	National Library of Medicine. 2011	non pertinent
5533	Nct – Rosenblum	2011	National Library of Medicine. 2011	non pertinent
5534	Navari et al.,	2011	J of supportive oncology. 2011; 9(5):188-195	non pertinent

5535	Nabhan et al.,	2011	J of neurosurgery. 2011; 114(1):240-244	non pertinent
5536	Mosges et al.,	2011	Current medical research and opinion. 2011; 27(4):871-878	non pertinent
5537	Morris et al.,	2011	BMC Musculoskelet Disord. 2011; 12(1):85	non pertinent
5538	Möri et al.,	2011	J of bodywork and movement therapies. 2011; 15(3):309-318	non pertinent
5539	Moore et al.,	2011	European j of anaesthesiology. 2011; 28(6):427-432	non pertinent
5540	Mohanty et al.,	2011	Indian j of palliative care. 2011; 17(4):91-103	non pertinent
5541	Moe et al.,	2011	BMC musculoskeletal disorders. 2011; 257-	non pertinent
5542	Miller et al.,	2011	Academic emergency medicine. 2011; 18(5):458-467	non pertinent
5543	Mieres et al.,	2011	J Womens Health (Larchmt). 2011; 20(9):1261-1268	non pertinent
5544	Mhalla et al.,	2011	Pain. 2011; 152(7):1478-1485	non pertinent
5545	Mennemeier et al.,	2011	Laryngoscope. 2011; 121(4):815-822	non pertinent
5546	Mendonca et al.,	2011	J Pain. 2011; 12(5):610-617	non pertinent
5547	Melilli et al.,	2011	European j of drug metabolism and pharmacokinetics. 2011; 36(2):71-78	non pertinent
5548	Mehdikhani et al.,	2011	Acta cardiologica. 2011; 66(2):219-224	non pertinent
5549	Meara et al.,	2011	Cleft palate-craniofacial j. 2011; 48(6):690-694	non pertinent
5550	McPherson et al.,	2011	Osteoarthritis and cartilage. 2011; 19:35-36	non pertinent
5551	McLoughlin et al.,	2011	J Pain. 2011; 12(6):640-651	non pertinent
5552	McCarthy et al.,	2011	PLoS One. 2011; 6(9)	non pertinent
5553	McCann et al.,	2011	Psychopharmacology (Berl). 2011; 217(4):475-484	non pertinent

5554	Matthey et al.,	2011	Basic and clinical pharmacology and toxicology. 2011; 109:120	non pertinent
5555	Manheimer	2011	Fertility and sterility. 2011; 95(8):2456-2461	non pertinent
5556	Manani et al.,	2011	Minerva Stomatol. 2011; 60(7-8):365-381	non pertinent
5557	Lueken et al.,	2011	Eur Neuropsychophar macol. 2011; 21(7):516-525	non pertinent
5558	Lu et al.,	2011	Clinical drug investigation. 2011; 31(4):221-229	non pertinent
5559	Louzada et al.,	2011	Thromb Res. 2011; 128(6):530-535	non pertinent
5560	Lopez-Contreras et al.,	2011	Movement disorders. 2011; 26:217	non pertinent
5561	Loggia et al.,	2011	PLoS One. 2011; 6(11):e27764	non pertinent
5562	Locatelli	2011	NDT plus. 2011; 4(SUPPL 1):i3-i5	non pertinent
5563	Ljotsson et al.,	2011	BMC Gastroenterol. 2011; 11:110	non pertinent
5564	Linnebank et al.,	2011	Annals of neurology. 2011; 69(2):352-359	non pertinent
5565	Lindstedt et al.,	2011	PLoS One. 2011; 6(3):e18252	non pertinent
5566	Liang et al.,	2011	Acta anaesthesiologica Scandinavica. 2011; 55(9):1113-1117	non pertinent
5567	Li et al.,	2011	Chinese medical j. 2011; 124(19):3049-3053	non pertinent
5568	Lee et al.,	2011	Canadian j of anesthesia. 2011; 58(11):1001-1006	non pertinent
5569	Lee et al.,	2011	Physiotherapy (united kingdom). 2011; 97:e674-	non pertinent
5570	Leal et al.,	2011	Pain. 2011; 152(10):2357-2364	non pertinent
5571	Laslett et al.,	2011	Internal medicine j. 2011; 41:34-	non pertinent
5572	Lang et al.,	2011	Cancer immunology, immunotherapy. 2011; 60(10):1447-1460	non pertinent
5573	Ku et al.,	2011	PLoS One. 2011; 6(10):e25155	non pertinent

5574	Kross et al.,	2011	Proc Natl Acad Sci U S A. 2011; 108(15):6270-6275	non pertinent
5575	Kristjansdottir et al.,	2011	European j of pain supplements. 2011; 5(1):259-260	non pertinent
5576	Kret et al.,	2011	Neuropsychologia. 2011; 49(5):1187-1193	non pertinent
5577	Kolykhalov et al.,	2011	Neuroscience and behavioral physiology. 2011; 41(5):542-547	non pertinent
5578	Kollins et al.,	2011	Pediatrics. 2011; 127(6):e1406-1413	non pertinent
5579	Kjotrod et al.,	2011	Human reproduction (Oxford, England). 2011; 26(8):2045-2053	non pertinent
5580	Kim et al.,	2011	Anaesth Intensive Care. 2011; 39(3):418-425	non pertinent
5581	Kim et al.,	2011	Paediatr Anaesth. 2011; 21(4):394-398	non pertinent
5582	Kim et al.,	2011	J Vasc Surg. 2011; 54(2):316-324	non pertinent
5583	Kim et al.,	2011	Pediatric infectious disease j. 2011; 30(12):e235-e243	non pertinent
5584	Khan et al.,	2011	British j of cancer. 2011; 104(5):750-755	non pertinent
5585	Kct – Seung-Hyun Yoon	2011	CRIS – Clinical Research Information Service. 2011	non pertinent
5586	Karschner et al.,	2011	Clinical chemistry. 2011; 57(1):66-75	non pertinent
5587	Karim et al.,	2011	AIDS (london, england). 2011; 25(7):957-966	non pertinent
5588	Jung et al.,	2011	Acta Anaesthesiol Scand. 2011; 55(1):110-117	non pertinent
5589	Jprn, Umin -	2011	Japan Primary Registries Network. 2011	non pertinent
5590	Jprn, Umin -	2011	Japan Primary Registries Network. 2011	non pertinent
5591	Joseph et al.,	2011	Indian j of medical research, supplement. 2011; 133(5):529-534	non pertinent
5592	Jo et al.,	2011	Korean j of anesthesiology. 2011; 60(3):198-204	non pertinent

5593	Jo et al.,	2011	Am J Sports Med. 2011; 39(10):2082-2090	non pertinent
5594	Janne et al.,	2011	Clinical cancer research. 2011; 17(5):1131-1139	non pertinent
5595	Jadidi et al.,	2011	European j of oral sciences. 2011; 119(3):211-218	non pertinent
5596	Isrctn - Ropars	2011	ISRCTN Registry. 2011	non pertinent
5597	Isrctn – Koskina	2011	ISRCTN Registry. 2011	non pertinent
5598	Isrctn – Yiqun	2011	ISRCTN Registry. 2011	non pertinent
5599	Isrctn – Morno-Egea	2011	ISRCTN Registry. 2011	non pertinent
5600	Isrctn - Bögels	2011	ISRCTN Registry. 2011	non pertinent
5601	Hursel et al.,	2011	American j of clinical nutrition. 2011; 94(3):804-808	non pertinent
5602	Hunstman et al.,	2011	European spine j. 2011; 20(4):503-504	non pertinent
5603	Hubbard et al.,	2011	J Neurosci. 2011; 31(35):12491-12500	non pertinent
5604	Horst	2011	Physiotherapy (united kingdom). 2011; 97:e1516-1517	non pertinent
5605	Holtmann et al.,	2011	Digestion. 2011; 84(4):289-298	non pertinent
5606	Hoft et al.,	2011	J of infectious diseases. 2011; 204(6):845-853	non pertinent
5607	Hesse et al.,	2011	Neurologie und rehabilitation. 2011; 17(5-6):233-238	non pertinent
5608	Hess et al.,	2011	Proc Natl Acad Sci U S A. 2011; 108(9):3731-3736	non pertinent
5609	Henry et al.,	2011	Diabetes, obesity & metabolism. 2011; 13(9):850-858	non pertinent
5610	Hara et al.,	2011	Vaccine. 2011; 29(46):8323-8329	non pertinent
5611	Hadjigeorgiou et al.,	2011	Physiotherapy (united kingdom). 2011; 97:e442-e443	non pertinent
5612	Gu et al.,	2011	Circulation j. 2011; 75(4):955-963	non pertinent
5613	Gronlund et al.,	2011	Clinical drug investigation. 2011; 31(3):143-153	non pertinent

5614	Gronlund et al.,	2011	Antimicrobial agents and chemotherapy. 2011; 55(3):1063-1067	non pertinent
5615	Griffin et al.,	2011	J Neurol. 2011; 258(6):991-1000	non pertinent
5616	Graversen et al.,	2011	J Neural Eng. 2011; 8(5):056014	non pertinent
5617	Goral et al.,	2011	Anaesthesia. 2011; 66(12):1140-1145	non pertinent
5618	Gooch et al.,	2011	Behav Pharmacol. 2011; 22(4):354-361	non pertinent
5619	Gomez Conesa et al.,	2011	Physiotherapy (united kingdom). 2011; 97:e418	non pertinent
5620	Goldstein et al.,	2011	J of the American College of Cardiology. 2011; 58(14):1414-1422	non pertinent
5621	Goktay et al.,	2011	J of oral and maxillofacial surgery. 2011; 69(6):1594-1599	non pertinent
5622	Gatti et al.,	2011	J of orthopaedic and sports physical therapy. 2011; 41(8):542-552	non pertinent
5623	Garnock-Jones	2011	CNS Drugs. 2011; 25(5):435-445	non pertinent
5624	Gambus et al.,	2011	Anesth Analg. 2011; 112(2):331-339	non pertinent
5625	Gajdos et al.,	2011	Human vaccines. 2011; 7(5):549-556	non pertinent
5626	Fung et al.,	2011	Physiotherapy (united kingdom). 2011; 97:e419-	non pertinent
5627	Fujita et al.,	2011	J of bone and mineral metabolism. 2011; 29(5):588-597	non pertinent
5628	Froholdt et al.,	2011	Spine j. 2011; 11(8):718-725	non pertinent
5629	Fritz et al.,	2011	American j of respiratory and critical care medicine. 2011; 183(1 MeetingAbstracts)	non pertinent
5630	Frenck et al.,	2011	Vaccine. 2011; 29(34):5666-5674	non pertinent
5631	Foroughi et al.,	2011	Clin Biomech (Bristol, Avon). 2011; 26(1):71-77	non pertinent
5632	Fonseca et al.,	2011	Physiotherapy (united kingdom). 2011; 97:e350-	non pertinent

5633	Foley et al.,	2011	AJR Am J Roentgenol. 2011; 197(2):367-373	non pertinent
5634	Fitzpatrick et al.,	2011	Bone. 2011; 49(4):845-852	non pertinent
5635	Fedak et al.,	2011	Annals of thoracic surgery. 2011; 92(4):1444-1450	non pertinent
5636	Euctr, P.T.	2011	EU Clinical Trials Register. 2011	non pertinent
5637	Euctr, I.T.	2011	EU Clinical Trials Register. 2011	non pertinent
5638	Euctr, D.E.	2011	EU Clinical Trials Register. 2011	non pertinent
5639	Eric et al.,	2011	Annals of surgery. 2011; 253(2):328-335	non pertinent
5640	Einsiedel et al.,	2011	Zeitschrift fur orthopadie und unfallchirurgie. 2011; 149(3):288-295	non pertinent
5641	Eichbaum et al.,	2011	BMC Cancer. 2011; 11	non pertinent
5642	Drks - Meissner	2011	Deutsches Register Klinischer Studien. 2011	non pertinent
5643	Drks - Kotchoubey	2011	Deutsches Register Klinischer Studien. 2011	non pertinent
5644	Diekhof et al.,	2011	Neuroimage. 2011; 54(2):1703-1714	non pertinent
5645	Dellapina et al.,	2011	Mov Disord. 2011; 26(1):153-157	non pertinent
5646	De Wijkerslooth et al.,	2011	Gastroenterology. 2011; 140(5):409-	non pertinent
5647	Daz-Insa et al.,	2011	International j of neuroscience. 2011; 121(12):655-661	non pertinent
5648	Davis et al.,	2011	J of clinical sleep medicine. 2011; 7(6):622-631	non pertinent
5649	Dastidar	2011	J of thoracic oncology. 2011; 6(6):832-833	non pertinent
5650	Dasilva et al.,	2011	Cephalalgia. 2011; 31:41	non pertinent
5651	Damush et al.,	2011	Circulation: cardiovascular quality and outcomes. 2011; 4(6)	non pertinent
5652	Cox et al.,	2011	Vaccine. 2011; 29(45):8049-8059	non pertinent
5653	Cortot et al.,	2011		non pertinent
5654	Cong et al.,	2011	Biol Res Nurs. 2011; 13(2):204-216	non pertinent

5655	Comstock et al.,	2011	Clinical ophthalmology (auckland, N.Z.). 2011; 5(1):177-186	non pertinent
5656	Coen et al.,	2011	Gastroenterology. 2011; 141(3):909-917.e901	non pertinent
5657	Christidis et al.,	2011	Rheumatology. 2011; 50:iii32-iii33	non pertinent
5658	Christensen et al.,	2011	J of rheumatology. 2011; 38(8):1563-1568	non pertinent
5659	Chrispijn et al.,	2011	Trials. 2011; 12:246	non pertinent
5660	Choi et al.,	2011	Korean j of biological psychiatry. 2011; 18(3):148-158	non pertinent
5661	Chiaretti et al.,	2011	Arch Dis Child. 2011; 96(2):160-163	non pertinent
5662	Chi, Ctr Trc - Wang	2011	Chinese Clinical Trial Registry. 2011	non pertinent
5663	Chevet et al.,	2011	Annales francaises d'anesthesie ET de reanimation. 2011; 30(1):17-24	non pertinent
5664	Cahn et al.,	2011	PLoS One. 2011; 6(8)	non pertinent
5665	Cady et al.,	2011	Headache. 2011; 51(7):1078-1086	non pertinent
5666	Buller	2011	J of thrombosis and haemostasis : JTH. 2011; 9(1):92-99	non pertinent
5667	Budde et al.,	2011	Transplantation. 2011; 92(3):321-327	non pertinent
5668	Brovold et al.,	2011	European geriatric medicine. 2011; 2:101-102	non pertinent
5669	Boy et al.,	2011	PLoS One. 2011; 6(8)	non pertinent
5670	Borckardt et al.,	2011	Clinical J of Pain. 2011; 27(6):486-494	non pertinent
5671	Bokov et al.,	2011	Pain Physician. 2011; 14(6):545-557	non pertinent
5672	Bókkon et al.,	2011	Electromagnetic biology and medicine. 2011; 30(3):115-127	non pertinent
5673	Bohmer et al.,	2011	International j of clinical pharmacology and therapeutics. 2011; 49(7):451-460	non pertinent

5674	Boellaard et al.,	2011	BMC Gastroenterol. 2011; 11:128	non pertinent
5675	Bø et al.,	2011	Neurology and urodynamics. 2011; 30(6):1104-1105	non pertinent
5676	Bernardini et al.,	2011	Minerva pediatrics. 2011; 63(4):247-255	non pertinent
5677	Bennell et al.,	2011	Bmj. 2011; 342:d2912	non pertinent
5678	Bellapianta et al.,	2011	Orthopedics. 2011; 34(11):e708-e712	non pertinent
5679	Beerekamp et al.,	2011	BMC Musculoskelet Disord. 2011; 12:151	non pertinent
5680	Baxter et al.,	2011	Vaccine. 2011; 29(12):2272-2278	non pertinent
5681	Bates et al.,	2011	Pharmacotherapy. 2011; 31(11):1092-1110	non pertinent
5682	Bar et al.,	2011	J of clinical psychopharmacology. 2011; 31(1):103-107	non pertinent
5683	Aziz et al.,	2011	International j of clinical pharmacy. 2011; 33(2):150-154	non pertinent
5684	Aydeniz et al.,	2011	Turkiye fiziksel tip ve rehabilitasyon dergisi. 2011; 57:254	non pertinent
5685	Atack et al.,	2011	J Psychopharmacol. 2011; 25(3):329-344	non pertinent
5686	Arts et al.,	2011	Eur Spine J. 2011; 20(1):51-57	non pertinent
5687	Aran et al.,	2011	Clinical and experimental rheumatology. 2011; 29(3):513-518	non pertinent
5688	Anderson et al.,	2011	American j of obstetrics and gynecology. 2011; 204(3):267.e261-267.e263	non pertinent
5689	Aminabadi et al.,	2011	J Clin Pediatr Dent. 2011; 36(2):127-132	non pertinent
5690	Alvarez et al.,	2011	Annals of hepatology. 2011; 10(4):458-468	non pertinent
5691	Alphs et al.,	2011	Annals of general psychiatry. 2011; 10	non pertinent
5692	Ali et al.,	2011	Cancers. 2011; 3(2):1593-1604	non pertinent

5693	Alegre et al.,	2011	Rheumatology. 2011; 50:iii87	non pertinent
5694	Alberto et al.,	2011	Archives of orthopaedic and trauma surgery. 2011; 131(12):1677-1685	non pertinent
5695	Aikins et al.,	2011	Psychiatry Res. 2011; 188(3):453-455	non pertinent
5696	Ahn et al.,	2011	J Acupunct Meridian Stud. 2011; 4(1):29-43	non pertinent
5697	Adenauer et al.,	2011	BMC Neurosci. 2011; 12:127	non pertinent
5698	Actrn - ?	2011	Australian New Zealand Clinical Trials Registry. 2011	non pertinent
5699	Actrn - ?	2011	Australian New Zealand Clinical Trials Registry. 2011	non pertinent
5700	Actrn - ?	2011	Australian New Zealand Clinical Trials Registry. 2011	non pertinent
5701	Lawrence et al.,	2011	Clinical orthopaedics and related research. 2011; 469(12):3522-3526	non pertinent
5702	Zyloney et al.,	2010	Mol Pain. 2010; 6:80	non pertinent
5703	Zink et al.,	2010	J Neurosci. 2010; 30(20):7017-7022	non pertinent
5704	Zeng et al.,	2010	J of clinical rehabilitative tissue engineering research. 2010; 14(33):6117-6120	non pertinent
5705	Yin et al.,	2010	British j of dermatology. 2010; 163(5):1064-1071	non pertinent
5706	Yang et al.,	2010	European j of integrative medicine. 2010; 2(4):190	non pertinent
5707	Xiong et al.,	2010	Nan fang yi ke da xue xue bao (J of Southern Medical University). 2010; 30(11):2558-2561	non pertinent
5708	Wigal et al.,	2010	Postgraduate medicine. 2011; 122(5):27-34	non pertinent
5709	Wiech et al.,	2010	J Neurosci. 2010; 30(48):16324-16331	non pertinent

5710	Wesnes et al.,	2010	J of psychopharmacology (Oxford, England). 2010; 24(11):1659-1669	non pertinent
5711	Wallace et al.,	2010	Regional anesthesia and pain medicine. 2010; 35(6):507-513	non pertinent
5712	Van Peer et al.,	2010	Psychoneuroendocrinology. 2010; 35(1):21-32	non pertinent
5713	Upadhyay et al.,	2010	Brain. 2010; 133(Pt 7):2098-2114	non pertinent
5714	Tullo et al.,	2010	Neurological sciences. 2010; 31(SUPPL 1):51-54	non pertinent
5715	Tomas et al.,	2010	Hip international. 2010; 20(3):314-319	non pertinent
5716	Toll et al.,	2010	Drug Alcohol Depend. 2010; 111(3):200-206	non pertinent
5717	Theiss et al.,	2010	International j of cardiology. 2010; 145(2):282-284	non pertinent
5718	Tan et al.,	2010	J Burn Care Res. 2010; 31(4):590-597	non pertinent
5719	Tan et al.,	2010	Clinical and experimental dermatology. 2010; 35(4):e109-e112	non pertinent
5720	Tabbert et al.,	2010	Neurobiol Learn Mem. 2010; 94(3):392-401	non pertinent
5721	Sykora	2010	Farmaceuticky obzor. 2010; 79(11-12):330-337	non pertinent
5722	Svensson et al.,	2010	Acta Radiol. 2010; 51(7):722-726	non pertinent
5723	Suris et al.,	2010	Annals of clinical psychiatry. 2010; 22(4):274-279	non pertinent
5724	Strigo et al.,	2010	PLoS One. 2010; 5(11):e15093	non pertinent
5725	Steinmiller et al.,	2010	Exp Clin Psychopharmacol. 2010; 18(3):277-283	non pertinent
5726	Spitzer et al.,	2010	Physician and sportsmedicine. 2010; 38(2):35-47	non pertinent
5727	Solanki et al.,	2010	European j of anaesthesiology. 2010; 27(8):708-713	non pertinent

5728	Slomovitz et al.,	2010	Cancer. 2010; 116(23):5415-5419	non pertinent
5729	Slater et al.,	2010	Lancet. 2010; 376(9748):1225-1232	non pertinent
5730	Sjogaard et al.,	2010	Eur J Appl Physiol. 2010; 108(4):657-669	non pertinent
5731	Sitges et al.,	2010	International J of Psychophysiology. 2010; 77(2):176-183	non pertinent
5732	Simon et al.,	2010	CNS spectrums. 2010; 15(7):436-443	non pertinent
5733	Sheftell et al.,	2010	Cephalalgia. 2010; 30(7):838-846	non pertinent
5734	Shaw et al.,	2010	Health technology assessment (Winchester, England). 2010; 14(26):1-113, iii-iv	non pertinent
5735	Scrivani et al.,	2010	Pain medicine (Malden, Mass.). 2010; 11(6):920-941	non pertinent
5736	Schulz et al.,	2010	J of child and adolescent psychopharmacology. 2010; 20(5):377-385	non pertinent
5737	Schoffski et al.,	2010	Eur J Cancer. 2010; 46(12):2206-2215	non pertinent
5738	Schoell et al.,	2010	PLoS One. 2010; 5(8):e12344	non pertinent
5739	Saracco et al.,	2010	Neurological sciences. 2010; 31 Suppl 1:179-180	non pertinent
5740	Sandsjo et al.,	2010	J Telemed Telecare. 2010; 16(6):329-335	non pertinent
5741	Salman et al.,	2010	Antimicrobial agents and chemotherapy. 2010; 54(1):360-366	non pertinent
5742	Sai et al.,	2010	J of gastroenterology. 2010; 45(3):335-341	non pertinent
5743	Ryoo et al.,	2010	Clinical psychopharmacology and neuroscience. 2010; 8(1):30-37	non pertinent
5744	Rutkowski et al.,	2010	J Oral Implantol. 2010; 36(1):11-23	non pertinent
5745	Rosenberg et al.,	2010	Expert opinion on drug safety. 2010; 9(4):573-592	non pertinent

5746	Rogers et al.,	2010	Diabetologia. 2010; 53:236-	non pertinent
5747	Roessing et al.,	2010	Cartilage. Conference: 9th world congress of the international cartilage repair society. Spain 1(2 Supplement 1):55	non pertinent
5748	Rodriguez-Raecke et al.,	2010	J Neurosci. 2010; 30(34):11363-11368	non pertinent
5749	Riegel et al.,	2010	Am Heart J. 2010; 159(3):392-398	non pertinent
5750	Rawlings et al.,	2010	Psychopharmacology (Berl). 2010; 212(4):625-634	non pertinent
5751	Rasetti et al.,	2010	Neuropsychopharmacology. 2010; 35(10):2101-2109	non pertinent
5752	Rao et al.,	2010	Gastroenterology. 2010; 139(5):1549-1558, 1558.e1541	non pertinent
5753	Rakesh et al.,	2010	Pharmacologyonline. 2010; 1:458-468	non pertinent
5754	Pruegsanusak et al.,	2010	J Med Assoc Thai. 2010; 93(5):558-565	non pertinent
5755	Pouga et al.,	2010	Hum Brain Mapp. 2010; 31(10):1469-1481	non pertinent
5756	Portenoy et al.,	2010	Pain. 2010; 151(3):617-624	non pertinent
5757	Pogatzki-Zahn et al.,	2010	Anesthesiology. 2010; 112(2):406-417	non pertinent
5758	Pettilä et al.,	2010	Clinical care (london, england). 2010; 14(4):R139	non pertinent
5759	Perkins et al.,	2010	Biology of blood and marrow transplantation. 2010; 16(7):937-947	non pertinent
5760	Patella et al.,	2010	Cartilage. 2010; 1(2):147-	non pertinent
5761	Orlando et al.,	2010	Clinical and experimental gastroenterology. 2010; 3(1):117-125	non pertinent
5762	Ogawa et al.,	2010	Japanese j of anesthesiology. 2010; 59(8):961-970	non pertinent
5763	Ntr - Verkalk	2010	Netherlands Trial Register. 2010	non pertinent
5764	Ntr – Dahan	2010	Netherlands Trial Register. 2010	non pertinent
5765	Nir et al.,	2010	Brain Res. 2010; 1344:77-86	non pertinent
5766	Nikzad et al.,	2010	J Oral Maxillofac Surg. 2010; 68(6):1353-1359	non pertinent

5767	Nicolini et al.,	2010	Clinical lymphoma, myeloma & leukemia. 2010; 10(5):394-399	non pertinent
5768	Ni et al.,	2010	Chang gung medical j. 2010; 33(4):443-452	non pertinent
5769	Ng et al.,	2010	Asia-pacific psychiatry. 2010; 2(3):A21-A22	non pertinent
5770	Ng et al.,	2010	Acta radiologica (stockholm, sweden : 1987). 2010; 51(4):368-374	non pertinent
5771	Nct – Pape	2010	National Library of Medicine. 2010	non pertinent
5772	Nct - David	2010	National Library of Medicine. 2010	non pertinent
5773	Nct - Sharar	2010	National Library of Medicine. 2010	non pertinent
5774	Nct – Peretti	2010	National Library of Medicine. 2010	non pertinent
5775	Nct – Meredith	2010	National Library of Medicine. 2010	non pertinent
5776	Nct – Datta	2010	National Library of Medicine. 2010	non pertinent
5777	Nct – Hargrove	2010	National Library of Medicine. 2010	non pertinent
5778	Nct – Brunet	2010	Am J Psychiatry. 2018 May; 175(5):427-433	non pertinent
5779	Nct – Olthuis	2010	National Library of Medicine. 2010	non pertinent
5780	Nct – Weck	2010	National Library of Medicine. 2010	non pertinent
5781	Nct – Brill	2010	National Library of Medicine. 2010	non pertinent
5782	Nct – Davidson	2010	National Library of Medicine. 2010	non pertinent
5783	Mustafah Khwaja et al.,	2010	Canadian j of emergency medicine. 2010; 12(1):39-44	non pertinent
5784	Muller et al.,	2010	Arch Orthop Trauma Surg. 2010; 130(7):927-935	non pertinent
5785	Morin et al.,	2010	Regional anesthesia and pain medicine. 2010; 35(2):194-199	non pertinent
5786	Miskowiak et al.,	2010	Psychopharmacology. 2010; 210(3):419-428	non pertinent
5787	Mhuircheartaigh et al.,	2010	J Neurosci. 2010; 30(27):9095-9102	non pertinent
5788	Merz et al.,	2010	Psychoneuroendocrinology. 2010; 35(1):33-46	non pertinent
5789	Mehta et al.,	2010	J of perinatology. 2010; 30(11):731-735	non pertinent

5790	Martinez-Sandoval et al.,	2010	J of travel medicine. 2010; 17(2):111-117	non pertinent
5791	Marmotti et al.,	2010	Haematologica. 2010; 95:121	non pertinent
5792	Marini et al.,	2010	Clinical J of Pain. 2010; 26(7):611-616	non pertinent
5793	Man et al.,	2010	Hong kong j of emergency medicine. 2010; 17(2):126-131	non pertinent
5794	Lyubkin et al.,	2010	Depress Anxiety. 2010; 27(11):1011-1016	non pertinent
5795	Lundström et al.,	2010	International J of Psychophysiology. 2010; 78(2):179-189	non pertinent
5796	Lundborg et al.,	2010	Diabetic medicine. 2010; 27(7):823-829	non pertinent
5797	Lu et al.,	2010	Pain. 2010; 148(1):75-83	non pertinent
5798	Lu et al.,	2010	J Formos Med Assoc. 2010; 109(8):589-595	non pertinent
5799	Litt et al.,	2010	Pain. 2010; 151(1):110-116	non pertinent
5800	Lindeboom et al.,	2010	Clin Oral Implants Res. 2010; 21(4):366-370	non pertinent
5801	Leslie et al.,	2010	Anesth Analg. 2010; 110(3):823-828	non pertinent
5802	Leppert et al.,	2010	International j of clinical practice. 2010; 64(12):1681-1687	non pertinent
5803	Lemmens et al.,	2010	BMC anesthesiology. 2010; 10:15 TN:NCT00473694	non pertinent
5804	Leistritz et al.,	2010	Methods Inf Med. 2010; 49(5):484-491	non pertinent
5805	Lee et al.,	2010	Korean j of anesthesiology. 2010; 58(6):537-541	non pertinent
5806	Lauta et al.,	2010	J Neurosurg Anesthesiol. 2010; 22(2):110-118	non pertinent
5807	Laudon et al.,	2010	Mayo clinic proceedings. 2010; 85(4):314-322	non pertinent
5808	Laretzaki et al.,	2010	J Psychopharmacol. 2010; 24(5):667-675	non pertinent
5809	Labuschagne et al.,	2010	Neuropsychopharmacology. 2010; 35(12):2403-2413	non pertinent

5810	Kojuri et al.,	2010	Cardiovasc Ther. 2010; 28(3):147-152	non pertinent
5811	Knittle et al.,	2010	Arthritis care & research. 2010; 62(10):1460-1472	non pertinent
5812	Keiser et al.,	2010	Vaccine. 2010; 28(43):6970-6976	non pertinent
5813	Kayiran et al.,	2010	Appl Psychophysiol Biofeedback. 2010; 35(4):293-302	non pertinent
5814	Jprn, Umin - ?	2010	Japan Primary Registries Network. 2010	non pertinent
5815	Jprn, Umin - ?	2010	Japan Primary Registries Network. 2010	non pertinent
5816	Jensen et al.,	2010	European j of integrative medicine. 2010; 2(4):195-196	non pertinent
5817	Jensch et al.,	2010	Eur Radiol. 2010; 20(1):146-156	non pertinent
5818	Jain et al.,	2010	Paediatr Anaesth. 2010; 20(4):330-337	non pertinent
5819	Isrctn - Marsh	2010	ISRCTN Registry. 2010	non pertinent
5820	Isrctn - Friesem	2010	ISRCTN Registry. 2010	non pertinent
5821	Isrctn – Hawley	2010	ISRCTN Registry. 2010	non pertinent
5822	Isrctn – Aram	2010	ISRCTN Registry. 2010	non pertinent
5823	Isrctn - Renton	2010	ISRCTN Registry. 2010	non pertinent
5824	Irct138807192573N – Moradveisi	2010	Iranian Registry Clinical Trials. 2010	non pertinent
5825	Irct138711151637N - Kahrizi	2010	Iranian Registry Clinical Trials. 2010	non pertinent
5826	Illman et al.,	2010	J Clin Monit Comput. 2010; 24(5):371-376	non pertinent
5827	Hurley et al.,	2010	BMC musculoskeletal disorders. 2010; 11:70	non pertinent
5828	Hurlemann et al.,	2010	Psychol Med. 2010; 40(11):1839-1848	non pertinent
5829	Ho et al.,	2010	Br J Anaesth. 2010; 105(3):371-376	non pertinent
5830	Henckens et al.,	2010	J of neuroscience. 2010; 30(38):12725-12732	non pertinent
5831	Heldestad et al.,	2010	Clin Neurophysiol. 2010; 121(11):1878-1885	non pertinent

5832	Hegarty et al.,	2010	BMC Public Health. 2010; 10:2	non pertinent
5833	Hartmann et al.,	2010	Alimentary pharmacology & therapeutics. 2010; 32(3):368-376	non pertinent
5834	Gustin et al.,	2010	Pain. 2010; 151(1):69-76	non pertinent
5835	Goren et al.,	2010	Clinical rehabilitation. 2010; 24(7):623-631	non pertinent
5836	Gobbi	2010	Cartilage. Conference: 9th world congress of the international cartilage repair society. Spain 1(2 Supplement 1):23-26	non pertinent
5837	Gierthmuhlen et al.,	2010	Mov Disord. 2010; 25(9):1195-1202	non pertinent
5838	Garcia-Leal et al.,	2010	J Psychopharmacol. 2010; 24(5):683-694	non pertinent
5839	Fusar-Poli et al.,	2010	The international j of neuropsychopharmacology. 2010; 13(4):421-432	non pertinent
5840	Frohman et al.,	2010	Therapeutic advances in neurological disorders. 2010; 3(1):15-28	non pertinent
5841	Freund et al.,	2010	Clin J Pain. 2010; 26(4):339-347	non pertinent
5842	Foster et al.,	2010	BMC Musculoskelet Disord. 2010; 11:186	non pertinent
5843	Fontaine et al.,	2010	J of headache and pain. 2010; 11(1):23-31	non pertinent
5844	Eun et al.,	2010	Laryngoscope. 2010; 120(11):2189-2192	non pertinent
5845	Euctr, S.E.	2010	EU Clinical Trials Register. 2010	non pertinent
5846	Euctr, L.T.	2010	EU Clinical Trials Register. 2010	non pertinent
5847	Euctr, G.B.	2010	EU Clinical Trials Register. 2010	non pertinent
5848	Euctr, F.R.	2010	EU Clinical Trials Register. 2010	non pertinent
5849	Erhart et al.,	2010	J Orthop Res. 2010; 28(7):873-879	non pertinent
5850	Ellis et al.,	2010	PLoS One. 2010; 5(1):1-9	non pertinent

5851	Ellerkmann et al.,	2010	Anaesth Intensive Care. 2010; 38(1):159-166	non pertinent
5852	Elhawary et al.,	2010	Spine j. conference: 25th Annual Meeting of the North American Spine Society. NASS Miami, FL United States. Conference Start: 20101005. Conference End: 20101009. Conference Publication:(10:(9 SUPPL 1)):130	non pertinent
5853	Ebert et al.,	2010	Cartilage. 2010; 1(2):51-	non pertinent
5854	Dulko et al.,	2010	J Am Acad Nurse Pract. 2010; 22(1):45-55	non pertinent
5855	Drescher et al.,	2010	Headache. 2010; 50(8):1328-1334	non pertinent
5856	Distler et al.,	2010	Arthritis Rheum. 2010; 62(1):291-300	non pertinent
5857	Dewall et al.,	2010	Psychological science. 2010; 21(7):931-937	non pertinent
5858	Denegar et al.,	2010	Clin Interv Aging. 2010; 5:199-206	non pertinent
5859	Demirtas-Tatlidede et al.,	2010	Schizophr Res. 2010; 124(1-3):91-100	non pertinent
5860	Delmastro et al.,	2010	Neurourology and urodynamics. 2010; 29:62-63	non pertinent
5861	De Boer et al.,	2010	Osteoarthritis and cartilage. 2010; 18:145-	non pertinent
5862	Daya et al.,	2010	J Sleep Res. 2010; 19(2):317-322	non pertinent
5863	Davies et al.,	2010	Current drug therapy. 2010; 5(2):118-121	non pertinent
5864	Daly et al.,	2010	Psychopharmacology. 2010; 210(4):499-510	non pertinent
5865	Cutshall et al.,	2010	Complement Ther Clin Pract. 2010; 16(2):92-95	non pertinent
5866	Coslett et al.,	2010	J Int Neuropsychol Soc. 2010; 16(4):603-612	non pertinent
5867	Cosgrove et al.,	2010	Nicotine Tob Res. 2010; 12(5):535-539	non pertinent
5868	Cockayne	2010	Trials. 2010; 11:12	non pertinent

5869	Chua	2010	Annals of the academy of medicine singapore. 2010; 39(11 SUPPL 1):338	non pertinent
5870	Chu et al.,	2010	Chinese j of integrative medicine. 2010; 16(5):399-405	non pertinent
5871	Cho et al.,	2010	Acta Anaesthesiol Scand. 2010; 54(4):453-457	non pertinent
5872	Chiarioni et al.,	2010	Digestive and liver disease. 2010; 42:119-	non pertinent
5873	Chen et al.,	2010	Pain management nursing. 2010; 11(3):159-168	non pertinent
5874	Chen et al.,	2010	International j of clinical practice. 2010; 64(11):1530-1534	non pertinent
5875	Celik et al.,	2010	J Spinal Disord Tech. 2010; 23(4):229-235	non pertinent
5876	Carter et al.,	2010	CNS Drugs. 2010; 24(4):337-361	non pertinent
5877	Carmona et al.,	2010	Vaccine. 2010; 28(36):5837-5844	non pertinent
5878	Cahill et al.,	2010	Current opinion in obstetrics & gynecology. 2010; 22(2):122-127	non pertinent
5879	Büttner et al.,	2010	Der Anaesthesist. 2010; 59(2):126-134	non pertinent
5880	Briggs et al.,	2010	Pain Physician. 2010; 13(6):E347-E355	non pertinent
5881	Brander et al.,	2010	Pm r. 2010; 2(4):259-267	non pertinent
5882	Boudreau et al.,	2010	J of oral rehabilitation. 2010; 37(9):704-718	non pertinent
5883	Borhani Haghighi et al.,	2010	International j of clinical practice. 2010; 64(4):451-456	non pertinent
5884	Bollens et al.,	2010	Annals of physical and rehabilitation medicine. 2010; 53:e177-	non pertinent
5885	Bhattacharyya et al.,	2010	Neuropsychopharmacology. 2010; 35(3):764-774	non pertinent
5886	Belavy et al.,	2010	J Appl Physiol (1985). 2010; 109(6):1801-1811	non pertinent
5887	Baygin et al.,	2010	Eur J Anaesthesiol. 2010; 27(4):341-346	non pertinent

5888	Aronson et al.,	2010	Plos neglected tropical diseases. 2010; 4(3):e628	non pertinent
5889	Apfel et al.,	2010	BMC musculoskeletal disorders. 2010; 11:155	non pertinent
5890	Aoki et al.,	2010	Internal medicine (Tokyo, Japan). 2010; 49(12):1085-1087	non pertinent
5891	Anderson et al.,	2010	Clinical nurse specialist CNS. 2010; 24(3):140-148	non pertinent
5892	Andersen et al.,	2010	Eur Spine J. 2010; 19(12):2200-2208	non pertinent
5893	Aide et al.,	2010	PLoS One. 2010; 5(11):e13838	non pertinent
5894	Adolph et al.,	2010	Neuroimage. 2010; 49(1):720-730	non pertinent
5895	Aderjan et al.,	2010	Pain. 2010; 151(1):97-103	non pertinent
5896	Actrn – Zacharin	2010	Australian New Zealand Clinical Trials Registry. 2010	non pertinent
5897	Actrn - Clanchy	2010	Australian New Zealand Clinical Trials Registry. 2010	non pertinent
5898	Simon et al.,	2010	CNS spectrums. 2010; 15(7):436-443	non pertinent
5899	J of neurology	2010	J of neurology. 2010; 257	non pertinent
5900	Zeni et al.,	2009	J of child and adolescent psychopharmacology. 2009; 19(5):553-561	non pertinent
5901	Yu et al.,	2009	Int J Nurs Stud. 2009; 46(11):1423-1430	non pertinent
5902	York et al.,	2009	BMC complementary and alternative medicine. 2009; 9:10	non pertinent
5903	Yen et al.,	2009	J Clin Neurosci. 2009; 16(2):214-219	non pertinent
5904	Xu et al.,	2009	Anesth Analg. 2009; 108(2):478-483	non pertinent
5905	Wu et al.,	2009	Zhonghua yi xue za zhi. 2009; 89(5):314-317	non pertinent
5906	Wosko et al.,	2009	Anestezjologia intensywna terapia. 2009; 41(1):22-27	non pertinent

5907	Watson et al.,	2009	Int J Neuropsychopharmacol. 2009; 12(3):357-370	non pertinent
5908	Wang et al.,	2009	Soc Cogn Affect Neurosci. 2009; 4(4):357-368	non pertinent
5909	Wang et al.,	2009	Acute pain. 2009; 11(2):43-50	non pertinent
5910	Wang et al.,	2009	Anesthesiology. 2009; 111(4):871-880	non pertinent
5911	Wang et al.,	2009	Biological psychiatry. 2009; 66(5):516-521	non pertinent
5912	Walker et al.,	2009	Pain. 2009; 141(1-2):79-87	non pertinent
5913	Walch et al.,	2009	Orthop Traumatol Surg Res. 2009; 95(7):463-470	non pertinent
5914	Von Delius et al.,	2009	Am J Gastroenterol. 2009; 104(2):318-325	non pertinent
5915	Vidailhet et al.,	2009	Lancet Neurol. 2009; 8(8):709-717	non pertinent
5916	Veldhuijzen et al.,	2009	Pain. 2009; 141(1-2):104-113	non pertinent
5917	Van Wingen et al.,	2009	Neuropsychopharmacology. 2009; 34(3):539-547	non pertinent
5918	Vaid et al.,	2009	Archives of gynecology and obstetrics. 2009; 280(6):893-897	non pertinent
5919	Tuveson et al.,	2009	Pain. 2009; 143(1-2):84-91	non pertinent
5920	Tripathi et al.,	2009	Annals of cardiac anaesthesia. 2009; 12(1):34-39	non pertinent
5921	Tan et al.,	2009	Anaesth Intensive Care. 2009; 37(5):807-814	non pertinent
5922	Takahashi et al.,	2009	J of child and adolescent psychopharmacology. 2009; 19(4):341-350	non pertinent
5923	Svanborg et al.,	2009	European child & adolescent psychiatry. 2009; 18(12):725-735	non pertinent
5924	Suzuki et al.,	2009	Japanese j of chemotherapy. 2009; 57(SUPPL 1):167-185	non pertinent
5925	Su et al.,	2009	BJOG. 2009; 116(11):1461-1466	non pertinent
5926	Smeets et al.,	2009	Eur J Pain. 2009; 13(1):71-81	non pertinent
5927	Sissoko et al.,	2009	PLoS One. 2009; 4(10):e6732	non pertinent
5928	Silberstein et al.,	2009	Headache. 2009; 49(8):1153-1162	non pertinent

5929	Sikand et al.,	2009	Pain. 2009; 144(1-2):66-75	non pertinent
5930	Shen et al.,	2009	Medical acupuncture. 2009; 21(4):263-268	non pertinent
5931	Schumacher et al.,	2009	Anesthesiology. 2009; 111(4):790-804	non pertinent
5932	Scheller et al.,	2009	Anesthesiology. 2009; 111(2):340-355	non pertinent
5933	Schanz et al.,	2009	JDDG – j of the german society of dermatology. 2009; 7(12):1055-1059	non pertinent
5934	Saps et al.,	2009	J of pediatric gastroenterology and nutrition. 2009; 48(SUPPL 2):101-103	non pertinent
5935	Santa Ana et al.,	2009	Drug Alcohol Depend. 2009; 104(3):220-227	non pertinent
5936	Samangaya et al.,	2009	Hypertension in pregnancy. 2009; 28(4):369-382	non pertinent
5937	Sallee et al.,	2009	J of the American Academy of Child and Adolescent Psychiatry. 2009; 48(2):155-165	non pertinent
5938	Sagara et al.,	2009	Malaria j. 2009; 8:63	non pertinent
5939	Roy et al.,	2009	Proc Natl Acad Sci U S A. 2009; 106(49):20900-20905	non pertinent
5940	Roth et al.,	2009	Primary care companion to the j of clinical psychiatry. 2009; 11(6):292-301	non pertinent
5941	Raynauld et al.,	2009	Ann Rheum Dis. 2009; 68(6):938-947	non pertinent
5942	Raynauld et al.,	2009	Osteoarthritis and cartilage. 2009; 17:176-	non pertinent
5943	Radpay et al.,	2009	Tanaffos. 2009; 8(3):51-57	non pertinent
5944	Raddi et al.,	2009	Archives of medical science. 2009; 5(1):57-62	non pertinent
5945	Puttini et al.,	2009	European j of pain (London, England). 2009; 13:5-6	non pertinent
5946	Poreisz et al.,	2009	BMC Neurosci. 2009; 10:54	non pertinent

5947	Philip et al.,	2009	J of allergy and clinical immunology. 2009; 124(5):942-948.e941-949	non pertinent
5948	Petrucci et al.,	2009	J of cardiovascular electrophysiology. 2009; 20(3):299-306	non pertinent
5949	Perkins et al.,	2009	J Neurosci. 2009; 29(40):12617-12624	non pertinent
5950	Perez-Palomares et al.,	2009	BMC musculoskeletal disorders. 2009; 10:92	non pertinent
5951	Park et al.,	2009	J of endourology / Endourological Society. 2009; 23(11):1913-1917	non pertinent
5952	Padmanabhan et al.,	2009	Anesth Analg. 2009; 109(5):1448-1455	non pertinent
5953	Ortega et al.,	2009	Exercise immunology review. 2009; 15:42-65	non pertinent
5954	Onur et al.,	2009	Soc Cogn Affect Neurosci. 2009; 4(2):119-126	non pertinent
5955	Ohtori et al.,	2009	J of Pain. 2009; 10(8):870-875	non pertinent
5956	Ogutu et al.,	2009	PLoS One. 2009; 4(3):e4708	non pertinent
5957	Nutt et al.,	2009	J of psychopharmacology (Oxford, England). 2009; 23(8):867-873	non pertinent
5958	Ndiaye et al.,	2009	Malaria j. 2009; 8:125	non pertinent
5959	Nct – Abrahão	2009	National Library of Medicine. 2009	non pertinent
5960	Nct – MFox	2009	National Library of Medicine. 2009	non pertinent
5961	Nct – Olivo	2009	National Library of Medicine. 2009	non pertinent
5962	Nct – Chu	2009	National Library of Medicine. 2009	non pertinent
5963	Nct – Froelich	2009	National Library of Medicine. 2009	non pertinent
5964	Nct – Skouen	2009	National Library of Medicine. 2009	non pertinent
5965	Nct – Borckardt et al.,	2009	Pain. 2011 Jan; 152(1):182-7	non pertinent
5966	Nct – Silva	2009	National Library of Medicine. 2009	non pertinent
5967	Nct – GSK Clinical Trials	2009	National Library of Medicine. 2009	non pertinent
5968	Nct – Curatolo	2009	National Library of Medicine. 2009	non pertinent
5969	Nct – Goldman	2009	National Library of Medicine. 2009	non pertinent

5970	Nct – Ang	2009	National Library of Medicine. 2009	non pertinent
5971	Nct – Vichinsky	2009	National Library of Medicine. 2009	non pertinent
5972	Nct – National Institute on Drug Abuse (NIDA)	2009	National Library of Medicine. 2009	non pertinent
5973	Nahmias et al.,	2009	Pain. 2009; 147(1-3):224-232	non pertinent
5974	Murphy et al.,	2009	British j of psychiatry. 2009; 194(6):535-540	non pertinent
5975	Mullally et al.,	2009	Pain Physician. 2009; 12(6):1005-1011	non pertinent
5976	Mulherin et al.,	2009	Foot (edinburgh, scotland). 2009; 19(2):98-100	non pertinent
5977	Moulson et al.,	2009	Developmental psychology. 2009; 45(1):17-30	non pertinent
5978	Montoya et al.,	2009	Current medical research and opinion. 2009; 25(11):2745-2754	non pertinent
5979	Miskowiak et al.,	2009	European neuropsychopharmacology. 2009; 19:189-190	non pertinent
5980	Miner et al.,	2009	Acad Emerg Med. 2009; 16(9):825-834	non pertinent
5981	Millàn-Guerrero et al.,	2009	European j of neurology. 2009; 16(1):88-94	non pertinent
5982	Mieres et al.,	2009	J of nuclear cardiology. 2009; 16(1):105-112	non pertinent
5983	Meunier et al.,	2009	Osteoporosis international. 2009; 20(10):1663-1673	non pertinent
5984	Messier et al.,	2009	BMC Musculoskelet Disord. 2009; 10:93	non pertinent
5985	Mérelle et al.,	2009	Cephalalgia. 2009; 29(6):606-615	non pertinent
5986	Mercadante et al.,	2009	Current medical research and opinion. 2009; 25(11):2805-2815	non pertinent
5987	Meininger et al.,	2009	Amyotrophic lateral sclerosis. 2009; 10(5-6):378-383	non pertinent
5988	McDonald et al.,	2009	J Pain Symptom Manage. 2009; 37(6):1050-1060	non pertinent
5989	McDonald	2009	Pain Manag Nurs. 2009; 10(3):142-148	non pertinent
5990	McCluney et al.,	2009	J of laryngology and otology. 2009; 123(6):626-630	non pertinent

5991	Masheb et al.,	2009	Pain. 2009; 141(1-2):31-40	non pertinent
5992	Marcus et al.,	2009	J of the American Academy of Child and Adolescent Psychiatry. 2009; 48(11):1110-1119	non pertinent
5993	Mankowski et al.,	2009	Obstetrics and gynecology. 2009; 113(5):1052-1057	non pertinent
5994	Manaktala et al.,	2009	Indian j of dermatology. 2009; 54(4):350-356	non pertinent
5995	Magni et al.,	2009	Anesth Analg. 2009; 109(2):567-571	non pertinent
5996	Luckett et al.,	2009	Psycho-oncology. 2009; 18(11):1129-1138	non pertinent
5997	Lohle et al.,	2009	Cardiovascular and interventional radiology. 2009; 32:268-	non pertinent
5998	Litt et al.,	2009	Pain. 2009; 145(1-2):160-168	non pertinent
5999	Leykin et al.,	2009	Arthroscopy. 2009; 25(9):1019-1024	non pertinent
6000	Levy et al.,	2009	Cancer. 2009; 115(1):207-216	non pertinent
6001	Leutgeb et al.,	2009	Biological psychology. 2009; 82(3):293-300	non pertinent
6002	Leslie et al.,	2009	Anesthesiology. 2009; 111(3):547-555	non pertinent
6003	Lera et al.,	2009	J of psychosomatic research. 2009; 67(5):433-441	non pertinent
6004	Lee et al.,	2009	J of rehabilitation medicine. 2009; 41(9):740-745	non pertinent
6005	Lee et al.,	2009	Br J Anaesth. 2009; 103(6):861-866	non pertinent
6006	Lazik et al.,	2009	KIM – komplementare und integrative medizin. Arztezeitschrift fur naturheilverfahren. 2009; 50(3):41-46	non pertinent
6007	Lascasas Porto et al.,	2009	International angiology. 2009; 28(3):222-231	non pertinent
6008	Lameris et al.,	2009	BMC (online). 2009; 339(7711):29-33	non pertinent

6009	Lakse et al.,	2009	American j of physical medicine & rehabilitation. 2009; 88(10):805-811	non pertinent
6010	LaForce et al.,	2009	Current medical research and opinion. 2009; 25(10):2353-2359	non pertinent
6011	Krishnamoorthy et al.,	2009	Expert review of cardiovascular therapy. 2009; 7(5):473-481	non pertinent
6012	Konradsen et al.,	2009	Ambulatory surgery. 2009; 15(1)	non pertinent
6013	Kong et al.,	2009	Neuroimage. 2009; 47(3):1066-1076	non pertinent
6014	Kong et al.,	2009	Neuroimage. 2009; 45(3):940-949	non pertinent
6015	Kerssens et al.,	2009	Anesthesiology. 2009; 111(3):518-524	non pertinent
6016	Kanovsky et al.,	2009	Clinical neuropharmacology. 2009; 32(5):259-265	non pertinent
6017	Johal et al.,	2009	J of trauma. 2009; 67(4):875-882	non pertinent
6018	Iwanaga et al.,	2009	Diabetes technology & theraapeutics. 2009; 11(2):81-86	non pertinent
6019	Isrctn - Biffi	2009	ISRCTN Registry. 2009	non pertinent
6020	Isrctn – Ong	2009	ISRCTN Registry. 2009	non pertinent
6021	Isrctn - Österman	2009	ISRCTN Registry. 2009	non pertinent
6022	Ishinova et al.,	2009	Span J Psychol. 2009; 12(2):715-724	non pertinent
6023	Irct38804082093N – Moin	2009	Iranian Registry of Clinical Trials. 2009	non pertinent
6024	Irct138712111719N - Otadi	2009	Iranian Registry of Clinical Trials. 2009	non pertinent
6025	Hurley et al.,	2009	BMC musculoskeletal disorders. 2009; 10:79	non pertinent
6026	Hurlemann et al.,	2009	European neuropsychopharmacology. 2009; 19:307	non pertinent
6027	Hunter et al.,	2009	Pain Med. 2009; 10(4):730-738	non pertinent
6028	Hun et al.,	2009	Postgraduate medicine. 2009; 121(2):119-124	non pertinent
6029	Holguin et al.,	2009	Spine J. 2009; 9(6):470-477	non pertinent

6030	Herrndobler et al.,	2009	Schmerz (berlin, germany). 2009; 23(2):134-144	non pertinent
6031	Hellings et al.,	2009	J of clinical psychopharmacology. 2009; 29(5):492-495	non pertinent
6032	Harris et al.,	2009	Neuroimage. 2009; 47(3):1077-1085	non pertinent
6033	Gwee et al.,	2009	European j of gastroenterology & hepatology. 2009; 21(4):417-424	non pertinent
6034	Gunzler	2009	Expert opinion on pharmacotherapy. 2009; 10(6):1027-1038	non pertinent
6035	Gruenewald et al.,	2009	Br J Anaesth. 2009; 103(4):586-593	non pertinent
6036	Gilron et al.,	2009	Lancet. 2009; 374(9697):1252-1261	non pertinent
6037	Gatchel et al.,	2009	J of occupational rehabilitation. 2009; 19(1):49-55	non pertinent
6038	García-Campayo et al.,	2009	Trials. 2009; 10:24	non pertinent
6039	Gacto et al.,	2009	Burns. 2009; 35(3):343-347	non pertinent
6040	Fusar-Poli et al.,	2009	Arch Gen Psychiatry. 2009; 66(1):95-105	non pertinent
6041	Furuzawa-carballeda et al.,	2009	European j of clinical investigation. 2009; 39(7):598-606	non pertinent
6042	Foley et al.,	2009	International j of dermatology. 2009; 48(11):1236-1245	non pertinent
6043	Fokkema et al.,	2009	Circulation. Cardiovascular interventions. 2009; 2(4):323-329	non pertinent
6044	Faraoni et al.,	2009	Br J Anaesth. 2009; 102(3):336-339	non pertinent
6045	Fang et al.,	2009	Hum Brain Mapp. 2009; 30(4):1196-1206	non pertinent
6046	Euctr, G.B.	2009	EU Clinical Trials Register. 2009	non pertinent
6047	Eser et al.,	2009	J Psychiatr Res. 2009; 43(4):393-400	non pertinent
6048	Eisenberger et al.,	2009	Neuroimage. 2009; 47(3):881-890	non pertinent
6049	Eippert et al.,	2009	Science. 2009; 326(5951):404	non pertinent

6050	Eippert et al.,	2009	Neuron. 2009; 63(4):533-543	non pertinent
6051	Dychter et al.,	2009	Current therapeutic research – clinical and experimental. 2009; 70(6):421-438	non pertinent
6052	Doufas et al.,	2009	Anesth Analg. 2009; 109(4):1097-1104	non pertinent
6053	Di Clemente et al.,	2009	Pain. 2009; 144(1-2):156-161	non pertinent
6054	Dharmapalaiah et al.,	2009	Rheumatology. 2009; 48:i42-i43	non pertinent
6055	Derntl et al.,	2009	Psychoneuroendocrinology. 2009; 34(5):687-693	non pertinent
6056	Deng et al.,	2009	Chinese j of traumatology = zhonghua chuang shang za zhi. 2009; 12(4):223-227	non pertinent
6057	Del-Ben et al.,	2009	European neuropsychopharmacology. 2009; 19:609-610	non pertinent
6058	Del Campo et al.,	2009	Annals of oncology: official j of the european society for medical oncology. 2009; 20(11):1794-1802	non pertinent
6059	Daniels et al.,	2009	Current medical research and opinion. 2009; 25(3):765-776	non pertinent
6060	Cunningham-Bussel et al.,	2009	Psychoneuroendocrinology. 2009; 34(5):694-704	non pertinent
6061	Coyle	2009	Annals of indian academy of neurology. 2009; 12(4):273-282	non pertinent
6062	Corsinovi et al.,	2009	Arch Gerontol Geriatr. 2009; 49(3):378-382	non pertinent
6063	Corinaldesi et al.,	2009	Alimentary pharmacology & therapeutics. 2009; 30(3):245-252	non pertinent
6064	Coppens et al.,	2009	Neuropsychologia. 2009; 47(12):2496-2503	non pertinent
6065	Cong et al.,	2009	Early Hum Dev. 2009; 85(9):561-567	non pertinent
6066	Cohen et al.,	2009	BMJ (Clinical research ed.). 2009; 338:b1088	non pertinent

6067	Coen et al.,	2009	Gastroenterology. 2009	non pertinent
6068	Coakley et al.,	2009	Complement Ther Clin Pract. 2009; 15(3):141-146	non pertinent
6069	Chu et al.,	2009	Zhong xi yi jie he xue bao (j of chinese integrative medicine). 2009; 7(8):729-735	non pertinent
6070	Childress et al.,	2009	J of child and adolescent psychopharmacology. 2009; 19(4):351-361	non pertinent
6071	Chi, Ctr Trc -	2009	Chinese Clinical Trial Registry. 2009	non pertinent
6072	Chi, Ctr Tcc -	2009	Chinese Clinical Trial Registry. 2009	non pertinent
6073	Chi, Ctr Trc -	2009	Chinese Clinical Trial Registry. 2009	non pertinent
6074	Chae et al.,	2009	Neurosci Lett. 2009; 450(2):80-84	non pertinent
6075	Carey et al.,	2009	HIV medicine. 2009; 10(3):163-172	non pertinent
6076	Cantiello et al.,	2009	BJU international. 2009; 103(9):1195-1198	non pertinent
6077	Busto et al.,	2009	Synapse. 2009; 63(8):681-689	non pertinent
6078	Bresolin et al.,	2009	European review for medical and pharmacological sciences. 2009; 13(5):365-370	non pertinent
6079	Bovend'Eerdt et al.,	2009	Clin Rehabil. 2009; 23(2):137-145	non pertinent
6080	Borruto et al.,	2009	Archives of gynecology and obstetrics. 2009; 280(5):707-712	non pertinent
6081	Borckardt et al.,	2009	Pain Med. 2009; 10(5):840-849	non pertinent
6082	Boggio et al.,	2009	Clinical j of Pain. 2009; 25(8):691-695	non pertinent
6083	Blonde et al.,	2009	Diabetes, obesity & metabolism. 2009; 11(10):978-986	non pertinent
6084	Blake et al.,	2009	Anaesthesia and intensive care. 2009; 37(5):720-725	non pertinent
6085	Berlemann et al.,	2009	European spine j. 2009; 18(11):1706-1712	non pertinent

6086	Berger et al.,	2009	American heart j. 2009; 158(6):998-1004.e1001	non pertinent
6087	Berberoglu et al.,	2009	Endocrinologist. 2009; 19(6):271-279	non pertinent
6088	Beran et al.,	2009	BMC infectious diseases. 2009; 9:2	non pertinent
6089	Ben Salah et al.,	2009	Plos neglected tropical diseases. 2009; 3(5):e432	non pertinent
6090	Bell et al.,	2009	Malaria j. 2009; 8:204	non pertinent
6091	Bedi et al.,	2009	Psychopharmacology (Berl). 2009; 207(1):73-83	non pertinent
6092	Banzhoff et al.,	2009	PLoS One. 2009; 4(2):e4384	non pertinent
6093	Aupperle et al.,	2009	CNS spectrums. 2009; 14(10):556-571	non pertinent
6094	Ashwathnarayana et al.,	2009	Vaccine. 2009; 28(1):148-151	non pertinent
6095	Arolt et al.,	2009	Ppmp psychotherapie psychosomatik medizinische psychologie. 2009; 59(3-4):124-131	non pertinent
6096	Apiliogullari et al.,	2009	Paediatric anaesthesia. 2009; 19(11):1078-1083	non pertinent
6097	Apfel et al.,	2009	Pain practice. 2009; 9:119-	non pertinent
6098	Apan et al.,	2009	Minerva Anestesiol. 2009; 75(5):239-244	non pertinent
6099	Andersen et al.,	2009	Spine (Phila Pa 1976). 2009; 34(21):2248-2253	non pertinent
6100	Amaravadi et al.,	2009	Clinical cancer research. 2009; 15(24):7711-7718	non pertinent
6101	Aman et al.,	2009	J of the American Academy of Child and Adolescent Psychiatry. 2009; 48(12):1143-1154	non pertinent
6102	Al-Azemi et al.,	2009	BJOG. 2009; 116(12):1646-1656	non pertinent
6103	Ackerstaff et al.,	2009	Head & neck. 2009; 31(1):77-84	non pertinent
6104	Zatloukal et al.,	2008	J of thoracic oncology. 2008; 3(8):894-901	non pertinent
6105	Yurgelun-Todd et al.,	2008	Experimental and clinical psychopharmacology. 2008; 16(3):191-198	non pertinent

6106	Yuge et al.,	2008	Anesthesia and resuscitation. 2008; 44(SUPPL):135-149	non pertinent
6107	Young et al.,	2008	Haemophilia. 2008; 14(2):287-294	non pertinent
6108	Yi et al.,	2008	Br J Anaesth. 2008; 101(2):234-238	non pertinent
6109	Xu et al.,	2008	PLoS One. 2008; 3(7):e2565	non pertinent
6110	Xie et al.,	2008	Nutrition j. 2008; 7:31	non pertinent
6111	Wurgler-Hauri et al.,	2008	Spine (Phila Pa 1976). 2008; 33(3):E66-72	non pertinent
6112	Worcester et al.,	2008	Primary care – clinics in office practice. 2008; 35(2):369-391	non pertinent
6113	White et al.,	2008	J of clinical hypertension (Greenwich, Conn.). 2008; 10(6):450-458	non pertinent
6114	Whalen et al.,	2008	Biol Psychiatry. 2008; 63(9):858-863	non pertinent
6115	Westin et al.,	2008	Int J Audiol. 2008; 47 Suppl 2:112-118	non pertinent
6116	Weinblatt et al.,	2008	Arthritis and rheumatism. 2008; 58(11):3309-	non pertinent
6117	Warrell et al.,	2008	Plos neglected tropical diseases. 2008; 2(4):e224	non pertinent
6118	Wang et al.,	2008	Acute pain. 2008; 10(2):65-71	non pertinent
6119	Vincenti et al.,	2008	American j of transplantation. 2008; 8(2):307-316	non pertinent
6120	Vemuri et al.,	2008	Physiology & behavior. 2008; 93(4-5):671-686	non pertinent
6121	Unlu et al.,	2008	J of manipulative and physiological therapeutics. 2008; 31(3):191-198	non pertinent
6122	Tveita et al.,	2008	BMC musculoskeletal disorders. 2008; 9:53	non pertinent
6123	Tufanogullari et al.,	2008	Anesthesia and Analgesia. 2008; 106(6):1741-1748	non pertinent
6124	Tuchman et al.,	2008	CNS Drugs. 2008; 22(10):877-886	non pertinent
6125	Tournier-Rangeard et al.,	2008	Radiother Oncol. 2008; 87(3):391-397	non pertinent

6126	Togha et al.,	2008	J of headache and pain. 2008; 9(2):77-82	non pertinent
6127	Thera et al.,	2008	PLoS One. 2008; 3(1):e1465	non pertinent
6128	Terpos et al.,	2008	Expert opinion on drug metabolism & toxicology. 2008; 4(5):639-654	non pertinent
6129	Terney et al.,	2008	J Pain Symptom Manage. 2008; 36(1):79-91	non pertinent
6130	Taucher et al.,	2008	Breast cancer research and treatment. 2008; 112(2)309-316	non pertinent
6131	Szmuk et al.,	2008	Anesthesia and Analgesia. 2008; 107(1):77-80	non pertinent
6132	Sundy et al.,	2008	Arthritis and rheumatism. 2008; 58(9):2882-	non pertinent
6133	Streitberger et al.,	2008	J of alternative and complementary medicine (New York, N.Y.). 2008; 14(5):505-513	non pertinent
6134	Stracke et al.,	2008	Experimental and clinical endocrinology & diabetes. 2008; 116(10):600-605	non pertinent
6135	Stegmann et al.,	2008	Current medical research and opinion. 2008; 24(11):3185-3196	non pertinent
6136	Soyka et al.,	2008	J of clinical psychopharmacology. 2008; 28(3):317-324	non pertinent
6137	Sokunbi et al.,	2008	Nigerian quarterly j of hospital medicine. 2008; 18(4):231-243	non pertinent
6138	Sng et al.,	2008	Anaesthesia and intensive care. 2008; 36(5):659-664	non pertinent
6139	Smeets et al.,	2008	Pain. 2008; 134(3):263-276	non pertinent
6140	Singer et al.,	2008	Emotion. 2008; 8(6):781-791	non pertinent
6141	Sierra et al.,	2008	BMC Cancer. 2008; 8:195	non pertinent
6142	Siddiqui et al.,	2008	Saudi medical j. 2008; 29(7):966-970	non pertinent
6143	Shoham et al.,	2008	Clinical endocrinology. 2008; 69(3):471-478	non pertinent

6144	Sheldon et al.,	2008	Clinical and experimental rheumatology. 2008; 26(4):611-619	non pertinent
6145	Shah et al.,	2008	Indian pediatrics. 2008; 45(10):819-823	non pertinent
6146	Sewell et al.,	2008	Neuroreport. 2008; 19(13):1339-1343	non pertinent
6147	Sengupta et al.,	2008	Arthritis research & therapy. 2008; 10(4):R85	non pertinent
6148	Scott et al.,	2008	Arch Gen Psychiatry. 2008; 65(2).220-231	non pertinent
6149	Schroeter et al.,	2008	Neurobiology of aging. 2008; 29(3):418-426	non pertinent
6150	Scherbaum et al.,	2008	Diabetes, obesity & metabolism. 2008; 10(11):1114-1124	non pertinent
6151	Santarcangelo et al.,	2008	Brain Res Bull. 2008; 75(5):692-697	non pertinent
6152	Sánchez-Guerrero et al.,	2008	J of rheumatology. 2008; 35(8):1567-1575	non pertinent
6153	Roitberg et al.,	2008	Neurosurgery. 2008; 63(1):115-120	non pertinent
6154	Roijezon et al.,	2008	J of neuroengineering and rehabilitation. 2008; 5	non pertinent
6155	Roestenberg et al.,	2008	PLoS One. 2008; 3(12):e3960	non pertinent
6156	Rawlani et al.,	2008	Plastic and reconstructive surgery. 2008; 122(1):39-52	non pertinent
6157	Ramos-Reyna et al.,	2008	Archivos de neurosciencias. 2008; 13(4):237-241	non pertinent
6158	Ramlau et al.,	2008	J of thoracic oncology. 2008; 3(7):735-744	non pertinent
6159	Putzier et al.,	2008	J Neurosurg Spine. 2008; 8(6):536-543	non pertinent
6160	Pradhan et al.,	2008	Kathmandu university medical j (KUJM). 2008; 6(2):166-172	non pertinent
6161	Poreisz et al.,	2008	Neuroreport. 2008; 19(2):193-196	non pertinent
6162	Pihan et al.,	2008	Brain Lang. 2008; 105(2):141-147	non pertinent
6163	Phan et al.,	2008	J Neurosci. 2008; 28(10):2313-2319	non pertinent

6164	Pascual et al.,	2008	J Headache Pain. 2008; 9(5):259-266	non pertinent
6165	Panzer et al.,	2008	Respiratory medicine. 2008; 102(9):1296-1304	non pertinent
6166	Palomba et al.,	2008	Menopause (New York, N.Y.). 2008; 15(4 Pt 1):730-736	non pertinent
6167	Paick et al.,	2008	J of sexual medicine. 2008; 5(11):2672-2680	non pertinent
6168	Ozone et al.,	2008	Pharmacopsychiatry. 2008; 41(3):106-114	non pertinent
6169	Owusu-Agyei et al.,	2008	PLoS One. 2008; 3(6):e2530	non pertinent
6170	Oelkers-Ax et al.,	2008	Eur J Pain. 2008; 12(3):301-313	non pertinent
6171	O'Dea et al.,	2008	Current medical research and opinion. 2008; 24(10):2785-2793	non pertinent
6172	Ntr – Wall	2008	Netherlands Trial Register. 2008	non pertinent
6173	Ntr – Bodden	2008	Netherlands Trial Register. 2008	non pertinent
6174	Nor Azlin et al.,	2008	J of obstetrics and gynaecology. 2008; 28(4):424-426	non pertinent
6175	Nir et al.,	2008	J Pain. 2008; 9(11):1058-1069	non pertinent
6176	Nguyen et al.,	2008	BMC pregnancy and childbirth. 2008; 8:17	non pertinent
6177	Nguyen et al.,	2008	Brain stimulation. 2008; 1(2):89-96	non pertinent
6178	Negaard et al.,	2008	Scand J Gastroenterol. 2008; 43(1):44-51	non pertinent
6179	Ndiaye et al.,	2008	Malaria j. 2008; 7:16	non pertinent
6180	Nct – Keener	2008	National Library of Medicine. 2008	non pertinent
6181	Nct – Haenggi	2008	National Library of Medicine. 2008	non pertinent
6182	Nct - Sambrook	2008	National Library of Medicine. 2008	non pertinent
6183	Nct – Heyer	2008	National Library of Medicine. 2008	non pertinent
6184	Nct – National University Hospital, Singapore	2008	National Library of Medicine. 2008	non pertinent
6185	Nct – Pitman	2008	National Library of Medicine. 2008	non pertinent
6186	Nct - Heishman	2008	National Library of Medicine. 2008	non pertinent
6187	Nct - Paludetti	2008	National Library of Medicine. 2008	non pertinent
6188	Nct - Baraniuk	2008	National Library of Medicine. 2008	non pertinent
6189	Nct - Schwartz	2008	National Library of Medicine. 2008	non pertinent

6190	Nct - Sobell	2008	National Library of Medicine. 2008	non pertinent
6191	Nct - Ekici	2008	National Library of Medicine. 2008	non pertinent
6192	Nct – Ortho-McNeil	2008	National Library of Medicine. 2008	non pertinent
6193	Nct - Weisbord	2008	National Library of Medicine. 2008	non pertinent
6194	Nct - Naylor	2008	National Library of Medicine. 2008	non pertinent
6195	Mullen et al.,	2008	PLoS One. 2008; 3(8):e2940	non pertinent
6196	Moore et al.,	2008	European j of anaesthesiology. 2008; 25(11):876-883	non pertinent
6197	Misra et al.,	2008	J Pain. 2008; 9(12):1116-1122	non pertinent
6198	Miskowiak et al.,	2008	Neuropsychopharmacology. 2008; 33(3):611-618	non pertinent
6199	Minnemann et al.,	2008	J of endocrinological investigation. 2008; 31(8):718-723	non pertinent
6200	Miller et al.,	2008	Surgical endoscopy. 2008; 22(11):2478-2484	non pertinent
6201	Milanés-Virelles et al.,	2008	BMC infectious diseases. 2008; 8:17	non pertinent
6202	Mercadante et al.,	2008	J of clinical monitoring and computing. 2008; 22(4):293-298	non pertinent
6203	Mendelson et al.,	2008	Neuropsychopharmacology. 2008; 33(4):749-760	non pertinent
6204	Mehta et al.,	2008	Psychopharmacology (Berl). 2008; 196(1):157-165	non pertinent
6205	McCormack et al.,	2008	Drugs. 2008; 68(4):487-506	non pertinent
6206	Maurer et al.,	2008	J Spinal Disord Tech. 2008; 21(1):55-62	non pertinent
6207	Mastronardi et al.,	2008	Spine (Phila Pa 1976). 2008; 33(14):1562-1566	non pertinent
6208	Marquis et al.,	2008	Osteoporosis international. 2008; 19(4):503-510	non pertinent
6209	Margetts et al.,	2008	European j of anaesthesiology. 2008; 25(12):1009-1013	non pertinent
6210	Marcellin et al.,	2008	New England j of medicine. 2008; 359(23):2442-2455	non pertinent
6211	Mañas et al.,	2008	Clinical & translational oncology. 2008; 10(5):281-287	non pertinent

6212	Maiteki-Sebuguzi et al.,	2008	Malaria j. 2008; 7:106	non pertinent
6213	Magnusson et al.,	2008	Spine. 2008; 33(16):E532-538	non pertinent
6214	Mackay-Sim et al.,	2008	Brain. 2008; 131(Pt 9):2376-2386	non pertinent
6215	Machata et al.,	2008	Br J Anaesth. 2008; 101(2):239-243	non pertinent
6216	Liu et al.,	2008	Phytotherapy research : PTR. 2008; 22(11):1539-1543	non pertinent
6217	Liu et al.,	2008	Acta Neurochir Suppl. 2008; 101:71-76	non pertinent
6218	Lee et al.,	2008	International clinical psychopharmacology. 2008; 23(6):337-341	non pertinent
6219	Lee et al.,	2008	Korean j of biological psychiatry. 2008; 15(1):35-45	non pertinent
6220	Leach et al.,	2008	Pediatric infectious disease j. 2008; 27(8):692-698	non pertinent
6221	Lauwick et al.,	2008	J canadien d'anesthesie (Canadian j of anaesthesia). 2008; 55(11):754-760	non pertinent
6222	Lamm et al.,	2008	Brain Res. 2008; 1227:153-161	non pertinent
6223	Kyle et al.,	2008	International j of clinical practice. 2008; 62(11):1684-1692	non pertinent
6224	Kweku et al.,	2008	PLoS One. 2008; 3(12):e4000	non pertinent
6225	Kowalski et al.,	2008	Clinical pharmacology and therapeutics. 2008; 83(6):857-866	non pertinent
6226	Korovessis et al.,	2008	J of spinal disorders & techniques. 2008; 21(4):293-298	non pertinent
6227	Kordon et al.,	2008	J of clinical psychopharmacology. 2008; 28(5):550-554	non pertinent
6228	Kocaman et al.,	2008	Turkiye fiziksel tip ve rehabilitasyon dergisi. 2008; 54(2):54-58	non pertinent

6229	Kobayashi et al.,	2008	Japanese j of chemotherapy. 2008; 56(SUPPL 1):36-48	non pertinent
6230	Ko et al.,	2008	Arthroscopy. 2008; 24(9):1005-1012	non pertinent
6231	Kleinert et al.,	2008	Anesthesia and Analgesia. 2008; 107(6):2048-2055	non pertinent
6232	Kim et al.,	2008	Helicobacter. 2008; 13(6):542-549	non pertinent
6233	Kim et al.,	2008	Helicobacter. 2008; 13(4):261-268	non pertinent
6234	Kim et al.,	2008	J Neurosurg Anesthesiol. 2008; 20(1):1-7	non pertinent
6235	Killgore et al.,	2008	Neuroreport. 2008; 19(15):1523-1527	non pertinent
6236	Kilic et al.,	2008	Digestive diseases and sciences. 2008; 53(12):3133-3137	non pertinent
6237	Keystone et al.,	2008	Arthritis and rheumatism. 2008; 58(11):3319-	non pertinent
6238	Keller et al.,	2008	Pain Med. 2008; 9(6):680-687	non pertinent
6239	Kearney et al.,	2008	Nature clinical practice gastroenterology and hepatology. 2008; 5(11):624-636	non pertinent
6240	Kaye	2008	Canadian urological association j. 2008; 2(6):604-608	non pertinent
6241	Kaviani et al.,	2008	World j of surgery. 2008; 32(11):2464-2470	non pertinent
6242	Katz et al.,	2008	International j of clinical practice. 2008; 62(9):1455-1464	non pertinent
6243	Karabekir et al.,	2008	Neurosciences (riyadh, saudi arabia). 2008; 13(3):248-252	non pertinent
6244	Kang et al.,	2008	J Clin Anesth. 2008; 20(1):12-16	non pertinent
6245	Kalman et al.,	2008	Nutrition j. 2008; 7:3	non pertinent
6246	Kalia et al.,	2008	The lancet neurology. 2008; 7(8):742-755	non pertinent
6247	Jones et al.,	2008	Arthritis Rheum. 2008; 58(2):612-622	non pertinent
6248	Jensch et al.,	2008	AJR Am J Roentgenol. 2008; 191(1):158-167	non pertinent

6249	Jadidi et al.,	2008	J of oral rehabilitation. 2008; 35(3):171-183	non pertinent
6250	Isrctn - Bongi	2008	ISRCTN Registry. 2008	non pertinent
6251	Isrctn – Choy	2008	ISRCTN Registry. 2008	non pertinent
6252	Isrctn – Toukhsati	2008	ISRCTN Registry. 2008	non pertinent
6253	Isrctn - Jiya	2008	ISRCTN Registry. 2008	non pertinent
6254	Huang et al.,	2008	BMC musculoskeletal disorders. 2008; 9:77	non pertinent
6255	Hua et al.,	2008	Cell Mol Neurobiol. 2008; 28(1):57-70	non pertinent
6256	Hu et al.,	2008	PLoS One. 2008; 3(4):e1952	non pertinent
6257	Hong et al.,	2008	Eur J Anaesthesiol. 2008; 25(6):460-467	non pertinent
6258	Hong et al.,	2008	Anesthesia and Analgesia. 2008; 107(3):1005-1010	non pertinent
6259	Holmes et al.,	2008	Epilepsy Res. 2008; 82(2-3):124-132	non pertinent
6260	Hoft et al.,	2008	J of infectious diseases. 2008; 198(10):1491-1501	non pertinent
6261	Hirsch et al.,	2008	European j of medical research. 2008; 13(11):505-510	non pertinent
6262	Harms et al.,	2008	PLoS One. 2008; 3(5):e2158	non pertinent
6263	Harada et al.,	2008	Japanese pharmacology and therapeutics. 2008; 36(2):129-140	non pertinent
6264	Hadjikhani et al.,	2008	Brain Cogn. 2008; 68(1):1-8	non pertinent
6265	Habib et al.,	2008	Anesthesia and Analgesia. 2008; 107(3):999-1004	non pertinent
6266	Habib et al.,	2008	Anesthesiology. 2008; 109(6):1085-1091	non pertinent
6267	Gürkov et al.,	2008	Malaria j. 2008; 7:179	non pertinent
6268	Gurbet et al.,	2008	European spine j. 2008; 17(9):1237-1241	non pertinent
6269	Graziani et al.,	2008	J of craniofacial surgery. 2008; 19(4):1061-1066	non pertinent
6270	Goff et al.,	2008	Schizophrenia research. 2008; 106(2-3):320-327	non pertinent

6271	Godard et al.,	2008	Respiratory medicine. 2008; 102(8):1124-1131	non pertinent
6272	Goadsby et al.,	2008	Cephalalgia. 2008; 28(4):383-391	non pertinent
6273	Goadsby	2008	Cephalalgia. 2008; 28(Suppl 2):36-41	non pertinent
6274	Ge et al.,	2008	Exp Brain Res. 2008; 187(4)::623-629	non pertinent
6275	Ganesh et al.,	2008	Anesthesiology. 2008; 109(5):890-894	non pertinent
6276	Galan-Herrera et al.,	2008	Clinical therapeutics. 2008; 30(9):1667-1674	non pertinent
6277	Furie et al.,	2008	Arthritis research & therapy. 2008; 10(5):R109	non pertinent
6278	Frestedt et al.,	2008	Nutrition j. 2008; 7:9	non pertinent
6279	Fregni et al.,	2008	J Clin Psychiatry. 2008; 69(1):32-40	non pertinent
6280	Franzen-Korzendorfer et al.,	2008	Ostomy Wound Manage. 2008; 54(6):16-31	non pertinent
6281	Foley et al.,	2008	Spine J. 2008; 8(3):436-442	non pertinent
6282	Feldblum et al.,	2008	PLoS One. 2008; 3(1):e1474	non pertinent
6283	Fassoulaki et al.,	2008	Anesthesia and Analgesia. 2008; 107(5):1715-1719	non pertinent
6284	Fadel et al.,	2008	Egyptian j of anaesthesia. 2008; 24(4):275-284	non pertinent
6285	Euctr, S.E.	2008	EU Clinical Trials Register. 2008	non pertinent
6286	Euctr, N.L.	2008	EU Clinical Trials Register. 2008	non pertinent
6287	Elewski et al.,	2008	J of the American Academy of Dermatology. 2008; 59(1):41-54	non pertinent
6288	Eippert et al.,	2008	J Neurosci. 2008; 28(21):5465-5472	non pertinent
6289	Ebell	2008	Contemporary hypnosis. 2008; 25(1):46-56	non pertinent
6290	E Silva et al.,	2008	Paediatric anaesthesia. 2008; 18(2):176-183	non pertinent
6291	Drapier et al.,	2008	Neuropsychologia. 2008; 46(11):2796-2801	non pertinent
6292	Dougherty et al.,	2008	Behav Brain Res. 2008; 193(1):63-68	non pertinent
6293	Derntl et al.,	2008	Psychoneuroendocrinology. 2008; 33(8):1031-1040	non pertinent

6294	Denenberg et al.,	2008	J Am Vet Med Assoc. 2008; 233(12):1874-1882	non pertinent
6295	Delmas et al.,	2008	Osteoporosis international. 2008; 19(7):1039-1045	non pertinent
6296	De Leeuw et al.,	2008	Pain practice. 2008; 8(4):241-247	non pertinent
6297	De La Garza et al.,	2008	Int J Neuropsychopharmacol. 2008; 11(6):729-741	non pertinent
6298	Davidson et al.,	2008	Reviews in cardiovascular medicine. 2008; 9(SUPPL 1):24-34	non pertinent
6299	Cornwell et al.,	2008	Brain Res. 2008; 1244:103-112	non pertinent
6300	Cornuz et al.,	2008	PLoS One. 2008; 3(6):e2547	non pertinent
6301	Cok et al.,	2008	Acta anaesthesiologica belgica. 2008; 59(1):27-32	non pertinent
6302	Codipietro et al.,	2008	Pediatrics. 2008; 122(3):e716-721	non pertinent
6303	Chuang et al.,	2008	J Clin Neurosci. 2008; 15(11):1210-1215	non pertinent
6304	Chotpitayasunondh et al.,	2008	PLoS One. 2008; 3(12):e4028	non pertinent
6305	Chacko et al.,	2008	J Pediatr Adolesc Gynecol. 2008; 21(4):187-193	non pertinent
6306	Cerqueira et al.,	2008	JACC Cardiovasc Imaging. 2008; 1(3):307-316	non pertinent
6307	Casey et al.,	2008	Psychopharmacology. 2008; 200(3):317-331	non pertinent
6308	Casale et al.,	2008	Neuroreport. 2008; 19(16):1633-1636	non pertinent
6309	Casajuana et al.,	2008	BMC blood disorders. 2008; 8:1TN:NCT00137579/ClinicalTrials.gov.	non pertinent
6310	Cartwright et al.,	2008	Clinical colorectal cancer. 2008; 7(6):390-397	non pertinent
6311	Carneiro et al.,	2008	Rev Bras Anestesiol. 2008; 58(6):569-581	non pertinent
6312	Carey et al.,	2008	Neurorehabil Neural Repair. 2008; 22(2):185-192	non pertinent
6313	Carbajal et al.,	2008	Pediatrics. 2008; 121(6):e1591-1598	non pertinent

6314	Calabrese et al.,	2008	J of alternative and complementary medicine (New York, N.Y.). 2008; 14(6):707-713	non pertinent
6315	Cakan et al.,	2008	J of neurosurgical anesthesiology. 2008; 20(3):169-173	non pertinent
6316	Bryant et al.,	2008	J of psychiatry & neuroscience. 2008; 33(2):142-146	non pertinent
6317	Brandt et al.,	2008	European j of obstetrics, gynecology, and reproductive biology. 2008; 141(2):158-162	non pertinent
6318	Boggio et al.,	2008	Eur J Neurol. 2008; 15(10):1124-1130	non pertinent
6319	Bodsworth et al.,	2008	Sexual health. 2008; 5(3):219-225	non pertinent
6320	Bjorkman et al.,	2008	Aging clinical and experimental research. 2008; 20(4):315-321	non pertinent
6321	Bhagat et al.,	2008	Anesth Analg. 2008; 107(4):1348-1355	non pertinent
6322	Berman et al.,	2008	J Neurosci. 2008; 28(2):349-359	non pertinent
6323	Belavy et al.,	2008	Spine (Phila Pa 1976). 2008; 33(5):E121-131	non pertinent
6324	Behets et al.,	2008	Sexually transmitted diseases. 2008; 35(9):818-826	non pertinent
6325	Baumgartner et al.,	2008	Neuron. 2008; 58(4):639-650	non pertinent
6326	Batra et al.,	2008	Biological psychiatry. 2008; 63(12):1178-1184	non pertinent
6327	Barkay et al.,	2008	Surgical endoscopy. 2008; 22(9):1971-1976	non pertinent
6328	Barakat et al.,	2008	Anesth Analg. 2008; 106(3):823-829	non pertinent
6329	Banzett et al.,	2008	Am J Respir Crit Care Med. 2008; 177(12):1384-1390	non pertinent
6330	Balconi et al.,	2008	International j of neuroscience. 2008; 118(10):1412-1424	non pertinent

6331	Babiloni et al.,	2008	Brain Res Bull. 2008; 75(5):581-590	non pertinent
6332	Babiloni et al.,	2008	J Pain. 2008; 9(10):902-911	non pertinent
6333	Ash et al.,	2008	AJNR Am J Neuroradiol. 2008; 29(6):1098-1103	non pertinent
6334	Arle et al.,	2008	J Neurosurg. 2008; 109(1):133-139	non pertinent
6335	Arai et al.,	2008	J of cancer research and clinical oncology. 2008; 134(12):1385-1396	non pertinent
6336	Apostol et al.,	2008	Headache. 2008; 48(7):1012-1025	non pertinent
6337	Andersen et al.,	2008	Exp Brain Res. 2008; 191(3):371-382	non pertinent
6338	Al'Absi et al.,	2008	Psychosom Med. 2008; 70(8):928-935	non pertinent
6339	Aggarwal et al.,	2008	Indian j of medical sciences. 2008; 62(5):179-184	non pertinent
6340	Addeo et al.,	2008	Cancer. 2008; 113(9):2524-2531	non pertinent
6341	Abulmagd et al.,	2008	Egyptian j of anaesthesia. 2008; 24(2):153-159	non pertinent
6342	Aasvang et al.,	2008	Anesthesia and Analgesia. 2008; 107(1):282-291	non pertinent
6343	Zimbroff et al.,	2007	International clinical psychopharmacology. 2007; 22(6):363-370	non pertinent
6344	Zhou et al.,	2007	Pediatric infectious disease j. 2007; 26(11):1001-1005	non pertinent
6345	Zaba et al.,	2007	CNS Drugs. 2007; 21(2):165-171	non pertinent
6346	Yu et al.,	2007	Am J Chin Med. 2007; 35(6):937-945	non pertinent
6347	Yang et al.,	2007	Br J Anaesth. 2007; 98(4):515-518	non pertinent
6348	Yamashiro et al.,	2007	J Neurol Neurosurg Psychiatry. 2007; 78(5):497-500	non pertinent
6349	Xiao et al.,	2007	J of clinical rehabilitative tissue engineering research. 2007; 11(8):1443-1446	non pertinent

6350	Wu et al.,	2007	Zhonghua shao shang za zhi (Chinese j of burns). 2007; 23(6):440-443	non pertinent
6351	Wu et al.,	2007	Helicobacter. 2007; 12(6):633-637	non pertinent
6352	White et al.,	2007	Clinical J of Pain. 2007; 23(8):714-719	non pertinent
6353	Watson et al.,	2007	Neuroreport. 2007; 18(8):771-775	non pertinent
6354	Wang et al.,	2007	J Altern Complement Med. 2007; 13(2):241-246	non pertinent
6355	Wang et al.,	2007	Anesth Analg. 2007; 104(2):325-331	non pertinent
6356	Wang et al.,	2007	J of clinical rehabilitative tissue engineering research. 2007; 11(28):5536-5539	non pertinent
6357	Von Arnim et al.,	2007	American j of gastroenterology. 2007; 102(6):1268-1275	non pertinent
6358	Vandenberghe et al.,	2007	Gastroenterology. 2007; 132(5):1684-1693	non pertinent
6359	Van Der Veen et al.,	2007	Neuropsychopharmacology. 2007; 32(1):216-224	non pertinent
6360	Vad et al.,	2007	Arch Phys Med Rehabil. 2007; 88(5):577-582	non pertinent
6361	Tursi et al.,	2007	Medical science monitor. 2007; 13(4):CR187-190	non pertinent
6362	Torisu et al.,	2007	Clin Neurophysiol. 2007; 118(5):999-1009	non pertinent
6363	Tekin et al.,	2007	Current therapeutic reseach – clinical and experimental. 2007; 68(5):313-324	non pertinent
6364	Takahashi-Yanaga et al.,	2007	J of pharmacological sciences. 2007; 104(4):293-302	non pertinent
6365	Suzuki et al.,	2007	Arch Oral Biol. 2007; 52(8):797-802	non pertinent
6366	Struys et al.,	2007	Br J Anaesth. 2007; 99(3):359-367	non pertinent

6367	Stravodimos et al.,	2007	International urology and nephrology. 2007; 39(3):893-896	non pertinent
6368	Staud et al.,	2007	Pain. 2007; 129(1-2):130-142	non pertinent
6369	Skouteri et al.,	2007	Acta anaesthesiologica belgica. 2007; 58(3):169-175	non pertinent
6370	Sinis et al.,	2007	Clin J Pain. 2007; 23(3):237-243	non pertinent
6371	Silvanto et al.,	2007	Acta anaesthesiologica Scandinavica. 2007; 51(9):1147-1154	non pertinent
6372	Siemer et al.,	2007	European urology. 2007; 52(4):1156-1163	non pertinent
6373	Sesti et al.,	2007	Fertility and sterility. 2007; 88(6):1541-1547	non pertinent
6374	Seidel et al.,	2007	Rheumatology international. 2007; 27(11):1025-1030	non pertinent
6375	Schmidt et al.,	2007	Anesthesiology. 2007; 106(4):707-714	non pertinent
6376	Schienle et al.,	2007	Eur Arch Psychiatry Clin Neurosci. 2007; 257(8):486-493	non pertinent
6377	Sansone-Parsons et al.,	2007	Pharmacotherapy. 2007; 27(6):825-834	non pertinent
6378	Sänger et al.,	2007	European j of pediatrics. 2007; 166(12):1229-1236	non pertinent
6379	Saletu et al.,	2007	Neuropsychobiology. 2007; 55(3-4):184-193	non pertinent
6380	Saarela et al.,	2007	Cereb Cortex. 2007; 17(1):230-237	non pertinent
6381	Saad et al.,	2007	Clinical genitourinary cancer. 2007; 5(6):390-396	non pertinent
6382	Rudick et al.,	2007	Ann Neurol. 2007; 62(4):335-346	non pertinent
6383	Rodriguez-Navarro et al.,	2007	Anesthesiology. 2007; 106(2):339-345	non pertinent
6384	Robertson et al.,	2007	J Clin Psychiatry. 2007; 68(2):261-267	non pertinent
6385	Robbins et al.,	2007	Clinical therapeutics. 2007; 29(11):2349-2364	non pertinent

6386	Rios et al.,	2007	Neurourology and urodynamics. 2007; 26(6):773-778	non pertinent
6387	Ringe et al.,	2007	Rheumatology international. 2007; 27(5):425-434	non pertinent
6388	Ray et al.,	2007	J Physiol. 2007; 585(Pt 1):287-294	non pertinent
6389	Rauck et al.,	2007	J of opioid management. 2007; 3(1):35-43	non pertinent
6390	Rahman Al-Refai et al.,	2007	Minerva Anestesiologica. 2007; 73(4):219-223	non pertinent
6391	Quan et al.,	2007	Zhongguo yi xue ke xue bao. Acta academiae medicinae sinicae. 2007; 29(1):107-110	non pertinent
6392	Prevenzano et al.,	2007	Clinical nephrology. 2007; 67(5):306-317	non pertinent
6393	Price et al.,	2007	Pain. 2007; 127(1-2):63-72	non pertinent
6394	Prairie et al.,	2007	Contraception. 2007; 76(5):383-388	non pertinent
6395	Phillips et al.,	2007	J Oral Maxillofac Surg. 2007; 65(6):1162-1173	non pertinent
6396	Patrocinio et al.,	2007	Brazilian j of otorhinolaryngology. 2007; 73(3):339-342	non pertinent
6397	Patil et al.,	2007	Nature medicine. 2007; 13(9):1102-1107	non pertinent
6398	Passard et al.,	2007	Brain. 2007; 130(Pt 10):2661-2670	non pertinent
6399	Park et al.,	2007	Current treatment options in cardiovascular medicine. 2007; 9(1):37-45	non pertinent
6400	Park et al.,	2007	Nuclear medicine communications. 2007; 28(10):766-770	non pertinent
6401	Papp et al.,	2007	J of cutaneous medicine and surgery. 2007; 11(2):57-66	non pertinent
6402	Oz et al.,	2007	Saudi medical j. 2007; 28(7):1055-1058	non pertinent
6403	Ondo et al.,	2007	Clinical neuropharmacology. 2007; 30(5):295-300	non pertinent

6404	Oncken et al.,	2007	Addict Behav. 2007; 32(2):296-309	non pertinent
6405	Omer et al.,	2007	Phytomedicine. 2007; 14(2-3):87-95	non pertinent
6406	Ochia et al.,	2007	Spine. 2007; 32(13):1394-1399	non pertinent
6407	Norbury et al.,	2007	British j of psychiatry. 2007; 190(JUNE):531-532	non pertinent
6408	Niddam et al.,	2007	Clin J Pain. 2007; 23(5):440-448	non pertinent
6409	Nct – NuVasive	2007	National Library of Medicine. 2007	non pertinent
6410	Nct – Redaelli	2007	National Library of Medicine. 2007	non pertinent
6411	Nct – Fitzgerald	2007	National Library of Medicine. 2007	non pertinent
6412	Nct – Brefel-Courbon	2007	National Library of Medicine. 2007	non pertinent
6413	Nct – Kusek	2007	National Library of Medicine. 2007	non pertinent
6414	Nct - Bell	2007	National Library of Medicine. 2007	non pertinent
6415	Nct – Barber	2007	National Library of Medicine. 2007	non pertinent
6416	Nct – Avery	2007	National Library of Medicine. 2007	non pertinent
6417	Nct - Morone	2007	National Library of Medicine. 2007	non pertinent
6418	Napadow et al.,	2007	Pain. 2007; 130(3):254-266	non pertinent
6419	Nagy et al.,	2007	J of B. O. ON. 2007; 389-394	non pertinent
6420	Moréot-Panzini et al.,	2007	J of neurophysiology. 2007; 97(2):1396-1404	non pertinent
6421	Miskowiak et al.,	2007	Biol Psychiatry. 2007; 62(11):1244-1250	non pertinent
6422	Miner et al.,	2007	J Emerg Med. 2007; 32(3):249-255	non pertinent
6423	Messier et al.,	2007	Osteoarthritis and cartilage. 2007; 15(11):1256-1266	non pertinent
6424	Mehrkens et al.,	2007	Acta Neurochir Suppl. 2007; 97(Pt 2):91-97	non pertinent
6425	Mathias et al.,	2007	Clinical therapeutics. 2007; 29(5):950-962	non pertinent
6426	Mason et al.,	2007	Exp Brain Res. 2007; 182(1):81-91	non pertinent
6427	Marson et al.,	2007	Health technology assessment (Winchester, England). 2007; 11(37):iii-iv, ix-x, 1-134	non pertinent

6428	Marier et al.,	2007	J of clinical pharmacology. 2007; 47(11):1381-1389	non pertinent
6429	Maihofner et al.,	2007	Eur J Neurosci. 2007; 26(5):1344-1356	non pertinent
6430	Madisch et al.,	2007	International j of colorectal disease. 2007; 22(12):1445-1451	non pertinent
6431	Ma et al.,	2007	Cancer chemotherapy and pharmacology. 2007; 59(2):207-215	non pertinent
6432	Lundquist	2007	Advances in therapy. 2007; 24(2):333-345	non pertinent
6433	Lundahl et al.,	2007	Psychopharmacology (Berl). 2007; 195(1):139-146	non pertinent
6434	Loo et al.,	2007	Singapore medical j. 2007; 48(9):834-839	non pertinent
6435	Ledowski et al.,	2007	Br J Anaesth. 2007; 99(4):547-551	non pertinent
6436	Le Pera et al.,	2007	Eur J Neurosci. 2007; 25(6):1900-1907	non pertinent
6437	Lanzi et al.,	2007	Psychopathology. 2007; 40(1):1-7	non pertinent
6438	Kujundzic et al.,	2007	J of surgical oncology. 2007; 96(6):518-524	non pertinent
6439	Krzeski et al.,	2007	Arthritis research & therapy. 2007; 9(5):R109	non pertinent
6440	Kramer et al.,	2007	Pain. 2007; 133(1-3):72-78	non pertinent
6441	Korostenskaja et al.,	2007	Brain Res Bull. 2007; 72(4-6):275-283	non pertinent
6442	Ko et al.,	2007	J of musculoskeletal pain. 2007; 15(1):11-19	non pertinent
6443	Klausner et al.,	2007	Current medical research and opinion. 2007; 23(11):2637-2645	non pertinent
6444	Klarenbeek et al.,	2007	BMC Surg. 2007; 7:16	non pertinent
6445	Kiss et al.,	2007	Brain. 2007; 130(Pt 11):2879-2886	non pertinent
6446	Kisicki et al.,	2007	Clinical therapeutics. 2007; 29(9):1967-1979	non pertinent
6447	Kim et al.,	2007	Clinical therapeutics. 2007; 29(9):1924-1936	non pertinent

6448	Khaw et al.,	2007	Ophthalmology. 2007; 114(10):1822-1830	non pertinent
6449	Kettenmann et al.,	2007	Clin J Pain. 2007; 23(8):663-668	non pertinent
6450	Kazemisaeid et al.,	2007	Indian heart j. 2007; 59(6):454-458	non pertinent
6451	Karlsen et al.,	2007	J Endourol. 2007; 21(1):28-33	non pertinent
6452	Kang et al.,	2007	Helicobacter. 2007; 12(6):623-628	non pertinent
6453	Jabbari	2007	Current pain and headache reports. 2007; 11(5):352-358	non pertinent
6454	Ito et al.,	2007	Muscle & nerve. 2007; 35(3):379-382	non pertinent
6455	Isrctn - Levine	2007	ISRCTN Registry. 2007	non pertinent
6456	Ingelmo et al.,	2007	Paediatric anaesthesia. 2007; 17(12):1166-1175	non pertinent
6457	Hunt et al.,	2007	J of behavior therapy and experimental psychiatry. 2007; 38(4):329-344	non pertinent
6458	Hui et al.,	2007	BMC Commplement Altern Med. 2007; 7:33	non pertinent
6459	Horsley et al.,	2007	Aust J Physiother. 2007; 53(4):239-245	non pertinent
6460	Hoff et al.,	2007	Scand J Gastroenterol. 2007; 42(7):885-889	non pertinent
6461	Hirsh et al.,	2007	Trials. 2007; 8	non pertinent
6462	Hemmeter et al.,	2007	J Psychiatr Res. 2007; 41(10):876-884	non pertinent
6463	Hatem et al.,	2007	Clin Neurophysiol. 2007; 118(7):1503-1516	non pertinent
6464	Harrison et al.,	2007	J of clinical pharmacology. 2007; 47(8):962-969	non pertinent
6465	Hamann et al.,	2007	J of opioid management. 2007; 3(3):137-143	non pertinent
6466	Hagenbach et al.,	2007	Spinal Cord. 2007; 45(8):551-562	non pertinent
6467	Haavik-Taylor et al.,	2007	Clinical Neurophysiology. 2007; 118(2):391-402	non pertinent

6468	Gurpegui et al.,	2007	European neuropsychopharmacology. 2007; 17(11):725-734	non pertinent
6469	Guedj et al.,	2007	European j of nuclear medicine and molecular imaging. 2007; 34(12):2115-2119	non pertinent
6470	Gross et al.,	2007	J of the european academy of dermatology and venereology : JEADV. 2007; 21(10):1404-1412	non pertinent
6471	Gridelli et al.,	2007	Lancet Oncol. 2007; 8(6):500-512	non pertinent
6472	Gordon et al.,	2007	J of neuroscience methods. 2007; 159(2):300-307	non pertinent
6473	Goossens et al.,	2007	Biol Psychiatry. 2007; 62(10):1119-1125	non pertinent
6474	Glassman et al.,	2007	Spine. 2007; 32(15):1693-1698	non pertinent
6475	Giamberardino et al.,	2007	J of Pain. 2007; 8(11):869-878	non pertinent
6476	Ghosh et al.,	2007	J of the indian medical association. 2007; 105(5):260-262	non pertinent
6477	Ghafoor et al.,	2007	American j of health-system pharmacy. 2007; 64(23):2447-2461	non pertinent
6478	Gerich et al.,	2007	Current medical research and opinion. 2007; 23(8):1791-1798	non pertinent
6479	George et al.,	2007	Schizophrenia research. 2007; 93(1-3):42-50	non pertinent
6480	Geha et al.,	2007	Pain. 2007; 128(1-2):88-100	non pertinent
6481	Gega et al.,	2007	International j of nursing studies. 2007; 44(3):397-405	non pertinent
6482	Gau et al.,	2007	J of child and adolescent psychopharmacology. 2007; 17(4):447-460	non pertinent
6483	Garside et al.,	2007	Health technology assessment. 2007; 11(45):iii-128	non pertinent
6484	Ga et al.,	2007	J of rehabilitation medicine. 2007; 39(5):374-378	non pertinent
6485	Franken et al.,	2007	Alcohol Clin Exp Res. 2007; 31(6):919-927	non pertinent

6486	Filipov et al.,	2007	Anaesthesiology and intensive care. 2007; 34(4):3-13	non pertinent
6487	Ferreira et al.,	2007	Pain. 2007; 131(1-2):31-37	non pertinent
6488	Fernández-Montequin et al.,	2007	International wound j. 2007; 4(4):333-343	non pertinent
6489	Euctr, I.T.	2007	EU Clinical Trials Register. 2007	non pertinent
6490	Etienne-Grimaldi et al.,	2007	Clinical pharmacokinetics. 2007; 46(11):953-963	non pertinent
6491	Ekman et al.,	2007	Anesth Analg. 2007; 105(3):688-695	non pertinent
6492	Dubey et al.,	2007	American j of respiratory and critical care medicine. 2007; 175(9):868-874	non pertinent
6493	Driessen et al.,	2007	BMC Psychiatry. 2007; 7:58	non pertinent
6494	Down et al.,	2007	British j of sports medicine. 2007; 41(8):501-505	non pertinent
6495	Donati et al.,	2007	Seizure. 2007; 16(8):670-679	non pertinent
6496	Domes et al.,	2007	Biological psychiatry. 2007; 62(10):1187-1190	non pertinent
6497	DiPalma et al.,	2007	Southern medical j. 2007; 100(11):1085-1090	non pertinent
6498	Dicko et al.,	2007	PLoS One. 2007; 2(10):e1045	non pertinent
6499	Dib et al.,	2007	Brazilian j of otorhinolaryngolo gy. 2007; 73(3):390-397	non pertinent
6500	Dawood et al.,	2007	American j of obstetrics and gynecology. 2007; 196(1):35.e31-35	non pertinent
6501	Dall'Era et al.,	2007	Arthritis and rheumatism. 2007; 56(12):4142-	non pertinent
6502	Curry et al.,	2007	J of emergency medicine. 2007; 33(4):407-411	non pertinent
6503	Couppé et al.,	2007	Clinical J of Pain. 2007; 23(1):23-27	non pertinent
6504	Conklin et al.,	2007	J of pediatric psychology. 2007; 32(9):1127-1139	non pertinent
6505	Cole	2007	Current pain and headache reports. 2007; 11(2):89-92	non pertinent
6506	Citrome et al.,	2007	International clinical psychopharmacol ogy. 2007; 22(6):356-362	non pertinent

6507	Chung et al.,	2007	Annals of surgery. 2007; 246(5):728-733	non pertinent
6508	Choi et al.,	2007	Helicobacter. 2007; 12(6):638-642	non pertinent
6509	Chiappori et al.,	2007	Clinical cancer research. 2007; 13(7):2091-2099	non pertinent
6510	Ceballos et al.,	2007	Biol Psychol. 2007; 74(3):414-416	non pertinent
6511	Cavusoglu et al.,	2007	Turk Neurosurg. 2007; 17(2):100-108	non pertinent
6512	Caumo et al.,	2007	Anesthesia and Analgesia. 2007; 105(5):1263-1271	non pertinent
6513	Caliskan et al.,	2007	European j of contraception & reproductive health care. 2007; 12(4):372-377	non pertinent
6514	Buxbaum et al.,	2007	American j of physical medicine & rehabilitation. 2007; 86(7):527-537	non pertinent
6515	Butts et al.,	2007	J of clinical oncology. 2007; 25(36):5777-5784	non pertinent
6516	Busch et al.,	2007	Cephalalgia. 2007; 27(2):165-172	non pertinent
6517	Busch et al.,	2007	Cephalalgia. 2007; 27(11):1206-1214	non pertinent
6518	Brown et al.,	2007	Calcified tissue international. 2007; 81(5):341-351	non pertinent
6519	Britton et al.,	2007	CNS Spectr. 2007; 12(8):600-605	non pertinent
6520	Brearley et al.,	2007	International j of clinical pharmacology and therapeutics. 2007; 45(6):307-318	non pertinent
6521	Borner et al.,	2007	Der Anaesthesist. 2007; 56(11):1120-1127	non pertinent
6522	Borckardt et al.,	2007	Pain Res Manag. 2007; 12(4):287-290	non pertinent
6523	Bonnema et al.,	2007	J Clin Endocrinol Metab. 2007; 92(9):3424-3428	non pertinent
6524	Bonhomme et al.,	2007	J of neurosurgical anesthesiology. 2007; 19(1):49-55	non pertinent

6525	Bone et al.,	2007	J of clinical endocrinology and metabolism. 2007; 92(12):4671-4677	non pertinent
6526	Boly et al.,	2007	Proc Natl Acad Sci U S A. 2007; 104(29):12187-12192	non pertinent
6527	Boddaert et al.,	2007	Blood. 2007; 110(1):401-408	non pertinent
6528	Block et al.,	2007	Antimicrobial agents and chemotherapy. 2007; 51(11):4001-4008	non pertinent
6529	Blanchard et al.,	2007	Behav Res Ther. 2007; 45(4):633-648	non pertinent
6530	Biasiotta et al.,	2007	Clin Neurophysiol. 2007; 118(10):2180-2188	non pertinent
6531	Bennell et al.,	2007	BMC Musculoskelet Disord. 2007; 8:96	non pertinent
6532	Becker et al.,	2007	Hepatology (baltimore, md.). 2007; 45(1):9-15	non pertinent
6533	Bays et al.,	2007	Diabetes & vascular disease research. 2007; 4(3):181-193	non pertinent
6534	Barst	2007	Expert opinion on pharmacotherapy. 2007; 8(1):95-109	non pertinent
6535	Barbanoj et al.,	2007	Neuropsychobiology. 2007; 55(3-4):203-212	non pertinent
6536	Bangs et al.,	2007	J of child and adolescent psychopharmacology. 2007; 17(4):407-420	non pertinent
6537	Babiloni et al.,	2007	Brain Res Bull. 2007; 71(5):475-484	non pertinent
6538	Azzaro et al.,	2007	J of clinical pharmacology. 2007; 47(8):978-990	non pertinent
6539	Ashwin et al.,	2007	Neuropsychologia. 2007; 45(1):2-14	non pertinent
6540	Arntz et al.,	2007	J of behavior therapy and experimental psychiatry. 2007; 38(4):345-370	non pertinent
6541	Apte et al.,	2007	Ophthalmology. 2007; 114(9):1702-1712	non pertinent
6542	Anderson et al.,	2007	Neuroreport. 2007; 18(13):1351-1355	non pertinent

6543	Akcaboy et al.,	2007	Saudi medical j. 2007; 28(10):1550-1554	non pertinent
6544	Aidar et al.,	2007	Revista de neurologia. 2007; 45(9):518-522	non pertinent
6545	- No authors listed	2007	Drugs in r and d. 2007; 8(1):61-68	non pertinent
6546	Zubieta et al.,	2006	Brain Behav Immun. 2006; 20(1):15-26	non pertinent
6547	Zohlnhöfer et al.,	2006	Jama. 2006; 295(9):1003-1010	non pertinent
6548	Zhou et al.,	2006	Respirology (carlton, vic.). 2006; 11(5):603-610	non pertinent
6549	Zepp et al.,	2006	J of pediatrics. 2006; 149(5);603-610	non pertinent
6550	Yuen et al.,	2006	Antiviral therapy. 2006; 11(8):977-983	non pertinent
6551	Yakhno et al.,	2006	Clinical drug investigation. 2006; 26(5):267-277	non pertinent
6552	Xiong et al.,	2006	Brazilian j of medical and biological research = revista brasileira de pesquisas medicas e biologicas. 2006; 39(1):85-90	non pertinent
6553	Wu et al.,	2006	European j of clinical investigation. 2006; 36(11):803-809	non pertinent
6554	Wright et al.	2006	Neuroimage. 2006; 29(2):628-636	non pertinent
6555	Wright et al.	2006	Health technology assessment. 2006; 10(21):iii-93	non pertinent
6556	Wolfs et al.,	2006	BMC musculoskeletal disorders. 2006; 7:14	non pertinent
6557	Witting et al.,	2006	Pain. 2006; 120(1-2):145-154	non pertinent
6558	Winner et al.,	2006	Headache. 2006; 46(2):212-222	non pertinent
6559	Winner et al.,	2006	Clinical therapeutics. 2006; 28(10):1582-1591	non pertinent
6560	Winhusen et al.,	2006	Pharmacology, biochemistry, and behavior. 2006; 85(1):29-38	non pertinent
6561	Williams et al.,	2006	Neuroimage. 2006; 29(2):347-357	non pertinent

6562	Williams et al.,	2006	J Neurosci. 2006; 26(36):9264-9271	non pertinent
6563	Williams et al.,	2006	Health technology assessment (Winchester, England). 2006; 10(40):iii-iv, ix-x, 1-195	non pertinent
6564	Wilens et al.,	2006	Archives of pediatrics & adolescent medicine. 2006; 160(1):82-90	non pertinent
6565	Wilder-Smith et al.,	2006	Scandinavian j of gastroenterology. 2006; 41(3):264-273	non pertinent
6566	Wik et al.,	2006	Int J Neurosci. 2006; 116(1):1-8	non pertinent
6567	White et al.,	2006	Anesth Analg. 2006; 103(1):76-80	non pertinent
6568	Weisler et al.,	2006	CNS spectrums. 2006; 11(8):625-639	non pertinent
6569	Warpechowski et al.,	2006	Pacing and clinical electrophysiology : PACE. 2006; 29(12):1375-1382	non pertinent
6570	Wang et al.,	2006	J of the chinese medical association. 2006; 69(10):453-460	non pertinent
6571	Walsh et al.,	2006	Sleep medicine. 2006; 7(2):155-161	non pertinent
6572	Wallace et al.,	2006	Neuromodulation. 2006; 9(2):75-86	non pertinent
6573	Wagner et al.,	2006	J of the American Academy of Child and Adolescent Psychiatry. 2006; 45(3):280-288	non pertinent
6574	Viola et al.,	2006	J of diabetes and ist complications. 2006; 20(1):34-39	non pertinent
6575	Vinciguerra et al.,	2006	Angiology. 2006; 57(3):331-339	non pertinent
6576	Veldhuijzen et al.,	2006	Psychopharmacology (Berl). 2006; 183(4):462-470	non pertinent
6577	Vejdani et al.,	2006	Digestive diseases and sciences. 2006; 51(8):1501-1507	non pertinent
6578	VanNatta et al.,	2006	American j of gastroenterology. 2006; 101(10):2209-2217	non pertinent
6579	Van der Heijde et al.,	2006	Arthritis and rheumatism. 2006; 54(4):1063-	non pertinent

6580	Van Aart et al.,	2006	Thrombosis research. 2006; 118(3):313-320	non pertinent
6581	Valovirta et al.,	2006	Allergy. 2006; 61(10):1177-1183	non pertinent
6582	Valjus et al.,	2006	Acta Anaesthesiol Scand. 2006; 50(1):32-39	non pertinent
6583	Vaideanu et al.,	2006	British j of ophthalmology. 2006; 90(6):713-717	non pertinent
6584	Usui et al.,	2006	Pain. 2006; 121(3):276-280	non pertinent
6585	Ustün et al.,	2006	J of oral and maxillofacial surgery. 2006; 64(9):1353-1358	non pertinent
6586	Uhl et al.,	2006	Clinical toxicology (Philadelphia, Pa.). 2006; 44 Suppl 1:17-28	non pertinent
6587	Tuchman et al.,	2006	CNS Drugs. 2006; 20(12):1019-1026	non pertinent
6588	Tu et al.,	2006	Gastrointestinal endoscopy. 2006; 63(1):87-94	non pertinent
6589	Torrabadella et al.,	2006	Diseases of the colon and rectum. 2006; 49(6):865-868	non pertinent
6590	Tomida et al.,	2006	J of glaucoma. 2006; 15(5):349-353	non pertinent
6591	Tohda et al.,	2006	J of asthma. 2006; 43(8):613-618	non pertinent
6592	Titta et al.,	2006	J of sexual medicine. 2006; 3(2):267-273	non pertinent
6593	Thurnher et al.,	2006	Neuroradiology. 2006; 48(11):795-801	non pertinent
6594	Thuluvath et al.,	2006	Alimentary pharmacology & therapeutics. 2006; 24(6):973-982	non pertinent
6595	Thom	2006	J of international medical research. 2006; 34(5):514-519	non pertinent
6596	Tfelt-Hansen et al.,	2006	J of headache and pain. 2006; 7(6):389-394	non pertinent
6597	Teppo et al.,	2006	Clinical otolaryngology. 2006; 31(4):303-309	non pertinent
6598	Taylor et al.,	2006	Retrovirology. 2006; 3:63	non pertinent
6599	Tauchmanovà et al.,	2006	Bone marrow transplantation. 2006; 37(1):81-88	non pertinent

6600	Tariot et al.,	2006	American j of geriatric psychiatry. 2006; 14(9):767-776	non pertinent
6601	Tan et al.,	2006	J of rehabilitation research and development. 2006; 43(4):461-474	non pertinent
6602	Takamatsu et al.,	2006	Br J Anaesth. 2006; 96(5):620-626	non pertinent
6603	Tak et al.,	2006	Child. 2006; 32(3):257-268	non pertinent
6604	Symonds et al.,	2006	J Neurophysiol. 2006; 95(6):3823-3830	non pertinent
6605	Swanson et al.,	2006	J of clinical psychiatry. 2006; 67(1):137-147	non pertinent
6606	Sunshine et al.,	2006	Clinical therapeutics. 2006; 28(8):1107-1115	non pertinent
6607	Stonell et al.,	2006	Anaesthesia. 2006; 61(3):240-247	non pertinent
6608	Stewart et al.,	2006	Diabetic medicine. 2006; 23(10):1069-1078	non pertinent
6609	Steele et al.,	2006	J canadien de pharmacologie clinique (Canadian j of clinical pharmacology). 2006; 13(1):e50-62	non pertinent
6610	Stark et al.,	2006	Neuroimage. 2006; 32(3):1290-1298	non pertinent
6611	Stan et al.,	2006	J of clinical endocrinology and metabolism. 2006; 91(12):4817-4824	non pertinent
6612	Sripada et al.,	2006	BJOG. 2006; 113(7):835-838	non pertinent
6613	Springhart et al.,	2006	J Endourol. 2006; 20(10):713-716	non pertinent
6614	Sprenger et al.,	2006	Anesth Analg. 2006; 103(3):729-737	non pertinent
6615	Sprenger et al.,	2006	Pain. 2006; 122(1-2):63-67	non pertinent
6616	Spoelstra-de Man et al.,	2006	J of human hypertension. 2006; 20(8):599-611	non pertinent
6617	Spencer et al.,	2006	Clinical therapeutics. 2006; 28(3):402-418	non pertinent

6618	Soetens et al.,	2006	Anesthesia and Analgesia. 2006; 103(1):182-186	non pertinent
6619	Sobo et al.,	2006	J Holist Nurs. 2006; 24(1):51-57	non pertinent
6620	Soares et al.,	2006	Obstetrics and gynecology. 2006; 108(6):1402-1410	non pertinent
6621	Smits et al.,	2006	Consult Clin Psychol. 2006; 74(6):1203-1212	non pertinent
6622	Smits et al.,	2006	Behaviour research and therapy. 2006; 44(12):1773-1785	non pertinent
6623	Smita et al.,	2006	AIDS research and therapy. 2006; 3(1)	non pertinent
6624	Smeets et al.,	2006	BMC Musculoskelet Disord. 2006; 7:5	non pertinent
6625	Smeets et al.,	2006	J of Pain. 2006; 7(4):261-271	non pertinent
6626	Small et al.,	2006	Pain. 2006; 120(1-2):124-130	non pertinent
6627	Skiveren et al.,	2006	Acta dermato-venereologica. 2006; 86(5):409-411	non pertinent
6628	Siquier et al.,	2006	J of antimicrobial chemotherapy. 2006; 57(3):536-545	non pertinent
6629	Sinatra et al.,	2006	Reg Anesth Pain Med. 2006; 31(2):134-142	non pertinent
6630	Silva et al.,	2006	J of child and adolescent psychopharmacology. 2006; 16(3):239-251	non pertinent
6631	Silva et al.,	2006	J of hepatology. 2006; 45(2):204-213	non pertinent
6632	Silberstein et al.,	2006	Clinical therapeutics. 2006; 28(7):1002-1011	non pertinent
6633	Shirakami et al.,	2006	J of anesthesia. 2006; 20(2):78-85	non pertinent
6634	Shinefield et al.,	2006	Pediatric infectious disease j. 2006; 25(4):287-292	non pertinent
6635	Shelton et al.,	2006	J of clinical psychiatry. 2006; 67(11):1674-1681	non pertinent
6636	Sheedy et al.,	2006	J of clinical and experimental neuropsychology. 2006; 28(5):755-772	non pertinent
6637	Shao et al.,	2006	American j of surgery. 2006; 191(2):245-249	non pertinent

6638	Shafiq et al.,	2006	Pharmacology. 2006; 78(3):136-143	non pertinent
6639	Semiglazov et al.,	2006	Anticancer research. 2006; 26(2B):1519-1529	non pertinent
6640	Sciamanna et al.,	2006	Headache. 2006; 46(1):92-100	non pertinent
6641	Schwartz et al.,	2006	Contraception. 2006; 74(2):133-140	non pertinent
6642	Schuurmans et al.,	2006	American j of geriatric psychiatry. 2006; 14(3):255-263	non pertinent
6643	Schutter et al.,	2006	J of psychiatry & neuroscience. 2006; 31(2):101-104	non pertinent
6644	Schreiber et al.,	2006	Clinical gastroenterology and hepatology. 2006; 4(3):325-334	non pertinent
6645	Schmitt et al.,	2006	Nephrology, dialysis, transplantation. 2006; 21(12):3520-3524	non pertinent
6646	Schmidt-Wilcke et al.,	2006	Pain. 2006; 125(1-2):89-97	non pertinent
6647	Schlaff et al.,	2006	Fertility and sterility. 2006; 85(2):314-325	non pertinent
6648	Schifitto et al.,	2006	J of neurovirology. 2006; 12(4):328-331	non pertinent
6649	Schiano et al.,	2006	Liver transplantation. 2006; 12(9):1381-1389	non pertinent
6650	Schell	2006	J of surgical research. 2006; 134(1):124-132	non pertinent
6651	Scaccianoce et al.,	2006	Canadian j of gastroenterology. 2006; 20(2):113-117	non pertinent
6652	Santo et al.,	2006	Diabetes research and clinical practice. 2006; 72(3):231-237	non pertinent
6653	Sangal et al.,	2006	Sleep. 2006; 29(12):1573-1585	non pertinent
6654	Sami et al.,	2006	Neuroscience. 2006; 140(1):269-279	non pertinent
6655	Salk et al.,	2006	J of bone and joint surgery American volume. 2006; 88(2):295-302	non pertinent
6656	Salinas et al.,	2006	Anesthesia and Analgesia. 2006; 102(4):1234-1239	non pertinent

6657	Salim et al.,	2006	British j of dermatology. 2006; 154(6):1169-1174	non pertinent
6658	Sakamoto et al.,	2006	J of gastroenterology. 2006; 41(1):70-76	non pertinent
6659	Sahney	2006	Pediatric drugs. 2006; 8(6):357-373	non pertinent
6660	Sahin et al.,	2006	Clinical rheumatology. 2006; 25(2):143-148	non pertinent
6661	Ryan et al.,	2006	J of urology. 2006; 176(3):972-978	non pertinent
6662	Rowland et al.,	2006	J of clinical psychology in medical settings. 2006; 13(3):263-271	non pertinent
6663	Rowan et al.,	2006	Clinical therapeutics. 2006; 28(6):921-932	non pertinent
6664	Rothstein et al.,	2006	Gastroenterology. 2006; 131(3):704-712	non pertinent
6665	Rothner et al.,	2006	Headache. 2006; 46(1):101-109	non pertinent
6666	Roth et al.,	2006	Sleep medicine. 2006; 7(4):312-318	non pertinent
6667	Ross et al.,	2006	Sexually transmitted infectious. 2006; 82(6):446-451	non pertinent
6668	Ronkainen et al.,	2006	J of pediatrics. 2006; 149(2):241-247	non pertinent
6669	Romsauerova et al.,	2006	Physiol Meas. 2006; 27(5):211-219	non pertinent
6670	Rojnuckarin et al.,	2006	Transactions of the Royal Society of Tropical Medicine and Hygiene. 2006; 100(9):879-884	non pertinent
6671	Rogers et al.,	2006	Academic emergency medicine. 2006; 13(6):617-622	non pertinent
6672	Roes et al.,	2006	European j of obstetrics, gynecology, and reproductive biology. 2006; 127(1):61-67	non pertinent
6673	Roback et al.,	2006	Annals of emergency medicine. 2006; 48(5):605-612	non pertinent

6674	Righart et al.,	2006	Cereb Cortex. 2006; 16(9):1249-1257	non pertinent
6675	Riest et al.,	2006	European j of anaesthesiology. 2006; 23(3):219-226	non pertinent
6676	Reyes et al.,	2006	American j of psychiatry. 2006; 163(3):402-410	non pertinent
6677	Reuben et al.,	2006	Regional anesthesia and pain medicine. 2006; 31(1):6-13	non pertinent
6678	Reni et al.,	2006	Pancreatology. 2006; 6(5):454-463	non pertinent
6679	Renda et al.,	2006	Clinical pharmacology and therapeutics. 2006; 80(3):264-274	non pertinent
6680	Reesink et al.,	2006	Gastroenterology. 2006; 131(4):997-1002	non pertinent
6681	Reed et al.,	2006	European j of cancer (Oxford, England : 1990). 2006; 42(2):179-185	non pertinent
6682	Reddy et al.,	2006	Obstetrics and gynecology. 2006; 108(1):41-48	non pertinent
6683	Reading et al.,	2006	Clinical microbiology and infection. 2006; 12(11):1082-1088	non pertinent
6684	Raskin et al.,	2006	Pain Medicine (Malden, Mass.). 2006; 7(5):373-385	non pertinent
6685	Raffaeli et al.,	2006	European j of anaesthesiology. 2006; 23(7):605-610	non pertinent
6686	Qvistgaard et al.,	2006	Osteoarthritis and cartilage. 2006; 14(2):163-170	non pertinent
6687	Pud et al.,	2006	Pain. 2006; 120(1-2):155-160	non pertinent
6688	Primavera et al.,	2006	Cutaneous and ocular toxicology. 2006; 25(3):165-171	non pertinent
6689	Preutthipan et al.,	2006	Fertility and sterility. 2006; 86(4):990-994	non pertinent
6690	Pratley et al.,	2006	Hormon- und Stoffwechselforschung / Hormones et metabolisme (Hormone and metabolic research). 2006; 38(6):423-428	non pertinent

6691	Prasad et al.,	2006	Heart (British Cardiac Surgery). 2006; 92(6):798-803	non pertinent
6692	Powles et al.,	2006	Breast cancer research. 2006; 8(2):R13	non pertinent
6693	Poston et al.,	2006	J of internal medicine. 2006; 260(4):388-398	non pertinent
6694	Ploner et al.,	2006	Cereb Cortey. 2006; 16(4):537-540	non pertinent
6695	Pilatti et al.,	2006	J of periodontology. 2006; 77(11):1809-1814	non pertinent
6696	Pignata et al.,	2006	BMC Cancer. 2006; 6:202	non pertinent
6697	Philip et al.,	2006	BJU international. 2006; 97(2):263-265	non pertinent
6698	Pessoa et al.,	2006	Cereb Cortex. 2006; 16(3):366-375	non pertinent
6699	Pendleton et al.,	2006	J of urology. 2006; 176(4 Pt 1):1372-1375	non pertinent
6700	Peeters et al.,	2006	Anesthesiology. 2006; 105(6):1135-1146	non pertinent
6701	Patkar et al.,	2006	J of clinical psychopharmacology. 2006; 26(6):653-656	non pertinent
6702	Patil et al.,	2006	J of hand surgery (edinburgh, scotland). 2006; 31(6):683-686	non pertinent
6703	Patel et al.,	2006	International j of clincial practice. 2006; 60(8):914-921	non pertinent
6704	Patel et al.,	2006	J of the American Academy of Dermatology. 2006; 54(6):1025-1032	non pertinent
6705	Paspatis et al.,	2006	J of pediatric gastroenterology and nutrition. 2006; 43(2):195-199	non pertinent
6706	Parlak et al.,	2006	Academic emergency medicine. 2006; 13(5):493-499	non pertinent
6707	Pareek et al.,	2006	Current medical research and opinion. 2006; 22(5):977-988	non pertinent
6708	Paick et al.,	2006	Prostata cancer and prostatic diseases. 2006; 9(3):261-265	non pertinent

6709	Ozkose et al.,	2006	Tohoku j of experimental medicine. 2006; 210(2):153-160	non pertinent
6710	Osmanagaoglu et al.,	2006	Climacteric. 2006; 9(6):464-472	non pertinent
6711	Oliver et al.,	2006	Hypertension (dallas, tex. : 1979). 2006; 48(4):622-627	non pertinent
6712	Ochsner et al.,	2006	Pain. 2006; 120(1-2):69-77	non pertinent
6713	O'Shaughnessy et al.,	2006	Clinical breast cancer. 2006; 6(6):505-510	non pertinent
6714	Nuñez et al.,	2006	Osteoarthritis and cartilage. 2006; 14(3):279-285	non pertinent
6715	Ntr - Hogenhuis	2006	Netherlands Trial Register. 2006	non pertinent
6716	Nitti et al.,	2006	BJU international. 2006; 97(6):1262-1266	non pertinent
6717	Nikkola et al.,	2006	Acta obstetrica ET gynecologica scandinavica. 2006; 85(2):188-194	non pertinent
6718	Newcorn et al.,	2006	Pediatrics. 2006; 118(6):e1701-1706	non pertinent
6719	Neumanitis et al.,	2006	J of clinical oncology. 2006; 24(29):4721-4730	non pertinent
6720	Nct - Rosin	2006	National Library of Medicine. 2006	non pertinent
6721	Nct – National Institutes of Health Clinical Center	2006	National Library of Medicine. 2006	non pertinent
6722	Nct – Gan	2006	National Library of Medicine. 2006	non pertinent
6723	Nct – Heilig	2006	National Library of Medicine. 2006	non pertinent
6724	Nct – Pomeroy	2006	National Library of Medicine. 2006	non pertinent
6725	Nct – Borckardt	2006	National Library of Medicine. 2006	non pertinent
6726	Nct – Paulus	2006	National Library of Medicine. 2006	non pertinent
6727	Nct – Garg	2006	National Library of Medicine. 2006	non pertinent
6728	Nct - Edinger	2006	National Library of Medicine. 2006	non pertinent
6729	Nct – Mirski	2006	National Library of Medicine. 2006	non pertinent
6730	Nakatsuka et al.,	2006	Canadian j of anesthesia. 2006; 53(11):1103-1110	non pertinent
6731	Nakahara et al.,	2006	International j of eating disorders. 2006; 39(1):20-26	non pertinent

6732	Naja et al.,	2006	Regional anesthesia and pain medicine. 2006; 31(3):196-201	non pertinent
6733	Muzii et al.,	2006	Fertility and sterility. 2006; 85(3):689-693	non pertinent
6734	Munizza et al.,	2006	Current medical research and opinion. 2006; 22(9):1703-1713	non pertinent
6735	Mulgaonkar et al.,	2006	American j of transplantation. 2006; 6(8):1848-1857	non pertinent
6736	Mukherjee	2006	Thrombosis and haematosis. 2006; 96(4):407-412	non pertinent
6737	Muellejans et al.,	2006	Critical care (london, england). 2006; 10(3):R91	non pertinent
6738	Muehlbacher et al.,	2006	Clinical J of Pain. 2006; 22(6):526-531	non pertinent
6739	Movafegh et al.,	2006	Anesthesia and Analgesia. 2006; 102(1):263-267	non pertinent
6740	Moulton et al.,	2006	Am J Physiol Regul Integr Comp Physiol. 2006; 291(2):R257-267	non pertinent
6741	Moulin et al.,	2006	Diabetes care. 2006; 29(3):515-520	non pertinent
6742	Moskowitz et al.,	2006	Osteoarthritis and cartilage. 2006; 14(11):1111-1118	non pertinent
6743	Morley et al.,	2006	Addiction (Abingdon, England). 2006; 101(10):1451-1462	non pertinent
6744	Morgan et al.,	2006	J of bone and joint surgery. American volume. 2006; 88(12):2606-2612	non pertinent
6745	Moratti et al.,	2006	Psychophysiology. 2006; 43(2):216-226	non pertinent
6746	Morales et al.,	2006	J of the American Geriatrics Society. 2006; 54(1):70-76	non pertinent
6747	Montaner et al.,	2006	Medgenmed: medscape general medicine. 2006; 8(2):36	non pertinent
6748	Mistraletti et al.,	2006	Regional anesthesia and pain medicine. 2006; 31(3):260-269	non pertinent

6749	Misbahuddin et al.,	2006	Clinical toxicology (Philadephia, Pa.). 2006; 44(2):135-141	non pertinent
6750	Miner et al.,	2006	Clinical therapeutics. 2006; 28(5):725-733	non pertinent
6751	Miller et al.,	2006	Pain Physician. 2006; 9(2):115-121	non pertinent
6752	Mikkelsen et al.,	2006	Acta anaesthesiologica Scandinavica. 2006; 50(7):809-815	non pertinent
6753	Michalská et al.,	2006	J of clinical endocrinology and metabolism. 2006; 91(3):870-877	non pertinent
6754	Michael Hill et al.,	2006	Clinical therapeutics. 2006; 28(9):1279-1295	non pertinent
6755	Meisel et al.,	2006	European spine j. 2006; 15 Suppl 3:397-405	non pertinent
6756	McQuellon et al.,	2006	Gynecologic oncology. 2006; 101(2):296-304	non pertinent
6757	McLaughlin et al.,	2006	American j of respiratory and critical care medicine. 2006; 174(11):1257-1263	non pertinent
6758	McCutcheon et al.,	2006	Anesthesia and Analgesia. 2006; 102(3):668-675	non pertinent
6759	McCall et al.,	2006	Current medical research and opinion. 2006; 22(9):1633-1642	non pertinent
6760	Mazzola et al.,	2006	Cereb Cortex. 2006; 16(7):960-968	non pertinent
6761	Mayhew et al.,	2006	Clin Neurophysiol. 2006; 117(6):1331-1344	non pertinent
6762	Mayer et al.,	2006	Anesthesia and Analgesia. 2006; 102(2):400-404	non pertinent
6763	May et al.,	2006	J Neurosci. 2006; 26(13):3589-3593	non pertinent
6764	Matsuhisa et al.,	2006	Helicobacter. 2006; 11(3):152-158	non pertinent
6765	Mathieu et al.,	2006	J canadien d’anesthesie (Canadian j of anaesthesia). 2006; 53(1):60-66	non pertinent

6766	Masuda et al.,	2006	Clin Biomech (Bristol, Avon). 2006; 21(3):245-253	non pertinent
6767	Massad et al.,	2006	Saudi medical j. 2006; 27(7):997-1000	non pertinent
6768	Maruyama et al.,	2006	International j of urology. 2006; 13(10):1280-1285	non pertinent
6769	Marcus et al.,	2006	Medical science monitor. 2006; 12(1):PI1-7	non pertinent
6770	Manninen et al.,	2006	Anesthesia and Analgesia. 2006; 102(1):237-242	non pertinent
6771	Manicourt et al.,	2006	Arthritis and rheumatism. 2006; 54(10):3205-	non pertinent
6772	Manchikanti et al.,	2006	Pain Physician. 2006; 9(1):47-51	non pertinent
6773	Manchikanti et al.,	2006	Pain Physician. 2006; 9(1):57-60	non pertinent
6774	Malviya et al.,	2006	Paediatric anaesthesia. 2006; 16(5):554-559	non pertinent
6775	Maltepe et al.,	2006	Anaesthesia and intensive care. 2006; 34(3):353-357	non pertinent
6776	MacDermid et al.,	2006	BMC musculoskeletal disorders. 2006; 7:25	non pertinent
6777	Lustig et al.,	2006	International j of obesity (2005). 2006; 30(2):331-341	non pertinent
6778	Lundeberg et al.,	2006	Paediatric anaesthesia. 2006; 16(2):164-169	non pertinent
6779	Luhmann et al.,	2006	Pediatrics. 2006; 118(4):e1078-1086	non pertinent
6780	Luginbühl et al.,	2006	British j of anaesthesia. 2006; 96(3):323-329	non pertinent
6781	Lucas et al.,	2006	Brain Behav Immun. 2006; 20(1):49-56	non pertinent
6782	Lu et al.,	2006	Clinical J of Pain. 2006; 22(9):799-804	non pertinent
6783	Lloyd et al.,	2006	J of neurophysiology. 2006; 95(1):205-214	non pertinent
6784	Lin et al.,	2006	Lancet (london, england). 2006; 368(9540):991-997	non pertinent
6785	Lima et al.,	2006	J Spinal Cord Med. 2006; 29(3):191-203	non pertinent

6786	Lilleby et al.,	2006	Bone marrow transplantation. 2006; 37(11):1031-1035	non pertinent
6787	Liedman et al.,	2006	Acta obstetrica ET gynecologica scandinavica. 2006; 85(2):207-211	non pertinent
6788	Li et al.,	2006	Clinical therapeutics. 2006; 28(3):419-427	non pertinent
6789	Li et al.,	2006	Human reprodruction (Oxford, England). 2006; 21(6):1461-1466	non pertinent
6790	Leung et al.,	2006	BJOG. 2006; 113(12):1459-1464	non pertinent
6791	Lemmens et al.,	2006	Anesthesia and Analgesia. 2006; 102(2):438-442	non pertinent
6792	Lémann et al.,	2006	Gastroenterology. 2006; 130(4):1054-1061	non pertinent
6793	Laurent et al.,	2006	Pacing and clinical electrophysiology : PACE. 2006; 29(4):351-357	non pertinent
6794	Lasalvia-Prisco et al.,	2006	Medical oncology (Northwood, London, England). 2006; 23(1):91-104	non pertinent
6795	Larsen et al.,	2006	American j of clinical nutrition. 2006; 83(3):606-612	non pertinent
6796	Lam et al.,	2006	American j of psychiatry. 2006; 163(5):805-812	non pertinent
6797	Krolewiecki et al.,	2006	J of acquired immune deficiency syndromes. 2006; 41(4):425-429	non pertinent
6798	Kroenke et al.,	2006	J of clinical psychiatry. 2006; 67(1):72-80	non pertinent
6799	Kranzler et al.,	2006	J of clinical psychopharmacology. 2006; 26(1):13-20	non pertinent
6800	Konstas et al.,	2006	Ophthalmology. 2006; 113(3):446-450	non pertinent
6801	Kobashigawa et al.,	2006	J of heart and lung transplantation. 2006; 25(8):935-941	non pertinent

6802	Kleinböhl et al.,	2006	Anesthesia and Analgesia. 2006; 102(3):840-847	non pertinent
6803	Kirchner et al.,	2006	Osteoarthritis and cartilage. 2006; 14(2):154-162	non pertinent
6804	Kimura et al.,	2006	Current rheumatology reports. 2006; 8(6):480-488	non pertinent
6805	Kim et al.,	2006	Anesth Analg. 2006; 102(1):156-159	non pertinent
6806	Kim et al.,	2006	British j of anaesthesia. 2006; 97(3):414-418	non pertinent
6807	Khalil et al.,	2006	British j of anaesthesia. 2006; 96(2):171-178	non pertinent
6808	Karimi et al.,	2006	American j of hematology. 2006; 81(11):809-816	non pertinent
6809	Karges et al.,	2006	Clinical therapeutics. 2006; 28(12):2094-2101	non pertinent
6810	Karaaslan et al.,	2006	J of clinical anesthesia. 2006; 18(8):589-593	non pertinent
6811	Kanazi et al.,	2006	Acta anaesthesiologica Scandinavica. 2006; 50(2):222-227	non pertinent
6812	Kanai et al.,	2006	Headache. 2006; 46(4):577-582	non pertinent
6813	Kan et al.,	2006	Human reproduction (Oxford, England). 2006; 21(10):2606-2611	non pertinent
6814	Kallio et al.,	2006	British j of anaesthesia. 2006; 97(5):704-709	non pertinent
6815	Jones et al.,	2006	J of cancer education. 2006; 21(4):237-242	non pertinent
6816	Johnson et al.,	2006	J of human hypertension. 2006; 20(7):496-503	non pertinent
6817	Ji et al.,	2006	J of gastroenterology and hepatology. 2006; 21(9):1381-1387	non pertinent
6818	Jensen et al.,	2006	American j of gastroenterology. 2006; 101(9):1991-1999	non pertinent
6819	Jenis et al.,	2006	Spine j. 2006; 6(1):14-20	non pertinent

6820	Jadhav et al.,	2006	J of the American College of Cardiology. 2006; 48(5):956-963	non pertinent
6821	Jackson et al.,	2006	J Clin Psychopharmacol. 2006; 26(1):4-8	non pertinent
6822	Iwama et al.,	2006	International j of clinical oncology. 2006; 11(2):133-139	non pertinent
6823	Ishiyama et al.,	2006	J of clinical anesthesia. 2006; 18(3):211-215	non pertinent
6824	Inui et al.,	2006	J Neurophysiol. 2006; 96(2):775-784	non pertinent
6825	Imai et al.,	2006	Clinical and experimental nephrology. 2006; 10(1):40-54	non pertinent
6826	Hunt et al.,	2006	J Behav Ther Exp Psychiatry. 2006; 37(4):283-298	non pertinent
6827	Huh et al.,	2006	Korean j of anesthesiology. 2006; 51(1):70-75	non pertinent
6828	Huber et al.,	2006	J of clinical oncology. 2006; 24(27):4397-4404	non pertinent
6829	Hu et al.,	2006	Chinese j of clinical rehabilitation. 2006; 10(4):62-64	non pertinent
6830	Hsiao et al.,	2006	Acta anaesthesiologica Taiwanica. 2006; 44(2):93-99	non pertinent
6831	Houghton et al.,	2006	Clinical therapeutics. 2006; 28(4):569-581	non pertinent
6832	Hörfelt et al.,	2006	British j of dermatology. 2006; 155(3):608-613	non pertinent
6833	Hoogma et al.,	2006	Diabetic medicine. 2006; 23(2):141-147	non pertinent
6834	Hofmann et al.,	2006	Arch Gen Psychiatry. 2006; 63(3):298-304	non pertinent
6835	Hocker et al.,	2006	Anaesthesia. 2006; 61(8):752-757	non pertinent
6836	Hirsh et al.,	2006	Digestive diseases and sciences. 2006; 51(11):1946-1951	non pertinent
6837	Hiller et al.,	2006	Anesthesia and Analgesia. 2006; 102(5):1365-1371	non pertinent
6838	Hill et al.,	2006	Anesthesiology. 2006; 104(5):1047-1053	non pertinent

6839	Herrlinger et al.,	2006	American j of gastroenterology. 2006; 101(4):793-797	non pertinent
6840	Herranz et al.,	2006	European j of clinical pharmacology. 2006; 62(10):805-815	non pertinent
6841	Henderson et al.,	2006	American j of gastroenterology. 2006; 101(1):119-123	non pertinent
6842	Henderson et al.,	2006	Pain. 2006; 120(3):286-296	non pertinent
6843	Heiman et al.,	2006	J of psychosomatic obstetrics and gynaecology. 2006; 27(1):31-41	non pertinent
6844	Heiligenstein et al.,	2006	J of child and adolescent psychopharmacology. 2006; 16(1-2):207-217	non pertinent
6845	Hauck et al.,	2006	European j of pain (London, England). 2006; 10(8):757-765	non pertinent
6846	Hashkes et al.,	2006	Current rheumatology reports. 2006; 8(6):450-458	non pertinent
6847	Harmer et al.,	2006	Biol Psychiatry. 2006; 59(9):816-820	non pertinent
6848	Han et al.,	2006	J of alternative and complementary medicine (New York, N.Y.). 2006; 12(6):535-541	non pertinent
6849	Hall et al.,	2006	Anesth Prog. 2006; 53(2):34-42	non pertinent
6850	Habrat et al.,	2006	Psychiatria polska. 2006; 40(3):579-597	non pertinent
6851	Gutmann et al.,	2006	Paediatr Anaesth. 2006; 16(3):266-274	non pertinent
6852	Gürgan et al.,	2006	J of periodontology. 2006; 77(3):370-384	non pertinent
6853	Gupta	2006	ANZ j of surgery. 2006; 76(8):718-721	non pertinent
6854	Gupta et al.,	2006	Anesthesiology. 2006; 105(4):784-793	non pertinent
6855	Guo et al.,	2006	International j of urology. 2006; 13(6):721-727	non pertinent

6856	Guo et al.,	2006	Neural regeneration research. 2006; 1(9):859-861	non pertinent
6857	Gunduz et al.,	2006	Paediatric anaesthesia. 2006; 16(2):158-163	non pertinent
6858	Guggenberger et al.,	2006	European j of anaesthesiology. 2006; 23(10):832-840	non pertinent
6859	Grundmann et al.,	2006	Anesthesia and Analgesia. 2006; 103(1):217-222	non pertinent
6860	Greenway et al.,	2006	International j of obesity (2005). 2006; 30(12):1737-1741	non pertinent
6861	Greenhill et al.,	2006	J of the American Academy of Child and Adolescent Psychiaty. 2006; 45(7):817-823	non pertinent
6862	Greenhill et al.,	2006	J of the American Academy of Child and Adolescent Psychiatry. 2006; 45(5):503-511	non pertinent
6863	Greenblatt et al.,	2006	J Clin Pharmacol. 2006; 46(12):1469-1480	non pertinent
6864	Graziani et al.,	2006	International J of Oral and Maxillofacial Surgery. 2006; 35(3):241-246	non pertinent
6865	Gramke et al.,	2006	Anesthesia and Analgesia. 2006; 102(3):755-758	non pertinent
6866	Goldstein et al.,	2006	Current medical research and opinion. 2006; 22(9):1715-1723	non pertinent
6867	Gold et al.,	2006	Cyberpsychol Behav. 2006; 9(2):207-212	non pertinent
6868	Gold et al.,	2006	HIV medicine. 2006; 7(3):146-155	non pertinent
6869	Glueck et al.,	2006	Clinical therapeutics. 2006; 28(6):933-942	non pertinent
6870	Giri	2006	Acta clinica Belgica. 2006; 61(5):286-294	non pertinent
6871	Gille et al.,	2006	Der Anaesthesist. 2006; 55(4):414-422	non pertinent
6872	Giannini et al.,	2006	J of clinical gastroenterology. 2006; 40(6):515-520	non pertinent

6873	Gau et al.,	2006	J of child and adolescent psychopharmacology. 2006; 16(4):441-455	non pertinent
6874	Gasbarrini et al.,	2006	Digestive diseases (basel, switzerland). 2006; 24(1-2):195-200	non pertinent
6875	Gallagher et al.,	2006	Annals of emergency medicine. 2006; 48(2):150-160, 160.e151-154	non pertinent
6876	Fuchs et al.,	2006	Osteoarthritis and cartilage. 2006; 14(1):82-88	non pertinent
6877	Friedman et al.,	2006	Headache. 2006; 46(6):934-941	non pertinent
6878	Fregni et al.,	2006	Arthritis and rheumatism. 2006; 54(12):3988-	non pertinent
6879	Fregni et al.,	2006	Pain. 2006; 122(1-2):197-209	non pertinent
6880	Franke et al.,	2006	Gynecological endocrinology. 2006; 22(12):692-697	non pertinent
6881	Foss et al.,	2006	J Neurophysiol. 2006; 95(2):730-736	non pertinent
6882	Fornai et al.,	2006	Anesthesiology. 2006 104(1):152-157	non pertinent
6883	Flowers et al.,	2006	Clinical therapeutics. 2006; 28(11):1803-1811	non pertinent
6884	Ferahbas et al.,	2006	American j of therapeutics. 2006; 13(4):332-336	non pertinent
6885	Fellström et al.,	2006	Clinical transplantation. 2006; 20(6):732-739	non pertinent
6886	Feldman et al.,	2006	J of the American College of Cardiology. 2006; 48(6):1198-1205	non pertinent
6887	Feld et al.,	2006	J Clin Anesth. 2006; 18(1):24-28	non pertinent
6889	Fassoulaki et al.,	2006	European j of anaesthesiology. 2006; 23(2):136-141	non pertinent
6890	Faessel et al.,	2006	J Clin Pharmacol. 2006; 46(12):1439-1448	non pertinent
6891	Fabian et al.,	2006	J of cardiovascular electrophysiology. 2006; 17(4):391-395	non pertinent

6892	Euctr, N.L.	2006	EU Clinical Trials Register. 2006	non pertinent
6893	Ersayli et al.,	2006	Spine. 2006; 31(19):2221-2226	non pertinent
6894	Emslie et al.,	2006	J of the American Academy of Child and Adolescent Psychiatry. 2006; 45(12):1440-1455	non pertinent
6895	Ekman et al.,	2006	Arthroscopy. 2006; 22(6):635-642	non pertinent
6896	Ekman et al.,	2006	American j of sports medicine. 2006; 34(6):945-955	non pertinent
6897	Edelman et al.,	2006	Obstetrics and gynecology. 2006; 107(2 Pt 1):269-275	non pertinent
6898	Ebbens et al.,	2006	J of allergy and clinical immunology. 2006; 118(5):1149-1156	non pertinent
6899	Dunn et al.,	2006	Arthroscopy. 2006; 22(5):479-483	non pertinent
6900	Duc et al.,	2006	Eur Radiol. 2006; 16(2):473-478	non pertinent
6901	Drewes et al.,	2006	Gut. 2006; 55(5):619-629	non pertinent
6902	Drewes et al.,	2006	Exp Brain Res. 2006; 174(3):443-452	non pertinent
6903	Drescher et al.,	2006	Headache. 2006; 46(10):1487-1491	non pertinent
6904	Donnenfeld et al.,	2006	J of cataract and refractive surgery. 2006; 32(9):1474-1482	non pertinent
6905	Di Liddo et al.,	2006	Annals of emergency medicine. 2006; 48(4):433-440, 440.e431	non pertinent
6906	Demiraran et al.,	2006	Paediatric anaesthesia. 2006; 16(10):1047-1050	non pertinent
6907	De Francisco et al.,	2006	International j of clinical practice. 2006; 60(12):1687-1696	non pertinent
6908	Das Neves et al.,	2006	Revista brasileira de anestesiologica. 2006; 56(4):370-376	non pertinent
6909	Darwish et al.,	2006	Clinical therapeutics. 2006; 28(5):715-724	non pertinent
6910	Dancey et al.,	2006	Nutrition j. 2006; 5:16	non pertinent

6911	Dalton et al.,	2006	Annals of emergency medicine. 2006; 48(5):615-623	non pertinent
6912	Dahaba et al.,	2006	Anesthesia and Analgesia. 2006; 103(2):378-384	non pertinent
6913	D'Antono et al.,	2006	Can J Cardiol. 2006; 22(5):411-417	non pertinent
6914	D'Amico et al.,	2006	Ophthalmology. 2006; 113(6):992-1001.e1006	non pertinent
6915	Cyriac et al.,	2006	Expert review of medical devices. 2006; 3(6):777-786	non pertinent
6916	Cutler et al.,	2006	CNS spectrums. 2006; 11(9):691-702	non pertinent
6917	Croom et al.,	2006	American j of cancer. 2006; 5(2):123-136	non pertinent
6918	Creinin et al.,	2006	Obstetrics and gynecology. 2006; 108(5):1089-1097	non pertinent
6919	Cramer et al.,	2006	Acta Neurol Scand. 2006; 113(3):156-162	non pertinent
6920	Cosyn et al.,	2006	J of periodontology. 2006; 77(3):406-415	non pertinent
6921	Corrocher et al.,	2006	Clinical therapeutics. 2006; 28(9):1296-1302	non pertinent
6922	Cobo Sanz et al.,	2006	Nutricion hospitalaria. 2006; 21(4):547-551	non pertinent
6923	Clegg et al.,	2006	Pediatric infectiousdisease j. 2006; 25(9):761-767	non pertinent
6924	Cittadini et al.,	2006	Archives of neurology. 2006; 63(11):1537-1542	non pertinent
6925	Church et al.,	2006	Laryngoscopy. 2006; 116(4):602-606	non pertinent
6926	Chu et al.,	2006	Hong kong medical j = xianggang yi xue za zhi. 2006; 12(6):442-447	non pertinent
6927	Chong et al.,	2006	Annals of oncology: official j of the european society for medical oncology. 2006; 17(3):437-442	non pertinent
6928	Chiron et al.,	2006	J of child neurology. 2006; 21(6):496-502	non pertinent

6929	Chiarioni et al.,	2006	Gastroenterology. 2006; 130(3):657-664	non pertinent
6930	Chia et al.,	2006	Anesthesia and Analgesia. 2006; 102(1):201-208	non pertinent
6931	Chevrel et al.,	2006	J of bone and mineral research. 2006; 21(2):300-306	non pertinent
6932	Cheon et al.,	2006	J of gastroenterology and hepatology. 2006; 21(10):1590-1595	non pertinent
6933	Chen et al.,	2006	Neuroimage. 2006; 29(4):1080-1091	non pertinent
6934	Chazova et al.,	2006	Diabetes, obesity & metabolism. 2006; 8(4):456-465	non pertinent
6935	Chang et al.,	2006	Cerebrovascular diseases (basel, switzerland). 2006; 22(4):258-262	non pertinent
6936	Chang et al.,	2006	Acta anaesthesiologica Taiwanica. 2006; 44(3):153-159	non pertinent
6937	Chandra et al.,	2006	International j of clinical pharmacology and therapeutics. 2006; 44(8):358-363	non pertinent
6938	Chan et al.,	2006	International j of obstetric anesthesia. 2006; 15(3):201-205	non pertinent
6939	Chambers et al.,	2006	Oral oncology. 2006; 42(9):946-953	non pertinent
6940	Carteni et al.,	2006	Oncologist. 2006; 11(7):841-848	non pertinent
6941	Cararach et al.,	2006	European j of obstetrics, gynecology, and reproductive biology. 2006; 127(2):204-208	non pertinent
6942	Cañete et al.,	2006	Current medical research and opinion. 2006; 22(11):2131-2136	non pertinent
6943	Canbakan et al.,	2006	J of gastroenterology and hepatology. 2006; 21(4):657-663	non pertinent
6944	Buvanendran et al.,	2006	Anesthesiology. 2006; 104(3):403-410	non pertinent

6945	Busch et al.,	2006	Cephalalgia. 2006; 26(1):50-55	non pertinent
6946	Bruce et al.,	2006	Paediatric anaesthesia. 2006; 16(3):302-308	non pertinent
6947	Brass et al.,	2006	J of vascular surgery. 2006; 43(4):752-759	non pertinent
6948	Brandt et al.,	2006	Rheumatology (Oxford). 2006; 45(11):1389-1394	non pertinent
6949	Boyle et al.,	2006	Neuroimage. 2006; 31(3):1278-1283	non pertinent
6950	Boussofara et al.,	2006	Tunisie medicale. 2006; 84(7):427-431	non pertinent
6951	Borrelli et al.,	2006	Clinical gastroenterology and hepatology. 2006; 4(6):744-753	non pertinent
6952	Borghi et al.,	2006	American heart j. 2006; 152(3):470-477	non pertinent
6953	Boone et al.,	2006	Digestive diseases and sciences. 2006; 51(6):1103-1112	non pertinent
6954	Bonifazi et al.,	2006	Psychoneuroendo crinology. 2006; 31(9):1076-1086	non pertinent
6955	Bolondi et al.,	2006	Clinical gastroenterology and hepatology. 2006; 4(11):1395-1402	non pertinent
6956	Bogels	2006	Behav Res Ther. 2006; 44(8):1199-1210	non pertinent
6957	Blockmans et al.,	2006	American j of medicine. 2006; 119(2):167.e123-130	non pertinent
6958	Blesa et al.,	2006	Neurologia (barcelona, spain). 2006; 21(6):289-296	non pertinent
6959	Blay et al.,	2006	Regional anesthesia and pain medicine. 2006; 31(2):127-133	non pertinent
6960	Black et al.,	2006	Sleep. 2006; 29(7):939-946	non pertinent
6961	Bjørnestad et al.,	2006	Acta anaesthesiologica Scandinavica. 2006; 50(3):358-363	non pertinent
6962	Bingham et al.,	2006	Arthritis and rheumatism. 2006; 54(11):3494-	non pertinent
6963	Bingel et al.,	2006	Pain. 2006; 120(1-2): 8-15	non pertinent

6964	Bilotta et al.,	2006	Paediatr Anaesth. 2006; 16(12):1232-1237	non pertinent
6965	Bijur et al.,	2006	J of Pain. 2006; 7(2):75-81	non pertinent
6966	Biederman et al.,	2006	Clinical therapeutics. 2006; 28(2):280-293	non pertinent
6967	Bhatnagar et al.,	2006	Anaesthesia and intensive care. 2006; 34(5):586-591	non pertinent
6968	Bettucci et al.,	2006	J of headache and pain. 2006; 7(1):34-36	non pertinent
6969	Bergasa et al.,	2006	Hepatology (baltimore, md.). 2006; 44(5):1317-1323	non pertinent
6970	Benedetti et al.,	2006	Pain. 2006; 121(1-2):133-144	non pertinent
6971	Belani et al.,	2006	Lung cancer (Amsterdam, Netherlands). 2006; 53(2):231-239	non pertinent
6972	Bartocci et al.,	2006	Pain. 2006; 122(1-2):109-117	non pertinent
6973	Barroso et al.,	2006	Brazilian j of medical and biological research = revista brasileira de pesquisas medicas e biologicas. 2006; 39(9):1241-1247	non pertinent
6974	Barrio et al.,	2006	J of immunotherapy. 2006; 29(4):444-454	non pertinent
6975	Barnes et al.,	2006	Planta medica. 2006; 72(4):378-382	non pertinent
6976	Barkhuizen et al.,	2006	J of rheumatology. 2006; 33(9):1805-1812	non pertinent
6977	Ballas et al.,	2006	Health and quality of life outcomes. 2006; 4:59	non pertinent
6978	Axelsson et al.,	2006	J canadien d'anesthesie (Canadian j of anaesthesia). 2006; 53(4):370-374	non pertinent
6979	Armingeat et al.,	2006	Osteoporosis international. 2006; 17(11):1659-1665	non pertinent

6980	Arikan et al.,	2006	J of otolaryngology. 2006; 35(3):167-172	non pertinent
6981	Arber et al.,	2006	Gut. 2006; 55(3):367-373	non pertinent
6982	Antila et al.,	2006	Paediatric anaesthesia. 2006; 16(5):548-553	non pertinent
6983	Anand et al.,	2006	American j of therapeutics. 2006; 13(3):183-187	non pertinent
6984	Alvarez-Soria et al.,	2006	Annals of the rheumatic diseases. 2006; 65(8):998-1005	non pertinent
6985	Alhashemi et al.,	2006	J canadien d'anesthesie (Canadian j of anaesthesia). 2006; 53(5):449-455	non pertinent
6986	Alhashemi et al.,	2006	British j of anaesthesia. 2006; 96(6):790-795	non pertinent
6987	Alessandri et al.,	2006	Acta obstetricia ET gynecologica scandinavica. 2006; 85(7):844-849	non pertinent
6988	Alberts et al.,	2006	American j of therapeutics. 2006; 13(4):291-297	non pertinent
6989	Aiello et al.,	2006	Investigative ophthalmology & visual science. 2006; 47(1):86-92	non pertinent
6990	Ahrendt et al.,	2006	Contraception. 2006; 74(6):451-457	non pertinent
6991	Ahles et al.,	2006	Annals of family medicine. 2006; 4(4):341-350	non pertinent
6992	Agren et al.,	2006	Wound repair and regeneration. 2006; 14(5):526-535	non pertinent
6993	Agrawal et al.,	2006	J of the American Geriatrics Society. 2006; 54(5):790-795	non pertinent
6994	Aftanas et al.,	2006	Neurosci Behav Physiol. 2006; 36(2):119-130	non pertinent
6995	Actrn	2006	Australian New Zealand Clinical Trials Registry. 2006	non pertinent
6996	Buckley et al.,	2006	Annals of nutrition and metabolism. 2006; 50(2):108-114	non pertinent

6997	Multicentre Australian Colorectal-neoplasia Screening (MACS) Group	2006	Medical j of Australia. 2006; 184(11):546-550	non pertinent
6998	Zubieta et al.,	2005	J of neuroscience. 2005; 25(34):7754-7762	non pertinent
6999	Ziaei et al.,	2005	BJOG. 2005; 112(4):466-469	non pertinent
7000	Zhou et al.,	2005	Chinese j of integrative medicine. 2005; 11(1):60-64	non pertinent
7001	Zhan et al.,	2005	European radiology. 2005; 15(8):1752-1756	non pertinent
7002	Zein	2005	J of hepatology. 2005; 42(3):315-322	non pertinent
7003	Zein et al.,	2005	Hepatology. 2005; (baltimore, md.). 2005; 42(4):762-771	non pertinent
7004	Zautra et al.,	2005	Clinical J of Pain. 2005; 21(6):471-477	non pertinent
7005	Zajicek et al.,	2005	J of neurology, neurosurgery, and psychiatry. 2005; 76(12):1664-1669	non pertinent
7006	Yousefi et al.,	2005	J of family practice. 2005; 54(12):1094-1095	non pertinent
7007	Yonkers et al.,	2005	Obstetrics and gynecology. 2005; 106(3):492-501	non pertinent
7008	Yogendran et al.,	2005	Can J Anaesth. 2005; 52(1):45-49	non pertinent
7009	Yin et al.,	2005	Clinical nutrition (edinburgh, scotland). 2005; 24(6):988-997	non pertinent
7010	Yardeni et al.,	2005	Endoscopy. 2005; 37(12):1205-1210	non pertinent
7011	Wu et al.,	2005	Chinese j of integrative medicine. 2005; 11(3):173-178	non pertinent
7012	Wright et al.,	2005	J of viral hepatitis. 2005; 12(1):58-66	non pertinent
7013	Wolff et al.,	2005	British j of dermatology. 2005; 152(6):1296-1303	non pertinent
7014	Winther et al.,	2005	Scandinavian j of rheumatology. 2005; 34(4):302-308	non pertinent
7015	Winner et al.,	2005	Headache. 2005; 45(10):1304-1312	non pertinent
7016	Win et al.,	2005	Anesthesia and Analgesia. 2005; 101(1):97-102	non pertinent

7017	Wildgruber et al.,	2005	Neuroimage. 2005; 24(4):1233-1241	non pertinent
7018	Wilder-Smith et al.,	2005	Anesthesiology. 2005; 103(3):619-628	non pertinent
7019	Wiegel et al.,	2005	Acta Anaesthesiol Scand. 2005; 49(4):552-557	non pertinent
7020	Wiech et al.,	2005	Neuroimage. 2005; 27(1):59-69	non pertinent
7021	Whitlow et al.,	2005	Gynecological surgery. 2005; 2(1):5-6	non pertinent
7022	Wheeler et al.,	2005	J Neurosurg Anesthesiol. 2005; 17(2):86-90	non pertinent
7023	Wémeau et al.,	2005	J of clinical endocrinology and metabolism. 2005; 90(2):841-848	non pertinent
7024	Weklser et al.,	2005	J of anesthesia. 2005; 19(1):36-39	non pertinent
7025	Weiss et al.,	2005	J of the American Academy of Child and Adolescent Psychiatry. 2005; 44(7):647-655	non pertinent
7026	Watson et al.,	2005	Ambulatory surgery. 2005; 12(1):35-38	non pertinent
7027	Wassilew	2005	J of the european academy of dermatology and venereology : JEADV. 2005; 19(1):47-55	non pertinent
7028	Warshaw et al.,	2005	J of the American Academy of Dermatology. 2005; 53(4):578-584	non pertinent
7029	Vranken et al.,	2005	Pain. 2005; 118(1-2):224-231	non pertinent
7030	Volmanen et al.,	2005	Acta anaesthesiologica Scandinavica. 2005; 49(4):453-458	non pertinent
7031	Vladyka et al.,	2005	J Neurosurg. 2005; 102 Suppl:214-219	non pertinent
7032	Versiani et al.,	2005	CNS Drugs. 2005; 19(2):137-146	non pertinent
7033	Vergara et al.,	2005	European j of pediatrics. 2005; 164(6):377-382	non pertinent
7034	Vercellini et al.,	2005	Fertility and sterility. 2005; 84(5):1375-1387	non pertinent

7035	Van Marle et al.,	2005	International j of clinical pharmacology and therapeutics. 2005; 43(6):282-293	non pertinent
7036	Van Ganse et al.,	2005	Pharmacoepidemiology and drug safety. 2005; 14(4):249-255	non pertinent
7037	Van der Post et al.,	2005	Br J Clin Pharmacol. 2005; 60(2):128-136	non pertinent
7038	Usher et al.,	2005	Paediatr Anaesth. 2005; 15(1):23-28	non pertinent
7039	Uher et al.,	2005	Biol Psychiatry. 2005; 58(12):990-997	non pertinent
7040	Tyrell et al.,	2005	Radiotherapy and oncology. 2005; 76(1):4-10	non pertinent
7041	Turner et al.,	2005	Psychopharmacology. 2005; 178(2-3):286-295	non pertinent
7042	Turker et al.,	2005	J of cardiothoracic and vascular anesthesia. 2005; 19(4):468-474	non pertinent
7043	Tsui et al.,	2005	Paediatr Anaesth. 2005; 15(5):397-401	non pertinent
7044	Travis et al.,	2005	Inflammatory bowel diseases. 2005; 11(8):713-719	non pertinent
7045	Torquati et al.,	2005	Neuroimage. 2005; 26(1):48-56	non pertinent
7046	Toplak et al.,	2005	Diabetes, obesity & metabolism. 2005; 7(6):699-708	non pertinent
7047	Tong et al.,	2005	J of nephrology. 2005; 18(2):204-208	non pertinent
7048	Thome et al.,	2005	J Neurosurg Spine. 2005; 3(2):129-141	non pertinent
7049	Thatcher et al.,	2005	Lancet (london, england). 2005; 366(9496):1527-1537	non pertinent
7050	Taylor et al.,	2005	Academic emergency medicine. 2005	non pertinent
7051	Taskaynatan et al.,	2005	Tohoku j of experimental medicine. 2005; 205(1):19-25	non pertinent
7052	Tascioglu et al.,	2005	Rheumatology international. 2005; 26(1):21-29	non pertinent

7053	Tariq et al.,	2005	JPMA. The j of the pakistan medical association. 2005; 55(5):202-204	non pertinent
7054	Tariot et al.,	2005	American j of geriatric psychiatry. 2005; 13(11):942-949	non pertinent
7055	Tant et al.,	2005	Current therapeutic research – clinical and experimental. 2005; 66(6):511-521	non pertinent
7056	Track et al.,	2005	Gut. 2005; 54(12):1707-1713	non pertinent
7057	Tabbert et al.,	2005	Int J Psychophysiol. 2005; 57(1):15-23	non pertinent
7058	Sykora et al.,	2005	J of clinical gastroenterology. 2005; 39(8):692-698	non pertinent
7059	Swanson et al.,	2005	Treatments in respiratory medicine. 2005; 4(1):31-39	non pertinent
7060	Sundarathiti et al.,	2005	Chotmaihet thangphaet (J of the Medical Association of Thailand). 2005; 88(4):513-520	non pertinent
7061	Sughondhabirom et al.,	2005	Psychopharmacology. 2005; 180(3):436-446	non pertinent
7062	Struys et al.,	2005	Anesthesiology. 2005; 103(4):730-743	non pertinent
7063	Stegmayr et al.,	2005	Scandinavian j of urology and nephrology. 2005; 39(6):489-497	non pertinent
7064	Staud et al.,	2005	J Pain. 2005; 6(5):323-332	non pertinent
7065	Starr et al.,	2005	J of the national medical association. 2005; 97(10 Suppl):11-16	non pertinent
7066	Stamenova et al.,	2005	European j of neurology. 2005; 12(6):453-461	non pertinent
7067	Spohr et al.,	2005	Minerva anestesiologica. 2005; 71(6):291-296	non pertinent
7068	Spencer et al.,	2005	CNS spectrums. 2005; 10(10 SUPPL 15):14-21	non pertinent
7069	Speel et al.,	2005	J of sexual medicine. 2005; 2(2):207-212	non pertinent

7070	Smeets et al.,	2005	European j of vascular and endovascular surgery. 2005;	non pertinent
7071	Sir et al.,	2005	J of clinical psychiatry. 2005; 66(10):1312-1320	non pertinent
7072	Singh et al.,	2005	Indian j of pediatrics. 2005; 72(9):741-744	non pertinent
7073	Silva et al.,	2005	J of child and adolescent psychopharmacol ogy. 2005; 15(4):637-654	non pertinent
7074	Shelley et al.,	2005	Australian & New Zealand j of obstetrics & gynaecology. 2005; 45(2):122-127	non pertinent
7075	Shaheen et al.,	2005	Hepatology (baltimore, md.). 2005; 41(3):588-594	non pertinent
7076	Seikaly et al.,	2005	J of pediatric orthopedics. 2005; 25(6):786-791	non pertinent
7077	Seftel et al.,	2005	International j of impotence research. 2005; 17(5):455-461	non pertinent
7078	Schreiber et al.,	2005	Gastroenterology. 2005; 129(3):807-818	non pertinent
7079	Schmitter et al.,	2005	J of oral and maxillofacial surgery. 2005; 63(9):1295-1303	non pertinent
7080	Schienle et al.,	2005	Int J Psychophysiol. 2005; 57(1):69-77	non pertinent
7081	Scheffer et al.,	2005	American j of psychiatry. 2005; 162(1):58-64	non pertinent
7082	Scharf et al.,	2005	Sleep. 2005; 28(6):720-727	non pertinent
7083	Sato et al.,	2005	Archives of internal medicine. 2005; 165(15):1743-1748	non pertinent
7084	Sätilä et al.,	2005	American j of physical medicine & rehabilitation. 2005; 84(5):355-365	non pertinent
7085	Sathyan et al.,	2005	Clincial pharmacokinetics. 2005; 44 Suppl 1:17-24	non pertinent
7086	Saricaoglu et al.,	2005	Anesthesia and Analgesia. 2005; 101(3):904-909	non pertinent

7087	Sandel et al.,	2005	Cancer Nurs. 2005; 28(4):301-309	non pertinent
7088	Sanchez-Borges et al.,	2005	Annals of allergy, asthma and immunology. 2005; 94(1):34-38	non pertinent
7089	Samarkandi et al.,	2005	European j of anaesthesiology. 2005; 22(3):189-196	non pertinent
7090	Sáez-Llorens et al.,	2005	Pediatric infectious disease j. 2005; 24(4):293-300	non pertinent
7091	Sacchidanand et al.,	2005	International j of infectious diseases. 2005; 9(5):251-261	non pertinent
7092	Sabatinelli et al.,	2005	Neuroimage. 2005; 24(4):1265-1270	non pertinent
7093	Ryans et al.,	2005	Rheumatology (oxford, england). 2005; 44(4):529-535	non pertinent
7094	Roy-Byrne et al.,	2005	Archives of general psychiatry. 2005; 62(3):290-298	non pertinent
7095	Rossignol et al.,	2005	Clinical gastroenterology and hepatology. 2005; 3(10):987-991	non pertinent
7096	Rosenberg et al.,	2005	Sleep medicine. 2005; 6(1):15-22	non pertinent
7097	Rosenberg et al.,	2005	British j of clinical pharmacology. 2005; 60(1):7-16	non pertinent
7098	Ronkin et al.,	2005	Obstetrics and gynecology. 2005; 105(6):1397-1404	non pertinent
7099	Rodrigues et al.,	2005	Pain. 2005; 115(1-2):5-11	non pertinent
7100	Roca et al.,	2005	J Spinal Disord Tech. 2005; 18 Suppl: 82-89	non pertinent
7101	Reynders et al.,	2005	Epilepsy Behav. 2005; 7(3):419-429	non pertinent
7102	Reid et al.,	2005	Addiction. 2005; 100 Suppl 1:(SUPPL 1):32-42	non pertinent
7103	Pukall et al.,	2005	Pain. 2005; 115(1-2):118-127	non pertinent
7104	Prantera et al.,	2005	Inflammatory bowel diseases. 2005; 11(5):421-427	non pertinent
7105	Praditsuwan et al.,	2005	Chotmaihet thangphaet (J of the Medical Association of Thailand). 2005	non pertinent

7106	Powers et al.,	2005	Pediatrics. 2005; 115(6):1666-1674	non pertinent
7107	Pourtois et al.,	2005	Neuroimage. 2005; 26(1):149-163	non pertinent
7108	Pourtois et al.,	2005	Cortex. 2005; 41(1):49-59	non pertinent
7109	Potkin et al.,	2005	J of clinical psychopharmacology. 2005; 25(4):301-310	non pertinent
7110	Posey et al.,	2005	Cancer chemotherapy and pharmacology. 2005; 56(2):182-188	non pertinent
7111	Pleger et al.,	2005	Ann Neurol. 2005 57(3):425-429	non pertinent
7112	Piola et al.,	2005	Lancet (london, england). 2005; 365(9469):1467-1473	non pertinent
7113	Phongmany et al.,	2005	Transactions of the Royal Society of Tropical Medicine and Hygiene. 2005; 99(6):451-458	non pertinent
7114	Petrovic et al.,	2005	Neuron. 2005; 46(6):957-969	non pertinent
7115	Pelham et al.,	2005	J of the American Academy of Child and Adolescent Psychiatry. 2005; 44(6):522-529	non pertinent
7116	Pauli et al.,	2005	Int J Psychophysiol. 2005; 57(1):33-41	non pertinent
7117	Paschoalini Mda et al.,	2005	Chest. 2005; 128(2):684-689	non pertinent
7118	Pariente et al.,	2005	Neuroimage. 2005; 25(4):1161-1167	non pertinent
7119	Pandey et al.,	2005	J canadien d'anesthesie (Canadian j of anaesthesia). 2005; 52(8):827-831	non pertinent
7120	Pandey et al.,	2005	J of neurosurgical anesthesiology. 2005; 17(2):65-68	non pertinent
7121	Packer et al.,	2005	J of cardiac failure. 2005; 11(1):12-20	non pertinent
7122	Overholt et al.,	2005	Gastrointestinal endoscopy. 2005; 62(4):488-498	non pertinent
7123	Omran et al.,	2005	Egyptian j of anaesthesia. 2005; 21(4):295-300	non pertinent

7124	Ohmizo et al.,	2005	J canadien d'anesthesie (Canadian j of anaesthesia). 2005; 52(6):595-599	non pertinent
7125	Oddsson et al.,	2005	Contraception. 2005; 71(3):176-182	non pertinent
7126	O'Riain et al.,	2005	Anesthesia and Analgesia. 2005; 100(1):244-249	non pertinent
7127	O'Mahony et al.,	2005	Gastroenterology. 2005; 128(3):541-551	non pertinent
7128	Niethard et al.,	2005	J of rheumatology. 2005; 32(12):2384-2392	non pertinent
7129	Nielsen et al.,	2005	Neuroimage. 2005; 27(3):520-532	non pertinent
7130	Nickel et al.,	2005	J of urology. 2005; 173(4):1252-1255	non pertinent
7131	Newton et al.,	2005	Psychopharmacology. 2005; 182(3):426-435	non pertinent
7132	Nehra et al.,	2005	J of urology. 2005; 173(6):2067-2071	non pertinent
7133	Nct – Jacobsen	2005	National Library of Medicine. 2005	non pertinent
7134	Nct – Lee	2005	National Library of Medicine. 2005	non pertinent
7135	Nct – Francisco	2005	National Library of Medicine. 2005	non pertinent
7136	Nct – Hassett	2005	National Library of Medicine. 2005	non pertinent
7137	Nct – Flood	2005	National Library of Medicine. 2005	non pertinent
7138	Nct - Shapira	2005	National Library of Medicine. 2005	non pertinent
7139	Nct - Gordon	2005	National Library of Medicine. 2005	non pertinent
7140	Nct – Zubieta	2005	National Library of Medicine. 2005	non pertinent
7141	Nct - Palfi	2005	National Library of Medicine. 2005	non pertinent
7142	Nct - Harrison	2005	National Library of Medicine. 2005	non pertinent
7143	Nct – Friedman	2005	National Library of Medicine. 2005	non pertinent
7144	Nct - Weil	2005	National Library of Medicine. 2005	non pertinent
7145	Nct - Martire	2005	National Library of Medicine. 2005	non pertinent
7146	Nct - Dumonceau	2005	National Library of Medicine. 2005	non pertinent
7147	Nct - Holtrop	2005	National Library of Medicine. 2005	non pertinent
7148	Nct – Schnitzer	2005	National Library of Medicine. 2005	non pertinent
7149	Nct – Naylor	2005	National Library of Medicine. 2005	non pertinent

7150	Navan et al.,	2005	European j of neurology. 2005; 12(1):1-8	non pertinent
7151	Naesh et al.,	2005	European j of anaesthesiology. 2005; 22(10):768-773	non pertinent
7152	Mustola et al.,	2005	Acta anaesthesiologica Scandinavica. 2005; 49(2):215-221	non pertinent
7153	Muñoz et al.,	2005	Anesthesia and Analgesia. 2005; 101(2):371-376	non pertinent
7154	Mukwaya et al.,	2005	Antimicrobial agents and chemotherapy. 2005; 49(12):4903-4910	non pertinent
7155	Muehlbacher et al.,	2005	J of clinical psychopharmacology. 2005; 25(6):580-583	non pertinent
7156	Morgan et al.,	2005	Gut. 2005; 54(5):601-607	non pertinent
7157	Morgan et al.,	2005	Gastroenterology. 2005; 128(4):882-890	non pertinent
7158	Moore et al.,	2005	International clinical psychopharmacology. 2005; 20(3):131-137	non pertinent
7159	Molimard et al.,	2005	Respiratory medicine. 2005; 99(6):770-778	non pertinent
7160	Mohammadzadeh et al.,	2005	Saudi medical j. 2005; 26(8):1231-1234	non pertinent
7161	Moen et al.,	2005	American j of cancer. 2005; 4(5):327-333	non pertinent
7162	Modic et al.,	2005	Radiology. 2005; 237(2):597-604	non pertinent
7163	Miner et al.,	2005	Academic emergency medicine. 2005; 12(3):190-196	non pertinent
7164	Mikol et al.,	2005	Multiple sclerosis (houndmills, basingstoke, england). 2005; 11(5):585-591	non pertinent
7165	Michel et al.,	2005	Arthritis and rheumatism. 2005; 52(3):779-	non pertinent
7166	Merle et al.,	2005	Annales francaises d'anesthesie ET de reanimation. 2005; 24(1):13-18	non pertinent
7167	Merenstein et al.,	2005	J of family practice. 2005; 54(2):144-151	non pertinent

7168	Ménigaux et al.,	2005	Anesthesia and Analgesia. 2005; 100(5):1394-1399	non pertinent
7169	Mehta et al.,	2005	Psychopharmacology (Berl). 2005; 180(4):654-663	non pertinent
7170	McLean	2005	J Electromyogr Kinesiol. 2005; 15(6):527-535	non pertinent
7171	McClung et al.,	2005	Archives of internal medicine. 2005; 165(15):1762-1768	non pertinent
7172	Mazières et al.,	2005	J of rheumatology. 2005; 32(8):1563-1570	non pertinent
7173	Mathew et al.,	2005	Headache. 2005; 45(4):293-307	non pertinent
7174	Martoni et al.,	2005	European j of cancer (Oxford, England : 1990); 41(1):81-92	non pertinent
7175	Marson et al.,	2005	Lancet (london, england). 2005; 365(9476):2007-2013	non pertinent
7176	Marco et al.,	2005	Academic emergency medicine. 2005; 12(4):282-288	non pertinent
7177	Mancini et al.,	2005	Neurological sciences. 2005; 26(1):26-31	non pertinent
7178	Maltoni et al.,	2005	Supportive care in cancer. 2005; 13(11):888-894	non pertinent
7179	Malkin et al.,	2005	Infection and immunity. 2005; 73(6):3677-3685	non pertinent
7180	Magnussen et al.,	2005	J of health psychology. 2005; 10(2):233-243	non pertinent
7181	Magni et al.,	2005	Neurosurg Anesthesiol. 2005; 17(3):134-138	non pertinent
7182	Madisch et al.,	2005	International j of colorectal disease. 2005; 20(4):312-316	non pertinent
7183	Ly-Pen et al.,	2005	Arthritis and rheumatism. 2005; 52(2):612-	non pertinent
7184	Lorenz et al.,	2005	Brain Behav Immun. 2005; 19(4):283-295	non pertinent
7185	Lohmander et al.,	2005	Annals of the rheumatic diseases. 2005; 64(3):449-456	non pertinent
7186	Lister et al.,	2005	American j of obstertics and gynecology. 2005; 193(4):1338-1343	non pertinent

7187	Lingford-Hughes et al.,	2005	Psychopharmacology (Berl). 2005; 180(4):595-606	non pertinent
7188	Leung et al.,	2005	J Altern Complement Med. 2005; 11(4):653-661	non pertinent
7189	Leslie et al.,	2005	Anaesthesia. 2005; 60(3):239-244	non pertinent
7190	Leniger et al.,	2005	Headache. 2005; 45(1):42-46	non pertinent
7191	Lehman et al.,	2005	Arthritis and rheumatism. 2005; 52(5):1360-	non pertinent
7192	Legrain et al.,	2005	Clin Neurophysiol. 2005; 116(9):2165-2174	non pertinent
7193	Legeby et al.,	2005	Acta anaesthesiologica Scandinavica. 2005; 49(9):1360-1366	non pertinent
7194	Lee et al.,	2005	Acta anaesthesiologica Scandinavica. 2005; 1477-1482	non pertinent
7195	Lee et al.,	2005	Progress in neuro-psychopharmacology & biological psychiatry. 2005; 29(5):639-647	non pertinent
7196	LeCheminant et al.,	2005	J of the American College of Nutrition. 2005; 24(5):347-353	non pertinent
7197	Le Loet et al.,	2005	BMC musculoskeletal disorders. 2005; 6	non pertinent
7198	Laugesen et al.,	2005	Clinical pharmacology and therapeutics. 2005; 77(4):312-323	non pertinent
7199	Lane et al.,	2005	Anaesthesia. 2005; 60(5):453-457	non pertinent
7200	Lamet et al.,	2005	Inflammatory bowel diseases. 2005; 11(7):625-630	non pertinent
7201	Lai et al.,	2005	Ophthalmology. 2005; 112(4):574-579	non pertinent
7202	Kussman et al.,	2005	J Cardiothorac Vasc Anesth. 2005; 19(3):316-321	non pertinent
7203	Kushner et al.,	2005	Obstetrics and gynecology. 2005; 106(2):227-233	non pertinent

7204	Kurihara et al.,	2005	Nephrology dialysis transplantation. 2005; 20(2):424-430	non pertinent
7205	Kurata et al.,	2005	Anesth Analg. 2005; 101(2):449-456	non pertinent
7206	Kudrow et al.,	2005	Headache. 2005; 45(9):1151-1162	non pertinent
7207	Kucukemre et al.,	2005	European j of anaesthesiology. 2005; 22(5):378-385	non pertinent
7208	Kratochvil et al.,	2005	J of the American Academy of Child and Adolescent Psychiatry. 2005; 44(9):915-924	non pertinent
7209	Kozuma et al.,	2005	Hypertension research. 2005; 28(9):711-718	non pertinent
7210	Koyama et al.,	2005	Proc Natl Acad Sci U S A. 2005; 102(36):12950-12955	non pertinent
7211	Korovessis et al.,	2005	European spine j. 2005; 14(7):630-638	non pertinent
7212	Komiyama et al.,	2005	J of orofacial pain. 2005; 19(2):119-126	non pertinent
7213	Köksal et al.,	2005	J of gastroenterology and hepatology. 2005; 20(4):637-642	non pertinent
7214	Kodama et al.,	2005	European j of clinical nutrition. 2005; 59(3):384-392	non pertinent
7215	Knight et al.,	2005	Neuroimage. 2005; 26(4):1193-1200	non pertinent
7216	Killgore et al.,	2005	Neuroreport. 2005; 16(15):1671-1675	non pertinent
7217	Kilkens et al.,	2005	Aliment Pharmacol Ther. 2005; 22(9):865-874	non pertinent
7218	Kiderman et al.,	2005	British j of general practice. 2005; 55(512):218-221	non pertinent
7219	Kekec et al.,	2005	Mount Sinal j of medicine. New York. 2005; 72(6):385-388	non pertinent
7220	Keidan et al.,	2005	J of urology. 2005; 174(4 Pt 2):1598-1600	non pertinent
7221	Katz et al.,	2005	J of Pain. 2005; 6(10):656-661	non pertinent

7222	Kato et al.,	2005	Alimentary pharmacology & therapeutics. 2005; 21 Suppl 2:27-31	non pertinent
7223	Kasper et al.,	2005	American j of geriatric psychiatry. 2005; 13(10):884-891	non pertinent
7224	Karpel et al.,	2005	Annals of pharmacotherapy. 2005; 39(12):1977-1983	non pertinent
7225	Kardash et al.,	2005	J canadien d'anesthesie (Canadian j of anaesthesia). 2005; 52(6):613-617	non pertinent
7226	Kandziora et al.,	2005	Injury. 2005; 36 Suppl 2:B27-35	non pertinent
7227	Kanai et al.,	2005	Japanese j of anesthesiology. 2005; 54(1):8-13	non pertinent
7228	Kamiji et al.,	2005	Arquivos de gastroenterologia. 2005; 42(3):167-172	non pertinent
7229	Kalisch et al.,	2005	J Cogn Neurosci. 2005; 17(6):874-883	non pertinent
7230	Kakagia et al.,	2005	Annals of plastic surgery. 2005; 55(3):258-261	non pertinent
7231	Kain et al.,	2005	Anesthesiology. 2005; 102(4):720-726	non pertinent
7232	Jünemann et al.,	2005	European urology. 2005; 48(3):478-482	non pertinent
7233	Johnstone et al.,	2005	Neuroimage. 2005; 25(4):1112-1113	non pertinent
7234	Johnston et al.,	2005	Pediatric infectious disease j. 2005; 24(3):225-232	non pertinent
7235	Jermany et al.,	2005	J of clinical pharmacology. 2005; 45(10):1172-1178	non pertinent
7236	Jean et al.,	2005	Acta anaesthesiologica Taiwanica. 2005; 43(2):73-77	non pertinent
7237	Järnerot et al.,	2005	Gastroenterology. 2005; 128(7):1805-1811	non pertinent
7238	Janssen et al.,	2005	Lancet (london, england). 2005; 365(9454):123-129	non pertinent
7239	Jalowiecki et al.,	2005	Anesthesiology. 2005; 103(2):269-273	non pertinent

7240	Jacobsohn et al.,	2005	J canadien d'anesthesie (Canadian j of anaesthesia). 2005; 52(8):848-857	non pertinent
7241	Jackson et al.,	2005	Neuroimage. 2005; 24(3):771-779	non pertinent
7242	Ivani et al.,	2005	Paediatric anaesthesia. 2005; 15(6):491-494	non pertinent
7243	Isrctn – McGregor	2005	ISRCTN registry. 2005	non pertinent
7244	Ishitani et al.,	2005	Psychiatry Clin Neurosci. 2005; 59(6):644-651	non pertinent
7245	Inoue et al.,	2005	J of anesthesia. 2005; 19(1):12-16	non pertinent
7246	Ilfeld et al.,	2005	Anesthesia and Analgesia. 2005; 100(4):1172-1178	non pertinent
7247	Iannetti et al.,	2005	Neuroimage. 2005; 28(3):708-719	non pertinent
7248	Huguenin et al.,	2005	British j of sports medicine. 2005; 39(2):84-90	non pertinent
7249	Hu et al.,	2005	Chinese j of clinical rehabilitation. 2005; 9(18):8-9	non pertinent
7250	Howard et al.,	2005	J Neurol Neurosurg Psychiatry. 2005; 76(11):1558-1564	non pertinent
7251	Hooper et al.,	2005	Climacteric. 2005; 8(3):251-262	non pertinent
7252	Home et al.,	2005	Diabetologia. 2005; 48(9):1726-1735	non pertinent
7253	Holman et al.,	2005	Arthritis and rheumatism. 2005; 52(8):2495-	non pertinent
7254	Holger et al.,	2005	American j of emergency medicine. 2005; 23(3):248-252	non pertinent
7255	Holdgate et al.,	2005	J of urology. 2005; 174(2):572-575	non pertinent
7256	Hirunwiwatkul et al.,	2005	Chotmaihet thangphaet (J of the Medical Association of Thailand). 2005; 88(5):647-650	non pertinent
7257	Hellings et al.,	2005	J of child and adolescent psychopharmacology. 2005; 15(4):682-692	non pertinent
7258	Heapy et al.,	2005	Pain. 2005; 118(3):360-368	non pertinent

7259	Hayton et al.,	2005	J of bone and joint surgery. American volume. 2005; 87(3):503-507	non pertinent
7260	Haupt et al.,	2005	International j of clinical pharmacology and therapeutics. 2005; 43(2):71-77	non pertinent
7261	Hasebe et al.,	2005	J of hypertension. 2005; 23(2):445-453	non pertinent
7262	Han et al.,	2005	Acta Anaesthesiol Scand. 2005; 49(1):85-91	non pertinent
7263	Haila et al.,	2005	Homeopathy. 2005; 94(3):175-181	non pertinent
7264	Gurbet et al.,	2005	International j of obstetric anesthesia. 2005; 14(2):121-125	non pertinent
7265	Gunusen et al.,	2005	Turk anesteziyoloji ve reanimasyon dernegi dergisi. 2005; 33(4):298-306	non pertinent
7266	Grunau et al.,	2005	Pain. 2005; 113(3):293-300	non pertinent
7267	Graziosi et al.,	2005	Human reproduction (Oxford, England). 2005; 20(8):2340-2347	non pertinent
7268	Grabska et al.,	2005	J Perinatol. 2005; 25(1):33-35	non pertinent
7269	Gotoh et al.,	2005	BJU international. 2005; 96(4):581-586	non pertinent
7270	Gori et al.,	2005	Brain Res Bull. 2005; 65(4):297-300	non pertinent
7271	Gordon et al.,	2005	Gut. 2005; 54(4):540-545	non pertinent
7272	Goodman et al.,	2005	Regional anesthesia and pain medicine. 2005; 30(2):134-139	non pertinent
7273	Gonenne et al.,	2005	Clinical gastroenterology and hepatology. 2005; 3(8):784-791	non pertinent
7274	Goldstein et al.,	2005	Pain. 2005; 116(1-2):109-118	non pertinent
7275	Goldmeier et al.,	2005	International j of STD & AIDS. 2005; 16(3):196-200	non pertinent

7276	Goel et al.,	2005	J of anaesthesiology, clinical pharmacology. 2005; 21(4):389-393	non pertinent
7277	Ginström et al.,	2005	Acta oto-laryngologica. 2005; 125(9):972-975	non pertinent
7278	Gilron et al.,	2005	Pain. 2005; 113(1-2):191-200	non pertinent
7279	Gillath et al.,	2005	Neuroimage. 2005; 28(4):835-847	non pertinent
7280	Geraets et al.,	2005	Australian j of physiotherapy. 2005; 51(2):87-94	non pertinent
7281	Gendreau et al.,	2005	J of rheumatology. 2005; 32(10):1975-1985	non pertinent
7282	Gawel et al.,	2005	Headache. 2005; 45(1):7-16	non pertinent
7283	Gallez et al.,	2005	J of neurophysiology. 2005; 94(5):3509-3515	non pertinent
7284	Gagiano et al.,	2005	Psychopharmacology. 2005; 179(3):629-636	non pertinent
7285	Gach et al.,	2005	Clinical and experimental dermatology. 2005; 30(1):14-16	non pertinent
7286	Frölich et al.,	2005	Anesthesia and Analgesia. 2005; 100(2):481-486	non pertinent
7287	Freeman et al.,	2005	Spine. 2005; 30(21):2369-2377	non pertinent
7288	Freedman et al.,	2005	Multiple sclerosis (houndmills, basingstoke, england). 2005; 11(1):41-45	non pertinent
7289	Frass et al.,	2005	Homeopathy. 2005; 94(2):75-80	non pertinent
7290	Fraser et al.,	2005	Annals of the rheumatic diseases. 2005; 64(6):859-864	non pertinent
7291	Foster et al.,	2005	J of the American Academy of Dermatology. 2005; 53(5):789-809	non pertinent
7292	Fong et al.,	2005	Anaesthesia and intensive care. 2005; 33(1):73-77	non pertinent
7293	Findling et al.,	2005	J of the American Academy of Child and Adolescent Psychiatry. 2005; 44(5):409-417	non pertinent

7294	Findling et al.,	2005	J of pediatrics. 2005; 147(3):348-354	non pertinent
7295	Fillingim et al.,	2005	Mayo clinic proceedings. 2005; 80(9):1119-1121	non pertinent
7296	Fiala et al.,	2005	Human reproduction (Oxford, England). 2005; 20(11):3072-3077	non pertinent
7297	Faulhaber et al.,	2005	International j of clinical pharmacology and therapeutics. 2005; 43(2):63-70	non pertinent
7298	Fabian et al.,	2005	Surgical infections. 2005; 6(3):269-282	non pertinent
7299	Ezzet et al.,	2005	Clinical pharmacokinetics. 2005; 44(2):211-220	non pertinent
7300	Euctr, S.E.	2005	EU Clinical Trials Register. 2005	non pertinent
7301	Esen et al.,	2005	J of oral and maxillofacial surgery. 2005; 63(4):457-463	non pertinent
7302	Engeler et al.,	2005	J of urology. 2005; 174(3):933-936	non pertinent
7303	El-Agroudy et al.,	2005	Kidney international. 2005; 67(5):2039-2045	non pertinent
7304	Egeli et al.,	2005	International j of pediatric otorhinolaryngology. 2005; 69(6):811-815	non pertinent
7305	Duda et al.,	2005	J of vascular and interventional radiology : JVIR. 2005; 16(3):331-338	non pertinent
7306	Dubey et al.,	2005	Anesthesia and Analgesia. 2005; 101(4):1060-1062	non pertinent
7307	Donofrio et al.,	2005	Clinical therapeutics. 2005; 27(9):1420-1431	non pertinent
7308	Dogra et al.,	2005	European j of pain (London, England). 2005; 9(5):543-554	non pertinent
7309	Dodick et al.,	2005	Headache. 2005; 45(4)315-324	non pertinent
7310	Dodick et al.,	2005	CNS Drugs. 2005; 19(2):125-136	non pertinent

7311	Dobrydnjov et al.,	2005	Acta anaesthesiologica Scandinavica. 2005; 49(4):538-545	non pertinent
7312	Diener et al.,	2005	Cephalalgia. 2005; 25(11):1031-1041	non pertinent
7313	Diener et al.,	2005	Cephalalgia. 2005; 25(10):776-787	non pertinent
7314	Diener	2005	Current medical research and opinion. 2005; 21(10):1603-1610	non pertinent
7315	Di Paolo et al.,	2005	International j of artificial organs. 2005; 28(10):1039-1050	non pertinent
7316	Di Blasi et al.,	2005	BMC health services research. 2005; 5	non pertinent
7317	Delaunay et al.,	2005	Regional anesthesia and pain medicine. 2005; 30(2):117-122	non pertinent
7318	deCharms et al.,	2005	Proc Natl Acad Sci U S A. 2005; 102(51):18626-18631	non pertinent
7319	De Tommaso et al.,	2005	Neurologica sciences. 2005; 26 Suppl 2:152-154	non pertinent
7320	De Tommaso et al.,	2005	Neurosci Lett. 2005; 384(1-2):150-155	non pertinent
7321	Danfors et al.,	2005	J of clinical psychopharmacology. 2005; 25(5):485-489	non pertinent
7322	Dahlöf et al.,	2005	Lancet (london, england). 2005; 366(9489):895-906	non pertinent
7323	D'Angelo et al.,	2005	European j of anaesthesiology. 2005; 22(10):774-779	non pertinent
7324	Cowan et al.,	2005	Pain medicine (Malden, Mass). 2005; 6(2):113-121	non pertinent
7325	Corbett et al.,	2005	Critical care medicine. 2005; 33(5):940-945	non pertinent
7326	Cools et al.,	2005	Psychopharmacology (Berl). 2005; 180(4):670-679	non pertinent
7327	Colonna et al.,	2005	Current medical research and opinion. 2005; 21(10):1659-1668	non pertinent

7328	Cohen et al.,	2005	Diseases of the colon and rectum. 2005; 48(6):1130-1139	non pertinent
7329	Cohen et al.,	2005	Current medical research and opinion. 2005; 21(10):1683-1692	non pertinent
7330	Ciraulo et al.,	2005	Addiction (Abingdon, England). 2005; 100 Suppl 1:12-22	non pertinent
7331	Chwalisz et al.,	2005	Human reproduction (Oxford, England). 2005; 20(4):1090-1099	non pertinent
7332	Childers et al.,	2005	Current medical research and opinion. 2005; 21(9):1485-1493	non pertinent
7333	Chia et al.,	2005	Acta anaesthesiologica Scandinavica. 2005; 49(6):865-869	non pertinent
7334	Cheng et al.,	2005	J of the chinese medical association : JCMA. 2005; 68(7):307-314	non pertinent
7335	Chen et al.,	2005	J of endourology / Endourological Society. 2005; 19(2):163-166	non pertinent
7336	Chen et al.,	2005	Acta anaesthesiologic Sandinavica. 2005; 49(4):546-551	non pertinent
7337	Chang et al.,	2005	American j of gastroenterology. 2005; 100(1):115-123	non pertinent
7338	Chan et al.,	2005	Human reproduction (Oxford, England). 2005; 20(3):829-833	non pertinent
7339	Celik et al.,	2005	J of cardiothoracic and vascular anesthesia. 2005; 19(1):67-70	non pertinent
7340	Ceballos et al.,	2005	International j of clinical pharmacology research. 2005; 25(4):175-186	non pertinent
7341	Catelin et al.,	2005	J Pain. 2005; 6(12):791-797	non pertinent
7342	Cassar et al.,	2005	British j of surgery. 2005; 92(2):159-165	non pertinent

7343	Carretie et al.,	2005	Neuroimage. 2005; 24(3):615-623	non pertinent
7344	Calvet et al.,	2005	American j of gastroenterology. 2005; 100(8):1696-1701	non pertinent
7345	Cakir et al.,	2005	Contraception. 2005; 71(5):337-342	non pertinent
7346	Bulach et al.,	2005	Br J Anaesth. 2005; 94(3):300-305	non pertinent
7347	Bryant et al.,	2005	Biol Psychiatry. 2005; 58(2):111-118	non pertinent
7348	Brooks et al.,	2005	British j of sports medicine. 2005; 39(2):75-79	non pertinent
7349	Brooks et al.,	2005	Neuroimage. 2005; 27(1):201-209	non pertinent
7350	Bretthauer et al.,	2005	Endoscopy. 2005; 37(8):706-709	non pertinent
7351	Brefel-Courbon et al.,	2005	Mov Disord. 2005; 20(12):1557-1563	non pertinent
7352	Brannan et al.,	2005	J of psychiatric research. 2005; 39(1):43-53	non pertinent
7353	Brandt et al.,	2005	American j of gastroenterology. 2005; 100(SUPPL 1):5-22	non pertinent
7354	Brandt et al.,	2005	Arthritis and rheumatism. 2005; 52(7):2015-	non pertinent
7355	Bradwejn et al.,	2005	British j of psychiatry. 2005; 187:352-359	non pertinent
7356	Bradley et al.,	2005	J of rheumatology. 2005; 32(3):417-423	non pertinent
7357	Bjorkholm et al.,	2005	Haematologica. 2005; 90(4):505-515	non pertinent
7358	Bezerra et al.,	2005	Arthritis and rheumatism. 2005; 52(10):3073-	non pertinent
7359	Bentley et al.,	2005	AANA j. 2005; 73(6):427-436	non pertinent
7360	Belza et al.,	2005	European j of clinical nutrition. 2005; 59(6):733-741	non pertinent
7361	Belani et al.,	2005	Annals of oncology: official j of the european society for medical oncology. 2005; 16(7):1069-1075	non pertinent
7362	Bekkering et al.,	2005	Physical therapy. 2005; 85(6):544-555	non pertinent

7363	Becker et al.,	2005	Academic emergency medicine. 2005; 12(4):289-295	non pertinent
7364	Beale et al.,	2005	British j of anaesthesia. 2005; 95(4):500-503	non pertinent
7365	Baughman et al.,	2005	J Clin Monit Comput. 2005; 19(3):201-205	non pertinent
7366	Banaszkiewicz et al.,	2005	J of pediatrics. 2005; 146(3):364-369	non pertinent
7367	Bailey et al.,	2005	Depress anxiety. 2005; 21(1):18-25	non pertinent
7368	Bailey et al.,	2005	Clinical therapeutics. 2005; 27(10):1548-1561	non pertinent
7369	Bahrami et al.,	2005	American j of gastroenterology. 2005; 100(4):856-861	non pertinent
7370	Baguley et al.,	2005	Otology & neurotology. 2005; 26(2):169-176	non pertinent
7371	Bachmann-Mennenga et al.,	2005	Acta anaesthesiologica Scandinavica. 2005; 49(4):532-537	non pertinent
7372	Avidan et al.,	2005	Anesthesiology. 2005; 102(2):276-284	non pertinent
7373	Atli et al.,	2005	Pain medicine (Malden, Mass.). 2005; 6(3):225-234	non pertinent
7374	Atassi et al.,	2005	Chest. 2005; 128(2):863-868	non pertinent
7375	Astruc et al.,	2005	British j of clinical pharmacology. 2005; 59(4):405-414	non pertinent
7376	Arguedas et al.,	2005	Pediatric infectious disease j. 2005; 24(2):153-161	non pertinent
7377	Aragona et al.,	2005	European j of pain (London, England). 2005; 9(1):33-38	non pertinent
7378	Antmen et al.,	2005	Paediatric anaesthesia. 2005; 15(3):214-219	non pertinent
7379	Amrhein et al.,	2005	J Anxiety Disord. 2005; 19(2):177-191	non pertinent
7380	Alpers et al.,	2005	Int J Psychophysiol. 2005; 57(1):25-32	non pertinent
7381	Alobid et al.,	2005	Allergy. 2005; 60(4):452-458	non pertinent

7382	Al-Azzawi et al.,	2005	Menopause (New York, N.Y.). 2005; 12(3):331-339	non pertinent
7383	Akin et al.,	2005	J of clinical anesthesia. 2005; 17(3):187-190	non pertinent
7384	Agarwal et al.,	2005	Anaesthesia. 2005; 60(10):978-981	non pertinent
7385	Abikoff et al.,	2005	J of the American Academy of Child and Adolescent Psychiatry. 2005; 44(5):418-427	non pertinent
7386	Yunus et al.,	2004	Arthritis Rheum. 2004; 51(4):513-518	non pertinent
7387	Yoshitani et al.,	2004	Anesth Analg. 2004; 98(2):471-476	non pertinent
7388	Yang et al.,	2004	J of the chinese medical association : JCMA. 2004; 67(7):336-343	non pertinent
7389	Wirth et al.,	2004	J of urology. 2004; 172(5 Pt 1):1865-1870	non pertinent
7390	Williams et al.,	2004	Brain Res Cogn Brain Res. 2004; 21(1):114-123	non pertinent
7391	Wigal et al.,	2004	J of the American Academy of Child and Adolescent Psychiatry. 2004; 43(11):1406-1414	non pertinent
7392	Weninger et al.,	2004	Anasthesiologie, Intensivmedizin, Notfallmedizin, Schmerztherapie. 2004; 39(4):212-219	non pertinent
7393	Wasner et al.,	2004	Brain. 2004; 127(Pt 5):1159-1171	non pertinent
7394	Wade et al.,	2004	Multiple sclerosis (houndmills, basingstoke, england). 2004; 10(4):434-441	non pertinent
7395	Vitton et al.,	2004	Human psychopharmacology. 2004; 19 Suppl 1:27-35	non pertinent
7396	Veselis et al.,	2004	Anesthesiology. 2004; 101(4):831-841	non pertinent
7397	Verstappen et al.,	2004	Annals of family medicine. 2004; 2(6):569-575	non pertinent

7398	Verri et al.,	2004	Revista portuguesa de cardiologia (Portuguese j of cardiology). 2004; 23(9):1089-1105	non pertinent
7399	Velikova et al.,	2004	J Clin Oncol. 2004; 22(4):714-724	non pertinent
7400	Van Duinen et al.,	2004	Neuro Endocrinol Lett. 2004; 25(5):386-390	non pertinent
7401	Usichenko et al.,	2004	Anesth Analg. 2004; 98(2):343-345	non pertinent
7402	Tursi et al.,	2004	Medical science monitor. 2004; 10(11):PI126-131	non pertinent
7403	Tsuchiya et al.,	2004	Chinese j of digestive diseases. 2004; 5(4):169-174	non pertinent
7404	Tomikawa et al.,	2004	J of gastroenterology and hepatology. 2004; 19(5):576-581	non pertinent
7405	Thimineur et al.,	2004	Pain. 2004; 109(3):242-249	non pertinent
7406	Thébault et al.,	2004	Pharmacotherapy. 2004; 24(10):1295-1305	non pertinent
7407	Tarantino et al.,	2004	Haemophilia. 2004; 10(5):428-437	non pertinent
7408	Tamir et al.,	2004	J Spinal Disord Tech. 2004; 17(6):467-469	non pertinent
7409	Talaie et al.,	2004	Clinical and experimental pharmacology & physiology. 2004; 31(11):777-782	non pertinent
7410	Taiji	2004	Folia pharmacologica japonica. 2004; 124(3):171-179	non pertinent
7411	Tabak et al.,	2004	J of rheumatology and medical rehabilitation. 2004; 15(4):234-240	non pertinent
7412	Szeimies et al.,	2004	J of the American Academy of Dermatology. 2004; 51(4):547-555	non pertinent
7413	Storro et al.,	2004	J of rehabilitation medicine. 2004; 36(1):12-16	non pertinent
7414	Stein et al.,	2004	American heart j. 2004; 147(4):E18	non pertinent

7415	Staretz et al.,	2004	J of periodontology. 2004; 75(10):1319-1326	non pertinent
7416	Song et al.,	2004	Anesthesiology. 2004; 100(5):1072-1075	non pertinent
7417	Song et al.,	2004	Anesth Analg. 2004; 98(3):687-691	non pertinent
7418	Skoumal et al.,	2004	European urology. 2004; 46(3):362-369	non pertinent
7419	Sinatra et al.,	2004	Anesth Analg. 2004; 98(1):135-140	non pertinent
7420	Simon et al.,	2004	J of psychiatric research. 2004; 38(5):491-495	non pertinent
7421	Shanahan et al.,	2004	Ann Rheum Dis. 2004; 63(9):1035-1040	non pertinent
7422	Schlünzen et al.,	2004	Acta anaesthesiologica Scandinavica. 2004; 48(10):1268-1276	non pertinent
7423	Sauer et al.,	2004	Reproductive biomedicine online. 2004; 9(5):487-493	non pertinent
7424	Sanchez et al.,	2004	American j of obstetrics and gynecology. 2004; 191(6):1898-1906	non pertinent
7425	Ruxrungtham et al.,	2004	J of clinical pharmacology. 2004; 44(7):793-802	non pertinent
7426	Ruf et al.,	2004	Infection. 2004; 32(4):191-198	non pertinent
7427	Roitberg et al.,	2004	Surgical neurology. 2004; 61(2):106-108	non pertinent
7428	Rogers et al.,	2004	Anesthesiology. 2004; 100(2):292-301	non pertinent
7429	Rodriguez-Rubio et al.,	2004	Scandinavian j of urology and nephrology. 2004; 38(6):477-480	non pertinent
7430	Roberts et al.,	2004	Cogn Affect Behav Neurosci. 2004; 4(3):307-316	non pertinent
7431	Rickels et al.,	2004	J of clinical psychopharmacology. 2004; 24(5):488-496	non pertinent
7432	Ribeiro et al.,	2004	J of pain and symptom management. 2004; 27(5):434-439	non pertinent

7433	Rhame et al.,	2004	Antimicrobial agents and chemotherapy. 2004; 48(11):4200-4208	non pertinent
7434	Reines et al.,	2004	Neurology. 2004; 62(1):66-71	non pertinent
7435	Rasmussen et al.,	2004	Eur J Neurol. 2004; 11(8):545-553	non pertinent
7436	Raij et al.,	2004	Neuroimage. 2004; 23(2):569-573	non pertinent
7437	Quante et al.,	2004	Arzneimittelforschung. 2004; 54(3):143-151	non pertinent
7438	Pradalier et al.,	2004	CNS Drugs. 2004; 18(15):1149-1163	non pertinent
7439	Pourtois et al.,	2004	Cereb Cortex. 2004; 14(6):619-633	non pertinent
7440	Pinnington et al.,	2004	Family practice. 2004; 21(4):372-380	non pertinent
7441	Pickering et al.,	2004	American j of hypertension. 2004; 17(12 Pt 1):1135-1142	non pertinent
7442	Petrakis et al.,	2004	Psychopharmacology. 2004; 172(3):291-297	non pertinent
7443	Peloso et al.,	2004	J of rheumatology. 2004; 31(12):2454-2463	non pertinent
7444	Pekindil et al.,	2004	Nuclear medicine communications. 2004; 25(1):29-37	non pertinent
7445	Paul et al.,	2004	Clinical therapeutics. 2004; 26(9):1508-1514	non pertinent
7446	Pande et al.,	2004	J Clin Psychopharmacol. 2004; 24(2):141-149	non pertinent
7447	Olander et al.,	2004	Clinical therapeutics. 2004; 26(10):1619-1629	non pertinent
7448	Ohara et al.,	2004	Clin Neurophysiol. 2004; 115(7):1641-1652	non pertinent
7449	Nct – National Institute of Dental and Craniofacial Research	2004	National Library of Medicine. 2004	non pertinent
7450	Narayan et al.,	2004	Oncol Rep. 2004; 11(1):97-103	non pertinent
7451	Myles et al.,	2004	Lancet. 2004; 363(9423):1757-1763	non pertinent
7452	Morris et al.,	2004	Neuroimage. 2004; 22(1):372-380	non pertinent

7453	Moretti et al.,	2004	Drugs under experimental and clinical research. 2004; 30(4):143-152	non pertinent
7454	Mohammadi et al.,	2004	J of child and adolescent psychopharmacology. 2004; 14(3):418-425	non pertinent
7455	Miceli-Richard et al.,	2004	Annals of the rheumatic diseases. 2004; 63(8):923-930	non pertinent
7456	Mencke et al.,	2004	Acta anaesthesiologica Scandinavica. 2004; 48(10):1245-1248	non pertinent
7457	Meissner et al.,	2004	Anesth Analg. 2004; 98(1):141-147	non pertinent
7458	Medina-Santillan et al.,	2004	Proceedings of the western pharmacology society. 2004; 47:109-112	non pertinent
7459	McDonald et al.,	2004	West J Nurs Res. 2004; 26(8):836-852	non pertinent
7460	Mathews et al.,	2004	J Cogn Neurosci. 2004; 16(10):11683-1694	non pertinent
7461	Malmgren et al.,	2004	Acta anaesthesiologica Scandinavica. 2004; 48(10):1277-1282	non pertinent
7462	Lossignol et al.,	2004	Revue medicale de bruxelles. 2004; 25(5):429-435	non pertinent
7463	Lonial et al.,	2004	Biology of blood and marrow transplantation. 2004; 10(12):848-857	non pertinent
7464	Linde et al.,	2004	Cephalalgia. 2004; 24(12):1057-1066	non pertinent
7465	Lee et al.,	2004	Clinical breast cancer. 2004; 5(5):371-376	non pertinent
7466	Lee et al.,	2004	J of periodontology. 2004; 75(11):1500-1508	non pertinent
7467	Launo et al.,	2004	Minerva Anestesiologica. 2004; 70(10):727-734	non pertinent
7468	Larijani et al.,	2004	Pharmacotherapy. 2004; 24(12):1675-1680	non pertinent

7469	Landis et al.,	2004	Brain Behav Immun. 2004; 18(4):304-313	non pertinent
7470	Kullenberg et al.,	2004	J of rheumatology. 2004; 31(11):2265-2268	non pertinent
7471	Kranzler et al.,	2004	Alcoholism, clinical and experimental research. 2004; 28(7):1051-1059	non pertinent
7472	Korn et al.,	2004	Arthritis and rheumatism. 2004; 50(12):3985-	non pertinent
7473	Kohno et al.,	2004	J of Clinical Oncology. 2005; 23(15):3314-21	non pertinent
7474	Koeppe et al.,	2004	Scand J Rheumatol Suppl. 2004; 119:24-27	non pertinent
7475	Knight et al.,	2004	Cogn Affect Behav Neurosci. 2004; 4(3):317-325	non pertinent
7476	Klapper et al.,	2004	Cephalalgia. 2004; 24(11):918-924	non pertinent
7477	Kiss et al.,	2004	Neurosurg Focus. 2004; 17(1):E5	non pertinent
7478	Killeen et al.,	2004	Alcoholism, clinical and experimental research. 2004; 28(11):1710-1717	non pertinent
7479	Khullar et al.,	2004	Urology. 2004; 64(2):269-274	non pertinent
7480	Khan et al.,	2004	Psychoneuroendocrinology. 2004; 29(9):1163-1171	non pertinent
7481	Kemp et al.,	2004	Neuroimage. 2004; 22(3):1084-1096	non pertinent
7482	Kapetanopoulos et al.,	2004	J of nuclear cardiology. 2004; 11(5):570-577	non pertinent
7483	Jones et al.,	2004	Worldviews Evid Based Nurs. 2004; 1 Suppl 1:13-20	non pertinent
7484	Jayaraman et al.,	2004	Eur J Appl Physiol. 2004; 93(1-2):30-38	non pertinent
7485	Jaju et al.,	2004	Indian j Physiol Pharmacol. 2004; 48(1):31-40	non pertinent
7486	Iversen et al.,	2004	J of urology. 2004; 172(5 Pt 1):1871-1876	non pertinent
7487	Hurley et al.,	2004	Spine (Phila Pa 1976). 2004; 29(20):2207-2216	non pertinent
7488	Horak et al.,	2004	Arzneimittel-forschung. 2004; 54(10):666-672	non pertinent

7489	Hopkins et al.,	2004	Clinical infectious diseases. 2004; 39(6):759-766	non pertinent
7490	Hoffman et al.,	2004	Neuroreport. 2004; 15(8):1245-1248	non pertinent
7491	Hofbauer et al.,	2004	Anesthesiology. 2004; 100(2):386-394	non pertinent
7492	Haid et al.,	2004	Spine J. 2004; 4(5):527-538	non pertinent
7493	Hackner et al.,	2004	British j of anaesthesia. 2004; 91(4):580-582	non pertinent
7494	Haas et al.,	2004	Headache. 2004; 44(10):1029-1037	non pertinent
7495	Guyer et al.,	2004	Spine j. 2004; 4(6 Suppl):252-259	non pertinent
7496	Guler et al.,	2004	J canadien d'anesthesie (Canadian j of anaesthesia). 2004; 51(7):718-722	non pertinent
7497	Greco et al.,	2004	J of General Internal Medicine. 2004; 19(8):813-818	non pertinent
7498	Grange et al.,	2004	J of rehabilitation research and development. 2004; 41(4):611-620	non pertinent
7499	Goldstein et al.,	2004	Clinical therapeutics. 2004; 26(10):1637-1643	non pertinent
7500	Girard et al.,	2004	Anesth Analg. 2004; 99(2):573-577	non pertinent
7501	Ginsberg	2004	Primary psychiatry. 2004; 11(10):27-28	non pertinent
7502	Geller et al.,	2004	J of the American Academy of Child and Adolescent Psychiatry. 2004; 43(11):1387-1396	non pertinent
7503	Gattaz et al.,	2004	Pharmacopsychiatry. 2004; 37(6):279-285	non pertinent
7504	Fritzer et al.,	2004	J Clin Neurophysiol. 2004; 21(2):99-104	non pertinent
7505	Fischer et al.,	2004	Cogn Affect Behav Neurosci. 2004; 4(3):326-334	non pertinent
7506	Finn et al.,	2004	Clinical nephrology. 2004; 62(3):193-201	non pertinent
7507	Farkas et al.,	2004	Magy Seb. 2004; 57(5):279-282	non pertinent

7508	Ericsson et al.,	2004	Drug metabolism and disposition. 2004; 32(9):923-929	non pertinent
7509	Erasmo et al.,	2004	Asian j of surgery / Asian Surgical Association. 2004; 27(3):227-235	non pertinent
7510	Epstein et al.,	2004	Vaccine. 2004; 22(13-14):1592-1603	non pertinent
7511	Emslie et al.,	2004	J of the American Academy of Child and Adolescent Psychiatry. 2004; 43(11):1397-1405	non pertinent
7512	Egeli et al.,	2004	International j of pediatric otorhinolaryngology. 2004; 68(8):1047-1051	non pertinent
7513	Drouin et al.,	2004	Diabetes, obesity & metabolism. 2004; 6(6):414-421	non pertinent
7514	Dowman	2004	Psychophysiology. 2004; 41(4):613-624	non pertinent
7515	Dowman	2004	Psychophysiology. 2004; 41(5):749-761	non pertinent
7516	Dionne et al.,	2004	J of clinical pharmacology. 2004; 44(12):1418-1424	non pertinent
7517	Diego et al.,	2004	Int J Neurosci. 2004; 114(1):31-44	non pertinent
7518	Di Lorenzo et al.,	2004	Gastroenterology. 2004; 126(1):33-40	non pertinent
7519	Derosa et al.,	2004	Diabetes, nutrition & metabolism. 2004; 17(4):222-229	non pertinent
7520	De Oliveira et al.,	2004	Pain. 2004; 110(1-2):400-408	non pertinent
7521	D'Souza et al.,	2004	Neuropsychopharmacology. 2004; 29(8):1558-1572	non pertinent
7522	Croxatto et al.,	2004	Contraception. 2004; 70(6):442-450	non pertinent
7523	Crippa et al.,	2004	Neuropsychopharmacology. 2004; 29(2):417-426	non pertinent
7524	Cicek et al.,	2004	Emergency medicine j. 2004; 21(3):323-326	non pertinent
7525	Carter et al.,	2004	J of rheumatology. 2004; 31(10):1973-1980	non pertinent
7526	Carragee et al.,	2004	Spine (Phila Pa 1976). 2004; 29(10):1112-1117	non pertinent

7527	Candell Riera et al.,	2004	Mapfre medicina. 2004; 15(2):134-140	non pertinent
7528	Bulut et al.,	2004	Clinical neurology and neurosurgery. 2004; 107(1):44-48	non pertinent
7529	Buffum et al.,	2004	J of the American Geriatrics Society. 2004; 52(7):1093-1097	non pertinent
7530	Buchbinder et al.,	2004	Ann Rheum Dis. 2004; 63(3):302-309	non pertinent
7531	Bronfort et al.,	2004	J of manipulative and physiological therapeutics. 2004; 27(8):503-508	non pertinent
7532	Brighina et al.,	2004	J of the neurological sciences. 2004; 227(1):67-71	non pertinent
7533	Boss et al.,	2004	J of shoulder and elbow surgery. 2004; 13(6):630-634	non pertinent
7534	Bookman et al.,	2004	CMAJ: Canadian Medical Association J. 2004; 171(4):333-338	non pertinent
7535	Boffito et al.,	2004	Antiviral therapy. 2004; 9(3):423-429	non pertinent
7536	Bischoff et al.,	2004	J of neurology. 2004; 251(2):219-221	non pertinent
7537	Bergendahl et al.,	2004	Acta anaesthesiologica Scandinavica. 2004; 48(10):1292-1300	non pertinent
7538	Ben-Chetrit et al.,	2004	Human reproduction (Oxford, England). 2004; 19(10):2372-2376	non pertinent
7539	Bell et al.,	2004	J Altern Complement Med. 2004; 10(2):285-299	non pertinent
7540	Bell et al.,	2004	International j of neuroscience. 2004; 114(9):1195-1220	non pertinent
7541	Beek et al.,	2004	J Thorac Cardiovasc Surg. 2004; 127(2):517-524	non pertinent
7542	Barbanoj et al.,	2004	Neuropsychobiology. 2004; 50(4):311-321	non pertinent

7543	Bajaj et al.,	2004	J of the indian medical association. 2004; 102(5):272, 274, 276-278	non pertinent
7544	Arnold et al.,	2004	J of child and adolescent psychopharmacology. 2004; 14(4):542-554	non pertinent
7545	Akcam	2004	Pediatrics international. 2004; 46(4):459-462	non pertinent
7546	Aguirre-Garay et al.,	2004	Clinical and experimental pharmacology & physiology. 2004; 31(7):462-465	non pertinent
7547	Agruss et al.,	2004	Ergonomics. 2004; 47(10):1103-1115	non pertinent
7548	Agarwala et al.,	2004	Melanoma research. 2004; 14(3):217-222	non pertinent
7549	Abratt et al.,	2004	Annals of oncology: official j of the european society for medical oncology. 2004; 15(11):1613-1621	non pertinent
7550	Zwanzger et al.,	2003	Neuropsychopharmacology. 2003; 28(5):979-984	non pertinent
7551	Zhang et al.,	2003	Brain Res. 2003; 982(2):168-178	non pertinent
7552	Zhang et al.,	2003	Magn Reson Med. 2003; 50(1):50-58	non pertinent
7553	Yetgin et al.,	2003	Leukemia. 2003; 17(2):328-333	non pertinent
7554	Yanagihara et al.,	2003	Biopharmaceutics & drug disposition. 2003; 24(1):37-43	non pertinent
7555	Winston et al.,	2003	Neuroimage. 2003; 20(1):84-97	non pertinent
7556	Willumsen et al.,	2003	Acta Odontol Scand. 2003; 61(2):93-99	non pertinent
7557	Weerakkody et al.,	2003	Somatosens Mot Res. 2003; 20(1):27-32	non pertinent
7558	Weber et al.,	2003	European j of anaesthesiology. 2003; 20(12):963-966	non pertinent
7559	Waish et al.,	2003	Prostata cancer and prostatic diseases. 2003; 6(3):242-244	non pertinent

7560	Wade et al.,	2003	J of cardiovascular pharmacology. 2003; 41(6):908-915	non pertinent
7561	Von Scheele et al.,	2003	Rheumatology (oxford, england). 2003; 42 Suppl 3:iii53-59	non pertinent
7562	Verne et al.,	2003	Pain. 2003; 105(1-2):223-230	non pertinent
7563	Veldhuyzen van Zanten et al.,	2003	J canadien de gastroenterologie (Canadian j of gastroenterology). 2003; 17(9):533-538	non pertinent
7564	Van Seventer et al.,	2003	Current medical research and opinion. 2003; 19(6):457-469	non pertinent
7565	Tuncer et al.,	2003	Anestezi dergisi. 2003; 11(2):117-120	non pertinent
7566	Taylor et al.,	2003	Antimicrobial agents and chemotherapy. 2003; 47(7):2199-2203	non pertinent
7567	Taub et al.,	2003	Physical medicine and rehabilitation clinics of North America. 2003; 14(1 SUPPL):77-91	non pertinent
7568	Stark et al.,	2003	Int J Psychophysiol. 2003; 50(3):225-234	non pertinent
7569	Smith et al.,	2003	Rheumatology. 2003; 42(12):1477-1485	non pertinent
7570	Sharp et al.,	2003	J Bone Joint Surg Br. 2003; 85(7):999-1005	non pertinent
7571	Schlereth et al.,	2003	Neuroimage. 2003; 20(1):441-454	non pertinent
7572	Schectman et al.,	2003	J Gen Intern Med. 2003; 18(10):773-780	non pertinent
7573	Scharf et al.,	2003	J Rheumatol. 2003; 30(5):1070-1074	non pertinent
7574	Sauder et al.,	2003	Acta ophthalmologica Scandinavica. 2003; 81(6):596-599	non pertinent
7575	Sarapa et al.,	2003	J of clinical pharmacology. 2003; 43(9):974-982	non pertinent

7576	Santens et al.,	2003	Acta neurologica belgica. 2003; 103(3):159-163	non pertinent
7577	Rugino et al.,	2003	Pediatric neurology. 2003; 29(2):136-142	non pertinent
7578	Rubinacci et al.,	2003	Menopause (New York, N.Y.). 2003 10(3):241-249	non pertinent
7579	Rosenwasser et al.,	2003	J of allergy and clinical immunology. 2003; 112(3):563-570	non pertinent
7580	Roifman et al.,	2003	International immunopharmacology. 2003; 3(9):1325-1333	non pertinent
7581	Ristvedt et al.,	2003	Am J Gastroenterol. 2003; 98(3):578-585	non pertinent
7582	Ringe et al.,	2003	Osteoporosis international. 2003; 14(10):801-807	non pertinent
7583	Ricci et al.,	2003	Giornale italiano di dermatologia e venereologia. 2003; 138(6):513-517	non pertinent
7584	Remy et al.,	2003	Neuroimage. 2003; 19(3):655-664	non pertinent
7585	Recart et al.,	2003	Anesth Analg. 2003; 96(4):1047-1050	non pertinent
7586	Purkins et al.,	2003	British j of clinical pharmacology. 2003; 56 Suppl 1: 62-68	non pertinent
7587	Purkins et al.,	2003	British j of clinical pharmacology. 2003; 56 Suppl 1: 10-16	non pertinent
7588	Pollo et al.,	2003	Pain. 2003; 102(1-2):125-133	non pertinent
7589	Piccart et al.,	2003	International j of gynecological cancer. 2003; 13 Suppl 2:196-203	non pertinent
7590	Phelan et al.,	2003	J of clinical psychiatry. 2003; 64(11):1328-1334	non pertinent
7591	Pfister et al.,	2003	European radiology. 2003; 13(11):2513-2520	non pertinent
7592	Petersen et al.,	2003	J of Pain. 2003; 4(7):400-406	non pertinent
7593	Paksoy et al.,	2003	Spine (Phila Pa 1976). 2003; 28(11):1183-1188	non pertinent

7594	Ostadal et al.,	2003	Molecular and cellular biochemistry. 2003; 246(1-2):45-50	non pertinent
7595	Oosterhof et al.,	2003	European urology. 2003; 44(5):519-526	non pertinent
7596	Niederhof et al.,	2003	Human psychopharmacology. 2003; 18(5):389-393	non pertinent
7597	Nemoto et al.,	2003	Neuroreport. 2003; 14(6):791-797	non pertinent
7598	Nct – Brown	2003	National Library of Medicine. 2003	non pertinent
7599	Nct – National Institute of Mental Health	2003	National Library of Medicine. 2003	non pertinent
7600	Nct –National Institutes of Health Clinical Center	2003	National Library of Medicine. 2003	non pertinent
7601	Murphy et al.,	2003	American j of cardiovascular drugs. 2003; 3(2):101-112	non pertinent
7602	Monk et al.,	2003	Neuroimage. 2003; 20(1):420-428	non pertinent
7603	Miner et al.,	2003	Acad Emerg Med. 2003; 10(6):638-643	non pertinent
7604	Miner et al.,	2003	Academic emergency medicine. 2003; 10(9):931-937	non pertinent
7605	Messeri et al.,	2003	Paediatric anaesthesia. 2003; 13(7):574-578	non pertinent
7606	Mendez et al.,	2003	Clinical colorectal cancer. 2003; 3(3):174-179	non pertinent
7607	Meletti et al.,	2003	Neurology. 2003; 60(3):426-431	non pertinent
7608	McDonald et al.,	2003	Res Nurs Health. 2003; 26(3):225-232	non pertinent
7609	McAfee et al.,	2003	Spine (Phila Pa 1976). 2003; 28(20):153-162	non pertinent
7610	Mayo et al.,	2003	Revista de la sociedad espanola del dolor. 2003; 10(7):411-418	non pertinent
7611	Martinsson et al.,	2003	Cerebrovascular diseases (basel, switzerland). 2003; 16(4):338-345	non pertinent
7612	Marchand et al.,	2003	Pain. 2003; 105(3):481-488	non pertinent

7613	Manfredi et al.,	2003	International j of geriatric psychiatry. 2003; 18(8):700-705	non pertinent
7614	Manchikanti et al.,	2003	Pain Physician. 2003; 6(3):259-267	non pertinent
7615	Luis Bautista et al.,	2003	Clinical therapeutics. 2003; 25(1):194-209	non pertinent
7616	Lorenz et al.,	2003	Brain. 2003; 126(Pt 5):1079-1091	non pertinent
7617	Loo et al.,	2003	Encephale. 2003; 29(2):165-171	non pertinent
7618	Loewen et al.,	2003	Annals of plastic surgery. 2003; 51(5):472-477	non pertinent
7619	Lin et al.,	2003	J of diabetes and its complications. 2003; 17(4):179-185	non pertinent
7620	Legrain et al.,	2003	Pain. 2003; 103(3):237-248	non pertinent
7621	Langsjo et al.,	2003	Anesthesiology. 2003; 99(3):614-623	non pertinent
7622	Kwarnström et al.,	2003	Acta anaesthesiologica Scandinavica. 2003; 47(7):868-877	non pertinent
7623	Kuiken et al.,	2003	Clinical gastroenterology and hepatology. 2003; 1(3):219-228	non pertinent
7624	Krohn et al.,	2003	European j of surgery. 2003; Supplement(588): 57-61	non pertinent
7625	Kayikcioglu et al.,	2003	European heart j. 2003; 24(22):1999-2005	non pertinent
7626	Jellish et al.,	2003	J of neurosurgical anesthesiology. 2003; 15(3):176-184	non pertinent
7627	Ind et al.,	2003	Respiratory medicine. 2003; 97(SUPPL A):9-21	non pertinent
7628	Holmes et al.,	2003	Brain Res Cogn Brain Res. 2003; 16(2):174-184	non pertinent
7629	Hoffman et al.,	2003	Cyberpsychol Behav. 2003; 6(2):127-131	non pertinent
7630	Herr et al.,	2003	J of cardiothoracic and vascular anesthesia. 2003; 17(5):576-584	non pertinent

7631	Henderson et al.,	2003	Australian & New Zealand j of obstetrics & gynaecology. 2003; 43(5):372-377	non pertinent
7632	Hayamizu et al.,	2003	Current therapeutic research – clinical and experimental. 2003; 64(8):551-567	non pertinent
7633	Hawkey et al.,	2003	Gut. 2003; 52(11):1537-1542	non pertinent
7634	Hanson et al.,	2003	Nicotine & tobacco research. 2003; 5(4):515-526	non pertinent
7635	Hackner et al.,	2003	Br J Anaesth. 2003; 91(4):580-582	non pertinent
7636	Gutteridge et al.,	2003	Calcified tissue international. 2003; 73(1):33-43	non pertinent
7637	Górecka et al.,	2003	Respiration: international review of thoracic diseases. 2003; 70(3):275-283	non pertinent
7638	Golladay et al.,	2003	Pediatric endosurgery and innovative techniques. 2003; 7(2):153-159	non pertinent
7639	Gerson et al.,	2003	Clinical gastroenterology and hepatology. 2003; 1(6):446-452	non pertinent
7640	Frerick et al.,	2003	Pain. 2003; 106(1-2):59-64	non pertinent
7641	Forestier et al.,	2003	Anesthesiology. 2003; 99(2):334-346	non pertinent
7642	Fitzgerald et al.,	2003	Eur J Anaesthesiol. 2003; 20(12):952-956	non pertinent
7643	Fernandez et al.,	2003	Dev Behav Pediatr. 2003; 24(4):261-266	non pertinent
7644	Etlin et al.,	2003	Pain research & management. 2003; 8(2):107-108	non pertinent
7645	Duffy et al.,	2003	Drugs. 2003; 63 Suppl 1:31-36	non pertinent
7646	Downar et al.,	2003	Neuroimage. 2003; 20(3):1540-1551	non pertinent

7647	Dietrich et al.,	2003	American j of gastroenterology. 2003; 98(11):2491-2499	non pertinent
7648	D'Amelio et al.,	2003	International j of tissue reactions. 2003; 25(2):73-78	non pertinent
7649	Csizy et al.,	2003	J of orthopaedic trauma. 2003; 17(2):106-112	non pertinent
7650	Cross et al.,	2003	Vaccine. 2003; 21(31):4576-4587	non pertinent
7651	Croom et al.,	2003	Drugs. 2003; 63(5):513-522	non pertinent
7652	Coimbra et al.,	2003	Anesth Analg. 2003; 97(3):839-842	non pertinent
7653	Choi et al.,	2003	Allergy: european j of allergy and clinical immunology. 2003; 58(11):1114-1116	non pertinent
7654	Chawla et al.,	2003	Therapeutic drug monitoring. 2003; 25(2):203-210	non pertinent
7655	Cecchi et al.,	2003	J Perinatol. 2003; 23(4):304-306	non pertinent
7656	Castro et al.,	2003	Head & neck. 2003; 25(9):717-731	non pertinent
7657	Caba et al.,	2003	Revista de la sociedad espanola del dolor. 2003; 10(6):341-348	non pertinent
7658	Buzzi et al.,	2003	Cephalalgia. 2003; 23(1_suppl):1-4	non pertinent
7659	Buggy et al.,	2003	Pain. 2003; 106(1-2):169-172	non pertinent
7660	Bruhn et al.,	2003	Anesthesiology. 2003; 98(3):621-627	non pertinent
7661	Bruehl et al.,	2003	Pain. 2003; 106(3):317-324	non pertinent
7662	Brotz et al.,	2003	J Neurol. 2003; 250(6):746-749	non pertinent
7663	Brien et al.,	2003	British j of clinical pharmacology. 2003; 56(5):562-568	non pertinent
7664	Brennan et al.,	2003	Oral surgery, oral medicine, oral pathology, oral radiology and endodontics. 2003; 95(4):416	non pertinent
7665	Bouwmeester et al.,	2003	Intensive care medicine. 2003; 29(11):2009-2015	non pertinent
7666	Bonnet et al.,	2003	J of clinical psychopharmacology. 2003; 23(5):514-519	non pertinent
7667	Bentley et al.,	2003	Neuroimage. 2003; 20(1):58-70	non pertinent

7668	Bentley et al.,	2003	J of neurophysiology. 2003; 90(2):1171-1181	non pertinent
7669	Beck et al.,	2003	Arch Ophthalmol. 2003; 121(7):944-949	non pertinent
7670	Barker et al.,	2003	J ect. 2003; 19(1):38-44	non pertinent
7671	Balit et al.,	2003	J of toxicology. Clinical toxicology. 2003; 41(6):801-808	non pertinent
7672	Bahr et al.,	2003	J of antimicrobial chemotherapy. 2003; 51(6):1377-1388	non pertinent
7673	Babul et al.,	2003	Clinical j of sport medicine. 2003; 13(3):138-147	non pertinent
7674	Attenburrow et al.,	2003	Psychopharmacology (Berl). 2003; 169(1):104-107	non pertinent
7675	Ashina et al.,	2003	Cephalalgia. 2003; 23(2):109-116	non pertinent
7676	Arrieta et al.,	2003	Antimicrobial agents and chemotherapy. 2003; 47(10):3179-3186	non pertinent
7677	Aristegui et al.,	2003	Vaccine. 2003; 21(25-26):3593-3600	non pertinent
7678	Appolinario et al.,	2003	Archives of general psychiatry. 2003; 60(11):1109-1116	non pertinent
7679	Angst et al.,	2003	Pain. 2003; 106(1-2):49-57	non pertinent
7680	Anderson et al.,	2003	J Neurosci. 2003; 23(13):5627-5633	non pertinent
7681	Almeida et al.,	2003	Drugs in R&D. 2003; 4(4):207-217	non pertinent
7682	Allen et al.,	2003	Psychopharmacology (Berl). 2003; 166(3):228-233	non pertinent
7683	Akhondzadeh et al.,	2003	J of clinical pharmacy and therapeutics. 2003; 28(5):379-384	non pertinent
7684	Ahmad et al.,	2003	Anesthesiology. 2003; 98(4):849-852	non pertinent
7685	Aaron et al.,	2003	Respiratory medicine. 2003; 97(11):1195-1199	non pertinent
7686	Aapro et al.,	2003	Annals of oncology. 2003; 14(3):441-448	non pertinent

7687	Müllges et al.,	2003	Cerebrovascular diseases (basel, switzerland). 2003; 15(3):222-229	non pertinent
7688	Raymond et al.,	2003	Alcoholism, clinical and experimental research. 2003; 27(7):1123-1131	non pertinent
7689	Zangara et al.,	2002	Psychopharmacology (Berl). 2002; 163(1):36-41	non pertinent
7690	Zachrisson et al.,	2002	European j of pain (London, England). 2002; 6(6):455-466	non pertinent
7691	Xia et al.,	2002	J of clinical anesthesia. 2002; 14(5):339-343	non pertinent
7692	Wu et al.,	2002	Neuroimage. 2002; 16(4):1028-1037	non pertinent
7693	Woessner et al.,	2002	Arthritis and rheumatism. 2002; 46(8):2201-	non pertinent
7694	Wilner et al.,	2002	J of clinical pharmacology. 2002; 42(9):1027-1030	non pertinent
7695	Williams et al.,	2002	J of rheumatology. 2002; 29(6):1280-1286	non pertinent
7696	Wiederhold et al.,	2002	IEEE transactions on information technology in biomedicine. 2002; 6(3):218-223	non pertinent
7697	Wetzel et al.,	2002	Current opinion in orthopaedics. 2002; 13(3):172-177	non pertinent
7698	Wetzel et al.,	2002	British j of sports medicine. 2002; 36(3):183-188	non pertinent
7699	Walters et al.,	2002	British j of clinical pharmacology. 2002; 53(3):305-311	non pertinent
7700	Wallace et al.,	2002	J of Pain. 2002; 3(3):227-233	non pertinent
7701	Van Honk et al.,	2002	Biol Psychiatry. 2002; 52(4):312-317	non pertinent
7702	Van der Post et al.,	2002	Cephalalgia. 2002; 22(4):271-281	non pertinent
7703	Vad et al.,	2002	Spine (Phila Pa 1976). 2002; 27(1):11-16	non pertinent
7704	Udelson et al.,	2002	Jama. 2002; 288(21):2693-2700	non pertinent

7705	Tracey et al.,	2002	J Neurosci. 2002; 22(7):2748-2752	non pertinent
7706	Tohda et al.,	2002	Clinical and experimental allergy. 2002; 32(8):1180-1186	non pertinent
7707	Tessitore et al.,	2002	J of neuroscience. 2002; 22(20):9099-9103	non pertinent
7708	Tervo et al.,	2002	Developmental medicine and neurology. 2002; 44(6):383-390	non pertinent
7709	Tecchio et al.,	2002	Hum Brain Mapp. 2002; 17(1):28-36	non pertinent
7710	Tebbutt et al.,	2002	Annals of oncology. 2002; 13(10):1568-1575	non pertinent
7711	Tang et al.,	2002	Human reproduction (Oxford, England). 2002; 17(11):2865-2868	non pertinent
7712	Suputtitada	2002	Am J Phys Med Rehabil. 2002; 81(10):770-775	non pertinent
7713	Storr et al.,	2002	BMC gastroenterology. 2002; 2	non pertinent
7714	Stewart et al.,	2002	J of ocular pharmacology and therapeutics. 2002; 18(3):211-220	non pertinent
7715	Spath	2002	Rheumatic disease clinics of north america. 2002; 28(2):319-328	non pertinent
7716	Smith et al.,	2002	Progress in anesthesiology. 2002; 16(14):219-230	non pertinent
7717	Slappendel et al.,	2002	European j of anaesthesiology. 2002; 19(11):829-831	non pertinent
7718	Skrumsager et al.,	2002	British j of clinical pharmacology. 2002; 53(3):284-295	non pertinent
7719	Siris et al.,	2002	Osteoporosis international. 2002; 13(11):907-913	non pertinent
7720	Shah et al.,	2002	Gastrointest Endosc. 2002; 55(7):832-837	non pertinent
7721	Schuck et al.,	2002	Fundam Clin Pharmacol. 2002; 16(1):57-65	non pertinent

7722	Schacker et al.,	2002	Antimicrobial agents and chemotherapy. 2002; 46(10):3243-3248	non pertinent
7723	Rosch et al.,	2002	Gastrointest Endosc. 2002; 55(7):870-876	non pertinent
7724	Röhss et al.,	2002	Digestive diseases and sciences. 2002; 47(5):954-958	non pertinent
7725	Rewari et al.,	2002	Anaesthesia and intensive care. 2002; 30(4):433-437	non pertinent
7726	Reinders et al.,	2002	Neuroimage. 2002; 17(4):1844-1853	non pertinent
7727	Poyares et al.,	2002	Progress in neuro-psychopharmacology & biological psychiatry. 2002; 26(3):539-545	non pertinent
7728	Porro et al.,	2002	J Neurosci. 2002; 22(8):3206-3214	non pertinent
7729	Pieniaszek et al.,	2002	J of clinical pharmacology. 2002; 42(7):738-753	non pertinent
7730	Petrovic et al.,	2002	Science (New York, N.Y.). 2002; 295(5560):1737-1740	non pertinent
7731	Peter et al.,	2002	American j of emergency medicine. 2002; 20(6):562-566	non pertinent
7732	Pecking et al.,	2002	British j of clinical pharmacology. 2002; 54(4):357-362	non pertinent
7733	Pearl et al.,	2002	Obstetrics and gynecology. 2002; 99(5 Pt 1):704-708	non pertinent
7734	Paterson	2002	Clinical breast cancer. 2002; 3(3):206-216	non pertinent
7735	Pandya et al.,	2002	Rheumatology international. 2002; 22(1):1-4	non pertinent
7736	Otsuji et al.,	2002	Therapeutic drug monitoring. 2002; 24(3):375-378	non pertinent
7737	Oga et al.,	2002	Psychiatry Clin Neurosci. 2002; 56(4):355-363	non pertinent
7738	Oerlemans et al.,	2002	Nederlands tijdschrift voor geneeskunde. 2002; 146(19):895-902	non pertinent

7739	Nct - Hoffman	2002	National Library of Medicine. 2002	non pertinent
7740	Nagasaki et al.,	2002	Masui. The japanese j of anesthesiology. 2002; 51(8):846-850	non pertinent
7741	Naber	2002	International j of antimicrobial agents. 2002; 20(1):18-27	non pertinent
7742	Myles et al.,	2002	Anesth Analg. 2002; 95(4):805-812	non pertinent
7743	Moulton et al.,	2002	Clinical cancer research. 2002; 8(7):2044-2051	non pertinent
7744	Morris et al.,	2002	Neuroimage. 2002; 17(1):214-222	non pertinent
7745	Moll et al.,	2002	J Neurosci. 2002; 22(7):2730-2736	non pertinent
7746	Mohar et al.,	2002	American j of gastroenterology. 2002; 97(10):2530-2535	non pertinent
7747	Mirzai et al.,	2002	Spine (Phila Pa 1976). 2002; 27(4):343-346	non pertinent
7748	Michelson et al.,	2002	American j of psychiatry. 2002; 159(11):1896-1901	non pertinent
7749	Mencke et al.,	2002	Der Anaesthesist. 2002; 51(9):721-725	non pertinent
7750	Martini et al.,	2002	J of cataract and refractive surgery. 2002; 28(6):1018-1022	non pertinent
7751	Martin-Garcia et al.,	2002	Chest. 2002; 121(6):1812-1817	non pertinent
7752	Malmstrom et al.,	2002	Clinical therapeutics. 2002; 24(10):1549-1560	non pertinent
7753	Maihofner et al.,	2002	Pain. 2002; 100(3):281-290	non pertinent
7754	Lowe et al.,	2002	J of cosmetic and laser therapy. 2002; 4(1):15-18	non pertinent
7755	Loughnan et al.,	2002	British j of anaesthesia. 2002; 89(3):466-472	non pertinent
7756	Looareesuwan et al.,	2002	British j of clinical pharmacology. 2002; 53(5):492-500	non pertinent
7757	Lok et al.,	2002	Human reproduction (Oxford, England). 2002; 17(8):2101-2106	non pertinent

7758	Lim et al.,	2002	J of international medical research. 2002; 30(2):137-143	non pertinent
7759	Levett et al.,	2002	HIV clinical trials. 2002; 3(4):272-278	non pertinent
7760	Leslie et al.,	2002	Anesth Analg. 2002; 94(5):1298-1303	non pertinent
7761	Lehmann et al.,	2002	Anesth Analg. 2002; 95(3):639-644	non pertinent
7762	Lee et al.,	2002	International j of urology. 2002; 9(5):247-252	non pertinent
7763	Laureys et al.,	2002	Neuroimage. 2002; 17(2):732-741	non pertinent
7764	Lang et al.,	2002	Spine (Phila Pa 1976). 2002; 27(20):2284-2290	non pertinent
7765	Kuo et al.,	2002	Aliment Pharmacol Ther. 2002; 16(2):225-233	non pertinent
7766	Kuiken et al.,	2002	Alimentary pharmacology & therapeutics. 2002; 16(11):1955-1962	non pertinent
7768	Krenn et al.,	2002	British j of anaesthesia. 2002; 89(4):637-640	non pertinent
7769	Kong et al.,	2002	J of alternative and complementary medicine (New York, N.Y.). 2002; 8(4):411-419	non pertinent
7770	Kelion et al.,	2002	Heart (British Cardiac Society). 2002; 87(2):115-120	non pertinent
7771	Kawamata et al.,	2002	Pain. 2002; 100(1-2).77-89	non pertinent
7772	Jernberg et al.,	2002	J of the American College of Cardiology. 2002; 40(3):437-445	non pertinent
7773	Iversen et al.,	2002	European urology. 2002; 42(3):204-211	non pertinent
7774	Hügler et al.,	2002	European j of pain (London, England). 2002; 6(6):435-445	non pertinent
7775	Holroyd	2002	J of consulting and clinical psychology. 2002; 70(3):656-677	non pertinent
7776	Hentgen et al.,	2002	Anesthesia and Analgesia. 2002; 95(3):597-605	non pertinent

7777	Hayashi et al.,	2002	J Neurosurg. 2002; 97(5 Suppl):433-437	non pertinent
7778	Hawkes et al.,	2002	Alimentary pharmacology & therapeutics. 2002; 16(9):1649-1654	non pertinent
7779	Harman et al.,	2002	Sleep. 2002; 25(7):775-783	non pertinent
7780	Hariri et al.,	2002	Neuroimage. 2002; 17(1):317-323	non pertinent
7781	Hariri et al.,	2002	Neuropsychophar macology. 2002; 27(6):1036-1040	non pertinent
7782	Hanas et al.,	2002	J of pediatrics. 2002; 140(3):315-320	non pertinent
7783	Halperin et al.,	2002	Pediatric infectious disease j. 2002; 21(5):399-405	non pertinent
7784	Guyader et al.,	2002	Hepatology (baltimore, md.). 2002; 36(5):1197-1205	non pertinent
7785	Grundman et al.,	2002	J of molecular neuroscience. 2002; 18(3):283-293	non pertinent
7786	Gross-Tsur et al.,	2002	J of child neurology. 2002; 17(12):863-866	non pertinent
7787	Green	2002	Somatosens Mot Res. 2002; 19(2):130-138	non pertinent
7788	Grachev et al.,	2002	Chem Neuroanat. 2002; 23(3):223-230	non pertinent
7789	Grachev et al.,	2002	J Neural Transm (Vienna). 2002; 109(10):1309-1334	non pertinent
7790	Gordon et al.,	2002	Anesthesia and Anagesia. 2002; 95(5):1351-1357	non pertinent
7791	Forseth et al.,	2002	Drugs. 2002; 62(4):577-592	non pertinent
7792	Floravanti et al.,	2002	Clinical therapeutics. 2002; 24(4):504-519	non pertinent
7793	Eisenach et al.,	2002	Anesthesiology. 2002; 96(1):24-28	non pertinent
7794	Eisenach et al.,	2002	Anesthesiology. 2002; 96(1):29-34	non pertinent
7795	Eimer et al.,	2002	Neuroreport. 2002; 13(4):427-431	non pertinent
7796	Dunbar et al.,	2002	Anesth Analg. 2002; 94(2):417-420	non pertinent

7797	Di Lazzaro et al.,	2002	Clin Neurophysiol. 2002; 113(11):1673-1679	non pertinent
7798	Desjardins et al.,	2002	European j of clinical pharmacology. 2002; 58(6):387-394	non pertinent
7799	Derbyshire et al.,	2002	Neuroimage. 2002; 16(1):158-168	non pertinent
7800	De los Rios Castillo et al.,	2002	Salud mental (mexico city, mexico). 2002; 25(2):21-31	non pertinent
7801	De Andres	2002	Expert review of neurotherapeutics. 2002; 2(6):791-799	non pertinent
7802	Dannecker et al.,	2002	J Sports Med Phys Fitness. 2002; 42(4):458-465	non pertinent
7803	Cook et al.,	2002	Psychopharmacology. 2002; 162(2):186-192	non pertinent
7804	Cohen et al.,	2002	Paediatric anaesthesia. 2002; 12(7):604-609	non pertinent
7805	Choy et al.,	2002	Clinical and experimental rheumatology. 2002; 20(3):351-358	non pertinent
7806	Chow et al.,	2002	Hepatology (baltimore, md.). 2002; 36(5):1221-1226	non pertinent
7807	Chou et al.,	2002	International j of clinical pharmacology research. 2002; 22(1):1-6	non pertinent
7808	Chang et al.,	2002	Clinical therapeutics. 2002; 24(4):490-503	non pertinent
7809	Catalano et al.,	2002	AJR. American j of roentgenology. 2002; 178(2):379-387	non pertinent
7810	Carragee et al.,	2002	Spine J. 2002; 2(1):25-34	non pertinent
7811	Carlsson et al.,	2002	Drugs & aging. 2002; 19(10):793-805	non pertinent
7812	Calbo et al.,	2002	Medicina clinica. 2002; 118(1):1-4	non pertinent
7813	Burton et al.,	2002	Annals of emergency medicine. 2002; 40(5):496-504	non pertinent

7814	Burkus et al.,	2002	J of spinal disorders. 2002; 15(5):337-349	non pertinent
7815	Buckenmaier et al.,	2002	Anesthesia and Analgesia. 2002; 95(5):1253-1257	non pertinent
7816	Buchei et al.,	2002	J Neurosci. 2002; 22(3):970-976	non pertinent
7817	Bruehl et al.,	2002	Pain. 2002; 100(1-2):191-201	non pertinent
7818	Bruehl et al.,	2002	Pain. 2002; 99(1-2):223-233	non pertinent
7819	Brandt et al.,	2002	American j of gastroenterology. 2002; 97(11 SUPPL):1-26	non pertinent
7820	Bernstein et al.,	2002	American j of gastroenterology. 2002; 97(2):319-327	non pertinent
7821	Berman et al.,	2002	Gastroenterology. 2002; 123(4):969-977	non pertinent
7822	Bergogne-Berezin	2002	Expert opinion on pharmacotherapy. 2002; 3(10):1471-1479	non pertinent
7823	Bacon et al.,	2002	British j of clinical pharmacology. 2002; 53(6):629-636	non pertinent
7824	Astin et al.,	2002	Arthritis care & research. 2002; 47(3):291-302	non pertinent
7825	Asik et al.,	2002	European j of anaesthesiology. 2002; 19(4):263-270	non pertinent
7826	Argyropoulos et al.,	2002	Psychoneuroendo crinology. 2002; 27(6):715-729	non pertinent
7827	Anegon et al.,	2002	Clinical therapeutics. 2002; 24(5):786-797	non pertinent
7828	Andrew et al.,	2002	Anaesthesia and intensive care. 2002; 30(3):321-325	non pertinent
7829	Anderson et al.,	2002	Pain. 2002; 99(1-2):207-216	non pertinent
7830	Agneskirchner et al.,	2002	Knee Surg Sports Traumatol Arthrosc. 2002; 10(3):160-168	non pertinent
7831	Abe et al.,	2002	Current medicinal chemistry – anti-cancer agents. 2002; 2(6):715-726	non pertinent
7832	Witting et al.,	2001	Neurology. 2001; 57(10):1817-1824	non pertinent
7833	Wissel et al.,	2001	J of neurology. 2001; 248(12):1073-1078	non pertinent

7834	Williams et al.,	2001	J of periodontology. 2001; 72(11):1535-1544	non pertinent
7835	Wilder-Smith et al.,	2001	Can J Anaesth. 2001; 48(5):439-445	non pertinent
7836	Wellmer et al.,	2001	J oft he peripheral nervous system : JPNS. 2001; 6(4):204-210	non pertinent
7837	Wambebe et al.,	2001	Phytomedicine. 2001; 8(4):252-261	non pertinent
7838	Vogt et al.,	2001	Wound repair and regeneration. 2001; 9(2):116-122	non pertinent
7839	Van Gaal et al.,	2001	Diabetes, obesity & metabolism. 2001; 3(5):326-331	non pertinent
7840	Van den Hout et al.,	2001	Pain. 2001; 92(1-2):247-257	non pertinent
7841	Turner et al.,	2001	HIV medicine. 2001; 2(2):68-77	non pertinent
7842	Toubro et al.,	2001	Ugeskrift for laeger. 2001; 163(21):2935-2940	non pertinent
7843	Tomer et al.,	2001	Thrombosis and haemostasis. 2001; 85(6):966-974	non pertinent
7844	Thompson et al.,	2001	British j of anaesthesia. 2001; 87(2):283-286	non pertinent
7845	Thompson et al.,	2001	International j of sport nutrition and exercise metabolism. 2001; 11(4):466-481	non pertinent
7846	Temple et al.,	2001	Rhinology. 2001; 39(4):230-232	non pertinent
7847	Tariot et al.,	2001	J of the American Geriatrics Society. 2001; 49(12):1590-1599	non pertinent
7848	Sterling et al.,	2001	Man Ther. 2001; 6(2):72-81	non pertinent
7849	Staats et al.,	2001	Pain medicine (Malden, Mass). 2001; 2(1):28-34	non pertinent
7850	Song et al.,	2001	European j of nuclear medicine. 2001; 28(4):489-497	non pertinent
7851	Singh et al.,	2001	Indian j of physiology and pharmacology. 2001; 45(2):181-190	non pertinent
7852	Shah et al.,	2001	Anaesthesia. 2001; 56(4):302-308	non pertinent

7853	Seong et al.,	2001	Ophthalmologica. J international d'ophthalmologie (International j of ophthalmology). 2001; 215(3):188-191	non pertinent
7854	Scorza et al.,	2001	Clinical and experimental rheumatology. 2001; 19(5):503-508	non pertinent
7855	Schwartz et al.,	2001	Aliment Pharmacol Ther. 2001; 15(12):1921-1928	non pertinent
7856	Schneider et al.,	2001	Neuroradiology. 2001; 43(6):472-476	non pertinent
7857	Sarullo et al.,	2001	Italian heart j. 2001; 2(8):605-611	non pertinent
7858	Saray et al.,	2001	Acta chirurgiae plasticae. 2001; 43(3):71-76	non pertinent
7859	Santiveri et al.,	2001	Revista espanola de anestesiologia y reanimacion. 2001; 48(9):409-414	non pertinent
7860	Sandler et al.,	2001	J Oral Maxillofac Surg. 2001; 59(6):603-611	non pertinent
7861	Salvarani et al.,	2001	J of rheumatology. 2001; 28(10):2274-2282	non pertinent
7862	Saller et al.,	2001	Forschende Komplementmedizin und klassische Naturheilkunde (Research in complementary and natural classical medicine). 2001; 8(6):373-382	non pertinent
7863	Rossini et al.,	2001	Multiple sclerosis (houndmills, basingstoke, england). 2001; 7(6):354-358	non pertinent
7864	Rome et al.,	2001	Clinical biomechanics (Britol, Avon). 2001; 16(10):901-905	non pertinent
7865	Rachev et al.,	2001	Current medical research and opinion. 2001; 17(2):105-110	non pertinent
7866	Quattrin et al.,	2001	J of pediatric endocrinology & metabolism. 2001; 14(3):267-277	non pertinent

7867	Pollak et al.,	2001	Psychophysiology. 2001; 38(2):267-274	non pertinent
7868	Ploghaus et al.,	2001	Neurosci. 2001; 21(24):9896-9903	non pertinent
7869	Piscoya et al.,	2001	Inflammation research. 2001; 50(9):442-448	non pertinent
7870	Phillips et al.,	2001	American j of gastroenterology. 2001; 96(2):409-416	non pertinent
7871	Petroni et al.,	2001	Alimentary pharmacology & therapeutics. 2001; 15(1):123-128	non pertinent
7872	Pessina et al.,	2001	Blood pressure. 2001; 10(3):176-183	non pertinent
7873	Persson et al.,	2001	Disability and rehabilitation. 2001; 23(8):325-335	non pertinent
7874	Pereira et al.,	2001	J of periodontology. 2001; 72(12):1720-1725	non pertinent
7875	Pendeville et al.,	2001	Acta anaesthesiologica belgica. 2001; 52(2):181-186	non pertinent
7876	Passàli et al.,	2001	Clinical therapeutics. 2001; 23(9):1508-1518	non pertinent
7877	Park et al.,	2001	J of the peripheral nervous system : JPNS. 2001; 6(4):232-240	non pertinent
7878	Pagella et al.,	2001	Otorinolaringologia. 2001; 51(2):77-81	non pertinent
7879	Ozaki et al.,	2001	J canadien d'anesthesie (Canadian j of anaesthesia). 2001; 48(11):1080-1083	non pertinent
7880	Onen et al.,	2001	J Sleep Res. 2001; 10(1):35-42	non pertinent
7881	Olausson et al.,	2001	Brain. 2001; 124(Pt 5):916-927	non pertinent
7882	Norman et al.,	2001	Anesthesiology. 2001; 94(4):599-603	non pertinent
7883	Nielson et al.,	2001	Clinical J of Pain. 2001; 17(4 SUPPL):114-127	non pertinent
7884	Negishi et al.,	2001	Anesthesiology. 2001; 94(2):218-222	non pertinent
7885	Nct - Shen	2001	National Library of Medicine. 2001	non pertinent

7886	Nct – National Institute of Dental and Craniofacial Research	2001	National Library of Medicine. 2001	non pertinent
7887	Nakabeppu et al.,	2001	Annals of nuclear medicine. 2001; 15(5):459-463	non pertinent
7888	Mullican et al.,	2001	Clinical therapeutics. 2001; 23(9):1429-1445	non pertinent
7889	Mould et al.,	2001	Vox sanguinis. 2001; 80(3):151-158	non pertinent
7890	Miller et al.,	2001	J Pediatr. 2001; 139(6):785-789	non pertinent
7891	Miller et al.,	2001	AANA j. 2001; 69(3):185-187	non pertinent
7892	Meyer et al.,	2001	Zeitschrift für medizinische physik. 2001; 11(1):5-13	non pertinent
7893	McDonald et al.,	2001	Res Nurs Health. 2001; 24(5):402-409	non pertinent
7894	Mathew	2001	Clinical cornerstone. 2001; 4(3):1-16	non pertinent
7895	Mannion et al.,	2001	Schmerz (berlin, germany). 2001; 15(6):468-473	non pertinent
7896	Luginbuhl et al.,	2001	Anesthesiology. 2001; 95(1):22-29	non pertinent
7897	Lefaucheur et al.,	2001	Muscle Nerve. 2001; 24(4):496-501	non pertinent
7898	Layton et al.,	2001	Depress Anxiety. 2001; 14(4):251-254	non pertinent
7899	Lancaster et al.,	2001	Int J Cardiol. 2001; 80(2-3):201-207	non pertinent
7900	Laakso et al.,	2001	Behav Brain Res. 2001; 118(2):187-193	non pertinent
7901	Krucoff et al.,	2001	American heart j. 2001; 142(5):760-769	non pertinent
7902	Kronenberg et al.,	2001	Depress Anxiety. 2001; 14(2):141-144	non pertinent
7903	Kozuch et al.,	2001	Cancer. 2001; 91(4):815-821	non pertinent
7904	Kopacz et al.,	2001	Anesthesiology. 2001; 95(6):1371-1376	non pertinent
7905	Ko et al.,	2001	Acta anaesthesiologica sinica. 2001; 39(4):169-177	non pertinent
7906	Kligman et al.,	2001	Arch Orthop Trauma Surg. 2001; 121(3):139-141	non pertinent

7907	Kieffer et al.,	2001	International angiology. 2001; 20(1):58-65	non pertinent
7908	Karl et al.,	2001	J Neurosci. 2001; 21(10):3609-3618	non pertinent
7909	Karim et al.,	2001	J of clinical pharmacology. 2001; 41(10):1111-1119	non pertinent
7910	Huse et al.,	2001	Pain. 2001; 90(1-2):47-55	non pertinent
7911	Hollenhorst et al.,	2001	AJR Am J Roentgenol. 2001; 176(4):865-868	non pertinent
7912	Hasaniya et al.,	2001	Surgical endoscopy. 2001; 15(9):962-964	non pertinent
7913	Harris et al.,	2001	Peritoneal dialysis international. 2001; 21 Suppl 3:144-147	non pertinent
7914	Hand et al.,	2001	AANA j. 2001; 69(6):466-470	non pertinent
7915	Hachero Torrejon et al.,	2001	Revista espanola de anestesologia y reanimacion. 2001; 48(8):364-369	non pertinent
7916	Gustorff et al.,	2001	Anesth Analg. 2001; 92(2):369-374	non pertinent
7917	Gürsoy et al.,	2001	Rheumatology international. 2001; 21(2):58-61	non pertinent
7918	Gulsoy et al.,	2001	Lasers Surg Med. 2001; 28(3):244-247	non pertinent
7919	Grusser et al.,	2001	Neuroscience. 2001; 102(2):263-272	non pertinent
7920	Grunau et al.,	2001	Pediatrics. 2001; 107(1):105-112	non pertinent
7921	Gomolin et al.,	2001	J of the American Geriatrics Society. 2001; 49(12):1606-1613	non pertinent
7922	Gasparini et al.,	2001	European j of epidemiology. 2001; 17(2):135-140	non pertinent
7923	Garcia et al.,	2001	Revista brasileira de anestesiologia. 2001; 51(5):377-384	non pertinent
7924	Gale et al.,	2001	Anaesth Intensive Care. 2001; 29(6):579-584	non pertinent
7925	Friedman et al.,	2001	Heart disease. 2001; 3(6):357-361	non pertinent
7926	Flor et al.,	2001	Lancet (london, england). 2001; 357(9270):1763-1764	non pertinent

7927	Flach et al.,	2001	Transactions of the american ophthalmological society. 2001; 99:205-212	non pertinent
7928	Fernieini et al.,	2001	Oral Surg Oral Med Oral Pathol Radiol Endod. 2001; 91(5):526-530	non pertinent
7929	Ferdinand et al.,	2001	American j of hypertension. 2001; 14(8 Pt 1):788-793	non pertinent
7930	Ehlers et al.,	2001	Annals of anatomy. 2001; 183(1):13-17	non pertinent
7931	Edwards et al.,	2001	J of gastroenterology and hepatology (australia). 2001; 16(11):1235-1238	non pertinent
7932	Di Pasquale et al.,	2001	Italian heart j. 2001; 2(10):751-756	non pertinent
7933	Demir et al.,	2001	J of gastroenterology and hepatology (australia). 2001; 16(9):1071-1074	non pertinent
7934	Dalery et al.,	2001	Encephale. 2001; 27(1):71-81	non pertinent
7935	D'Hooge et al.,	2001	International angiology. 2001; 20(4):288-294	non pertinent
7936	Convery et al.,	2001	British j of anaesthesia. 2001; 87(4):570-576	non pertinent
7937	Coan et al.,	2001	Psychophysiology. 2001; 38(6):912-925	non pertinent
7938	Chung et al.,	2001	Clinical therapeutics. 2001; 23(6):858-870	non pertinent
7939	Chen et al.,	2001	Neurosci Lett. 2001; 297(3):183-186	non pertinent
7940	Chang et al.,	2001	Clinical therapeutics. 2001; 23(9):1446-1455	non pertinent
7941	Cha et al.,	2001	International j of clinical pharmacology and therapeutics. 2001; 39(12):539-545	non pertinent
7942	Cabarrocas	2001	Clinical therapeutics. 2001; 23(11):1867-1875	non pertinent
7943	Cabane et al.,	2001	Presse medicale. 2001; 30(18):885-891	non pertinent

7944	Brooks et al.,	2001	J Am Coll Cardiol. 2001; 38(6):1589-1595	non pertinent
7945	Brennum et al.,	2001	Acta anaesthesiologica Scandinavica. 2001; 45(8):954-960	non pertinent
7946	Boucher et al.,	2001	J canadien d'anesthesie (Canadian j of anaesthesia). 2001; 48(5):466-469	non pertinent
7947	Boccalon et al.,	2001	Annales of cardiologie ET d'angeiologie. 2001; 50(3):175-182	non pertinent
7948	Biella et al.,	2001	Neuroimage. 2001; 14(1 Pt 1):60-66	non pertinent
7949	Baran	2001	British j of dermatology. 2001; 145 Suppl 60:15-19	non pertinent
7950	Baldwin et al.,	2001	J Gerontol A Biol Sci Med Sci. 2001; 56(8):M510-513	non pertinent
7951	Bakheit et al.,	2001	European j of neurology. 2001; 8(6):559-565	non pertinent
7952	Arslan et al.,	2001	Rheumatology international. 2001; 21(1):20-23	non pertinent
7953	Amanzio et al.,	2001	Pain. 2001; 90(3):205-215	non pertinent
7954	Allescher et al.,	2001	Endoscopy. 2001; 33(12):1007-1017	non pertinent
7955	Alfieri	2001	J Neurosurg Sci. 2001; 45(1):15-18	non pertinent
7956	Alden et al.,	2001	Appl Psychophysiol Biofeedback. 2001; 26(2):117-126	non pertinent
7957	Aichelmann-Reidy	2001	J of periodontology. 2001; 72(8):998-1005	non pertinent
7958	Affaitati et al.,	2001	Drugs under experimental and clinical research. 2001; 27(2):69-76	non pertinent
7959	European Atosiban Study Group	2001	Acta obstetrica ET gynecologica scandinavica. 2001; 80(5):413-422	non pertinent
7960	Zhai et al.,	2000	Skin pharmacology and applied skin physiology. 2000; 13(6):352-357	non pertinent

7961	Yocum et al.,	2000	Archives of internal medicine. 2000; 160(19):2947-2954	non pertinent
7962	Wang et al.,	2000	Pain. 2000; 88(2):189-197	non pertinent
7963	Walker et al.,	2000	J of clinical psychopharmacology. 2000; 20(6):636-644	non pertinent
7964	Vogel et al.,	2000	Proc-annu-meet-am-soc-clin-oncol. 2000; 19:Abstract275	non pertinent
7965	Videman et al.,	2000	Clin Biomech (Bristol, Avon). 2000; 15(2):83-86	non pertinent
7966	Vélez et al.,	2000	Transactions of the Royal Society of Tropical Medicine and Hygiene. 2000; 94(6):698-703	non pertinent
7967	Veldhuyzen Van Zanten et al.,	2000	Alimentary pharmacology & therapeutics. 2000; 14(12):1605-1611	non pertinent
7968	Van zandwijk et al.,	2000	J of the National Cancer Institute. 2000; 92(12):977-986	non pertinent
7969	Van Dyck et al.,	2000	Archives of general psychiatry. 2000; 57(2):157-164	non pertinent
7970	Van den Hout et al.,	2000	Eur J Pain. 2000; 4(4):335-346	non pertinent
7971	Uhlemann et al.,	2000	Physikalische medizin rehabilitationsmedizin kurortmedizin. 2000; 10(3):86-93	non pertinent
7972	Thjodleifsson et al.,	2000	Digestive diseases and sciences. 2000; 45(5):845-853	non pertinent
7973	Telch et al.,	2000	Behaviour research and therapy. 2000; 38(4):373-387	non pertinent
7974	Stowers et al.,	2000	Annals of emergency medicine. 2000; 35(1):17-25	non pertinent
7975	Spiegel et al.,	2000	Clin Neurophysiol. 2000; 111(4):725-735	non pertinent
7976	Sperber et al.,	2000	Archives of family medicine. 2000; 9(10):979-985	non pertinent

7977	Silberstein et al.,	2000	Headache. 2000; 40(6):445-450	non pertinent
7978	Shekelle et al.,	2000	Health Serv Res. 2000; 34(7):1429-1448	non pertinent
7979	Shao et al.,	2000	Anesth Analg. 2000; 91(4):871-875	non pertinent
7980	Schimel et al.,	2000	J Pers Soc Psychol. 2000; 78(3):446-462	non pertinent
7981	Schachter et al.,	2000	Expert opinion on investigational drugs. 2000; 9(4):871-883	non pertinent
7982	Sawamoto et al.,	2000	J Neurosci. 2000; 20(19):7438-7445	non pertinent
7983	Sarullo et al.,	2000	Italian heart j supplement. 2000; 1(1):81-87	non pertinent
7984	Rouillon et al.,	2000	International clinical psychopharmacology. 2000; 15(3):133-140	non pertinent
7985	Rosen et al.,	2000	Ann Med. 2000; 32(5):350-364	non pertinent
7986	Romanowski et al.,	2000	AIDS (london, england). 2000; 14(9):1211-1217	non pertinent
7987	Romaniello et al.,	2000	Exp Brain Res. 2000; 132(3):306-313	non pertinent
7988	Ripouteau et al.,	2000	Bmj. 2000; 321(7274):1460-1463	non pertinent
7989	Prahalad et al.,	2000	Arthritis and rheumatism. 2000; 43(10):2339-	non pertinent
7990	Ploner et al.,	2000	J Neurophysiol. 2000; 83(3):1770-1776	non pertinent
7991	Petrovic et al.,	2000	Pain. 2000; 85(1-2):19-30	non pertinent
7992	Pépin et al.,	2000	Bulletin of the world health organization. 2000; 78(11):1284-1295	non pertinent
7993	Penson et al.,	2000	Clinical pharmacology and therapeutics. 2000; 68(6):667-676	non pertinent
7994	Pasqualucci et al.,	2000	Acta anaesthesiologica Scnadinavica. 2000; 44(8):910-918	non pertinent
7995	Pares et al.,	2000	J of hepatology. 2000; 32(4):561-566	non pertinent

7996	Palme et al.,	2000	Laryngoscope. 2000; 110(12):2000-2004	non pertinent
7997	Paddock et al.,	2000	J Genet Psychol. 2000; 161(4):453-468	non pertinent
7998	Otto et al.,	2000	J of anxiety disorders. 2000; 14(4):345-358	non pertinent
7999	Ong et al.,	2000	Anaesthesia and intensive care. 2000; 28(5):527-531	non pertinent
8000	Nishina et al.,	2000	Paediatric anaesthesia. 2000; 10(6):645-651	non pertinent
8001	Nguyen et al.,	2000	Arch Med Res. 2000; 31(3):263-265	non pertinent
8002	Nevitt et al.,	2000	Archives of internal medicine. 2000; 160(1):77-85	non pertinent
8003	Mosimann et al.,	2000	Psychiatry Res. 2000; 94(3):251-256	non pertinent
8004	Morgan et al.,	2000	American j of hypertension. 2000; 13(11):1161-1167	non pertinent
8005	Moreno et al.,	2000	J of physiology and biochemistry. 2000; 56(3):209-216	non pertinent
8006	Moore et al.,	2000	American j of hematology. 2000; 64(1):26-31	non pertinent
8007	Miller et al.,	2000	Clinical therapeutics. 2000; 22(12):1433-1442	non pertinent
8008	Meyers et al.,	2000	Appl Neuropsychol. 2000; 7(3):133-139	non pertinent
8009	Malaguarnera et al.,	2000	Diabetes, obesity & metabolism. 2000; 2(1):33-38	non pertinent
8010	Lozano-Tonkin et al.,	2000	Anales de medicina interna. 2000; 17(8):399-405	non pertinent
8011	Lazowski	2000	Annals of transplantation. 2000; 5(1):28-29	non pertinent
8012	Lauretti et al.,	2000	J of clinical anesthesia. 2000; 12(6):444-448	non pertinent
8013	Lanza et al.,	2000	American j of gastroenterology. 2000; 95(11):3112-3117	non pertinent
8014	Kwan et al.,	2000	Pain. 2000; 85(3):359-374	non pertinent

8015	Kraj et al.,	2000	Acta poloniae pharmaceutica. 2000; 57 Suppl:113-116	non pertinent
8016	Kornowski et al.,	2000	Seminars in interventional cardiology : SIIC. 2000; 5(2):97-101	non pertinent
8017	Kokki et al.,	2000	British j of anaesthesia. 2000; 85(6):836-840	non pertinent
8018	Kjeldsen et al.,	2000	J of hypertension. 2000; 18(5):629-642	non pertinent
8019	Kaufmann et al.,	2000	J of clinical oncology. 2000; 18(7):1399-1411	non pertinent
8020	Karasawa et al.,	2000	J of anesthesia. 2000; 14(3):135-137	non pertinent
8021	Kanda et al.,	2000	Brain research. 2000; 853(2):282-289	non pertinent
8022	Kain et al.,	2000	Anesthesiology. 2000; 93(1):141-147	non pertinent
8023	Kahirlas et al.,	2000	Alimentary pharmacology & therapeutics. 2000; 14(10):1249-1258	non pertinent
8024	Jones et al.,	2000	Anaesthesia and intensive care. 2000; 28(5):491-500	non pertinent
8025	Jayousi	2000	Saudi medical j. 2000; 21(11):1071-1073	non pertinent
8026	Humphreys et al.,	2000	J of pediatric gastroenterology and nutrition. 2000; 31(1):47-51	non pertinent
8027	Homma et al.,	2000	Dementia and geriatric cognitive disorders. 2000; 11(6):299-313	non pertinent
8028	Hombrink et al.,	2000	Strahlentherapie und Onkologie. 2000; 176(4):173-179	non pertinent
8029	Hofman et al.,	2000	Prensa medica argentina. 2000; 87(5):513-520	non pertinent
8030	Hill et al.,	2000	Psychopharmacology. 2000; 152(1):31-39	non pertinent
8031	Hawkey et al.,	2000	Arthritis and rheumatism. 2000; 43(2):370-	non pertinent
8032	Hariri et al.,	2000	Neuroreport. 2000; 11(1):43-48	non pertinent
8033	Hanly et al.,	2000	J of rheumatology. 2000; 27(3):719-722	non pertinent

8034	Hadhazy et al.,	2000	J of rheumatology. 2000; 27(12):2911-2918	non pertinent
8035	Guignard et al.,	2000	Anesth Analg. 2000; 90(1):161-167	non pertinent
8036	Green et al.,	2000	Physiol Behav. 2000; 68(5):631-639	non pertinent
8037	Grahame	2000	Joint, bone, spine. 2000; 67(3):157-163	non pertinent
8038	Gehanno et al.,	2000	Scandinavian j of infectious diseases. 2000; 32(6):679-684	non pertinent
8039	Furst et al.,	2000	Therapeutic apheresis. 2000; 4(5):363-373	non pertinent
8040	Fujioka et al.,	2000	Diabetes, obesity & metabolism. 2000; 2(3):175-187	non pertinent
8041	Frenck et al.,	2000	Clinical infectious diseases. 2000; 31(5):1134-1138	non pertinent
8042	Franke et al.,	2000	Rheumatology. 2000; 39(8):894-902	non pertinent
8043	Fogelman et al.,	2000	J of clinical endocrinology and metabolism. 2000; 85(5):1895-1900	non pertinent
8044	Fock et al.,	2000	Alimentary pharmacology & therapeutics. 2000; 14(2):225-231	non pertinent
8045	File et al.,	2000	J of chemotherapy (florence, italy). 2000; 12(4):314-325	non pertinent
8046	Farber et al.,	2000	Scandinavian j of rheumatology, supplement. 2000; 29(113):49-54	non pertinent
8047	El Saied et al.,	2000	J canadien d'anesthesie (Canadian j of anaesthesia). 2000; 47(10):962-967	non pertinent
8048	Edmondston et al.,	2000	Man Ther. 2000; 5(3):158-164	non pertinent
8049	Drummond	2000	Anesthesia and Analgesia. 2000; 90(4):923-927	non pertinent
8050	Dormandy	2000	European j of vascular and endovascular surgery. 2000; 20(4):358-362	non pertinent

8051	Diamond et al.,	2000	Cephalalgia. 2000; 20(6):597-602	non pertinent
8052	Desbiens et al.,	2000	J of the American Geriatrics Society. 2000; 48(1):183-186	non pertinent
8053	Delchier et al.,	2000	Scandinavian j of gastroenterology. 2000; 35(12):1245-1250	non pertinent
8054	DeAbate et al.,	2000	Respiratory medicine. 2000; 94(11):1029-1037	non pertinent
8055	De Deyne et al.,	2000	J Clin Anesth. 2000; 12(1):52-57	non pertinent
8056	Davis et al.,	2000	J Neurophysiol. 2000; 83(6):3575-3577	non pertinent
8057	Craig et al.,	2000	Nat Neurosci. 2000; 3(2):184-190	non pertinent
8058	Chung et al.,	2000	Acta anaesthesiologica Scandinavica. 2000; 44(7):790-798	non pertinent
8059	Christiansen et al.,	2000	Cephalalgia. 2000; 20(5):437-444	non pertinent
8060	Chiaretti et al.,	2000	Child's nervous system : chns : official j of the international society for pediatric neurosurgery. 2000; 16(2):93-99	non pertinent
8061	Castro et al.,	2000	Revista de la sociedad espanola del dolor. 2000; 7(1):12-16	non pertinent
8062	Casey et al.,	2000	J of neurophysiology. 2000; 84(1):525-533	non pertinent
8063	Carragee et al.,	2000	Spine (Phila Pa 1976). 2000; 25(23):3065-3071	non pertinent
8064	Cameron et al.,	2000	Spine (Phila Pa 1976). 2000; 25(15):1917-1924	non pertinent
8065	Calaluce et al.,	2000	Cancer epidemiology, biomarkers & prevention. 2000; 9(12):1287-1292	non pertinent
8066	Cady et al.,	2000	Clinical therapeutics. 2000; 22(9):1035-1048	non pertinent
8067	Buchner et al.,	2000	Neuroreport. 2000; 11(6):1289-1293	non pertinent

8068	Bradwejn et al.,	2000	J of clinical psychopharmacology. 2000; 20(6):680-684	non pertinent
8069	Boon et al.,	2000	Clinical therapeutics. 2000; 22(1):76-90	non pertinent
8070	Bonk et al.,	2000	J of musculoskeletal pain. 2000; 8(1-2):123-132	non pertinent
8071	Bonina et al.,	2000	J of pharmacy and pharmacology. 2000; 52(10):1279-1285	non pertinent
8072	Blomhoff et al.,	2000	Scand J Gastroenterol. 2000; 35(6):583-589	non pertinent
8073	Bendtsen et al.,	2000	Cephalalgia. 2000; 20(6):603-610	non pertinent
8074	Azad et al.,	2000	Bangladesh medical research council bulletin. 2000; 26(2):41-47	non pertinent
8075	Ashina et al.,	2000	Ugeskrift for laeger. 2000; 162(2):171-173	non pertinent
8076	Apkarian et al.,	2000	J Neurophysiol. 2000; 83(5):3113-3122	non pertinent
8077	Angst et al.,	2000	Anesthesiology. 2000; 92(2):312-324	non pertinent
8078	Abouleish et al.,	2000	J canadien d'anesthesie (Canadian j of anaesthesia). 2000; 47(12):1171-1175	non pertinent
8079	Zullo et al.,	1999	Italian j of gastroenterology and hepatology. 1999; 31(9):831-835	non pertinent
8080	Zanten et al.,	1999	Alimentary pharmacology & therapeutics. 1999; 13(3):289-295	non pertinent
8081	Ylikorkala et al.,	1999	Revista brasileira de medicina. 1999; 56(10):1042-1047	non pertinent
8082	Wulsin et al.,	1999	International j of psychiatry in medicine. 1999; 29(1):97-105	non pertinent
8083	Wu et al.,	1999	Radiology. 1999; 212(1):133-141	non pertinent

8084	Woodman	1999	Obstetrics and gynecology. 1999; 93(5 Pt 1):775-779	non pertinent
8085	Wong et al.,	1999	Addiction (Abingdon, England). 1999; 94(8):1227-1237	non pertinent
8086	Winblad et al.,	1999	International j of geriatric psychiatry. 1999; 14(2):135-146	non pertinent
8087	Wilder-Smith et al.,	1999	British j of anaesthesia. 1999; 83(4):590-595	non pertinent
8088	Wakakura et al.,	1999	Jpn J Ophthalmol. 1999; 43(2):127-132	non pertinent
8089	Valenzuela et al.,	1999	Anesth Analg. 1999; 88(5):1107-1108	non pertinent
8090	Tajti et al.,	1999	Neurobiology (budapest, hungary). 1999; 7(2):103-108	non pertinent
8091	Suri et al.,	1999	Clinical pharmacokinetics. 1999; 37(SUPPL 2):61-68	non pertinent
8092	Stein et al.,	1999	Pain. 1999; 83(3):525-532	non pertinent
8093	Staszewski et al.,	1999	International j of clinical practice. Supplement. 1999; 103:10-15	non pertinent
8094	Sramek et al.,	1999	Clinical drug investigation. 1999; 17(2):137-144	non pertinent
8095	Spruance et al.,	1999	J of infectious diseases. 1999; 179(2):303-310	non pertinent
8096	Smith et al.,	1999	J of psychopharmacology (Oxford, England). 1999; 13(2):148-151	non pertinent
8097	Segerstrom et al.,	1999	Brain Behav Immun. 1999; 13(2):80-92	non pertinent
8098	Sechter et al.,	1999	European psychiatry. 1999; 14(1):41-48	non pertinent
8099	Schuitmaker et al.,	1999	Anaesthesia and intensive care. 1999; 27(6):615-622	non pertinent
8100	Schnitzer et al.,	1999	Clinical therapeutics. 1999; 21(10):1688-1702	non pertinent
8101	Schmitz et al.,	1999	Hernia. 1999; 3(3):125-129	non pertinent
8102	Schaffler et al.,	1999	Arzneimittelforschung. 1999; 49(4):374-379	non pertinent

8103	Salminen et al.,	1999	Spine (Phila Pa 1976). 1999; 24(13):1316-1321	non pertinent
8104	Sakai et al.,	1999	Acta Anaesthesiol Scand. 1999; 43(2):212-216	non pertinent
8105	Rossi et al.,	1999	Brain Res. 1999; 818(1):12-22	non pertinent
8106	Rosen et al.,	1999	J Cardiovasc Pharmacol. 1999; 34(4):554-560	non pertinent
8107	Radanov et al.,	1999	J Neurol Neurosurg Psychiatry. 1999; 66(4):485-489	non pertinent
8108	Rabben et al.,	1999	J of pharmacology and experimental therapeutics. 1999; 289(2):1060-1066	non pertinent
8109	Ploghaus et al.,	1999	Science. 1999; 284(5422):1979-1981	non pertinent
8110	Pivik et al.,	1999	Sleep Res Online. 1999; 2(4):89-100	non pertinent
8111	Pisegna et al.,	1999	American j of gastroenterology. 1999; 94(10):2874-2880	non pertinent
8112	Peichl et al.,	1999	Gynecological endocrinology. 1999; 13(1):7-14	non pertinent
8113	Pauli et al.,	1999	Pain. 1999; 80(1-2):359-364	non pertinent
8114	Patel et al.,	1999	European heart j. 1999; 20(1):51-57	non pertinent
8115	Patat et al.,	1999	Fundam Clin Pharmacol. 1999; 13(5):582-594	non pertinent
8116	Papa et al.,	1999	Academic medicine. 1999; 74(10 Suppl):16-18	non pertinent
8117	Overgaard et al.,	1999	Biol Neonate. 1999; 75(5):279-284	non pertinent
8118	Orhan et al.,	1999	Experimental and toxicologic pathology. 1999; 51(4-5):403-408	non pertinent
8119	Nichols et al.,	1999	J of antimicrobial chemotherapy. 1999; 44(2):263-273	non pertinent
8120	Muller et al.,	1999	Hormone research. 1999; 51(SUPPL 3):109-112	non pertinent
8121	Mossner	1999	Surgical clinics of north america. 1999; 79(4):861-872	non pertinent
8122	Morley et al.,	1999	Pain. 1999; 80(1-2):1-13	non pertinent

8123	Monnerat et al.,	1999	Praxis. 1999; 88(5):178-188	non pertinent
8124	Metaxotos et al.,	1999	British j of plastic surgery. 1999; 52(4):290-293	non pertinent
8125	Mahaffey et al.,	1999	Circulation. 1999; 99(18):2371-2377	non pertinent
8126	Maclsaac et al.,	1999	Obstetrics and gynecology. 1999; 93(5 Pt 1):766-770	non pertinent
8127	Loose et al.,	1999	Neurogastroenterol Motil. 1999; 11(3):163-171	non pertinent
8128	Liu et al.,	1999	Anesthesiology. 1999; 90(3):727-733	non pertinent
8129	List et al.,	1999	J Clin Oncol. 1999; 17(3):1020-1028	non pertinent
8130	Liénard et al.,	1999	Melanoma research. 1999; 9(5):491-502	non pertinent
8131	Lentz et al.,	1999	J Rheumatol. 1999; 26(7):1586-1592	non pertinent
8132	Lee	1999	Epilepsia. 1999; 40(12):1767-1774	non pertinent
8133	Lebovits et al.,	1999	Br J Anaesth. 1999; 82(6):861-866	non pertinent
8134	Lauritsen et al.,	1999	J canadien de gastroenterologie (Canadian j of gastroenterology). 1999; 13(10):806-813	non pertinent
8135	Kurtis et al.,	1999	Pediatrics. 1999; 103(2):E23	non pertinent
8136	Koswig et al.,	1999	Strahlentherapie und Onkologie. 1999; 175(10):500-508	non pertinent
9137	Knost et al.,	1999	Psychophysiology. 1999; 36(6):755-764	non pertinent
8138	Kaminester et al.,	1999	J of the American Academy of Dermatology. 1999; 41(6):996-1001	non pertinent
8139	Jones et al.,	1999	British j of general practice. 1999; 49(438):39-41	non pertinent
8140	Janiszewski et al.,	1999	Anesthesia and Analgesia. 1999; 88(5):1149-1154	non pertinent
8141	Jain et al.,	1999	Contraception. 1999; 60(6):353-356	non pertinent
8142	Howard et al.,	1999	American j of obstetrics and gynecology. 1999; 181(6):1506-1511	non pertinent

8143	Holas et al.,	1999	European j of anaesthesiology. 1999; 16(11):741-748	non pertinent
8144	Hirota et al.,	1999	Eur J Anaesthesiol. 1999; 16(8):516-518	non pertinent
8145	Heeschen et al.,	1999	Lancet. 1999; 354(9192):1757-1762	non pertinent
8146	Haubrich et al.,	1999	AIDS (london, england). 1999; 13(17):2411-2420	non pertinent
8147	Harms et al.,	1999	Fetal diagnosis and therapy. 1999; 14(6):368-374	non pertinent
8148	Harker et al.,	1999	Drug safety. 1999; 21(4):325-335	non pertinent
8149	Grillon et al.,	1999	Int J Psychophysiology. 1999; 32(1):63-73	non pertinent
8150	Goldenberg	1999	Clinical therapeutics. 1999; 21(2):309-318	non pertinent
8151	Gautier et al.,	1999	Am J Clin Nutr. 1999; 70(5):806-810	non pertinent
8152	Garcia-Larrea et al.,	1999	Pain. 1999; 83(2):259-273	non pertinent
8153	Fullerton et al.,	1999	J Clin Pharmacol. 1999; 39(1):17-29	non pertinent
8154	Fukumoto et al.,	1999	Lancet (london, england). 1999; 354(9192):1790-1791	non pertinent
8155	Fredman et al.,	1999	J of clinical anesthesia. 1999; 11(8):635-640	non pertinent
8156	Ettinger et al.,	1999	J of the american medical association. 1999; 282(7):637-645	non pertinent
8157	Emery et al.,	1999	Lancet (london, england). 1999; 354(9196):2106-2111	non pertinent
8158	Elie et al.,	1999	J of clinical psychiatry. 1999; 60(8):536-544	non pertinent
8159	Ehrich et al.,	1999	J of rheumatology. 1999; 26(11):2438-2447	non pertinent
8160	Dziadzio et al.,	1999	Arthritis and rheumatism. 1999; 42(12):2646-	non pertinent
8161	Doyle et al.,	1999	Alimentary pharmacology & therapeutics. 1999; 13(7):897-906	non pertinent

8162	Dell'Anna et al.,	1999	European review for medical and pharmacological sciences. 1999; 3(3):105-110	non pertinent
8163	De Wit et al.,	1999	J of international medical research. 1999; 27(5):223-232	non pertinent
8164	De Gelder et al.,	1999	Neuroreport. 1999; 10(18):3759-3763	non pertinent
7165	Cunningham et al.,	1999	Seminars in oncology. 1999; 26(1 SUPPL 5):6-12	non pertinent
8166	Cooper et al.,	1999	Osteoporosis international. 1999; 9(4):358-366	non pertinent
8167	Casati et al.,	1999	European j of anaesthesiology. 1999; 16(11):784-789	non pertinent
8168	Casati et al.,	1999	Anesthesiology. 1999; 90(4):1047-1052	non pertinent
8169	Carreno et al.,	1999	Hepatology (baltimore, md.). 1999; 30(1):277-282	non pertinent
8170	Calabrese et al.,	1999	J of clinical psychiatry. 1999; 60(2):79-88	non pertinent
8171	Burke et al.,	1999	Clinical therapeutics. 1999; 21(10):1664-1677	non pertinent
8172	Bultmann et al.,	1999	Der Anaesthesist. 1999; 48(7):439-443	non pertinent
8173	Brenner et al.,	1999	European j of applied physiology and occupational physiology. 1999; 80(5):452-460	non pertinent
8174	Breen et al.,	1999	International j of obstetric anesthesia. 1999; 8(4):226-230	non pertinent
8175	Bramer et al.,	1999	Clinical pharmacokinetics. 1999; 37 Suppl 2:41-51	non pertinent
8176	Bramer et al.,	1999	Clinical pharmacokinetics. 1999; 37 Suppl 2:1-11	non pertinent
8177	Biswas et al.,	1999	Eye (london, england). 1999; 13(4):537-540	non pertinent
8178	Benedetti et al.,	1999	Pain. 1999; 80(1-2):377-382	non pertinent

8179	Benedetti et al.,	1999	J of neuroscience. 1999; 19(9):3639-3648	non pertinent
8180	Benedetti et al.,	1999	European j of neuroscience. 1999; 11(2):625-631	non pertinent
8181	Ben-Ari et al.,	1999	J Pers Soc Psychol. 1999; 76(1):35-45	non pertinent
8182	Baird et al.,	1999	J Am Acad Child Adolesc Psychiatry. 1999; 38(2):195-199	non pertinent
8183	Atanassoff et al.,	1999	Somatosens Mot Res. 1999; 16(4):291-298	non pertinent
8184	Apkarian et al.,	1999	J Neurophysiol. 1999; 81(6):2956-2963	non pertinent
8185	Antonicelli et al.,	1999	American j of medicine. 1999; 107(3):234-239	non pertinent
8186	Alvarez et al.,	1999	Prensa medica argentina. 1999; 86(7):698-705	non pertinent
8187	Albrecht et al.,	1999	Der Anaesthesist. 1999; 48(11):794-801	non pertinent
8188	Agid et al.,	1999	Movement disorders. 1999; 14(1):38-44	non pertinent
8189	Adolf et al.,	1999	International angiology. 1999; 18(2):122-126	non pertinent
8190	Thimineur et al.,	1998	Clin J Pain. 1998; 14(3):256-267	non pertinent
8191	Terrell et al.,	1998	Arch Otolaryngol Head Neck Surg. 1998; 124(9):964-971	non pertinent
8192	Srivastava et al.,	1998	Clinical cancer research. 1998; 4(1):61-68	non pertinent
8193	Spencer et al.,	1998	British j of radiology. 1998; 71(851):1130-1135	non pertinent
8194	Song et al.,	1998	Anesth Analg. 1998; 87(6):1245-1248	non pertinent
8195	Silverst et al.,	1998	Clinical therapeutics. 1998; 20(3):438-453	non pertinent
8196	Serour et al.,	1998	Acta anaesthesiologica Scandinavica. 1998; 42(2):260-263	non pertinent
8197	Sellick et al.,	1998	Cancer prevention and control. 1998; 2(1):7-14	non pertinent

8198	Saito et al.,	1998	International j of immunotherapy. 1998; 14(2):115-128	non pertinent
8199	Rampil et al.,	1998	Anesthesiology. 1998; 89(3):671-677	non pertinent
8200	Porro et al.,	1998	J of neurophysiology. 1998; 80(6):3312-3320	non pertinent
8201	Pippi Salle et al.,	1998	J Urol. 1998; 160(3 Pt 2):1080-1083	non pertinent
8202	Ping et al.,	1998	Chinese j of antibiotics. 1998; 23(4):315-318	non pertinent
8203	Peduto et al.,	1998	Acta anaesthesiologica Scandinavica. 1998; 42(3):293-298	non pertinent
8204	Payne et al.,	1998	J Clin Oncol. 1998; 16(4):1588-1593	non pertinent
8205	Paulson et al.,	1998	Pain. 1998; 76(1-2):223-229	non pertinent
8206	Oshiro et al.,	1998	Neuroreport. 1998; 9(10):2285-2289	non pertinent
8207	Olausson	1998	Exp Brain Res. 1998; 122(1):44-54	non pertinent
8208	Okhuysen et al.,	1998	Clinical infectious diseases. 1998; 26(6):1324-1329	non pertinent
8209	Nishiyama et al.,	1998	J canadien d'anesthesie (Canadian j of anaesthesia). 1998; 45(6):551-555	non pertinent
8210	Netzer et al.,	1998	Alimentary pharmacology & therapeutics. 1998; 12(4):337-342	non pertinent
8211	Muller et al.,	1998	Arzneimittelforschung/drug research. 1998; 48(6):675-679	non pertinent
8212	Michalski	1998	Acta Neurobiol Exp (Wars). 1998; 58(1):55-64	non pertinent
8213	May et al.,	1998	Pain. 1998; 74(1):61-66	non pertinent
8214	Malaguarnera et al.,	1998	International j of clinical pharmacology and therapeutics. 1998; 36(8):441-445	non pertinent
8215	Lotsch et al.,	1998	Chem Senses. 1998; 23(6):755-759	non pertinent

8216	Lorenz et al.,	1998	Neuroreport. 1998; 9(2):187-191	non pertinent
8217	Kondziolka et al.,	1998	Arch Neurol. 1998; 55(12):1524-1529	non pertinent
8218	Jones et al.,	1998	Human psychopharmacology. 1998; 13(1):35-42	non pertinent
8219	Iselin-Chaves et al.,	1998	Anesthesia and Analgesia. 1998; 87(4):949-955	non pertinent
8220	Iadarola et al.,	1998	Brain. 1998; 121(Pt 5):931-947	non pertinent
8221	Hauser et al.,	1998	Movement disorders. 1998; 13(4):643-647	non pertinent
8222	Grillon et al.,	1998	Int J Psychophysiol. 1998; 28(3):223-231	non pertinent
8223	Green et al.,	1998	Somatosens Mot Res. 1998; 15(4):269-275	non pertinent
8224	Goodarzi	1998	Paediatr Anaesth. 1998; 8(2):131-134	non pertinent
8225	Gill et al.,	1998	Spine (Phila P 1976). 1998; 23(3):371-377	non pertinent
8226	Furuya et al.,	1998	J Neurol Neurosurg Psychiatry. 1998; 64(2):221-226	non pertinent
8227	Fu et al.,	1998	Anesthesia and Analgesia. 1998; 86(5):1127-1130	non pertinent
8228	Drewes et al.,	1998	Scand J Rheumatol. 1998; 27(3):180-187	non pertinent
8229	Disbrow et al.,	1998	Hum Brain Mapp. 1998; 6(3):150-159	non pertinent
8230	Cunningham	1998	British j of cancer. 1998; 77(SUPPL 2):15-21	non pertinent
8231	Coetzee et al.,	1998	Br J Anaesth. 1998; 81(5):737-741	non pertinent
8232	Choi et al.,	1998	Korean j of anesthesiology. 1998; 34(2):353-358	non pertinent
8233	Chey et al.,	1998	Alimentary pharmacology & therapeutics. 1998; 12(12):1263-1267	non pertinent
8234	Carpentier et al.,	1998	J des maladies vasculaires. 1998; 23(2):106-112	non pertinent

8235	Carling et al.,	1998	Alimentary pharmacology & therapeutics. 1998; 12(10):985-990	non pertinent
8236	Brough et al.,	1998	European j of nuclear medicine. 1998; 25(11):1520-1523	non pertinent
8237	Boneau et al.,	1998	Thrombosis and haemostasis. 1998; 79(2):338-341	non pertinent
8238	Bjorneboe et al.,	1998	Scandinavian j of rheumatology. 1998; 27(1):26-31	non pertinent
8239	Bischoff et al.,	1998	Anasthesiologie, Intensivmedizin, Notfallmedizin, Schmerztherapie. 1998; 33(2):88-95	non pertinent
8240	Bessette et al.,	1998	Scandinavian j of rheumatology. 1998; 27(2):112-116	non pertinent
8241	Balderston et al.,	1998	Spine (Phila Pa 1976). 1998; 23(1):54-58	non pertinent
8242	Williams et al.,	1997	Clinical radiology. 1997; 52(1):46-49	non pertinent
8243	Van Tulder et al.,	1997	Spine. 1997; 22(18):2128-2156	non pertinent
8244	Svensson et al.,	1997	J Neurophysiol. 1997; 78(1):450-460	non pertinent
8245	Sinczuk-Walczak et al.,	1997	International j of occupational medicine and environmental health. 1997; 10(4):429-440	non pertinent
8246	Sengoku et al.,	1997	Psychiatry Clin Neurosci. 1997; 51(1):23-26	non pertinent
8247	Schüttler et al.,	1997	Anaesthesia. 1997; 52(4):307-317	non pertinent
8248	Sandman et al.,	1997	Am J Ment Retard. 1997; 102(2):182-199	non pertinent
8249	Rundshagen et al.,	1997	Anasthesiologie, Intensivmedizin, Notfallmedizin, Schmerztherapie. 1997; 32(10):604-609	non pertinent
8250	Romero Ganuza et al.,	1997	Spinal Cord. 1997; 35(2):124-128	non pertinent
8251	Rizzato et al.,	1997	Respiratory medicine. 1997; 91(8):449-460	non pertinent
8252	Pinto et al.,	1997	J of aging and physical activity. 1997; 5(4):311-328	non pertinent

8253	Persson et al.,	1997	Eur Spine J. 1997; 6(4):256-266	non pertinent
8254	Perlis et al.,	1997	Int J Neurosci. 1997; 89(3-4):265-280	non pertinent
8255	Motsch et al.,	1997	Acta anaesthesiologica Scandinavica. 1997; 41(7):877-883	non pertinent
8256	Mjahed et al.,	1997	Annales francaises d'anesthesie ET de reanimation. 1997; 16(5):488-491	non pertinent
8257	Matsushima et al.,	1997	Biol Psychiatry. 1997; 41(12):1211-1217	non pertinent
8258	Lukins et al.,	1997	J Behav Ther Exp Psychiatry. 1997; 28(2):97-104	non pertinent
8259	Lotsch et al.,	1997	Eur J CLin Pharmacol. 1997; 52(5):359-364	non pertinent
8260	Loo et al.,	1997	Annals of the academy of medicine singapore. 1997; 26(2):200-204	non pertinent
8261	Likar et al.,	1997	Der Anaesthesist. 1997; 46(3):186-190	non pertinent
8262	Li et al.,	1997	IOVS. 1997; 38:ARVO Abstract 4515	non pertinent
8263	Lewis et al.,	1997	Neurology. 1997; 48(6):1542-1550	non pertinent
8264	Lerman et al.,	1997	Health psychology. 1997; 16(1):87-99	non pertinent
8265	Kosuge et al.,	1997	Br J Clin Pharmacol. 1997; 43(4):367-372	non pertinent
8266	Knott et al.,	1997	J Anxiety Disord. 1997; 11(4):365-376	non pertinent
8267	Jarvik et al.,	1997	Radiology. 1997; 204(2):447-454	non pertinent
8268	Hummel et al.,	1997	European j of clinical pharmacology. 1997; 52(2):107-114	non pertinent
8269	Herrick et al.,	1997	Anesth Analg. 1997; 84(6):1285-1291	non pertinent
8270	Harte et al.,	1997	J Paediatr Child Health. 1997; 33(4):335-338	non pertinent
8271	Gutierrez et al.,	1997	Abstract book/association for health services research. 1997; 14:105-106	non pertinent

8272	Grillon et al.,	1997	Psychophysiology. 1997; 34(5):511-517	non pertinent
8273	Greenspan et al.,	1997	Pain. 1997; 72(1-2):13-25	non pertinent
8274	Grant et al.,	1997	Pharmacotherapy. 1997; 17(2):333-341	non pertinent
8275	Gobel et al.,	1997	Drugs. 1997; 53(SUPPL 2):34-39	non pertinent
8276	Garcia-Losa	1997	Medicina clinica. 1997; 109(4):130-134	non pertinent
8277	Garcia et al.,	1997	Fundamental & clinical pharmacology. 1997; 11(5):466-475	non pertinent
8278	Furmark et al.,	1997	Neuroreport. 1997; 8(18):3957-3960	non pertinent
8279	Freeborn et al.,	1997	J Gen Intern Med. 1997; 12(10):619-625	non pertinent
8280	Flor et al.,	1997	Pain. 1997; 73(3):413-421	non pertinent
8281	Fleischmann et al.,	1997	Clinical therapeutics. 1997; 19(4):642-655	non pertinent
8282	Ellrich et al.,	1997	Brain Res. 1997; 764(1-2):214-220	non pertinent
8283	Eisenach et al.,	1997	Anesthesiology. 1997; 86(6):1279-1287	non pertinent
8284	Doenicke et al.,	1997	Anesth Analg. 1997; 85(6):1399-1403	non pertinent
8285	Derbyshire et al.,	1997	Pain. 1997; 73(3):431-445	non pertinent
8286	Chareonthaitawee et al.,	1997	American heart j. 1997; 134(4):639-646	non pertinent
8287	Cannoni et al.,	1997	Medecine ET maladies infectieuses. 1997; 27(11):915-921	non pertinent
8288	Buzelin et al.,	1997	British j of urology. 1997; 80(4):597-605	non pertinent
8289	Bono et al.,	1997	Drugs. 1997; 53(SUPPL 2):40-49	non pertinent
8290	Basler et al.,	1997	Patient education and counseling. 1997; 31(2):113-124	non pertinent
8291	Angrilli et al.,	1997	Physiol Behav. 1997; 62(2):391-397	non pertinent
8292	Amr et al.,	1997	Environ Res. 1997; 73(1-2):200-206	non pertinent

8293	Adler et al.,	1997	Anesth Analg. 1997; 84(1):120-126	non pertinent
8294	Zaslansky et al.,	1996	Electroencephalogr Clin Neurophysiol. 1996; 100(5):384-391	non pertinent
8295	Vogt et al.,	1996	Eur J Neurosci. 1996; 8(7):1461-1473	non pertinent
8296	Turk et al.,	1996	J of consulting and clinical psychology. 1996; 64(1):139-146	non pertinent
8297	Thurauf et al.,	1996	Br J Clin Pharmacol. 1996; 41(2):115-123	non pertinent
8298	Tanaka et al.,	1996	British j of haematology. 1996; 92(4):795-803	non pertinent
8299	Sviderskaya et al.,	1996	Neurosci Behav Physiol. 1996; 26(6):532-538	non pertinent
8300	Studd et al.,	1996	Clinical drug investigation. 1996; 11(4):205-213	non pertinent
8301	Stanley et al.,	1996	J of nervous and mental disease. 1996; 184(9):567-572	non pertinent
8302	Sartori et al.,	1996	Cancer. 1996; 78(7):1477-1482	non pertinent
8303	Sansom et al.,	1996	Clinical and experimental dermatology. 1996; 21(1):33-37	non pertinent
8304	Portier et al.,	1996	Medecine ET madalies infectieuses. 1996; 26(12):1188-1193	non pertinent
8305	Petersen-Felix et al.,	1996	British j of anaesthesia. 1996; 77(2):165-171	non pertinent
8306	Pedersen et al.,	1996	Anesthesiology. 1996; 84(5):1020-1026	non pertinent
8307	Parker et al.,	1996	Spine (Phila Oa 1976). 1996; 21(16):1909-1916	non pertinent
8308	Nikolajsen et al.,	1996	Pain. 1996; 67(1):69-77	non pertinent
8309	McNamara et al.,	1996	Australian dental j. 1996; 41(6):377-387	non pertinent
8310	Madawi et al.,	1996	Spine. 1996; 21(18):2123-2129	non pertinent
8311	Louvel et al.,	1996	Gut. 1996; 39(5):741-747	non pertinent
8312	Liu et al.,	1996	Anesthesiology. 1996; 84(1):64-69	non pertinent

8313	Kubin et al.,	1996	J of photochemistry and photobiology B: biology. 1996; 36(2):103-108	non pertinent
8314	Krittayaphong et al.,	1996	Clin J Pain. 1996; 12(2):126-133	non pertinent
8315	Kostyunina et al.,	1996	Neurosci Behav Physiol. 1996; 26(4):340-343	non pertinent
8316	Kochs et al.,	1996	Anesthesiology. 1996; 85(2):304-314	non pertinent
8317	Kinouchi et al.,	1996	J of pharmacy and pharmacology. 1996; 48(3):310-315	non pertinent
8318	Hsieh et al.,	1996	Pain. 1996; 64(2):303-314	non pertinent
8319	Hommel et al.,	1996	Dermatology (basel, switzerland). 1996; 193(2):127-130	non pertinent
8320	Hetem et al.,	1996	Psychopharmacology. 1996; 127(3):276-282	non pertinent
8321	Goodwin	1996	Clinical immunotherapeutics. 1996; 6(5):405-412	non pertinent
8322	Firestone et al.,	1996	Anesthesia and Analgesia. 1996; 82(6):1247-1251	non pertinent
8323	Dowman	1996	Psychophysiology. 1996; 33(4):398-408	non pertinent
8324	Dawson et al.,	1996	J of cranio-maxillo-facial surgery. 1996; 24(3):151-154	non pertinent
8325	Cleeland et al.,	1996	Neuropsychopharmacology. 1996; 15(3):252-262	non pertinent
8326	Casey et al.,	1996	J of neurophysiology. 1996; 76(1):571-581	non pertinent
8327	Anton et al.,	1996	Alcohol Alcohol. 1996; 31 Suppl 1:43-53	non pertinent
8328	Kieburtz et al.,	1996	J of neuro-aids. 1996; 1(4):1-5	non pertinent
8329	Vlaeyen et al.,	1995	The british j of clinical psychology. 1995; 34(1):95-118	non pertinent
8330	Vestergaard et al.,	1995	Pain. 1995; 61(2):177-186	non pertinent
8331	Uchida et al.,	1995	Clinical therapeutics. 1995; 17(3):460-466	non pertinent

8332	Taniguchi et al.,	1995	Acta anaesthesiologica belgica. 1995; 46(2):69-73	non pertinent
8333	Schonfeld et al.,	1995	Pediatrics. 1995; 95(4):480-486	non pertinent
8334	Patat et al.,	1995	J of clinical pharmacology. 1995; 35(6):633-643	non pertinent
8335	Ono et al.,	1995	Japanese j of chemotherapy. 1995; 43(2):222-230	non pertinent
8336	Nordin et al.,	1995	J of physiology. 1995; 489(3):885-894	non pertinent
8337	Nienaber et al.,	1995	Circulation. 1995; 92(6):1465-1472	non pertinent
8338	Morgan et al.,	1995	Biol Psychiatry. 1995; 38(6):378-385	non pertinent
8339	Mini et al.,	1995	Int J Psychophysiol. 1995; 19(1):67-77	non pertinent
8340	Lötsch et al.,	1995	British j of pharmacology. 1995; 40(6):545-552	non pertinent
8341	Isaia et al.,	1995	Minerva medica. 1995; 86(3):101-104	non pertinent
8342	Ilyuchina et al.,	1995	Alcohol (Fayetteville, N.Y.). 1995; 12(6):511-517	non pertinent
8343	Hartvig et al.,	1995	Clin Pharmacol The. 1995; 58(2):165-173	non pertinent
8344	Gronemeyer et al.,	1995	Wiener medizinische wochenschrift (1946). 1995; 145(6):129-139	non pertinent
8345	Goddard et al.,	1995	Biol Psychiatry. 1995; 38(2):74-85	non pertinent
8346	Gamzu Elkan Pradalier et al.,	1995	8th-ecnp-(european-college-of-neuropsychopharmacology)-congress,-venice,-italy: pathologie biologie. 1995; 43(9):806-813	non pertinent
8347	Frederikson et al.,	1995	Psychophysiology. 1995; 32(1):43-48	non pertinent
8348	Foy et al.,	1995	European heart j. 1995; 16(6):770-778	non pertinent
8349	Devulder et al.,	1995	Clin J Pain. 1995; 11(2):147-150	non pertinent
8350	Daidsson et al.,	1995	Current medical research and opinion. 1995; 13(5):285-297	non pertinent

8351	Canavero et al.,	1995	J Neurol. 1995; 242(9):561-567	non pertinent
8352	Cagnolati et al.,	1995	Revista brasileira de anestesiologia. 1995; 45(4):215-223	non pertinent
8353	Bruck et al.,	1995	Child development. 1995; 66(1):193-208	non pertinent
8354	Bromm et al.,	1995	Electroencephalography and clinical neurophysiology. 1995; 95(1):14-26	non pertinent
8355	Bergmann et al.,	1995	Gastroenterologie clinique ET biologique. 1995; 19(5):482-486	non pertinent
8356	Bergdahl et al.,	1995	J of oral pathology & medicine. 1995; 24(5):213-215	non pertinent
8357	Bakker et al.,	1995	J Rheumatol. 1995; 22(7):1304-1310	non pertinent
8358	Arnold	1995	J of abnormal child physiology. 1995; 23(1):125-140	non pertinent
8359	Aldrete et al.,	1995	Reg Anesth. 1995; 20(1):75-79	non pertinent
8360	No authors listed	1995	Advances in therapy. 1995; 12(5):313-320	non pertinent
8361	Wagner et al.,	1994	Acta Radiol. 1994; 35(2):182-185	non pertinent
8362	Van Dyck et al.,	1994	Psychiatry research. 1994; 55(4):181-191	non pertinent
8363	Triano et al.,	1994	J of manipulative and physiological therapeutics. 1994; 4(17):291	non pertinent
8364	Thurauf et al.,	1994	Br J Clin Pharmacol. 1994; 38(6):545-555	non pertinent
8365	Shima et al.,	1994	Japanese pharmacology and therapeutics. 1994; 22(SUPPL 4):75-95	non pertinent
8366	Satku et al.,	1994	Annals of the academy of medicine, singapore. 1994; 23(6):828-831	non pertinent
8367	Post et al.,	1994	Ep Brain Res. 1994; 100(1):107-120	non pertinent
8368	Pfenninger et al.,	1994	Der Anaesthesist. 1994; 43(Suppl 2):68-75	non pertinent

8369	McCann et al.,	1994	Anxiety. 1994; 1(6):258-267	non pertinent
8370	Lucker et al.,	1994	International j of clinical pharmacology and therapeutics. 1994; 32(8):409-414	non pertinent
8371	Kollerup et al.,	1994	Calcified tissue international. 1994; 54(1):12-15	non pertinent
8372	Kempenaers et al.,	1994	Neuropsychobiology. 1994; 30(2-3):66-72	non pertinent
8373	Ilacqua	1994	Headache. 1994; 34(2):99-102	non pertinent
8374	Hotta et al.,	1994	Clinical therapeutics. 1994; 16(6):1007-1015	non pertinent
8375	Hilz et al.,	1994	Muscle Nerve. 1994; 17(12):1456-1460	non pertinent
8376	He et al.,	1994	Pain. 1994; 59(1):55-63	non pertinent
8377	Hasenfratz et al.,	1994	Psychopharmacology. 1994; 114(2):281-287	non pertinent
8378	Guinsburg et al.,	1994	J de pediatra. 1994; 70(2):82-90	non pertinent
8379	Girardi et al.,	1994	Minerva Anesthesiologica. 1994; 60(6):321-328	non pertinent
8380	Fredman et al.,	1994	Anesth Analg. 1994; 79(1):75-79	non pertinent
8381	Dordoni et al.,	1994	Anaesthesia. 1994; 49(12):1046-1049	non pertinent
8382	Doenicke et al.,	1994	Anesth Analg. 1994; 79(5):933-939	non pertinent
8383	De et al.,	1994	Annales medicales de nancy ET de l'est. 1994; 33(2):113-117	non pertinent
8384	Davis et al.,	1994	Pain. 1994; 59(2):189-199	non pertinent
8385	Creamer et al.,	1994	Osteoarthritis Cartilage. 1994; 2(2):133-140	non pertinent
8386	Brückle et al.,	1994	Clinical rheumatology. 1994; 13(2):209-216	non pertinent
8387	Blyth et al.,	1994	British j of rheumatology. 1994; 33(5):461-463	non pertinent
8388	Beck et al.,	1994	J of consulting and clinical psychology. 1994; 62(4):818-826	non pertinent

8389	Vaupel et al.,	1993	J Pharmacol Exp Ther. 1993; 267(3):1386-1394	non pertinent
8390	Schwender et al.,	1993	Der Anaesthesist. 1993; 42(9):583-591	non pertinent
8391	Rossi et al.,	1993	Rivista di neuroradiologia. 1993; 6(4):445-452	non pertinent
8392	Peduzzi et al.,	1993	Stat Med. 1993; 12(13):1185-1195	non pertinent
8393	Mössner	1993	Digestion. 1993; 54 Suppl 2:35-39	non pertinent
8394	Morse et al.,	1993	Int J Psychosom. 1993; 40(1-4):68-76	non pertinent
8395	Morgan et al.,	1993	Psychopharmacology (Berl). 1993 110(3):342-346	non pertinent
8396	King et al.,	1993	Anesthesia and Analgesia. 1993; 77(1)84-88	non pertinent
8397	Jerosch et al.,	1993	Knee surgery, sports traumatology, arthroscopy. 1993; 1(2):80-84	non pertinent
8398	Hasenfratz et al.,	1993	psychopharmacology. 1993; 113(1):37-44	non pertinent
8399	Goddard et al.,	1993	Psychiatry Res. 1993; 48(2):119-133	non pertinent
8400	Borkovec et al.,	1993	Behavior research and therapy. 1993; 31(3):321-324	non pertinent
8401	Bischoff et al.,	1993	Der Anaesthesist. 1993; 42(3):142-148	non pertinent
8402	Wigley et al.,	1992	J of rheumatology. 1992; 19(9):1407-1414	non pertinent
8403	Veerasarn et al.,	1992	Pain. 1992; 49(3):349-360	non pertinent
8404	Sevarino et al.,	1992	J Clin Anesth. 1992; 4(4):285-288	non pertinent
8405	Pietrowsky et al.,	1992	Psychopharmacology. 1992; 107(1):50-54	non pertinent
8406	Mazieres et al.,	1992	Rev rheum mal osteo-articulares. 1992; 59(7-8):466-472	non pertinent
8407	Maier et al.,	1992	Der radiologe. 1992; 32(8):393-396	non pertinent
8408	Klement et al.,	1992	Pain. 1992; 48(2):269-274	non pertinent

8409	De Vlieger et al.,	1992	Diagnostic microbiology and infectious disease. 1992; 15(4 Suppl):123-127	non pertinent
8410	Choi et al.,	1992	Taehan kanho (Korean nurse). 1992; 31(4):77-99	non pertinent
8411	Aranda et al.,	1992	Arzneim.-Forsch. Drug res. 42(11 A):1406-1409	non pertinent
8412	Woods et al.,	1991	Psychiatry Res. 1991; 36(2):115-127	non pertinent
8413	Treves et al.,	1991	Rev rhum mal osteo-articulaires. 1991; 58(7):549-552	non pertinent
8414	Sinatra et al.,	1991	J Clin Anesth. 1991; 3(3):219-224	non pertinent
8415	Sienra-Pérez et al.,	1991	Archivos del instituto de cardiologia de mexico. 1991; 61(4):365-373	non pertinent
8416	Sevarino et al.,	1991	Can J Anaesth. 1991; 38(4 Pt 1):450-453	non pertinent
8417	Russell et al.,	1991	Behaviour research and therapy. 1991; 29(2):191-196	non pertinent
8418	Reynolds et al.,	1991	J Rheumatol. 1991; 18(3):452-454	non pertinent
8419	Pochobradsky et al.,	1991	Drugs under experimental and clinical research. 1991; 17(3):197-204	non pertinent
8420	Loubser et al.,	1991	Paraplegia. 1991; 29(1):25-36	non pertinent
8421	Heller et al.,	1991	Medical j of Australia. 1991; 155(5):329-332	non pertinent
8422	Grabow et al.,	1991	Klinische wochenschrift. 1991; 69 Suppl 26:100-103	non pertinent
8423	Floris et al.,	1991	Int j clin pharmacol ther toxicol. 1991; 29(4):139-143	non pertinent
8424	El-Hadid et al.,	1991	J of international medical research. 1991; 19(3):219-227	non pertinent
8425	Cinnamon et al.,	1991	AJNR. American j of neuroradiology. 1991; 12(6):1099-1103	non pertinent
8426	Cherkin et al.,	1991	Spine. 1991; 16(10):1168-1172	non pertinent

8427	Cassell et al.,	1991	J of antimicrobial chemotherapy. 1991; 27(Suppl A):47-59	non pertinent
8428	Bromm et al.,	1991	Arzneimittel-forschung. 1991; 41(11):1123-1129	non pertinent
8429	Arendt-Nielsen et al.,	1991	Acta anaesthesiologica Scandinavica. 1991; 35(1):24-29	non pertinent
8430	Zachariae et al.,	1990	Archives of dermatological research. 1990; 282(8):539-543	non pertinent
8431	Yonemura et al.,	1990	Masui. The japanese j of anesthesiology. 1990; 39(4):478-486	non pertinent
8432	Wiedermann et al.,	1990	Vaccine. 1990; 8(6):581-584	non pertinent
8433	Hale et al.,	1990	Archives of sexual behavior. 1990; 19(6):569-581	non pertinent
8434	Goodman et al.,	1990	Arch Gen Psychiatry. 1990; 47(6):577-585	non pertinent
8435	Doenicke et al.,	1990	Der Anaesthesist. 1990; 39(10):475-480	non pertinent
8436	Copp et al.,	1990	J Clin Psychopharmacol. 1990; 10(3 Suppl):52-60	non pertinent
8437	Borkovec et al.,	1990	Behaviour research and therapy. 1990; 28(1):69-73	non pertinent
8438	Westenberg et al.,	1989	Psychopathology. 1989; 22 Suppl 1:68-77	non pertinent
8439	Valtonen	1989	Acta Anaesthesiol Scand. 1989; 33(2):170-173	non pertinent
8440	Stuttmann et al.,	1989	Der Anaesthesist. 1989; 38(8):421-423	non pertinent
8441	Smith et al.,	1989	Pediatrics. 1989; 84(2):290-295	non pertinent
8442	Ringe	1989	Clinical rheumatology. 1989; 8 Suppl 2; 109-115	non pertinent
8443	Portenoy et al.,	1989	Cancer. 1989; 63(11 Suppl):2284-2288	non pertinent
8444	Lichstein et al.,	1989	Behaviour research and therapy. 1989; 27(5):569-572	non pertinent
8445	Leigh et al.,	1989	Clinical rheumatology. 1989; 8(4):489-493	non pertinent

8446	Johnson et al.,	1989	Headache. 1989; 29(6):358-365	non pertinent
8447	Hauri et al.,	1989	Sleep. 1989; 12(4):323-337	non pertinent
8448	Egger et al.,	1989	J of pediatrics. 1989; 114(1):51-58	non pertinent
8449	Dettori et al.,	1989	Angiology. 1989; 40(4 Pt 1):237-248	non pertinent
8450	Charney et al.,	1989	Psychiatry Res. 1989; 27(2):173-182	non pertinent
8451	Arnold et al.,	1989	Fortschritte der medizin. 1989; 107(21):43-46	non pertinent
8452	Uhde et al.,	1988	Am J Psychiatry. 1988; 145(9):1104-1109	non pertinent
8453	Rösch	1988	Drugs. 1988; 35 Suppl 3:127-133	non pertinent
8454	Miltner et al.,	1988	Pain. 1988; 35(2):205-213	non pertinent
8455	Micieli et al.,	1988	Headache. 1988; 28(3):196-200	non pertinent
8456	Matejcek et al.,	1988	Neuropsychobiology. 1988; 19(4):202-211	non pertinent
8457	Valdes et al.,	1987	European j of neurology and inflammation. 1987; 9(1-2):133-136	non pertinent
8458	Pohl et al.,	1987	Biol Psychiatry. 1987; 22(9):1127-1136	non pertinent
8459	Jaffe et al.,	1987	International j of cardiology. 1987; 15(1):77-89	non pertinent
8460	Davidoff et al.,	1987	Pain. 1987; 29(2):151-161	non pertinent
8461	Szekely et al.,	1986	Headache. 1986; 26(2):86-92	non pertinent
8462	Knott et al.,	1986	Neuropsychobiology; 1986; 16(1):9-14	non pertinent
8463	Hijzen et al.,	1986	J of oral rehabilitation. 1986; 13(6):529-539	non pertinent
8464	Bromm et al.,	1986	Pain. 1986; 25(2):245-257	non pertinent
8465	Bromm et al.,	1986	Neuropsychobiology. 1986; 16(2-3):152-156	non pertinent
8466	Schwartz	1985	Am J Emerg Med. 1985; 3(1):1-10	non pertinent
8467	Saletu et al.,	1985	J Clin Psychiatry. 1985; 46(3 Pt 2):45-52	non pertinent
8468	Velasco et al.,	1984	Neuropharmacology. 1984; 23(8):931-938	non pertinent
8469	Thomas	1984	Aust NZ j med suppl. 1984; 14(3 Suppl 1):349	non pertinent

8470	Stacher et al.,	1983	Br J Clin Pharmacol. 1983; 16(2):149-156	non pertinent
8471	Mc Cauley et al.,	1983	Archives of physical medicine and rehabilitation. 1983; 64(11):548-552	non pertinent
8472	Large et al.,	1983	Pain. 1983; 17(2):167-177	non pertinent
8473	Janssen	1983	J Psychosom Res. 1983; 27(3):243-253	non pertinent
8474	Stacher et al.,	1982	Peptides. 1982; 3(6):955-962	non pertinent
8475	Feuerstein et al.,	1982	J-psychoso-res. 1982; 26(2):167-182	non pertinent
8476	Sebastian et al.,	1981	J of research in personality. 1981; 15(3)	non pertinent
8477	Willer et al.,	1980	Brain Res. 1980; 198(2):419-426	non pertinent
8478	Schlutter et al.,	1980	British j of medical psychology. 1980; 53(1):47-52	non pertinent
8479	Newman et al.,	1980	Cognitive therapy and research. 1980; 4(4):397-407	non pertinent
8480	Melzack et al.,	1980	Canadian medical association j. 1980; 2(122):189-191	non pertinent
8481	Melzack et al.,	1980	Pain. 1980; 9(2):209-217	non pertinent
8482	Stacher et al.,	1979	Pain. 1979; 7(2):159-172	non pertinent
