# Supplementary material for: Functional Changes in Brain Activity Using Hypnosis: A Systematic Review
Source: Brain Sci. 2022 Jan 13;12(1):108. doi: 10.3390/brainsci12010108 (PMC8773773; doi:10.3390/brainsci12010108)
Supplement: Supplementary file 1 [file brainsci-12-00108-s001.zip › 4_Table S2.suppl.Material_Qualityassessment.pdf]

Quality Assessment

ID	Author	Sources	Type of study	Method	Oral conditions	Quality Assessment
1	London et al.,	Nature. **1968**; 219(6):71-72	Observ. Study	EEG	No oral conditions	Fair
2	Hart	Nature. **1970**; 227(19):1261-1262	CCS	EEG	No oral conditions	Good
3	Morgan et al.,	Psychophysiology. **1974**; 11(3):275-	Observ. Study	EEG	No oral conditions	Poor
4	Tebecis et al.,	The J of Nervous and Mental Disease. **1975**; 161(1):1-16	CCS	EEG	No oral conditions	Fair
5	Zachariae et al.,	International J of Clinical and Experimental Hypnosis **1994**, 42(1), 56-80	CSS	Argon Laser	No oral conditions	Fair
6	Graffin et al.,	J of abnormal Psychology. **1995**; 104(1):123-131	Comparative Study	EEG	No oral conditions	Good
7	De Pascalis et al.,	Int J Psychophysiol **1996**, 21(1996), 163-175	CSS	EEG	No oral conditions	Fair
8	De Pascalis et al.,	Int J of Psychophysiology. **1998**; 29(1998):255-275	COS	EEG	No oral conditions	Fair
9	Maquet et al.,	Society of Biological Psychiatry. **1999**; 45, 327-333	Case-Control Study	PET	No oral conditions	Fair
10	Rainville et al.,	J of Cognitive Neuroscience. **1999**; 11(1):110-125	Observ. Study	EEG, PET	No oral conditions	Fair
11	Faymonville et al.,	Anesthesiology **2000**, 92(5), 1257-1267	CSS	PET	No oral conditions	Fair
12	Freeman et al.,	American J of Clinical Hypnosis **2000**, 43(2), 137-148	CSS	EEG	No oral conditions	Good
13	De Pascalis et al.,	Clin Neurophysiol **2001**, 112(8), 1475-1485	CCS	rCBF + EEG	No oral conditions	Good
14	Friedrich et al.,	Psychophysiology **2001**, 38(5), 768-	CSS	Thulium YAG Laser + EEG	No oral conditions	Fair
15	Isotani et al.,	Neuropsychobiology. **2001**; 44:192-198	Pilot Study	EEG	No oral conditions	Fair
16	Willumsen et al.,	Acta Odontol Scand **2001**, 59(6), 335-340	RCS	Cognitive Therapy	Dental fear	Good
17	De Pascalis et al.,	Pain. **2004**; 112:27-36	Observ. Cohort	EEG	No oral conditions	Good
18	Harandi et al.,	Contemporary hypnosis **2004**, 21(1), 28-34	RCT	Hypnosis	No oral conditions	Poor
19	Wager et al.,	Science (New York, N.Y.) **2004**, 303(5661), 1162-	CCS	fMRI	No oral conditions	Poor

		1167				
20	*Egner et al.,*	*NeuroImage.* ***2005****; 27:969-978*	*Observ. Cohort*	*EEG, fMRI*	*No oral conditions*	Fair
21	*Milling et al.,*	*Ann Behav Med* ***2005****, 29(2), 116-127*	*CSS*	*Hypnosis*	*No oral conditions*	Good
22	*Batty et al.,*	*Brain Res Bull,* ***2006****, 71(1-3), 83-90*	*RCT*	*EEG*	*No oral conditions*	Fair
23	*Eitner et al.,*	*Intl J of Clinical and Experimental Hypnosis.* ***2006****; 54(4):457-479*	*Comp. Interdiscip. Clinical Study*	*EEG, ECG, heart rate, blood pressure, blood oxygen saturation, respiration rate, salivary cortisol concentration, body temperature*	*Oral and maxillofacial treatments*	Good
24	*Saadat et al.,*	*Anesthesia and Analgesia* ***2006****, 102(5), 1394-1396*	*RCT*	*Hypnosis*	*No oral conditions*	Good
25	*Milling et al.,*	*Ann Behav Med* ***2007****, 33(2), 167-178*	*RCT*	*Hypnosis + cognitive-behavioral treatment*	*No oral conditions*	Fair
26	*De Pascalis et al.,*	*Pain.* ***2008****; 134(2008):197-208*	*CCS*	*EEG*	*No oral conditions*	Fair
27	*Marc et al.,*	*J Womens Health (Larchmt)* ***2009****, 18(9), 1441-1447*	*RCT*	*Hypnosis*	*No oral conditions*	Good
28	*Vanhaudenhuyse et al.,*	*Neuroimage* ***2009****, 47(3), 1047-1054*	*CSS*	*Thulium YAG laser fMRI*	*No oral conditions*	Good
29	*Krummenacher,*	*Schmerz (berlin, germany)* ***2010****, 148(3), 368-374*	*CCS*	*rTMS*	*No oral conditions*	Poor
30	*Miltner et al.,*	*International J of Psychophysiology* ***2010****, 77(3), 206-238*	*Article*	*EEG*	*No oral conditions*	Poor
31	*Brockardt et al.,*	*Pain* ***2011****, 152(1), 182-187*	*RCS*	*rTMS*	*No oral conditions*	Good
32	*Pyka et al.,*	*NeuroImage.* ***2011****; 56(2011), 2173-2182*	*CT*	*fMRI*	*No oral condition*	Poor
33	*Trehune et al.,*	*Psychophysiology.* ***2011****; 48(2011):1444-1447*	*Case-Control Study*	*EEG*	*No oral conditions*	Poor
34	*Zeidan et al.,*	*J Neurosci* ***2011****, 31(14), 5540-* [illegible]	*RCS*	*MRI*	*No oral conditions*	Good
35	*Stein et al.,*	*Pain* ***2012****, 153(11), 2210-2217*	*CCS*	*DTI, MRI*	*No oral conditions*	Good
36	*Hilbert et al.,*	*BioMed Research International.* ***2013****;*	*Case-Control Study*	*fMRI*	*Dental phobia*	Good
37	*Williams et al.,*	*The International J of Clinical and* [illegible]	*Observational Study*	*EEG*	*No oral conditions*	Good
38	*Dufresne et al.,*	*Int J Clin Exp Hypn* ***2014****, 58(1), 82-101*	*RCT*	*Hypnosis*	*No oral conditions*	Fair
39	*Jensen et al.,*	*Pain Med* ***2014****, 15(12), 2055-2063.*	*RCT*	*EEG*	*No oral conditions*	Fair
40	*Halsband et al.,*	*J of Physiology.* ***2015****; 109:131-142*	*CCS*	*fMRI*	*Dental phobia*	Good

41	*De Pascalis et al.,*	*PLoS One* ***2016****, 11(8)*	*Research*	*EEG, EMG, EOG*	*No oral conditions*	Good
42	*Jiang et al.,*	*Cerebral Cortex* ***2016****, 2016, 1-11*	*Observational Study*	*fMRI*	*No oral conditions*	Good
43	*Williams et al.,*	*Contemporary Clinical Trials,* ***2020****; 90(2020):105935*	*RCT*	*EEG*	*No oral conditions*	Good
